# Deciphering the Structural Basis of Eukaryotic Protein Kinase Regulation

**DOI:** 10.1371/journal.pbio.1001680

**Published:** 2013-10-15

**Authors:** Hiruy S. Meharena, Philip Chang, Malik M. Keshwani, Krishnadev Oruganty, Aishwarya K. Nene, Natarajan Kannan, Susan S. Taylor, Alexandr P. Kornev

**Affiliations:** 1 Biomedical Sciences, University of California, San Diego, La Jolla, California, United States of America; 2 Department of Chemistry and Biochemistry, University of California, San Diego, La Jolla, California, United States of America; 3 Department of Pharmacology, University of California, San Diego, La Jolla, California, United States of America; 4 Department of Biochemistry and Molecular Biology, University of Georgia, Athens, Georgia, United States of America; 5 Institute of Bioinformatics, University of Georgia, Athens, Georgia, United States of America; 6 Howard Hughes Medical Institute, University of California, San Diego, La Jolla, California, United States of America; Brandeis University, United States of America

## Abstract

Eukaryotic protein kinases (EPKs) regulate numerous signaling processes by phosphorylating targeted substrates through the highly conserved catalytic domain. Our previous computational studies proposed a model stating that a properly assembled nonlinear motif termed the Regulatory (R) spine is essential for catalytic activity of EPKs. Here we define the required intramolecular interactions and biochemical properties of the R-spine and the newly identified “Shell” that surrounds the R-spine using site-directed mutagenesis and various *in vitro* phosphoryl transfer assays using cyclic AMP-dependent protein kinase as a representative of the entire kinome. Analysis of the 172 available Apo EPK structures in the protein data bank (PDB) revealed four unique structural conformations of the R-spine that correspond with catalytic inactivation of various EPKs. Elucidating the molecular entities required for the catalytic activation of EPKs and the identification of these inactive conformations opens new avenues for the design of efficient therapeutic EPK inhibitors.

## Abstract 2

Biochemical and structural analysis of two features of kinase structure, the “R-spine” and “Shell,” afford a detailed insight into the regulation of eukaryotic protein kinases.

## Author Summary

Eukaryotic protein kinases (EPKs) have a highly conserved enzymatic kinase core that is involved in the regulation of numerous cell signaling processes through the transfer of a phosphate group from adenosine triphosphate (ATP) to more than 30% of human proteins. EPKs have been implicated in numerous human diseases, including cancer, cardiovascular diseases, and diabetes, making them one of the most sought-after therapeutic drug targets. The lack of structural diversity of the active kinase core has created a bottle-neck for designing successful therapeutic inhibitors. Here we describe the intramolecular interactions required for differentiating between the active and inactive states of EPKs. Kinases contain a hydrophobic regulatory spine (“R-spine”) that is disassembled in inactive kinases, and here we define an additional hydrophobic “Shell” that surrounds one end of the R-spine. Biochemical analysis of the five nonconsecutive R-spine residues and three nonconsecutive Shell residues shows that proper assembly of the R-spine and Shell is essential for maintaining kinase activity. Structural analysis of the 172 known structures of EPKs without bound ligands led to the identification of four inactive conformations that correlate with the disassembly of the R-spine. Understanding the molecular elements involved in the regulation of kinase activity and the identification of these diverse groups of inactive conformations should aid the design of more specific therapeutic EPK inhibitors.

## Introduction

Eukaryotic protein kinases (EPKs) phosphorylate a serine, threonine, or tyrosine residue in approximately 30% of human proteins and thus regulate numerous cellular and metabolic processes [1]. Abnormal catalytic activity of EPKs is implicated in numerous human diseases, including cancer, cardiovascular diseases, and diabetes. Therefore, EPKs are considered to be one of the most promising therapeutic drug targets. Of the more than 500 EPKs identified in the human genome, approximately 180 are associated with human diseases, either as causative agents or as therapeutic intervention points. Currently, 24 small molecule EPK inhibitors are FDA approved and numerous compounds are in clinical trials [2]. Some of the major challenges for designing efficient therapeutic drugs include the promiscuous nature of these inhibitors targeting multiple members of the family as well as patient relapse due to mutations that drive drug resistance [3].

**Figure 1:**
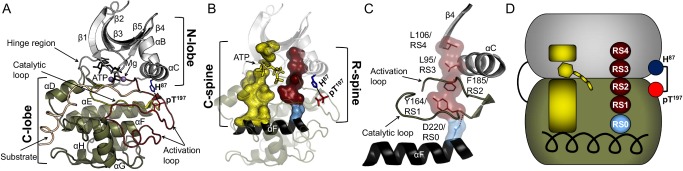
The architecture of EPKs. (A) The conserved EPK structural core is shown mapped on the catalytic subunit of PKA (PDB ID: 1ATP). The N-lobe (Grey) is mostly composed of β-sheets and the C-lobe (tan) is mostly α-helical. ATP (black) and two atoms of magnesium (purple) are bound in the cleft between the lobes. (B) R-spine (maroon) and C-spine (yellow) are bound to the large αF-helix (black) in the center of the C-lobe. They span the whole kinase core and the C-spine is completed by the adenine ring of ATP (yellow). Activation loop phosphorylation at residue T197 (pT197 (red)) is crucial for the complete activation of PKA, and pT197 forms a H-bond with H87 (blue) in the αC-helix from the N-lobe. (C) The different components of the R-spine are labeled as RS1 from catalytic loop (tan), RS2 from the activation loop (tan), RS3 from the αC-helix (grey), RS4 from the β4(grey), and is anchored by RS0 (light blue) from the αF-helix (black). (D) A cartoon representation of the R-spine and the major components of the EPK core.

EPKs have a highly conserved structural core that consists of two lobes: a small N-terminal lobe (N-lobe) and a larger C-terminal lobe (C-lobe) [4],[5]. The smaller, N-lobe is primarily involved in anchoring and orienting the nucleotide (Figure 1A). This lobe is predominantly constructed of antiparallel β-sheet structures that are unique among nucleotide binding proteins. A short loop known as the “hinge region” is the only structure that connects these two lobes. The deep cleft between the two lobes forms the active site where the phosphoryl transfer process occurs. Both the N- and C-lobes participate in the binding of ATP with 2 magnesium ions. The C-lobe binds the substrate, bringing it in close proximity to ATP, resulting in the phosphorylation of the substrate.

Previous computational analysis of EPKs proposed that the core is organized around three major elements (Figure 1B): a large hydrophobic αF-helix in the middle of the C-lobe and two nonlinear hydrophobic motifs termed “spines”: the Catalytic (C) spine and the Regulatory (R) spine [6],[7]. The spines are anchored to the αF-helix and secure the position of ATP, substrate, and amino acid residues that are important for catalysis. The spines are unusual structural motifs as they consist of amino acid residues that come from different parts of the EPK sequence and do not form a conventional sequence motif. A unique feature of the C-spine is that the adenine ring of ATP is part of this spine and connects the hydrophobic residues from the N- and the C-lobes (Figure 1B). The geometry of the R-spine is relatively stable as it remains intact throughout the phosphoryl transfer process.

Unlike other enzymes, EPKs are unique as they do not have a single active and inactive conformation [8]. The active state of the enzyme is highly dynamic where the core toggles between the open and closed conformations. The inactive state has traditionally been divided into two general groups defined by the positioning of the phenylalanine of the DFG motif from the activation loop [9]–[11]. If the DFG-phenylalanine moves far enough from its active position, it is classified as the “DFG-out” conformation, which is currently the major target for therapeutic drug design. The second inactive conformation known as the “DFG-in” conformation is when the phenylalanine does not move substantially from the active conformation. The most common inactive DFG-in conformation is caused by the movement of the αC-helix, but other less understood inactive conformations, not caused by the movement of the DFG-motif or αC-helix, also belong to this group.

Since the R-spine is a geometrically preserved motif that spans both lobes of all EPKs in the active state, we sought to elucidate the properties required for a catalytically functional R-spine (Figure 1B). Using an *E. coli* expression system, site-directed mutagenesis, Western blotting, and a radioactive phosphoryl transfer assay, we elucidated the biochemical and biophysical properties required for a catalytically viable R-spine using cyclic AMP-dependent protein kinase (PKA) as a model system. We identified three additional hydrophobic residues around the R-spine that we refer to as the “Shell,” which play a crucial role in supporting the R-spine's ability to maintain catalytic function. We experimentally tested the relationship between the phosphorylation state of the activation loop, the R-spine, and the catalytic activity in PKA. Additionally, qualitative structural analysis of 172 available Apo EPK structures from the protein data bank (PDB) lead to the identification of four distinct ways the R-spine is disassembled corresponding with catalytic inactivation of EPKs.

## Results

PKA is one of the well-studied EPKs, and therefore, it is often used as a prototype for understanding the biophysical and biochemical properties of the entire kinome. In PKA, the R-spine has two residues from the C-Lobe: tyrosine 164 (RS1) of the YRD/HRD motif from the catalytic loop and phenylalanine 185 (RS2) of the activation loop DFG motif (Figure 1C,D). It is completed by two N-lobe residues: leucine 95 (RS3) of the αC-helix and leucine 106 (RS4) of β4-strand. This nonlinear motif is anchored to the αF-helix through aspartic acid 220 (RS0). PKA has a phosphorylation site, threonine 197 (T197) on the activation loop, which is required for the completion of the catalytic activation process (Figure 1A,B,D) [12]. The phosphorylation of T197 (pT197) initiates an intricate hydrogen bond network observed in the C-lobe, which is required for substrate binding [13]. pT197 also forms a hydrogen bond (H-bond) with histidine 87 (H87) from the αC-helix, and previous studies demonstrated that mutating H87 to alanine (H87A) increases the catalytic activity by 2–3-fold [14]. T197 is a trans autophosphorylation site as well as a substrate for 3-phosphoinositide-dependent protein kinase 1 (PDK1) [15]. When PKA is expressed in bacteria, trans autophosphorylation of pT197 is a qualitative indicator for the catalytic activity of PKA [16]. The catalytic activity is negatively controlled by mutating glutamic acid 91 into an alanine (E91A), resulting in the loss of the salt bridge with the catalytic lysine 72, which is essential for the phosphoryl transfer process.

### Is the Aromatic or Aliphatic Property of the R-Spine Essential for Catalytic Activity?

**Figure 2:**
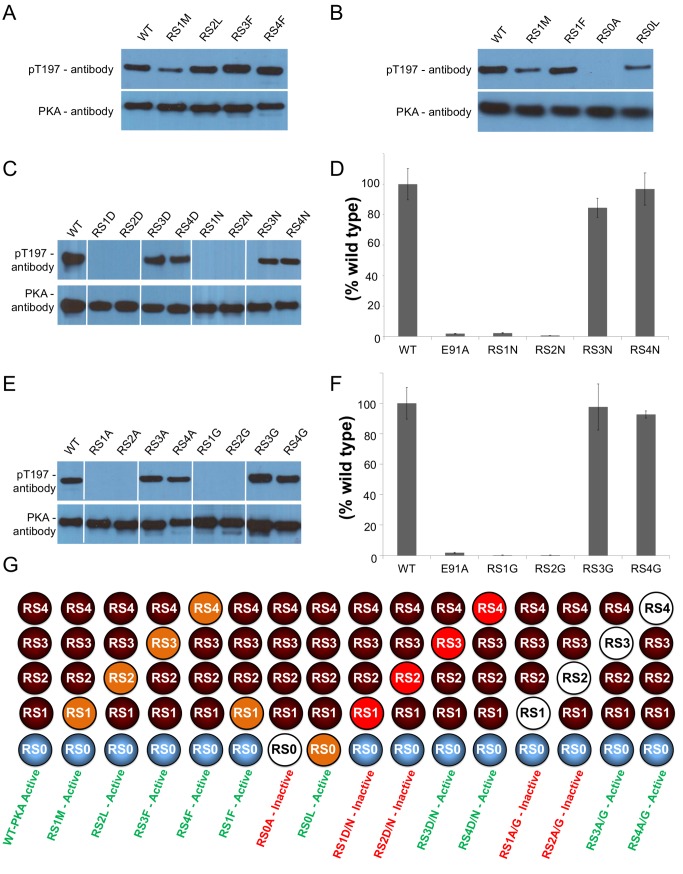
Understanding the properties required for a catalytically active R-spine. PKA mutants were expressed in *E. coli* and the catalytic activity was analyzed using Western blot assay to determine the effect of (A) aromatic and aliphatic properties of the R-spine and (B) specific interactions of RS1. A qualitative Western blot assay (C) and quantitative radioactive phosphoryl transfer assay (D) were carried out for PKA mutants containing hydrophilic R-spine mutations. A qualitative Western blot assay (E) and quantitative radioactive phosphoryl transfer assay (F) were carried out for PKA mutants containing removal of side chain atoms of the R-spine residues. (G) Cartoon summary of the R-spine mutants (orange circle represents introducing a hydrophobic residue, red circle represents introducing a hydrophilic residue, and white circle represents removal of side chain atoms) along with a summary of their catalytic activity (green label indicates active and red label indicates inactive).

In most EPKs the R-spine consists of two aromatic residues from the C-lobe and two aliphatic residues from the N-lobe. Alignment of more than 13,000 EPK sequences (Table S1) showed that RS1 is conserved as an aromatic residue in ∼99% of EPKs (Table 1). Previous studies in *Drosophila* Src64 showed that mutating RS1 from a histidine into a leucine does not eliminate the catalytic activity [17]. To examine whether PKA can tolerate an aliphatic residue instead of a tyrosine at the RS1 position, we inserted a mutation converting RS1 to a methionine (RS1M). The Western blot assay illustrates that the mutant remains catalytically active even though activity is reduced (Figure 2A,G). The second R-spine residue from the C-lobe, RS2, is conserved as a phenylalanine in approximately ∼90% of EPKs, but this residue is also a leucine (aliphatic) in about ∼6.7% of EPKs. To test if this naturally occurring variant is tolerable in PKA, we mutated RS2 to a leucine (RS2L). We observed that RS2L remains as catalytically active as the wild-type PKA (WT-PKA) (Figure 2A,G). The N-lobe residues RS3 and RS4, on the other hand, are conserved as aliphatic residues in approximately 90% and 80% of EPKs, respectively. In order to determine if PKA can tolerate an aromatic residue instead of an aliphatic residue, we individually mutated RS3 and RS4 from a leucine into a phenylalanine (RS3F and RS4F). The results show that RS3F and RS4F mutants have normal levels of catalytic activity in comparison to the WT-PKA (Figure 2A,G).

**Table 1:** Summary of the alignment of the more than 13,000 EPK sequences.

Amino acids	RS0	RS1	RS2	RS3	RS4	Sh1	Sh2	Sh3
***Aromatic***	***0.23***	***99.45***	***91.57***	***8.18***	***20.37***	***5.06***	***20.52***	***7.43***
***Aliphatic***	***99.77***	***0.55***	***8.43***	***91.82***	***79.63***	***94.94***	***79.48***	***92.57***
***Hydrophobic***	***0.3***	***8.71***	***99.67***	***89.37***	***96.31***	***89.68***	***82.37***	***98.24***
Alanine (A)	0.08	0.01	0.11	2.73	1.83	2.86	0.18	0.41
Cysteine (C)	0.01	0.01	0.04	1.15	1.5	1.31	0.51	0.25
Aspartic acid (D)	96.38	0	0.01	0.07	0.02	0.02	0.02	0.01
Glutamic acid (E)	0.43	0.02	0.01	0.09	0.04	0.08	0.41	0.17
Phenylalanine (F)	0	0.53	88.64	1.45	10.5	0.11	10.9	6.64
Glycine (G)	0.04	0	0.01	0.55	0.02	0.08	0.11	0.01
Histidine (H)	0.01	91.04	0.01	3.13	0.17	0	0.19	0.07
Isoleucine (I)	0	0.09	0.15	5.28	7.39	22.47	2.59	26.54
Lysine (K)	0.04	0.01	0.01	0.18	0.06	0.05	0.05	0.02
Leucine (L)	0.01	0.22	6.76	53.35	54.74	8.32	18.42	46.02
Methionine (M)	0	0	0.7	21.26	5.21	1.33	38.36	10.92
Asparagine (N)	0.44	0.07	0.1	0.75	0.07	0.2	0.23	0.02
Proline (P)	0.02	0.04	0.05	0.08	0.37	4.89	0.02	0.06
Glutamine (Q)	0.02	0.04	0	1.96	0.14	0.14	2.04	0.23
Arginine (R)	0.02	0.01	0.01	0.31	0.02	0.16	0.26	0.02
Serine (S)	0.07	0.01	0.01	0.83	0.52	0.39	1.31	0.11
Threonine (T)	2.13	0.02	0.07	1.51	0.76	3	12.49	0.81
Valine (V)	0.08	0.02	0.39	1.8	7.31	54.53	2.5	6.98
Tryptophan (W)	0	0.01	1.3	0.33	0.08	0.04	0.02	0.06
Tyrosine (Y)	0.21	7.83	1.61	3.19	9.26	0.02	9.4	0.67

Percentile of aromatic, aliphatic, hydrophobic (bold/italics), and representative amino acids for each R-spine and Shell residues from alignment of more than 13,000 EPK protein sequences.

### Which Interactions of RS1 Are Required for Catalytic Activity?

The RS1 residue is conserved as a histidine in ∼91% and tyrosine in ∼8% of EPKs (Table 1). The side chain of RS1 has the ability to interact with the neighboring amino acid residues in three different ways: first, the hydrophobic interaction of tyrosine with RS2, which we demonstrated to be sufficient for maintaining some catalytic activity (RS1M) (Figure 2B,G). Next is the CH-π interaction with RS2, which is conserved in approximately 90% of EPKs. To assess whether this interaction is sufficient for maintaining catalytic activity, we replaced RS1 with phenylalanine (RS1F). The Western blot results show that RS1F is sufficient for maintaining catalytic activity, and the introduction of the aromatic ring improves the catalytic activity when compared to RS1M (Figure 2B,G). Since RS1 is conserved as histidine (RS1H) in 91% of EPKs, we mutated RS1 from a tyrosine to histidine to see if these two residues were interchangeable, and Western blot analysis shows that the mutant remains catalytically active (Figure S1). Finally, there is the polar interaction of RS1 with RS0; here, the main chain of RS1 interacts with the side chain of RS0 and this interaction is conserved in more than 95% of EPKs. To examine if this polar interaction is required to maintain the proper assembly of the R-spine, we mutated RS0 to alanine (RS0A), and Western blot results show that catalytic activity was abolished (Figure 2B,G). This is consistent with recent studies on Aurora kinase, where mutation of RS0 to an alanine (RS0A) abolished Aurora kinase activity [18]. To test if loss of this polar interaction could be rescued through a hydrophobic interaction, we replaced RS0 with a leucine (RS0L); results show that some catalytic activity was rescued (Figure 2B,G).

### Is the Hydrophobic Property of the R-Spine Residues Mandatory for Catalytic Activity?

Based on sequence alignment (Table 1), the three R-spine residues (RS2, RS3, and RS4) are highly conserved as a hydrophobic residue, whereas only ∼8% of EPKs including PKA have a hydrophobic residue at the RS1 position. To address if the hydrophobic property is required for catalytic activity, we introduced the hydrophilic residues aspartic acid (RS1D, RS2D, RS3D, and RS4D) or asparagine (RS1N, RS2N, RS3N, and RS4N) to each of the four R-spine positions individually. Using Western blotting techniques and radioactive phosphoryl transfer assays, we discovered that the two C-lobe residues (RS1 and RS2) were highly sensitive to the introduction of a hydrophilic residue. Western blot analysis demonstrates that the catalytic activity was abolished when RS1 or RS2 were substituted with a hydrophilic residue (RS1D, RS2D, RS1N, and RS2N) (Figure 2C,G). Quantitative analysis of the hydrophilic noncharged asparagine mutation using the radioactive phosphoryl transfer assay confirmed that the catalytic activity of RS1N and RS2N was reduced by more than 95% (Figure 2D,G and Table S2). In contrast, when the N-lobe R-spine residues were mutated to hydrophilic residues (RS3D, RS4D, RS3N, and RS4N), the mutants remained catalytically active (Figure 2C,G). The enzyme retained 85% and 95% of its activity when the RS3 and RS4 positions were mutated to asparagine (RS3N and RS4N), respectively (Figure 2D,G and Table S2).

### Are the Side Chain Atoms of the R-Spine Residues Crucial for Catalytic Activity?

To evaluate whether the side chain of each R-spine residue is required for catalytic activity, we individually mutated each residue into an alanine or a glycine. After mutating the RS1 and RS2 (RS1A, RS2A RS1G, and RS2G), the catalytic activity was abolished, as illustrated by the Western blots (Figure 2E,G). The radioactive phophoryl transfer assay confirmed that the catalytic activity was reduced by more than 99% for RS1G and RS2G (Figure 2F,G and Table S2). However, the Western blots of RS3 and RS4 to alanine and glycine mutants (RS3A RS4A, RS3G, and RS4G) showed that these mutants had comparable levels of catalytic activity as the WT-PKA (Figure 2E,G). The quantitative data for RS3G and RS4G confirm that the catalytic activity was only reduced by ∼15% and ∼5%, respectively (Figure 2F,G and Table S2).

### Why Is the N-Lobe Region of the R-Spine Unaffected by the Alteration of the Side Chains?

**Figure 3:**
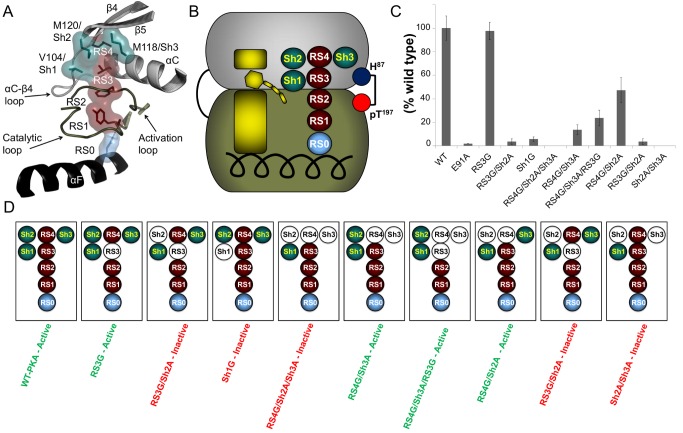
The role of the Shell for catalytic activity. (A) The 3-dimensional structure of the Shell (teal) is shown surrounding the R-spine (maroon) in PKA (PDB ID: 1ATP). (B) The R-spine and Shell are represented as a cartoon. (C) A radioactive phosphoryl transfer assay was carried out on various mutants elucidating the relation of the Shell with the R-spine and the required role for catalytic activity. (D) The catalytic activity of the PKA mutants is summarized as a cartoon representation for clarity (white circle represents removal of side chain atoms, green label represents active and red label represents inactive).

To understand why the catalytic activity was unaffected by the introduction of a hydrophilic residue or the removal of the side chain to the N-lobe region of the R-spine, we analyzed the amino acid residues that are within 4 Å of RS3 and RS4 in PKA. Looking at the previous Local Spatial Pattern (LSP) alignment data [7], only three out of the 14 amino acid residues surrounding RS3 and RS4 are highly conserved. We termed these three residues as the Shell, as they seemed to be supporting the N-lobe region of the R-spine (Figure 3A). In PKA these residues are valine 104 (Sh1), which is conserved as a hydrophobic residue in ∼90% of EPKs, the gatekeeper residue (methionine 120 (Sh2)), which is conserved as a hydrophobic residue in ∼82% of EPKs, and methionine 118 (Sh3), conserved as a hydrophobic residue in ∼98% of EPKs (Table 1 and Figure 3B). To understand the role of the Shell for catalytic activity, we made multiple mutations followed by radioactive phosphoryl transfer assays in PKA.

Above, we showed that RS3G has comparable catalytic activity to the WT-PKA (Figure 3C,D and Table S2). To destabilize this mutant, we decided to introduce an alanine mutation at the Sh2 position (RS3G/Sh2A). Results showed that the catalytic activity was significantly reduced by ∼96% (Figure 3C,D and Table S2). This indicates that RS3 is essential in the absence of Sh2. Next, to understand the role of Sh1 on the catalytic activity, we mutated Sh1 into a glycine (Sh1G). In the absence of the Sh1 side chain, the catalytic activity of the EPK was reduced by ∼94% (Figure 3C,D and Table S2). Sh1 is a crucial residue for catalytic activity as previous studies describe that the αC-β4-loop is crucial for anchoring the αC-helix [19].

To understand the significance of each of the three residues (RS4, Sh3, and Sh2), we mutated the RS4 residue to a glycine and SH2 and SH3 residues to an alanine (RS4G/Sh2A/Sh3A). This catalytically dead triple mutant serves as the reference point for understanding the role of each residue on the catalytic activity of the EPK (Figure 3C,D and Table S2). We then reintroduced each residue individually into the triple mutant to assay for rescue of catalytic activity. When returning Sh2 to a methionine in the triple mutant (RS4G/Sh3A), we were able to rescue ∼13% of the catalytic activity (Figure 3C,D and Table S2). Next, we mutated RS3 to a glycine (RS4G/Sh3A/RS3G) to address whether Sh2 could maintain activity through Sh1. Here we were able to rescue the catalytic activity by ∼23%, which indicates that Sh1 plays a role in maintaining a viable hydrophobic interaction between the N- and C-lobes through Sh2 (Figure 3C,D and Table S2). Next, we attempted to rescue some of the catalytic activity by mutating Sh3 back to a methionine in the triple mutant (RS4G/Sh2A), and we were able to rescue ∼47% of the catalytic activity (Figure 3C,D and Table S2). Above, we showed that in the absence of Sh2 and RS3, the catalytic activity was abolished, and this indicates that Sh3 requires the presence of RS3 to maintain catalytic activity (Figure 3C,D and Table S2). Finally, when we returned RS4 back to a leucine in the triple mutant (Sh3A/Sh2A), we were unable to recover any catalytic activity (Figure 3C,D and Table S2). This indicates that RS4 requires the presence of either Sh2 or Sh3 to maintain catalytic activity.

### What Is the Association Between the R-Spine and Phosphorylation of the Activation Loop?

**Figure 4:**
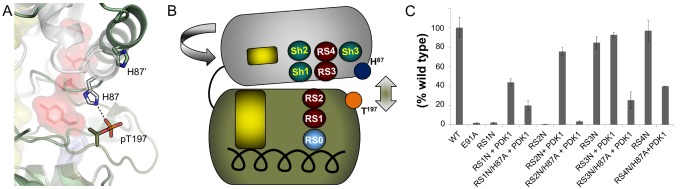
Phosphorylation of the activation loop is involved in stabilizing the assembled R-spine. (A) A comparison of the phosphorylated (PDB ID: 1ATP) and unphosphorylated (PDB ID: 4DFY) structures of PKA showed that the H-bond between pT197 and H87 is disrupted in the unphosphorylated state and the two lobes move away from each other and twist. (B) The inactivation of the C-subunit by twisting, separation of the two lobes, and disruption of the H-bond is shown as a cartoon representation. (C) The hydrophilic R-spine mutants were coexpressed with PDK1 to ensure complete activation loop phosphorylation and their activity measured using a radioactive phosphoryl transfer assay. Then H87A mutation was introduced to the hydrophilic R-spine mutants and coexpressed with PDK1 and activity was measured.

Complete activation of PKA is achieved after phosphorylation of T197 (pT197) on the activation loop [20]. pT197 initiates a major hydrogen bonding network in the C-lobe [13] and forms a H-bond between the activation loop and the αC-helix through H87. Previous studies showed that eliminating the H-bond between pT197 and H87 improves the catalytic activity by 2–3-fold [14]. We hypothesized that destabilization of the R-spine through hydrophilic mutations would cause disorientation of the N- and C-lobes, and this loss of catalytic activity can be rescued through the H-bond formed between pT197 and H87 (Figure 4A,B). To test this hypothesis we began by rescuing the catalytic activity of RS1N and RS2N by co-expressing these constructs with PDK1, which phosphorylates PKA on T197 and introduces the pT197-H87 H-bond. Using a radioactive phosphoryl transfer assay, we observed a ∼43% rescue of catalytic activity from ∼2% for RS1N and ∼75% rescue from less than 1% catalytic activity for RS2N (Figure 4C and Table S2). As a control we co-expressed RS3N with PDK1 (RS3+PDK1), and results show that the catalytic activity is comparable with RS3N. To understand if this rescue of function was due the intricate hydrogen bond network formed in the C-lobe or due to pT197-H87, we introduced the H87A mutation into all the asparagine mutants and co-expressed these double mutants with PDK1 (RS1N/H87A+PDK1, RS2N/H87A+PDK1, RS3N/H87A+PDK1, and RS4N/H87A+PDK1). The results from the radioactive phosphoryl transfer assay show that the catalytic activity for RS2N/H87A+PDK1 was reduced by ∼95% in comparison to RS2N+PDK1 and the catalytic activity for RS3N/H87A+PDK1 was reduced by ∼73% with respect to RS3N+PDK1 (Figure 4C and Table S2). However, the catalytic activity of RS1N/H87A+PDK1 and RS4N/H87A+PDK1 was reduced by ∼50% and ∼55%, when compared to RS1N+PDK1 and RS4N, respectively (Figure 4C and Table S2). Although the effect is not as drastic for RS1 and RS4, these results demonstrate that any instability of the R-spine affects the catalytic activity.

## Discussion

In 2006, the R-spine hypothesis for EPK regulation was proposed based on the computational comparison of 23 EPK structures [6]. Despite the lack of solid biochemical validation of this model, it quickly became popular and has been widely accepted as a framework for analysis of EPKs [21]–[24]. Nevertheless, many questions related to the properties of the R-spine residues remained unanswered. In this work we present the first systematic study of the R-spine in PKA, an EPK that has served as a prototype for the entire kinome for more than two decades. Our findings establish that the hydrophobic nature of the R-spine and the nonpolar CH-π interaction of RS1 with RS2 are mandatory for catalytic activity. The interaction of RS0 with RS1 is crucial for catalytic activity, but we demonstrate that a hydrophobic interaction can maintain the anchoring of the R-spine to the αF-helix. We also revealed that the N-lobe region of the R-spine is supported by a three-residue hydrophobic ensemble that we termed the “Shell” (Sh1, Sh2, and Sh3). The absence of Sh1 causes catalytic inactivation, indicating that the interaction of Sh1 with RS3 is crucial for anchoring the αC-helix in the active conformation. Sh1 is also capable of maintaining some catalytic activity in the absence of RS3 by completing the R-spine through the gatekeeper residue (Sh2). Previous studies showed that Sh2 and Sh3 are equally important for catalytic activity because either residue has the ability to compensate for the absence of the other, as in IL2-inducible T-cell kinase (Itk), and that at least one was mandatory for maintaining catalytic activity [25]. Here we confirm that the absence of Sh3 and Sh2 in PKA abolishes catalytic activity and returning either one enables the partial rescue of catalytic function. This is supported by the numerous disease-driving bulky hydrophobic single-nucleotide polymorphisms of the gatekeeper (Sh2) residue that boost activity [21]. The absence of a perfectly assembled R-spine results in loss of catalytic activity. However, this loss of function can be rescued by phosphorylating the activation loop, thus creating the pT197-H87 H-bond that stabilizes the assembled conformation of the R-spine.

**Figure 5:**
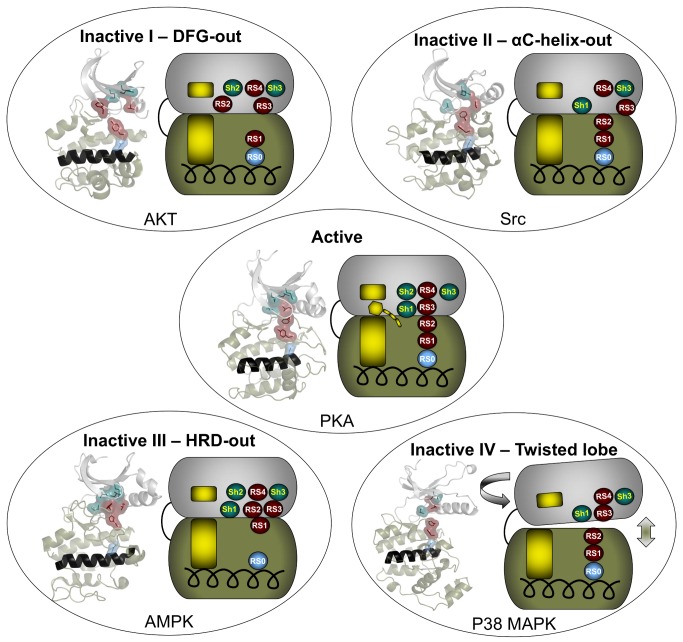
The R-spine and Shell configuration in the inactive state of EPKs. The four inactive conformations of the EPKs are shown with representative structures as well as cartoons in order to illustrate the configurations of the R-spine and Shell. The structures are inactive I (AKT; PDB ID: 1GZK), representing the DFG-out configuration; inactive II (Src; PDB ID: 1FMK), representing the C-helix out configuration; inactive III (AMPK; PDB ID: 3H4J), representing the HRD-out configuration; inactive IV (P38 MAPK; PDB ID: 1WFC), representing the twisted lobe configuration. The active EPK conformation (PKA; PDB ID: 1ATP) is shown for comparison (center).

Since the assembly of the R-spine is required for catalytic activity, we searched for naturally occurring disassembled conformations of the R-spine that correlated with catalytic inactivation. From the available 172 Apo EPK structures available in the PDB, we identified four different ways the R-spine can be disassembled corresponding to catalytically inactive EPKs (Table S3). The first two inactive groups were described as the DFG-out and DFG-in inactive conformations, respectively [9]. Inactive I or the DFG-out conformation is where the side chain of the RS2 of the DFG motif is misplaced from the active conformation as illustrated by the structure of Protein kinase B (AKT) [26] (Figure 5, Table S3). This inactive conformation was previously described in ABL kinase, and we were able to mimic this conformation in PKA through the RS2G mutant. Inactive II or the αC-helix out conformation, which was previously described in Src, occurs when RS3 is removed from the active conformation due to the αC-helix twisting out and moving away from the active site (Figure 5 and Tables S3 and S4) The RS3G+Sh2A mutant mimics this inactive conformation as the Sh2 residue in Src is the small hydrophilic residue threonine. In the inactive III or the YRD/HRD-out conformation, represented by 5′ AMP-activated protein kinase [27], we observe that RS1 from the YRD/HRD motif of the catalytic loop is no longer anchored to the αF-helix (Figure 5, Tables S3 and S4). We were able to mimic this conformation through the RS0A mutant in PKA. The last inactive conformation is the inactive IV or the twisted lobe conformation, which occurs when the two lobes move away from each other and twist, causing the R-spine to split in half (Figure 5, Tables S3 and S4). This conformation is represented by the structure of P38 mitogen-activated protein kinases [28]. Identification of the inactive I and inactive II conformations enabled the design of successful drugs such as Imatinib [29] and Lapatinib [30], respectively (Figure S2). We believe that the identification and the functional understanding of the R-spine and Shell will generate novel approaches to designing more efficient therapeutic EPK inhibitors as well providing insight towards understanding some of the disease-causing mutations.

## Materials and Methods

### Sequence Alignment

Representative EPK sequences from major taxonomic groups and families (∼13,690 sequences) were identified and multiply aligned using the MAPGAPS program [31]. The aligned columns were used to calculate amino acid frequencies at each of the R-spine positions (RS0–RS4) and the Shell positions (Sh1–3).

### Site-Directed Mutagenesis

QuikChange II site-directed mutagenesis kit (Agilent technologies) was used to introduce various mutations.

### Western Blot Phosphoryl Transfer Assay

The His6-tagged murine Cα-subunit of cAMP-dependent protein kinase (PKA) in pET15b was expressed in *E. coli* (BL21 (DE3)). Cultures were grown at 37°C to an A600 of ∼0.6 and induced with 0.5 mM isopropyl β-d-thiogalactopyranoside (IPTG). The cultures were allowed to grow overnight at 16°C before harvesting. The expression of PKA was confirmed using PKA C-subunit antibodies from BD Biosciences, and the phosphorylation state of the activation loop was confirmed using a polyclonal pT197 antibody from Invitrogen.

### Radioactive Phosphoryl Transfer Assay

The His6-tagged wild-type and mutation containing PKA in pET15b as well as mutants co-expressed with GST-tagged PDK1 were expressed in *E. coli* (BL21 (DE3)). Cultures were grown at 37°C to an A600 of ∼0.6 and induced with 0.5 mM IPTG. The cultures were allowed to grow overnight at 16°C before being harvested. The pellet was resuspended in lysis buffer (50 mM KH2PO4, 20 mM Tris-HCl, 100 mM NaCl, 5 mM β-mercaptoethanol, pH 8.0) and lysed using a microfluidizer (Microfluidics) at 18,000 p.s.i. The cells were clarified by centrifugation at 15,000 rpm at 4°C for 60 min in a Beckman JA20 rotor, and the supernatant was incubated with TALON His-Tag Purification Resin (Clontech) overnight at 4°C using gravity. The resin was washed twice (20× bed volume) with the lysis buffer and twice with using two different concentrations of imidazole in the wash buffer (50 mM KH2PO4, 20 mM Tris-HCl, 100 mM or 1 m NaCl, 50 mM/100 mM imidazole, and 5 mM β-mercaptoethanol, pH 7). A 250 mM imidazole elution buffer was used to elute the His-tagged protein (Figure S3A).

The kinetics was carried out with common reaction mix containing 50 mM MOPS pH 7.4, 1 mM Kemptide, 10 mM MgCl_2_, 1 mM ATP, and ^32^ γP radiolabelled ATP (specific activity 500–1,000 cpm/pmol) in a final volume of 20 µL. The reaction was initiated by addition of PKA with final concentration of 50 nM in a volume of 10 µl to 10 µl of the reaction mix described above. The reaction was carried out as an end point assay with 3 min as fixed time, and at the end-point the reaction was quenched with 90 µl of 30% Acetic acid. 50 µl of the quenched reaction was then spotted on p81 phosphocellulose paper, washed three times for 5 min each with 5% phosphoric acid, and finally washed with acetone (1×); air dried; and counted on liquid scintillation counter. The background counts were subtracted from the experimental time points and plotted to compare their activities. The wild-type protein was used as positive control and E91A mutant as the negative control. Each reaction was carried out in triplicates and data plotted are mean percent of WT-PKA ± relative standard error. The plots were made using MS Excel.

### Protein Folding

The His6-tagged murine Cα-subunit of PKA containing catalytically inactive mutants in pET15b was co-expressed with GST-tagged PDK1 in *E. coli* (BL21 (DE3)). PDK1 required properly folded PKA to phosphorylate PKA on the activation loop T197 [32]. Cultures were grown at 37°C to an A600 of ∼0.6 and induced with 0.5 mM IPTG. The cultures were allowed to grow overnight at 16°C before harvesting. The expression of PKA was confirmed using PKA C-subunit antibodies from BD Biosciences, and the phosphorylation state of the activation loop was confirmed using a polyclonal pT197 antibody from Invitrogen (Figure S3B).

## Supporting Information

Figure S1 Western blot analysis comparing the catalytic activity of WT-PKA and RS1H.
(TIF)

Figure S2 Inactive conformation stabilizing EPK inhibitors. (A) Imatinib (Gleevec) bound to Bcr-Abl tyrosine-kinase (3K5V.pdb) in the DFG-out conformation (Inactive I) and (B) Lapatinib (Tykreb) bound to Receptor tyrosine-protein kinase erbB-4 (3BBT.pdb) in the αC-helix-out conformation (Inactive II). (C) Table summarizing the R-spine and Shell residues in PKA, Bcr-Abl, and erbB4.
(TIF)

Figure S3 Purification and protein folding. (A) PKA mutants were purified on Talon resin and analyzed by SDS-PAGE and immunoblotting with anti-PKA antibody to check for the radioactive phosphoryl transfer assay. (B) The catalytically inactive PKA mutants were co-expressed with PDK1 and tested for proper folding by looking at the PKA expression levels and activation loop phosphorylation.
(TIF)

Table S1 Extract of an alignment of more than 13,000 sequences used for analysis of conservation of the R-spine (RS0, RS1, RS2, RS3, and RS4) and Shell (Sh1, Sh2, and Sh3) residues in EPKs. The R-spine residues are shown in red and in a larger font. The Shell residues are shown in cyan and in a larger font.
(PDF)

Table S2 Radioactive phosphoryl transfer assay of different PKA mutants. The activity is represented by the percent of catalytic activity of each mutant relative to the WT-PKA for triplicate experiments using a radioactive phosphoryl transfer assay and the standard error of each mutant.
(PDF)

Table S3 Summary of different conformations of the 172 Apo EPK structures. Number of EPK structures that belong to the active and four inactive conformations and the PDB IDs of the structures belonging to each group.
(PDF)

Table S4 Amino acid numbers of each R-spine and Shell residues for the representatives of each conformation. A list of the R-spine and Shell residues for PKA, AKT, Src, AMPK, and P38-MAPK.
(PDF)

## Abbreviations

EPK: eukaryotic protein kinase
R-spine: regulatory spine
RS0-4: R-spine residue 0–5
SH1–3: shell residue 1–3
