# Supplementary figures and images for: Deciphering the Structural Basis of Eukaryotic Protein Kinase Regulation
Source: PLoS Biol. 2013 Oct 15;11(10):e1001680. doi: 10.1371/journal.pbio.1001680 (PMC3797032; doi:10.1371/journal.pbio.1001680)

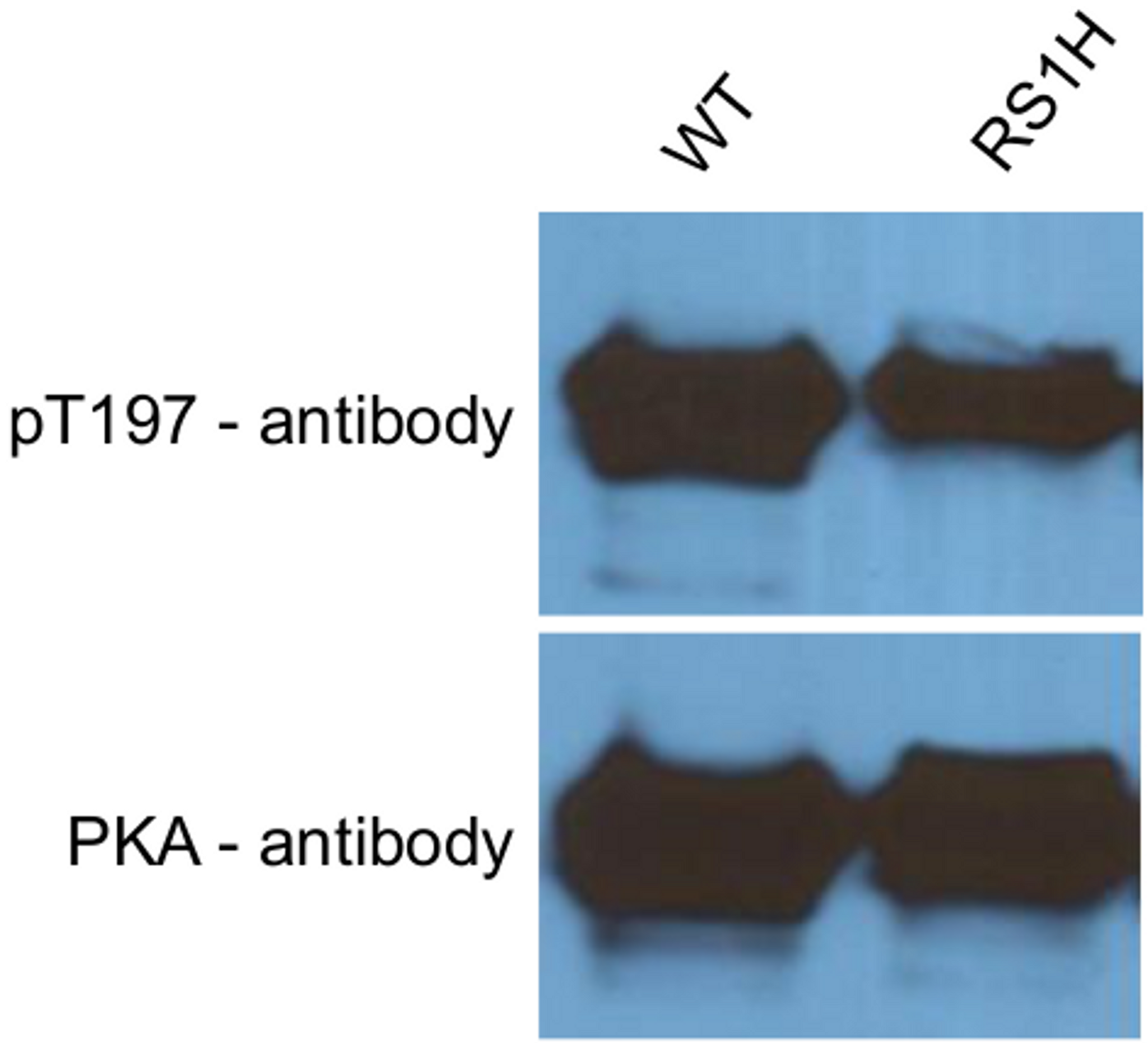

Supplement: Figure S1 — Western blot analysis comparing the catalytic activity of WT-PKA and RS1H. (TIF) [file pbio.1001680.s001.tif]

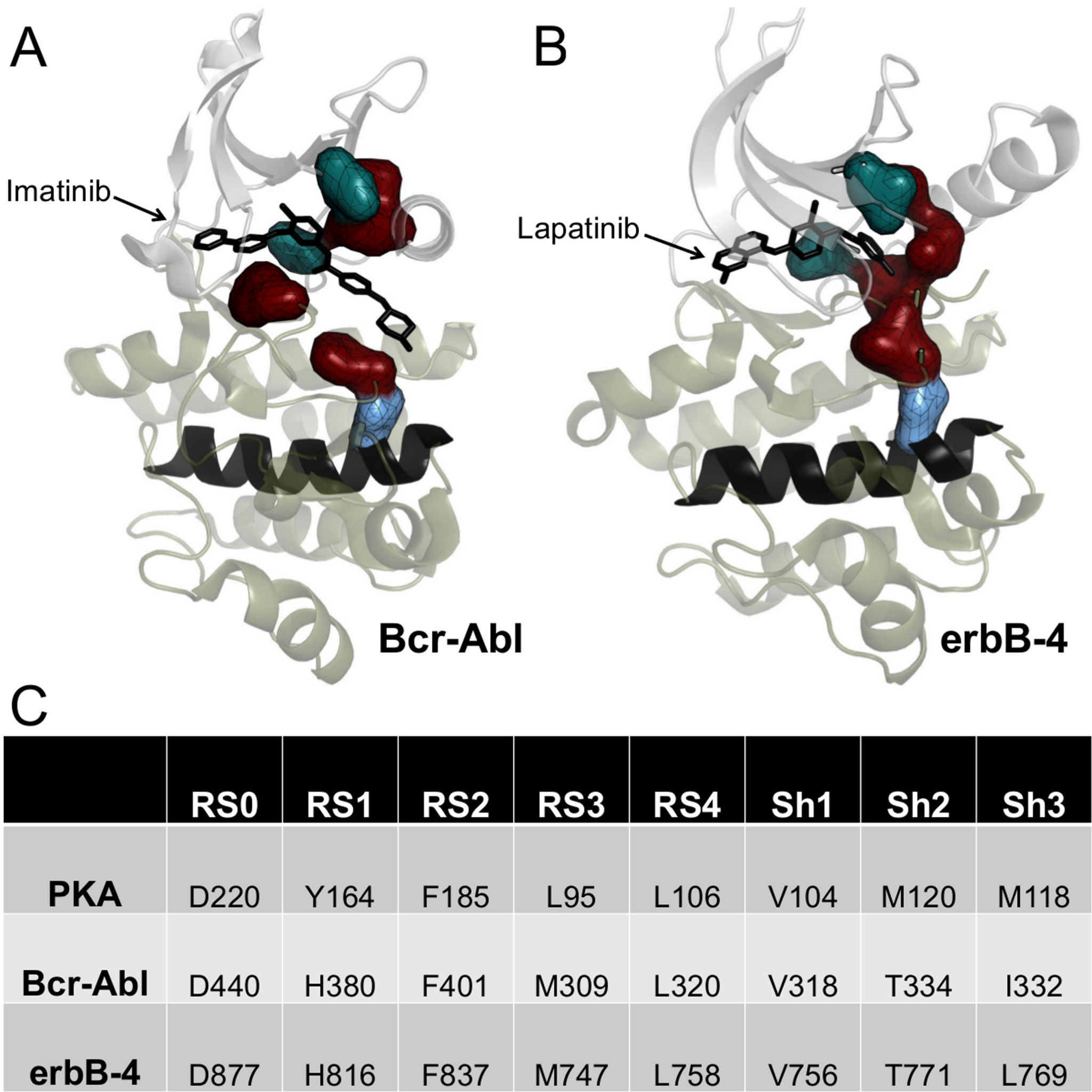

Supplement: Figure S2 — Inactive conformation stabilizing EPK inhibitors. (A) Imatinib (Gleevec) bound to Bcr-Abl tyrosine-kinase (3K5V.pdb) in the DFG-out conformation (Inactive I) and (B) Lapatinib (Tykreb) bound to Receptor tyrosine-protein kinase erbB-4 (3BBT.pdb) in the αC-helix-out conformation (Inactive II). (C) Table summarizing the R-spine and Shell residues in PKA, Bcr-Abl, and erbB4. (TIF) [file pbio.1001680.s002.tif]

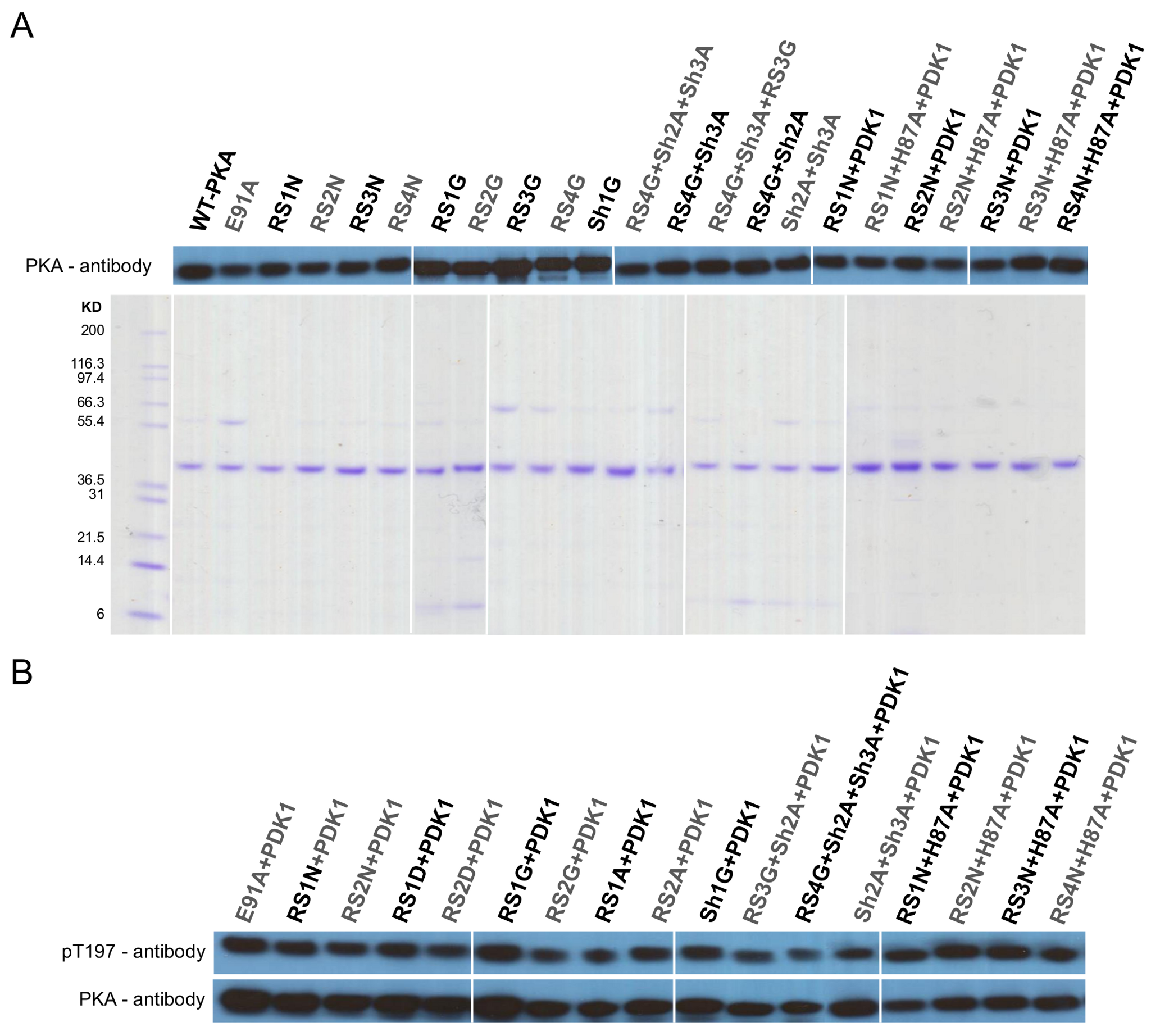

Supplement: Figure S3 — Purification and protein folding. (A) PKA mutants were purified on Talon resin and analyzed by SDS-PAGE and immunoblotting with anti-PKA antibody to check for the radioactive phosphoryl transfer assay. (B) The catalytically inactive PKA mutants were co-expressed with PDK1 and tested for proper folding by looking at the PKA expression levels and activation loop phosphorylation. (TIF) [file pbio.1001680.s003.tif]
