# Supplementary material for: Deciphering the Structural Basis of Eukaryotic Protein Kinase Regulation
Source: PLoS Biol. 2013 Oct 15;11(10):e1001680. doi: 10.1371/journal.pbio.1001680 (PMC3797032; doi:10.1371/journal.pbio.1001680)
Supplement: Table S1 — Extract of an alignment of more than 13,000 sequences used for analysis of conservation of the R-spine (RS0, RS1, RS2, RS3, and RS4) and Shell (Sh1, Sh2, and Sh3) residues in EPKs. The R-spine residues are shown in red and in a larger font. The Shell residues are shown in cyan and in a larger font. (PDF) [file pbio.1001680.s004.pdf]

protein gi code	RS3	Sh1.RS4	Sh3.Sh2	RS1	RS2	RS0
consensus	......QIMKQ	......IVRLY	......YIVME	......IIHRD	......CDFGL	......QSDWW......
157109822	......DIMAS	......ITSLQ	......YLVME	......YVHRD	......ADFGN	......TCDFW......
157137158	......DVLVF	......ITNLH	......YLVMD	......YVHRD	......ADFGS	......ECDWW......
157137156	......DVLVF	......ITNLH	......YLVMD	......YVHRD	......ADFGS	......ECDWW......
157130798	......DIMAS	......ITSLQ	......YLVME	......YVHRD	......ADFGN	......TCDFW......
157110310	......YIMAH	......IVQLH	......YMVMD	......FIHRD	......ADFGT	......ECDWW......
109018017	......DVLVN	......ITTLH	......YLVMD	......YVHRD	......ADFGS	......ECDWW......
109098938	......NILSR	......IPQLQ	......YLVME	......YVHRD	......VDFGS	......DCDWW......
109102096	......DIMAF	......VVQLF	......YMVME	......LIHRD	......ADFGT	......ECDWW......
109105846	......DVLVK	......VTTLH	......YLVMD	......YIHRD	......ADFGS	......QCDWW......
109121749	......DIMAF	......VVQLF	......YMVME	......FIHRD	......ADFGT	......ECDWW......
109121745	......DIMAF	......VVQLF	......YMVME	......FIHRD	......ADFGT	......ECDWW......
109125210	......DVLVN	......ITQLH	......YLVME	......YVHRD	......ADFGS	......ECDWW......
109125208	......DVLVN	......ITQLH	......YLVME	......YVHRD	......ADFGS	......ECDWW......
109459787	......DVLVK	......VTALH	......YLVMD	......YVHRD	......ADFGS	......QCDWW......
110750994	......DVLVY	......ITNLH	......YLVMD	......YVHRD	......ADFGS	......ECDWW......
114576237	......DIMAF	......VVQLF	......YMVME	......LIHRD	......ADFGT	......ECDWW......
114638316	......DVLVK	......VTTLH	......YLVMD	......YVHRD	......ADFGS	......QCDWW......
114647245	......NILSR	......IPQLQ	......YLVME	......YVHRD	......VDFGS	......DCDWW......
114647243	......NILSR	......IPQLQ	......YLVME	......YVHRD	......VDFGS	......DCDWW......
114654938	......DVLVN	......ITALH	......YLVMD	......YVHRD	......ADFGS	......ECDWW......
114672506	......DIMAF	......VVQLF	......YMVME	......FIHRD	......ADFGT	......ECDWW......
114672512	......DIMAF	......VVQLF	......YMVME	......FIHRD	......ADFGT	......ECDWW......
114672510	......DIMAF	......VVQLF	......YMVME	......FIHRD	......ADFGT	......ECDWW......
114672514	......DIMAF	......VVQLF	......YMVME	......FIHRD	......ADFGT	......ECDWW......
114672508	......DIMAF	......VVQLF	......YMVME	......FIHRD	......ADFGT	......ECDWW......
115729098	......DIMAH	......IVQLH	......YMVMD	......FIHRD	......ADFGT	......ECDWW......
115742102	......DIMAH	......IVQLH	......YMVMD	......FIHRD	......ADFGT	......ECDWW......
115803078	......DVLVH	......ITNLH	......YLVMD	......YVHRD	......ADFGS	......ECDWW......
115959258	......DIMAH	......IVQLH	......YMVMD	......FIHRD	......ADFGT	......ECDWW......
116283275	......DVLVK	......ITQLH	......YLVME	......YVHRD	......ADFGS	......ECDWW......
117616318	......NILSR	......IPQLQ	......YLVME	......YVHRD	......VDFGS	......DCDWW......
118086860	......DIMAF	......VVQLF	......YMVME	......FIHRD	......ADFGT	......ECDWW......
118087687	......DVLVN	......ITTLH	......YLVMD	......YVHRD	......ADFGS	......ECDWW......
118087693	......DVLVN	......ITTLH	......YLVMD	......YVHRD	......ADFGS	......ECDWW......
118087689	......DVLVN	......ITTLH	......YLVMD	......YVHRD	......ADFGS	......ECDWW......
118087691	......DVLVN	......ITTLH	......YLVMD	......YVHRD	......ADFGS	......ECDWW......
118089030	......DIMAF	......VVQLF	......YMVME	......LIHRD	......ADFGT	......ECDWW......
118092038	......NVLVN	......ITTLH	......YLVMD	......YVHRD	......ADFGS	......ECDWW......
118098817	......SILSQ	......IPQLQ	......YLVME	......YVHRD	......VDFGS	......ECDWW......
126131093	......DVLVN	......ITQLH	......YLVME	......YVHRD	......ADFGS	......ECDWW......
126091095	......DVLVN	......ITQLH	......YLVME	......YVHRD	......ADFGS	......ECDWW......
119577785	......DVLVN	......ITQLH	......YLVME	......YVHRD	......ADFGS	......ECDWW......
119577788	......DVLVN	......ITQLH	......YLVME	......YVHRD	......ADFGS	......ECDWW......
119594722	......DVLVK	......VTTLH	......YLVMD	......YVHRD	......ADFGS	......QCDWW......
119590212	......DVLVN	......ITTLH	......YLVMD	......YVHRD	......ADFGS	......ECDWW......
119590215	......DVLVN	......ITTLH	......YLVMD	......YVHRD	......ADFGS	......ECDWW......
119618563	......NILSR	......IPQLQ	......YLVME	......YVHRD	......VDFGS	......DCDWW......
119618562	......NILSR	......IPQLQ	......YLVME	......YVHRD	......VDFGS	......DCDWW......
119621342	......DIMAF	......VVQLF	......YMVME	......LIHRD	......ADFGT	......ECDWW......
119908233	......DVLVN	......ITTLH	......YLVMD	......YVHRD	......ADFGS	......ECDWW......
119908235	......DVLVN	......ITTLH	......YLVMD	......YVHRD	......ADFGS	......ECDWW......
119916226	......DIMAF	......VVQLF	......YMVME	......FIHRD	......ADFGT	......ECDWW......
124487319	......NILSR	......IPQLQ	......YLVME	......YVHRD	......VDFGS	......DCDWW......
125805206	......DIMAF	......VVQLF	......YMVME	......FIHRD	......ADFGT	......ECDWW......
125815425	......SILAL	......IPQLQ	......CLVME	......YVHRD	......ADFGW	......ESDWW......
125846503	......DICAF	......L-LC	......YLVME	......FIHRD	......ADFGT	......ECDWW......
126091040	......DVLVN	......ITQLH	......YLVME	......YVHRD	......ADFGS	......ECDWW......
126290183	......DVLVN	......ITTLH	......YLVMD	......YVHRD	......ADFGS	......ECDWW......
126306938	......DVLVN	......ITTLH	......YLVMD	......YVHRD	......ADFGS	......ECDWW......
126306936	......DVLVN	......ITTLH	......YLVMD	......YVHRD	......ADFGS	......ECDWW......
126303995	......DIMAF	......VVQLF	......YMVME	......LIHRD	......ADFGT	......ECDWW......
126324680	......SILCR	......IPQLH	......YLVME	......YVHRD	......VDFGA	......ECDWW......
126324682	......SILCR	......IPQLH	......YLVME	......YVHRD	......VDFGA	......ECDWW......
126324684	......SILCR	......IPQLH	......YLVME	......YVHRD	......VDFGA	......ECDWW......
126329573	......DVLVN	......VTQLH	......YLVME	......YVHRD	......ADFGS	......ECDWW......
134949013	......DIMAF	......VVQLF	......YMVME	......LIHRD	......ADFGT	......ECDWW......

```
147779786   ......DIMAH......IMAIH......YMAMD......YVHRD......ADFGT......ECDYW......
148666064   ......DIMAF......VVQLF......YMVME......LIHRD......ADFGT......ECDWW......
148681214   ......DVLVN......ITTLH......YLVMD......YVHRD......ADFGS......ECDWW......
148691158   ......DVLVK......ITQLH......YLVME......YVHRD......ADFGS......ECDWW......
148687893   ......NILSR......IPQLQ......YLVME......YVHRD......VDFGS......DCDWW......
148686692   ......DVLVN......ITALH......YLVMD......YVHRD......ADFGS......ECDWW......
148691159   ......DVLVK......ITQLH......YLVME......YVHRD......ADFGS......ECDWW......
148691160   ......DVLVK......ITQLH......YLVME......YVHRD......ADFGS......ECDWW......
148687892   ......NILSR......IPQLQ......YLVME......YVHRD......VDFGS......DCDWW......
148686691   ......DVLVN......ITALH......YLVMD......YVHRD......ADFGS......ECDWW......
149270272   ......DVLVK......VTALH......YLVMD......YVHRD......ADFGS......QCDWW......
114704212   ......DIMAF......VVQLF......YMVME......LIHRD......ADFGT......ECDWW......
149031714   ......DIMAF......VVQLF......YMVME......FIHRD......ADFGT......ECDWW......
149031715   ......DIMAF......VVQLF......YMVME......FIHRD......ADFGT......ECDWW......
149040859   ......DVLVN......ITTLH......YLVMD......YVHRD......ADFGS......ECDWW......
149044094   ......DVLVN......ITALH......YLVMD......YVHRD......ADFGS......ECDWW......
149040860   ......DVLVN......ITTLH......YLVMD......YVHRD......ADFGS......ECDWW......
149056808   ......DVLVK......ITQLH......YLVME......YVHRD......ADFGS......ECDWW......
149062168   ......DVLVK......VTALH......YLVMD......YVHRD......ADFGS......QCDWW......
149408635   ......DVLVN......ITTLH......YLVMD......YVHRD......ADFGS......ECDWW......
149625628   ......DIMAF......VVQLF......YMVME......FIHRD......ADFGT......ECDWW......
149641639   ......DVLVN......ITTLH......YLVMD......YVHRD......ADFGS......ECDWW......
149720545   ......NILSR......IPQLQ......YLVME......YVHRD......VDFGS......GCDWW......
149720547   ......NILSR......IPQLQ......YLVME......YVHRD......VDFGS......GCDWW......
149720835   ......DIMAF......VVQLF......YMVME......FIHRD......ADFGT......ECDWW......
149728145   ......DIMAF......VVQLF......YMVME......LIHRD......ADFGT......ECDWW......
149737731   ......DVLVN......ITTLH......YLVMD......YVHRD......ADFGS......ECDWW......
149748847   ......DVLVN......ITTLH......YLVMD......YVHRD......ADFGS......ECDWW......
149748764   ......DVLVN......ITTLH......YLVMD......YVHRD......ADFGS......ECDWW......
149748845   ......DVLVN......ITTLH......YLVMD......YVHRD......ADFGS......ECDWW......
149748803   ......DVLVN......ITTLH......YLVMD......YVHRD......ADFGS......ECDWW......
149748805   ......DVLVN......ITTLH......YLVMD......YVHRD......ADFGS......ECDWW......
156523116   ......DVLVK......VTALH......YLVMD......YVHRD......ADFGS......QCDWW......
157114479   ......TMLSG......IVCMT......CFILD......IVYRD......SDLGL......SADWF......
157112363   ......QILQK......VVNLA......CLVLT......IVYRD......SDLGL......SPDWF......
109049003   ......EILEK......IVSLA......CLVMS......IVYRD......SDLGL......PVDWF......
109079929   ......QILEK......VVSLA......CLVLT......IVYRD......SDLGL......SPDWW......
109079935   ......QILEK......VVSLA......CLVLT......IVYRD......SDLGL......SPDWW......
109079933   ......QILEK......VVSLA......CLVLT......IVYRD......SDLGL......SPDWW......
109079927   ......QILEK......VVSLA......CLVLT......IVYRD......SDLGL......SPDWW......
109079931   ......QILEK......VVSLA......CLVLT......IVYRD......SDLGL......SPDWW......
109093660   ......IMLSL......IVCMT......CFILD......VVYRD......SDLGL......SADWF......
109105422   ......IMLSL......IVCMS......SFILD......VVYRD......SDLGL......SADWF......
110759182   ......IMLSA......IVCMT......CFILD......IVYRD......SDLGL......SADWF......
114592962   ......RILEK......VVSLA......CLVLT......IVYRD......SDLGL......SPDWW......
114592972   ......RILEK......VVSLA......CLVLT......IVYRD......SDLGL......SPDWW......
114592964   ......RILEK......VVSLA......CLVLT......IVYRD......SDLGL......SPDWW......
114592974   ......RILEK......VVSLA......CLVLT......IVYRD......SDLGL......SPDWW......
114592968   ......RILEK......VVSLA......CLVLT......IVYRD......SDLGL......SPDWW......
114592970   ......RILEK......VVSLA......CLVLT......IVYRD......SDLGL......SPDWW......
114592966   ......RILEK......VVSLA......CLVLT......IVYRD......SDLGL......SPDWW......
114589563   ......EILEK......IVSLA......CLVMS......IVYRD......SDLGL......PVDWF......
114592960   ......RILEK......VVSLA......CLVLT......IVYRD......SDLGL......SPDWW......
114592958   ......RILEK......VVSLA......CLVLT......IVYRD......SDLGL......SPDWW......
114638767   ......IMLSL......IVCMS......SFILD......VVYRD......SDLGL......SADWF......
121582340   ......RILEK......VVSLA......CLVLT......IVYRD......SDLGL......SPDWW......
51873045f   ......RILEK......VVSLA......CLVLT......IVYRD......SDLGL......SPDWW......
117616410   ......QILEK......VVNLA......CLVLT......TVYRD......SDLGL......SPDYW......
118093105   ......QILEK......VVNLA......CLVLT......TVYRD......SDLGL......SPDYW......
118095069   ......EILEK......IVTLA......CLVMS......IIYRD......SDLGL......PVDWF......
118097365   ......QILEK......VVSLA......CLVLT......IVYRD......SDLGL......SPDWW......
118098625   ......IMLSL......IVCMT......CFILD......VVYRD......SDLGL......SADWF......
119594987   ......IMLSL......IVCMS......SFILD......VVYRD......SDLGL......SADWF......
119580102   ......IMLSL......IVCMT......CFILD......VVYRD......SDLGL......SADWF......
119580105   ......IMLSL......IVCMT......CFILD......VVYRD......SDLGL......SADWF......
119594988   ......IMLSL......IVCMS......SFILD......VVYRD......SDLGL......SADWF......
119894337   ......RILEK......VVSLC......CLVLT......IVYRD......SDLGL......SPDWW......
119605403   ......QILEK......VVSLA......CLVLT......IVYRD......SDLGL......SPDWW......
119605407   ......QILEK......VVSLA......CLVLT......IVYRD......SDLGL......SPDWW......
119605405   ......QILEK......VVSLA......CLVLT......IVYRD......SDLGL......SPDWW......
125824753   ......QILEK......VVSLA......CLVLT......IIYRD......SDLGL......SPDWW......
125828506   ......QILER......VVSLA......CLVLT......VVYRD......SDLGL......SVDWW......
```

125848675	......QILEK.....VVSLA.....CLVLT.....IVYRD.....SDLGL.....SPDWW......
126273369	......QILEK.....VVNLA.....CLVLT.....TVYRD.....SDLGL.....SPDYW......
126291608	......QILEK.....VVSLA.....CLVLT.....IVYRD.....SDLGL.....SPDWW......
126324522	......IMLSL.....IVCMT.....CFILD.....VVYRD.....SDLGL.....SADWF......
126332159	......RILEK.....VVSLS.....CLVLT.....IVYRD.....SDLGL.....SPDWW......
126337242	......KILEK.....IVSLA.....CLVMT.....IVYRD.....SDLGL.....SVDYF......
126338152	......EILEK.....IVTLA.....CLVMS.....IVYRD.....SDLGL.....PVDWF......
145699427	......IMLSL.....IVCMT.....CFILD.....VVYRD.....SDLGL.....SADWF......
148669903	......QILEK.....VVNLA.....CLVLT.....TVYRD.....SDLGL.....SPDYW......
148688009	......IMLSL.....IVCMT.....CFILD.....VVYRD.....SDLGL.....SADWF......
148688010	......IMLSL.....IVCMT.....CFILD.....VVYRD.....SDLGL.....SADWF......
148701097	......IMLSL.....IVCMS.....SFILD.....VVYRD.....SDLGL.....SADWF......
148709249	......QILEK.....VVSLA.....CLVLT.....IVYRD.....SDLGL.....SPDWW......
148709250	......QILEK.....VVSLA.....CLVLT.....IVYRD.....SDLGL.....SPDWW......
148709248	......QILEK.....VVSLA.....CLVLT.....IVYRD.....SDLGL.....SPDWW......
149057654	......RILAK.....IVSLA.....CLVMT.....IVYRD.....SDLGL.....SVDYF......
149039869	......QILEK.....VVSLA.....CLVLT.....IVYRD.....SDLGL.....SPDWW......
149039870	......QILEK.....VVSLA.....CLVLT.....IVYRD.....SDLGL.....SPDWW......
149063663	......IMLSL.....IVCMT.....CFILD.....VVYRD.....SDLGL.....SADWF......
149063661	......IMLSL.....IVCMT.....CFILD.....VVYRD.....SDLGL.....SADWF......
149431519	......QILER.....VVSLA.....CLVLT.....IVYRD.....SDLGL.....SPDWW......
149537413	......IMLSL.....IVCMS.....SFILD.....VVYRD.....SDLGL.....SADWF......
149634652	......QILEK.....VVNLA.....CLVLT.....TVYRD.....SDLGL.....SPDYW......
149635792	......KILAK.....IVSLA.....CLVMT.....IVYRD.....SDLGL.....SVDYF......
149641013	......RILEK.....VVSLS.....CLVLT.....IVYRD.....SDLGL.....SPDWW......
149690032	......QILEK.....VVNLA.....CLVLT.....TIYRD.....SDLGL.....SPDYW......
149720466	......IMLSL.....IVCMT.....CFILD.....VVYRD.....SDLGL.....SADWF......
149725624	......IMLSL.....IVCMS.....SFILD.....VVYRD.....SDLGL.....SADWF......
149726617	......QILEK.....VVSLA.....CLVLT.....IVYRD.....SDLGL.....SPDWW......
149729989	......EILER.....IVSLA.....CLVMS.....IVYRD.....SDLGL.....PVDWF......
149756995	......RILEK.....VVSLS.....CLVLT.....IVYRD.....SDLGL.....SPDWW......
149759598	......KILAK.....IVSLA.....CLVMT.....IVYRD.....SDLGL.....SVDYF......
157119785	......RVLKK.....LISLK.....CFVMQ.....IVYRD.....ADFGL.....AVDWW......
109020041	......RVLKN.....LTSLK.....CFVME.....IVYRD.....TDFGL.....AVDWW......
109085000	......RVLQN.....LTALK.....CFVME.....VVYRD.....TDFGL.....AVDWW......
109085004	......RVLQN.....LTALK.....CFVME.....VVYRD.....TDFGL.....AVDWW......
109085008	......RVLQN.....LTALK.....CFVME.....VVYRD.....TDFGL.....AVDWW......
109124744	......RVLQN.....LTALK.....CFVME.....VVYRD.....TDFGL.....AVDWW......
109124742	......RVLQN.....LTALK.....CFVME.....VVYRD.....TDFGL.....AVDWW......
109124756	......RVLQN.....LTALK.....CFVME.....VVYRD.....TDFGL.....AVDWW......
110761240	......RVLRT.....LISLK.....CFVME.....IIYRD.....ADFGL.....AVDWW......
169620806	......SVLAQ.....IVPLK.....YLVLA.....VIYRD.....CDFGL.....SVDWW......
111305899	......RVLKN.....LTSLK.....CFVME.....IVYRD.....TDFGL.....AVDWW......
115384658	......SVLAQ.....IVPLK.....YLVLA.....VIYRD.....CDFGL.....SVDWW......
115389310	......NILVR.....IVGLK.....YLVTD.....IVYRD.....CDFGL.....MVDFW......
114677301	......RVLQN.....LTALK.....CFVME.....VVYRD.....TDFGL.....AVDWW......
114677299	......RVLQN.....LTALK.....CFVME.....VVYRD.....TDFGL.....AVDWW......
114677297	......RVLQN.....LTALK.....CFVME.....VVYRD.....TDFGL.....AVDWW......
114677277	......RVLQN.....LTALK.....CFVME.....VVYRD.....TDFGL.....AVDWW......
115497974	......RVLKT.....LVKLH.....YLVLD.....IVYRD.....TDFGL.....TVDWW......
115696683	......RVLQK.....LTSLK.....CFVME.....VIYRD.....TDFGL.....AVDWW......
115954471	......RMVQK.....LASLE.....YFVME.....VIYRD.....TDFGL.....AVDWW......
116058825	......DILTA.....IVSLR.....YLILD.....IVHRD.....TDFGL.....GVDF-......
169856742	......TVLAL.....IVPLK.....YLVMS.....VVYRD.....CDFGL.....TVDWW......
169858586	......LVLAQ.....IVPLK.....YLVLA.....VVYRD.....CDFGL.....SIDWW......
169860621	......KILQS.....LVGLK.....YLVTD.....IVYRD.....CDFGL.....IVDFW......
169845511	......AVLKR.....VVKLW.....FLVMD.....VIYRD.....TDFGL.....EVDWW......
116830885	......EILSQ.....LPTLY.....CLVME.....IVYRD.....SDFDL.....AVDWW......
118572620	......RVLKN.....LTSLK.....CFVME.....IVYRD.....TDFGL.....AVDWW......
121702511	......NILVR.....IVGLK.....YLVTD.....IVYRD.....CDFGL.....MVDFW......
121715498	......SVLAQ.....IVPLK.....YLVLA.....VIYRD.....CDFGL.....SVDWW......
119481023	......SVLAQ.....IVPLK.....YLVLA.....VIYRD.....CDFGL.....TVDWW......
119496505	......NILVR.....IVGLK.....YLVTD.....IVYRD.....CDFGL.....MVDFW......
119577345	......RVLQN.....LTALK.....CFVME.....VVYRD.....TDFGL.....AVDWW......
123379254	......DVLLV.....IVHFC.....YLGLE.....IVYRD.....TDFGL.....SVDIW......
123398078	......NILTK.....IVSLR.....YLGLE.....IVYRD.....TDFGL.....AVDWW......
123397662	......NTLIN.....IVKLF.....YLVLE.....IIYRD.....TDFGF.....AVDWW......
123402993	......NILSK.....IISLK.....YLGLE.....VVYRD.....TDFGL.....EVDWW......
123404560	......NTLIN.....IVKLF.....YLVLE.....IIYRD.....TDFGF.....AVDWW......
123407797	......DVLIM.....VVKIC.....YIGLE.....IIYRD.....SDFGL.....ELDWW......
123405881	......NILFK.....IVKMK.....YLGME.....VVYRD.....CDFGL.....PVDWW......

```
123400474      ......NIMMK.....VVNLC.....YLGLE.....IIYRD.....TDFGL.....KIDEW......
123407171      ......NNLMR.....IVRIK.....YIGME.....IVYRD.....VDFGL.....QIDWW......
123401372      ......NTLTK.....VVNIL.....YIGME.....IVYRD.....ADFGL.....SVDWW......
123422323      ......NTLMK.....IVHIY.....YMGFE.....IIYRD.....TDFGL.....KIDWW......
123433133      ......DLLLK.....LVGAH.....FMILD.....YIYRD.....TDFGL.....AVDWW......
123426269      ......DVLLK.....LVSAR.....FLVLD.....ICHRD.....TDFGL.....NVDWW......
123425520      ......DILKS.....IVSLL.....YLVLE.....IIYRD.....TDFGI.....AIDWW......
123428717      ......EILKT.....IVSLL.....YLVLE.....IIYRD.....TDFGI.....AIDWW......
123431521      ......NALMR.....ITRLF.....YFVME.....IVYRD.....ADFGL.....AVDWW......
123438459      ......NLLFS.....IISAE.....YIGLE.....IVYRD.....TDFGL.....KVDWW......
123440214      ......DVLLS.....LVSAR.....FLVLD.....IMHRD.....TDFGL.....SVDWW......
123448976      ......NVVLK.....LISAR.....FLVLD.....ICYRD.....TDFGL.....SVDWW......
123457208      ......NVLVR.....IVSLK.....YFAME.....IIYRD.....TDFGL.....AVDWW......
123454460      ......NIMMK.....IVNLC.....YLGLE.....IIYRD.....TDFGL.....KIDEW......
123457243      ......NFLMR.....LVSLY.....YLGLE.....IIYRD.....VDFGL.....NVDEW......
123452218      ......NILVK.....IVNIK.....YIGME.....IVYRD.....TDFGL.....MIDWW......
123454963      ......NILMH.....IVHIY.....YMGFE.....IVYRD.....TDFGL.....KIDWW......
123477265      ......NILSK.....IVALR.....YLGLE.....IVYRD.....ADFGL.....SVDWW......
123475971      ......NIMMR.....LVNLC.....YLGLE.....IVYRD.....TDFGL.....AIDEW......
123473345      ......NIMIK.....IVQLK.....YLGLE.....VIYRD.....TDFGL.....SVDWW......
123476080      ......NILSV.....IVTMY.....YIGLE.....ILYRD.....TDFGL.....EIDWW......
123475679      ......NVLVR.....IVSLK.....YFAME.....IIYRD.....TDFGL.....AVDWW......
123468962      ......NIMMK.....VVNLC.....YLGLE.....IIYRD.....TDFGL.....KIDEW......
123471010      ......NLMMK.....LVSLH.....YFGLE.....IVYRD.....VDFGL.....AVDEW......
123499825      ......NVLLK.....IVSLS.....YLGLE.....VIYRD.....TDFGL.....AIDWW......
123488204      ......NVLLK.....LVSAH.....FMVID.....FVYRD.....TDFGL.....AVDWW......
123479981      ......NILSK.....IVQLH.....YLCME.....IVYRD.....TDFGS.....KVDWW......
123499371      ......NVLMK.....IVSLK.....YLGLE.....VIYRD.....TDFGL.....AIDWW......
154412131      ......NIMVK.....IVQLK.....YLGLE.....IIYRD.....TDFGL.....AIDWW......
123505904      ......TSFLK.....FNPIK.....KLFYE.....IIYKD.....TDPGL.....SADWY......
123489768      ......NILLL.....IVGIK.....YLGLE.....VIYRD.....TDFGL.....EIDWW......
123505776      ......NVLLT.....LVCAH.....FMVLD.....FIYRD.....TDFGL.....AVDWW......
123479676      ......NILMK.....IVNLC.....YLGLE.....IVYRD.....TDFGL.....KVDEW......
123486538      ......NSLMK.....IVRLC.....YLGLE.....IVYRD.....TDFGL.....GVDWW......
154422241      ......NILAK.....IVNLK.....YLGLE.....VIYRD.....TDFGL.....SVDWW......
154413613      ......NILMK.....IVQLF.....YLGLE.....IIYRD.....TDFGL.....KIDWW......
154421281      ......NILMK.....IVSLS.....YLGMQ.....IIYRD.....TDFGL.....AIDWW......
154413412      ......DVLLK.....IVSAK.....FLVLD.....VVHRD.....TDFGL.....AVDWW......
154420633      ......EILMK.....IIKLL.....YLVLE.....IIYRD.....TDFGI.....AVDWW......
154414554      ......NTLMR.....IIHLF.....LFVLE.....IIYRD.....ADFGT.....AIDWW......
145474201      ......DIMVR.....IIKLH.....AFVLE.....IVYRD.....SDFGL.....LVDCY......
145475205      ......NILTS.....LVNLI.....YLTLD.....IIHRD.....TDFGL.....AVDYF......
145475591      ......KILEK.....VVKLR.....YFIVD.....VIYRD.....SDFGL.....ACDFY......
145475099      ......ILLQK.....LVNMR.....YIVLD.....VLHRD.....TDMGI.....ACDYF......
145479085      ......NVLSV.....IVKLR.....FMILD.....IIFRD.....TDFGL.....AVDWY......
145480227      ......DIMTV.....INKLE.....VFVLE.....VVYRD.....ADFGL.....QVDHY......
145476171      ......FLKSY.....IEQLE.....HFLFK.....ITYGD.....TDFGY.....MSDWY......
145479535      ......KMLRE.....LVNIK.....YYQMK.....IVYGD.....AYLGL.....SVDVY......
145477507      ......EILIQ.....IINLH.....YFVFD.....IIYRD.....VDFGL.....SLDYY......
145480023      ......KILEI.....ITKLH.....YLIME.....IIYRD.....CDFGL.....SCDWW......
145476281      ......KVLEY.....VIRLQ.....YLVMD.....VIYRD.....TDFGL.....TVDLW......
145475967      ......NLLAQ.....LINMN.....YLVMD.....IIHRD.....TDLGI.....AVDYF......
145480435      ......DIMTV.....IIKLE.....VFVLE.....VVYRD.....ADFGL.....QVDHY......
145480379      ......IILEK.....ISKLH.....YYVID.....ILYRD.....IDFGL.....SCDWW......
145478283      ......NILSY.....IVKLR.....CLLMD.....IIYRD.....TDFGL.....AVDWY......
145476037      ......NIMVA.....LCKLL.....CFALE.....IIYRD.....ADFGL.....QIDHY......
145476955      ......KILEQ.....LVSLE.....YFVMK.....IIYRD.....TDYGL.....TADWW......
145484611      ......KILTY.....IVKLH.....YMILD.....IVYRD.....TDFGL.....PLDWY......
145485719      ......TILNQ.....IVYPQ.....YFVSE.....IIYRD.....TDFGL.....KVDCW......
145483513      ......QLLSN.....LINMQ.....YLVMD.....ILHRD.....TDLGI.....AVDYF......
145482783      ......YVMEQ.....LAKLF.....VFVME.....IVYRD.....SDFGL.....MVDIY......
145483263      ......YLLEH.....LVNMW.....FLIID.....IIHRD.....TDLGI.....VADYY......
145485303      ......RLLQN.....IVNAI.....YLVLD.....IIHRD.....TDLGV.....PADYY......
145482527      ......DVMVN.....ITPLH.....AFVLD.....IVYRD.....SDFGL.....LVDCY......
145484803      ......IILAT.....IVKLR.....YLVVD.....IIYRD.....TDFGL.....TCDWW......
145481011      ......LVLEH.....LVNLH.....FFVTQ.....IIYRD.....TDFGM.....SADWW......
145486166      ......ILLSK.....IANIH.....YLVMD.....IIHRD.....TDFGI.....GVDHF......
145483493      ......DILIQ.....TNQIE.....VFGIE.....IIYRD.....ADFGL.....LVDYY......
145482817      ......DILIE.....TSKIQ.....IFGID.....MIYRD.....ADFGL.....LVDYY......
145480935      ......NVLKQ.....IVKLK.....FFVME.....VIYRD.....TDFGL.....EVDIW......
145481185      ......NVLSV.....IVKLR.....FMILD.....IIFRD.....TDFGL.....ALDWY......
145482965      ......NIMTL.....LLHLS.....VFVLE.....ILYRD.....ADFGL.....TVDFY......
145482807      ......TLLSG.....LINMQ.....YLVMD.....VLHRD.....TDLGI.....AVDYF......
```

145480915	......ILLSE......LVNMN......YLIMD......IIHRD......TDFGI......AVDYY......
145484292	......NVLSV......IVKLR......FMILD......IIFRD......TDFGL......AVDWY......
145487971	......QLLQK......IINMV......YLILD......VIHRD......TDFGI......CVDYY......
145488496	......QILTQ......IISLH......YFIFD......IIYRD......IDFGL......TIDYY......
145486359	......EVLSV......IVKLN......FLVMD......IIFRD......TDFGL......MADWY......
145490453	......IILAT......LVRLR......YLVVD......IIYRD......TDFGL......TCDWW......
145489699	......NILAL......IVNLY......YFVID......IIYRD......TDFGL......SADWY......
145487516	......DIMVR......VTPLQ......AFVLD......IVYRD......SDFGL......LVDCY......
145488667	......NLLAQ......LINMN......YLVMD......IIHRD......TDLGI......AVDYF......
145488611	......NIMVA......LCKLL......CFALE......IIYRD......ADFGL......QIDHY......
145488149	......MLLSQ......LINMY......YLVMD......IIHRD......TDLGI......AVDYY......
145490754	......QILTR......IINLY......YFVFD......IIYRD......ADFGL......TQDYY......
145491654	......MLLSQ......LINMY......YLVMD......IIHRD......TDLGI......AVDYY......
145494189	......EVLSI......IVKLN......FLVMD......IIFRD......TDFGL......MADWY......
145490618	......ILLSK......IANIH......HLVMD......IIHRD......TDFGI......GVDHF......
145492276	......DIMTV......IIKLE......VFVLE......VVYRD......ADFGL......QVDHY......
145490935	......DVLKM......IVKLN......YLVMD......IIFRD......TDFGL......QVDWY......
145495079	......DIMVH......LCKLL......CFAME......VIYRD......ADFGL......QIDHY......
145493754	......FVLKS......IVKLR......YFVMD......IIYRD......TDFGL......EVDFW......
145492656	......RLLEN......IVNAV......YLVLD......IIHRD......TDLGV......PADYY......
145494564	......QILQQ......IIKLY......FLGTE......IIYRD......TDFGL......CVDWY......
145494852	......NVLSV......IVRLH......FMVLD......IIFRD......TDFGL......TVDWY......
145495009	......----Y......LIPLE......YFIMP......IVHGD......CDFGY......YSDYW......
145499588	......KILQV......IIPLY......CFLME......VLYRD......TDFGL......PADFY......
145497511	......YLLSN......LVNMH......YLVMD......VIHRD......TDLGI......ASDYF......
145498867	......NLLAQ......LINMN......YLVMD......IIHRD......TDLGI......AVDYF......
145496422	......NILSY......IVKLR......CLLMD......IIYRD......TDFGL......AVDWY......
145497041	......MLLSQ......LINMY......YLVMD......IIHRD......TDLGI......AVDYY......
145498590	......NILVM......IVNLH......YIVID......IIYRD......TDFGL......TADWY......
145495728	......IILEK......ISKLH......YYVID......ILYRD......IDFGL......SCDWW......
145499198	......DIMTV......MIKLE......-----......VVYRD......SKLNM......QVDHY......
145497585	......NILSN......LVNIY......FLALD......IIHRD......TDLGI......SVDYF......
145495884	......KILSQ......VIPLY......CFLME......ILYRD......TDFGL......PADFY......
145499164	......FILQH......ITKLH......YLIMD......ILYRD......IDFGL......QCDWW......
145499564	......DIMTV......IIKLE......IFVLE......IVYRD......ADFGL......QVDHY......
145497025	......YLLEH......LVNMS......YLIID......IIHRD......TDLGI......VADYY......
145502751	......SVLIR......VVKLK......YLVME......VVYRD......TDFGL......SVDWW......
145501590	......YLLTN......LVNMH......YLVMD......VLHRD......TDLGI......ASDYF......
145503884	......IVLQH......LVSLN......FFVTQ......IIYRD......TDFGM......AADWW......
145503770	......DIMSN......IVGLQ......VFVLE......IIYRD......ADFGL......QLDLY......
145500462	......AILEN......IVQLH......YLIMD......IIYRD......TDFGL......CVDWW......
145501846	......FILQK......IIKMY......NMVME......VAYRD......ADMGL......SVDFW......
145504164	......NLLAQ......LINMN......YLVMD......VIHRD......TDLGI......AVDYF......
145502875	......NILSN......LVNIY......FLVLD......SLHRD......TDLGI......AVDYF......
145502301	......KILEQ......LVSLE......YFVMK......IIYRD......TDYGL......TADWW......
145503958	......EVLNN......VVNLK......YFVLE......IIYRD......TDFGL......EVDLW......
145505597	......NILQN......IIKLE......YFCLQ......IIYRD......TDFGF......AADWW......
145509344	......NILKQ......IVNLF......YLVMD......IIYRD......VDFGL......ATDVY......
145506841	......NVLEY......VIKLQ......YLVME......VIYRD......TDFGL......TVDWW......
145507476	......DIMIK......IIPLH......AFVLD......IVYRD......SDFGL......LVDCY......
145505287	......DIMVK......IIPLH......AFVLE......IVYRD......SDFGL......LVDCY......
145506909	......KILSN......IVPLY......CFLME......VLYRD......TDFGL......PADFY......
145506921	......----Y......MEQLE......HFFFK......TIYGD......TDFGF......MSDWY......
145506034	......DIMTK......VTPLH......AFVLE......IVYRD......SDFGL......LVDCY......
145509234	......ILLSQ......IANIH......YLVLD......IIHRD......TDFGI......GVDHF......
145509038	......STLQT......IMHFV......YFLEE......IIYRD......IDLGT......SVDLW......
145504677	......QLLSQ......IVNMN......YLVMD......IIHRD......TDFGI......AVDYF......
145506735	......NVLVS......IIKLA......YFVLE......IIYRD......TDFGL......PVDWW......
145508115	......SILSV......IVKLH......FLVMD......IMYRD......TDFGL......SVDWY......
145509214	......NVLKQ......IVQLK......YLVME......VIYRD......TDFGL......EVDLW......
145507786	......NILQN......IIKLE......YFCLQ......IIYRD......TDFGF......AADWW......
145510150	......KIMSL......SVKLH......YMVMD......IIYRD......TDFGL......AVDFY......
145513028	......NILVE......IIKMF......YFVLE......IIYRD......TDFGL......PVDWW......
145513230	......MVLEL......VMQFV......YFLVQ......IVYRD......IDLGT......HVDLW......
145513574	......QILEV......IVKLH......YLVTD......IIYRD......TDFGL......SADWW......
145510624	......DIMVK......ITPLH......AFVLD......IVYRD......SDFGL......LVDCY......
145513104	......KLLEE......LISLE......FFVMK......VIYRD......SDYGL......EVDWW......
145512633	......TLLSQ......LVNMN......YLLMD......IIHRD......TDFGI......AVDYF......
145513250	......QILKT......IVRLY......YLATE......ILYRD......TDFGL......ATDIY......
145512285	......DVLKV......IVKLN......YLVMD......IIFRD......TDFGL......QVDWY......

```
145510528    ......DIMSQ......IVKMQ......VFVLE......IIYRD......ADFGL......QLDLY......
145511812    ......KLLEI......IIKLV......IYVLP......YVYRD......TDLGL......MIDWW......
145515803    ......QLLQK......IINMV......YLILD......VIHRD......TDFGI......CVDYY......
145514526    ......NVLSV......IVRLH......FMVLD......IIFRD......TDFGL......TVDWY......
145515481    ......DIMTK......VTPLH......AFVLE......IVYRD......SDFGL......LVDCY......
145521544    ......KILQV......IIPLY......CFLME......VLYRD......TDFGL......PADFY......
145517931    ......KILSN......IVPLY......CFLME......VLYRD......TDFGL......PADFY......
145520741    ......MILEH......LVNLA......FFIMQ......IVYRD......TDFGM......TADWW......
145520166    ......DIMCL......VVKMY......VFVLE......IIYRD......ADFGL......QLDLY......
145523227    ......TILNQ......IVSLS......YFVLE......IIYRD......TDFGL......KVDCW......
145522388    ......TVLEQ......IMGFI......YFLVE......IVYRD......IDLGL......NVDLW......
145522159    ......TLLSQ......LVNMN......YLLMD......IIHRD......TDFGI......AVDYF......
145523936    ......EILKS......IIKSY......YIIME......ILYRD......IDFGL......ASDIY......
145523852    ......HILSN......LVNIY......FLVLD......SLHRD......TDLGI......AVDYF......
145521845    ......DVLKI......IVKLN......YLVMD......IIFRD......TDFGL......QVDWY......
145523219    ......NILSN......LVNIY......FLVLD......SLHRD......TDLGI......AVDYF......
145523940    ......SVLIR......VVKLK......YFVME......VIYRD......TDFGL......SVDWW......
145525493    ......DIMAF......TIQML......VFILE......IMYRD......SDFGL......QVDHY......
145527126    ......YLLCN......LVNMH......YLVMD......ILHRD......TDLGI......ASDYF......
145525324    ......QILQC......LVELK......YMVVN......IIYRD......TDFGL......EVDYY......
145527678    ......NILSQ......LVNLR......YLCLD......IIHRD......TDFGM......GVDYF......
145526827    ......AILEN......IVQLH......YLIMD......IIYRD......TDFGL......CVDWW......
145526312    ......NILSY......IVKLR......CLLMD......IIYRD......TDFGL......AVDWY......
145525629    ......DVMVQ......ICQLE......CFGME......VLYRD......ADFGL......AVDYY......
145527318    ......YLLSN......LVNMH......YLVMD......VIHRD......TDLGI......AVDYF......
145528453    ......QLLQK......IINMV......YLILD......VIHRD......TDFGI......CVDYY......
145524836    ......-----......VEQLE......HFFFK......ITFGD......TDFGY......MSDWY......
145529872    ......MILEH......LVNLA......FFIMQ......IVYRD......TDFGM......TADWW......
145528564    ......TVLEQ......IMGFV......YFLVD......IVYRD......IDLGT......SVDLW......
145528848    ......QFLTE......LINAF......YLVLD......IIHRD......TDLGV......AVDFF......
145530123    ......DIMSI......IVKMQ......VFVLE......IIYRD......ADFGL......QLDLY......
145528590    ......DIMVH......LCKLL......CFAME......VIYRD......ADFGL......QIDHY......
145533214    ......SILSN......LVNIY......FLVLD......IIHRD......TDLGI......AVDYF......
145532821    ......SVLTS......LMKYY......FFLVE......IVYRD......IDMGT......PVDIW......
145534103    ......NILVE......IIKMF......YFVLE......IIYRD......TDFGL......PVDWW......
145531351    ......NIMIE......IIKLV......YFCME......IIFRD......TDFGL......AVDWW......
145532954    ......NILSQ......LVNLI......YLALD......IIHRD......TDFGL......AADYF......
145533364    ......KILEK......VVKLR......YFIVD......VIYRD......SDFGL......TCDFY......
145532252    ......LIMSS......LTRLH......VFVMD......VIYRD......SDFGL......MLDCY......
145533611    ......QILEV......IVQLH......YLVTD......IIYRD......TDFGL......SADWW......
145535087    ......KLLET......IIKLV......VFVLP......YLYRD......TDLGL......MIDWW......
145535882    ......NLLSQ......IANIH......YLVMD......IIHRD......TDFGI......GVDHF......
145535718    ......QTLQT......IMHFV......YFLEE......IIYRD......IDLGT......SVDLW......
145537039    ......KIMSL......MVKLH......YMVMD......IIYRD......TDFGL......VVDFY......
145535866    ......DVLKQ......IVQLK......YLVME......VIYRD......TDFGL......EVDLW......
145537345    ......DLMCN......IVKIQ......IFVLE......IIYRD......ADFGL......QLDLY......
145537792    ......KLLEE......LISLE......FFVMR......VVYRD......TDYGL......GVDWW......
145537530    ......NILAV......LNKLE......IFITE......IIYRD......ADFGL......QLDHY......
145536089    ......NVLGA......VVKLK......YLVME......VIYRD......ADFGL......MVDFW......
145536099    ......NVLKM......ILGLH......YLVLD......IIYRD......ADFGL......PSDIY......
145538584    ......KILEQ......LVSLE......YFVMK......IIYRD......TDYGL......TADWW......
145541165    ......DIMSI......IVKMQ......VFVLE......IIYRD......ADFGL......QLDLY......
145542570    ......KLLSE......LVNMN......YLIMD......IIHRD......TDFGI......AVDFY......
145539628    ......KLLRK......IISVN......YYQMK......IIYRD......ADLGL......SVDVY......
145541688    ......NVLKM......ILKLH......YLVLD......IIYRD......ADFGL......PSDIY......
145540369    ......DILIE......TSKIE......IFGIE......IIYRD......ADFGL......LVDYY......
145542684    ......LVLEH......LVNLH......FFVTQ......IIYRD......TDFGM......SADWW......
145541906    ......KILEI......ITKLH......YFIID......IIYRD......CDFGL......VCDWW......
145542195    ......NILAL......IVNLY......YFVID......IIYRD......TDFGL......SADWY......
145540353    ......ALLCG......LINMQ......YLVMD......ILHRD......TDLGI......AVDYF......
145540575    ......NIMTL......LLHLS......VFVLE......ILYRD......ADFGL......TVDFY......
145540766    ......KLLEE......LISLE......FFVMR......VVYRD......TDYGL......GVDWW......
145540331    ......QVMEQ......LAKLF......VFVLE......VVYRD......SDFGL......MVDIY......
145538802    ......NLLAQ......LINMN......YLVMD......IIHRD......TDLGI......AVDYF......
145539149    ......VLLSQ......LVNMN......YLIMD......IIHRD......TDFGI......AADYY......
145542408    ......NLLTQ......LINMV......YLVMD......VIHRD......TDLGI......AVDYF......
145541672    ......NVLGA......VVKLK......YLVME......VIYRD......TDFGL......MVDFW......
145540890    ......YLLEH......LVNMW......FLIID......IIHRD......TDLGI......VVDYY......
145540505    ......NVLSI......IVKLR......VMILD......IIYRD......TDFGL......AVDWY......
145539812    ......QILRT......IVKLY......YIVTE......IIYRD......TDFGL......ATDIY......
145538989    ......NLLAQ......LINMN......YLVMD......VIHRD......TDLGI......AVDYF......
```

145539219	......IVLEH.....LVSLN.....FFITQ.....IIYRD.....TDFGM.....AADWW.....
145545295	......SIMKY.....VVKLH.....YMLLE.....LIYRD.....IDFGL.....STDIY.....
145547970	......DILKQ.....IVNLF.....YLVMD.....IIYRD.....VDFGL.....ATDVY.....
145547256	......DIMVN.....ITPLH.....AFVLD.....IVYRD.....SDFGL.....LVDCY.....
145544703	......DIMVR.....ITKLH.....AFVLE.....IVYRD.....SDFGL.....LVDCY.....
145547431	......KFLTE.....IVNAY.....YLVLD.....IIHRD.....TDLGV.....PADYF.....
145548036	......MLLSQ.....LINMY.....YLVMD.....IIHRD.....TDLGI.....AVDYY.....
145548052	......YLLEH.....LVNMS.....YLIID.....IIHRD.....TDLGI.....VADYY.....
145546965	......QVLTS.....LMRYY.....FFLVE.....IVYRD.....IDMGT.....PVDIW.....
145546438	......NVLSL.....IVRLR.....CLIMD.....IIFRD.....TDFGL.....AVDWY.....
145544422	......ELLSQ.....LVNMC.....YLIMD.....IIHRD.....TDLGI.....AVDYF.....
145543697	......QVMEQ.....LAKLF.....VFVME.....VVYRD.....SDFGL.....MVDIY.....
145547545	......DIMTV.....IIKLE.....VFVLE.....VVYRD.....ADFGL.....QVDHY.....
145546817	......DVMVQ.....ICQLE.....CFGME.....VLYRD.....ADFGL.....AVDYY.....
145546520	......DIMVQ.....VTPLH.....AFVLD.....IVYRD.....SDFGL.....LVDCY.....
145545784	......IILAT.....LVRLR.....YLVVD.....IIYRD.....TDFGL.....TCDWW.....
145543723	......ALLCG.....LINMQ.....YLVMD.....ILHRD.....TDLGI.....AVDYF.....
145543859	......NVLSM.....IVKLR.....IMMLD.....IIYRD.....TDFGL.....AVDWY.....
145546685	......DIMAF.....TIQML.....VFILE.....IMYRD.....SDFGL.....QVDHY.....
145543739	......DILIE.....TSKIE.....IFGIE.....IIYRD.....ADFGL.....MVDYY.....
145552912	......QLLSN.....MINMQ.....YLVMD.....ILHRD.....TDLGI.....GVDYF.....
145548473	......SILSV.....IVKLH.....FLVMD.....IMYRD.....TDFGL.....SVDWY.....
145551470	......DILIE.....TSKIE.....IFGIE.....MIYRD.....ADFGL.....LVDYY.....
145553187	......DIMCN.....IVKIQ.....VFVLE.....IIYRD.....ADFGL.....QLDLY.....
145551514	......YVMEH.....LAKLF.....VFVME.....IVYRD.....SDFGL.....MVDIY.....
145473773	......WIMSN.....LTRLH.....VFVMD.....VIYRD.....SDFGL.....LLDCY.....
145552936	......DILIQ.....TNQIE.....VFGIE.....IIYRD.....ADFGL.....LVDYY.....
145551488	......TLLIK.....LINMQ.....YLVMD.....VLHRD.....TDLGI.....AVDYF.....
145549281	......YLLEH.....LVNMW.....FLIID.....IIHRD.....TDLGI.....VADYY.....
145553449	......NIMSQ.....FVKLR.....YMVMD.....IMYRD.....TDFGL.....TVDFY.....
145550002	......DIMVK.....ITPLH.....AFVLD.....IIYRD.....SDFGL.....LVDCY.....
145549888	......DIMYN.....IVKMQ.....VFVLE.....IIYRD.....ADFGL.....QLDLY.....
145549464	......KIMTL.....SVKLH.....YLVMD.....IIYRD.....TDFGL.....VVDFY.....
145551294	......NIMTL.....LLHLS.....VFVLE.....ILYRD.....ADFGL.....TVDFY.....
112253673	......NVLQV.....IVELH.....HFVLE.....IIYRD.....TDFGL.....AVDWY.....
125834130	......RVLKN.....LTSLK.....CFVME.....IVYRD.....TDFGL.....AVDWW.....
126290348	......RVLQN.....LTALK.....CFVME.....VVYRD.....TDFGL.....AVDWW.....
126307179	......RVLKN.....LTSLK.....CFVME.....IVYRD.....TDFGL.....AVDWW.....
126329305	......RVLQN.....LTALK.....CFVME.....VVYRD.....TDFGL.....AVDWW.....
145232559	......SVLSQ.....IVPLK.....YLVLA.....VIYRD.....CDFGL.....TVDWW.....
154341547	......IILEE.....IVHLH.....YMILD.....IIYRD.....TDFGL.....AVDWW.....
154340517	......TILTS.....IVRCH.....VLVMD.....VVHRD.....TDFGL.....AVDWW.....
154338946	......NVLTR.....LLKLY.....FFVMD.....VIYRD.....ADFGL.....AVDFW.....
146093678	......ATLQR.....VVRLY.....YFVLE.....VVLRD.....ENGGA.....SSDLW.....
146089211	......NVLTR.....LLKLY.....FFVMD.....VIYRD.....ADFGL.....AVDFW.....
146093638	......IILEE.....IVHLH.....YMILD.....IIYRD.....TDFGL.....AVDWW.....
145255520	......NILVR.....IVGLK.....YLVTD.....IVYRD.....CDFGL.....MVDFW.....
146092107	......AILTS.....IVRCH.....VFVLD.....VVHRD.....TDFGL.....AVDWW.....
134104886	......RVLQN.....LTALK.....CFVME.....VVYRD.....TDFGL.....AVDWW.....
145348563	......DILTA.....IVSLR.....YLILE.....IMHRD.....TDFGL.....TADWW.....
145616654	......NILVR.....IVSLK.....YLVTD.....IVYRD.....CDFGL.....MVDFW.....
145608478	......SVLAQ.....IVPLK.....YFVLA.....VVYRD.....CDFGL.....AVDWW.....
145605591	......EILQV.....IVSLH.....YLCME.....LIYRD.....LDTRS.....AVDWW.....
146185241	......MILEH.....LVNLV.....FFVMQ.....IVYRD.....SDFGM.....TTDWW.....
146186209	......NILQT.....IVKMK.....FFVLE.....IIYRD.....TDFGL.....PVDWW.....
146183986	......KILEQ.....LVNLE.....FFAMK.....IIYRD.....TDFGL.....TADYW.....
146182205	......NILKN.....IVKLH.....YLMMD.....IMYRD.....TDFGL.....MADFW.....
146169354	......KVMQT.....IMRFF.....YFLLE.....IIYRD.....IDMGT.....LVDLW.....
146163394	......DILAN.....IIKLH.....FFALD.....IIYRD.....SDFGL.....PVDWW.....
146161347	......NVLSV.....IVSLK.....YLVMD.....IVFRD.....TDFGL.....SLDWY.....
146415376	......TVLAR.....IVPLK.....YLVLS.....VIYRD.....CDFGL.....SVDWW.....
146417529	......DILVR.....IVGLK.....YLVTD.....IVYRD.....CDFGL.....MVDFW.....
149248728	......NILVR.....IVGLK.....FLVTD.....IVYRD.....CDFGL.....MVDFW.....
149247428	......TVLAR.....IVPLK.....YLVLS.....VIYRD.....CDFGL.....SVDWW.....
147800623	......EILRM.....LPTLY.....CLVME.....VVYRD.....TDFDL.....AVDWW.....
148686640	......RVLQN.....LTALK.....CFVME.....VVYRD.....TDFGL.....AVDWW.....
156095919	......NVLKC.....IVKLY.....YFILE.....IIYRD.....TDFGL.....AVDWW.....
149040829	......RVLKN.....LTSLK.....CFVME.....IVYRD.....TDFGL.....AVDWW.....
149056509	......RVLQN.....LTALK.....CFVME.....VVYRD.....TDFGL.....AVDWW.....
149263735	......RVLQN.....LTALK.....CFVME.....VVYRD.....TDFGL.....AVDWW.....
150864017	......TVLAR.....IVPLK.....YLVLS.....VIYRD.....CDFGL.....SVDWW.....
150864829	......NILVR.....IVGLK.....YLVTD.....IVYRD.....CDFGL.....MVDFW.....

149641667	......RVLKN.....LTSLK.....CFVME.....IVYRD.....TDFGL.....AVDWW......
149722123	......RVLQN.....LTALK.....CFVME.....VVYRD.....TDFGL.....AVDWW......
149737757	......RVLQN.....LTALK.....CFVME.....VVYRD.....TDFGL.....AVDWW......
154271095	......SVLSQ.....IVPLK.....YLVLA.....VIYRD.....CDFGL.....SVDWW......
154284460	......NILVR.....IVGLK.....YLVTD.....IVYRD.....CDFGL.....MVDFW......
154270226	......SVLSQ.....IVPLK.....YLVLA.....IIYRD.....CDFGL.....AVDWW......
154292128	......NILVR.....IVGLK.....YLVTD.....IVYRD.....CDFGL.....MVDFW......
154291241	......SVLSQ.....IVPLK.....YFVLA.....VIYRD.....CDFGL.....TVDWW......
157113137	......DILSF.....VVSMY.....CLVME.....IVHRD.....TDFGL.....PVDWW......
157167717	......NALAL.....CVILY.....YLVME.....IVHRD.....TDFGL.....QVDWW......
109003954	......DILTF.....VVSMF.....CMVME.....IVHRD.....TDFGL.....PVDWW......
109003948	......DILTF.....VVSMF.....CMVME.....IVHRD.....TDFGL.....PVDWW......
109003938	......DILTF.....VVSMF.....CMVME.....IVHRD.....TDFGL.....PVDWW......
109003957	......DILTF.....VVSMF.....CMVME.....IVHRD.....TDFGL.....PVDWW......
109003951	......DILTF.....VVSMF.....CMVME.....IVHRD.....TDFGL.....PVDWW......
109003945	......DILTF.....VVSMF.....CMVME.....IVHRD.....TDFGL.....PVDWW......
109003932	......DILTF.....VVSMF.....CMVME.....IVHRD.....TDFGL.....PVDWW......
109003935	......DILTF.....VVSMF.....CMVME.....IVHRD.....TDFGL.....PVDWW......
109077439	......DILTF.....VVSMY.....CMVME.....IVHRD.....TDFGL.....PVDWW......
109123660	......DILTF.....VVGMF.....CMVME.....IVHRD.....TDFGL.....PVDWW......
109123957	......DILTF.....VVSMF.....CMVME.....IVHRD.....TDFGL.....PVDWW......
109464300	......DILTF.....VVSMY.....CMVME.....IVHRD.....TDFGL.....PVDWW......
109503160	......DILTF.....VVGMF.....CMVME.....IVHRD.....TDFGL.....PVDWW......
109503156	......DILTF.....VVGMF.....CMVME.....IVHRD.....TDFGL.....PVDWW......
109503158	......DILTF.....VVGMF.....CMVME.....IVHRD.....TDFGL.....PVDWW......
109503934	......DILTF.....VVGMF.....CMVME.....IVHRD.....TDFGL.....PVDWW......
109503932	......DILTF.....VVGMF.....CMVME.....IVHRD.....TDFGL.....PVDWW......
110749524	......DIMSF.....VVSMY.....CLVME.....IVHRD.....TDFGL.....PVDWW......
112363074	......DILTF.....VVSMF.....CMVME.....IVHRD.....TDFGL.....PVDWW......
112363078	......DILTF.....VVSMF.....CMVME.....IVHRD.....TDFGL.....PVDWW......
114556123	......DILTF.....VVSMF.....CMVME.....IVHRD.....TDFGL.....PVDWW......
114676060	......DILTF.....VVSMF.....CMVME.....IVHRD.....TDFGL.....PVDWW......
115502256	......DILTF.....VVSMY.....CMVME.....IVHRD.....TDFGL.....PVDWW......
115938031	......DILTF.....VVALY.....CMVME.....IVHRD.....TDFGL.....PVDWW......
57547570	......DILTF.....VVSMY.....CMVME.....IVHRD.....TDFGL.....PVDWW......
116487447	......DILTF.....VVSMF.....CMVME.....IVHRD.....TDFGL.....PVDWW......
118094583	......DILTF.....VVSMF.....CMVME.....IVHRD.....TDFGL.....PVDWW......
118103299	......DILTF.....VVSMF.....CMVME.....IVHRD.....TDFGL.....PVDWW......
119571705	......DILTF.....VVSMY.....CMVME.....IVHRD.....TDFGL.....PVDWW......
119571704	......DILTF.....VVSMY.....CMVME.....IVHRD.....TDFGL.....PVDWW......
119571706	......DILTF.....VVSMY.....CMVME.....IVHRD.....TDFGL.....PVDWW......
119894536	......DILTF.....VVSMF.....CMVME.....IVHRD.....TDFGL.....PVDWW......
119605063	......DILTF.....VVSMF.....CMVME.....IVHRD.....TDFGL.....PVDWW......
119604724	......DILTF.....VVGMF.....CMVME.....IVHRD.....TDFGL.....PVDWW......
119604723	......DILTF.....VVGMF.....CMVME.....IVHRD.....TDFGL.....PVDWW......
119627354	......DILTF.....VVSMF.....CMVME.....IVHRD.....TDFGL.....PVDWW......
119627353	......DILTF.....VVSMF.....CMVME.....IVHRD.....TDFGL.....PVDWW......
123255658	......DILTF.....VVSMF.....CMVME.....IVHRD.....TDFGL.....PVDWW......
145483315	......NVFEI.....VVKAF.....LFLLE.....IIHRD.....ADFGL.....SIDWW......
145485608	......NIFEI.....VVKAY.....CFVQE.....IVHRD.....ADFGL.....STDYW......
145483443	......NIFEI.....VVKAF.....CFVQE.....IVHRD.....ADFGL.....TLDYW......
145500596	......NIFEI.....VVKAY.....CFVQE.....IVHRD.....ADFGL.....SCDYW......
145504807	......NIFEI.....VVKAY.....CFVQE.....IVHRD.....ADFGL.....SLDYW......
145510378	......NIFEI.....VVKAY.....CFVQE.....IVHRD.....ADFGL.....SADYW......
145517496	......NIFEI.....VVKAY.....IFVQE.....IVHRD.....ADFGL.....SSDYW......
145521021	......NIFEI.....VAKAF.....CFVQE.....IVHRD.....ADFGL.....TLDYW......
145518646	......NIFEI.....VVKAY.....CFVQE.....IVHRD.....ADFGL.....SSDYW......
145526701	......NIFEI.....VVKAY.....CFVQE.....IVHRD.....ADFGL.....SCDYW......
145536247	......NIFEI.....VVKAY.....CFAQE.....IVHRD.....ADFGL.....SQDFW......
145536013	......TIFSV.....VAKAY.....FYVLE.....IIHRD.....TDFGL.....SLDQW......
145541806	......NIFEI.....VVKAY.....CFAQE.....IIHRD.....ADFGL.....SQDFW......
145541582	......TIFSV.....VAKAY.....FYVLE.....IIHRD.....TDFGL.....SLDQW......
145545165	......NIFEI.....VVKAY.....CFVQE.....IVHRD.....ADFGL.....SLDYW......
145549337	......NVFEI.....VVKAY.....LFLLE.....IIHRD.....ADFGL.....SIDWW......
145549720	......NIFEI.....VVKAF.....CFVQE.....IVHRD.....ADFGL.....SADYW......
145552986	......NIFEI.....VVKAF.....CFVQE.....IVHRD.....ADFGL.....TLDYW......
125804509	......DILTF.....VVSMF.....CMVME.....IVHRD.....TDFGL.....PVDWW......
125813773	......DILTF.....VVSMF.....CMVME.....IVHRD.....TDFGL.....PVDWW......
125817906	......DILTF.....VVSMY.....CMVME.....IVHRD.....TDFGL.....PVDWW......
125819684	......DILTF.....VVSMF.....CMVME.....IVHRD.....TDFGL.....PVDWW......
125820126	......DILTF.....VVSMF.....CMVME.....IVHRD.....TDFGL.....PVDWW......
125820197	......DILTF.....VVSMF.....CMVME.....IVHRD.....TDFGL.....PVDWW......

125830177	......DILTF.....VVSMF.....CMVME.....IVHRD.....TDFGL.....PVDWW.....
126215744	......DILTF.....VVGMF.....CMVME.....IVHRD.....TDFGL.....PVDWW.....
126317241	......DILTF.....VVSMY.....CMVME.....IVHRD.....TDFGL.....PVDWW.....
126323498	......DILTF.....VVSMF.....CMVME.....IVHRD.....TDFGL.....PVDWW.....
148668446	......DILTF.....VVSMY.....CMVME.....IVHRD.....TDFGL.....PVDWW.....
148679025	......DILTF.....VVGMF.....CMVME.....IVHRD.....TDFGL.....PVDWW.....
148679024	......DILTF.....VVGMF.....CMVME.....IVHRD.....TDFGL.....PVDWW.....
148698662	......DILTF.....VVSMF.....CMVME.....IVHRD.....TDFGL.....PVDWW.....
148696927	......DILTF.....VVGMF.....CMVME.....IVHRD.....TDFGL.....PVDWW.....
148727255	......DILTF.....VVSMY.....CMVME.....IVHRD.....TDFGL.....PVDWW.....
157822407	......DILTF.....VVSMF.....CMVME.....IVHRD.....TDFGL.....PVDWW.....
149036073	......DILTF.....VVGMF.....CMVME.....IVHRD.....TDFGL.....PVDWW.....
149037826	......DILTF.....VVGMF.....CMVME.....IVHRD.....TDFGL.....PVDWW.....
149551314	......DILTF.....VVSMY.....CMVME.....IVHRD.....TDFGL.....PVDWW.....
149628931	......DILTF.....VVSMF.....CMVME.....IVHRD.....TDFGL.....PVDWW.....
149632387	......DILTF.....VVSMF.....CMVME.....IVHRD.....TDFGL.....PVDWW.....
149732856	......DILTF.....VVSMY.....CMVME.....IVHRD.....TDFGL.....PVDWW.....
115454919	......DILIT.....VVRFF.....YLVME.....IVHRD.....TDFGL.....SADWW.....
157107396	......DVLVE.....VVKMY.....YLIME.....FIHRD.....SDFGL.....ACDWW.....
157137575	......DILAE.....VVKLY.....YFVMD.....FIHRD.....TDFGL.....LCDWW.....
157124880	......DVLVE.....VVKMY.....YLIME.....FIHRD.....SDFGL.....ACDWW.....
109070924	......DILVE.....VVKMF.....YLIME.....FIHRD.....SDFGL.....LCDWW.....
109088499	......DALAL.....IVHLY.....YLVME.....IIHRD.....TDFGL.....AVDWW.....
109096055	......DILVE.....VVKMF.....YLIME.....FIHRD.....SDFGL.....LCDWW.....
109121397	......DILAE.....VVKLY.....YFVMD.....FIHRD.....TDFGL.....LCDWW.....
109121399	......DILAE.....VVKLY.....YFVMD.....FIHRD.....TDFGL.....LCDWW.....
109457529	......DILAE.....VVRLY.....YFVMD.....FIHRD.....TDFGL.....LCDWW.....
134053877	......DILVE.....VVKMF.....YLIME.....FIHRD.....SDFGL.....LCDWW.....
157817113	......DALAL.....VVHLY.....YLIME.....IIHRD.....TDFGL.....AVDWW.....
110740013	......NILIT.....LVRFF.....YLVME.....IVHRD.....TDFGL.....AADWW.....
110756258	......DILAE.....VVKLY.....YFVMD.....FIHRD.....TDFGL.....LCDWW.....
115482504	......NLLAE.....IVKLY.....YLIME.....YIHRD.....SDFGL.....ECDWW.....
115401772	......AIMMQ.....VAKLY.....YLVME.....IVHRD.....TDFGL.....MSDWW.....
115400379	......DFLAS.....IVPLI.....YLIID.....GDTKD.....ESFGV.....RCDWW.....
115386624	......DILTA.....LVKLL.....YLAME.....YIHRD.....TDFGL.....TVDYW.....
115391099	......DILAD.....LVKLH.....YLLME.....FLHRD.....TDFGL.....LCDWW.....
114607162	......DILVE.....VVKMF.....YLIME.....FIHRD.....SDFGL.....LCDWW.....
114607168	......DILVE.....VVKMF.....YLIME.....FIHRD.....SDFGL.....LCDWW.....
114607164	......DILVE.....VVKMF.....YLIME.....FIHRD.....SDFGL.....LCDWW.....
114607166	......DILVE.....VVKMF.....YLIME.....FIHRD.....SDFGL.....LCDWW.....
114607170	......DILVE.....VVKMF.....YLIME.....FIHRD.....SDFGL.....LCDWW.....
114609742	......DILAE.....VVRLY.....YFVMD.....FIHRD.....TDFGL.....LCDWW.....
114609744	......DILAE.....VVRLY.....YFVMD.....FIHRD.....TDFGL.....LCDWW.....
114629807	......DALAL.....IVHLY.....YLVME.....IIHRD.....TDFGL.....AVDWW.....
114645553	......DILVE.....VVKMF.....YLIME.....FIHRD.....SDFGL.....LCDWW.....
114648937	......DILAE.....VVKLY.....YFVMD.....FIHRD.....TDFGL.....LCDWW.....
114648935	......DILAE.....VVKLY.....YFVMD.....FIHRD.....TDFGL.....LCDWW.....
115742081	......DILAE.....VVKLY.....YFVMD.....FIHRD.....TDFGL.....LCDWW.....
115901678	......DILVE.....VVKMY.....YLIME.....FIHRD.....SDFGL.....VCDWW.....
116059539	......NLLAE.....VVKLY.....YLVME.....FIHRD.....SDFGL.....ECDWW.....
116059864	......DALIK.....TIKLY.....YLVTE.....ITHRD.....ADFGL.....GVDWW.....
116061055	......EIMKR.....LTRAV.....YLLMD.....YVYRD.....TDFGF.....LADYW.....
169860855	......MILMQ.....VAKLY.....YLVME.....IVHRD.....TDFGL.....AVDWW.....
169861109	......DVLAE.....VVSLF.....YLVME.....FIHRD.....SDFGL.....ECDWW.....
169847285	......DILKS.....IVRLH.....YLILE.....FIHRD.....SDFGL.....TCDWW.....
169849209	......DVLAE.....VVQLF.....YLIME.....YIHRD.....SDFGL.....ECDWW.....
118085059	......DILAE.....VVKLY.....YFVMD.....FIHRD.....TDFGL.....LCDWW.....
118085660	......DALAL.....IVHLY.....YLVME.....IIHRD.....TDFGL.....AVDWW.....
118088357	......DILAE.....VVRLY.....YFVMD.....FIHRD.....TDFGL.....LCDWW.....
118102361	......DILVE.....VVKMF.....YLIME.....FIHRD.....SDFGL.....LCDWW.....
121699695	......DFLAS.....IVPLI.....YLIMD.....WIHRD.....SDFGL.....RCDWW.....
121703960	......DILTA.....LVKLL.....YLAME.....YIHRD.....TDFGL.....TVDYW.....
121708161	......DILAD.....LVKLH.....YLLME.....FLHRD.....TDFGL.....LCDWW.....
121716062	......AIMMQ.....VAKLY.....YLVME.....IVHRD.....TDFGL.....MGDWW.....
119481871	......AIMMQ.....VAKLY.....YLVME.....IVHRD.....TDFGL.....MSDWW.....
119467340	......DILTA.....LVKLL.....YLAME.....YIHRD.....TDFGL.....TVDYW.....
119469765	......DFLAS.....IVPLI.....YLVMD.....WIHRD.....SDFGL.....RCDWW.....
119500172	......DILAD.....LVKLH.....YLLME.....FLHRD.....TDFGL.....LCDWW.....
155371915	......DILVE.....VVKMF.....YLIME.....FIHRD.....SDFGL.....LCDWW.....
119606473	......DALAL.....IVHLY.....YLVME.....IIHRD.....TDFGL.....AVDWW.....
119904736	......DILAE.....VVKLY.....YFVMD.....FIHRD.....TDFGL.....LCDWW.....
166157492	......DALAL.....IVHLY.....YLVME.....IIHRD.....TDFGL.....AVDWW.....
119901628	......DILAE.....VVRLY.....YFVMD.....FIHRD.....TDFGL.....LCDWW.....
123328810	......DILLQ.....IVKFY.....YLVME.....IIHRD.....TDFGL.....TADYW.....
123370064	......DILLE.....VVTFY.....YLVTE.....IIHRD.....TDFGL.....AVDFW.....
123374553	......DILAH.....FTYLN.....YLVLE.....FLHRD.....TDFGL.....LSDFW.....

```
123402404    ......DFMVQ......AVQLY......YFVME......FIHRD......TDFGL......SVDYW......
123423488    ......DILLK......IVEIY......YLVMD......ILHRD......TDFGL......CVDYW......
123445948    ......DILKN......IVTFY......YLVTE......IIHRD......TDFGL......GVDYW......
123439667    ......DILHD......VVSFY......YLFME......IIHRD......TDFGL......TTDYW......
123448633    ......DILLQ......VTNFY......YLVME......IIHRD......ADFGL......TTDYW......
123976131    ......MTLSQ......VVSMY......YIVME......IIHRD......IDFGL......SVDYW......
123976849    ......NIMLH......TIRFF......YMVME......VIHRD......VDFGL......QADYW......
123473711    ......EVMCR......TISLF......FFVMS......VVHCD......TDFGL......KVDWW......
123471530    ......DILAI......IVQLQ......YLILE......FIHSD......TDFGI......KNDWW......
123478484    ......NILSV......IVNLH......YLLLD......YVHSD......TDFGI......ESDWW......
123510101    ......NILVK......VVSLY......YIVME......IVHRD......ADFGL......PADYW......
123500624    ......DILLQ......IVDFY......YLVME......IVHRD......TDFGL......TADYW......
123487575    ......DLLSR......SVQLI......YFVME......YIHCD......SDYGL......EVDLW......
123480500    ......DILLH......IINFY......YIVME......IIHRD......TDFGL......PVDYW......
154421156    ......DFLVQ......SVQLY......YLVME......FIHRD......TDFGL......SVDYW......
154420625    ......DFYTQ......SAKLY......YLIME......FIHRD......IDFGL......NADFW......
154416213    ......DFLFQ......IVKIY......YIVMD......FVHRD......IDFGL......SSDYW......
123232013    ......DALAL......VVHLY......YLIME......IIHRD......TDFGL......AVDWW......
145475387    ......KVLEE......IVEMK......YLVME......FIHRD......SDFGL......TVDWW......
145478689    ......DILSQ......VVELK......YLVME......YIHRD......SDFGL......TVDWW......
145489496    ......DLLAA......IVQLK......YLVME......YIHRD......SDFGL......TADWW......
145497641    ......KVLEE......IVEMK......YLVME......FIHRD......SDFGL......SVDWW......
145510472    ......DILVT......IVELK......YLVME......YIHRD......SDFGL......LVDWW......
145520068    ......DLLAA......IVQLK......YLVME......YIHRD......SDFGL......TADWW......
145527474    ......KVLEE......IVEMK......YLVME......FIHRD......SDFGL......SVDWW......
145530221    ......DLLAA......IVQLK......YLVME......YIHRD......SDFGL......TADWW......
145530870    ......DILSQ......VVELK......YLVME......YIHRD......SDFGL......TVDWW......
145533140    ......KVLEE......IVEMK......YLVME......FIHRD......SDFGL......TVDWW......
145537375    ......DLLVQ......IVELK......YLVME......YIHRD......SDFGL......LVDWW......
145537502    ......NVFEI......VVKAF......LFLLE......IIHRD......SEIAL......SIDWW......
145543308    ......DILVQ......VVQLK......YLVME......YIHRD......SDFGL......LVDWW......
145549818    ......DILVS......IVELK......YLVME......YIHRD......SDFGL......LVDWW......
145553153    ......DLLVQ......IVELK......YLVME......YIHRD......SDFGL......LVDWW......
125991876    ......DILVE......VVKMF......YLIME......FIHRD......SDFGL......LCDWW......
125524717    ......NLLAE......IVKLY......YLIME......YIHRD......SDFGL......ECDWW......
125537456    ......DILIS......VVRFF......YLVME......VIHRD......TDFGL......TADWW......
125545475    ......DILIT......VVRFF......YLVME......IVHRD......TDFGL......SADWW......
125552955    ......NLLAE......IVKLY......YLIME......YIHRD......SDFGL......ECDW-......
125569326    ......NLLAE......IVKLY......YLIME......YIHRD......SDFGL......ECDWW......
125580114    ......DILIS......VVRFF......YLVME......VIHRD......NDFGL......TADWW......
125587679    ......DILIT......VVRFF......YLVME......IVHRD......TDFGL......SADWW......
125594848    ......NLLAE......IVKLY......YLIME......YIHRD......SDFGL......ECDW-......
125825790    ......DILAE......VVRLY......YFVMD......FIHRD......TDFGL......LCDWW......
126133354    ......DVLAG......VVSLY......YLIME......FIHRD......SDFGL......MVDWW......
126309927    ......DILVE......VVKMF......YLIME......FIHRD......SDFGL......LCDWW......
126310687    ......DILAE......VVRLY......YFVMD......FIHRD......TDFGL......LCDWW......
126327371    ......DILAE......VVKLY......YFVMD......FIHRD......TDFGL......LCDWW......
126340336    ......DILVE......VVKMF......YLIME......FIHRD......SDFGL......LCDWW......
126341449    ......DALAL......IVHLY......YLVME......IIHRD......TDFGL......AVDWW......
145229667    ......DILAD......LVKLH......YLLMD......FLHRD......TDFGL......LCDWW......
154331217    ......DVLAE......VVHLY......YMVME......FVHRD......SDFGL......GCDWW......
146071803    ......DVLAE......VVHLY......YMVME......FVHRD......SDFGL......GCDWW......
145244060    ......DFLAS......VVPLI......YLIMD......WIHRD......SDFGL......RCDWW......
145247144    ......DILTA......LVKLL......YLAME......YIHRD......TDFGL......TVDYW......
145250677    ......AIMMQ......VAKLY......YLVME......IVHRD......TDFGL......MSDWW......
145350653    ......NLLAE......VVTLY......YLVME......FIHRD......SDFGL......ECDWW......
145353560    ......ELMKR......LTRAL......YLLMD......YVYRD......TDFGF......LADYW......
145603101    ......DFLVA......IVPLI......YLVME......CIHRD......SDFGL......RCDWW......
145333046    ......NLLAE......IVKLY......YLIME......YIHRD......SDFGL......ECDWW......
145336472    ......NILIT......LVRFF......YLVME......IVHRD......TDFGL......AADWW......
146183557    ......KIMKY......IPELY......YIVMA......FSHRD......IDF--......NGDLW......
146161265    ......DVLAT......IVELY......YLVME......YIHRD......SDFGL......IVDWW......
146423210    ......DVLAG......VVSLY......YLIME......FIHRD......SDFGL......ECDWW......
146422159    ......AVMMQ......VAQLY......YLVME......IIHRD......TDFGL......ASDWW......
146414862    ......DILTN......LVKLL......FLAME......FTHRD......TDFGL......TIDYW......
149247488    ......DVLAN......VVSLF......YLIME......FIHRD......SDFGL......ECDWW......
149245092    ......DILTN......LVKLL......FLAME......FTHRD......TDFGL......TIDYW......
149235526    ......AVMMQ......VAQLY......YLVME......IIHRD......TDFGL......ASDWW......
147815455    ......NLLAE......IVKLY......YLVME......YIHRD......SDFGL......ECDWW......
148690059    ......DILVE......VVKIF......YLIME......FIHRD......SDVGL......LCDWW......
148671596    ......DILAE......VVRLY......YFVMD......FIHRD......TDFGL......LCDWW......
148690654    ......DILVE......VVKMF......YLIME......FIHRD......SDFGL......LCDWW......
148704219    ......DILAE......VVKLY......YFVMD......FIHRD......TDFGL......LCDWW......
```

```
148704218   ......DILAE......VVKLY......YFVMD......FIHRD......TDFGL......LCDWW......
148707942   ......DILVE......VVKMF......YLIME......FIHWD......SDFGL......LCDWW......
149043496   ......DILVE......VVRMF......YLIME......FIHRD......SDFGL......LCDWW......
149039526   ......DILAE......VVRLY......YFVMD......FIHRD......TDFGL......LCDWW......
149234236   ......DILVE......VVKMF......YLIME......FIHWD......SDFGL......LCDWW......
150864913   ......AVMMQ......VAQLY......YLVME......IIHRD......TDFGL......SSDWW......
150865844   ......DILTN......LVKLL......FLAME......FTHRD......TDFGL......TIDYW......
149409972   ......DILAE......VVRLY......YFVMD......FIHRD......TDFGL......LCDWW......
149634728   ......DALAL......IVHLY......YLVME......IIHRD......TDFGL......EVDWW......
149732471   ......DILVE......VVKMF......YLIME......FIHRD......SDFGL......LCDWW......
149743489   ......DALAL......IVHLY......YLVME......IIHRD......TDFGL......AVDWW......
149744111   ......DILAE......VVRLY......YFVMD......FIHRD......TDFGL......LCDWW......
154284834   ......AIMMQ......VAKLY......YLVME......IVHRD......TDFGL......MSDWW......
154281673   ......DFLVA......IVPLI......YLVMD......----D......SDFGL......RCDWW......
154287318   ......DILAD......LVKLH......YLLME......FLHRD......TDFGL......LCDWW......
154318155   ......DILAE......VVKLF......YMLME......FIHRD......TDFGL......DCDWW......
154300102   ......DILTN......LVRLL......YLAME......YIHRD......TDFGL......TVDYW......
157115672   ......EALNR......FLNLF......YFVMT......IIHRD......ADFGS......ASDIW......
157113413   ......EALNR......FLKLF......FFVVT......IIHRD......ADFGS......ASDLW......
110645413   ......DVMSR......FVKLY......YFGLS......IIHRD......TDFGT......SSDLW......
110743925   ......IVLDQ......IVKLF......YMALE......LIHRD......ADFGS......GNDLW......
110749130   ......EVLNM......FVRLF......YFVLS......IIHRD......TDFGS......ASDLW......
81538200    ......IVLDQ......VIRLF......YMALE......LIHRD......ADFGS......GNDLW......
115391607   ......DTLNR......IVRLY......YFVLD......VIHRD......TDFGT......ASDLW......
116268081   ......DILSC......FVKLF......YFALS......IIHRD......TDFGT......SSDLW......
115617850   ......EVLSR......FVRLY......YFGLS......IIHRD......TDFGT......SSDLW......
169851485   ......DALIA......IVRLH......YFVLD......VAHRD......ADFGS......RGDLW......
90074974    ......DVMSR......FVKLY......YFGLS......IIHRD......TDFGT......SSDLW......
121705994   ......DTLNR......IVRLY......YFVLD......VIHRD......TDFGT......ASDLW......
119491148   ......DTLNR......IVRLY......YFVLD......VIHRD......TDFGT......ASDLW......
123449026   ......DALTK......VVKLY......YFVLD......IIHRD......TDFGT......GSDMW......
123472560   ......DAMTT......IIRLY......YYVVE......IIHRD......TDFGT......ATDLW......
154421080   ......DAMTK......IVKLY......FYVVE......IMHRD......TDFGT......SSDLW......
154416136   ......DAMNA......IIRLY......YYVVE......IIHRD......TDFGT......ATDLW......
145498618   ......EVLSR......LINMI......YLVLE......IAHRD......IDFGT......QADLW......
145516458   ......EMLLY......VVQLY......YFVME......IAHRD......IDFGT......AVDLW......
145514694   ......EMLQY......IIKLH......YFLVE......IVHRD......IDFGT......GTDLW......
145515281   ......EVLSN......IIKLF......YYSLE......LVHRD......IDFGT......QADLW......
145524755   ......-CFYT......LFNYI......YILLW......IAHRD......IDFGT......AVDLW......
145542215   ......EVLTQ......LINLI......YLVLE......IAHRD......IDFGT......QSDLW......
145538902   ......EVLTQ......LINLI......YLVLE......IAHRD......IDFGT......QSDLW......
145546751   ......EVLAY......IIKLF......YYSLE......LVHRD......IDFGT......QADLW......
145545432   ......EVLSR......LINMI......YLVLE......IAHRD......IDFGT......QADLW......
145551223   ......NALTS......VIKCY......YLSLE......VVHRD......IDFGT......QSDLW......
125528547   ......IVLDQ......VIRLF......YMALE......LIHRD......ADFGS......GNDLW......
125572812   ......IVLDQ......VIRLF......YMALE......LIHRD......ADFGS......GNDLW......
126275913   ......HALNR......IISLY......YFVLD......VIHRD......TDFGT......PGDIW......
126314344   ......EALAR......FVKLY......YFGLS......IIHRD......TDFGS......SSDLW......
145233247   ......DTLNR......IVRLY......YFVLD......VIHRD......TDFGT......ASDLW......
146167965   ......EVLTH......IIQLL......YFVLE......IIHRD......IDFGT......SADLW......
146418507   ......TTLHR......IVQLY......FFVLD......VIHRD......TDFGA......EADVW......
146418102   ......HALNR......IISLY......YFVLD......VIHRD......TDFGT......PGDIW......
149239618   ......HALNR......VISLY......YFVLD......VIHRD......TDFGT......PGDIW......
149235911   ......LTLHR......IVQLY......FFVLD......IIHRD......TDFGA......ESDVW......
149051988   ......DVMSR......FVKLY......YFGLS......IIHRD......TDFGT......SSDLW......
150865712   ......ITLHR......IVQLY......FFVLD......VIHRD......TDFGA......EADVW......
149388921   ......HALNR......IISLY......YFVLD......VIHRD......TDFGT......PGDIW......
149426614   ......DVMSR......FVKLY......YFGLS......IIHRD......TDFGT......SSDLW......
154276386   ......-----......VVRLY......YFVLD......VIHRD......TDFGT......ASDLW......
154295500   ......DTLNR......IIRLF......YYVLE......VIHRD......TDFGT......ASDLW......
157130711   ......NILRE......VVNMR......YMLFE......IVYRD......TDFGF......AVDWW......
157125826   ......RILQS......LVNME......YLVMP......LIYRD......TDFGF......SVDWW......
109008719   ......RILQA......LVRLE......YMVME......LIYRD......TDFGF......AVDWW......
109008713   ......RILQA......LVRLE......YMVME......LIYRD......TDFGF......AVDWW......
109008710   ......RILQA......LVRLE......YMVME......LIYRD......TDFGF......AVDWW......
109008704   ......-----......-----......YMVME......LIYRD......TDFGF......AVDWW......
109008701   ......RILQA......LVRLE......YMVME......LIYRD......TDFGF......AVDWW......
109008692   ......RILQA......LVRLE......YMVME......LIYRD......TDFGF......AVDWW......
109008716   ......-----......-----......YMVME......LIYRD......TDFGF......AVDWW......
109008722   ......RILQA......LVRLE......YMVME......LIYRD......TDFGF......AVDWW......
109008698   ......RILQA......LVRLE......YMVME......LIYRD......TDFGF......AVDWW......
109008707   ......RILQA......LVRLE......YMVME......LIYRD......TDFGF......AVDWW......
```

109123703	......RILQA.....LVKLE.....YMVME.....LIYRD.....TDFGF.....AVDWW.....
109129834	......SVLKE.....LIRLF.....YMLME.....IVYRD.....TDFGF.....AVDWW.....
109157913	......RILQA.....LVKLE.....YMVME.....LIYRD.....ADFGF.....AVDWW.....
109157919	......RILQA.....LVKLE.....YMVME.....LIYRD.....ADFGF.....AVDWW.....
109157911	......RILQA.....LVKLE.....YMVME.....LIYRD.....ADFGF.....AVDWW.....
110749905	......RILEC.....IVFME.....YMVLP.....LVYRD.....TDFGF.....SVDWW.....
110760107	......TVLKE.....IVNML.....YMLLE.....IVYRD.....TDFGF.....AVDWW.....
110747125	......RMLGE.....LITLW.....YMVMD.....IIYRD.....TDFGF.....SVDWW.....
115383950	......RMLNR.....LITLW.....YMVMD.....IIYRD.....TDFGF.....SVDWW.....
114557436	......RILQA.....LVRLE.....YMVME.....LIYRD.....TDFGF.....AVDWW.....
114557404	......RILQA.....LVRLE.....YMVME.....LIYRD.....TDFGF.....AVDWW.....
114557396	......-----.....-----.....YMVME.....LIYRD.....TDFGF.....AVDWW.....
114557402	......RILQA.....LVRLE.....YMVME.....LIYRD.....TDFGF.....AVDWW.....
114557422	......RILQA.....LVRLE.....YMVME.....LIYRD.....TDFGF.....AVDWW.....
114557412	......RILQA.....LVRLE.....YMVME.....LIYRD.....TDFGF.....AVDWW.....
114557432	......RILQA.....LVRLE.....YMVME.....LIYRD.....TDFGF.....AVDWW.....
114557428	......RILQA.....LVRLE.....YMVME.....LIYRD.....TDFGF.....AVDWW.....
114557400	......RILQA.....LVRLE.....YMVME.....LIYRD.....TDFGF.....AVDWW.....
114557406	......RILQA.....LVRLE.....YMVME.....LIYRD.....TDFGF.....AVDWW.....
114557410	......RILQA.....LVRLE.....YMVME.....LIYRD.....TDFGF.....AVDWW.....
114557434	......RILQA.....LVRLE.....YMVME.....LIYRD.....TDFGF.....AVDWW.....
114557426	......RILQA.....LVRLE.....YMVME.....LIYRD.....TDFGF.....AVDWW.....
114557392	......RILQA.....LVRLE.....YMVME.....LIYRD.....TDFGF.....AVDWW.....
114557398	......RILQA.....LVRLE.....YMVME.....LIYRD.....TDFGF.....AVDWW.....
114625245	......RILQA.....LVKLQ.....YLVME.....LIHRD.....TDFGF.....AVDWW.....
114675715	......RILQA.....LVKLE.....YMVME.....LIYRD.....TDFGF.....AVDWW.....
114675719	......RILQA.....LVKLE.....YMVME.....LIYRD.....TDFGF.....AVDWW.....
114691904	......SVLKE.....LIRLF.....YMLME.....IVYRD.....TDFGF.....AVDWW.....
115666450	......KILQA.....IVSLA.....YMVLD.....LIYRD.....TDFGF.....AVDWW.....
115666452	......KILQA.....IVSLA.....YMVLD.....LIYRD.....TDFGF.....AVDWW.....
116057215	......RALEE.....VCNYY.....YLVVE.....YVYRD.....GDFGF.....AADIW.....
116061733	......ETMAQ.....LVNLH.....FMLMD.....IIYRD.....TDFSF.....AVDWW.....
118793092	......NILQE.....IVNMR.....YMLFE.....IVYRD.....TDFGF.....AVDWW.....
169853607	......YILSR.....IVDLF.....YMLMS.....IIYRD.....TDFGF.....AADWW.....
169846638	......KMLES.....IINLW.....-----.....IVYRD.....ADFGF.....SVDWY.....
117616646	......RILQA.....LVKLE.....YMVME.....LIYRD.....TDFGF.....AVDWW.....
118084212	......SVLKE.....LIRLF.....YMLME.....IVYRD.....TDFGF.....AVDWW.....
121713166	......KTLAA.....VTTLL.....YMLLE.....VVYRD.....VDFGF.....AVDWW.....
121715784	......RMLNR.....LITLW.....YMVMD.....IIYRD.....TDFGF.....SVDWW.....
119481301	......RMLNR.....LITLW.....YMVMD.....IIYRD.....TDFGF.....SVDWW.....
119479599	......KALAA.....ITTLI.....YMLLD.....VVYRD.....VDFGF.....AVDWW.....
119582876	......RILQA.....LVKLQ.....YLVME.....LIHRD.....TDFGF.....AVDWW.....
119619132	......SVLKE.....LIRLF.....YMLME.....IVYRD.....TDFGF.....AVDWW.....
119920680	......SVLKE.....LVKLF.....YMLME.....IVYRD.....TDFGF.....AVDWW.....
145476401	......TVLEQ.....MMRFY.....YFLIE.....IVYRD.....IDMGT.....SVDLW.....
145480951	......NILNE.....LVQLK.....YMLFE.....IVYRD.....ADFGF.....SIDWW.....
145498638	......QILES.....IIKFY.....FLLLE.....IIHRD.....IDVGI.....LVDLW.....
145503938	......NILNE.....LVQLK.....YMLFE.....IVYRD.....ADFGF.....SIDWW.....
145515996	......DILTF.....IVKMK.....YMLFE.....IVYRD.....TDFGF.....SVDQW.....
145519549	......QVLEI.....IISFM.....YFLLE.....IIYRD.....IDLGT.....LVDLW.....
145524653	......LLLEI.....IISFM.....YFLLE.....IIYRD.....IDLGT.....LVDLW.....
145538305	......QVLST.....LMQFY.....YFLVE.....LLIWD.....IDMGT.....TVDLW.....
145539179	......NILNE.....LVQLK.....YMLFE.....IVYRD.....ADFGF.....SIDWW.....
145540972	......QVLEL.....LVCFR.....YLLTE.....IIYRD.....IDMGT.....STDLW.....
145542620	......NILNE.....LVQLK.....YMLFE.....IVYRD.....ADFGF.....SIDWW.....
145543089	......QVLST.....LMQFY.....YFLVE.....IIYRD.....IDMGT.....SVDLW.....
145544150	......QVLQT.....LMQFY.....YFLIE.....IIYRD.....IDMGT.....TVDLW.....
125803970	......EVLTE.....LVRLF.....YMLME.....IVYRD.....TDFGF.....AVDWW.....
125805184	......RILQA.....LVRLE.....YMVME.....LIYRD.....TDFGF.....AVDWW.....
125744616	......RMLAD.....LITLW.....YMVMD.....IIYRD.....TDFGF.....SVDWW.....
126138238	......RMLKL.....LIRMW.....FMVMD.....IIYRD.....TDFGF.....SVDWW.....
126644805	......AILSR.....LVRMF.....YIMME.....IIYRD.....TDFGF.....PVDWW.....
126306123	......RILQA.....LVKLE.....YMVME.....LVYRD.....TDFGF.....AVDWW.....
126323328	......RILQS.....LVKLE.....YMVME.....LIYRD.....TDFGF.....AVDWW.....
126337073	......SVLKE.....LIRLY.....YMLME.....IVYRD.....TDFGF.....AVDWW.....
126343882	......RILQA.....LVRLE.....YMVME.....LIYRD.....TDFGF.....AVDWW.....
145232221	......RMLNR.....LITLW.....YMVMD.....IIYRD.....TDFGF.....SVDWW.....
154345119	......GILME.....IVNMM.....YFVLE.....VIYRD.....TDFGF.....AVDWW.....
154335603	......SLLQE.....IVNML.....YILLE.....IVYRD.....TDFGF.....AVDWW.....
146101797	......GILME.....IVNMM.....YFVLE.....VIYRD.....TDFGF.....AVDWW.....

```
146101775    ......GILME.....IVNMM.....YFVLE.....VIYRD.....TDFGF.....AVDWW......
146084101    ......SLLQE.....IVSML.....YILLE.....IVYRD.....TDFGF.....AVDWW......
145237884    ......KTLAD.....ITTLI.....YMLLD.....VVYRD.....VDFGF.....AVDWW......
134104894    ......RILQA.....LVKLE.....YMVME.....LIYRD.....TDFGF.....AVDWW......
134104896    ......RILQA.....LTKLE.....YMVME.....LIYRD.....TDFGF.....AVDWW......
145342065    ......QTMAQ.....LVNLR.....YMLMD.....ITYRD.....TDFSF.....AVDWW......
145346024    ......RALEE.....VCNYY.....YLVVE.....YVYRD.....GDFGF.....AADIW......
145353408    ......EVTSE.....LMCQH.....YMLFD.....IIYRD.....GDFGY.....AVDWW......
146182902    ......KILQS.....IVQMK.....YMLFE.....IVYRD.....TDFGF.....SVDWW......
146181550    ......KVLEV.....MMKFI.....YFLLE.....IIYRD.....IDMGT.....SVDLW......
146163404    ......TILSN.....IVKMD.....YLVLE.....IIYRD.....TDFGF.....AVDWW......
146386490    ......RILQA.....LVKLE.....YMVME.....LIYRD.....TDFGF.....AVDWW......
146422256    ......RMLKL.....LIRMW.....FMVMD.....IIYRD.....TDFGF.....SVDWW......
146420301    ......RMLKL.....IIRMW.....FMIMD.....IIYRD.....TDFGF.....SVDWW......
149247234    ......RMLKL.....IIRMW.....FMIMD.....IIYRD.....TDFGF.....SVDWW......
149240045    ......KMLKL.....LIRMW.....FMVMD.....IIYRD.....TDFGF.....SVDWW......
153792580    ......RILQA.....LVSLK.....YMVLE.....LIYRD.....TDFGF.....AVDWW......
156081692    ......KILNY.....CVNLY.....YLVLE.....IVYRD.....TDFGF.....AADWW......
156101830    ......EITAE.....IIRLV.....YFLTE.....IVYRD.....IDFGC.....TVDIW......
149026195    ......RILQA.....LVRLE.....YMVME.....LIYRD.....TDFGF.....AVDWW......
149243958    ......RILQA.....LVKLE.....YMVME.....LIYRD.....TDFGF.....AVDWW......
149243775    ......RILQA.....LTKLE.....YMVME.....LIYRD.....TDFGF.....AVDWW......
150863908    ......RMLKL.....IIRMW.....FMIMD.....IIYRD.....TDFGF.....SVDWW......
149525592    ......RILQA.....LVCLE.....YMVME.....LIYRD.....TDFGF.....AVDWW......
149638302    ......SVLKE.....LIRLY.....YMLME.....IVYRD.....TDFGF.....AVDWW......
149709421    ......RILQA.....LVRLE.....YMVME.....LIYRD.....TDFGF.....AVDWW......
149758977    ......SVLKE.....LVKLF.....YMLME.....IVYRD.....TDFGF.....AVDWW......
154285746    ......ETLNA.....ITTLV.....YMLLE.....IAYRD.....VDFGF.....AVDWW......
154281957    ......RMLQR.....LITLW.....YMVMD.....IIYRD.....TDFGF.....SVDWW......
151556165    ......RILQA.....LVRLE.....YMVME.....LIYRD.....TDFGF.....AVDWW......
157132858    ......AILQN.....VVNLE.....FVVME.....IVHCD.....CDFGF.....SLDMW......
109083221    ......AILQN.....VVNLE.....FVVME.....IVHCD.....CDFGF.....SLDMW......
109102643    ......AILQN.....IVNLE.....FVVME.....IVHCD.....CDFGF.....SLDMW......
109477836    ......AILQN.....IVNLE.....FVVME.....IVHCD.....CDFGF.....SLDMW......
109478194    ......AILQN.....VVNLE.....FVVME.....IVHCD.....CDFGF.....SLDMW......
110764026    ......AILQN.....VVNLE.....FVVME.....IVHCD.....CDFGF.....SLDMW......
114576962    ......AILQN.....IVNLE.....FVVME.....IVHCD.....CDFGF.....SLDMW......
114652488    ......AILQN.....VVNLE.....FVVME.....IVHCD.....CDFGF.....SLDMW......
114652490    ......AILQN.....VVNLE.....FVVME.....IVHCD.....CDFGF.....SLDMW......
114652494    ......AILQN.....VVNLE.....FVVME.....IVHCD.....CDFGF.....SLDMW......
114652492    ......AILQN.....VVNLE.....FVVME.....IVHCD.....CDFGF.....SLDMW......
114677956    ......AILQS.....IVNLE.....FVVME.....IVHCD.....CDFGF.....SLDMW......
115529463    ......AILQN.....VVNLE.....FVVME.....IVHCD.....CDFGF.....SLDMW......
115728593    ......AILHK.....VVNLE.....FVVME.....IVHCD.....CDFGF.....SLDMW......
153945802    ......AILQN.....VVNLE.....FVVME.....IVHCD.....CDFGF.....SLDMW......
117938781    ......AILQS.....IVNLE.....FVVME.....IVHCD.....CDFGF.....SLDMW......
118088112    ......AILQN.....IVNLE.....FVVME.....IVHCD.....CDFGF.....SLDMW......
119903340    ......AILQN.....IVNLE.....FVVME.....IVHCD.....CDFGF.....SLDMW......
119910752    ......AILQS.....IVNLE.....FVVME.....IVHCD.....CDFGF.....SLDMW......
119913888    ......AILQN.....VVNLE.....FVVME.....IVHCD.....CDFGF.....SLDMW......
125821776    ......AILQR.....VIHLE.....FVVME.....IAHCD.....CDFGF.....SLDTW......
125842293    ......AILQN.....IVNLE.....FVVME.....IVHCD.....CDFGF.....SLDMW......
125842606    ......AILQN.....IVNLE.....FVVME.....IVHCD.....CDFGF.....SLDMW......
126282022    ......AILQN.....VVNLE.....FVVME.....VVHCD.....CDFGF.....SLDMW......
126282019    ......AILQN.....VVNLE.....FVVME.....VVHCD.....CDFGF.....SLDMW......
126304548    ......AILQN.....IVNLE.....FVVME.....IVHCD.....CDFGF.....SLDMW......
148710130    ......AILQS.....IVNLE.....FVVME.....IVHCD.....CDFGF.....SLDMW......
148710129    ......AILQS.....IVNLE.....FVVME.....IVHCD.....CDFGF.....SLDMW......
148706531    ......AILQN.....IVNLE.....FVVME.....IVHCD.....CDFGF.....SLDMW......
148706530    ......AILQN.....IVNLE.....FVVME.....IVHCD.....CDFGF.....SLDMW......
148704845    ......AILQN.....VVNLE.....FVVME.....IVHCD.....CDFGF.....SLDMW......
149050625    ......AILQN.....IVNLE.....FVVME.....IVHCD.....CDFGF.....SLDMW......
149051197    ......AILQN.....VVNLE.....FVVME.....IVHCD.....CDFGF.....SLDMW......
149410439    ......AILQN.....VVNLE.....FVVME.....IVHCD.....CDFGF.....SLDMW......
149692893    ......AILQN.....VVNLE.....FVVME.....IVHCD.....CDFGF.....SLDMW......
149727640    ......AILQN.....IVNLE.....FVVME.....IVHCD.....CDFGF.....SLDMW......
157135003    ......DIMLS.....IVRLY.....YFLME.....MIYRD.....VDFGF.....AVDYW......
157117039    ......EIMSE.....IVKLF.....YMLME.....IIYRD.....VDFGF.....SADYW......
157111148    ......KIMLS.....ITRLY.....YMLLE.....IVYRD.....VDFGF.....SVDYW......
```

```
109074352    ......RILEE.....IVKLY.....YMLLE.....IIYRD.....VDFGF.....SVDFW.....
110225911    ......EIMSE.....IVRLY.....YMLME.....IIYRD.....VDFGF.....SADYW.....
110755169    ......HIMQA.....ICKLY.....YFLME.....IVYRD.....VDFGF.....AVDYW.....
114594014    ......RILEE.....IVKLY.....YMLLE.....IIYRD.....VDFGF.....SVDFW.....
115394122    ......EIMGE.....IVKLL.....YMLME.....IIYRD.....VDFGF.....SADYW.....
115707222    ......NIMLE.....IVRLY.....YMLLE.....IVYRD.....VDFGF.....SADYW.....
115733118    ......KIMME.....IVKLF.....YMLME.....IVYRD.....VDFGF.....SCDYW.....
116109346    ......QIMQG.....IVRLY.....YMLME.....IIYRD.....VDFGF.....SADYW.....
117616148    ......RILEE.....IVKLY.....YMLLE.....IIYRD.....VDFGF.....SVDFW.....
119393857    ......RIMNE.....IVKLY.....YMLME.....IIYRD.....VDFGF.....SADYW.....
119894089    ......KILEE.....IVKLY.....YMLLE.....IIYRD.....VDFGF.....SVDFW.....
157426955    ......RILLE.....IVKMY.....YMLLE.....IIYRD.....VDFGF.....SVDFW.....
125813857    ......KILQA.....IVRLH.....YMIME.....ILYRD.....VDFGF.....AADFW.....
125830540    ......QIMQE.....IVRLY.....YMLME.....IIYRD.....VDFGF.....SADYW.....
125832733    ......IILQQ.....IVRLF.....YMLLE.....IVYRD.....ADFGF.....GADCW.....
126272695    ......QIMQG.....IVRLY.....YMLME.....IIYRD.....VDFGF.....SADYW.....
126331034    ......KILEE.....IVKFY.....YMLLE.....IIYRD.....VDFGF.....SVDFW.....
148342515    ......QIMQG.....IVRLY.....YMLME.....IIYRD.....VDFGF.....SADYW.....
148688379    ......RILEE.....IVKLY.....YMLLE.....IIYRD.....VDFGF.....SVDFW.....
148709762    ......QIMQG.....IVRLY.....YMLME.....IIYRD.....VDFGF.....SADYW.....
149062700    ......QIMQG.....IVRLY.....YMLME.....IIYRD.....VDFGF.....SADYW.....
149604537    ......RILEE.....VVKLY.....YMLLE.....VIYRD.....VDFGF.....SVDFW.....
55962534     ......QIMQG.....IVRLY.....YMLME.....IIYRD.....VDFGF.....SADYW.....
57209582     ......QIMQG.....IVRLY.....YMLME.....IIYRD.....VDFGF.....SADYW.....
108707214    ......RVLSS.....VPTLY.....CFLMD.....FVYRD.....SDFDL.....GVDWW.....
115452853    ......DILTK.....VVQLR.....YLVLD.....IMHRD.....TDFGL.....AADWW.....
108862062    ......QILDL.....LPTLY.....CLITD.....IIYRD.....TDFDL.....AVDWW.....
115486852    ......QILDL.....LPTLY.....CLITD.....IIYRD.....TDFDL.....AVDWW.....
108862061    ......QILDL.....LPTLY.....CLITD.....IIYRD.....TDFDL.....AVDWW.....
108862674    ......EILEM.....LPTLY.....CLVME.....VIYRD.....SDFDL.....AVDWW.....
115483707    ......QILDL.....LPTLY.....CLITD.....IIYRD.....TDFDL.....AVDWW.....
108863902    ......QILDL.....LPTLY.....CLITD.....IIYRD.....TDFDL.....AVDWW.....
157128448    ......EALKE.....IVKFV.....YIVLE.....IAHCD.....IDFSF.....SSDLW.....
157128448    ......QVLES.....LVTMH.....HIILD.....IIYRD.....TDFGL.....TVDWW.....
157106184    ......EILLR.....IVSLY.....YLVME.....VVHRD.....CDLGF.....ACDIW.....
157106184    ......NILAD.....IVKLH.....YLILD.....IIYRD.....TDFGL.....AADWW.....
157109405    ......NILEA.....IVELV.....YLILE.....IIYRD.....TDFGL.....AVDWW.....
109073296    ......--LRY.....IITLK.....YLVME.....VVHRD.....CDFGF.....ACDIW.....
109073296    ......DILAE.....IVKLH.....YLILD.....IIYRD.....TDFGL.....SADWW.....
109084673    ......TALKL.....IVKLH.....FLVME.....VVHRD.....IDFGF.....SCDLW.....
109084673    ......QVLEH.....LVTLH.....HLILD.....IIYRD.....TDFGL.....AVDWW.....
109105416    ......NILES.....IVELA.....YLILE.....IIYRD.....TDFGL.....AVDWW.....
109105569    ......AALRL.....VVNLH.....YLVLE.....VVHRD.....IDFGF.....SCDLW.....
109105569    ......SVLEL.....LVTLH.....HLILD.....IIYRD.....TDFGL.....AVDWW.....
109105571    ......AALRL.....VVNLH.....YLVLE.....VVHRD.....IDFGF.....SCDLW.....
109105571    ......SVLEL.....LVTLH.....HLILD.....IIYRD.....TDFGL.....AVDWW.....
109105573    ......SVLEL.....LVTLH.....HLILD.....IIYRD.....TDFGL.....AVDWW.....
109114561    ......NLFLV.....IYLFI.....XLILE.....IIYRD.....TDFGL.....AVDWW.....
109131379    ......----Y.....IITLK.....YLVTD.....VVHRD.....CDFGF.....ACDIW.....
109131379    ......DILVE.....IVKLH.....YLILD.....IVYRD.....TDFGL.....SADWW.....
109131377    ......----Y.....IITLK.....YLVTD.....VVHRD.....CDFGF.....ACDIW.....
109131377    ......DILVE.....IVKLH.....YLILD.....IVYRD.....TDFGL.....SADWW.....
109457756    ......--LRY.....IITLK.....YLVME.....VVHRD.....CDFGF.....ACDVW.....
109457756    ......DILAE.....IVKLH.....YLILD.....IIYRD.....TDFGL.....SADWW.....
109460775    ......--LRY.....IITLK.....YLVME.....VVHRD.....CDFGF.....ACDVW.....
109460775    ......DILAE.....IVKLH.....YLILD.....IIYRD.....TDFGL.....SADWW.....
109460777    ......--LRY.....IITLK.....YLVME.....VVHRD.....CDFGF.....ACDVW.....
109460777    ......DILAE.....IVKLH.....YLILD.....IIYRD.....TDFGL.....SADWW.....
109460779    ......--LRY.....IITLK.....YLVME.....VVHRD.....CDFGF.....ACDVW.....
109460779    ......DILAE.....IVKLH.....YLILD.....IIYRD.....TDFGL.....SADWW.....
109460781    ......DILAE.....IVKLH.....YLILD.....IIYRD.....TDFGL.....SADWW.....
109510713    ......DILVE.....IVKLH.....YLILD.....IIYRD.....TDFGL.....SADWW.....
109510713    ......--LRY.....IITLK.....YVVTE.....VVHRD.....CDFGF.....ACDIW.....
109511028    ......----Y.....IISLK.....YLVTD.....VVHRD.....CDFGF.....ACDIW.....
109511028    ......DILVE.....IVKLH.....YLILD.....IVYRD.....TDFGL.....SADWW.....
110739125    ......EILES.....LPTLY.....CLLTE.....IVYRD.....TDFDL.....AVDWW.....
110758729    ......SLLRS.....IVKLI.....YLIME.....VVHRD.....VDFGF.....SCDLW.....
110758729    ......QVLEA.....LVTLH.....HLILD.....IIYRD.....TDFGL.....AVDWW.....
110767999    ......NILEA.....IVDLM.....YLILE.....IIYRD.....TDFGL.....AVDWW.....
169610485    ......SILES.....IVNLY.....YLILE.....VIYRD.....TDFGL.....AVDWW.....
113195406    ......-----.....FIKLF.....LLIMD.....IIHND.....CDYGL.....HFDWW.....
115435510    ......EILGL.....LPTLY.....CLVME.....IVYRD.....SDFDL.....AVDWW.....
115447165    ......DVLLA.....LPSLR.....GFAID.....VVYRD.....VDFDL.....AVDWW.....
115447661    ......EILQL.....LPTLY.....CLVME.....VVYRD.....SDFDL.....AVDWW.....
```

```
115451955   ......RVLSS......VPTLY......CFLMD......FVYRD......SDFDL......GVDWW......
115457630   ......EIYAL......LPTLY......CLITD......IIYRD......TDFDL......AIDWW......
115474099   ......DILTK......VVQLR......YLVLD......IMHRD......TDFGL......AADWW......
115479857   ......SVLSG......LPSLL......AWAVP......IAYRD......TDFDL......AVDWW......
115483448   ......EILQL......LPTLY......CLVME......VVYRD......SDFDL......AVDWW......
115488564   ......EILEM......LPTLY......CLVME......VIYRD......SDFDL......AVDWW......
159901308   ......QILA-......IPEIY......ALVME......IIHRD......LDFGI......RSDLY......
113911589   ......EVIAL......LPTLY......CLITD......IIYRD......TDFDL......AIDWW......
115400549   ......RMLEH......LCNLR......YIVVD......IIHRD......ADFNV......EVDWW......
115396144   ......TILES......VVKFF......YLILE......VIYRD......TDFGL......ACDWW......
115387583   ......EILAT......IVTLY......YLCME......FIYRD......SDFDL......AVDWW......
114638230   ......AALRL......VVNLH......YLVLE......VVHRD......IDFGF......SCDLW......
114638230   ......SVLEL......LVTLH......HLILD......IIYRD......TDFGL......AVDWW......
114638836   ......NILES......IVELA......YLILE......IIYRD......TDFGL......AVDWW......
114638838   ......NILES......IVELA......YLILE......IIYRD......TDFGL......AVDWW......
114654449   ......TALKL......IVKLH......FLVME......VVHRD......IDFGF......SCDLW......
114654449   ......QVLEH......LVTLH......HLILD......IIYRD......TDFGL......AVDWW......
114669680   ......NILEE......IVDLI......YLILE......IIYRD......TDFGL......AVDWW......
118150568   ......--LRY......IITLK......YLVTE......VVHRD......CDFGF......GCDIW......
115375110   ......ELLRR......VPRLE......YLVME......GVHRD......MDFGS......ADDVY......
115378930   ......ELLSR......VPRLR......YLVMD......GVHRD......VDFGS......ADDVY......
115374001   ......SLLTR......VPVLH......YLVMQ......GVHRD......MDYGA......SADLF......
115373332   ......QVMAN......IPHLR......YIVTD......VLIRD......VDLGS......TGDLY......
72012370f   ......NILEA......IVDLH......YLILA......IIYRD......TDFGL......DVDWW......
115894391   ......TTLQL......IVHLK......YIIME......IVHRD......IDFGF......SCDVW......
115894391   ......QVLEA......LVTLH......NLILD......IIYRD......TDFGL......AVDWW......
115940443   ......TTLQL......IVHLK......YIIME......IVHRD......IDFGF......SCDVW......
115940443   ......QVLEA......LVTLH......NLILD......IIYRD......TDFGL......AVDWW......
116055724   ......EVHAM......IDKLL......YLVLE......YVYRD......ADFGM......EADFW......
116056580   ......TLLRE......VARLM......YMGFE......VVHRD......CDFGS......AVDLW......
116055620   ......TILNQ......VATLF......YFLME......FVYRD......TDFDL......AVDWW......
116061224   ......EVTSE......LMCQH......YMLFD......IIYRD......GDFGY......AVDWW......
55743138f   ......AALRL......VVNLH......YLVLE......VVHRD......IDFGF......SCDLW......
55743138f   ......SVLEL......LVTLH......HLILD......IIYRD......TDFGL......AVDWW......
116309430   ......EILQM......LPTLY......CLVME......VIYRD......SDFDL......SVDWW......
116310251   ......EILQL......LPTLY......CLVME......VVYRD......SDFDL......AVDWW......
169861430   ......EILAT......IVTLY......YFCME......FIYRD......SDFDL......AVDWW......
169852690   ......TALAL......IAGVI......YTILE......LVHRD......TDFGL......SVDWY......
148228068   ......AALRL......IVALH......YLVME......VVHRD......IDFGF......LCDLW......
148228068   ......NVLEH......LVTLH......HLILD......VVYRD......TDFGL......IVDWW......
117616278   ......NILEE......IVDLI......YLILE......IIYRD......TDFGL......AVDWW......
118034843   ......ELKEK......AVRPI......VLVLE......FVHKD......TGFGI......RSDLY......
118084086   ......--LRY......IITLK......YVVTE......VVHRD......CDFGF......ACDIW......
118084086   ......DILVE......IVKLH......YLILD......IIYRD......TDFGL......SADWW......
118088287   ......--LRY......IITLK......YLVME......VVHRD......CDFGF......ACDIW......
118088287   ......DILAE......IVKLH......YLILD......IIYRD......TDFGL......SADWW......
118089497   ......----Y......IITLK......YLVTE......VVHRD......CDFGF......ACDIW......
118089497   ......DILVE......IVKLH......YLILD......IVYRD......TDFGL......SADWW......
118600443   ......SVLEL......LVTLH......HLILD......IIYRD......TDFGL......AVDWW......
90076660    ......NILEE......IVDLI......YLILE......IIYRD......TDFGL......AVDWW......
90080640    ......AALRL......VVNLH......YLVLE......VVHRD......IDFGF......SCDLW......
121699518   ......RMLEH......LCNLR......YIVVD......IIHRD......ADFNV......EVDWW......
121705492   ......AILES......VVKLF......YLILE......VIYRD......TDFGL......ACDWW......
121713674   ......EILAT......IVTLY......YLCME......FIYRD......SDFDL......AVDWW......
119479027   ......EILAT......IVTLY......YLCME......FIYRD......SDFDL......AVDWW......
119470475   ......RMLEH......LCNLR......YIVVD......IIHRD......ADFNV......EVDWW......
119491913   ......AILES......VVKLF......YLILE......VIYRD......TDFGL......ACDWW......
119567908   ......DILAE......IVKLH......YLILD......IIYRD......TDFGL......SADWW......
119567907   ......--LRY......IITLK......YLVME......VVHRD......CDFGF......ACDIW......
119567907   ......DILAE......IVKLH......YLILD......IIYRD......TDFGL......SADWW......
119595026   ......NILES......IVELA......YLILE......IIYRD......TDFGL......AVDWW......
119594668   ......SVLEL......LVTLH......HLILD......IIYRD......TDFGL......AVDWW......
119628203   ......--LRY......IITLK......YLVTE......VVHRD......CDFGF......GCDIW......
119628203   ......DILAD......VVKLH......YLILD......IIYRD......TDFGL......SADWW......
119619381   ......DILVE......IVKLH......YLILD......IIYRD......TDFGL......SADWW......
119628205   ......--LRY......IITLK......YLVTE......VVHRD......CDFGF......GCDIW......
119628205   ......DILAD......VVKLH......YLILD......IIYRD......TDFGL......SADWW......
119901748   ......--LRY......IITLK......YLVTE......VVHRD......CDFGF......ACDIW......
119901748   ......DILAE......IVKLH......YLILD......IIYRD......TDFGL......SADWW......
119903012   ......TALRL......IVKLH......FLVME......VVHRD......IDFGF......SCDLW......
119903012   ......QVLEH......LVTLH......HLILD......IIYRD......TDFGL......AVDWW......
119919377   ......NILES......IVELA......YLILE......IIYRD......TDFGL......AVDWW......
119922867   ......DILVE......IVKLH......YLILD......IVYRD......TDFGL......SADWW......
119922867   ......----Y......IITLK......YLVTD......VVHRD......CDFGF......ACDIW......
```

123406819	......NTLMR......IIRLY......FFVLE......IIYRD......ADFGT......AIDWW......
123484461	......NILLR......IVQLK......YFGLE......IIYRD......TDFGL......EVDWW......
123506379	......NVLIR......IVKLY......YLVLD......IVYRD......TDFGI......TIDWW......
123232698	......DILVE......IVKLH......YLILD......IVYRD......TDFGL......SADWW......
123232698	......---Y......ISLK......YLVTD......VVHRD......CDFGF......ACDIW......
123232696	......DILVE......IVKLH......YLILD......IVYRD......TDFGL......SADWW......
123232696	......---Y......ISLK......YLVTD......VVHRD......CDFGF......ACDIW......
123232697	......DILVE......IVKLH......YLILD......IVYRD......TDFGL......SADWW......
123232697	......---Y......ISLK......YLVTD......VVHRD......CDFGF......ACDIW......
123231821	......QVLEH......LVTLH......HLILD......IVYRD......TDFGL......AVDWW......
123231821	......AALRQ......IVTLH......YLVME......VVHRD......IDFGF......ACDLW......
145475447	......SILSN......LVNIY......FLYLR......IIHRD......TDLGI......AVDYF......
145475765	......YLLSN......LVNMH......YLVMD......VLHRD......TDLGI......ASDYF......
145479675	......QLLSQ......IINMQ......YLVMD......ILHRD......TDMGI......GVDYY......
145485713	......NILSN......LVNIY......FLVLD......SLHRD......TDLGI......AVDYF......
145483487	......NVLSI......IVKLR......FMILD......IIFRD......TDFGL......AVDWY......
145488926	......TLLSQ......LINMI......YLVMD......IIHRD......TDLGI......AVDYF......
145494578	......FLLEH......LVNMH......YLVLD......IIHRD......TDLGV......PVDYY......
145494700	......NLLKQ......IVNMH......YLVLD......IIHRD......TDMGI......AVDFF......
145493846	......NLLSK......IVNIV......YLVMD......IIHRD......TDLGI......GVDYF......
145492503	......TLLCQ......LINMV......YLVMD......IIHRD......TDLGI......AVDYF......
145496047	......ENSAL......ISSQY......LNIDT......ILHRD......TDLGI......ACDYF......
145497825	......FLLEH......IVNMH......YLVLD......IIHRD......TDLGV......PVDYY......
145500542	......YLLEH......LVNMH......YLVLD......IIHRD......TDLGV......PVDYY......
145503978	......ILLSQ......LVNMN......YLIMD......IIHRD......TDFGI......AVDYY......
145508331	......QILST......IINIV......YLAMD......IIHRD......TDFGI......AVDYF......
145513748	......NVLKQ......IVRLK......YFIME......VIYRD......TDFGL......EVDIW......
145513784	......ILLSQ......IANIH......FLVMD......IIHRD......TDFGI......GVDHF......
145509687	......FLLEH......IVNMH......YLVLD......IIHRD......TDLGV......PVDYY......
145509783	......NLLKQ......IVNMH......YLVLD......ILHRD......TDMGI......AVDFF......
145514692	......NLLIQ......IVNMH......YLVLD......IIHRD......TDMGI......SVDFF......
145520559	......QILST......IINII......YLAMD......IIHRD......TDFGI......AVDYF......
145527632	......FLLEQ......IVNMH......YLVLD......IIHRD......TDLGV......PADYY......
145526753	......YLLEH......LVNMH......YLVLD......IIHRD......TDLGV......PVDYY......
145525849	......ALLCQ......LINLV......YLVMD......IIHRD......TDLGI......AVDYF......
145529692	......NVLSI......IVKLR......FMILD......IIFRD......TDFGL......AVDWY......
145533467	......NVLKQ......IVRLK......YFVME......VIYRD......TDFGL......EVDIW......
145536906	......ELLSQ......LVNMC......YLIMD......IIHRD......TDLGI......AVDYF......
145536662	......QLLEI......IVKLK......YLVVE......IVYRD......TDFGL......KCDVW......
145539790	......QLLSQ......IINMQ......YLVMD......ILHRD......TDMGI......GVDYY......
145540172	......KVLEI......ITKLH......YLIME......IIYRD......CDFGL......CCDWW......
145544208	......QLLEI......IVKLI......YLVVE......IKYRD......TDFGL......KCDIW......
145545554	......FLKNY......IELE......NLIFK......IIFGD......TDFGY......MSDWY......
145543494	......YLLEH......LVNMH......FLVLD......IIHRD......TDLGV......PVDYY......
145550257	......YLLEH......LVNMH......YLVLD......IIHRD......TDLGV......PVDYY......
145552948	......NVLSI......IVKLR......FMILD......IIFRD......TDFGL......AVDWY......
145548253	......QILST......IINII......YLAMD......IIHRD......TDFGI......AVDYF......
131888399	......-----......----......YLVTE......VVHRD......CDFGF......ACDIW......
131888399	......DILVE......IVKLH......YLILD......IIYRD......TDFGL......SADWW......
125524625	......DILRT......LPTLY......CLVME......VVYRD......SDFDL......AVDWW......
125537399	......-----......----......----......IVYRD......TDFDL......AVDWW......
125533416	......EILEA......LPRLF......CLLTE......IVYRD......TDFDL......SVDWW......
125535444	......QILDL......LPTLY......CLITD......IIYRD......TDFDL......AVDWW......
125540193	......DVLLA......LPSLR......GFAID......VVYRD......VDFDL......AVDWW......
125540537	......EILQL......LPTLY......CLVME......VVYRD......SDFDL......AVDWW......
125536618	......EILEM......LPTLY......CLVME......VIYRD......SDFDL......AVDWW......
125538997	......EILGL......LPTLY......CLLME......VVYRD......SDFDL......AVDWW......
125540961	......EILQC......LPTLY......CLVME......IIYRD......SDFDL......AVDWW......
125543147	......RVLSS......VPTLY......CFLMD......FVYRD......SDFDL......GVDWW......
125547690	......EIYAL......LPTLY......CLITD......IIYRD......TDFDL......AIDWW......
125548195	......EILQM......LPTLY......CLVME......VIYRD......SDFDL......SVDWW......
158512936	......EILQC......LPTLY......CLVME......IIYRD......SDFDL......AVDWW......
125559622	......DILTK......VVQLR......YLVLD......IMHRD......TDFGL......AADWW......
125561997	......EILQS......LPTLY......CLVME......IIYRD......SDFDL......AVDWW......
125564116	......EILQS......LPTLY......CLVME......IIYRD......SDFDL......AVDWW......
125562953	......EILQC......LPTLY......CLVME......IIYRD......SDFDL......AVDWW......
125569645	......EILGL......LPTLY......CLVME......IVYRD......SDFDL......AVDWW......
125569221	......DILRT......LPTLY......CLVME......VVYRD......SDFDL......AVDWW......
125564178	......SVLSG......LPSLL......AWAVP......IAYRD......TDFDL......AVDWW......
125575701	......EILQL......LPTLY......CLVME......VVYRD......SDFDL......AVDWW......
125575911	......QILDL......LPTLY......CLITD......IIYRD......TDFDL......AVDWW......

```
125580070    ......RILRQ.....LPTLF.....CAVME.....IVYRD.....TDFDL.....AVDWW.....
125590822    ......DVLMS.....LPPRR.....RI--D.....IVYRD.....VDFDL.....SVDWW.....
125587244    ......RVLRR.....LPTMF.....CVVME.....IVYRD.....TDFDL.....GVDWW.....
125581677    ......EILGL.....LPTLY.....CLLME.....VVYRD.....SDFDL.....AVDWW.....
125590307    ......EILQM.....LPTLY.....CLVME.....VIYRD.....SDFDL.....SVDWW.....
125589802    ......EIYAL.....LPTLY.....CLITD.....IIYRD.....TDFDL.....AIDWW.....
125593419    ......EILGL.....LPTLY.....CLVME.....IVYRD.....SDFDL.....AVDWW.....
125606086    ......EILQS.....LPTLY.....CLVME.....IIYRD.....SDFDL.....AVDWW.....
125604911    ......EILQC.....LPTLY.....CLVME.....IIYRD.....SDFDL.....AVDWW.....
125600913    ......EILEA.....LPRLF.....CLLTE.....IVYRD.....TDFDL.....SVDWW.....
125606143    ......SVLSG.....LPSLL.....AWAVP.....IAYRD.....TDFDL.....AVDWW.....
125601531    ......DILTK.....VVQLR.....YLVLD.....IMHRD.....TDFGL.....AADWW.....
125817622    ......AALRQ.....IVTLH.....YLVME.....VVHRD.....IDFGF.....ACDLW.....
125817622    ......QVLEH.....LVTLH.....HLILD.....IVYRD.....TDFGL.....AVDWW.....
125827833    ......SILEE.....IVDLI.....YLILE.....IIYRD.....TDFGL.....AVDWW.....
125834099    ......DILAE.....IVKLH.....YLILD.....IIYRD.....TDFGL.....SADWW.....
125834099    ......--LRY.....IITLK.....YLVME.....VVHRD.....CDFGF.....ACDIW.....
125837402    ......IIQRQ.....LLSLR.....FIMCD.....IIHRD.....TDFGL.....RAD--.....
125837247    ......IIQRQ.....LLSLR.....FIMCD.....IIHRD.....TDFGL.....RAD--.....
125842169    ......--LRY.....IITLK.....YLVTE.....VVHRD.....CDFGF.....GCDIW.....
125842169    ......DILAD.....VVKLH.....YLILD.....IIYRD.....TDFGL.....SADWW.....
125842404    ......AALKL.....IVKLH.....YLVLE.....VVHRD.....IDFGF.....SCDLW.....
125842404    ......QVLEH.....LVTLH.....HLILD.....IVYRD.....TDFGL.....AVDWW.....
125853579    ......DILVE.....IVKLH.....YLILD.....IIYRD.....TDFGL.....SADWW.....
125853579    ......--LRY.....IITLK.....YLVTE.....VVHRD.....CDFGF.....ACDIW.....
125853591    ......DILVE.....IVKLH.....YLILD.....IIYRD.....TDFGL.....SADWW.....
125853591    ......--LRY.....IITLK.....YLVTE.....VVHRD.....CDFGF.....ACDIW.....
126215735    ......DILVE.....IVKLH.....YLILD.....IVYRD.....TDFGL.....SADWW.....
126215735    ......----Y.....IISLK.....YLVTD.....VVHRD.....CDFGF.....ACDIW.....
126282277    ......TALKL.....IVKLY.....FLVME.....VVHRD.....IDFGF.....SCDLW.....
126282277    ......QVLEH.....LVTLH.....HLILD.....IIYRD.....TDFGL.....AVDWW.....
126307416    ......NILEE.....IVDLI.....YLILE.....IIYRD.....TDFGL.....AVDWW.....
126310777    ......--LRY.....IITLK.....YLVME.....VVHRD.....CDFGF.....ACDIW.....
126310777    ......DILAE.....IVKLH.....YLILD.....IIYRD.....TDFGL.....SADWW.....
126310775    ......--LRY.....IITLK.....YLVME.....VVHRD.....CDFGF.....ACDIW.....
126310775    ......DILAE.....IVKLH.....YLILD.....IIYRD.....TDFGL.....SADWW.....
126325567    ......--LRY.....IITLK.....YVVTE.....VVHRD.....CDFGF.....ACDIW.....
126325567    ......DILVE.....IVKLH.....YLILD.....IIYRD.....TDFGL.....SADWW.....
126328669    ......--LRY.....IITLK.....YLVTE.....VVHRD.....CDFGF.....GCDIW.....
126328669    ......DILAD.....VVKLT.....YLILD.....IIYRD.....TDFGL.....SADWW.....
126338764    ......SILEA.....IVELV.....YLILE.....IIYRD.....TDFGL.....AVDWW.....
126342567    ......DILVE.....IVKLH.....YLILD.....IVYRD.....TDFGL.....SADWW.....
126342567    ......----Y.....IITLK.....YLVTE.....VVHRD.....CDFGF.....ACDIW.....
126343800    ......AALRL.....VVKLQ.....YLVLE.....VVHRD.....IDFGF.....SCDLW.....
126659243    ......QILYT.....IPHFR.....FLVQD.....IIHRD.....IDFGS.....NSDLY.....
134101641    ......EALTK.....VVKLL.....VLDLE.....VWHRD.....FDFSL.....HAERY.....
154334712    ......RMLLM.....IVKFH.....LYVTE.....VIHRD.....IDFGT.....ASDYW.....
154339565    ......RILES.....VAHLL.....VIVME.....IIHRD.....IDFGL.....KGDLF.....
146090520    ......RILES.....VAHLL.....VIVME.....IIHRD.....IDFGL.....VGDLF.....
146082257    ......RMLLM.....IVKFH.....LYVTE.....VIHRD.....IDFGT.....ASDYW.....
145252368    ......TILES.....VVKLF.....YLILE.....VIYRD.....TDFGL.....ACDWW.....
145254868    ......EILAT.....IVTLY.....YLCME.....FIYRD.....SDFDL.....AVDWW.....
145344223    ......ALLRL.....VAALR.....YLGME.....IVHRD.....CDFGS.....AVDLW.....
145355187    ......EVHAT.....IDKLL.....YLVLE.....YVYRD.....ADFGM.....EADIW.....
145355076    ......TILNE.....VATLF.....YFLME.....FVYRD.....TDFDL.....SVDWW.....
145362057    ......EIISL.....LPTLY.....CLITD.....IVYRD.....ADFDL.....AIDWW.....
146185624    ......QLLSS.....LINMI.....YLVMD.....ILHRD.....TDLGI.....AVDYY.....
146185253    ......NVLSL.....IVKLN.....FLILD.....IIFRD.....TDFGL.....AVDWY.....
146185247    ......QILEN.....IINMY.....YLIMD.....IIHRD.....TDFGI.....ESDFF.....
146420351    ......EILAT.....IVTLY.....FLCME.....FIYRD.....SDFDL.....AVDWW.....
146411937    ......QILTN.....IVKLF.....YLILE.....IVYRD.....TDFGL.....AVDWW.....
146415484    ......SILEM.....IVLLY.....YLILE.....VIYRD.....TDFGL.....AVDWW.....
149247126    ......QILEI.....IVKLY.....YLILE.....VIYRD.....TDFGL.....LVDWW.....
149240233    ......EILAT.....IVTLY.....YLCME.....FIYRD.....SDFDL.....AVDWW.....
147817142    ......DILIK.....IVSLR.....YLIMD.....IVHRD.....TDFGL.....DADWW.....
147828664    ......RILKM.....LPTLY.....GIVME.....IIYRD.....SDFSF.....GVDWW.....
147854550    ......EIISM.....LPTLY.....CLITD.....IIYRD.....ADFDL.....AIDWW.....
147857163    ......EILQL.....LPTLY.....CLVME.....VVYRD.....SDFDL.....AVDWW.....
147864073    ......EILEM.....LPALY.....CLLTE.....IVYRD.....TDFDL.....AVDWW.....
147866010    ......DILSA.....LPTLY.....CLLID.....VVYRD.....SDFDL.....GVDWW.....
148472877    ......NILAH.....IVKLH.....YLILD.....IVYRD.....TDFGL.....AADFW.....
148472877    ......--LRH.....VVKLF.....YMIEE.....VAHRD.....VDFGF.....SCDVW.....
148682071    ......----Y.....IISLK.....YLVTD.....VVHRD.....CDFGF.....ACDIW.....
148682071    ......DILVE.....IVKLH.....YLILD.....IVYRD.....TDFGL.....SADWW.....
```

```
148686949   ......TALKL.....IVKLH.....FLVME.....VVHRD.....IDFGF.....SCDLW.....
148686947   ......TALKL.....IVKLH.....FLVME.....VVHRD.....IDFGF.....SCDLW.....
148686947   ......QVLEH.....LVTLH.....HLILD.....IIYRD.....TDFGL.....AVDWW.....
148686948   ......QVLEH.....LVTLH.....HLILD.....IIYRD.....TDFGL.....AVDWW.....
148686948   ......TALKL.....IVKLH.....FLVME.....VVHRD.....IDFGF.....SCDLW.....
148686950   ......TALKL.....IVKLH.....FLVME.....VVHRD.....IDFGF.....SCDLW.....
148708874   ......DILVE.....IVKLH.....YLILD.....IIYRD.....TDFGL.....SADWW.....
148708874   ......--LRY.....ITTLK.....YVVTE.....VVHRD.....CDFGF.....ACDIW.....
148701306   ......SVLEL.....LVTLH.....HLILD.....IIYRD.....TDFGL.....AVDWW.....
148701306   ......AALRL.....VVNLH.....YLV--.....VVHRD.....IDFGF.....SCDLW.....
162462776   ......RILRA.....LPTLF.....SVVTE.....IVYRD.....TDFDL.....AVDWW.....
166091450   ......NILES.....IVELA.....YLILE.....IIYRD.....TDFGL.....AVDWW.....
149024192   ......DILAD.....VVKLH.....YLILD.....IIYRD.....TDFGL.....SADWW.....
149024192   ......--LRY.....ITTLK.....YLVTE.....VVHRD.....CDFGF.....GCDIW.....
149024193   ......--LRY.....ITTLK.....YLVTE.....VVHRD.....CDFGF.....GCDIW.....
149024193   ......DILAD.....VVKLH.....YLILD.....IIYRD.....TDFGL.....SADWW.....
149042423   ......--LRY.....ITTLK.....YVVTE.....VVHRD.....CDFGF.....ACDIW.....
149042423   ......DILVE.....IVKLH.....YLILD.....IIYRD.....TDFGL.....SADWW.....
149055509   ......----Y.....ISSLK.....YLVTD.....VVHRD.....CDFGF.....ACDIW.....
149055509   ......DILVE.....IVKLH.....YLILD.....IVYRD.....TDFGL.....SADWW.....
157818011   ......AALRL.....VVNLH.....YL---.....VVHRD.....IDFGF.....SCDLW.....
157818011   ......SVLEL.....LVTLH.....HLILD.....IIYRD.....TDFGL.....AVDWW.....
150864037   ......TILEK.....IVKLY.....YLILE.....VIYRD.....TDFGL.....SVDWW.....
150865106   ......EILAT.....IVTLY.....YLCME.....FIYRD.....SDFDL.....AVDWW.....
149408606   ......AALKL.....IVKLH.....FLVME.....VVHRD.....IDFGF.....SCDLW.....
149483029   ......DILVE.....IVKLH.....YLILD.....IVYRD.....TDFGL.....SADWW.....
149483029   ......----Y.....ITTLK.....YLVTE.....VVHRD.....CDFGF.....ACDIW.....
149635566   ......NILEE.....IVDLI.....YLILE.....IIYRD.....TDFGL.....AVDWW.....
149637488   ......DILAE.....IVKLH.....YLILD.....IIYRD.....TDFGL.....SADWW.....
149638276   ......--LRY.....ITTLK.....YVVTE.....VVHRD.....CDFGF.....ACDIW.....
149638276   ......DILVE.....IVKLH.....YLILD.....IIYRD.....TDFGL.....SADWW.....
149694207   ......DILAD.....VVKLH.....YLILD.....IIYRD.....TDFGL.....SADWW.....
149694207   ......--LRY.....ITTLK.....YLVTE.....VVHRD.....CDFGF.....GCDIW.....
149724008   ......NILEE.....IVDLI.....YLILE.....IIYRD.....TDFGL.....AVDWW.....
149725474   ......NILES.....IVELA.....YLILE.....IIYRD.....TDFGL.....AVDWW.....
149725476   ......NILES.....IVELA.....YLILE.....IIYRD.....TDFGL.....AVDWW.....
149737588   ......TALKL.....IVKLH.....FLVME.....VVHRD.....IDFGF.....SCDLW.....
149737588   ......QVLEH.....LVTLH.....HLILD.....IIYRD.....TDFGL.....AVDWW.....
149743978   ......--LRY.....ITTLK.....YLVME.....VVHRD.....CDFGF.....ACDIW.....
149743978   ......DILAE.....IVKLH.....YLILD.....IIYRD.....TDFGL.....SADWW.....
149743980   ......--LRY.....ITTLK.....YLVME.....VVHRD.....CDFGF.....ACDIW.....
149743980   ......DILAE.....IVKLH.....YLILD.....IIYRD.....TDFGL.....SADWW.....
149744381   ......DILVE.....IVKLH.....YLILD.....IIYRD.....TDFGL.....SADWW.....
149744381   ......--LRY.....ITTLK.....YVVTE.....VVHRD.....CDFGF.....ACDIW.....
149920348   ......RLGSR.....LVSVH.....YVCMD.....LVHRD.....SDFGI.....AADLF.....
154284492   ......AILES.....VVKLY.....YLILE.....VVYRD.....TDFGL.....ACDWW.....
154274734   ......RMLEH.....LCNLR.....YIVVD.....IIHRD.....ADFNV.....EVDWW.....
154295698   ......SILES.....VVKLF.....YLILE.....VVYRD.....TDFGL.....AVDWW.....
154311789   ......EILAT.....IVTLY.....YLCME.....FIYRD.....SDFDL.....AVDWW.....
154301355   ......RMLEH.....ICNLR.....YLVVD.....IIHRD.....ADFNV.....GADWW.....
151176133   ......EILDM.....LPALY.....CLITD.....IIYRD.....TDFDL.....AVDWW.....
109072810   ......NVLKN.....LVGLH.....YFVLD.....IVYRD.....TDFGL.....TVDWW.....
109092014   ......SVLKN.....LVGLR.....YFVLD.....IIYRD.....TDFGL.....AVDWW.....
109469183   ......NVLKN.....LVGLR.....YFVLD.....IIYRD.....TDFGL.....AVDWW.....
109471255   ......NVLKN.....LVGLR.....YFVLD.....IIYRD.....TDFGL.....AVDWW.....
115495153   ......SVLKN.....LVGLR.....YFVLD.....IIYRD.....TDFGL.....AVDWW.....
114609499   ......NVLKN.....LVGLH.....YFVLD.....IVYRD.....TDFGL.....TVDWW.....
114620355   ......NVLKN.....LVGLH.....YFVLD.....IVYRD.....TDFGL.....TVDWW.....
114620365   ......NVLKN.....LVGLH.....YFVLD.....IVYRD.....TDFGL.....TVDWW.....
114620367   ......NVLKN.....LVGLH.....YFVLD.....IVYRD.....TDFGL.....TVDWW.....
114682163   ......SVLKN.....LVGLR.....YFVLD.....IIYRD.....TDFGL.....AVDWW.....
116267997   ......SVLKN.....LVGLH.....YFVLD.....IVYRD.....TDFGL.....TVDWW.....
115728654   ......SVLKN.....LVSLH.....YFVLD.....IIYRD.....TDFGL.....SVDWW.....
115963951   ......SVLKN.....LVSLH.....YFVLD.....IIYRD.....TDFGL.....SVDWW.....
117558469   ......NVLKN.....LVGLH.....YFVLD.....IIYRD.....TDFGL.....TVDWW.....
118100543   ......NVLKN.....LVGLH.....YFVLD.....IIYRD.....TDFGL.....TVDWW.....
90082519    ......NVLKN.....LVGLH.....YFVLD.....IVYRD.....TDFGL.....TVDWW.....
119367372   ......NVLKN.....LVGLH.....YFVLD.....IVYRD.....TDFGL.....TVDWW.....
126291796   ......NVLKN.....LVGLR.....YFVLD.....IIYRD.....TDFGL.....TVDWW.....
126310584   ......NVLKN.....LVGLH.....YFVLD.....IVYRD.....TDFGL.....TVDWW.....
126321306   ......NVLKN.....LVGLH.....YFVLD.....IVYRD.....TDFGL.....TVDWW.....
126632798   ......SVLKN.....LVGLH.....YFVLD.....IVYRD.....TDFGL.....TVDWW.....
160333875   ......NVLKN.....LVGLH.....YFVLD.....IVYRD.....TDFGL.....TVDWW.....
146182046   ......NIMVN.....IVQLK.....YFVMD.....TIYRD.....CDFGL.....EVDWY.....
149060944   ......NVLKN.....LVGLH.....YFVLD.....IVYRD.....TDFGL.....TVDWW.....
149411098   ......NVLQN.....LVGLH.....YFVLD.....IVYRD.....TDFGL.....TVDWW.....
```

```
149640149   ......NVLKN......LVGLH......YFVLD......IVYRD......TDFGL......TVDWW......
149640151   ......NVLKN......LVGLH......YFVLD......IVYRD......TDFGL......TVDWW......
149642451   ......NVLKN......LVGLH......YFVLD......PHFTD......TDFGL......TVDWW......
149721349   ......NVLKN......LVGLH......YFVLD......IVYRD......TDFGL......TVDWW......
149723217   ......NVLKN......LVGLH......YFVLD......IVYRD......TDFGL......TVDWW......
149733659   ......SVLKN......LVGLR......YFVLD......IIYRD......TDFGL......AVDWW......
57210108    ......SVLKN......LVGLR......YFVLD......IIYRD......TDFGL......AVDWW......
109073700   ......QIMQG......LVNLW......FMVVD......IIHRD......TDFNI......PVDWW......
109073698   ......QIMQG......LVNLW......FMVVD......IIHRD......TDFNI......PVDWW......
109079210   ......QIMQG......LVNLW......FMVVD......IIHRD......TDFNI......AVDWW......
109079212   ......QIMQG......LVNLW......FMVVD......IIHRD......TDFNI......AVDWW......
157821711   ......QIMQG......LVNLW......FMVVD......IIHRD......TDFNI......PVDWW......
116007934   ......ELLSS......LVNLW......FMVCD......VVHRD......TDFNI......PVDWW......
116008547   ......ELLSS......LVNLW......FMVCD......VVHRD......TDFNI......PVDWW......
114602621   ......QIMQG......LVNLW......FMVVD......IIHRD......TDFNI......AVDWW......
114602619   ......QIMQG......LVNLW......FMVVD......IIHRD......TDFNI......AVDWW......
114633414   ......EILQE......LVNLW......FMVVD......IIHRD......TDFNI......EVDWW......
115869839   ......EILQA......LVNLW......FMVVD......IIHRD......TDFNI......PVDWW......
169859296   ......RLLEE......VVNLR......FFVLD......IIHRD......TDFNV......QIDWW......
169849653   ......-----......IVNLR......FFVLD......IMHRD......TDFNI......YIDWW......
117616922   ......QIMQG......LVNLW......FMVVD......IIHRD......TDFNI......PVDWW......
118090690   ......QIMQG......LVNLW......FMVVD......IIHRD......TDFNI......PVDWW......
118093202   ......EILQE......LVNLW......FMGVD......IIHRD......TDFNI......EVDWW......
119569511   ......EILQE......LVNLW......FMVVD......IIHRD......TDFNI......EVDWW......
119582231   ......QIMQG......LVNLW......FMVVD......IIHRD......TDFNI......AVDWW......
119894201   ......QIMQG......LVNLW......FMVVD......IIHRD......TDFNI......PVDWW......
119917965   ......EILQE......LVNLW......FMVVD......IIHRD......TDFNI......EVDWW......
157280003   ......QIMQG......LVNLW......FMVVD......IIHRD......TDFNI......AVDWW......
125848924   ......QIMQN......LVNFW......FMVVD......IIHRD......TDFNI......DVDWW......
126272159   ......EILQD......LVNLW......FMVVD......IIHRD......SDFNI......EVDWW......
126291166   ......QIMQC......LVNLW......FMVVD......IIHRD......TDFNI......AVDWW......
126332052   ......QIMQG......LVNLW......FMVVD......IIHRD......TDFNI......PVDWW......
145608604   ......-----......-----......-----......IIHRD......TDFNV......RADWW......
149636813   ......QIMQG......LVNLW......FMVVD......IIHRD......TDFNI......PVDWW......
149689564   ......EILQE......LVNLW......FMVVD......IIHRD......TDFNI......EVDWW......
149703185   ......QIMQG......LVNLW......FMVVD......IIHRD......TDFNI......PVDWW......
149726764   ......QIMQG......LVNLW......FMVVD......IIHRD......TDFNI......AVDWW......
157132882   ......HVFET......LVGLH......FFVIE......IIYRD......TDYGM......SVDWW......
157114762   ......RVLAL......LVQLH......YFVME......IVYRD......ADFGM......SVDWW......
157110821   ......RILAL......LTALH......FFVME......VIYRD......ADFGM......SVDWW......
109039102   ......RVLTL......LTHLI......FFVME......IIYRD......ADFGM......SVDWW......
109044366   ......HVFEQ......LVGLH......FFVIE......IIYRD......TDYGM......SVDWW......
109102821   ......RILAL......LTQLY......FFVME......VIYRD......ADFGM......SVDWW......
109102815   ......RILAL......LTQLY......FFVME......VIYRD......ADFGM......SVDWW......
109102823   ......RILAL......LTQLY......FFVME......VIYRD......ADFGM......SVDWW......
109102825   ......RILAL......LTQLY......FFVME......VIYRD......ADFGM......SVDWW......
109102819   ......RILAL......LTQLY......FFVME......VIYRD......ADFGM......SVDWW......
109102827   ......RILAL......LTQLY......FFVME......VIYRD......ADFGM......SVDWW......
109125951   ......RVLAL......LTQLH......YFVME......IIYRD......TDFGM......SVDWW......
109125953   ......RVLAL......LTQLH......YFVME......IIYRD......TDFGM......SVDWW......
109125949   ......RVLAL......LTQLH......YFVME......IIYRD......TDFGM......SVDWW......
109127932   ......RVLAL......LTQLH......YFVME......IIYRD......ADFGM......SVDWW......
109127934   ......RVLAL......LTQLH......YFVME......IIYRD......ADFGM......SVDWW......
109127930   ......RVLAL......LTQLH......YFVME......IIYRD......ADFGM......SVDWW......
109127926   ......RVLAL......LTQLH......YFVME......IIYRD......ADFGM......SVDWW......
109127928   ......RVLAL......LTQLH......YFVME......IIYRD......ADFGM......SVDWW......
109464704   ......HVFEQ......LVGLH......FFVIE......IIYRD......TDYGM......SVDWW......
109466476   ......HVFEQ......LVGLH......FFVIE......IIYRD......TDYGM......SVDWW......
109505113   ......RVLSL......LTHMF......FFVME......IVYRD......ADFGM......SVDWW......
110761314   ......RVLAL......LVQLH......YFVME......IVYRD......ADFGM......SVDWW......
110764993   ......HVFET......LVGLH......FFVIE......IIYRD......TDYGM......SVDWW......
116003953   ......RILSL......LTQLF......FFVME......IIYRD......ADFGM......AVDWW......
116007692   ......HVFET......LVGLH......FFVIE......IIYRD......TDYGM......SVDWW......
116007694   ......HVFET......LVGLH......FFVIE......IIYRD......TDYGM......SVDWW......
116007690   ......HVFET......LVGLH......FFVIE......IIYRD......TDYGM......SVDWW......
116007688   ......HVFET......LVGLH......FFVIE......IIYRD......TDYGM......SVDWW......
114550608   ......SIPPE......LVALN......FLVIE......IIYRD......TDYGM......SVDWW......
133908623   ......HVFEQ......LVGLH......FFVIE......IIYRD......TDYGM......SVDWW......
114629235   ......RVLSL......LTHMF......FFVME......IVYRD......ADFGM......SVDWW......
```

```
114653375    ......RILSL......LTQLF......FFVME......IIYRD......ADFGM......AVDWW......
114653371    ......RILSL......LTQLF......FFVME......IIYRD......ADFGM......AVDWW......
114653377    ......RILSL......LTQLF......FFVME......IIYRD......ADFGM......AVDWW......
166795947    ......RILAL......LTQLY......FFVME......VIYRD......ADFGM......SVDWW......
115718038    ......RVLAL......LTALH......FFVME......VIYRD......ADFGM......AVDWW......
115921039    ......HVLGL......LTHLY......FFVME......IIYRD......ADFGM......SVDWW......
115975377    ......RVLAL......LTALH......FFVME......VIYRD......ADFGM......AVDWW......
118404592    ......RVLAL......LTHLH......YFVME......IVYRD......ADFGM......SVDWW......
169858240    ......RVFLT......LLGLH......YFVME......IIYRD......ADYGL......AVDWW......
118150830    ......RVLAL......LTHLF......FFVME......IIYRD......ADFGM......SVDWW......
118092280    ......RILSL......LTKLY......FFVME......IIYRD......ADFGM......DVDWW......
118097952    ......RVLAL......LTQLH......YFVME......IIYRD......ADFGM......SVDWW......
118101016    ......HVFEQ......LVGLH......FLVIE......IIYRD......TDYGM......SVDWW......
119390008    ......RVLAL......LTQLH......YFVME......IIYRD......ADFGM......SVDWW......
121713752    ......RVFLI......LLNLH......YFVME......VIYRD......GDYGL......AVDWW......
119478942    ......RVFLI......LLNLH......YFVME......VIYRD......ADYGL......AVDWW......
119576203    ......RVLAL......LTQLH......YFVME......IIYRD......ADFGM......SVDWW......
119576535    ......HVFEQ......LVGLH......FLVIE......IIYRD......TDYGM......SVDWW......
119884884    ......HVFEQ......LVGLH......FFVIE......IIYRD......TDYGM......SVDWW......
119601206    ......RILSL......LTQLF......FFVME......IIYRD......ADFGM......AVDWW......
119914588    ......RVLAL......LTHLF......FFVME......IIYRD......ADFGM......SVDWW......
119606799    ......RVLSL......LTHMF......FFVME......IVYRD......ADFGM......SVDWW......
119601208    ......RILSL......LTQLF......FFVME......IIYRD......ADFGM......AVDWW......
119905135    ......RVLSL......LTHMF......FFVME......IVYRD......ADFGM......SVDWW......
162287148    ......RILAL......LTQLY......FFVME......VIYRD......ADFGM......SVDWW......
119911049    ......RVLAL......LTQLH......YFVME......IIYRD......TDFGM......SVDWW......
125802902    ......RVLAL......LTHLH......YFVME......IIYRD......ADFGM......SVDWW......
125813308    ......RVLAQ......LTQLH......YFVME......RLFQD......ADFGM......SVDWW......
125831524    ......RILAL......LTQLF......FFVME......VIYRD......ADFGM......SVDWW......
176866349    ......RVLSL......LTQLY......FFVME......IVYRD......ADFGM......SVDWW......
125839202    ......RVLAL......LTSLY......YYVME......IIYRD......ADFGM......AVDWW......
126275466    ......RVFLT......LLNLH......YFVME......IVYRD......GDYGL......SVDWW......
126282918    ......RILAL......LTQLF......FFVME......IIYRD......ADFGM......AVDWW......
126308564    ......RVLAL......LTQLH......YFVME......IIYRD......ADFGM......SVDWW......
126303903    ......RILAL......LTQLY......FFVME......VIYRD......ADFGM......SVDWW......
126335171    ......RVLAL......LTQLH......YFVME......IIYRD......ADFGM......SVDWW......
126335173    ......RVLAL......LTQLH......YFVME......IIYRD......ADFGM......SVDWW......
126336610    ......RVLAL......LTHLY......FFVME......IIYRD......ADFGM......SVDWW......
126338379    ......HVFEQ......LVGLH......FFVIE......IIYRD......TDYGM......SVDWW......
126339723    ......HVFEQ......LVGLH......FLVIE......IIYRD......TDYGI......SVDWW......
126339721    ......HVFEQ......LVGLH......FLVIE......IIYRD......TDYGI......SVDWW......
126340346    ......RVLSL......LTHVY......FFVME......IIYRD......ADFGM......SVDWW......
126343293    ......HVFEQ......LVGLH......FLVIE......IIYRD......TDYGM......SVDWW......
126343848    ......HVFEQ......LVGLH......FLVIE......IIYRD......TDYGI......SVDWW......
127802765    ......HVFEQ......LVGLH......FFVIE......IIYRD......TDYGM......SVDWW......
133778989    ......HVFEQ......LVGLH......FFVIE......IIYRD......TDYGM......SVDWW......
148237022    ......RVLSL......LTHLY......FFVME......VVYRD......ADFGM......SVDWW......
154147716    ......HVFEQ......LVGLH......FLVIE......IIYRD......TDYGM......SVDWW......
141794892    ......HVFEQ......LVGLH......FLVIE......IIYRD......TDYGM......SVDWW......
146186667    ......RVLAL......LTQLH......YFVME......IIYRD......ADFGM......SVDWW......
146415368    ......RVFLT......LLNLH......YFVME......IVYRD......GDYGL......AVDWW......
149240453    ......RVFLT......LLNLH......YFVME......IVYRD......ADYGL......SVDWW......
148685332    ......RVLAL......LTQLH......YFVME......IIYRD......ADFGM......SVDWW......
148685333    ......RVLAL......LTQLH......YFVME......IIYRD......ADFGM......SVDWW......
148692812    ......RVLAL......LTHLI......FFVME......IIYRD......ADFGM......SVDWW......
148692811    ......RVLAL......LTHLI......FFVME......IIYRD......ADFGM......SVDWW......
148704556    ......RILSL......LTQLF......FFVME......IIYRD......ADFGM......AVDWW......
149021004    ......RVLSL......LTHMF......FFVME......IVYRD......ADFGM......SVDWW......
157786690    ......RVLAL......LTQLH......YFVME......IIYRD......ADFGM......SVDWW......
149050488    ......RILAL......LTQLY......FFVME......VIYRD......ADFGM......SVDWW......
149048611    ......HVFEQ......LVGLH......FFVIE......IIYRD......TDYGM......SVDWW......
149068002    ......RVLAL......LTQLH......YFVME......IIYRD......ADFGM......SVDWW......
149068003    ......RVLAL......LTQLH......YFVME......IIYRD......ADFGM......SVDWW......
149389041    ......RVFLT......LLNLH......YFVME......IVYRD......GDYGL......SVDWW......
149436978    ......RVLSL......LTHVY......FFVME......IVYRD......ADFGM......SVDWW......
149574552    ......HVFEQ......LVGLH......FFVIE......IIYRD......TDYGM......SVDWW......
149635080    ......RVLAL......LTQLH......YFVME......IIYRD......ADFGM......SVDWW......
149723625    ......RVLAL......LTQLH......YFVME......IIYRD......ADFGM......SVDWW......
149727601    ......RILAL......LTQLY......FFVME......VIYRD......ADFGM......SVDWW......
149731400    ......HVFEQ......LVGLH......FFVIE......IIYRD......TDYGM......SVDWW......
```

```
149737155   ......RILSL......LTQLF......FFVME......IIYRD......ADFGM......AVDWW......
149743729   ......RVLSL......LTHMF......FFVME......IVYRD......ADFGM......SVDWW......
149757255   ......RVLAL......LTQLH......YFVME......IIYRD......ADFGM......SVDWW......
149757056   ......RVLAL......LTQLH......YFVME......IIYRD......TDFGM......SVDWW......
149758352   ......HVFEQ......LVGLH......FLVIE......IIYRD......TDYGM......SVDWW......
154283339   ......RVFLI......LLNLH......YFVME......VIYRD......ADYGL......AVDWW......
154309167   ......RVFLI......LLTLH......YFVME......VIYRD......ADYGL......AVDWW......
157120307   ......RIFEV......LVNLF......CFVME......IIYRD......ADFGL......AVDWW......
109110114   ......RPCLR......LVPVS......GSPSH......IIYRD......ADFGL......AVDWW......
114557566   ......RIFET......LVNLF......CFVME......IVYRD......ADFGL......AVDWW......
114557568   ......RIFET......LVNLF......CFVME......IVYRD......ADFGL......AVDWW......
114557572   ......RIFET......LVNLF......CFVME......IVYRD......ADFGL......AVDWW......
114557570   ......RIFET......LVNLF......CFVME......IVYRD......ADFGL......AVDWW......
114626998   ......RILEA......LLSLL......CFVTE......IIYRD......ADFGL......AVDWW......
114627000   ......RILEA......LLSLL......CFVTE......IIYRD......ADFGL......AVDWW......
114675659   ......RILAA......LVNLF......CFVME......IVYRD......ADFGL......AVDWW......
115527928   ......RIFET......LVNLF......CFVME......IVYRD......ADFGL......AVDWW......
115650806   ......RIFEA......LVNLF......CFVME......IVYRD......ADFGL......AVDWW......
117616644   ......RIFET......LVNLF......CFVME......IVYRD......ADFGL......AVDWW......
118094409   ......RIFET......LVNLF......CFVME......IVYRD......ADFGL......AVDWW......
90081972    ......RILAA......LVNLF......CFVME......IVYRD......TDFGL......AVDWW......
122692517   ......RILAA......LVNLF......CFVME......IVYRD......ADFGL......AVDWW......
119593571   ......RIFET......LVNLF......CFVME......IVYRD......ADFGL......AVDWW......
119593569   ......RIFET......LVNLF......CFVME......IVYRD......ADFGL......AVDWW......
119889898   ......RIFET......LVNLF......CFVME......IVYRD......ADFGL......AVDWW......
119604826   ......RILAA......LVNLF......CFVME......IVYRD......ADFGL......AVDWW......
122065871   ......RIFET......LVNLF......CFVME......IVYRD......ADFGL......AVDWW......
122890948   ......RIFEA......LVNLF......CFVME......IVYRD......ADFGL......AVDWW......
122891175   ......RIFET......LVNLF......CFVME......IVYRD......ADFGL......AVDWW......
125804346   ......RIFEA......LVNLF......CFVME......IVYRD......ADFGL......AVDWW......
125805765   ......RIFET......LVNLF......CFVME......IVYRD......ADFGL......AVDWW......
125816307   ......RIFET......LVNLY......CFVME......IVYRD......ADFGL......AVDWW......
126297764   ......RILEM......LLSLL......CFVTE......IVYRD......ADFGL......AVDWW......
126305915   ......RIFET......LVNLF......CFVME......IVYRD......ADFGL......AVDWW......
148223205   ......RIFET......LVNLF......CFVME......IVYRD......ADFGL......AVDWW......
148676486   ......RILET......LLSLL......CFVTE......IIYRD......ADFGL......AVDWW......
148680095   ......RIFET......LVNLF......CFVME......IVYRD......ADFGL......AVDWW......
157786736   ......RIFET......LVNLF......CFVME......IVYRD......ADFGL......AVDWW......
149039127   ......RILET......LLSLL......CFVTE......IIYRD......ADFGL......AVDWW......
149039128   ......RILET......LLSLL......CFVTE......IIYRD......ADFGL......AVDWW......
149443554   ......RILEV......LLPLL......CFITE......IVYRD......ADFGL......AVDWW......
149709387   ......RIFET......LVNLF......CFVME......IVYRD......ADFGL......AVDWW......
149709383   ......RIFET......LVNLF......CFVME......IVYRD......ADFGL......AVDWW......
149737996   ......RILEA......LLCLL......CFVTE......IIYRD......ADFGL......AVDWW......
149737998   ......RILEA......LLCLL......CFVTE......IIYRD......ADFGL......AVDWW......
108708333   ......RILSS......LVQFY......YIVME......VVHRD......IDFGL......EADMW......
108705987   ......QIMHH......VVAIK......HIVME......VMHRD......IDFGL......ESDVW......
157129371   ......RVLKR......IVQLF......YLIME......VVHRD......SDFGL......AVDVW......
109018533   ......AVLKK......IVTLE......YLVMQ......IVHRD......TDFGL......AVDCW......
109034723   ......AVLHK......IVALD......YLIMQ......IVHRD......SDFGL......AVDCW......
109078211   ......GVLLR......IVSLK......QLIFE......IVHRD......ADFGL......EVDMW......
109088269   ......SLVHT......IVALE......YLVMQ......IVHRD......SDFGL......AVDCW......
109130457   ......SICHM......IVELL......YMVFE......IIHRD......GGFGV......PVDVW......
109132714   ......AVLRR......IVALE......YLAME......IVHRD......SDFGL......PVELW......
118490868   ......VILRL......VIRLK......YIVME......IIHRD......ADFGL......QADLW......
109459220   ......NILKT......IIHLE......YLVME......IVHRD......SDFGL......QCDIW......
109658702   ......SICHM......IVELL......YMVFE......IIHRD......GGFGV......PVDVW......
110742404   ......EIMKH......IVSLK......HIVME......VMHRD......IDFGL......EVDIW......
110738509   ......QILIA......VVQFH......YIVME......LVHRD......TDFGL......ESDVW......
110740798   ......EIMRH......VVTLK......HLVME......VMHRD......IDFGL......EVDIW......
110764289   ......RVLRR......IVQLL......YLVME......VVHRD......SDFGL......AVDVW......
89032786    ......AVLKR......ILTLV......YLVTD......IVHRD......ADFGL......PVDIW......
115440853   ......AIMRH......IVSLR......HLVME......VIHRD......IDFGL......EIDIW......
115447975   ......QIMHH......VVAIK......HIVME......VIHRD......IDFGL......EADVW......
115453157   ......RILSS......LVQFY......YIVME......VVHRD......IDFGL......EADMW......
115460140   ......QIMHH......VVAIK......HIVME......VMHRD......IDFGL......EADVW......
115464625   ......QIMYH......VVELK......HLVME......VIHRD......TDFGL......EADIW......
115473397   ......KILKA......LVQFY......YIVME......VVHRD......IDFGL......EADVW......
115482890   ......KILKA......LVKFY......YIIME......VVHRD......IDFGL......EADMW......
115442708   ......GLVQH......CSSLA......CLSKP......VVHRD......ADFGL......SVDMW......
115384714   ......AVLKR......ILTLV......YLVTD......IVHRD......ADFGL......PVDIW......
115384342   ......EMLQA......IVHFV......YIVTQ......IVHRD......ADFGI......AVDMW......
114572240   ......AVLKK......IVTLE......YLVMQ......IVHRD......TDFGL......AVDCW......
```

114601168	......GVLLR......IKLK......SLVLE......IVHRD......ADFGL......EVDMW......
114629378	......AVLRK......IVALE......YLVMQ......IVHRD......SDFGL......AVDCW......
114629376	......AVLRK......IVALE......YLVMQ......IVHRD......SDFGL......AVDCW......
114804153	......QIMHH......IVELK......HLVME......VMHRD......TDFGL......EADIW......
116003891	......AVLKK......IVTLE......YLVMQ......IVHRD......TDFGL......AVDCW......
116003961	......AVLHK......IVALD......YLIMQ......IVHRD......SDFGL......AVDCW......
115374461	......KVLTS......VISVL......YYTMD......VIHRD......LDFGL......ASDLY......
92110522	......QIMHH......VISIK......HLVME......VMHRD......IDFGL......EADVW......
89032784	......EMLQR......IVKFH......YIVTQ......VVHRD......ADFGI......PVDMW......
115647190	......AILKK......IIYLK......YLVME......IVHRD......TDFGL......QCDIW......
115688630	......AILKQ......IVALL......YLVIE......IVHRD......SDFGL......EIDIW......
115698864	......SVLQS......IIKLV......YLQLE......IVHRD......ADFGL......AVDMW......
115955908	......AILKQ......IVALL......YLVIE......IVHRD......SDFGL......EIDIW......
115976734	......SVLQS......IIKLV......YLQLE......IVHRD......ADFGL......AVDMW......
116055144	......EVMRT......VCYLY......HLLME......IIYRD......TDFGL......PADVW......
116056705	......RILHH......VVELV......YIVME......VMHRD......IDFGL......EADIW......
116056328	......EVLSG......VVELY......FMVCE......VVHRD......IDFGL......ACDMW......
116059799	......-----......-VELV......HLIME......VMHRD......IDFGL......EADVW......
116057901	......ELARS......VVQTY......FVVME......IIHRD......TDFGL......SVDIW......
116058764	......KVMKD......IIRLV......YFVME......IIHRD......ADFGL......ACDAW......
116060050	......DILKV......VLRLH......YVVTE......VLHRD......ADFGL......AIDIW......
169861442	......QIMRG......IVKLY......FLVLE......VVHRD......ADFGL......SVDMW......
169862036	......AVLKK......IVTLH......YLVFD......VVHRD......ADFGL......PVDVW......
169856102	......EVLKG......IVKFY......YLSFE......IVHRD......ADFGI......AVDIW......
116634228	......LVMRR......VIDLY......HLVLE......IVHRD......MDFGL......KSDMW......
116831157	......EIMRQ......IVTLK......HLVME......VMHRD......IDFGL......EIDIW......
116831129	......EIMRC......IVSFK......YLVME......VIHRD......IDFGL......EIDVW......
117616846	......AVLKR......IVTLE......YLVMQ......IVHRD......TDFGL......AVDCW......
133504495	......SICHM......IVELL......YMVFE......IIHRD......GGFGV......PVDVW......
118083992	......SICHM......IVELL......YMVFE......IIHRD......GGFGV......PVDVW......
118102447	......AVLKK......IVTLE......YLVMQ......IVHRD......TDFGL......AVDCW......
118103232	......GVLLR......IIKLK......ALVLE......VVHRD......GDFGL......EVDMW......
118139676	......QTMHH......VVSIK......HIVME......VMHRD......IDFGL......ESDVW......
91992434	......QIMQH......IVEFK......HVVME......VMHRD......TDFGL......EIDIW......
90076896	......GVLLR......IIKLK......SLVLE......IVHRD......ADFGL......EVDMW......
121705376	......AVLKR......ILTLV......YLVTE......IVHRD......ADFGL......PVDIW......
121716080	......KIMKD......IVQYI......YIIME......ITHRD......SDFGL......SVDMW......
121716363	......EMLQA......IVHFV......YIVTQ......IVHRD......ADFGI......AVDMW......
121710430	......QIMRQ......IVQLI......YIVLE......VVHRD......ADFGL......SVDMW......
119481599	......EMLQA......IVHFV......YIVTQ......IVHRD......ADFGI......AVDLW......
119492019	......AVLKR......ILTLV......YLVTE......IVHRD......ADFGL......PVDIW......
119498311	......QIMRQ......IVKLI......YIVLE......VVHRD......ADFGL......SVDMW......
119579796	......SICHM......IVELL......YMVFE......IIHRD......GGFGV......PVDVW......
119593241	......AVLRR......IVALE......YLAME......IVHRD......SDFGL......AVDVW......
119593242	......AVLRR......IVALE......YLAME......IVHRD......SDFGL......AVDVW......
119850848	......SILKT......IIHLE......YLVME......IVHRD......TDFGL......QCDIW......
123703389	......AVLRK......IVALE......YLIMQ......IVHRD......SDFGL......AVDCW......
123500580	......QIQLR......IIKLY......ALVLE......IVHRD......ADFGL......SCDMW......
123229049	......AVLRR......IVALE......YLAME......IVHRD......SDFGL......AVDVW......
145475713	......AIMRR......LMKLF......YVALE......IMHRD......ADFGL......ICDIY......
145475555	......TIMRK......LIKMY......FFILE......VLHRD......TDFGL......KIDIF......
145475703	......QLMQR......IIHLE......YLILE......IMHRD......VDFGL......ICDMF......
145475355	......SILKE......IVSVY......YIITE......VVHRD......IDFGT......KCDVW......
145475457	......NILKN......IVKLF......YLVTE......IVHRD......IDFGT......KCDIW......
145478389	......KRLIK......IVKAY......YVILE......ICHRD......TDFNV......MVDMW......
145475989	......EIMRN......IIKLF......YLVME......ICHRD......IDFGL......SCDIW......
145480381	......FMMRS......IMKLH......QLVIS......IMHRD......GDFGF......SCDVF......
145477961	......KNLKK......IVKME......YVVME......CCHRD......TDFNV......QVDLW......
145479561	......QIMRI......LMKLY......YMCLE......IMHRD......ADFGL......ICDVY......
145477219	......NLLKN......IVKLY......YLITE......IVHRD......IDFGC......KCDVW......
145480305	......NILKN......IVKLY......YLITE......IVHRD......IDFGT......KCDIW......
145476907	......KVMRK......IVKLH......YFVVD......IAHRD......ADFGL......KCDVF......
145476511	......NILSH......FIKLH......YLILD......IIHRD......VDFGL......VCDIF......
145479735	......NILKS......IIKIY......FIITE......IVHRD......IDFGT......KCDIW......
145479633	......KILMD......IVKLY......YLISE......IVHRD......IDFGA......KCDIW......
145478061	......KRLVE......IVKAY......FVIME......ICHRD......TDFNV......MVDMW......
145479393	......NLMAT......IVKLE......YIILE......IMHRD......VDFGL......ICDIF......
145476743	......GILRT......VIKLH......FLVFE......ILHRD......IDFGL......KVDVF......
145478309	......KNLKK......IVAMK......LVIME......CCHRD......TDFNV......QVDLW......
145484260	......EIARL......LMQYY......FVVFE......VLHRD......TDFGL......KVDVY......
145481133	......DILRQ......TSQII......YLIQE......VLHRD......SDFGL......KVDVY......

145485169	......EILRN......IIKLY......YLIME......IAHRD......IDFGL......LCDIW......
145485961	......HILQE......IVKFF......LIEME......IAHRD......TDFGL......AIDIW......
145486009	......KVMRR......LVRLY......YFVLD......CMHRD......ADFGL......KCDVF......
145484135	......NIFIR......CVPYM......L--ME......VVHFD......CDFGF......KCDVF......
145482595	......EINNQ......FVKVK......YIVMD......IMHRD......VDLGL......ACDIF......
145482403	......NLMAT......VVKLE......YLILE......IMHRD......VDFGL......ICDIF......
145480751	......QVMRA......IVQLL......YFVLE......IFHRD......ADFGL......KCDIF......
145483109	......TIMSN......IVKLE......YLILE......IMHRD......VDFGL......ICDMF......
145485640	......QIMRK......LIKMH......LLIQD......ILHRD......ADFGL......KVDIF......
145485729	......EIMSQ......IVKVL......YIVLE......VVHRD......SDFGV......AVDYW......
145482623	......EILKA......IVKVL......YIVTE......IVHRD......IDFGA......KVDVW......
145483793	......RTVEL......VIRVF......YVIME......IVHRD......IDFGL......RCDLW......
145482233	......LVMND......IIKLH......NMVLQ......VMHRD......ADFGL......KCDIF......
145487366	......NILKN......IVKLY......YMITE......IVHRD......IDFGC......KCDIW......
145488651	......EIMRN......IIKLF......YLVME......ICHRD......IDFGL......SCDIW......
145487798	......EILKT......IVKIL......YIITE......IVHRD......IDFGA......KVDVW......
145487762	......FVLQQ......IIKFY......YICMD......LVHRD......IDFGL......KCDVW......
145490166	......EIMRI......IVNLH......NLVLD......VMHRD......GDFGL......ICDIF......
145487760	......YVLQR......IIKFH......YICMD......IIHRD......IDFGL......KCDVW......
145487179	......EILKQ......IVKIL......FIVTE......IVHRD......IDFGT......KCDIW......
145486896	......RVMRR......LVHLH......YFVLD......CIHRD......ADLGL......KCDIF......
145494368	......QIMRQ......IIKIY......CIVME......IIHRD......CDFGL......KVDVY......
145493649	......RVLQK......IIKIH......NLVSD......IIHGD......IGFGS......KCDLW......
145491205	......TIMEL......ILRVF......YMVLE......IVHRD......IDFGI......KCDIW......
145492385	......SILRK......ILKMY......YLVTE......IVHRD......IDFGL......EVDYW......
145491013	......NILKN......IVKLY......YLITE......IVHRD......IDFGT......KCDVW......
145495428	......EILRK......IVRFY......YFVME......VMHRD......IDFGL......KCDDW......
145493385	......EILKR......IIKIY......YIVTE......IVHRD......VDFGT......KCDVW......
145493946	......NLLKN......VVKLY......YLITE......IVHRD......IDFGC......KCDVW......
145495115	......SIMRK......VIKLY......FLVCE......VLHRD......ADF--......KVDIF......
145493569	......RVMRR......LVHLH......YFVLD......CIHRD......ADFGL......KCDIF......
145494744	......NILKN......IVKLY......YLVTE......IVHRD......IDFGT......KCDVW......
145493668	......KVMRR......IVRLH......YFVVD......IAHRD......ADFGL......KCDVF......
145491157	......FIMRS......IPKLY......ELVMT......IMHRD......IDFGF......QCDVY......
145494860	......SIMRK......IIRLY......WLVTD......IIHRD......ADFGL......KVDVF......
145497252	......KLMRQ......IIKLH......YVCLE......IMHRD......ADFGL......VCDIY......
145499878	......SVLRR......TIKFY......FVVEE......IIHRD......KKFQL......KVDVF......
145497675	......SILKE......IVNIY......YIITE......IVHRD......IDFGT......KCDVW......
145497701	......QIMKE......IISFH......NLVLE......IMHRD......ADFGL......KCDIF......
145496527	......YVLQR......IIKFH......YICMD......IIHRD......IDFGL......KCDVW......
145499592	......NVYKQ......ILKLY......YIIIE......IVHRD......VDWGT......KCDIW......
145495816	......NILKN......IVKLY......YLITE......IVHRD......IDFGT......KCDIW......
145499992	......SIMRK......VIKLC......FMVCE......VLHRD......ADFGL......KIDIF......
145496234	......EIMQN......IIKLF......YLVME......ICHRD......IDFGL......LCDVW......
145495724	......FMMWS......IMKLH......RLIIC......IMHQD......GDFGF......CMDCP......
145498855	......EIMRN......IIKLF......YLVME......ICHRD......IDFGL......SCDIW......
145497567	......NILKN......IVKLF......YLVTE......IVHRD......IDFGT......KCDIW......
145499363	......SIMRK......VIKLY......FLVCE......VLHRD......ADFGL......KIDIF......
145500199	......DIYQQ......IVQCK......FLIME......IIHRD......ADFGL......QVDSW......
145502134	......DILKL......IVKLH......YLITE......IIHRD......IDFGT......KVDVW......
145502251	......QVMRK......VVKLH......YFVVD......IAHRD......ADFGL......KCDVF......
145501576	......SIMKK......VIKLY......YLVLE......ILHRD......GDFGL......KIDIY......
145500588	......SILRQ......ITRLF......YIILE......IMHRD......ADFGL......KIDIY......
145500590	......QILRQ......VIKLN......YLILD......ILHRD......ADFGL......KVDVF......
145501522	......AIMRK......LMRLF......---LE......IMHRD......ADFGL......ICDIY......
145504302	......FVLQQ......IIKFY......YICMD......LVHRD......IDFGL......KCDVW......
145501516	......QLMQK......IIHLE......YLILE......IMHRD......VDFGL......ICDMF......
145500740	......NILRK......IVKLF......YLITE......IVHRD......IDFGT......KCDIW......
145501538	......YVLQR......IIKFN......YICME......IVHRD......IDFGM......KCDIW......
145500446	......QIMRQ......IMQMY......YVGLE......IMHRD......ADYGL......VCDIY......
145502571	......KVMRR......LVRLH......YFILD......CIHRD......ADFGL......KCDVF......
145500846	......YIMRI......IVNLH......YLIMD......VMHRD......ADFGL......KCDIF......
145500728	......LLLRQ......VIKLF......YLIYE......IMHRD......ADFGL......KVDIF......
145500262	......SILRQ......IIKLH......YLVME......IMHRD......GDFGL......KVDIF......

145506551	......AILNF......FIRLY......YLILD......IIHRD......VDFGL......VCDIF......
145506731	......DILKQ......IISIY......YIVTE......IVHRD......ADFGT......KCDIW......
145508315	......NILKS......IIKIF......YIVTE......IVHRD......IDFGT......KCDIW......
145505357	......YVLQK......IIKFY......YIIMD......IIHRD......IDFGL......KCDVW......
145508920	......DLLRS......IIKLY......YLVME......IMHRD......ADFGL......KVDVF......
145509266	......HYLRL......IIRLY......IFITD......IFHRD......IDLGL......KVDVY......
145506577	......NILKS......IVRVF......IIVTE......IVHRD......IDFGT......KVDIW......
145508409	......RTVEL......VIRVF......YVVME......IVHRD......IDFGL......KCDLW......
145505403	......QVMRK......LIKLH......YMVLD......IMHRD......ADFGL......ICDVY......
145505790	......YILQK......IIKFH......YICMD......IIHRD......IDFGL......KCDVW......
145507556	......YVLQK......IIKFY......YIIMD......IIHRD......IDFGL......KCDVW......
145505746	......QILKD......IVKIL......YIITE......IVHRD......IDFGA......KVDVW......
145507732	......EILVQ......IIRVY......HFVME......VTHRD......IDFGL......TCDEW......
145507610	......SVMRQ......LIKLH......YMVLD......IMHRD......ADFGL......ICDVY......
145504637	......EILKQ......IVKIL......FIVTE......IVHRD......IDFGS......KCDIW......
145506096	......TIMEL......ILRLY......YMVLE......IVHRD......IDFGI......KCDVW......
145511339	......ENMKK......IMAVY......YIISQ......IIHRD......IDWGL......KCDLW......
145510847	......EIMRI......IVNLH......NLVLD......VMHRD......GDFGL......ICDIF......
145512479	......SVLKT......FLTLK......CLVTS......IIHRD......VDFGL......KVDIF......
145513188	......SILMD......IVKLY......FLISE......IVHRD......IDFGA......KCDIW......
145509501	......QIMRQ......IIKIY......CIVME......IIHRD......CDFGL......KVDVY......
145512527	......EILTQ......VVKLY......YMVLE......IAHRD......SDFGL......AIDFW......
145511530	......KSLQK......IVKVH......YAIME......ICHRD......TDFNV......MVDMW......
145512946	......NLMAA......IVKLE......YIILE......IMHRD......VDFGL......ICDMF......
145513126	......QIMRD......LMKLY......YMGLE......LMHRD......ADFGL......ICDMF......
145510460	......TIMRE......IMKLY......YMGLE......IMHRD......ADFGL......VCDIY......
145512982	......DILRQ......IIKLY......YLVME......ICHRD......IDFGL......LCDIW......
145513078	......DILRA......IIKLY......YLVME......ICHRD......IDFGL......LCDIW......
145509943	......SIMRK......IIRLY......WLVTD......IIHRD......ADFGL......KVDVF......
145510845	......EIMRI......IVNLH......NLVLD......VMHRD......GDFGL......ICDIF......
145512485	......FIMRS......IPKLH......ELIMT......IMHRD......IDFGF......QCDVY......
145513524	......NLLRS......IIKLY......YLVME......IMHRD......ADFGL......KVDVF......
145515866	......KIMRR......IVKLY......YFVLD......VIHRD......ADFGL......KCDVF......
145517384	......LLLRP......VIKLL......YLIYE......IIHRD......ADFGL......KIDIF......
145517762	......QITRL......ICQLY......TLIMD......IFHRD......IDFGL......KVDVY......
145517518	......QIVRT......ITHFY......FIVYE......IMHRD......TDFGL......KIDIY......
145514211	......DILTQ......IIKVF......HFVME......IAHRD......IDFGL......QCDEW......
145514163	......NILKN......IVKLY......YLVTE......IVHRD......IDFGT......KCDVW......
145515277	......TILKN......IVNLF......YLITE......IVHRD......IDFGT......KCDVW......
145517348	......NILKD......IVKLC......YLVTE......IVHRD......IDFGT......KCDVW......
145514656	......NILKN......IVKLY......YLVTE......IVHRD......IDFGT......KCDVW......
145515529	......TIMEL......ILRLY......YMVLE......IVHRD......IDFGI......KCDVW......
145514389	......NILKN......IVKLF......YLVTE......IVHRD......IDFGT......KCDIW......
145516348	......KNLKR......IVSMK......CVIME......CCHRD......TDFNV......QVDLW......
145517748	......QILRL......IVQME......ILVFD......IFHRD......IDFAL......NVDVF......
145514610	......DILTQ......IIKVF......HFVME......IAHRD......IDFGL......QCDEW......
145517716	......SIMRK......VIKMY......YLVLE......ILHRD......CDFGL......KVDVY......
145520038	......DIMRK......VLQLH......YLIVD......ILHRD......ADFGL......KVDMF......
145518267	......EILNL......IIKLY......YLILD......IIHRD......VDFGL......VCDVF......
145518664	......QVMRL......IIKLR......YIVTD......IIHRD......ADFGL......KVDIY......
145518530	......KILRI......CVKFN......YIVME......IMHRD......TDFGL......KVDVY......
145519073	......KVMRK......IVKLH......YFVLD......LIHRD......ADFGL......KCDVF......
145521542	......NVYKQ......ILKLY......YIITE......IVHRD......VDWGT......KCDIW......
145518748	......LLLRP......VIKLL......YLIYE......IMHRD......ADFGL......KIDIF......
145518233	......NILKS......IVRVF......IIATE......IVHRD......IDFGT......KVDIW......
145518079	......DILKQ......IISIY......YIVTE......IVHRD......ADFGT......KCDIW......
145520823	......TILKQ......IVKLY......YLITE......IVHRD......IDFGT......KCDVW......
145522985	......LILKK......ILKVY......SIVTE......IVHRD......VDFGT......KCDVW......
145522734	......NILKN......IVKLY......YLITE......IVHRD......IDFGT......KCDVW......
145524211	......KLMRK......IVKLY......YFILD......IIHRD......ADFGL......KCDVF......
145523093	......DILRL......IVKLH......YMITE......IIHRD......IDFGT......KVDVW......
145523521	......KVMRR......LVRLH......YFILD......CMHRD......ADFGL......KCDVF......
145523235	......EIMSQ......IVKVL......YIVLE......VVHRD......SDFGV......AVDYW......
145524663	......KNLRR......IVSMK......CVIME......CCHRD......TDFNF......QVDLW......
145522566	......TIMEL......ILRVF......YMILE......IVHRD......IDFGI......KCDIW......
145523413	......NTLKE......IVKLH......YLVTE......VVHRD......IDFGT......KCDVW......
145522069	......EILTQ......VVKLY......YMVLE......IVHRD......SDFGL......AIDFW......
145522031	......FIMRS......IPKLY......ELLMT......IMHRD......IDFGF......QCDVF......
145524082	......KVMRR......LVRLH......YFVLD......CIHRD......ADFGL......KCDVF......
145524022	......NTLKE......IVKLH......YLITE......IVHRD......IDFGT......KCDVW......
145525881	......NILKE......IFKLH......YLITE......IVHRD......IDFGS......KCDVW......
145526845	......KVLRI......CVKFY......YIIME......IMHRD......TDFGL......KVDVY......

```
145525457    ......KIMRE.....LMKLN.....YMVLE.....IMHRD.....ADFGL.....ICDIY.....
145525212    ......NILKS.....IVRVF.....IVVTE.....IVHRD.....IDFGT.....KVDIW.....
145527534    ......QIMKD.....IISFH.....NLVLE.....IMHRD.....ADFGL.....KCDIF.....
145525803    ......QIMNE.....IIKLY.....NMVLE.....IMHRD.....ADFGL.....KCDIF.....
145527074    ......QLMQR.....IIQLI.....YMILE.....IMHRD.....VDFGL.....ICDEF.....
145527202    ......TIMRN.....LMKLY.....YVGLE.....IMHRD.....ADFGL.....ICDIY.....
145526855    ......KIMRQ.....IMQMH.....YVALE.....IMHRD.....ADFGL.....VCDIF.....
145526541    ......NILKD.....IVKLC.....YLITE.....IVHRD.....IDFGT.....KCDVW.....
145527154    ......SVIRQ.....TMKFY.....YVIVE.....IMHRD.....TDFKL.....KVDVY.....
145527308    ......SIMRK.....VIKLH.....YFVLD.....ILHRD.....TDFGL.....KIDIF.....
145525713    ......LILRK.....ILKMY.....YLVTE.....IVHRD.....IDFGL.....EVDYW.....
145527378    ......NILKN.....IVKLF.....YLVTE.....IVHRD.....IDFGT.....KCDIW.....
145527901    ......KVMRR.....IVKLH.....YFVLD.....LIHRD.....ADFGL.....KCDVF.....
145527152    ......SIMKK.....VIRIY.....FLVLE.....ILHRD.....GDFGL.....KIDVY.....
145526442    ......KSMFK.....IVKVH.....YVVME.....ICHRD.....TDFNV.....MVDMW.....
145526717    ......QIIRS.....ITKFF.....ILIYE.....ILHRD.....ADFGL.....KIDVY.....
145530247    ......DVMRK.....VIQLY.....YLIID.....IIHRD.....ADFGL.....KVDIF.....
145529413    ......ENMKK.....IMAVY.....YIVSQ.....IMHRD.....IDWGL.....KCDLW.....
145529970    ......RTVEL.....IIRVF.....YVVME.....IVHRD.....IDFGL.....KCDIW.....
145528540    ......SIMRK.....VIKLY.....FLVCE.....VLHRD.....ADFGL.....KVDIF.....
145529800    ......TILKQ.....IVKLY.....YLITE.....IVHRD.....IDFGT.....KCDVW.....
145534171    ......NLMAA.....IVKME.....YLILE.....IMHRD.....VDFGL.....ICDMF.....
145533869    ......SILSQ.....IVQFK.....LIEMA.....VVHRD.....VDFGL.....PVDIW.....
145532304    ......EILKR.....IIKIY.....YIVTE.....IVHRD.....VDFGT.....KCDVW.....
145534057    ......DILRA.....IIKLY.....YLVME.....ICHRD.....IDFGL.....LCDIW.....
145534434    ......MHLRR.....LMRLI.....YQVFD.....VFHRD.....SNFCY.....KIDVF.....
145531723    ......EILSR.....IIRVF.....YFVME.....IIHRD.....IDFGL.....TCDEW.....
145532130    ......RVMRR.....LVHLH.....YFVID.....CIHRD.....ADFGL.....KCDIF.....
145533655    ......NLLRS.....IIKLY.....YLVME.....IMHRD.....ADFGL.....KVDVF.....
145531185    ......DIYQQ.....IVQCK.....FLIME.....IIHRD.....ADFGL.....QIDSW.....
145532847    ......AILMD.....IVKLY.....YLVSE.....IVHRD.....IDFGA.....KCDIW.....
145534416    ......ENMKK.....IMAIY.....YIVSQ.....IMHRD.....IDWGL.....KCDLW.....
145533108    ......SILKE.....IVSVY.....YIITE.....VVHRD.....IDFGT.....KCDVW.....
145532062    ......KMLQK.....IIKIF.....YIVTE.....IVHRD.....IDFGT.....KCDIW.....
145533224    ......NILKN.....IVKLF.....YLVTE.....IVHRD.....IDFGT.....KCDIW.....
145536640    ......NILRN.....IVKLY.....YLITE.....IVHRD.....IDFGT.....KCDVW.....
145536009    ......SVHRR.....ALKFY.....IIVLE.....IIHRD.....IKFQL.....KVDVF.....
145538257    ......NILKN.....IVKLY.....YLITE.....IVHRD.....IDFGT.....KCDVW.....
145537383    ......TIMRE.....IMKLY.....YMGLE.....IMHRD.....ADFGL.....VCDIY.....
145537542    ......DILRQ.....IIKLY.....YLVME.....ICHRD.....IDFGL.....SCDIW.....
145535622    ......DLLRS.....IIKLY.....YLVME.....IMHRD.....ADFGL.....KVDVF.....
145536756    ......EILTQ.....VVKLY.....YMVLE.....IVHRD.....SDFGL.....AIDFW.....
145537908    ......QIMRE.....LMSLY.....YMGLE.....IMHRD.....ADFGL.....VCDVY.....
145536714    ......QIMRI.....LINLI.....YLIMD.....IMHRD.....ADFGL.....ACDIF.....
145539666    ......QIMRI.....LMKLY.....YMCLE.....IMHRD.....ADFGL.....VCDMY.....
145539994    ......ALLKD.....IVKLF.....YLVTE.....IIHRD.....IDFGT.....KCDIW.....
145539476    ......KLMAA.....IVKLE.....YLILE.....IMHRD.....VDFGL.....ICDMF.....
145540497    ......DILKR.....IIKIY.....YIVTE.....IVHRD.....VDFGT.....KCDIW.....
145539860    ......NILKS.....IIKIY.....YIITE.....IVHRD.....IDFGT.....KCDIW.....
145540146    ......RMHQN.....IIQFK.....YIIME.....IVHRD.....VDFNF.....KMDVY.....
145541489    ......TILAS.....IVKFI.....LIIME.....VVHRD.....SDFGL.....NVDIW.....
145542057    ......KLMRQ.....IIKLH.....YVCLE.....IMHRD.....ADFGL.....VCDIY.....
145540587    ......QILRL.....LLTIY.....YFIQE.....VLHRD.....GDFGL.....KVDIY.....
145542428    ......QVMRA.....IVQLV.....YFVLD.....IFHRD.....ADFGL.....KCDIF.....
145540553    ......EILRK.....VITVL.....FIVTE.....VIHRD.....SDFGL.....KADCY.....
145540968    ......DILRQ.....IIKLY.....YLVME.....ICHRD.....IDFGL.....SCDIW.....
145538520    ......QVMRR.....VVKLH.....YFVVD.....IAHRD.....ADFGL.....KCDVF.....
145540034    ......QIMMY.....IVKIY.....YIVYE.....IVHKD.....IDFGA.....S-DVW.....
145540657    ......NILKN.....ILKLF.....YLVTE.....IVHRD.....IDFGT.....KCDLW.....
145539742    ......NILMD.....IVKLY.....YLINE.....IVHRD.....IDFGA.....KCDIW.....
145539632    ......DILRA.....IIKLY.....YLVTE.....ICHRD.....IDFGL.....LCDIW.....
145547204    ......QILMD.....IVKLY.....YLLSE.....IVHRD.....IDFGA.....KCDIW.....
145545845    ......TIMRK.....LMKMY.....YVALE.....IMHRD.....ADFGL.....VCDIY.....
145546675    ......TILKN.....IVNLF.....YLITE.....IVHRD.....IDFGT.....KCDVW.....
145543907    ......EIMRK.....VISVL.....FIVTE.....VIHRD.....SDFGL.....KADCY.....
145543039    ......NILKN.....IVKLY.....YLITE.....IVHRD.....IDFGT.....KCDVW.....
145545871    ......NILKD.....IVNIF.....YLITE.....IVHRD.....IDFGT.....KCDVW.....
145543570    ......NILKN.....IVNIY.....YLITE.....IVHRD.....IDFGT.....KCDIW.....
145544715    ......TIMEL.....ILRLF.....YMVLE.....IVHRD.....-DFGI.....KCDVW.....
145546645    ......QIMRE.....LMKLY.....YLVLE.....IMHRD.....ADFGL.....VCDVY.....
145547948    ......RVMRR.....ILHLY.....YFVLD.....CMHRD.....ADFGL.....KCDVF.....
145544296    ......EILTQ.....VVKLY.....YMVLE.....IVHRD.....SDFGL.....AIDFW.....
```

```
145543931   ......QILRL......LLTIY......YFVQE......VLHRD......GDFGL......KVDIY......
145547350   ......EILRT......IVKVL......YIVTE......IVHRD......IDFGA......KVDVW......
145542995   ......KIMRT......LINLI......YLIMD......IMHRD......ADFGL......ACDIF......
145546995   ......AILMD......IVKLY......YLVSE......IVHRD......IDFGA......KSDIW......
145546390   ......HILKN......IVKLY......YMITE......IVHRD......IDFGC......KCDIW......
145544222   ......QIMRI......LINLI......YLIMD......IMHRD......ADFGL......ACDIF......
145543528   ......SIMRK......LMRMH......YVGLE......IMHRD......ADFGL......VCDIY......
145547098   ......NLMAI......VVKLE......YLILE......IMHRD......VDFGL......ICDIF......
145543855   ......DILKR......IIKIY......YIVTE......IVHRD......VDFGT......KCDIW......
145551689   ......QILQK......IIKIY......YIVTE......IVHRD......IDFGT......KCDIW......
145552092   ......RVMRR......ILHLH......YFILD......CMHRD......ADLGL......KCDIF......
145549806   ......TIMRE......IMKLY......YMGLE......IMHRD......ADFGL......VCDIY......
145550197   ......TIMRK......LMRMY......YVGLE......IMHRD......ADFGL......VCDIY......
145551987   ......TIMRK......LMRMH......YVALE......IMHRD......ADFGL......VCDIY......
145553149   ......TIMRE......IMKLY......YMGLE......IMHRD......ADFGL......VCDIY......
145550118   ......EILKT......IVKVL......YIVTE......IVHRD......IDFGA......KVDVW......
145473563   ......KMLQK......IIKIH......YIVTE......IVHRD......IDFGT......KCDIW......
145551963   ......NILKG......IVNIY......YLITE......IVHRD......IDFGT......KCDLW......
145550722   ......EILRL......FTKLL......YLVFE......YMHRD......SDLGL......KVDIY......
145552609   ......KRLVQ......IVKAY......FVVME......ICHRD......TDFNV......MVDMW......
145473645   ......RVMRK......LVHLH......YFVID......CIHRD......ADFGL......KCDIF......
145552286   ......TILKN......IVNLF......YLITE......IVHRD......IDFGT......KCDVW......
145549309   ......DILRQ......IIKLY......YLVME......ICHRD......IDFGL......SCDIW......
145549133   ......SIMAN......IVKLE......YLILE......IMHRD......VDFGL......ICDMF......
145551083   ......EILRL......FTRLL......YLVFE......YMHRD......SDLGL......KVDIY......
125528093   ......QIMYH......VVELK......HLVME......VIHRD......TDFGL......EADIW......
125571219   ......QIMHH......IVELR......NLIME......VFHRD......TDFGL......EADIW......
125549484   ......QIMHH......VVAIK......HIVME......VMHRD......IDFGL......EADVW......
125528267   ......AIMRH......IVSLR......HLVME......VIHRD......IDFGL......EIDIW......
125526897   ......QIMHH......IVELR......NLIME......VFHRD......TDFGL......EADIW......
125536652   ......QIMHH......IISIR......HVVME......VMHRD......IDFGL......EADVW......
125536654   ......RIMHH......IISIR......HIVME......VMHRD......IDFGL......QADVW......
125535919   ......QIMHH......VVRIR......HIVME......VMHRD......TDFGL......EADVW......
125540710   ......QIMHH......VVAIK......HIVME......VIHRD......IDFGL......EADVW......
125541742   ......QIMHH......VVTIQ......HIVME......VMHRD......IDFGL......EADVW......
125545980   ......QIMHH......VVSIV......QLVME......VMHRD......IDFGL......EVDVW......
125543996   ......RILSS......LVQFY......YIVME......VVHRD......IDFGL......EADMW......
125542233   ......QIMHH......VVAIK......HIVME......VMHRD......IDFGL......ESDVW......
125552816   ......QIMYH......VVELK......HLVME......VIHRD......TDFGL......EADIW......
125553882   ......EVMAR......VVDLK......HLVME......IVHRD......ADFGL......AADVW......
125558852   ......DIMRQ......IVTLR......HLVME......VMHRD......IDFGL......EVDVW......
125558054   ......EVYLS......IKCLS......HVFDR......LVHRD......TDFGL......ESDVW......
125552653   ......AIMRH......IVSLR......HLVME......VIHRD......IDFGL......EIDIW......
125559198   ......KILKA......LVQFY......YIVME......VVHRD......IDFGL......EADVW......
125552800   ......LVMRR......VINLH......HLVLE......IVHRD......MDFGL......ASDMW......
125564042   ......EIMQH......IVTLK......YLVME......VVHRD......ADFGL......KVDVW......
125562377   ......AIMRR......VVRLR......HLVME......VIHRD......IDFGL......EVDVW......
125564359   ......EILRR......VVRLR......HLVME......VMHRD......IDFGL......EADVW......
125575371   ......KILKA......LVKFY......YIIME......VVHRD......IDFGL......EADMW......
125579361   ......QIMHH......IISIR......HVVME......VMHRD......IDFGL......EADVW......
125572527   ......AIMRH......IVSLR......HLVME......VIHRD......IDFGL......EIDIW......
125578642   ......QIMHH......VVRIR......HIVME......VMHRD......TDFGL......EADVW......
125579363   ......RIMHH......IISIR......HIVME......VMHRD......IDFGL......QADVW......
125584270   ......QIMHH......VVTIQ......HIVME......VMHRD......IDFGL......EADVW......
125588181   ......QIMYH......VISIR......HLVME......VMHRD......IDFGL......EADVW......
125583283   ......QIMHH......VVAIK......HIVME......VIHRD......IDFGL......EADVW......
125595897   ......EVMAR......VVDLK......HLVME......IVHRD......ADFGL......AADVW......
125599195   ......QIMHH......VVSIV......HLVME......VMHRD......IDFGL......EVDVW......
125591421   ......QIMHH......VVAIK......HIVME......VMHRD......IDFGL......EADVW......
125595374   ......ATATS......A----......NLVME......VMHRD......TDFGL......EADIW......
125600430   ......QIMQH......IVEFR......HVVME......VMHRD......TDFGL......EIDVW......
125594548   ......AIMRH......IVSLR......HLVME......VIHRD......IDFGL......EIDIW......
125606315   ......EILRR......VVRLR......HLVME......VMHRD......IDFGL......EADVW......
125606007   ......EIMQH......IVTLK......YLVME......VVHRD......ADFGL......KVDVW......
125601109   ......KILKA......LVQFY......YIVME......VVHRD......IDFGL......EADVW......
125604180   ......AIMRR......VVRLR......HLVME......VIHRD......IDFGL......EVDVW......
125828873   ......AVLHK......IVSLE......YLVMQ......IVHRD......SDFGL......AVDCW......
125830232   ......GVLLR......IIRLK......FLILE......VVHRD......ADFGL......EVDMW......
125856286   ......AVLRK......IVALE......YLIMQ......IVHRD......SDFGL......AVDCW......
126135404   ......KLLQE......IVGFR......YIVTQ......IVHRD......ADFGI......PCDIW......
126644378   ......QLLSI......IAKLF......YLIME......IVHRD......IDFGL......KCDIW......
```

```
126644493 ......ELLKK......IMKLF......YIVGE......IVHRD......IDFGL......KCDVW......
126275445 ......QVLKR......ILALV......YLITD......IVHRD......ADFGL......SVDVW......
126306771 ......AVLKK......IVTLE......YLVMQ......IVHRD......TDFGL......AVDCW......
126320672 ......GVLLR......IIKLK......SLVLE......IVHRD......ADFGL......EVDMW......
126325499 ......SICHM......IVELL......YMVFE......IIHRD......GGFGV......PVDVW......
126332441 ......TILKT......IIHLE......YLVME......IVHRD......TDFGL......QCDIW......
126336395 ......AVLHK......IVALD......YLIMQ......IVHRD......SDFGL......AVDCW......
126340367 ......AVLRK......IVALE......YLVMQ......IVHRD......SDFGL......AVDCW......
126342116 ......AVLKK......IVALE......YLAME......IVHRD......SDFGL......AVDVW......
126470867 ......KILHI......IVQIY......HMIID......IAHRD......ADFGS......KVDVW......
156717838 ......AVLKK......IVSLE......YLAME......IVHRD......SDFGL......AVDVW......
136255681 ......SIMKQ......IIHLE......YLVTE......IVHRD......TDFGL......QCDLW......
145232483 ......EMLQA......IVHFV......YIVTQ......IVHRD......ADFGI......AVDMW......
154340128 ......DVMGR......VVQMT......YIILD......VIHRD......ADFGL......KCDIW......
154339680 ......EILRR......IITLY......YFALD......IVHRD......ADFGL......SVDVW......
146100686 ......EILRR......IITLH......YFALD......IVHRD......ADFGL......SVDVW......
146091250 ......DVMGR......VVQMI......YIILD......VIHRD......ADFGL......KCDIW......
145238450 ......QIMRQ......IVKLV......YIVLE......VVHRD......ADFGL......SVDMW......
145252208 ......AVLKR......ILTLV......YLVTD......IVHRD......ADFGL......PVDIW......
134104767 ......AVLRK......IVALE......YLVMQ......IVHRD......SDFGL......AVDCW......
145344348 ......RILHH......VVELV......YIVME......VMHRD......IDFGL......ESDVW......
145346688 ......ELALE......VVKVY......YVVME......IVHRD......VDFGL......AVDMW......
145347715 ......KIMHH......VVELV......HLVME......VMHRD......IDFGL......GADVW......
145351520 ......DILRV......MLRLE......YVVTE......VLHRD......ADFGL......AIDMW......
145356431 ......EVLSE......VVELY......FMICE......IVHRD......IDFGL......ACDMW......
145610749 ......AVLKK......ILTLV......YLVTD......IVHRD......ADFGL......PVDIW......
145604396 ......QIMRQ......IIKMV......YIILE......VVHRD......ADFGL......SVDMW......
145334921 ......QILIA......VVQFH......YIVME......LVHRD......TDFGL......ESDVW......
145559463 ......SICHM......IVELL......YMVFE......IIHRD......GGFGV......PVDVW......
145579755 ......AVLKK......IVTLE......YLVMQ......IVHRD......TDFGL......AVDCW......
146184611 ......EILRL......ILKIY......YIILE......IVHRD......VDWGT......KCDVW......
146182351 ......EILKS......VIKFY......HLVME......ICHRD......IDFGL......RCDIW......
146182326 ......AILRQ......IVKVF......YLISE......IVHRD......IDFGA......KCDVW......
146181511 ......NILKN......ILRLI......YLVTE......IVHRD......IDFGT......KCDVW......
146176302 ......NIMRN......IIKLY......YLVME......ICHRD......IDFGL......GCDIW......
146173227 ......LHLRE......IVKVY......YTIME......VCHRD......TDFNV......NIDIW......
146179021 ......EILKS......IIKVF......YIVTE......ICHRD......VDFGT......KCDIW......
146165567 ......EILTE......VVKLY......YMVLE......IAHRD......SDFGL......TIDYW......
146166031 ......DILKT......ILKIY......YIITE......YAHRD......IDWGT......KVDIW......
146165684 ......ALLKS......ILKIL......FFVTE......IVHRD......IDFGC......KCDIW......
146165813 ......NILKE......IIKLY......YLVTE......VIHRD......IDFGI......QVDNW......
146165668 ......SIFRQ......IAKLY......YLVME......IVHRD......IDFGL......RCDIW......
146163580 ......RLLQQ......IVKFK......VIIME......ILHRD......ADFGL......PVDLW......
146162967 ......QIMKE......CLKLI......NLILE......IIHRD......ADFGL......KVDVF......
146162645 ......EILKQ......IIKIY......YIVTE......IVHRD......VDFGT......KCDIW......
146161904 ......EIMAN......IVKCT......YMVME......IAHRD......SDFGL......AVDYW......
146161589 ......AILRK......LIKLY......FLIYE......IVHRD......SNFFL......KVDIF......
156523074 ......SICHM......IVELL......YMVFE......IIHRD......GGFGV......PVDVW......
166234051 ......QIMHH......IVTIK......HIVME......VMHRD......IDFGL......EADVW......
166234052 ......QIMHH......IVSIK......HIVME......VMHRD......IDFGL......EADVW......
134254746 ......KILKA......VVQFN......YIVME......LVHRD......TDFGL......ESDVW......
134254734 ......QIMHH......VISIK......HVVME......VMHRD......IGFGL......EADVW......
134254738 ......EIMKH......IVTLK......HIVME......VMHRD......IDFGL......EVDVW......
134254736 ......KILKA......VVQFY......YIVME......LVHRD......TDFGL......ESDVW......
146419768 ......KLLQQ......IVSFV......YIVTQ......IVHRD......ADFGI......PCDIW......
146422262 ......TILRQ......IVLFV......YIVQE......IVHRD......ADFGL......EVDMW......
146418783 ......NVLSK......VLRLI......YIILE......IVHRD......ADFGL......AVDIW......
149247827 ......KIMKR......VLQLF......FLILE......IVHRD......ADFGL......AVDIW......
149239907 ......GMLEE......IVGFR......YLVTQ......IVHRD......ADFGI......PCDIW......
149240031 ......TIMRQ......IVRFI......YIVQE......IVHRD......ADFGL......EVDMW......
147780571 ......QIMHH......IISIK......HLVME......VMHRD......IDFGL......EADVW......
147778668 ......LILEH......IVEFK......HLVME......VMHRD......TDFGL......EIDVW......
147777348 ......QIMHH......IVELK......HLVME......VVHRD......TDFGL......EVDIW......
147779603 ......QIMHH......VISIE......HVVME......VMHRD......IDFGL......EADVW......
147781033 ......KVMSL......ILQIV......HMVME......VAHRD......ADFGS......KVDVW......
147784771 ......QIMKR......IVSLK......HIVME......VMHRD......IDFGL......EIDIW......
147784630 ......KILRA......LVQFY......YIVME......VVHRD......IDFGL......EADVW......
147789502 ......KILEA......VVQFH......YIVME......LVHRD......TDFGL......ESDVW......
147814808 ......QIMHH......IVELK......NLVME......VMHRD......TDFGL......EADIW......
147821643 ......KIMQL......VVQIF......DIILE......VAHRD......GDFGS......KVDVW......
```

147833399	......KILKA......LVKFH......YIIME......VVHRD......IDFGL......EADVW......
147852275	......KILRA......LVQFY......YIVME......VVHRD......IDFGL......EADVW......
147864481	......QIMHH......IVTIK......HIVME......VMHRD......IDFGL......EADVW......
148676018	......AVLRK......IVALE......YLVMQ......IVHRD......SDFGL......AVDCW......
148680996	......AVLKR......IVTLE......YLVMQ......IVHRD......TDFGL......AVDCW......
148703759	......SICHM......IVELL......YMVFE......IIHRD......GGFGV......PVDVW......
148697942	......AVLRR......IVALE......YLAME......IVHRD......SDFGL......AVDVW......
148703760	......SICHM......IVELL......YMVFE......IIHRD......GGFGV......PVDVW......
148703761	......SICHM......IVELL......YMVFE......IIHRD......GGFGV......PVDVW......
148607974	......LVMRK......VIHLY......HLILE......IVHRD......MDFGL......KSDIW......
156094495	......ELLKM......IMKLY......YLVSD......VVHRD......IDFGL......KCDIW......
156094163	......DIMKN......VIKLL......YLVME......ICHRD......IDFGL......KCDLW......
156093667	......SLLKS......IIKLF......YLVTE......IVHRD......VDFGL......KCDVW......
156096258	......LIMKQ......IIKLY......YLVLE......IMHRD......IDWGF......RCDIW......
156095781	......EVLRK......IIKLI......YLVLE......VVHRD......IDFGL......KVDIF......
149017144	......GVLLR......IIKLK......SLVLE......IVHRD......ADFGL......EVDMW......
149029926	......AVLRR......IVALE......YLAME......IVHRD......SDFGL......AVDVW......
149044338	......SICHM......IVELL......YMVFE......IIHRD......GGFGV......PVDVW......
149044336	......SICHM......IVELL......YMVFE......IIHRD......GGFGV......PVDVW......
149044335	......SICHM......IVELL......YMVFE......IIHRD......GGFGV......PVDVW......
149044337	......SICHM......IVELL......YMVFE......IIHRD......GGFGV......PVDVW......
149068365	......NILKT......IIHLE......YLVME......IVHRD......SDFGL......QCDIW......
149195781	......RLTAL......IMNIH......YFTMD......VVHLD......CDWGL......QTDVF......
150865936	......LIMKR......IIKLL......FLVLE......IVHRD......ADFGL......SVDIW......
150866405	......TIMRQ......IVRFI......YIVQE......IVHRD......ADFGL......EVDMW......
149389040	......QVLKR......ILALV......YLITD......IVHRD......ADFGL......SVDVW......
149411627	......AVLKK......IVTLE......YLVMQ......IVHRD......TDFGL......AVDCW......
149437019	......AVLRK......IVALE......YLVMQ......IVHRD......SDFGL......AVDCW......
149633969	......-----......-IKLK......ALILE......VVHRD......GDFGL......EVDMW......
149633889	......SICHM......IVELL......YMVFE......IIHRD......GGFGV......PVDVW......
149634126	......AVLHK......IVALD......YLIMQ......IVHRD......SDFGL......AVDCW......
149637356	......AVLKK......IVALE......YLAME......IVHRD......TDFGL......AVDVW......
149642303	......GVLLR......IIKLK......SLILE......IVHRD......ADFGL......EVDMW......
149708654	......AVLKK......IVTLE......YLVMQ......IVHRD......TDFGL......AVDCW......
149720014	......NILKS......IIHLE......YLVME......IVHRD......TDFGL......QCDIW......
149743575	......AVLRK......IVALE......YLVMQ......IVHRD......SDFGL......AVDCW......
149744457	......SICHM......IVELL......YMVFE......IIHRD......GGFGV......PVDVW......
149744455	......SICHM......IVELL......YMVFE......IIHRD......GGFGV......PVDVW......
149744459	......SICHM......IVELL......YMVFE......IIHRD......GGFGV......PVDVW......
154279934	......DMLQR......IVRFR......-----......VVHRD......ADFGI......AVDMW......
154279708	......QIMRQ......IVKLI......YIVLE......VVHRD......ADFGL......SVDMW......
154278267	......AVLKR......ILTLV......YLVTD......IVHRD......ADFGL......PVDIW......
154291477	......AVLKR......ILTLV......YLVTD......IVHRD......ADFGL......PVDIW......
154296422	......SIMRG......IIKLI......YLVLE......VVHRD......ADFGL......SVDMW......
157137568	......RICRK......IVRLH......YLVFD......VVHRD......ADFGL......AVDIW......
109066776	......RICRL......IVRLH......YLVFD......VVHRD......ADFGL......PVDIW......
109075458	......RICRL......IVRLH......YLVFD......IVHRD......ADFGL......PVDMW......
109075454	......RICRL......IVRLH......YLVFD......VVHRD......ADFGL......PVDMW......
109075464	......RICRL......IVRLH......YLVFD......VVHRD......ADFGL......PVDMW......
109075448	......RICRL......IVRLH......YLVFD......IVHRD......ADFGL......PVDMW......
109075452	......RICRL......IVRLH......YLVFD......VVHRD......ADFGL......PVDMW......
109075446	......RICRL......IVRLH......YLVFD......IVHRD......ADFGL......PVDMW......
109075460	......RICRL......IVRLH......YLVFD......IVHRD......ADFGL......PVDMW......
109079282	......RICRL......IVRLH......YLIFD......VVHRD......ADFGL......PVDLW......
109089281	......RICRL......IVRLH......YLVFD......IVHRD......ADFGL......PVDIW......
109075456	......RICRL......IVRLH......YLVFD......VVHRD......ADFGL......PVDMW......
110755823	......RICRK......IVRLH......YLVFD......VVHRD......ADFGL......PVDIW......
118404282	......RICRL......IVRLH......YLIFD......VVHRD......ADFGL......PVDIW......
114602787	......PLCRI......LIRLH......YLIFD......VVHRD......ADFGL......PVDLW......
114602791	......PLCRI......LIRLH......YLIFD......VVHRD......ADFGL......PVDLW......
114602795	......NFCIF......LVRLH......YLIFD......VVHRD......ADFGL......PVDLW......
114595762	......RICRL......IVRLH......YLVFD......VVHRD......ADFGL......PVDMW......
114602813	......NFCIF......LVRLH......YLIFD......VVHRD......ADFGL......PVDLW......
114602807	......PLCRI......LIRLH......YLIFD......VVHRD......ADFGL......PVDLW......
114602785	......-----......-VRLH......YLIFD......VVHRD......ADFGL......PVDLW......
114602815	......NFCIF......LVRLH......YLIFD......VVHRD......ADFGL......PVDLW......
114602809	......PLCRI......LIRLH......YLIFD......VVHRD......ADFGL......PVDLW......
114602803	......NFCIF......LVRLH......YLIFD......VVHRD......ADFGL......PVDLW......
114602811	......NFCIF......LVRLH......YLIFD......VVHRD......ADFGL......PVDLW......
114602805	......RVLVL......VVRLH......YLIFD......VVHRD......ADFGL......PVDLW......
114602799	......PLCRI......LIRLH......YLIFD......VVHRD......ADFGL......PVDLW......
114602797	......RVLVL......VVRLH......YLIFD......VVHRD......ADFGL......PVDLW......
114602819	......PLCRI......LIRLH......YLIFD......VVHRD......ADFGL......PVDLW......
114602801	......NFCIF......LVRLH......YLIFD......VVHRD......ADFGL......PVDLW......

```
114602793    ......NFCIF......LVRLH......YLIFD......VVHRD......ADFGL......PVDLW......
114595766    ......RICRL......IVRLH......YLVFD......VVHRD......ADFGL......PVDMW......
114602789    ......PLCRI......LIRLH......YLIFD......VVHRD......ADFGL......PVDLW......
114631324    ......RICRL......IVRLH......YLVFD......IVHRD......ADFGL......PVDIW......
115646410    ......RICRK......IVRLH......YLVFD......VVHRD......ADFGL......SVDIW......
119574923    ......RICRL......IVRLH......YLVFD......IVHRD......ADFGL......PVDIW......
118092664    ......RICRL......IVRLH......YLVFD......IVHRD......ADFGL......PVDIW......
90074998     ......RICRL......IVRLH......YLVFD......IVHRD......ADFGL......PVDIW......
90075068     ......RICRL......IVRLH......YLVFD......IVHRD......ADFGL......PVDIW......
119574920    ......RICRL......IVRLH......YLVFD......IVHRD......ADFGL......PVDIW......
119574926    ......RICRL......IVRLH......YLVFD......IVHRD......ADFGL......PVDIW......
119626702    ......RICRL......IVRLH......YLVFD......IVHRD......ADFGL......PVDMW......
119626704    ......RICRL......IVRLH......YLVFD......IVHRD......ADFGL......PVDMW......
119626705    ......RICRL......IVRLH......YLVFD......IVHRD......ADFGL......PVDMW......
120537540    ......RICRL......IVRLH......YLLFD......VVHRD......ADFGL......PVDIW......
123227825    ......RICRL......IVRLH......YLLFD......VVHRD......ADFGL......PVDIW......
125803232    ......RICRL......IVRLH......YLVFD......IVHRD......ADFGL......PVDMW......
125828623    ......RICRL......IVRLH......YLLFD......VVHRD......ADFGL......PVDIW......
125833121    ......RICRL......IVRLH......YLVFD......IVHRD......ADFGL......PVDIW......
125834151    ......RICRL......IVRLH......YLVFD......IVHRD......ADFGL......PVDIW......
125834153    ......RICRL......IVRLH......YLVFD......IVHRD......ADFGL......PVDIW......
126215729    ......RICRL......IVRLH......YLVFD......IVHRD......ADFGL......PVDMW......
126272763    ......RICRL......IVRLH......YLVFD......IVHRD......ADFGL......PVDIW......
126272765    ......RICRL......IVRLH......YLVFD......IVHRD......ADFGL......PVDIW......
126272783    ......RICRL......IVRLH......YLVFD......IVHRD......ADFGL......PVDIW......
126272775    ......RICRL......IVRLH......YLVFD......IVHRD......ADFGL......PVDIW......
126272785    ......RICRL......IVRLH......YLVFD......IVHRD......ADFGL......PVDIW......
126272771    ......RICRL......IVRLH......YLVFD......IVHRD......ADFGL......PVDIW......
126272769    ......RICRL......IVRLH......YLVFD......IVHRD......ADFGL......PVDIW......
126272767    ......RICRL......IVRLH......YLVFD......IVHRD......ADFGL......PVDIW......
126272777    ......RICRL......IVRLH......YLVFD......IVHRD......ADFGL......PVDIW......
126272787    ......RICRL......IVRLH......YLVFD......IVHRD......ADFGL......PVDIW......
126272773    ......RICRL......IVRLH......YLVFD......IVHRD......ADFGL......PVDIW......
126272779    ......RICRL......IVRLH......YLVFD......IVHRD......ADFGL......PVDIW......
126272781    ......RICRL......IVRLH......YLVFD......IVHRD......ADFGL......PVDIW......
126291226    ......RICRL......IVRLH......YLIFD......VVHRD......ADFGL......PVDLW......
126302965    ......RICRL......IVRLH......YLVFD......VVHRD......ADFGL......PVDIW......
126302967    ......RICRL......IVRLH......YLVFD......VVHRD......ADFGL......PVDIW......
126302955    ......RICRL......IVRLH......YLVFD......VVHRD......ADFGL......PVDIW......
126302963    ......RICRL......IVRLH......YLVFD......VVHRD......ADFGL......PVDIW......
126302957    ......RICRL......IVRLH......YLVFD......VVHRD......ADFGL......PVDIW......
126302959    ......RICRL......IVRLH......YLVFD......VVHRD......ADFGL......PVDIW......
126302961    ......RICRL......IVRLH......YLVFD......VVHRD......ADFGL......PVDIW......
126331174    ......RICRL......IVRLH......YLVFD......VVHRD......ADFGL......PVDMW......
154147642    ......RICRL......IVRLH......YLVFD......VVHRD......ADFGL......AVDLW......
134025715    ......RICRL......IVRLH......YLIFD......VVHRD......ADFGL......PVDIW......
134302816    ......RICRL......IVRLH......YLIFD......VVHRD......ADFGL......PVDLW......
148669540    ......RICRL......IVRLH......YLVFD......IVHRD......ADFGL......PVDIW......
148669539    ......RICRL......IVRLH......YLVFD......IVHRD......ADFGL......PVDIW......
148669542    ......RICRL......IVRLH......YLVFD......IVHRD......ADFGL......PVDIW......
148677846    ......RICRL......IVRLH......YLIFD......VVHRD......ADFGL......PVDLW......
148708625    ......RICRL......IVRLH......YLVFD......VVHRD......ADFGL......PVDIW......
149025903    ......RICRL......IVRLH......YLVFD......IVHRD......ADFGL......PVDMW......
149047660    ......RICRL......IVRLH......YLVFD......VVHRD......ADFGL......PVDIW......
149047662    ......RICRL......IVRLH......YLVFD......VVHRD......ADFGL......PVDIW......
149064377    ......RICRL......IVRLH......YLIFD......VVHRD......ADFGL......PVDLW......
149265235    ......RICRL......IVRLH......YLVFD......IVHRD......ADFGL......PVDIW......
149412617    ......RICRL......IVRLH......YLIFD......VVHRD......ADFGL......PVDLW......
109066729    ......AVLEL......VINLH......ILVLE......VVHLD......VDFGL......ATDMW......
109081470    ......SILRQ......VITLH......VLILE......IAHFD......IDFGL......EADMW......
109100479    ......RILSL......VIPIH......EKRNH......IVHLD......VDFGM......ATDMW......
109112045    ......SILKE......VITLH......ILILE......IAHFD......IDFGL......EADMW......
109122939    ......NILRE......IITLH......VLILE......IAHFD......IDFGI......EADMW......
109255245    ......AVLEL......VINLH......ILVLE......VVHLD......VDFGL......ATDMW......
110590709    ......NILRE......IITLH......VLILE......IAHFD......IDFGI......EADMW......
110757319    ......GLLAR......IVSLH......VLLLE......VAHLD......IDLGL......GTDLW......
110762010    ......ALLSL......VVRLH......ILVME......IAHLD......CDFEI......AADMW......
114608535    ......AVLEL......VINLH......ILVLE......VVHLD......VDFGL......ATDMW......
114613021    ......AVLEI......VINLH......ILVLE......VVHLD......VDFGL......ATDMW......
114625378    ......SILKE......VITLH......ILILE......IAHFD......IDFGL......EADMW......
114657494    ......SILRQ......VITLH......VLILE......IAHFD......IDFGL......EADMW......
115722888    ......SILEE......IISLH......VLILE......ICHLD......IDFGL......NTDMW......
114205408    ......SILKE......VITLH......ILILE......IAHFD......IDFGL......EADMW......
118150438    ......AVLES......VVGLH......ILVLE......IVHLD......VDFGL......ATDMW......
118104156    ......SILKE......VITLH......ILILE......IAHFD......IDFGL......EADMW......
118137263    ......SILRQ......VITLH......VLILE......IAHFD......IDFGL......EADMW......
```

118137243	......SILRQ.....VITLH.....VLILE.....IAHFD.....IDFGL.....EADMW.....
118137271	......SILRQ.....VITLH.....VLILE.....IAHFD.....IDFGL.....EADMW.....
118137254	......SILRQ.....VITLH.....VLILE.....IAHFD.....IDFGL.....EADMW.....
119390449	......NILRE.....IITLH.....VLILE.....IAHFD.....IDFGI.....EADMW.....
119583130	......SILKE.....VITLH.....ILILE.....IAHFD.....IDFGL.....EADMW.....
119583129	......SILKE.....VITLH.....ILILE.....IAHFD.....IDFGL.....EADMW.....
119583132	......SILKE.....VITLH.....ILILE.....IAHFD.....IDFGL.....EADMW.....
119598063	......SILRQ.....VITLH.....VLILE.....IAHFD.....IDFGL.....EADMW.....
119902480	......SILRQ.....VITLH.....VLILE.....IAHFD.....IDFGL.....EADMW.....
119922339	......SILKE.....VITLH.....ILILE.....IAHFD.....IDFGL.....EADMW.....
147906447	......AILEM.....VVDLH.....ILVME.....VVHLD.....VDFGL.....ATDMW.....
125816713	......SILKE.....VITLH.....ILILE.....ISHFD.....IDFGL.....EADMW.....
125821477	......NILQQ.....IVMLH.....VLILE.....IAHFD.....IDFGL.....EADMW.....
125853000	......AVLEL.....VVNLH.....VLVLE.....VVHLD.....VDFGL.....ATDMW.....
125855139	......NILQE.....IIAMH.....TLVLE.....IAHFD.....IDFGL.....EADMW.....
126277344	......HILQQ.....VIKLH.....VLILE.....IAHFD.....IDFGL.....EADMW.....
126323190	......DILRE.....IITLH.....VLILE.....IAHFD.....IDFGI.....EADMW.....
126326459	......AVLEL.....VINLH.....ILVLE.....IVHLD.....VDFGM.....ATDMW.....
126333919	......NILKE.....VITLH.....ILILE.....IAHFD.....IDFGL.....EADMW.....
126342981	......AVLEL.....VINLH.....ILVLE.....SERED.....VDFGL.....ATDMW.....
126631885	......NILQQ.....IVMLH.....VLILE.....IAHFD.....IDFGL.....EADMW.....
147899107	......AVLEL.....VIKLH.....ILVLE.....IVHLD.....VDFGL.....ATDMW.....
134085657	......AVLEL.....VINLH.....ILVLE.....VVHLD.....VDFGL.....ATDMW.....
148226644	......AVLEL.....VVNLH.....VLVLE.....VVHLD.....VDFGL.....ATDMW.....
148667586	......AVLEL.....VINLH.....ILVLE.....IVHLD.....VDFGM.....ATDMW.....
148699503	......SILRE.....IITLH.....VLILE.....IAHFD.....IDFGI.....EADMW.....
148709354	......SILKE.....VITLH.....ILILE.....IAHFD.....IDFGL.....EADMW.....
153792613	......SILKE.....VITLH.....ILILE.....ISHFD.....IDFGL.....EADMW.....
149042010	......SILRQ.....IITLH.....VLILE.....IAHFD.....IDFGL.....EADMW.....
149459134	......DILRE.....IITLH.....VLILE.....IAHFD.....IDFGI.....EADMW.....
149588168	......AVLEL.....VINLH.....ILVLE.....VVHLD.....VDFGL.....ATDMW.....
149692328	......SILRQ.....VITLH.....VLILE.....IAHFD.....IDFGL.....EADMW.....
149704784	......AVLEL.....VINLH.....ILVLE.....VVHLD.....VDFGL.....ATDMW.....
149755239	......GILKE.....VITLH.....VLILE.....IAHFD.....IDFGL.....EADMW.....
149760001	......NILRE.....IITLH.....VLILE.....IAHFD.....IDFGI.....EADMW.....
149760003	......NILRE.....IITLH.....VLILE.....IAHFD.....IDFGI.....EADMW.....
157123126	......ELHWR.....IVNII.....LVVME.....IAHRD.....TDFGF.....SCDIW.....
157107075	......ETFHH.....ILQLL.....YLVFE.....IAHRD.....CDFDL.....RCDLW.....
109004099	......ETLYQ.....ILELI.....YLVFE.....IAHRD.....CDFDL.....RCDLW.....
109018585	......ELHWR.....IVRIV.....LIVME.....IAHRD.....TDFGF.....SCDMW.....
109018587	......ELHWR.....IVRIV.....LIVME.....IAHRD.....TDFGF.....SCDMW.....
109039378	......DHHWQ.....IVRIL.....LIIME.....IAHRD.....TDFGF.....SCDMW.....
109098783	......RLHMM.....IVQII.....LIVME.....IAHRD.....CDFGF.....SCDLW.....
109122818	......EMLYQ.....VLELI.....YLVFE.....IAHRD.....CDFDL.....RCDLW.....
110763838	......ETFHH.....IIQLI.....YLVFE.....IAHRD.....CDFDL.....RCDLW.....
147900259	......ECHIR.....IVHVL.....LIVMD.....IAHRD.....TDFGF.....SCDMW.....
118403932	......DCHIR.....IVHVL.....LIVME.....IAHRD.....TDFGF.....SCDMW.....
114556374	......ETLYQ.....ILELI.....YLVFE.....IAHRD.....CDFDL.....RCDLW.....
114556364	......ETLYQ.....ILELI.....YLVFE.....IAHRD.....CDFDL.....RCDLW.....
114556356	......ETLYQ.....ILELI.....YLVFE.....IAHRD.....CDFDL.....RCDLW.....
114556368	......ETLYQ.....ILELI.....YLVFE.....IAHRD.....CDFDL.....RCDLW.....
114556370	......ETLYQ.....ILELI.....YLVFE.....IAHRD.....CDFDL.....RCDLW.....
114556350	......ETLYQ.....ILELI.....YLVFE.....IAHRD.....CDFDL.....RCDLW.....
114572227	......ELHWR.....IVRIV.....LIVME.....IAHRD.....TDFGF.....SCDMW.....
114587093	......DHHWQ.....IVRIL.....LIIME.....IAHRD.....TDFGF.....SCDMW.....
114587091	......DHHWQ.....IVRIL.....LIIME.....IAHRD.....TDFGF.....SCDMW.....
114794592	......ETLYQ.....ILELI.....YLVFE.....IAHRD.....CDFDL.....RCDLW.....
115964017	......NLHSR.....IVKII.....LVVME.....IAHRD.....SDFGF.....GCDMW.....
148228669	......EMLYQ.....VLELI.....YLVFE.....IAHRD.....CDFDL.....RCDLW.....
118094607	......ETLYQ.....ILELI.....YLVFE.....IAHRD.....CDFDL.....RCDLW.....
118096722	......EYHWR.....IVHIL.....LIVME.....IAHRD.....TDFGF.....SCDMW.....
118098554	......RLHMM.....IVQII.....LIVME.....IAHRD.....CDFGF.....SCDLW.....
121583879	......ELHWR.....IVRIL.....LIIME.....IAHRD.....TDFGF.....SCDMW.....
119895059	......EMLYQ.....VLELI.....YLVFE.....IAHRD.....CDFDL.....RCDLW.....
119618385	......RLHMM.....IVQII.....LIVME.....IAHRD.....CDFGF.....SCDLW.....
119613937	......ELHWR.....IVRIV.....LIVME.....IAHRD.....TDFGF.....SCDMW.....
119909433	......RLHMM.....IVQII.....LIVME.....IAHRD.....CDFGF.....SCDLW.....
122891035	......RLHMM.....IVKIM.....LIVME.....IAHRD.....CDFGF.....SCDLW.....
156119632	......ELHCR.....IVEIE.....LLVME.....IAHRD.....TDFGF.....SCDMW.....
168229182	......ETLYQ.....ILELI.....YLVFE.....IAHRD.....CDFDL.....RCDLW.....
126031692	......ELHWR.....IVRIV.....LIVME.....IAHRD.....TDFGF.....SCDMW.....

```
126305764      ......ETLYQ.....ILELI.....YLVFE.....IAHRD.....CDFDL.....RCDLW.....
126306759      ......DLHWR.....IVKIV.....LIIME.....IAHRD.....AQMRS.....SCDMW.....
126324189      ......RLHMM.....IVQII.....LIVME.....IAHRD.....CDFGF.....SCDLW.....
126323605      ......EMLYQ.....VLELI.....YLVFE.....IAHRD.....CDFDL.....RCDLW.....
126336080      ......EHHWQ.....IVRIM.....LIVME.....IAHRD.....TDFGF.....SCDMW.....
126722151      ......ETLYH.....ILQLI.....YLIFE.....IAHRD.....CDFGL.....RCDLR.....
145292014      ......NIFKT.....IVQLL.....YLVME.....IAHRD.....CDLDL.....KCDTW.....
145292015      ......NIFKT.....IVQLL.....YLVME.....IAHRD.....CDLDL.....KCDTW.....
145579773      ......ELHWR.....IVRIV.....LIVME.....IAHRD.....TDFGF.....SCDMW.....
145580326      ......ELHWR.....IVRIV.....LIVME.....IAHRD.....TDFGF.....SCDMW.....
148689230      ......DHHWQ.....IVRIL.....LIVME.....IAHRD.....TDFGF.....SCDMW.....
148689231      ......DHHWQ.....IVRIL.....LIVME.....IAHRD.....TDFGF.....SCDMW.....
148707774      ......ELHWR.....IVHIV.....LIVME.....IAHRD.....TDFGF.....SCDMW.....
149242859      ......ELHWR.....IVRIV.....LIVME.....IAHRD.....TDFGF.....SCDMW.....
149694480      ......ETLYQ.....ILELI.....YLVFE.....IAHRD.....CDFDL.....RCDLW.....
149720653      ......RLHMM.....IVQII.....LIVME.....IAHRD.....CDFGF.....SCDLW.....
149728855      ......DHHWQ.....IVRIL.....LIVME.....IAHRD.....TDFGF.....SCDMW.....
108861911      ......SILNI.....ILHLH.....VMIFE.....IGHFD.....IEFGQ.....ATDMW.....
157136400      ......EMLRT.....IASLL.....ILVME.....IAHLD.....VDFGA.....QTDIW.....
157117639      ......AIMRS.....LLQLI.....IMVVE.....IVHLD.....IDFGL.....QSDMW.....
157116486      ......DIMSC.....LIQLF.....YVILE.....IIHLD.....IDFGF.....YTDMW.....
157110444      ......HMMNV.....IAQLY.....CMVME.....IVHLD.....IDFGL.....ATDMW.....
157167707      ......DIMNQ.....LIHLH.....VLIYE.....IIHLD.....IDFGL.....YTDMW.....
109033458      ......SIMNC.....LVQCV.....VMVLE.....IVHLD.....IDFGL.....ATDMW.....
109033454      ......SIMNC.....LVQCV.....VMVLE.....IVHLD.....IDFGL.....ATDMW.....
109033470      ......SIMNC.....LVQCV.....VMVLE.....IVHLD.....IDFGL.....ATDMW.....
109069431      ......SVMNQ.....LIQLY.....VLVME.....ILHLD.....IDFGL.....PTDMW.....
109128385      ......NIMNQ.....LIQLY.....TLVME.....ILHLD.....IDFGL.....PTDMW.....
109468337      ......SILNI.....ILYLH.....VMIFE.....IGHFD.....IEFGQ.....ATDMW.....
109470142      ......SILNI.....ILYLH.....VMIFE.....IGHFD.....IEFGQ.....ATDMW.....
109505717      ......SVMNQ.....LIQLY.....ILVME.....ILHLD.....IDFGL.....ATDMW.....
110349715      ......SILNI.....ILHLH.....VMIFE.....IGHFD.....IEFGQ.....ATDMW.....
110349713      ......SILNI.....ILHLH.....VMIFE.....IGHFD.....IEFGQ.....ATDMW.....
110349719      ......SILNI.....ILHLH.....VMIFE.....IGHFD.....IEFGQ.....ATDMW.....
110349717      ......SILNI.....ILHLH.....VMIFE.....IGHFD.....IEFGQ.....ATDMW.....
110749268      ......DLLRN.....IVRAF.....TIVLE.....RAHLD.....IDLGE.....YTDMW.....
110756547      ......EALRS.....IAMLE.....VFILE.....YSHLD.....VDMGS.....QTDIW.....
110758185      ......RIMNM.....LLLLA.....VMVTE.....VVHLD.....IDFGL.....ESDMW.....
110766631      ......EIMRR.....LIQLY.....YVILE.....ILHLD.....IDFGL.....GTDMW.....
114588887      ......SIMNC.....LVQCV.....VMVLE.....IVHLD.....IDFGL.....ATDMW.....
114588891      ......SIMNC.....LVQCV.....VMVLE.....IVHLD.....IDFGL.....ATDMW.....
114588901      ......SIMNC.....LVQCV.....VMVLE.....IVHLD.....IDFGL.....ATDMW.....
114605202      ......SVMNQ.....LIQLY.....VLVME.....ILHLD.....IDFGL.....PTDMW.....
115663135      ......EIMNK.....LLQCL.....IMVLE.....ILHLD.....IDFGL.....TTDMW.....
115663137      ......DLMSE.....LMALI.....VMILE.....IMHLD.....IDFGL.....LTDMW.....
115681464      ......EVMKK.....LLNLH.....IMILE.....LVHLD.....IDFGL.....PTDMW.....
115768155      ......EIMMI.....LLQLY.....VMILE.....VLHLD.....IDFGL.....GTDMW.....
116008192      ......SIMNC.....LVQCV.....VMVLE.....IVHLD.....IDFGL.....ATDMW.....
116008188      ......SIMNC.....LVQCV.....VMVLE.....IVHLD.....IDFGL.....ATDMW.....
77812699f      ......SILNI.....ILYLH.....VMIFE.....IGHFD.....IEFGQ.....ATDMW.....
77812697f      ......SILNI.....ILYLH.....VMIFE.....IGHFD.....IEFGQ.....ATDMW.....
118086495      ......SVMNQ.....LIQLY.....VLVME.....ILHLD.....IDFGL.....PTDMW.....
118096343      ......NIMNQ.....LIQLY.....TLIME.....ILHLD.....IDFGL.....PTDMW.....
118102815      ......ELMNL.....LVQCL.....VMVME.....VVHLD.....VDFGL.....ATDMW.....
167466233      ......SVMNQ.....LIQLY.....VLVME.....ILHLD.....IDFGL.....PTDMW.....
118573874      ......SVMNQ.....LIQLY.....ILVMD.....ILHLD.....IDFGL.....STDMW.....
119596821      ......EVMNQ.....LIQLY.....VLFME.....VLHLD.....IDFGL.....KTDMW.....
119596820      ......EVMNQ.....LIQLY.....VLFME.....VLHLD.....IDFGL.....KTDMW.....
119603093      ......NIMNQ.....LIQLY.....TLVME.....ILHLD.....IDFGL.....PTDMW.....
119599844      ......SIMNC.....LVQCV.....VMVLE.....IVHLD.....IDFGL.....ATDMW.....
119599852      ......SIMNC.....LVQCV.....VMVLE.....IVHLD.....IDFGL.....ATDMW.....
119599849      ......SIMNC.....LVQCV.....VMVLE.....IVHLD.....IDFGL.....ATDMW.....
119631423      ......SILNI.....ILHLH.....VMIFE.....IGHFD.....IEFGQ.....ATDMW.....
119631420      ......SILNI.....ILHLH.....VMIFE.....IGHFD.....IEFGQ.....ATDMW.....
119631424      ......SILNI.....ILHLH.....VMIFE.....IGHFD.....IEFGQ.....ATDMW.....
119631418      ......SILNI.....ILHLH.....VMIFE.....IGHFD.....IEFGQ.....ATDMW.....
139948193      ......EVMNQ.....LIQLY.....VLFME.....VLHLD.....IDFGL.....KTDMW.....
```

119915940	......SVMNQ.....LIQLY.....VLVME.....ILHLD.....IDFGL.....PTDMW.....
119909925	......NIMNQ.....LIQLY.....TLVME.....VLHLD.....IDFGL.....PTDMW.....
126157499	......SIMNC.....LVQCV.....VMVLE.....IVHLD.....IDFGL.....ATDMW.....
123232572	......SILNI.....ILYLH.....VMIFE.....IGHFD.....IEFGQ.....ATDMW.....
160358754	......SILNI.....ILYLH.....VMIFE.....IGHFD.....IEFGQ.....ATDMW.....
124376290	......SVMNQ.....LIQLY.....VLVME.....ILHLD.....IDFGL.....PTDMW.....
125812656	......ELMNF.....LVQCL.....VMVME.....ILHLD.....IDFGL.....TTDMW.....
125812660	......ELMNF.....LVQCL.....VMVME.....ILHLD.....IDFGL.....TTDMW.....
125818476	......GIMNS.....LVQCM.....VMVME.....IVHLD.....IDFGL.....PTDMW.....
157743338	......GVMNQ.....LIQLY.....TLIME.....ILHLD.....IDFGL.....PTDMW.....
125832237	......QVMNQ.....LIQLY.....ILVLE.....ILHLD.....IDFGL.....NTDMW.....
125844769	......EILQS.....IMAIR.....VLIVE.....VAHFD.....IDFGM.....AADMW.....
126296194	......NIMNQ.....LIQLY.....TLVME.....ILHLD.....IDFGL.....PTDMW.....
126293901	......EVMNQ.....LIQLY.....ILFME.....VLHLD.....IDFGL.....KTDMW.....
126307854	......DILQQ.....IMKLH.....VLVLE.....IAHFD.....TGFEM.....VADMW.....
126322069	......SVMNQ.....IIQLY.....VLVME.....ILHLD.....IDFGL.....PTDMW.....
126325943	......DIMNC.....LVQCV.....VMVLE.....IVHLD.....IDFGL.....ATDMW.....
126326684	......SILNT.....ILYLH.....VMIFE.....IGHFD.....IEFGQ.....ATDMW.....
126363069	......QTMSV.....LVNLH.....VMIYE.....YVHLD.....IDFGL.....YTDMW.....
158937333	......NIMNQ.....LIQLY.....TLVME.....ILHLD.....IDFGL.....PTDMW.....
146219832	......NIMNQ.....LIQLY.....TLVME.....ILHLD.....IDFGL.....PTDMW.....
170015991	......ATLNV.....FLLLH.....VMIYE.....YGHFD.....IELGQ.....VTDMW.....
110005909	......ETLNI.....FLYLH.....VLIYE.....YCHFD.....IEMGQ.....ATDMW.....
148665451	......SIMNC.....LVQCV.....VMVLE.....IVHLD.....IDFGL.....ATDMW.....
148695270	......SILNI.....ILYLH.....VMIFE.....IGHFD.....IEFGQ.....ATDMW.....
148700415	......SVMNQ.....LIQLY.....ILVME.....ILHLD.....IDFGL.....STDMW.....
149031008	......EVMNQ.....LIQLY.....ILFME.....VLHLD.....IDFGL.....KTDMW.....
149045261	......SVMNQ.....LIQLY.....ILVME.....ILHLD.....IDFGL.....ATDMW.....
149060618	......SIMNC.....LVQCV.....VMVLE.....IVHLD.....IDFGL.....ATDMW.....
149412799	......SVMNQ.....IIQLY.....VLVME.....ILHLD.....IDFGL.....PTDMW.....
149579802	......NIMNQ.....LIQLY.....TLVME.....ILHLD.....IDFGL.....PTDMW.....
149639729	......SILNI.....ILYLH.....VMIFE.....IGHFD.....IEFGQ.....ATDMW.....
149699469	......NIMNQ.....LIQLY.....TLIME.....ILHLD.....IDFGL.....PTDMW.....
149731757	......NVMNQ.....LIQLY.....VLVME.....ILHLD.....IDFGL.....PTDMW.....
149733175	......EVMNQ.....LIQLY.....VLFME.....VLHLD.....IDFGL.....KTDMW.....
149733177	......EVMNQ.....LIQLY.....VLFME.....VLHLD.....IDFGL.....KTDMW.....
157137241	......QILRQ.....IIELQ.....FLVFE.....IVHRD.....TDFGF.....EVDIW.....
109066474	......DILRK.....IIQLK.....FLVFD.....IVHRD.....TDFGF.....EVDMW.....
114613552	......DILRK.....IIQLK.....FLVFD.....IVHRD.....TDFGF.....EVDMW.....
114662079	......HILRQ.....IITLI.....FLVFD.....IVHRD.....SDFGF.....EVDLW.....
115631507	......LILNK.....IIALI.....FLVFE.....IVHRD.....SDFGM.....KIDLW.....
117616630	......HILRQ.....IITLI.....FLVFD.....IVHRD.....SDFGF.....EVDLW.....
126314131	......DILRK.....VIQLK.....FLVFD.....IVHRD.....TDFGF.....EVDMW.....
126334508	......QILRQ.....IITLI.....FLVFD.....IVHRD.....SDFGF.....EVDLW.....
149067714	......HILRQ.....IITLI.....FLVFD.....IVHRD.....SDFGF.....EVDLW.....
149725697	......DILRK.....IIQLK.....FLVFD.....IVHRD.....TDFGF.....EVDMW.....
149725825	......HILRQ.....IITLI.....FLVFD.....IVHRD.....SDFGF.....EVDLW.....
109070954	......VLLKK.....VIRLL.....VLILE.....VLHRD.....IDFGS.....SAAVW.....
109094598	......VLLRK.....VIRLL.....LLVLE.....VVHRD.....IDFGS.....SATVW.....
109130673	......ALLWK.....VIRLL.....MLVLE.....VVHRD.....IDFGS.....PATVW.....
112418755	......TLANM.....IIQLL.....VMVME.....VFHRD.....IDFGC.....PATVY.....
114688494	......ALLWK.....VIRLL.....MLVLE.....VVHRD.....IDFGS.....PATVW.....
114794810	......ALLWK.....VIRLL.....MLVLE.....VVHRD.....IDFGS.....PATVW.....
116517274	......VLLKK.....VIKML.....IIVME.....VVHRD.....IDFGS.....SATVW.....
83305340	......VLLKK.....VIRLL.....VLILE.....VLHRD.....IDFGS.....SAAVW.....
119920321	......ALLWK.....VIRLL.....MLVLE.....VVHRD.....IDFGS.....PATVW.....
122890494	......ALNKG.....IIKLL.....IMILE.....VFHRD.....IDFGC.....PATVW.....
122890495	......ALNKG.....IIKLL.....IMILE.....VFHRD.....IDFGC.....PATVW.....
125803274	......ALMTR.....VLQLL.....ILILE.....VLHRD.....LDFGC.....PATVW.....
125803284	......ALMTR.....VLQLL.....ILILE.....VLHRD.....LDFGC.....PATVW.....
125803286	......ALMTR.....VLQLL.....ILILE.....VLHRD.....LDFGC.....PATVW.....
125803297	......ALMTR.....VLQLL.....ILILE.....VLHRD.....LDFGC.....PATVW.....
125803292	......ALMTR.....VLQLL.....ILILE.....VLHRD.....LDFGC.....PATVW.....
125803270	......ALMTR.....VLQLL.....ILILE.....VLHRD.....LDFGC.....PATVW.....
125803726	......VLTKG.....IIQLL.....IMIFE.....VFHRD.....IDFGC.....PATAW.....
125803913	......ALMTP.....VLQLL.....ILILE.....LLAAD.....LDFGC.....PATVW.....
125804240	......STMHY.....IIKLL.....VLILE.....VFHRD.....IDFGC.....PATAF.....
170016003	......ALMTR.....VLQLL.....VLILE.....VLHRD.....LDFGC.....PLTVW.....

```
169153920    ......ALMTR.....VLQLL.....VLILE.....VLHRD.....LDFGC.....PLTVW.....
125805671    ......ALMTR.....VLQLL.....VLILE.....VLHRD.....LDFGC.....PLTVW.....
125805673    ......TLASM.....IIQLL.....VMVME.....VFHRD.....IDFGC.....PATVY.....
125805661    ......ALMTR.....VLQLL.....VLILE.....VLHRD.....LDFGC.....PLTVW.....
125805665    ......TLASM.....IIQLL.....VMVME.....VFHRD.....VDFGC.....PATVY.....
125805570    ......ALMTR.....VLQLL.....VLILE.....VLHRD.....LDFGC.....PLTVW.....
125805770    ......ALTQG.....IIQML.....IMVLE.....VFHRD.....IDFGC.....EATVW.....
125805805    ......ALLVL.....IIKLL.....IMVLE.....VFHRD.....IDFGC.....TTTVW.....
125805805    ......VLQTL.....IIELL.....CMVLE.....VLHRD.....IDFGC.....PATVW.....
125805811    ......ALLVL.....IIKLL.....IMVLE.....VFHRD.....IDFGC.....TTTVW.....
125805811    ......VLQTL.....IIELL.....CMVLE.....VLHRD.....IDFGC.....PATVW.....
125805774    ......ALTQG.....IIQML.....IMVLE.....VFHRD.....IDFGC.....EATVW.....
125805767    ......ALTLL.....IIQML.....IMVLE.....VFHRD.....IDFGC.....EATVW.....
125805813    ......ALLVL.....IIKLL.....IMVLE.....VFHRD.....IDFGC.....TTTVW.....
125805813    ......VLQTL.....IIELL.....CMVLE.....VLHRD.....IDFGC.....PATVW.....
125805879    ......ALSQE.....IIKLL.....IMVLE.....VFHRD.....IDFGC.....PTTVW.....
125806105    ......ALQIL.....ILELL.....LMVLE.....VLHRD.....IDFGC.....PATVW.....
125806103    ......ALHIL.....ILELL.....LMVLE.....VLHRD.....IDFGC.....PATVW.....
125815553    ......ALNKG.....IIKLL.....IMILE.....VFHRD.....IDFGC.....PATVW.....
125815551    ......ALNKG.....IIKLL.....IMILE.....VFHRD.....IDFGC.....PATVW.....
125816423    ......VLLSM.....TVRLF.....HLVME.....VFHND.....IDFGL.....ESTVK.....
125816912    ......ALMTR.....VLQLL.....ILILE.....DLHRD.....LDFGC.....PATVW.....
125816946    ......ALMTR.....VLQLL.....ILILE.....VLHRD.....LDFGC.....PATVW.....
125816929    ......ALMTR.....VLQLL.....ILILE.....VLHRD.....LDFGC.....PATVW.....
125816927    ......ALMTR.....VLQLL.....ILILE.....VLHRD.....LDFGC.....PATVW.....
125816914    ......ALMTR.....VLQLL.....ILILE.....VLHRD.....LDFGC.....PATVW.....
125816938    ......ALMTR.....VLQLL.....ILILE.....VLHRD.....LDFGC.....PATVW.....
125818203    ......ALTIL.....IIQML.....IMILE.....VLHRD.....IDFGC.....AATVW.....
125818267    ......ALTIL.....IIQML.....IMILE.....VLHRD.....IDFGC.....AATVW.....
125818212    ......ALTIL.....IIQML.....IMILE.....VLHRD.....IDFGC.....AATVW.....
125818215    ......ALTIL.....IIQML.....IMILE.....VLHRD.....IDFGC.....AATVW.....
125818206    ......ALTIL.....IIQML.....TMILE.....VLHRD.....IDFGC.....AATVW.....
125818246    ......ALTIL.....IIQML.....IMILE.....VLHRD.....IDFGC.....AATVW.....
125818255    ......ALTIL.....IIQML.....IMILE.....VLHRD.....IDFGC.....AATVW.....
125818705    ......ALLIL.....IIQLL.....IMVLE.....VFHRD.....IDFGC.....PATVY.....
125819849    ......ALMTR.....VLQLL.....ILILE.....VLHRD.....LDFGC.....PATVW.....
125819847    ......ALMTR.....VLQLL.....ILILE.....VLHRD.....LDFGC.....PATVW.....
125821508    ......GLTKG.....IIDLL.....IMVLQ.....VFHRD.....IDFGC.....PATVY.....
125821637    ......GLTKG.....IIDLL.....IMVLQ.....VFHRD.....IDFGC.....PATVY.....
125821539    ......GLTKG.....IIDLL.....IMVLQ.....VFHRD.....IDFGC.....PATVY.....
125821537    ......GLTKG.....IIDLL.....IMVLQ.....VFHRD.....IDFGC.....PATVY.....
125821593    ......GLTKG.....IIDLL.....IMVLQ.....VFHRD.....IDFGC.....PATVY.....
125821510    ......GLTKG.....IIELL.....IMVLQ.....VFHRD.....IDFGC.....PATVY.....
125821591    ......GLTKG.....IIDLL.....IMVLQ.....VFHRD.....IDFGC.....PATVY.....
125821487    ......GLTKG.....IIELL.....IMVLQ.....VFHRD.....IDFGC.....PATVY.....
125821543    ......GLTKG.....IIDLL.....IMVLQ.....VFHRD.....IDFGC.....PATVY.....
125821512    ......GLTKG.....IIDLL.....IMVLQ.....VFHRD.....IDFGC.....PATVY.....
125821516    ......GLTKG.....IIDLL.....IMVLQ.....VFHRD.....IDFGC.....PATVY.....
125821613    ......GLTKG.....IIDLL.....IMVLQ.....VFHRD.....IDFGC.....PATVY.....
125821597    ......GLTKG.....IIDLL.....IMVLQ.....VFHRD.....IDFGC.....PATVY.....
125821626    ......GLTKG.....IIDLL.....IMVLQ.....VFHRD.....IDFGC.....PATVY.....
125821493    ......GLTKG.....IIELL.....IMVLQ.....VFHRD.....IDFGC.....PATVY.....
125821533    ......GLTKG.....IIDLL.....IMVLQ.....VFHRD.....IDFGC.....PATVY.....
125821595    ......GLTKG.....IIDLL.....IMVLQ.....VFHRD.....IDFGC.....PATVY.....
125821541    ......GLTKG.....IIDLL.....IMVLQ.....VFHRD.....IDFGC.....PATVY.....
125821531    ......GLTKG.....IIELL.....IMVLQ.....VFHRD.....IDFGC.....PATVY.....
125821514    ......GLTKG.....IIDLL.....IMVLQ.....VFHRD.....IDFGC.....PATVY.....
125821611    ......GLTKG.....IIDLL.....IMVLQ.....VFHRD.....IDFGC.....PATVY.....
125821483    ......GLTKG.....IIDLL.....IMVLQ.....VFHRD.....IDFGC.....PATVY.....
125821633    ......GLTKG.....IIDLL.....IMVLQ.....VFHRD.....IDFGC.....PATVY.....
125821601    ......GLTKG.....IIDLL.....IMVLQ.....VFHRD.....IDFGC.....PATVY.....
125821489    ......GLTKG.....IIDLL.....IMVLQ.....VFHRD.....IDFGC.....PATVY.....
125821631    ......GLTKG.....IIDLL.....IMVLQ.....VFHRD.....IDFGC.....PATVY.....
125821624    ......GLTKG.....IIDLL.....IMVLQ.....VFHRD.....IDFGC.....PATVY.....
125821599    ......GLTKG.....IIDLL.....IMVLQ.....VFHRD.....IDFGC.....PATVY.....
125822067    ......GLTKG.....IIELL.....IMVLQ.....VLHRD.....IDFGC.....PATVY.....
125823337    ......ALLIL.....IIQLL.....IMVLE.....VLHRD.....IDFGC.....PATVW.....
```

125823329	......ALLIL......IIQLL......IMVLE......VLHRD......IDFGC......PATVW......
125823333	......ALLIL......IIQLL......IMVLE......VLHRD......IDFGC......PATVW......
125823331	......ALLIL......IIQLL......IMVLE......VLHRD......IDFGC......PATVW......
125823352	......ALLIL......IIQLL......IMVLE......VLHRD......IDFGC......PATVW......
125823335	......ALLIL......IIQLL......IMVLE......VLHRD......IDFGC......PATVW......
125823782	......ALMTR......VLQLL......ILILE......VLHRD......LDFGC......PATVW......
125823778	......ALMTR......VLQLL......ILILE......VLHRD......LDFGC......PATVW......
125823791	......ALMTR......VLQLL......ILILE......VLHRD......LDFGC......PATVW......
125823786	......ALMTR......VLQLL......ILILE......VLHRD......LDFGC......PATVW......
125823776	......ALMTR......VLQLL......ILILE......VLHRD......LDFGC......PATVW......
125823822	......TLASM......IIQLL......VMVME......VFHRD......IDFGC......PATVY......
125823852	......TLASM......IIQLL......VMVME......VFHRD......IDFGC......PATVY......
125823948	......TLASM......IIQLL......VMVME......VFHRD......IDFGC......PATVY......
125823834	......TLASM......IIQLL......VMVME......VFHRD......IDFGC......PATVY......
125823826	......TLASM......IIQLL......VMVME......VFHRD......IDFGC......PATVY......
125823888	......TLASM......IIQLL......VMVIE......VFHRD......IDFGC......PATVY......
125823971	......TLASM......IIQLL......VMVME......VFHRD......IDFGC......PATVY......
125823819	......TLASM......IIQLL......VMVME......VFHRD......IDFGC......PATVY......
125823813	......TLASM......IIQLL......VMVME......VFHRD......IDFGC......PATVY......
125823832	......TLASM......IIQLL......VMVME......VFHRD......IDFGC......PATVY......
125823856	......TLASM......IIQLL......VMVME......VFHRD......IDFGC......PATVY......
125823957	......TLASM......IIQLL......VMVME......VFHRD......IDFGC......PATVY......
125823954	......TLASM......IIQLL......VMVME......VFHRD......IDFGC......PATVY......
125823876	......TLASM......IIQLL......VMVME......VFHRD......IDFGC......PATVY......
125823816	......TLASM......IIQLL......VMVME......VFHRD......IDFGC......PATVY......
125823828	......TLASM......IIQLL......VMVME......VFHRD......IDFGC......PATVY......
125823842	......TLASM......IIQLL......VMVME......VFHRD......IDFGC......PATVY......
125823969	......TLASM......IIQLL......VMVME......VFHRD......IDFGC......PATVY......
125823840	......TLASM......IIQLL......VMVME......VFHRD......IDFGC......PATVY......
125823965	......TLASM......IIQLL......VMVME......VFHRD......IDFGC......PATVY......
125823967	......TLASM......IIQLL......VMVME......VFHRD......IDFGC......PATVY......
125823836	......TLASM......IIQLL......VMVME......VFHRD......IDFGC......PATVY......
125824938	......GLMYL......IIQPL......VLVVE......VYHSD......IDFGA......TATVY......
125826031	......ALMLM......IIRLL......ILVLE......VFHRD......IDFGC......PATVW......
125827928	......TLANM......IIQLL......VMVME......VFHRD......IDFGC......PATVY......
125827994	......TLANM......IIQLL......VMVME......VFHRD......IDFGC......PATVY......
125827883	......TLANM......IIQLL......VMVME......VFHRD......IDFGC......PATVY......
125827894	......TLANM......IIQLL......VMVME......VFHRD......IDFGC......PATVY......
125827944	......TLANM......IIQLL......VMVME......VFHRD......IDFGC......PATVY......
125827879	......TLTNM......IIQLL......VMVME......VFHRD......IDFGC......PATVY......
125827992	......TLANM......IIKLL......VMVME......VFHRD......IDFGC......PATVY......
125827934	......TLANM......IIQLL......VMVME......VFHRD......IDFGC......PATVY......
125827938	......TLANM......IIQLL......VMVME......VFHRD......IDFGC......PATVY......
125827900	......TLANM......IIQLL......VMVME......VFHRD......IDFGC......PATVY......
125827996	......TLANM......IIQLL......VMVME......VFHRD......IDFGC......PATVY......
125827940	......TLANM......IIQLL......VMVME......VFHRD......IDFGC......PATVY......
125827946	......TLANM......IIQLL......VMVME......VFHRD......IDFGC......PATVY......
125827916	......TLANM......IIQLL......VMVME......VFHRD......IDFGC......PATVY......
125827904	......TLANM......IIQLL......VMVME......VFHRD......IDFGC......PATVY......
125827985	......TLANM......IIQLL......VMVME......VFHRD......IDFGC......PATVY......
125827912	......TLANM......IIQLL......VMVME......VFHRD......IDFGC......PATVY......
125827906	......TLANM......IIQLL......VMVME......VFHRD......IDFGC......PATVY......
125827896	......TLANM......IIQLL......VMVME......VFHRD......IDFGC......PATVY......
125827960	......TLANM......IIQLL......VMVME......VFHRD......IDFGC......PATVY......
125827887	......TLANM......IIQLL......VMVME......VFHRD......IDFGC......PATVY......
125827971	......TLANM......IIKLL......VMVME......VFHRD......IDFGC......PATVY......
125827950	......TLANM......IIQLL......VMVME......VFHRD......IDFGC......PATVY......
125828672	......ALKTH......VLQLL......VLILE......ILHRD......LDFSC......PATVR......
125828686	......C----......VLQLL......VLILE......ILHRD......LDFGC......PATVW......
125828682	......ALKTH......VLQLL......VLILE......ILHWD......LDFSC......PATVW......
125828678	......C----......VLQLL......VLILE......ILHRD......LDFSC......PATVR......
125829033	......ALMLM......IIRLL......ILVLE......VFHRD......IDFGC......PATVW......
125829994	......ALTKG......IVKLL......FLVLE......VLHRD......TDFGC......PATVW......
125830592	......ALMTR......VLQLL......VLILE......VLHRD......LDFGC......PATVW......
125832776	......TLANM......IIQLL......VMVME......VFHRD......IDFGC......PATVY......
125832800	......TLANM......IIQLL......VMVME......VFHRD......IDFGC......PATVY......
125832696	......TLANM......IIKLL......VMVME......VFHRD......IDFGC......PATVY......

125832798	......TLANM......IIKLL......VMVME......VFHRD......IDFGC......PATVY......
125832782	......TLANM......IIQLL......VMVME......VFHRD......IDFGC......PATVY......
125832698	......TLANM......IIQLL......VMVME......VFHRD......IDFGC......PATVY......
125832677	......TLANM......IIQLL......VMVME......VFHRD......IDFGC......PATVY......
125832647	......TLANM......IIQLL......VMVME......VFHRD......IDFGC......PATVY......
125832701	......TLANM......IIKLL......VMVME......VFHRD......IDFGC......PATVY......
125832681	......TLANM......IIKLL......VMVME......VFHRD......IDFGC......PATVY......
125832780	......TLANM......IIQLL......VMVME......VFHRD......IDFGC......TATVY......
125832692	......TLANM......IIQLL......VMVME......VFHRD......IDFGC......PATVY......
125832705	......TLANM......IIKLL......VMVME......VFHRD......IDFGC......PATVY......
125832784	......TLANM......IIKLL......VMVME......VFHRD......IDFGC......PATVY......
125832707	......TLANM......IIKLL......VMVME......VFHRD......IDFGC......PATVY......
125832786	......TLANM......IIQLL......VMVME......VFHRD......IDFGC......PATVY......
125832645	......TLANM......IIKLL......VMVME......VFHRD......IDFGC......PATVY......
125832703	......TLANM......IIKLL......VMVME......VFHRD......IDFGC......PATVY......
125832794	......TLANM......IIKLL......VMVME......VFHRD......IDFGC......PATVY......
125832688	......TLANM......IIQLL......VMVME......VFHRD......IDFGC......PATVY......
125833074	......ALMTR......VLQLL......VLILE......VLHRD......LDFGC......PATVW......
125833076	......ALMTR......VLQLL......VLILE......VLHRD......LDFGC......PATVW......
125834442	......ALMTR......VLQLL......VLILE......VLHRD......LDFGC......PATVW......
125834426	......ALMTR......VLQLL......VLILE......VLHRD......LDFGC......PATVW......
125834428	......ALMTR......VLQLL......VLILE......VLHRD......LDFGC......PATVW......
125834977	......ALHKS......IVELL......FMVLE......VLHRD......IDFGC......PATVW......
125835645	......ALMTR......VLQLL......ILILE......VLHRD......LDFGC......PATVW......
125835647	......ALMTR......VLQLL......ILILE......VLHRD......LDFGC......PATVW......
125835649	......ALMTR......VLQLL......ILILE......VLHRD......LDFGC......PATVW......
125835692	......ALMTR......VLQLL......ILILE......VLHRD......LDFGC......PATVW......
125835694	......ALMTR......VLQLL......ILILE......VLHRD......LDFGC......PATVW......
125835643	......ALMTR......VLQLL......ILILE......VLHRD......LDFGC......PATVW......
125836082	......ALHFL......IIELL......FMVLE......VLHRD......IDFGC......PATVW......
125836086	......ALHFL......IIELL......FMVLE......VLHRD......IDFGC......PATVW......
125836080	......ALHFL......IIELL......FMVLE......VLHRD......IDFGC......PATVW......
125836084	......ALHFL......IIELL......FMVLE......VLHRD......IDFGC......PATVW......
125838153	......ALTKG......LVQLL......FMVLE......VLHRD......TDFGS......SATVW......
125840546	......ALQIL......ILELL......LMVLE......VLHRD......IDFGC......PATVW......
125841417	......ALAIM......IIKLI......IIIME......VFHRD......IDFGC......PATVW......
125841428	......ALAIM......IIKLI......IIIME......VFHRD......IDFGC......PATVW......
125841430	......ALAIM......IIKLI......IIIME......VFHRD......IDFGC......PATVW......
125841425	......ALAIM......IIKLI......IIIME......VFHRD......IDFGC......PATVW......
125842438	......ALMTR......VLQLL......ILILE......VLHRD......LDFGC......PATVW......
125843048	......ALTIL......IIRML......IMILE......VLHRD......IDFGC......AATVW......
125843046	......ALTIL......IIQML......IMILE......VLHRD......IDFGC......AATVW......
125844916	......GLLIL......IIELL......IMILE......VFHRD......IDFGC......PSTVW......
125844914	......GLLIL......IIELL......IMILE......VFHRD......IDFGC......PSTVW......
125844918	......GLLIL......IIELL......IMILE......VFHRD......IDFGC......PSTVW......
125844938	......GLLIL......IIELL......IMILE......VFHRD......IDFGC......PSTVW......
125844955	......GLLIL......IIELL......IMILE......VFHRD......IDFGC......PSTVW......
125844928	......GLLIL......IIELL......IMILE......VFHRD......IDFGC......PSTVW......
125844949	......GLLIL......IIELL......IMILE......VFHRD......IDFGC......PSTVW......
125844940	......GLLIL......IIELL......IMILE......VFHRD......IDFGC......PSTVW......
125844936	......GLLIL......IIELL......IMILE......VFHRD......IDFGC......PSTVW......
125844920	......GLLIL......IIELL......IMILE......VFHRD......IDFGC......PSTIW......
125844926	......GLLIL......IIELL......IMILE......VFHRD......IDFGC......PSTVW......
125844932	......GLLIL......IIELL......IMILE......VFHRD......IDFGC......PSTVW......
125844942	......GLLIL......IIELL......IMILE......VFHRD......IDFGC......PSTVW......
125844922	......GLLIL......IIELL......IMILE......VFHRD......IDFGC......PSTVW......
125844953	......GLLIL......IIELL......IMILE......VFHRD......IDFGC......PSTVW......
125844946	......GLLIL......IIELL......IMILE......VFHRD......IDFGC......PSTVW......
125844930	......GLLIL......IIELL......IMILE......VFHRD......IDFGC......PSTVW......
125844912	......GLLIL......IIELL......IMILE......VFHRD......IDFGC......PSTVW......
125844924	......GLLIL......IIELL......IMILE......VFHRD......IDFGC......PSTVW......
125846739	......ALTKG......IVKLL......FLVLE......VLHRD......TDFGC......PATVW......
125846733	......ALTKG......LVQLL......FMVLE......VLHRD......IDFGC......PATVW......
125846745	......ALTKG......IVELL......FMVLE......VFHRD......IDFGC......PATVW......
125846828	......ALLAT......IAELL......IIVVE......VFHSD......IDFGS......PATVW......
125846817	......ALLAT......IAELL......IIVVE......VFHSD......IDFGS......PATVW......
125846859	......ALHFL......IIELL......FMVLE......VLHRD......IDFGC......PATVW......
125846887	......ALTKG......LVQLL......FMVLE......VFHRD......IDFGC......PATVW......
125846865	......ALLAT......IAELL......IIVVE......VFHSD......IDFGS......PATVW......
125846811	......ALLAT......IAELL......IIVVE......VFHSD......IDFGS......PATVW......
125846863	......ALLAT......IAELL......IIVVE......VFHSD......IDFGS......PATVW......
125846874	......ALHFL......VVQLL......LMVLE......VFHRD......IDFGC......PATVW......

```
125846819   ......ALLAT......IAELL......IIVVE......VFHSD......IDFGS......PATVW......
125846823   ......ALLAT......IAELL......IIVVE......VFHSD......IDFGS......PATVW......
125846871   ......ALTKG......LVQLL......FMVLE......VLHRD......IDFGC......PATVW......
125847123   ......ALSKG......IVKLL......LLVLE......VLHRD......TDFGC......PATVW......
125847125   ......ALSKG......IVKLL......LLVLE......VLHRD......TDFGC......PATVW......
125847127   ......ALSKG......IVKLL......LLVLE......VLHRD......TDFGC......PATVW......
125847234   ......ALLAT......IAELL......IIVVE......VFHSD......IDFGS......PATVW......
125847240   ......ALLAT......IAELL......IIVVE......VFHSD......IDFGS......PATVW......
125847412   ......TLANM......IIKLL......VMVME......VFHRD......IDFGC......PATVY......
125847392   ......TLANM......IIKLL......VMVME......VFHRD......IDFGC......PATVY......
125847394   ......TLANM......IIKLL......VMVME......VFHRD......IDFGC......PATVY......
125847821   ......ALTIL......IIRML......IMILE......VLHRD......IDFGC......AATVW......
125847837   ......ALTIL......IIRML......IMILE......VLHRD......IDFGC......AATVW......
125850745   ......ALTKG......IIKLL......VLILE......VFHRD......IDFGC......PATVF......
125850836   ......ALTKG......IIKLL......VLILE......VFHRD......IDFGC......PATVF......
125850966   ......ALLIL......IIQLL......IMVLE......VLHRD......IDFGC......PATVW......
125850956   ......ALLIL......IIQLL......IMVLE......VLHRD......IDFGC......PATVW......
125850958   ......ALLIL......IIQLL......IMVLE......VLHRD......IDFGC......PATVW......
125850952   ......ALLIL......IIQLL......IMVLE......VLHRD......IDFGC......PATVW......
125850960   ......ALLIL......IIQLL......IMVLE......VLHRD......IDFGC......PATVW......
125850962   ......ALLIL......IIQLL......IMVLE......VLHRD......IDFGC......PATVW......
125850964   ......ALLIL......IIQLL......IMVLE......VLHRD......IDFGC......PATVW......
125851012   ......ALLIL......IIQLL......IMVLE......VLHRD......IDFGC......PATVW......
125851010   ......ALLIL......IIQLL......IMVLE......VLHRD......IDFGC......PATVW......
125851105   ......ALLIL......IIQLL......IMVLE......VLHRD......IDFGC......PATVW......
125851016   ......ALLIL......IIQLL......IMVLE......VLHRD......IDFGC......PATVW......
125851014   ......ALLIL......IIQLL......IMVLE......VLHRD......IDFGC......PATVW......
125851008   ......ALLIL......IIQLL......IMVLE......VLHRD......IDFGC......PATVW......
125851103   ......ALLIL......IIQLL......IMVLE......VLHRD......IDFGC......PATVW......
125851006   ......ALLIL......IIQLL......IMVLE......VLHRD......IDFGC......PATVW......
125854225   ......ALMIR......VLQLL......ILILE......VLHRD......LDFGC......PATVW......
125854223   ......ALMIR......VLQLL......VLILE......VLHRD......LDFGC......PATVW......
126031488   ......VLLKK......VIRLL......VLILE......VLHRD......IDFGS......SAAVW......
126309941   ......LLLKK......VIRLL......ILVLE......VLHRD......IDFGS......SAAVW......
126338690   ......RLGVG......VFKLL......LLVME......VVHRD......IDFGS......SATVW......
126342853   ......ALLRK......VIQLL......VLVLE......VVHRD......IDFGS......PATVW......
126632529   ......GLLIL......IIELL......IMILE......VFHRD......IDFGC......PSTVW......
126632863   ......ALLIL......IIQLL......IMVLE......VLHRD......IDFGC......PATVW......
126632868   ......ALLIL......IIQLL......IMVLE......VLHRD......IDFGC......PATVW......
126632866   ......ALLIL......IIQLL......IMVLE......VLHRD......IDFGC......PATVW......
126632867   ......ALLIL......IIQLL......IMVLE......VLHRD......IDFGC......PATVW......
134133222   ......TLANM......IIKLL......VMVME......VFHRD......IDFGC......PATVY......
134104604   ......VLLKK......VIRLL......VLILE......VLHRD......IDFGS......SAAVW......
147904146   ......ALHFL......IIELL......FMVLE......VLHRD......IDFGC......PATVW......
148237667   ......ALTKG......IVKLL......FLVLE......VLHRD......TDFGC......PATVW......
146218597   ......VLLRK......VIRLL......LLVLE......VVHRD......IDFGS......SATVW......
148701982   ......ALLWK......VIRLL......MLVLE......VVHRD......IDFGS......PATVW......
148725441   ......ALHKS......IVELL......FMVLE......VLHRD......IDFGC......PATVW......
148725650   ......GLLIL......IIELL......IMILE......VFHRD......IDFGC......PSTVW......
148725649   ......GLLIL......IIELL......IMILE......VFHRD......IDFGC......PSTVW......
148725648   ......GLLIL......IIELL......IMILE......VFHRD......IDFGC......PSTVW......
149028435   ......ALLWK......VIRLL......MLVLE......VVHRD......IDFGS......PATVW......
149431651   ......LLLEK......VIHLL......CLVME......VLHRD......IDFGS......SAAVW......
149732159   ......VLLKK......VIRLL......VLILE......VLHRD......IDFGS......SAAVW......
149744622   ......ALLWK......VIRLL......MLVLE......VVHRD......IDFGS......PATVW......
107770479   ......AVLMG......VLCLK......HLVLE......IVHRD......ADFGL......AVDVW......
157116342   ......NIMKS......VIKMH......FMVLE......ITHRD......SDFGL......KVDIW......
109093721   ......EILKK......IIKIK......YIVLE......IIHRD......TDFGH......AVDCW......
109093717   ......EILKK......IIKIK......YIVLE......IIHRD......TDFGH......AVDCW......
109093707   ......EILKK......IIKIK......YIVLE......IIHRD......TDFGH......AVDCW......
109093723   ......EILKK......IIKIK......YIVLE......IIHRD......TDFGH......AVDCW......
109093709   ......EILKK......IIKIK......YIVLE......IIHRD......TDFGH......AVDCW......
110591043   ......EILKK......IIKIK......YIVLE......IIHRD......TDFGH......AVDCW......
110755537   ......KILKA......IIRME......YIVLE......ITHRD......SDFGL......QVDVW......
115433630   ......RVLD-......-----......--VTP......IVHRD......SDFGC......KVDMW......
115389048   ......GLLMG......LLCLK......YLVLE......IVHRD......GDFGL......AVDIW......
114685627   ......EILKK......IIKIK......YIVLE......IIHRD......TDFGH......AVDCW......
114685629   ......EILKK......IIKIK......YIVLE......IIHRD......TDFGH......AVDCW......
114685643   ......EILKK......IIKIK......YIVLE......IIHRD......TDFGH......AVDCW......
114685641   ......EILKK......IIKIK......YIVLE......IIHRD......TDFGH......AVDCW......
114685649   ......EILKK......IIKIK......YIVLE......IIHRD......TDFGH......AVDCW......
115898478   ......RILKR......IIGIE......YIVLE......ITHRD......TDFGL......AVDCW......
117616316   ......EILKK......IIKIK......YIVLE......IIHRD......TDFGQ......AVDCW......
```

121583180	......EILKK......LIKIK......YIVLE......IIHRD......TDFGQ......AVDCW......
121713286	......NILAR......IISVE......YLFQD......IVHRD......TDFGC......AVDLW......
121711199	......GLLMG......LLCLK......YLVLE......IVHRD......GDFGL......AVDIW......
119482536	......GLLMG......LLCLK......YLVLE......IVHRD......GDFGL......AVDIW......
119479723	......RILAR......IIRVE......YLFQD......IAHRD......TDFGC......AVDLW......
119580160	......EILKK......IIKIK......YIVLE......IIHRD......TDFGH......AVDCW......
119580161	......EILKK......IIKIK......YIVLE......IIHRD......TDFGH......AVDCW......
145496336	......KRLIK......IVKAY......YVILE......ICHRD......TDFNV......MVDMW......
145511818	......KRLIK......IVKVH......FQ---......ICHRD......TDFNV......MVDMW......
145535077	......QRLIK......IVKVH......YVVME......ICHRD......TDFNV......MVDMW......
126324935	......EILKK......IIKIK......FIVLE......IIHRD......TDFGQ......AVDCW......
145235283	......LILKD......-----......YLFQE......IIHRD......SDFGC......AADMW......
145247807	......GILMG......LLCLR......YLVLE......IIHRD......GDFGL......AVDVW......
145613492	......AVLMG......VLCLK......YLVLE......IVHRD......ADFGL......AVDVW......
146416461	......QILRK......IVALK......YLVME......ILHRD......TDFGL......LVDIW......
149238209	......DLLLS......IVQYR......YLVLE......IIHRD......ADFGL......KVDLW......
148688050	......EILKK......IIKIK......YIVLE......IIHRD......TDFGQ......AVDCW......
149063701	......EILKK......IIKIK......YIVLE......IIHRD......TDFGQ......AVDCW......
150863790	......NLLLS......IVRFI......YLVLE......IIHRD......ADFGL......KVDMW......
150864233	......SILRK......IVSLK......YIVME......ISHRD......TDFGL......LVDIW......
149634294	......EILKK......IVKIK......FIVLE......IIHRD......TDFGQ......SVDCW......
149720308	......EILKK......IIKIK......YIVLE......IIHRD......TDFGQ......AVDCW......
154278792	......AVLMS......VLCLK......YLVLE......IVHRD......IIYSS......AVDIW......
154323894	......AILMG......ILCLK......YLVLE......IVHRD......ADFGL......AVDIW......
157138526	......ELVRN......LIRFH......YIVMQ......VVHRD......IDFGF......QSDIW......
157116072	......KTMIA......IVSVY......CIFMD......FCHRD......SDFTF......KYDMW......
157108314	......SVLMK......IIKIH......FIFMD......IAHRD......ADFGF......MADVW......
157127747	......AILLR......IIRVQ......FIFMR......IAHRD......SDFGF......ASDMW......
109001139	......QIVRT......IIQVY......CLVME......VAHRD......TDFGF......KGDVW......
109083131	......QVMKV......LINFY......YIILE......IVHRD......SDFGF......LSDTW......
109083133	......QVMKV......LINFY......YIILE......IVHRD......SDFGF......LSDTW......
109126614	......SILRG......IVHVF......YIVME......LVHRD......TDFGF......KYDVW......
110756729	......EILTK......IIQVH......FIFMR......IAHRD......ADFGF......KADVW......
110758568	......DILVK......VVHVH......YIFMR......IAHRD......ADFGF......KADLW......
114555341	......QIVRT......IIQVY......CLVME......VAHRD......TDFGF......KGDVW......
114601164	......EILAM......IIKTY......YIVME......VVHRD......SDFSF......KVDIW......
114652335	......QVMKV......LINFY......YIILE......IVHRD......SDFGF......LSDTW......
114652329	......QVMKV......LINFY......YIILE......IVHRD......SDFGF......LSDTW......
114652333	......QVMKV......LINFY......YIILE......IVHRD......SDFGF......LSDTW......
114652331	......QVMKV......LINFY......YIILE......IVHRD......SDFGF......LSDTW......
114685065	......DILAT......IVKTY......YIIME......IVHRD......SDFGF......KVDIW......
115686274	......KIVRN......IIELL......FLIME......VYHRD......TDFGF......NADIW......
117676382	......LSLNT......IVQLY......YLVLE......IVHRD......TDFGF......QADLW......
117616832	......DILAT......IIKTY......YIVME......VVHRD......SDFGF......KVDIW......
117616854	......QVMKV......LINFY......YIILE......IVHRD......SDFGF......LSDTW......
118101772	......QIIER......IIHVY......YLVME......VAHRD......TDFSF......KGDIW......
119586474	......QVMKV......LINFY......YIILE......IVHRD......SDFGF......LSDTW......
119894461	......SILRG......IVHVF......YIVME......LVHRD......TDFGF......KYDVW......
119906182	......SSLNT......VVQLY......YLVLE......IVHRD......SDFGF......QADLW......
89001368	......QVMKV......LINFY......YIILE......IVHRD......SDFGF......LSDTW......
89001366	......QVMKV......LINFY......YIILE......IVHRD......SDFGF......LSDTW......
126323048	......YSLNT......VIQLY......YLVLE......IVHRD......TDFGF......QADLW......
126323434	......SILRG......IVHVF......YIVME......LVHRD......TDFGF......KYDVW......
126324866	......EILAM......IVKTY......YIVME......VVHRD......SDFGF......KVDIW......
126324868	......DILAT......IIKTY......YIVME......VVHRD......SDFGF......KVDIW......
126330235	......QIVRS......IIQVY......YLVME......VAHRD......TDFGF......KGDVW......
126347609	......VILAR......LVTAY......FLVMQ......VVHRD......TDFGI......PADVY......
149066103	......LSLNT......IVQLY......YLVLE......IVHRD......TDFGF......QADLW......
149063988	......QVMKV......LINFY......YIILE......IVHRD......SDFGF......LSDTW......
149433988	......YSLNT......VIQLY......YLVLE......IVHRD......TDFGF......RADLW......
149495278	......DILAT......IIKTY......YIVME......VVHRD......SDFGF......KVDIW......
149521808	......SILRG......IVHVF......YIVME......LVHRD......TDFGF......KYDVW......
149641931	......SVLRV......IVQVL......YIVME......VVHRD......TDFSF......KYDMW......
149694113	......QIVRT......IVQVY......YLVME......VAHRD......TDFGF......KGDVW......
149756130	......QVMKV......LINFY......YIILE......IVHRD......SDFGF......LSDTW......
149757622	......SSLNT......VVQLY......YLVLE......IVHRD......TDFGF......QADLW......
149758681	......EILAM......IVKTY......YIVME......VVHRD......SDFGF......KVDIW......
149758711	......DILAT......IIKTY......YIVME......IVHRD......SDFGF......KVDIW......
109488136	......EALKR......LAQLH......VLILE......ILHLD......IDLGN......QTDIW......
109488136	......DILAT......VTGLL......ILILE......ILHLD......CDFGF......GSDIW......

```
114573062   ......EALKG.....LAQLH.....VLILE.....ILHLD.....VDLGN.....QTDIW......
114573062   ......DILAA.....VTGLL.....ILILE.....VLHLD.....CDFGF.....ASDIW......
114588801   ......ALLQH.....YITLH.....ILILE.....VAHLD.....IDLED.....GTDIW......
114599040   ......GILQS.....LVGLL.....ILVLE.....IAHLD.....ADFGD.....TSDTW......
115739539   ......SMMQP.....ICSLH.....ILVLD.....IIHLD.....IDFGD.....GTDVW......
118086576   ......GVMQN.....LIGLI.....ILVLE.....IAHLD.....ADFGD.....TSDVW......
118093831   ......ALLQH.....YITIH.....ILVLE.....VAHLD.....IDLED.....STDIW......
97180266    ......EALKG.....LAQLH.....VLILE.....ILHLD.....VDLGN.....QTDIW......
97180266    ......DILAA.....VTGLL.....ILILE.....VLHLD.....CDFGF.....ASDIW......
119591159   ......EVLRT.....IMSLH.....VLIAE.....VLHLD.....VDFGS.....ATDIW......
119591159   ......RLLAR.....VLYFH.....VIVTE.....VLHLD.....CDFGN.....VTDIW......
119590276   ......EALKG.....LAQLH.....VLILE.....ILHLD.....VDLGN.....QTDIW......
119591158   ......EVLRT.....IMSLH.....VLIAE.....VLHLD.....VDFGS.....ATDIW......
119591158   ......RLLAR.....VLYFH.....VIVTE.....VLHLD.....CDFGN.....VTDIW......
119590275   ......EALKG.....LAQLH.....VLILE.....ILHLD.....VDLGN.....QTDIW......
119879610   ......ALLQH.....YITLH.....ILILE.....VAHLD.....IDLED.....GTDIW......
119888455   ......EVLRT.....LMALH.....VLIAE.....VLHLD.....VDFGS.....ATDIW......
119888455   ......RLLAR.....ILYFH.....VIVTE.....VLHLD.....CDFGN.....VTDIW......
119894437   ......EALKS.....LAQLQ.....VLILE.....ILHLD.....IDFGN.....QTDIW......
119894437   ......DILAG.....VTALL.....ILILE.....ILHLD.....CDFGF.....ASDIW......
122890189   ......DILAA.....VTGLL.....ILILE.....VLHLD.....CDFGF.....ASDIW......
122890189   ......EALKG.....LAQLH.....VLILE.....ILHLD.....VDLGN.....QTDIW......
160011671   ......ALLQH.....YVTLH.....ILILE.....VAHLD.....IDLED.....GTDIW......
123295462   ......EILKS.....IMALH.....VLITE.....ILHLD.....IDFGS.....PADIW......
123295462   ......ALLAE.....IVRFH.....IILTE.....IIHLD.....CDFGN.....STDIW......
125819783   ......ALLAE.....IVRFH.....IILTE.....IIHLD.....CDFGN.....STDIW......
125819783   ......EILKS.....IMALH.....VLITE.....ILHLD.....IDFGS.....PADIW......
125823608   ......DLLSR.....LACLL.....VLVVE.....ILHLD.....CDFGF.....ATDIW......
125823608   ......QVLRR.....IVQLH.....LLIEE.....IVHLD.....VDLGS.....ETDIW......
125826484   ......DILKS.....IMALH.....VLISE.....ILHLD.....IDFGS.....PADIW......
125826484   ......NILSH.....ILYFH.....IIITE.....ILHLD.....CDFGN.....ATDIW......
157074231   ......SVLQR.....LVRLL.....ALVLE.....IVHLD.....TDFGD.....SSDLW......
125864597   ......DILRH.....LVSLI.....MLILE.....VAHLD.....SDLGD.....STDVW......
126253818   ......GILQN.....LVSLL.....VLVLE.....IAHLD.....ADFGD.....TADTW......
126337770   ......GLLSR.....VIYFH.....VIVTE.....VLHLD.....CDFGN.....VTDVW......
126337770   ......EVLRS.....LMSLH.....VLIAE.....VLHLD.....VDFGS.....ATDIW......
148665444   ......ALLQH.....YVTLH.....ILILE.....VAHLD.....IDLED.....GTDIW......
148675740   ......EALKR.....LAQLH.....VLILE.....ILHLD.....IDLGN.....QTDIW......
148667988   ......EVLRT.....LMSLH.....VLIAE.....VLHLD.....VDFGS.....ATDIW......
148667988   ......RLLAR.....VLYFH.....VIVTE.....VLHLD.....CDFGN.....VTDIW......
148676947   ......GILQN.....LVSLL.....VLVLE.....IAHLD.....ADFGD.....TADTW......
148833506   ......EALKG.....LAQLH.....VLILE.....ILHLD.....VDLGN.....QTDIW......
148833506   ......DILAA.....VTGLL.....ILILE.....VLHLD.....CDFGF.....ASDIW......
148839466   ......ALLQH.....YITLH.....ILILE.....VAHLD.....IDLED.....GTDIW......
149052760   ......EALKR.....LAQLH.....VLILE.....ILHLD.....IDLGN.....QTDIW......
149060631   ......ALLQH.....YVTLH.....ILILE.....VAHLD.....IDLED.....GTDIW......
149266512   ......GILQN.....LVSLL.....VLVLE.....IAHLD.....ADFGD.....TADTW......
149267522   ......ALLQH.....YVTLH.....ILILE.....VAHLD.....IDLED.....GTDIW......
149267524   ......ALLQH.....YVTLH.....ILILE.....VAHLD.....IDLED.....GTDIW......
149508070   ......GVMQN.....LVGLL.....VLVLE.....IVHLD.....ADFGD.....TSDIW......
149567517   ......EVLKG.....IARLH.....VLVLE.....ILHLD.....LDFGN.....QTDVW......
151358127   ......DILAT.....VTGLL.....ILILE.....ILHLD.....CDFGF.....GSDIW......
151358127   ......EALKR.....LAQLH.....VLILE.....ILHLD.....IDLGN.....QTDIW......
151554487   ......EALKS.....LAQLQ.....VLILE.....ILHLD.....IDFGN.....QTDIW......
151554487   ......DILAG.....VTALL.....ILILE.....ILHLD.....CDFGF.....ASDIW......
156121071   ......GILQN.....LVGLL.....VLVLE.....IVHLD.....ADFGD.....TSDTW......
108705966   ......STMKL.....VVQLH.....YMVLE.....VYHRD.....SDFGL.....AADVW......
115452923   ......SVMNL.....IVQLY.....YFILE.....VYHRD.....SDFGL.....KADVW......
108707855   ......CTMKL.....VVRLF.....FIVLE.....VYHRD.....SDFGL.....AADIW......
108707856   ......CTMKL.....VVRLF.....FIVLE.....VYHRD.....SDFGL.....AADIW......
115454909   ......SIMKM.....IVNLI.....YMVLE.....IIHGD.....GDFSV.....AADTW......
108707591   ......KILRL.....IIRLY.....YVVME.....VVHRD.....ADFGL.....EVDVW......
108803785   ......RSAAS.....IVAIY.....YIAME.....IVHRD.....ADFGI.....RSDLY......
108803574   ......RTAAR.....VVQVY.....YIVME.....VVHRD.....SDFGI.....ESDVY......
161611313   ......KIAAD.....VVPVF.....YYTMP.....ILHRD.....LDWGF.....STDIY......
115487184   ......STMKL.....VVRIY.....YIVLE.....VYHRD.....SDFGL.....MADLW......
108862929   ......AAMRR.....VLRLH.....YLVME.....VAHRD.....SDFGL.....KADAW......
157133027   ......QIMSS.....IIHIY.....VLVME.....ICHRD.....ADFGL.....EVDCW......
157133029   ......QIMSS.....IIHIY.....VLVME.....ICHRD.....ADFGL.....EVDCW......
157117199   ......STLEA.....ILRLF.....YLVSE.....YVHRD.....ADFGF.....PVDIW......
157115362   ......QNLKL.....IIKLY.....FMIME.....IVHRD.....ADFGL.....EVDIW......
```

157111789	......KLLRK......VIELL......YLIME......IVHKD......SDFGV......KVDIW......
157103753	......RIMKL......IVKLF......YLVME......IIHRD......ADFGF......EVDVW......
157103491	......TICHM......IVELL......YMVFE......IIHRD......GGFGS......ACDVW......
157138605	......RCMKL......VVRLY......YLILE......VVHRD......TDFGF......AVDVW......
157133960	......SILKV......ITRLY......YLVTE......IVHRD......ADFGF......KSDIW......
157114788	......AIMKL......VLGLT......YLVLE......ICHRD......ADFGM......RADVW......
109018728	......EIMSS......IIAIH......VIVME......VVHRD......ADFGL......EVDSW......
109041214	......RCMKL......IVRLY......YLILE......VVHRD......TDFGF......AVDIW......
109077138	......SSMEK......IIRLY......HLVME......IIHRD......GDFGF......YVDIW......
109077066	......QNLKL......IIKLY......FMVME......VVHRD......ADFGL......EVDIW......
109077068	......QNLKL......IIKLY......FMVME......VVHRD......ADFGL......EVDIW......
109087449	......QLPS-......ITGIV......YVFFE......VVLGD......EDDAL......AADVW......
109087447	......QLPS-......ITGIV......YVFFE......VVLGD......EDTHI......AADVW......
109092519	......---RL......VAQPT......YAFFT......LVLRD......VDACV......AADVW......
109098543	......EIMSS......IISIY......VIIME......VVHRD......ADFGL......EVDSW......
109102098	......FCLSA......INQIT......YVFFE......LVLRD......EDAYI......AADVW......
109105637	......RIMKV......IVKLF......YLVME......IVHRD......ADFGF......EVDVW......
109105625	......RIMKV......IVKLF......YLVME......IVHRD......ADFGF......EVDVW......
109105627	......RIMKV......IVKLF......YLVME......IVHRD......ADFGF......EVDVW......
109105633	......RIMKV......IVKLF......YLVME......IVHRD......ADFGF......EVDVW......
109105635	......RIMKV......IVKLF......YLVME......IVHRD......ADFGF......EVDVW......
109105631	......RIMKV......IVKLF......YLVME......IVHRD......ADFGF......EVDVW......
109105629	......RIMKV......IVKLF......YLVME......IVHRD......ADFGF......EVDVW......
109105639	......RIMKV......IVKLF......YLVME......IVHRD......ADFGF......EVDVW......
109105623	......RIMKV......IVKLF......YLVME......IVHRD......ADFGF......EVDVW......
109107378	......NILKS......IIHLE......YLVME......IVHRD......TDFGL......QCDIW......
109109165	......CINKM......VVKFY......YLFLE......ITHRD......SDFGL......PVDVW......
109109167	......CINKM......VVKFY......YLFLE......ITHRD......SDFGL......PVDVW......
109110965	......EALKN......ICQLY......FMVLE......YAHRD......IDFGL......EADVW......
109122721	......QLLRR......VIQLV......YMVME......IVHKD......SDLGV......KVDIW......
109125166	......RIMKG......IVKLF......YLVME......IVHRD......ADFGF......EVDIW......
109457814	......NIMRK......IVSLI......YLIME......IVHRD......IDFGL......KNDMW......
109457827	......NIMRK......IVSLL......YLIME......IVHRD......IDFGL......KNDMW......
109457801	......NIMRK......IVSLL......YLIME......IVHRD......IDFGL......KNDMW......
109457874	......NIMRK......IVSLI......YLIME......IVHRD......IDFGL......KNDMW......
109457823	......NIMRK......IVSLL......YLIME......IVHRD......IDFGL......KNDMW......
109458305	......RIMKG......IVKLF......YLVME......IVHRD......ADFGF......EVDIW......
109457859	......NIMRK......IVSLI......YLIME......IVHRD......IDFGL......KNDMW......
109483102	......DIMMS......IISLF......YLIME......IVHRD......IDFGL......KVDVW......
109460327	......NIMRK......IVSLI......YLIME......IVHRD......IDFGL......KNDMW......
109457598	......EIMKI......IVSLL......YIIME......IIHRD......IDFGL......KVDIW......
109457791	......EIMMR......IVSLI......YLIME......IIHRD......VDFGS......KVDVW......
109457882	......NIMRK......IVSLI......YLIME......IVHQD......IDFGL......KNDMW......
109457816	......NIMRK......IVSLI......YLIME......IVHRD......IDFGL......KNDMW......
109457793	......EIMMR......IVSLI......YLIME......IIHRD......IDFGS......KVDVW......
109457783	......EIMMR......IVSLI......YLIME......IIHRD......VDFGS......KVDVW......
109457789	......EIMMR......IVSLI......YLIME......IIHRD......IDFGS......KVDVW......
109457857	......NIMRK......IVSLI......YLIME......IVHRD......IDFGL......KNDMW......
109457795	......EIMMR......IVSLI......YLIME......IIHRD......VDFGS......KVDVW......
109457852	......NIMRK......IVSLI......YLIME......IVHRD......IDFGL......KNDMW......
109457810	......NIMRK......IVSLL......YLIME......IVHRD......IDFGL......KNDMW......
109457812	......NIMRK......IVSLI......YLIME......IVHRD......IDFGL......KNDMW......
109457848	......NIMRK......IVSLL......YLIKE......IVHRD......IDFQL......KNDMW......
109457872	......NIMRK......IVSLL......---ME......IVHRD......IDFGL......KNDMW......
109457846	......NIMRK......IVSLL......YLIME......IVHRD......IDFGL......KNDMW......
109457884	......NIMRK......IVSLI......YLIME......IVHRD......IDFGL......KNDMW......
109457878	......NIMRK......IVSLI......YLIME......IVHRD......IDFGL......KNDMW......
109457840	......NIMRK......IVSLI......YLIME......IVHRD......IDFGL......KNDMW......
109457820	......NIMRK......IVSLI......YLIME......IVHRD......IDFGL......KNDMW......
109458129	......AILKL......VLKLH......YLVLE......ICHRD......ADFGM......RADMW......
109459630	......AILKL......VLKLH......YLVLE......ICHRD......ADFGM......KADVW......
109460329	......NIMRK......IVSLL......YLIME......IVHRD......IDFGL......KNDMW......
109460325	......NIMRK......IVSLI......YLIME......IVHRD......IDFGL......KNDMW......
109460836	......NIMRK......IVSLI......YLIME......IVHRD......IDFGL......KNDMW......
109460843	......NIMRK......IVSLI......YLIME......IVHRD......IDFGL......KNDMW......
109461187	......AILKL......VLKLH......YLVLE......ICHRD......ADFGM......RADMW......
109463218	......AILKL......VLKLH......YLVLE......ICHRD......ADFGM......KADVW......
109468993	......DLMRS......IIQLL......FLVME......IAHRD......ADFGL......IVDMW......
109468984	......DLMTS......IISLL......FLVME......IAHRD......SDFGL......PADIW......
109471001	......DLMTS......VIRLL......FLVME......IAHRD......SDFGL......PADIW......
157820995	......QLLRR......VIQLV......YMVME......IVHKD......SDLGV......KVDIW......
109480356	......AILET......IIRLF......NFVLE......IVHRD......TDFGL......KTDVW......
109480362	......TILES......IISLF......HFILQ......IVHRD......IDFGV......KADVW......
109480383	......TTLES......IISLY......HFILQ......IVHRD......IDFGQ......SSDVW......

```
109481721    ......AILET......IIRLF.....NFVLE.....IVHRD.....TDFGL.....KTDVW.....
109484648    ......QIMKM......IIRLY.....YLVTE.....IVHRD.....ADFGF.....KVDIW.....
109484684    ......QIMKM......IIKLY.....YLVTE.....VVHRD.....ADFGF.....QLDIW.....
109486445    ......SILKM......IICLY.....HLVTE.....IVHRD.....IDFGL.....KSDVW.....
109486442    ......SILKM......IICLY.....HLVTE.....IVHRD.....IDFGL.....KSDVW.....
109487715    ......GILKK......IISLH.....FLILE.....IVHRD.....VDFGL.....KVDTW.....
109492909    ......QIQQM......ITQLL.....YLVME.....VVHRD.....IDFGL.....KIDVW.....
109494007    ......QIQQM......ITQLL.....YLVME.....VVHRD.....IDFGL.....KIDVW.....
109503491    ......EIMKS......IIKLL.....FIVLE.....IVHRD.....SDFGL.....AIDVW.....
109504351    ......EIMKS......IIKLL.....FIVLE.....IVHRD.....SDFGL.....AIDVW.....
109504729    ......EIMKL......ITKLF.....CMVME.....IAHRD.....CDFGL.....RADIW.....
109504735    ......DIIKS......IIKLF.....YMVME.....IAHRD.....CDFGL.....QADIW.....
109505139    ......DILKS......IIKVV.....HLVME.....IVHRD.....CDFGL.....PFDVW.....
109505109    ......DILKS......IIKVV.....HLVME.....IIHRD.....CDFGL.....PFDVW.....
109505130    ......DILKS......IIKVV.....HLVME.....IVHRD.....CDFGL.....PFDVW.....
109505115    ......DILKS......IIKVV.....HLVME.....IVHRD.....CDFGL.....PFDVW.....
109505134    ......DILKS......IIKVV.....HLVME.....IVHRD.....CDFGL.....PFDVW.....
109505132    ......DILKS......IIKVV.....HLVME.....IVHRD.....CDFGL.....PFDVW.....
109505123    ......DILKS......IIKVV.....HLVME.....IVHRD.....CDFGL.....PFDVW.....
109505128    ......DILKS......IIKVV.....HLVME.....IVHRD.....CDFGL.....PFDVW.....
109505119    ......DILKS......IIKVV.....HLVME.....IVHRD.....CDFGL.....PFDVW.....
109505319    ......DILKS......IIKVV.....HLVME.....IVHRD.....CDFGL.....PFDVW.....
109505321    ......DILKS......IIKVV.....HLVME.....IVHRD.....CDFGL.....PFDVW.....
109505331    ......DILKS......IIKVV.....HLVME.....IVHRD.....CDFGL.....PFDVW.....
109505331    ......DILKS......ITKVV.....HLVME.....IVHRD.....CDFGL.....PFDVW.....
109505543    ......DIIKS......IIKLF.....YMVME.....IAHRD.....CDFGL.....QADIW.....
109506268    ......DILKS......IIKVV.....HLVME.....IVHRD.....CDFGL.....PFDVW.....
109518360    ......TTLES......IISLY.....HFILQ.....IVHRD.....IDFGQ.....SSDVW.....
109519716    ......ELLQS......IVRFF.....YIIME.....IAHRD.....CDFGM.....AGDMW.....
109513165    ......NIMRK......IVSFL.....YLIME.....IFHRD.....IDFQL.....KNDMW.....
109514426    ......DILKS......IIKVV.....HLVME.....IVHRD.....CDFGL.....PFDVW.....
109730639    ......RIMKI......IVKLF.....YLVME.....IVHRD.....ADFGF.....EVDVW.....
94482160     ......TVLRA......VVQLK.....CLILP.....IIHRD.....GDFGA.....AVDIW.....
126179714    ......RFWED......IVTVH.....FVEME.....VIHRD.....TDWGM.....ATDIY.....
110590744    ......CINAM......VVKFY.....YLFLE.....ITHRD.....SDFGL.....PVDVW.....
110590725    ......CINKM......VVKFY.....YLFLE.....ITHRD.....SDFGL.....PVDVW.....
110590560    ......QNLKL......IIKLY.....FMVME.....VVHRD.....ADFGL.....EVDIW.....
110737680    ......SAMRL......IVELH.....YFVME.....VCHRD.....SDFGL.....KADVW.....
110741211    ......EILQC......LPTLY.....CLVME.....IIYRD.....SDFDL.....AVDWW.....
110743751    ......INHRS......IIRFK.....AIVME.....ICHRD.....CDFGY.....HADVW.....
110749474    ......RIMKM......IVKLF.....YLVME.....IIHRD.....ADFGF.....EVDVW.....
110759306    ......TICHM......IVELL.....YMVFE.....IIHRD.....RGFSV.....PGDVW.....
110761573    ......HIMKR......IIRLY.....YLVTE.....VVHRD.....ADFGF.....RADVW.....
110763851    ......QIMSS......IIHIY.....VLVME.....ICHRD.....ADFGL.....EVDCW.....
111020177    ......QLLAS......VVPVY.....LLVME.....ILHRD.....TDFGI.....ATDVY.....
111023174    ......RNAAK......SIAMY.....WLVME.....IVHRD.....SDFGI.....ASDVF.....
111023678    ......QTLAQ......IVRIF.....LLYMQ.....VLHRD.....ADFNV.....PADIF.....
111020362    ......RAAGR......VVNVL.....YIVMP.....ILHRD.....SDFGI.....AADVY.....
111017776    ......RAMGR......IVNIL.....YIVMP.....ILHRD.....TDFGI.....AADVY.....
111019707    ......RAMGR......IVSAL.....YIVMP.....VLHRD.....TDFGI.....ASDIY.....
111020359    ......RAAGR......IVNVF.....FIVMP.....ILHRD.....TDFGI.....AADVY.....
111017717    ......RLAAT......VVAVH.....YIVME.....ILHRD.....TDFGI.....TDDLY.....
111020043    ......QTLAQ......IVRVY.....VLFME.....VLHRD.....ADFNV.....RSDIF.....
111026426    ......RVMGQ......IVSAL.....FIVMP.....IVHRD.....TDFGI.....AADIY.....
111025634    ......RAMGR......IVNVL.....FIVMP.....ILHRD.....TDFGI.....AADIY.....
111026453    ......RAMGR......IMSVL.....YLVMP.....IVHRD.....ADFGI.....ASDVY.....
111026885    ......RAAGR......VVNVL.....YIVMP.....ILHRD.....TDFGI.....AADVY.....
121610423    ......QILPV......VPRFV.....YLVME.....VCHLD.....LDFGL.....RSDIF.....
110665974    ......INHRS......IIRFK.....AIVME.....ICHRD.....CDFGY.....LADVW.....
169613745    ......EIWRF......ILPLL.....FCITK.....VVHRD.....CDFGM.....AADIW.....
169616360    ......QILRQ......IVRLH.....GIILE.....IVHRD.....TDFGF.....KVDVW.....
169604334    ......ILHSH......IIHCL.....WIAME.....VAHRD.....SDFGL.....NIDIW.....
169602253    ......RIMES......IVEFV.....YIIME.....ITHRD.....SDFGL.....SVDIW.....
111221034    ......ETARR......TAEVL.....YLVTE.....IVHRD.....IDFGI.....AVDIW.....
111220653    ......RAAGR......AVAVL.....WIVME.....IVHRD.....TDFGI.....TGDRW.....
111223814    ......AILIR......VVAIH.....AIVME.....IVHRD.....SDFGV.....AADVY.....
111219919    ......QMLGR......IVRIH.....LLVME.....IAHRD.....TDFGI.....ATDVY.....
111222708    ......RAAAR......LVTVY.....WIVME.....VVHRD.....TDFGI.....AGDVW.....
111224062    ......RVLAG......VVKAY.....LVVME.....VLHRD.....TDFGI.....ATDLY.....
111223990    ......RVLAG......VVRAF.....LVVME.....VLHRD.....GDFGI.....ATDLY.....
111220793    ......RLLER......IVRVH.....MIIME.....VIHRD.....SDFGI.....ATDLY.....
111223723    ......RLLAG......IVRVF.....LIVME.....VLHLD.....ADFGI.....ATDLY.....
111219555    ......RVLAE......IVRIH.....LLVME.....VLHRD.....TDFGI.....STDLY.....
```

```
111219920   ......RLLGR......IVQIY......LLVME......VLHRD......TDFGI......ATDLY......
111219763   ......RITAR......VPAVY......YLVMQ......LVHRD......LDFGL......ATDLY......
111224871   ......RIVAS......IVRVF......LIVME......LLHRD......TDFGI......AADVY......
111225188   ......DLLVD......LVAVR......AIVMD......IVHRD......TDFGI......AADVY......
111220490   ......AAARR......TAQVL......YLVTE......IVHRD......IDFGI......AVDVF......
111224669   ......SVLTR......VVKVW......AIVMD......IVHRD......TDFGI......PADVY......
111222721   ......RILAR......IVRVY......LIVME......VLHRD......ADFGI......ATDLY......
111219921   ......RLLSR......IAQVY......LLVME......VLHRD......TDFGI......ATDLY......
111220319   ......AAVSS......VQTLG......YEISP......LVHRD......GDYGI......ARDYW......
111220652   ......RALAR......IVSVY......WIIME......VLHRD......TDFGI......ASDLW......
115495687   ......DILKS......IIHLE......YLVME......IVHRD......TDFGL......QCDIW......
112491250   ......RIMKI......IVKLF......YLVME......IVHRD......ADFGF......EVDVW......
113951717   ......QLLRR......VIQLV......YMVME......IVHKD......SDLGV......KVDIW......
114679850   ......HLMK-......FVNLF......TLILD......IIHND......CDYGL......SMDWW......
115447397   ......KLLAA......VVQVH......HMVMD......VAHRD......ADFGS......KADVW......
115465057   ......KILRL......IIRLY......YVVME......VVHRD......ADFGL......EVDVW......
115473597   ......ATLKL......VVRLH......YMVLE......VYHRD......SDFGL......LSDIW......
115474071   ......AILPR......VMRLR......HFVLD......VYHRD......ADFGL......KVDIW......
115479185   ......SIMRM......VVGIR......FVVME......VAHRD......TDFGL......RADLW......
115483484   ......INHRS......IIRFK......AIVME......ICHRD......CDFGY......ATDVW......
115489454   ......AAMRR......VLRLH......YLVME......VAHRD......SDFGL......KADAW......
159899587   ......QLLAR......MTKVT......FLVME......IIHRD......IDLGI......RSDLY......
159899682   ......EITSK......IIEIY......YTVMA......IIHRD......CDFGI......QSDIF......
145595768   ......KTIAR......VVAVY......YLVME......LVHRD......ADFGL......RTDVY......
159898976   ......ELLAA......IPEIY......-LIMK......VFHRD......IDFGL......KSDIY......
145596853   ......RAAGR......VVDLF......YLIME......VLHRD......ADFGL......AVDVY......
145594644   ......RIMAA......VVQVF......YLIME......VVHRD......VDFGV......ATDIY......
159896556   ......QAIAQ......IVRLY......YMVME......VIHRD......ADFGL......QTDLY......
115438410   ......CISSS......VIHTL......CEVME......VAHRD......TDFGN......AVDIW......
115400970   ......HHHRQ......IARLY......WLVLE......CVHRD......CDFGF......KVDVW......
115438194   ......YCHKR......IVNLI......YLVLE......LYHRD......GDFGL......KADLW......
115386730   ......VIMKL......VISLY......YLVLE......ICHRD......ADFGM......KADIW......
115442844   ......HVLDY......IVEME......YIEMH......VVHRD......IDFGS......EQDIW......
115433412   ......QYLQL......IIKLY......VMVLE......IVHRD......ADFGL......EVDVW......
115390046   ......TVHKH......IISFF......WIAME......VGHRD......ADFGL......DVDIW......
115396244   ......KLHHL......VVSLV......YVVIE......IYHRD......ADFGL......PNDVW......
115491699   ......RVWRY......LLTLD......FCFTK......VVHRD......CDFGM......SVDIW......
115492651   ......SILRD......IVRLH......GIIME......IVHRD......TDFGF......KVDVW......
115491531   ......AIVSL......ICGMR......YMLFE......IVHRD......IDFGL......EVDVW......
87312440    ......MNHRS......IIRFK......AIVME......VCHRD......CDFGY......KVDVW......
87312444    ......MNHRS......IIRFK......AIVME......VCHRD......CDFGY......KVDVW......
114763648   ......RAAGR......LVTVF......YLVME......IVHRD......TDFGI......RADIY......
114572749   ......RIMKI......IVKLF......YLVME......IVHRD......ADFGF......EVDVW......
114572761   ......RIMKI......IVKLF......YLVME......IVHRD......ADFGF......EVDVW......
114572753   ......RIMKI......IVKLF......YLVME......IVHRD......ADFGF......EVDVW......
114572757   ......RIMKI......IVKLF......YLVME......IVHRD......ADFGF......EVDVW......
114572751   ......RIMKI......IVKLF......YLVME......IVHRD......ADFGF......EVDVW......
114572755   ......RIMKI......IVKLF......YLVME......IVHRD......ADFGF......EVDVW......
114572747   ......RIMKI......IVKLF......YLVME......IVHRD......ADFGF......EVDVW......
114572759   ......RIMKI......IVKLF......YLVME......IVHRD......ADFGF......EVDVW......
114576171   ......FCLSA......INQIT......YVFFE......LVLRD......EDAYI......AADVW......
114584219   ......AILSR......IIKVL......QLVME......IIHRD......IDFGS......ELEMW......
114584213   ......AILSR......IIKVL......QLVME......IIHRD......IDFGS......ELEMW......
114584209   ......AILSR......IIKVL......QLVME......IIHRD......IDFGS......ELEMW......
114584211   ......AILSR......IIKVL......QLVME......IIHRD......IDFGS......ELEMW......
114584221   ......AILSR......IIKVL......QLVME......IIHRD......IDFGS......ELEMW......
114586395   ......RCMKL......IVRLY......YLILE......VVHRD......TDFGF......AVDIW......
114600411   ......SSMEK......IIRLY......HLVME......IIHRD......GDFGF......YVDIW......
114621649   ......QLPS-......ITGIV......YVFFE......IVLGD......EDTHI......AADVW......
114624496   ......EALKN......ICQLY......FMVLE......YAHRD......IDFGL......EADVW......
114624504   ......EALKN......ICQLY......FMVLE......YAHRD......IDFGL......EADVW......
114624506   ......EALKN......ICQLY......FMVLE......YAHRD......IDFGL......EADVW......
114640293   ......QIMKM......IIKLY......YLVTE......IVHRD......ADFGF......QLDIW......
114640690   ......QIMKM......IIRLY......YLVTE......IVHRD......ADFGF......KVDIW......
114641089   ......CINKM......VVKFY......YLFLE......ITHRD......SDFGL......PVDVW......
114641077   ......CINKM......VVKFY......YLFLE......ITHRD......SDFGL......PVDVW......
114646685   ......EIMSS......IISIY......VIIME......VVHRD......ADFGL......EVDSW......
114654983   ......RIMKI......IVKLF......YLIME......IVHRD......ADFGF......EVDVW......
114654993   ......RIMKI......IVKLF......YLIME......IVHRD......ADFGF......EVDVW......
114654973   ......RIMKI......IVKLF......YLIME......IVHRD......ADFGF......EVDVW......
114654989   ......RIMKI......I----......-----......IVHRD......ADFGF......EVDVW......
114654987   ......RIMKI......IVKLF......YLIME......IVHRD......ADFGF......EVDVW......
```

```
114654977    ......RIMKI......IVKLF......YLIME......IVHRD......ADFGF......EVDVW......
114654975    ......RIMKI......IVKLF......YLIME......IVHRD......ADFGF......EVDVW......
114654981    ......RIMKI......IVKLF......YLIME......IVHRD......ADFGF......EVDVW......
114654971    ......RIMKI......IVKLF......YLIME......IVHRD......ADFGF......EVDVW......
114654979    ......RIMKI......IVKLF......YLIME......IVHRD......ADFGF......EVDVW......
114654995    ......RIMKI......IVKLF......YLIME......IVHRD......ADFGF......EVDVW......
114654991    ......RIMKI......IVKLF......YLIME......IVHRD......ADFGF......EVDVW......
114674399    ......QLLRR......VIQLV......YMVME......IVHKD......SDLGV......KVDIW......
114677740    ......RIMKG......IVKLF......YLVME......IVHRD......ADFGF......EVDIW......
114684554    ......QLMKL......IIKLY......YIVTE......IVHRD......ADFGF......QLDIW......
114798837    ......QILAA......IARLY......YMIME......IIHRD......IDFGI......LSDVY......
114798139    ......ITTRD......IVAVY......FVSME......VIHRD......LDFGI......AADIY......
114798457    ......QFLAR......IARLL......WMVME......IVHRD......IDFGV......ASDVY......
115359734    ......SLCEA......IVALL......FAVFE......IVHRD......LDFGV......ASDLY......
115379559    ......RAVAR......ILEVF......YIVTE......VIHRD......MDFGI......EADVF......
115380088    ......TALAA......IIQIF......YFVME......IIHRD......ADFGL......RADLF......
115379360    ......DHVRV......IARVL......FLVVE......VLHRD......MDFGL......ATDLY......
115379821    ......EIASQ......IIEVF......YLVME......VVHRD......LDFGI......RTDLF......
115375777    ......LALAA......IVTLY......FMVLE......IIHRD......MDFGI......RTDIF......
115375403    ......RTRVG......LLQLH......FFTME......LVHRD......LDFGL......AADLY......
115377625    ......RLARR......VCRVF......FLTME......VIHRD......TDFGL......ASDLY......
115377459    ......ELLSR......VPRLY......YVVME......GVHRD......MDFGS......ADDVY......
115376121    ......RTLTR......VVSLF......YLVME......IVHRD......LDFGI......RADLY......
115375998    ......EVLYT......VPTLY......FLAME......FVHCD......FDFGL......RTDVY......
115375733    ......ELLSR......VPRLL......YLVME......GVHRD......TDLGS......ADDVY......
115376448    ......ACLQQ......XIRMW......YIVLD......XXHRD......IDFGA......EADIF......
115376976    ......ALLCR......VPRLF......YLVME......AVHRD......MDYGA......ADDVF......
115377265    ......KLLQR......VVKLR......YLVME......AVHRD......MDLGA......ADDLY......
115375548    ......RISAT......IAQVY......FLAME......LVHRD......VDFGI......LTDVW......
115375859    ......ELLSR......VPRLI......YLTMQ......GVHRD......TDFGS......SDDLF......
115378978    ......-----......VVPLH......YFVRD......VWHRD......LD--L......ADDLY......
115375214    ......QVCAQ......IVRLI......FTVFE......VIHRD......LDFGL......NSDLY......
115378800    ......ELLSR......VPRLH......FLVMD......GIHRD......VDFGS......ADDLY......
115375200    ......EVLSR......IPRLF......YLVMD......CLHRD......LDFGC......HDDVY......
115378155    ......RANAR......VVQTF......YILME......VVHRD......SDFGI......RNDVF......
115373058    ......ELLSR......VPRLH......YLVMD......GVHRD......MDFGS......ADDLY......
115372252    ......ELLSR......VPRLQ......FIVMD......GLHRD......IDFGS......ADDVY......
115371836    ......ELLSR......IPRLR......FLVMD......CLHRD......TDFGS......ADDLY......
115374624    ......SILLR......VIRGF......YIAMD......VVHRD......VDYGI......SDDVW......
115371960    ......RAVNA......IIDIF......YIVME......VVHRD......LDFGL......MTDLY......
115373288    ......HVASR......VPRIL......SLLME......VVHRD......LDFGM......RMDVY......
115374848    ......ELLSR......VPRLV......YVVMQ......GLHRD......LDFGC......ADDVY......
115374374    ......ALLSR......VLQAH......FLVMQ......GVHRD......VDFGS......ADDVF......
115372742    ......KVGKK......MLRVY......FFTTQ......-PHSD......TDYGL......RMDIY......
115373983    ......RLLLQ......VVRVV......YVVLE......VLHRD......ADFGA......PTDDW......
115372135    ......ELLAR......VPRLL......YLVME......GVHRD......MDFGS......RVDVY......
115372649    ......ELLSR......VPCLR......FLVMD......GVHRD......IDFGA......ADDIY......
115372560    ......AALAT......IVDIV......YLVME......VIHRD......TDFGL......RADIF......
115373474    ......RVVVH......IARVY......YLAME......LVHRD......IDFGA......RADVY......
115373296    ......GLLSR......VPRLL......FLVME......GVHRD......VDFGA......ADDVF......
115373051    ......ELLSR......VPRLW......YLVMD......IVHRD......MDFGS......ADDLY......
115373603    ......RSLSD......LVTLH......FLTME......KLHRD......LDFGL......ASDWY......
115374086    ......ELLSR......VPRLY......YLVME......GVHRD......MDFGS......ADDVY......
115374073    ......EVLSR......VPRLL......YLVMD......CLHRD......VDFGC......EDDVY......
115372507    ......ELLSR......IPRLI......FLAMQ......AIHRD......TDFGS......YVDLF......
115502239    ......QIMKM......IIRLY......YLVTE......IVHRD......ADFGF......KVDIW......
115623693    ......SCMER......IIRLY......HLVME......IIHRD......GDFGF......PVDTW......
115673148    ......SCMER......IIRLY......HLVME......IIHRD......GDFGF......PVDTW......
115728513    ......KAMKE......ICTLY......FMVIE......YAHRD......IDFGL......EADIW......
115754817    ......KIMKL......IVKLF......YLAME......VVHRD......ADFGF......EVDVW......
115916143    ......KVMKL......IIRLY......YLVTE......IVHRD......ADFGF......KADVW......
115927496    ......GLLQL......IVRLL......YLVLE......VVHRD......IDFGL......KVDVW......
116000520    ......TNHRN......IVKFK......AVAME......ISHRD......CDFGF......ASDVW......
116000772    ......DVLQV......FPEKL......HIVTD......VAHRD......IDFGS......KCDVW......
116000407    ......VMMQQ......IVTIR......HVVMD......VVHRD......VDFGL......GVDVW......
116056476    ......VNHRG......VVGFI......AIVLE......VAHRD......CDFGY......TADVW......
116056342    ......ELQSL......VVEAY......RLVME......VVHYD......GDFGC......KADVW......
116059842    ......AIHRH......VTYLL......YTASE......VTHND......ADLGL......KVDVW......
116060774    ......AVLKK......IRTLH......ILVLE......IAHMD......ADFGV......RADIW......
116061023    ......KALAD......IVDLI......FMVMT......IYHRD......GDFGL......PADVW......
116060001    ......ELLSK......VVKIV......QVVME......VVFRD......VDFGR......AADMW......
116089335    ......AILKL......VLKLH......YLVLE......ICHRD......ADFGM......KADVW......
118788016    ......SILKV......ITRLY......YLVTE......IVHRD......ADFGF......KSDIW......
```

118788018	......SILKV......ITRLY......YLVTE......IVHRD......ADFGF......KSDIW......
119112475	......STLEC......ILRLF......HLISE......FVHRD......ADFGF......PVDIW......
118782399	......QNLKL......IIKLY......FMIME......---CD......ADFGL......EVDIW......
116621582	......RSIAA......ICTVH......YLVME......LIHRD......LDFGL......RADIW......
116626212	......QLARK......VCKVF......FLTME......VIHRD......TDFGL......RSDIY......
116626655	......RILAA......IARLL......YLVME......IVHRD......LDFGI......ATDIY......
116620747	......RILAA......IARLL......YLVME......IVHRD......LDFGI......ATDIY......
116241272	......AILKL......VLKLH......YLVLE......ICHRD......ADFGM......KADVW......
116284192	......TIMKN......IVKLF......FLVME......IVHRD......ADFGF......EVDVW......
116265972	......SVMRL......IVELK......FFIME......VYHRD......SDFGL......KADIW......
116265924	......YVMRL......IIKLY......YFVME......VYHRD......SDFGL......KADIW......
116265946	......SVMRL......IVQLY......YFVME......VYHRD......SDFGL......KADIW......
116265966	......STMKL......VVRMY......YIVLQ......VYHRD......SDFGL......KADLW......
116265938	......SAMHR......VLKIH......YLVME......VSHRD......SDFGL......KADAW......
116265932	......SVMKM......IVELH......YFAME......VYHRD......TDFGL......KADLW......
116265944	......STMKL......VIKIF......YIVIE......VFHRD......SDFGL......ASDVW......
116265942	......STMKL......VIKIF......YIVIE......VFHRD......SDFGL......ASDVW......
116265926	......ETMKL......VVRLY......FIVLE......VFHRD......SDFGL......TADLW......
116265922	......ATLKL......VVRLH......YMVLE......VFHRD......SDFGL......TSDIW......
116265964	......STMKL......VVRMY......YIVLE......VYHRD......SDFGL......KADLW......
116265962	......SIMHL......IVELK......FFVME......VSHRD......SDFGF......KADTW......
116265958	......SIMSR......IVRLH......YFVME......VFHRD......TDFGL......KVDIW......
116265970	......SIMKI......IVRLH......YIILE......VYHRD......SDFGL......AADVW......
116265936	......SVMKM......IVELN......YFAME......VYHRD......TDFGL......KADLW......
116265952	......SILRR......IVQLF......YFVME......VFHRD......SDFGL......KVDIW......
116265968	......SIMKL......IVMLH......YIILE......VYHRD......SDFGL......AADVW......
116265928	......ATMKR......VVRLY......FIVLE......VFHRD......SDFGL......PADLW......
116265948	......SIMRK......IVKLI......YCVME......VFHRD......SDFGL......KIDVW......
116265930	......SAMHR......ILKIH......YLVME......VSHRD......SDFGL......KADAW......
116265940	......SVMRL......IVQLN......YFVME......VYHRD......TDFGL......KADIW......
116265950	......SIMRR......IVKLI......YFVIE......IFHRD......SDFGL......KVDVW......
116265974	......SVMHL......IVELK......FFIME......VYHRD......SDFGL......KADTW......
116265954	......SILRR......IVQLF......YFVME......VFHRD......SDFGL......KVDIW......
116265960	......SVMRL......VVELY......YFVME......VSHRD......SDFGL......KADIW......
116265956	......SILRL......IVQLF......YFVME......VFHRD......SDFGL......KVDIW......
169866440	......EVLRH......ITPLL......VLVLP......IVHRD......TDFGL......ETDAW......
169867308	......ALSML......ICGMR......YMVFE......VVHRD......IDFGL......EVDVW......
169865730	......DLTKS......VVPLL......EIEVV......VAHRD......IDFGF......PLDVY......
169858220	......SIWSS......ILPLF......YFVTI......LVHRD......GDFGL......GQDIW......
169858334	......KIVRS......IIAFH......MITMT......VVHND......VDFGF......KSDVW......
169856611	......HHHRQ......ITQMY......WIVTE......IVHRD......GDFGF......EVDVW......
169861259	......QIHRR......IVSIH......YAVMD......IYHRD......ADFGL......HNDIW......
169854875	......EVLRT......IVRLY......GIILE......IVHRD......TDFGF......AVDIW......
169844071	......YVMSA......ICPLL......YLVLP......IVHRD......IDFGS......EQDVW......
169843311	......CISST......VVETV......CEVME......VAHRD......GDYGA......RVDIW......
169851350	......SLHYH......IIDLY......YFVMQ......VYHRD......SDFGL......MSDVW......
169851808	......ALHQL......VVTLH......YIIME......IYHRD......TDFGL......MFDIW......
169843000	......RIHSA......VLEFL......YMLME......VCHRD......SDFGL......PIDVW......
169851100	......TMMKL......IMRIY......FLVLE......IIHRD......ADWGM......ATDIW......
169845736	......EYLRT......IIKLY......IFVLE......IVHRD......ADFGL......EIDVW......
169849345	......VVMKL......IMKLY......YLVLE......IAHRD......ADFGM......AADIW......
169846911	......VLLSF......IVKCL......YLVQE......ISHRD......IDFGS......PAEVW......
169846891	......SIMEK......ICELK......NLVLE......IAHRD......ADFGL......LVDSW......
116672577	......VLMEG......LVKFV......FLVQE......IVHRD......IDFGI......ADDIY......
116670167	......RILST......LVKAH......GLIMD......FMHSD......ADLGV......ERDLY......
116666738	......CINKM......VVKFY......YLFLE......ITHRD......SDFGL......PVDVW......
123797745	......NIMKK......IVSLL......YLIME......IVHRD......IDFGL......KSDLW......
117306475	......ELLMM......IISLL......YLIME......IVHRD......IDFGL......KIDVW......
116874261	......QNLKL......IIKLY......FMVME......VVHRD......ADFGL......EVDIW......
117644574	......EIMSS......IIAIH......VIVME......VVHRD......ADFGL......EVDSW......
117616998	......ELLMM......IISLL......YLIME......IVHRD......IDFGL......KIDVW......
117616926	......AILKL......VLKLH......YLVLE......ICHRD......ADFGM......KADVW......
117617000	......ELLMM......IISLL......YLIME......IVHRD......IDFGL......KIDVW......
117616996	......GIMKR......IISLH......FLILE......IVHRD......IDFGL......KVDTW......
117616208	......QNLKL......IIKLY......FMVME......VVHRD......ADFGL......EVDIW......
118036898	......RKAQV......IITVY......YLTME......FVHCD......IDFGI......RDDIY......
118048423	......RSAAR......IMPIY......YIAMK......IIHRD......TDFGL......PTDIY......
118047984	......SIAAR......ISRLI......YTVFE......IVHRD......IDFGI......LSDIF......
118045544	......ELLAK......LPRVT......YLVME......IIFRD......VDFGI......RSDIY......
118047034	......-LAAS......LLPVI......YMVTP......VVHRD......TNLSL......SLDIY......
146305147	......ALTAR......VVRLF......FITLE......VLHGD......FDYGL......AADVY......
156742770	......QAAAK......IVEVY......YLVME......MVHRD......ADFGL......RSDIY......
156744038	......EITSQ......IVPIY......YTVME......VIHRD......ADFGI......LSDLY......

```
156742771   ......RAIAA.....IVEVY.....YLVME.....MIHRD.....TDFGL.....RSDIY.....
156740544   ......DSAAA.....IVPIY.....YIAMK.....VIHRD.....TDFGL.....PTDIY.....
156742101   ......TLLRE.....IVEYV.....YLATR.....IIHRD.....IDFSI.....ASDQF.....
118082815   ......EIMSS.....IITIY.....VIIME.....VVHRD.....ADFGL.....EVDSW.....
118085967   ......RCMKL.....IVRLY.....YLILE.....VVHRD.....TDFGF.....AVDIW.....
118087871   ......RIMKI.....IVKLF.....YLVME.....IVHRD.....ADFGF.....EVDVW.....
118088844   ......RIHQM.....VVQLY.....YMVME.....IVHRD.....VDFGL.....KVDVW.....
118091273   ......AILKL.....VLKLH.....YLVLE.....ICHRD.....ADFGM.....KADVW.....
118092044   ......RIMKI.....IVKLF.....YLIME.....IVHRD.....ADFGF.....EVDVW.....
118092046   ......RIMKI.....IVKLF.....YLIME.....IVHRD.....ADFGF.....EVDVW.....
118094947   ......AILLK.....IIKVL.....QLVME.....ILHRD.....VDFGS.....ELEMW.....
118101823   ......QIMKM.....IIKLY.....YLVTE.....VVHRD.....ADFGF.....QLDIW.....
118101966   ......QIMKM.....IIRLY.....YLVTE.....IVHRD.....ADFGF.....KVDIW.....
118102392   ......EIMSS.....IIAVH.....VIVME.....IVHRD.....ADFGL.....EVDSW.....
118103757   ......SSMEK.....IIRLY.....HLVME.....IIHRD.....GDFGF.....YVDIW.....
118462951   ......RTTAM.....IAAVH.....YLVME.....LVHRD.....TDFGI.....ASDVY.....
118469501   ......RVAAR.....VIPVH.....YIDMR.....LVHRD.....VDFGI.....PADIY.....
148539580   ......CIHRM.....IIRFY.....YLFVE.....VTHRD.....SDFGL.....PADVW.....
118498499   ......AL--R.....VLAPA.....LFTMD.....VVHRD.....SDFGI.....PADLF.....
118615936   ......RTTAM.....IAAVH.....YLVME.....LVHRD.....TDFGI.....SSDVY.....
118725943   ......EILKR.....LPRIC.....YIVED.....IIYGD.....IDFGI.....ATDIY.....
118505062   ......QNLKL.....IIKLY.....FMVME.....VVHRD.....ADFGL.....EVDIW.....
155369277   ......EIMKS.....IIKLL.....FIVLE.....IIHRD.....SDFGL.....AIDVW.....
119224653   ......QIMKM.....IIRLY.....YLVTE.....IVHRD.....ADFGF.....KVDIW.....
121700206   ......TVHKH.....IISFF.....WIAME.....VGHRD.....ADFGL.....DVDIW.....
121701787   ......AIVSL.....ICGMR.....YMLFE.....IVHRD.....IDFGL.....EVDVW.....
121701629   ......RVWRY.....ILTLD.....FCFTK.....VVHKD.....CDFGM.....SVDIW.....
121701021   ......SILRD.....IVRLH.....GIIME.....IVHRD.....TDFGF.....KVDVW.....
121705590   ......KLHHL.....VVSLV.....YVVIE.....IYHRD.....ADFGL.....PNDVW.....
121704074   ......VIMKL.....VISLY.....YLVLE.....ICHRD.....ADFGM.....KADIW.....
121710616   ......HVLDY.....IVEME.....YIEMK.....VVHRD.....IDFGS.....EQDIW.....
121712636   ......CISSS.....VIHTL.....CEVME.....VAHRD.....TDFGN.....AVDVW.....
121709942   ......HHHRQ.....IARLY.....WLVLE.....CVHRD.....CDFGF.....KVDVW.....
121713184   ......SIAQR.....IVESV.....NHVME.....VAHRD.....TDFGC.....RLDVW.....
121712744   ......DCHKR.....IVNLI.....YLVLE.....MYHRD.....GDFGL.....KADIW.....
121714020   ......MVMLL.....FPTLA.....AIVMS.....LSYDD.....IDFGL.....PNDVW.....
121710864   ......QYLQL.....IIKLY.....IMVLE.....IVHRD.....ADFGL.....EVDVW.....
119472956   ......TVHKH.....IISFF.....WIAME.....VSHRD.....ADFGL.....DVDIW.....
119482696   ......SLSSS.....ILRTI.....CLVTE.....IAHRD.....ADFGS.....RADVW.....
119478266   ......MIMLL.....FPTLH.....AIVMS.....ITYDD.....IDFGI.....QTALW.....
119479617   ......SIARN.....VVETV.....SHVME.....IAHRD.....TDFGC.....RVDIW.....
119467222   ......VIMKL.....VISLY.....YLVLE.....ICHRD.....ADFGM.....KADIW.....
119477300   ......EAMFK.....FVESF.....FVTME.....FAHRD.....GDFGV.....MVDIW.....
119467966   ......HHHRQ.....IARLY.....WLVLE.....CVHRD.....CDFGF.....KVDVW.....
119498565   ......CISSS.....VIHTL.....CEVME.....VAHRD.....TDFGN.....AVDVW.....
119488353   ......QLLRE.....LMRMA.....CLVMD.....WVHRD.....SDFGL.....GVDLW.....
119491821   ......KLHHL.....VVSLV.....YVVIE.....IYHRD.....ADFGL.....PNDVW.....
119494956   ......SILRD.....IVRLH.....GIIME.....IVHRD.....TDFGF.....KVDVW.....
119495572   ......RVWRY.....ILTLD.....FCFTK.....VVHRD.....CDFGM.....SVDIW.....
119497951   ......QYLQL.....IIKLY.....IMVLE.....IVHRD.....ADFGL.....EVDVW.....
119495767   ......AIVSL.....ICGMR.....YMLFE.....IVHRD.....IDFGL.....EVDVW.....
119498187   ......HVLDY.....IVEME.....YIEMH.....VVHRD.....IDFGS.....EQDIW.....
119498465   ......YCHKR.....IVNLI.....YLVLE.....MYHRD.....GDFGL.....KADIW.....
119472288   ......WVGQR.....VMKVL.....YHICE.....MVHRD.....IDYGV.....KSDLF.....
119493807   ......RTLKG.....IPQFY.....YLAME.....IIHRD.....LDFGA.....-SDLY.....
119486268   ......VVLDR.....IPNLL.....YIVQD.....VIHRD.....LDFGA.....STDLY.....
119513409   ......KTLGK.....LPRLL.....YLIQE.....VIHRD.....IDFGA.....ASDIY.....
119511037   ......LRLAL.....IVQVE.....CMVME.....LLHRD.....IDFGI.....YIDVY.....
119512146   ......AILYQ.....VPKFR.....FLVED.....IIHRD.....IDFGL.....HTDLY.....
119512045   ......SVLYK.....IPRFR.....FLVQD.....VIHRD.....IDFGA.....HSDLY.....
119511567   ......ATLEY.....IPKLY.....YLVQE.....IIHRD.....IDFGV.....ATDIY.....
119577740   ......RIMKG.....IVKLF.....YLVME.....IVHRD.....ADFGF.....EVDIW.....
119576455   ......SSMEK.....IIRLY.....HLVME.....IIHRD.....GDFGF.....YVDIW.....
119716210   ......RLAAR.....VVAVY.....WLVME.....IVHRD.....SDFGI.....ASDVW.....
119577736   ......RIMKG.....IVKLF.....YLVME.....IVHRD.....ADFGF.....EVDIW.....
119577737   ......RIMKG.....IVKLF.....YLVME.....IVHRD.....ADFGF.....EVDIW.....
119589029   ......NILKS.....IIHLE.....YLVME.....IVHRD.....TDFGL.....QCDIW.....
119594585   ......RIMKV.....IVKLF.....YLVME.....IVHRD.....ADFGF.....EVDVW.....
119594580   ......RIMKV.....IVKLF.....YLVME.....IVHRD.....ADFGF.....EVDVW.....
119589945   ......QLLRR.....VIQLV.....YMVME.....IVHKD.....SDLGV.....KVDIW.....
119591648   ......AILSR.....IIKVL.....QLVME.....IIHRD.....IDFGS.....ELEMW.....
119594586   ......RIMKV.....IVKLF.....YLVME.....IVHRD.....ADFGF.....EVDVW.....
```

119589944	......QLLRR.....VIQLV.....YMVME.....IVHKD.....SDLGV.....KVDIW......
119591642	......AILSR.....IIKVL.....QLVME.....IIHRD.....IDFGS.....ELEMW......
119591647	......AILSR.....IIKVL.....QLVME.....IIHRD.....IDFGS.....ELEMW......
119897469	......WVARR.....VIKAW.....YVAME.....MLHRD.....ID--L.....AADLF......
119898876	......AVASR.....IIRIF.....YVVME.....IVHRD.....TDFGL.....KTDLY......
119897547	......RIVSP.....IVPIF.....YLVFE.....IIHRD.....MDFGI.....RIDVF......
170720795	......WFLRR.....FPELH.....YYVMR.....LLHRD.....LDFGL.....RQDLY......
159040276	......RAAGR.....VVDLF.....YLIME.....VLHRD.....ADFGL.....AVDVY......
159037852	......RIMAA.....VVQVF.....YLIME.....VVHRD.....IDFGV.....ATDIY......
119874973	......QIQQM.....ITQLL.....YLVME.....VVHRD.....IDFGL.....KIDVW......
119890140	......QNLKL.....IIKLY.....FMVME.....VVHRD.....ADFGL.....EVDIW......
119890620	......AILCK.....IIKVL.....QLVME.....ILHRD.....IDFGS.....ELEMW......
119895231	......QLLRR.....VIQLV.....YMVME.....IVHKD.....SDLGV.....KVDIW......
119622843	......AILKL.....VLKLH.....YLVLE.....ICHRD.....ADFGM.....KADVW......
119602222	......RIMKI.....IVKLF.....YLIME.....IVHRD.....ADFGF.....EVDVW......
119602223	......RIMKI.....IVKLF.....YLIME.....IVHRD.....ADFGF.....EVDVW......
119602219	......RIMKI.....IVKLF.....YLIME.....IVHRD.....ADFGF.....EVDVW......
119602224	......RIMKI.....IVKLF.....YLIME.....IVHRD.....ADFGF.....EVDVW......
119613704	......RIMKI.....IVKLF.....YLVME.....IVHRD.....ADFGF.....EVDVW......
119622846	......AILKL.....VLKLH.....YLVLE.....ICHRD.....ADFGM.....KADVW......
119622844	......AILKL.....VLKLH.....YLVLE.....ICHRD.....ADFGM.....KADVW......
119629901	......QLMKL.....IIKLY.....YIVTE.....IVHRD.....ADFGF.....QLDIW......
119906386	......NSLKT.....IVKLL.....FIVME.....VVHRD.....SDFSL.....EEDVW......
119907074	......QIMKM.....IIRLY.....YLVTE.....IVHRD.....ADFGF.....KVDIW......
119907014	......KIMKL.....IIKLY.....YLVTE.....IVHRD.....ADFGF.....QLDIW......
119908074	......EIMSS.....IIAIH.....VIVME.....VVHRD.....ADFGL.....EVDSW......
119908176	......RIMKI.....IVKLF.....YLVME.....IVHRD.....ADFGF.....EVDVW......
119911126	......AILKL.....VLKLH.....YLVLE.....ICHRD.....ADFGM.....RADMW......
119919553	......AGGSP.....VGQGH.....YLVLE.....ICHRD.....ADFGM.....KADVW......
119921848	......RIMKI.....IVKLF.....YLIME.....IVHRD.....ADFGF.....EVDVW......
119961778	......EIHGR.....IVRIL.....FVVME.....IVHND.....ADFGI.....ASDIY......
119962082	......NLMAQ.....IVTIF.....YLAME.....IVHRD.....TDFGI.....PVDIW......
119964125	......RLLAG.....LVAAF.....FLVME.....VIHRD.....ADFGV.....ASDIY......
162461684	......SVMRL.....VVQLH.....YFAME.....VYHRD.....SDFGL.....KADIW......
162461847	......GTLKL.....VVRLH.....YMVLE.....VYHRD.....SDFGL.....LSDLW......
120603505	......TVMAS.....VAAVW.....FFVME.....IVHRD.....IDFGL.....RADLY......
120611728	......AACAR.....IVQLL.....FAVFE.....ITHRD.....LDFGI.....QADLY......
121309739	......MNHRS.....IIRFK.....AIVME.....ICHRD.....CDFGY.....IADVW......
153004050	......RLVVQ.....LVSIH.....YIAMD.....VVHRD.....IDFGI.....RSDVF......
153004299	......EAAAQ.....IVALH.....YLVFE.....VMHRD.....LDFGL.....RADVF......
153003112	......RAYAR.....IVGVH.....YLVTD.....LLHRD.....MDFGI.....GADVW......
153003980	......RAVNL.....IVGIY.....YYVME.....VVHRD.....VDFGI.....RTDVY......
153003203	......EIASA.....IVRVT.....FLVME.....VVHRD.....LDFGI.....RADLY......
158520571	......EMAAI.....IIQVY.....YFTMQ.....IVHRD.....MDFGI.....RVDVY......
123315580	......DCLMR.....IVRLY.....YMVLE.....IAHCD.....GDFGL.....KSDIW......
123318718	......RIMAR.....IAKVH.....MIVME.....ICHRD.....IDFGF.....KAYIW......
123368595	......DALCK.....IMRIY.....FLILE.....FAHHD.....SDFGM.....KSDVW......
123383043	......NALVE.....VIKIY.....FIILE.....IANHD.....ADFGF.....LSDIW......
123377403	......RLLQI.....LPKIH.....FIVME.....ISHRD.....IDFGL.....KADIW......
123381252	......NIMRE.....AVKFY.....YIVME.....VAHRD.....IDFGL.....AADMW......
123376497	......SLMRM.....IIKLH.....YIILE.....ICHRD.....ADFGF.....AADIW......
123377387	......RLCIR.....IIKVH.....MIIMD.....IAHHD.....IDFGL.....KSDIW......
123382064	......RLMQS.....VIHTF.....YIVME.....VAHRD.....SDFGL.....KSDIW......
123385232	......KALTS.....IVRVY.....FLILE.....FAHCD.....CDFGF.....KCDIW......
123391599	......ECMKL.....IVNLY.....YMLLE.....ISHSD.....GDFGL.....KSDIW......
123392026	......NALSK.....IINVF.....FLILD.....ITHRD.....ADFGL.....KADVW......
123393985	......ECFKQ.....IVSLY.....YLLME.....IAHCD.....TDFGL.....YADIW......
123392747	......RLLSR.....IVKVY.....MIVME.....IAHRD.....VDFGL.....KSDIW......
123407199	......RIQSR.....IAKVY.....AIVME.....ISHRD.....IDFGF.....KADIW......
123404173	......NALTH.....VVRVY.....AIILE.....ITHSD.....CNFGL.....KADVW......
123405907	......RIDQQ.....IVQIV.....YVVME.....IAHRD.....SDFGL.....ASDIW......
123402445	......RLCSR.....IVKVF.....MIVME.....ICHKD.....IDFGL.....KADIW......
123400888	......QLLQD.....IIQLF.....-LVFE.....IVHRD.....IDWGC.....GGDCW......
123408499	......AIYNK.....LTQFY.....YIALE.....IIHRD.....IDFGL.....SIDIW......
123402448	......RLCSR.....IVKVF.....MIVME.....ISHRD.....VDFGL.....KADVW......
123405836	......RVIQN.....IIGVK.....MMVMP.....ISHRD.....IDFGL.....KVDVW......
123403339	......ELMER.....ISDLY.....YIIME.....IAHRD.....IDFGL.....SCDIW......
123401530	......RLTCR.....IVKVL.....MIIME.....VCHRD.....IDFGF.....KADVW......
123398160	......AIMRL.....LLHLE.....FLVEQ.....ICHRD.....ADFGL.....IADIW......
123402441	......RLSSR.....IVSVY.....MIVME.....ICHRD.....IDFGL.....KADIW......
123405686	......RLCVR.....IVKIY.....MMVME.....ISHRD.....IDFGL.....KADIW......
123408149	......DVLQS.....IVRLY.....FLLME.....IAHND.....CDFGL.....KADIW......

123404237	......TVLSN......IIGVY......VLVLE......VAHHD......ADFGI......QSDIW......
123404990	......RLASR......VVKVY......MIIME......VAHRD......IDFGL......KSDVW......
123408325	......RLIQR......LPAVQ......MIVME......IAHRD......VDFGF......KADIW......
123405790	......KALMN......IIRLY......FVILE......IAHRD......SDFGI......QADIW......
123405682	......RLCSR......IVKIL......MIVME......ISHRD......IDFGF......KSDIW......
123401527	......RLSTR......IAKVY......MIIME......ISHRD......IDFGL......KADLW......
123416004	......ALMRF......LLKLE......YIVME......FCHRD......GDFGF......CADIW......
123414011	......IVLKS......IPQLF......FIVKE......VIFDD......VDYEF......KADTW......
123416156	......QILRE......ICGLY......YIVLE......IVHRD......IDFGF......SVDIW......
123410541	......EILSK......IVRIY......FMILE......FAHCD......CDFGL......KADVW......
123412897	......RLSAR......IVKVY......MIIME......ISHRD......IDFGL......KADVW......
123415043	......KVLPT......IVKFI......YLIEE......VSHRD......IDFGL......KADIW......
123412462	......RILSN......IVRFI......FLIME......LLHRD......IDFGL......KADIW......
123420211	......NAMAF......IVALY......YLIID......IAHRD......SDFGL......LSDVW......
123413776	......QNMMS......IVTLY......YMIQE......IAHRD......GDFGF......LADIW......
123412893	......RLSSR......IAKVH......MIVME......ISHRD......VDFGL......KADIW......
123431372	......RLLAR......IVKIY......MIVME......IAHRD......IDFGL......MSDVW......
123427907	......NALVE......VIKIY......FIILE......IANHD......ADFGF......LSDVW......
123432614	......HLHMK......ISQMF......YLIIE......IAHRD......TDFGI......KSDIW......
123424859	......KIFQQ......IITLY......YIFME......IAHRD......SDFGL......TSDVW......
123436254	......EIMAA......ILDAK......IILMS......IVHRD......SDFSV......KADVW......
123427345	......RLISR......LPKIY......MIIME......IAHRD......IDFGF......KADMW......
123434356	......AVMRI......LLKLL......YLVLE......ICHRD......ADFGF......PADIW......
123434194	......KILKK......IVRLH......YMIME......FVHQD......GDFGI......PADVW......
123434214	......RLSER......IVKVF......MIVME......VCHRD......IDFGV......KVDVW......
123428218	......EILAS......VLPII......ILMMP......IVHRD......SDFSV......KADVW......
123431314	......RMLQT......ILKLY......YMIME......IVHQD......SDFGI......KEDVW......
123431310	......HITEL......IIQLL......NLVLE......ILHKD......IDFGL......DSDIY......
123427555	......DILSN......IIGVF......VLVLE......VAHHD......ADFGI......QSDIW......
123437289	......NIMRS......IIQAM......CVVMP......IMHRD......ADFGF......KIDLW......
123432124	......HIHKQ......LVRFY......YIIME......VCHMD......IDFGI......KVDIW......
123426587	......DIMRL......ICDLY......YLVME......VAHRD......IDFGL......SSDVW......
123426047	......RIISR......FPEIY......MVVME......IAHRD......IDFGL......KADIW......
123429283	......ALMKI......IIKLI......FIIQE......ICHRD......ADFGF......KADIW......
123445150	......SVFMQ......VCSCY......ILFIE......LVHRD......TDFGL......KCDVW......
123445565	......SVFMQ......ICSLY......YFFME......LAHRD......TDFGL......KCDVW......
123439047	......NAQAF......VIQTY......VLVME......IVHND......IDFDL......KVDVW......
123445077	......EILRK......VAELF......CIIME......IVHRD......IDFGL......AADIW......
123438401	......STLRL......VTELY......WVVLE......CVHRD......ADFGF......YVDSW......
123444757	......RILEH......IVDIY......MIIME......IMHRD......IDFGL......AVDIW......
123446373	......SALIH......IIQIY......FIILE......YSHND......ADFGL......KSDIW......
123447338	......ECMMR......IVALY......YLLME......IAHCD......ADFGL......AADIW......
123445124	......RLSAR......VIKVH......LIVMD......IAHRD......IDFGL......KGDIW......
123439673	......SFLKQ......ISELF......YIVME......IAHRD......IDFGL......SSDIW......
123447886	......ALMKL......IIRLI......FIVTE......ICHRD......GDLSF......KADIW......
123445126	......RLISR......IVKMY......CIIME......ISHRD......IDFGF......KADMW......
123445542	......KILST......CLKII......YLIVP......IVHRD......CDFSV......KSDVW......
123440000	......DALIN......IIKIY......YIILE......IAHHD......ADFGF......ASDVW......
123439920	......SLLQQ......ITHFY......YVFME......IAHRD......TDFGL......AADVW......
123447177	......ECLKR......IVNLY......YMLLE......IAHCD......TDFGL......AADIW......
123444390	......RIMQK......IIKLL......TLVIE......IAHRD......IDLGL......AADVW......
123438280	......RLIFR......FPKYY......ILVME......IAHRD......IDFGF......KADIW......
123438351	......VIAPL......IARVY......FQIMD......LCHRD......TDFGF......KADIW......
123438323	......EIMSQ......LIKLH......FLVLD......AVHRD......ADFGL......PADIW......
123439591	......RLSER......LPKFY......CLLME......ISHRD......IDLGL......KADIW......
123446940	......SILSA......IAEYH......YLFQE......VGHRD......IDFGL......RSDMW......
123446952	......DIMYE......VLKIV......LIITQ......IVHRD......SDFSV......KADVW......
123447672	......AILRN......VIKIY......MIVME......IAHHD......VDFSV......KADIW......
123437868	......RLCSR......IVKVY......MIVME......ISHRD......IDFGL......KSDMW......
123446233	......VLMRI......LLRFI......YIITE......ICHRD......ADFGF......AADVW......
123445993	......RILER......IAKFH......VIIME......IAHRD......LDFGF......KSDMW......
123445630	......KALST......IIRLY......YYILE......IAHRD......TDFGL......KGDVW......
123439031	......QIMKM......TVDFY......YIVME......IVHRD......IDFGL......QVDIW......
123457082	......TLMKL......LLGLI......YIITE......ICHRD......ADFGF......KADIW......
123456779	......KFHKN......ILHFY......FVILE......LLHSD......IDFGL......ASDIW......
123457131	......RIQQQ......VVQIY......YIMME......VVHRD......SDFGL......ISDMW......
123975987	......DALKK......ICRFF......YLIME......IAHRD......IDFGF......KVDIW......
123457151	......EILRS......ISALY......YAVID......ILHRD......VDFGV......KSDIW......
123457257	......EIVKR......IVSVK......FIVQE......IAHRD......IDFGF......KTDIW......
123453428	......RLHSR......IVKVL......MIVMD......IWIRD......IDFGL......QADIW......

```
123456533    ......ALMSF.....LLKLV.....YIVME.....FCHRD.....GDFGF.....CADIW.....
123453907    ......RMITR.....IVKVQ.....LIIME.....IAHRD.....IDFGF.....KADIW.....
123457328    ......RINQQ.....VVALI.....YIIME.....VCHRD.....SDFGL.....KSDVW.....
123975447    ......QLMSE.....IVPLI.....IIILP.....IIHRD.....SDFSV.....PADCW.....
123455769    ......RVLQS.....IAQIY.....IVVME.....IAHRD.....IDFGV.....KSDIW.....
123452699    ......KLLSN.....IIQIY.....GAILD.....IVHRD.....SDFGL.....AADIW.....
123453850    ......NALIN.....IVRVY.....FIVLE.....FAHCD.....CDFGL.....KADIW.....
123449485    ......RLCSR.....IVKVY.....LIVME.....IAHRD.....IDFGM.....KGDIW.....
123454355    ......RLSTR.....VCKVL.....MVVME.....ISHRD.....IDFGL.....KADIW.....
123456369    ......NLLKQ.....INELY.....YLIME.....VAHRD.....IDFGL.....AADMW.....
123454468    ......TALLQ.....IVYTF.....YIISE.....ICHRD.....IDFGF.....SCDIF.....
123455002    ......EIMKI.....IVELF.....FLIMD.....IAHRD.....IDFGL.....ASDIW.....
123457255    ......EILSK.....IVRIY.....FIILE.....IAHCD.....CDFGL.....KSDVW.....
123452665    ......RIDQQ.....IVQIV.....YVVME.....IAHRD.....SDFGL.....SSDVW.....
123974842    ......RLSTR.....IVKTY.....LIVME.....ICHRD.....IDFGM.....KADIF.....
123449384    ......DIMKK.....VCDLY.....YIVME.....IAHRD.....IDFGL.....ASDVW.....
123456337    ......QLLQD.....IVQLY.....-LVFE.....VVHRD.....IDWGC.....AGDCW.....
123456440    ......QTLVM.....IIKLY.....YLILE.....ISHRD.....ADFGL.....KADVW.....
123455231    ......RILER.....LIKVY.....LIVME.....IAHRD.....IDFGL.....KADIW.....
123976145    ......TALST.....IIRLY.....YYILE.....IAHRD.....SDFGL.....KGDVW.....
123975991    ......CIMKE.....ICRFY.....YIVME.....AAHRD.....IDFGF.....KIDVW.....
123448960    ......RIMER.....IVNFE.....IVVME.....IAHRD.....IDFGM.....KADIW.....
123476142    ......EILKS.....IIYLY.....YMVYE.....ISHGD.....SNFDL.....KADIW.....
123470583    ......KSLAS.....ITRLY.....YLVME.....ISHGD.....CDFGM.....KADIC.....
123470106    ......RINQQ.....IVSLY.....YIIME.....ICHRD.....SDFGL.....KSDIW.....
123477862    ......QCLMS.....IIRLY.....YLLME.....IAHCD.....TDFGM.....KADIW.....
123472696    ......SILKS.....ISELF.....YLIME.....VAHRD.....IDFGL.....ASDIW.....
123466981    ......YALSV.....IVRLY.....FLILE.....FAHHD.....ADFGL.....FVDIW.....
123468845    ......SILRK.....ITELY.....YLVME.....IAHRD.....IDFGL.....TADIW.....
123471643    ......STRIQ.....IVRVY.....AIILE.....IAHSD.....CDFGF.....KADVW.....
123475037    ......ELLMA.....TIQLI.....-LVFE.....IVHRD.....IDWGC.....GCDTW.....
123458325    ......RILAT.....VIRLY.....IVVTE.....IFHRD.....IDWGL.....AVDIW.....
123469511    ......RFHQS.....ICQLY.....FLIME.....IAHRD.....GDFGF.....KSDIW.....
123464790    ......GALSK.....VINIY.....FIILD.....ISHRD.....GDFGL.....KADVW.....
123477860    ......DCFQL.....IVNLY.....YLLME.....IAHSD.....NDFGN.....KADIW.....
123472833    ......RLSSR.....IIKVF.....MIVME.....ISHRD.....IDFGL.....KADVW.....
123471038    ......DIQAS.....MQNVI.....LIVFD.....VAILD.....GVLTS.....AADIW.....
123472831    ......RLSSR.....IVEVY.....MIIME.....VSHRD.....IDFGL.....KSDIW.....
123478346    ......EISAS.....IIDLY.....YIVME.....IAHRD.....IDFGL.....EADIW.....
123472133    ......RLHSE.....IVKIF.....AIIME.....IYHRD.....IDFGM.....KIDIW.....
123469827    ......RINQQ.....IVGMI.....YVIME.....VTHRD.....SDFGL.....TSDVW.....
123463184    ......NVIIK.....IIKIH.....YLVMD.....IAHRD.....IDFGF.....LADLW.....
123472843    ......KVLPT.....IVKFI.....YLVEE.....ISHRD.....IDFGL.....KADIW.....
123473462    ......EILKK.....ISHLF.....YIVME.....VCHRD.....IDFGL.....GCDVW.....
123477179    ......NFLKQ.....IAQYF.....YFVME.....VAHRD.....IDFGF.....QADVW.....
123459124    ......ALMQF.....LIELY.....YMILE.....ICHRD.....ADFGF.....IADVW.....
123472038    ......ELLSS.....IQKYI.....YIILE.....IVHLD.....GNFYN.....AADVF.....
123460555    ......ILSPL.....IIHVE.....FQFMQ.....IVHRD.....SDFGL.....KADMW.....
123473209    ......EIHKV.....IVQLY.....YLIME.....LIHRD.....IDFGL.....SHDIW.....
123475318    ......RILQQ.....IAQLF.....YVVLE.....VVHRD.....TDFGF.....KNDIW.....
123458344    ......KLMKM.....CLTFY.....YMAME.....IAHRD.....IDFGL.....SADLW.....
123463809    ......RTLQT.....IVRLY.....YLIME.....VAHRD.....ADFGL.....ASDVW.....
123477033    ......ELMKQ.....IVQLY.....YLVME.....ISHRD.....IDFGL.....GSDIW.....
123467029    ......ENFGK.....ILRLI.....YMIMT.....IAHRD.....ADFGF.....MADMW.....
123458061    ......ALMRL.....LLKLI.....YIILE.....ICHRD.....ADFGF.....ASDIW.....
123472107    ......TLLKR.....IRSIQ.....FQVME.....IAHHD.....SDIDL.....KADVW.....
123478146    ......RIMQQ.....IVQLF.....YIVTE.....VCHRD.....SDFGF.....KSDIW.....
123471472    ......ALMRL.....LLKLI.....YIVTD.....ICHRD.....ADFGF.....AADVW.....
123474754    ......ELMSK.....IIKNL.....CVITQ.....IIHRD.....SDFSV.....IADVW.....
123478425    ......HCLIE.....VCNLY.....YLLME.....IVHVD.....CDFGL.....IADIW.....
123475552    ......HALTV.....IIRLY.....FLILE.....IVHHD.....ADFGL.....FVDIW.....
123462103    ......RVLER.....IVKIQ.....CIIME.....VAHRD.....VDFGL.....KADIW.....
123457925    ......RLHSR.....IVKIH.....MIVMD.....ICIRD.....IDFGL.....KAHIW.....
123473308    ......KALMS.....IIRLY.....FIILE.....IAHRD.....SDFGI.....QADIW.....
123488803    ......RINQQ.....VVGLI.....YVVME.....ISHRD.....SDFGL.....TSDVW.....
123501733    ......DCMKQ.....IVRLY.....YLLME.....VAHLD.....CDFGF.....KADMW.....
123498927    ......SIMQI.....IAQLY.....FLFME.....VVHRD.....IDFGL.....AADIW.....
123495188    ......RCLES.....VVKLY.....NFVLE.....IIHRD.....CDFGS.....ALDLW.....
123482966    ......SLLQQ.....IMHFF.....YVIME.....IAHRD.....SDFGL.....SADIW.....
123483667    ......ECIKH.....VIRIY.....YILME.....IAHCD.....SDFGL.....KADIW.....
123502987    ......DCLMT.....IVRLY.....YLLME.....IAHGD.....CDFGM.....RADIW.....
```

```
123482300    ......ALMRL.....LLKLK.....YVILE.....ICHRD.....ADFGF.....LADIW......
123479049    ......RISQQ.....VVALY.....YIIIE.....ICHRD.....SDFGL.....KSDIW......
123495361    ......ETMRM.....IISLR.....YIVIE.....IVHQD.....SDFGI.....KEDVW......
123481830    ......NSLAS.....IIKLY.....FLILE.....LCHLD.....ADFGF.....KADIW......
123503314    ......RIAQQ.....IVELC.....YVIME.....VSHRD.....SDFGL.....TTDVW......
123479216    ......RALIS.....IIRIY.....VMILE.....VAHRD.....GDFGL.....AADVF......
123509751    ......DIHSS.....IVKFY.....YIVME.....FIHHD.....CDFGL.....ACDIW......
123510098    ......DTTAN.....IVNMH.....YLVLD.....VAHRD.....SDFGL.....KSDIW......
123489961    ......SVFMQ.....ICSLY.....YFFME.....LVHRD.....TDFGL.....KCDVW......
123482482    ......QCLVN.....VVRLY.....YMLME.....AAHSD.....SDFGM.....IVDIW......
123509274    ......LALTT.....IVRLY.....YMILE.....IAHHD.....ADFGL.....LADIW......
123502058    ......QVIIK.....IIKIH.....YLIMD.....IAHRD.....VDFGF.....MADMW......
154411795    ......EALKS.....VISYY.....NILME.....FAHRD.....SDFGV.....QADMW......
123481907    ......KIQKQ.....IVTFY.....VIVME.....VIHRD.....ADFGL.....SVDVW......
123508776    ......RINQQ.....VVQII.....YIFME.....IAHRD.....SDFGL.....KSDLW......
123499124    ......EILGS.....IQKLI.....AQIVE.....IVHLD.....GGF-L.....KADVW......
123499315    ......QVMSM.....CIQFK.....YIIEE.....IVHRD.....GDYSA.....PADVW......
123499410    ......DIMKH.....IADFY.....FIVME.....VVHRD.....IDFGL.....SSDIW......
123505938    ......DTMAY.....LVKLH.....YLVLD.....IAHRD.....SDFGL.....LSDIW......
123494857    ......NALTD.....IIRVF.....IIILE.....FAHRD.....ADFNL.....KTDIF......
123498639    ......RITSR.....IVHVY.....YIIME.....ISHRD.....IDFGF.....KADIW......
123506247    ......QILSN.....IVRIY.....FQILE.....IAHCD.....CDFGL.....MADVW......
123492314    ......RIMRR.....IADFY.....YLVME.....IAHRD.....IDFGF.....SSDIW......
123480523    ......KLMQS.....TVQFF.....YIVME.....MAHRD.....IDFGL.....AADIW......
123491348    ......EIMKK.....ITELY.....FLVME.....VAHRD.....IDFGL.....SSDIW......
123479406    ......LIHSK.....VTQLK.....YIFLE.....VAHRD.....TDFGL.....KADIW......
123481626    ......NIHKI.....IATCY.....FLSME.....VIHRD.....IDFGL.....SVDIW......
123492491    ......LIHSK.....ITQLK.....YIFIE.....VAHRD.....TDFGL.....KADIW......
123499495    ......ELMKT.....SVNVY.....YLIME.....IAHCD.....SDFGL.....ASDIW......
123485331    ......AIISS.....IVRYH.....YLFQE.....VGHRD.....VDFGL.....QADIW......
123479452    ......KALMS.....IIRLY.....YMILE.....IAHRD.....SDFGI.....QADIW......
123498651    ......DCMKQ.....VIRLY.....YMVME.....IAHKD.....CDFGL.....EADIW......
123977203    ......KILNE.....IISKK.....SLCLE.....IIYRD.....SDFGL.....KVDIW......
154414544    ......RMMKL.....TCQFY.....GAVLE.....MIHQD.....IDFGF.....SSDIF......
124000947    ......EVMRK.....IVKFY.....YLIIE.....IVHRD.....IDFGL.....PADVW......
154420001    ......SIMRL.....LLKLV.....YIILE.....ICHRD.....ADFGF.....AADIW......
154412827    ......EIQSK.....IVKSY.....YILME.....IIHRD.....IDFGV.....QVDIW......
154417261    ......SVFMQ.....LAKLF.....FFFME.....IAHRD.....IDFGL.....KCDVF......
154421118    ......AYLEK.....ILKPI.....CSVFP.....VMHRD.....IDLGM.....ECDIF......
154412702    ......EILKS.....IAKFI.....YIITE.....IVHRD.....IDFGL.....PADIW......
123967314    ......DLLKK.....CVSFY.....YLVME.....CVHRD.....IDFGL.....NTDLW......
154416554    ......SLMRR.....ILKLF.....YLVIE.....MVHQD.....ADFGV.....KEDVW......
154421028    ......EALVK.....IMRIY.....FMILE.....FAHHD.....SDFGM.....KADVW......
154413442    ......RILEK.....ICKLY.....MVVIK.....ICHRD.....IDFGI.....AADIW......
154421604    ......DVMAY.....IVQLY.....FLVLD.....VAHRD.....SDFGL.....KSDIW......
154416369    ......ALMRL.....ILKLL.....YIVLE.....ICHRD.....ADFGF.....KADIW......
154422983    ......RLSTR.....IVKIY.....VIAME.....IAHRD.....IDFGL.....KADIW......
124001135    ......DAMAY.....LVALH.....YLVMD.....IAHRD.....ADFGL.....LSDVW......
154419337    ......EIMKK.....ITDFY.....FIVME.....VAHRD.....IDFGL.....SCDIW......
154413760    ......DIMMK.....IVSLY.....YLVME.....IVHRD.....LDFGL.....KVDMW......
154418600    ......NVFKL.....VVEFY.....VVALE.....IAHRD.....IDFGF.....LSDVW......
154421116    ......ELMSN.....IIQAR.....CLVMP.....ICHRD.....TDLGF.....SIDIW......
154420486    ......ALMRL.....ILKLL.....YIGLE.....ICHRD.....ADFGF.....CADVW......
154416004    ......QALIN.....ILRSY.....FMILE.....YAHRD.....ADFGL.....KVDIW......
154413062    ......TALMS.....IIRLY.....FMILE.....IAHRD.....SDFGI.....QADIW......
154415971    ......ALMGL.....IMRLI.....YLVLE.....ICHRD.....ADFGF.....KADIW......
154416078    ......ALMRL.....LLKLI.....YIVLE.....ICHRD.....ADFGF.....KADIW......
124001043    ......AIMKQ.....IAQIY.....YLIME.....IIHRD.....IDFGL.....AADIW......
154415803    ......RIIRQ.....IVQLY.....YIIME.....ICHRD.....SDFGL.....KSDMW......
154421929    ......RIMKY.....IVQLY.....YIIME.....IAHLD.....GDFGI.....FIDMW......
122937359    ......RIMKV.....IVKLF.....YLVME.....IVHRD.....ADFGF.....EVDVW......
123226421    ......AMMLR.....IIRLH.....VLVLE.....VFHGD.....IDFGC.....PAYVW......
123229624    ......RIMKT.....IVQLF.....YLVME.....IVHRD.....ADFGF.....EVDIW......
123232864    ......AMMLR.....IIRLH.....VLILE.....VFHGD.....IDFGC.....PAYVW......
123226420    ......AMMLR.....IIRLH.....VLVLE.....VFHGD.....IDFGC.....PAYVW......
123232866    ......AMMLR.....IIRLH.....VLILE.....VFHGD.....IDFGC.....PAYVW......
123226423    ......AMMLR.....IIRLH.....VLILE.....VFHGD.....IDFGC.....PAYVW......
123233069    ......RIMKI.....IVKLF.....YLVME.....IVHRD.....ADFGF.....EVDVW......
122720713    ......SIMKI.....IVRLY.....YIVLE.....VYHRD.....SDFGL.....AADIW......
122937793    ......STLEA.....ILRLF.....YLVSE.....YVHRD.....ADFGF.....PVDIW......
124007123    ......AILKL.....VLKLH.....YLVLE.....ICHRD.....ADFGM.....KADVW......
```

124056495	......RIMKV......IVKLF......YLVME......IVHRD......ADFGF......EVDVW......
124268135	......FLHEH......IVTVF......YIAME......IVHRD......LDFGI......RTDVF......
145474551	......HILKL......VIQLY......FLVME......IVHRD......VDFGL......LVDIW......
145475315	......QILKQ......LVQLY......FLVME......VVHRD......IDFGL......QVDIW......
145475759	......SIMKK......VIKLY......YLVLE......VLHRD......GDFGL......KVDIY......
145475781	......QILRQ......ISNLV......FLIME......IFHRD......IDFSL......NIDVY......
145475607	......EIHPT......IVKVK......-IVLE......YVHMD......GDFGN......KMDIW......
145475373	......SINNA......FIKLH......YMVMD......IMHRD......VDFGL......VCDVF......
145475783	......RILRR......VQKYI......IIVQE......VMHRD......TSFGL......KVDVY......
145477133	......QIGSM......LIKVK......YQIME......ICHRD......LDFGV......KVDIW......
145475909	......SITKL......IINLI......HIITE......IVHRD......IDFGL......SVDVF......
145478881	......QIHRG......LITLL......CLVYD......LAHRD......SDLGF......CVDLF......
145477725	......KNTRI......IIKVY......YLVME......IAHRD......IDFGV......RVDLW......
145479671	......LNTVS......ICQML......YLVME......LCHRD......IDFGV......KIDIW......
145478203	......EIHQK......IVKML......YVVLE......VAHRD......CDFGS......LADVW......
145477705	......VILEL......IVNLY......VLEME......VIHRD......IDFGL......CLDIW......
145476551	......NLLKA......IVKLY......YLIME......IIHRD......IDLGL......AVDIF......
145477863	......KILKE......IVKVF......IIIME......FVHRD......IDFGL......CVDVW......
145479871	......ALLKD......IVKLF......YLVTE......IIHRD......IDFGT......KCDIW......
145476849	......SILRK......LIKMY......YLVQD......ILHRD......ADFGL......KVDIF......
145479817	......AIMKK......VVQLF......YLVME......VIHRD......ADFGV......PVDIW......
145478009	......HILNQ......IIKLI......NLIME......IIHRD......IDFGF......EVDMW......
145476941	......SILNA......IVKLH......TMVLE......IVHCD......IDFGV......SQDVL......
145476821	......SILRK......VVQLF......YIVTE......IVHRD......VDFGL......FTDIW......
145478787	......KNARM......IIKVY......FLVME......VAHRD......IDFGV......KVDLW......
145482079	......KILQM......VVQLY......YLFME......IVHRD......VDFGL......QTDLW......
145482199	......QILYR......IVRIK......VIIHE......LLHRD......IDFGL......KSDIF......
145485554	......HILRK......IIQLY......YLITE......IVHRD......VDFGL......KTDIW......
145485564	......KILRL......VMTLI......FVVQE......VIHRD......SDFGL......KVDVY......
145485727	......HILRK......IVQLY......YLIME......VAHRD......VDFGL......KTDIW......
145482269	......SILRK......ILKMY......YLVTE......IVHRD......IDFGL......EVDYW......
145482157	......TLMKL......CIRLY......YLIME......ILHRD......CDFGV......ASDIW......
145483085	......QVHSQ......LVELY......TLILE......YIHCD......GDLGL......KIDIW......
145481423	......QILKQ......LVQLY......FLVME......IVHRD......IDFGL......NSDIW......
145485881	......RLLRY......VIKLY......FVVME......VAHRD......ADFGL......DADVW......
145482679	......KILKS......ILKMI......YIIFE......YVHRD......IDFGF......RSDIF......
145484653	......KNTRI......IIKVY......YLVME......VAHRD......IDFGV......RVDLW......
145481613	......KMMQL......IPGVI......CIMMD......VAHRD......CDFGY......EADLF......
145482155	......LIQKK......ILKLC......FLILE......IVHRD......GDFGW......KSDLW......
145484789	......KILKE......IVKVF......IIIME......FIHRD......IDFGL......CVDVW......
145484988	......KIMQL......IPGVI......CIMMD......VAHRD......CDFGY......EADLF......
145483227	......AIMKK......VVQLY......YLVME......VIHRD......ADFGV......PVDIW......
145485963	......AIMKK......LIQLF......FMVLE......VVHRD......ADFGV......AADIW......
145482103	......QILQS......IVQLL......QLVME......IIHRD......IDFGF......PADIW......
145482747	......EILNQ......IINLY......YLSME......ICHRD......IDFGY......SVDVW......
145484230	......QILRL......VLSIK......FLV--......IIHRD......ADFGL......KVDIY......
145488954	......LIQKK......ILKLC......FLILE......IVHRD......GDFGW......KTDIW......
145490261	......EILEK......IVKII......NIIIE......IAHRD......IDFGF......KSDVW......
145488705	......SITKL......IINLI......HIITE......IVHRD......IDFGL......SVDVF......
145490026	......AILNK......IIKLH......YLITE......IIHRD......LDFGL......KGDIF......
145489560	......SILKR......IIKLY......NLVME......ICHRD......IDFGF......ASDIW......
145486768	......LILQL......IIKLY......YLIQE......IIHRD......IDFGY......KVDVW......
145490361	......SILKS......IIKVL......WIVQE......IVHRD......IDFGF......NSDMF......
145487922	......SIMEN......IIKFY......YLVME......IIHRD......ADFGL......AVDMW......
145488946	......ALMKL......CIRLY......YLIME......ILHRD......CDFGV......ASDIW......
145488906	......QILYR......IVKIK......VIIHE......LLHRD......IDFGL......KSDIF......
145488211	......AILKK......VVRLV......YLIME......IVHRD......ADFGV......PADIW......
145487758	......YMLQK......IIKFH......YICMD......IVHRD......IDFGL......KCDIW......
145487967	......YVLRK......IIKLF......ILVTE......YVHRD......IDFGF......VGDVF......
145490076	......NIQSK......IVSMV......YMLLE......ILHRD......ADFTW......KLDIW......
145493838	......EILKK......IINLF......ILITE......YVHRD......IDFGF......VGDVF......
145495677	......EILEK......IVKIL......NIILE......IAHRD......IDFGF......KSDVW......
145491524	......NQMRY......ITRLI......YFVLE......ICQRD......IDLGF......KSEIW......
145492051	......QIWSM......VAKFY......YVVME......IIHRD......IDFGL......KSDIF......
145492949	......KILQS......IIKVL......WIVQE......IVHRD......IDFGF......NSDMF......
145491997	......EILEK......IVKAL......NMIIE......VVHRD......IDFGI......QIDIW......
145495238	......IMLQS......IIKIL......YIVMN......IVHRD......IDFGL......SVDIW......
145493160	......NVMRK......LINLH......YLILD......IIHRD......SDFGI......KCDVF......
145494434	......RQLKQ......IIKII......YFVME......IVHRD......ADFGF......EADIF......

```
145494438    ......RALKS......IVKIY......Y-IME......IIHRD......IDFGI......LVDVW......
145495667    ......SNLRK......ICYIV......YVLYE......VFHRD......GNFCY......KVDLF......
145492053    ......AIMKK......IVQLY......YLVLE......IAHMD......ADFGV......PIDVW......
145493075    ......EILEK......IVKIL......NIILE......IAHRD......IDFGF......KSDVW......
145491588    ......AILKK......VVRLV......YLIME......IVHRD......ADFGV......PADIW......
145495240    ......QLLNQ......IVRVT......FIMMD......IIHRD......IDFGL......SVDIW......
145493874    ......QIGSM......LIKVK......YQIME......VCHRD......LDFGV......KIDMW......
145495290    ......LIHQQ......IVNFI......YIITE......IAHRD......CDFDV......ECDIH......
145495575    ......QILQS......IIKVL......WIVQE......IVHRD......IDFGF......NSDMF......
145493051    ......SNLRK......ICYIV......YILYE......VFHRD......GNFCY......KVDLF......
145494991    ......EIMKL......IVRLV......YLVTE......IVHRD......IDFGF......KIDNF......
145495420    ......VVMRK......LINLH......YLILD......VIHRD......SDFGI......KCDVF......
145496485    ......KILSK......IIKLV......HVIME......VVHRD......IDFGF......PADVW......
145497220    ......YLLKS......IVKFY......VIVME......LIHRD......VDFGI......HIDVW......
145497355    ......SIMKK......IIHLY......FLVLE......ILHRD......GDFGL......KIDIY......
145499542    ......NILKQ......LVNLI......CIILE......ICHRD......ADFGF......KADIF......
145496525    ......FILQK......IIKFH......YICMD......IVHRD......IDFGL......KCDIW......
145498600    ......NALRI......ITKLY......NLVME......VCHRD......IDFGF......GADIW......
145499546    ......EVLRS......IVKVY......AVIME......VVHRD......IDFGI......SVDIW......
145497609    ......QNLKL......ILKLY......YIISE......LVHRD......IDWGF......KCDIW......
145497218    ......QILKQ......IVHLI......ILVME......LIHRD......VDFGI......LSDVW......
145496625    ......EIMKL......IIRFV......NIITE......IVHRD......IDFGF......KVDNF......
145497655    ......AINND......FIKLH......YMVMD......IMHRD......VDFGL......VCDIF......
145500233    ......SLLQE......IVKLY......YLIME......IIHRD......IDLGL......ALDIF......
145498299    ......LTLQT......IIQIL......YIVMN......IVHRD......IDFGL......SVDIW......
145499677    ......AIMKK......IVQLY......YLVLE......IAHMD......ADFGV......PIDVW......
145495920    ......EVLRS......IVKVF......AVIME......IVHRD......IDFGI......SVDIW......
145497697    ......QILKK......LVQLY......FLVME......IVHRD......IDFGL......QVDIW......
145504294    ......YILQK......IIKFH......YICMD......IVHRD......IDFGL......KCDIW......
145503890    ......INSQL......IAKCY......YLLME......VCHRD......IDFGV......QVDLW......
145501462    ......AIMKK......IIRLY......YMVME......IVHRD......CDFGV......KVDIW......
145504300    ......YMLQR......IIKFH......YICMD......IVHRD......IDFGL......KCDIW......
145502549    ......YILNT......IVQYY......YFLIE......IIHRD......IDFGL......SVDIW......
145501089    ......EVMKN......IIKII......VLIME......LVHRD......IDFGI......SIDIW......
145500706    ......KIHKQ......IIQLL......YLILE......IIHRD......CDFGL......SVDIW......
145502861    ......HILRK......IVQLY......YLIME......IAHRD......VDFGL......KTDIW......
145501532    ......SILRQ......VVHLY......YVVME......IMHRD......ADFGL......KVDVF......
145503692    ......SILKI......FLILR......YLVTE......IIHRD......VDFGL......KVDVF......
145502005    ......NINRT......IIRSY......YWVME......ICHRD......IDFGV......KLDVW......
145502265    ......LILKQ......IVKLY......ILIME......IVHCD......IDFGV......GQDVW......
145500632    ......AILRK......VIKLY......YLVME......IVHRD......ADFGL......KSDIW......
145501667    ......QLLIK......IVRVS......FITMD......IIHRD......IDFGL......SIDIW......
145504418    ......SIMES......IIKFY......YLIME......IIHRD......ADFGL......AVDMW......
145500452    ......HILKL......IIKYV......NLIVE......IVHRD......IDFGF......PVDVW......
145501705    ......KILQS......IQKI-......WFITE......YVHRD......IDFGL......KSDVF......
145500574    ......ELMRI......VVTIL......FIVQE......IIHRD......ADFGL......KVDIY......
145500744    ......RILQM......IINIY......NLLLE......ITHRD......IDFGF......PADIW......
145503694    ......NYLQG......IVEYI......YIVLE......IVHRD......CDFNW......GVDIW......
145508898    ......TILRS......ILKLY......SLVTE......IIHRD......IDFGL......KVDVF......
145507280    ......EIMKL......IVRFH......NIITE......IVHRD......IDFGF......KVDNF......
145505603    ......QILKV......IVKLY......YLFME......IIHRD......ADFGL......MTDVW......
145507700    ......QTLRM......ILKLY......CLLTE......VMHRD......IDFGL......QCDIF......
145505830    ......KILSK......IIKLV......HVIME......VVHRD......IDFGF......PADVW......
145505668    ......SIMEN......IIKFY......YLVME......IIHRD......ADFGL......AVDMW......
145505491    ......QTLRM......ILKLY......CLLTD......VMHRD......IDFGL......QCDIF......
145507224    ......HILKL......VIQLY......FLVME......IVHRD......VDFGL......LVDIW......
145507528    ......HILKL......IIQLY......FLVME......VVHRD......VDFGL......KVDLW......
145507794    ......QILKI......IVKLY......YLFME......VIHRD......ADFGL......QTDIW......
145505335    ......HILKL......IIQLY......FLVME......VVHRD......VDYGL......KVDLW......
145505071    ......EIMKL......IVRFH......NIITE......IVHRD......IDFGF......KVDNF......
145508493    ......INSTI......IAKCY......YMLME......ICHRD......IDFGV......QIDLW......
145505664    ......EIMKL......IIRFV......NIITE......IVHRD......IDFGF......KVDNF......
145505027    ......HILKL......VIQLY......FLVME......IVHRD......VDFGL......LVDIW......
145509066    ......HILKL......VIQLY......FLVME......IVHRD......VDFGL......LVDIW......
145511592    ......NNNRC......IAQDI......YLIME......ICHRD......IDFGV......QVDIW......
145513448    ......EINNQ......FVKIK......YIVMD......IIHRD......VDFGL......ACDIF......
145511381    ......KILNS......IISIL......NWYTT......YVHRD......IDFGL......KTDVF......
145510130    ......QILRR......IQKLL......YILFD......IIHKD......ANFAS......KIDIF......
145509851    ......TIMKI......FAQLY......YIIME......ILHRD......CDFGV......ASDIW......
145509573    ......SHQAE......IIKII......YFVME......IVHRD......ADFGF......QIQLV......
```

```
145513196 ......NQLKN......VIKIY......HIVID......LCHRD......VDFGF......KCDIW......
145513208 ......EIMKQ......ICKML......YLVMD......LCHRD......IDFGV......QIDVW......
145510951 ......NIQSK......IVSMV......YMLLE......VLHRD......ADFTW......KLDIW......
145513420 ......SLLKN......IVKLF......YLVTE......IIHRD......IDFGT......KCDIW......
145510312 ......MILRK......VIKLY......YLVME......ITHRD......ADFGL......KSDIW......
145510999 ......AILHK......IIKLH......GLITE......IIHRD......LDFGL......KGDIF......
145509579 ......RALKS......IVKIY......Y-IME......IIHRD......IDFGI......LVDVW......
145513500 ......KILRA......ILSIQ......SIITD......IIHRD......IDFGL......KVDVF......
145514289 ......RIMNK......ISGVI......CIMMD......VAHRD......CDFGF......EADLF......
145514754 ......EILKK......IVKFL.....-IVLE......IFHRD......IDFGL......KADIW......
145514910 ......RALKS......IVKIY......Y-IME......IIHRD......IDFGI......LVDVW......
145513933 ......IFGDA......IVKIY......VYIME......IIHGD......ICFGI......QVDVW......
145516889 ......NNTRC......IAQDI......YLIME......ICHRD......VDFGV......EVDIW......
145516727 ......KINRQ......VIKAI......HLVME......VCHRD......IDFGV......KVDIW......
145516909 ......HILKI......IIMLY......FIVME......IVHRD......VDFGL......KADIW......
145516671 ......KSMFK......IVKVH......HVVME......ICHRD......TDFNV......MVDMW......
145517522 ......ELLRL......VVTIQ......YIVQE......ILHRD......ADFGL......KIDVY......
145517476 ......SILKK......IIKLY......YLVME......IAHRD......ADFGL......KSDIW......
145514191 ......TLMKI......FASLF......YIIME......ILHRD......CDFGV......ASDIW......
145517520 ......HILRQ......IIQLH......YLVFD......IFHRD......ADLGL......KVDIY......
145516613 ......NNTRC......IIELY......YLVME......ICHRD......VDFGV......EVDVW......
145514067 ......EILRK......IVKFL.....-IVLE......IYHRD......IDFGL......KADIW......
145515988 ......EILKK......IIQIY......YLIME......IVHRD......VDFGL......LIDIW......
145514522 ......RIMNK......ISGVI......CIMMD......VAHRD......CDFGF......EADLF......
145514628 ......TLMKI......FASLF......YIIME......ILHRD......CDFGV......ASDIW......
145520709 ......TIIKS......IAKCY......YMLME......ICHRD......IDFGV......QVDIW......
145519171 ......EILKK......IIQIY......YLIME......IVHRD......VDFGL......LIDIW......
145519778 ......EILSR......IIRVY......YFVME......IIHRD......IDFGL......TCDEW......
145519107 ......ELLNK......IVKMK......YLITE......IIHRD......IDFGL......KTDIW......
145521588 ......KILNL......IINLI......CMVLE......ICHRD......ADFGF......KADIF......
145521138 ......NLLKA......IVKLY......YLIME......IIHRD......IDLGL......AVDIF......
145518626 ......ELLRV......IINIQ......YIVHD......ILHRD......ADFGL......KVDVY......
145519792 ......EILSK......IVKAI......NMILE......VVHRD......IDFGI......QIDIW......
145520134 ......SILKR......IIKLY......NLVME......ICHRD......IDFGF......SSDIW......
145518450 ......SIMRK......VIKMY......YLVLE......ILHRD......CDFGL......KVDVY......
145521454 ......SILRK......LIKLH......YLVQD......ILHRD......TDFGL......KVDIF......
145519525 ......SALKA......IVKLL......NLVLE......ICHRD......IDFGF......TTDIW......
145521428 ......SILRK......VVQLF......YIVTE......VVHRD......VDFGL......FTDIW......
145523475 ......AIMKK......LIQLF......FMVLE......VVHRD......ADFGV......SADIW......
145524333 ......EILKK......IIQIY......YLIME......IVHRD......VDFGL......LIDIW......
145523389 ......RLLRY......VIKLY......FVVME......VAHRD......ADFGL......DADVW......
145523860 ......HILRK......VVQLY......YLIME......VAHRD......VDFGL......KTDIW......
145524647 ......HINRA......VVRAL......HLVME......ICHRD......IDFGV......KVDIW......
145524098 ......TILST......IVQYY......YFLIE......IIHRD......IDFGL......SVDIW......
145524006 ......RYMRH......VIKLY......FVVME......VAHRD......GDFGL......EADVW......
145522952 ......QILRR......IQKLL......YILFD......IIHKD......ANFAS......KIDIF......
145527480 ......AINNE......FIKLH......YMVMD......IMHRD......VDFGL......VCDIF......
145526555 ......NILRK......IVRLF......YLIIE......IVHRD......IDFGT......KCDIW......
145526841 ......HILKL......IIKYV......NLVVE......IVHRD......IDFGF......PVDVW......
145527094 ......QILRT......ISNLV......FLLQE......IFHRD......IDFSL......NIDVY......
145526128 ......HILKI......IIMLY......FIVME......IVHRD......VDFGL......KADIW......
145527418 ......QNLKL......ILKLY......YIISE......IVHRD......IDWGF......KCDIW......
145526559 ......RILQM......IIQIY......NLLIE......ITHRD......IDFVF......PADIW......
145526300 ......KINRQ......VIKAI......HFVME......VCHRD......IDFGV......KVDIW......
145526719 ......ELMRI......VVSIL......FLIQQ......IIHRD......ADFGL......KVDIY......
145527530 ......QILKK......LVQLY......FLVME......IVHRD......IDFGL......HIDIW......
145528349 ......SALKA......IVKLL......NLVLE......ICHRD......IDFGF......TTDIW......
145526386 ......NNTRC......IIELY......HLVME......ICHRD......VDFGV......EVDIW......
145526725 ......EISQE......LIKIF......FIFMR......IIHRD......GDFDC......EYDIF......
145527935 ......ELLNK......IVKMK......YLITE......IIHRD......IDFGL......KTDIW......
145526667 ......TILRK......VIKLY......YLVME......IVHRD......ADFGL......KSDIW......
145526505 ......KIQQT......IVAMV......YMMLE......ILHRD......ADFTW......KVDIW......
145531062 ......QIHRG......LIKLL......CLVYD......LAHRD......SDLGF......SVDIF......
145530483 ......AIMKK......LIQLY......FMVLE......IVHRD......ADFGV......AADIW......
145528698 ......DIMKL......IVRSV......YLVTE......IVHRD......IDFGF......KIDNF......
145529900 ......TIIKS......IAKCY......YMVME......ICHRD......IDFGV......QVDIW......
145530624 ......KILQS......IQKIV......WFITE......YVHRD......IDFGL......KSDVF......
145530960 ......VILEQ......IVKLY......VLELE......VIHRD......IDFGL......SVDIW......
145531701 ......EILSK......IVKAI......NMIIE......VVHRD......IDFGI......QIDIW......
145533066 ......QILKQ......LVQLY......FLVME......IVHRD......IDFGL......QVDIW......
```

145533945	......EIMKQ......ICKML......YLVME......LCHRD......IDFGV......KIDVW......
145533741	......NLLKN......IVKLY......YLVTE......IIHRD......IDFGT......KCDIW......
145534764	......NIQSK......VVAMV......YMLLE......ILHRD......ADFTW......KLDIW......
145533376	......EIHPT......IVKVK......-IVLE......YVHMD......GDFGN......KMDIW......
145533126	......SINNT......FIKLH......YMVMD......IMHRD......VDFGL......VCDVF......
145531149	......SLLQE......IVKLY......YLIME......IIHRD......IDLGL......ALDIF......
145532819	......NCLKK......IIQIY......YLIME......VMHRD......VDFGL......KADTW......
145533683	......KILRT......ILSFQ......SIITE......IMHRD......IDFGL......KVDVF......
145531603	......HILMI......IVKLI......YLVLE......IAHRD......NDFGL......KADIW......
145532795	......HILKL......VIQLY......FIVME......IVHRD......VDFGL......MVDIW......
145536057	......TILQM......VVQLY......YLFME......IVHRD......VDFGL......QTDLW......
145536484	......NYLQC......IVEFI......YIVLE......IVHRD......CDFNW......GVDVW......
145535738	......HILKL......VIQLY......FLVME......IVHRD......VDFGL......LVDIW......
145537217	......SILRK......VIKLY......YLVME......ITHRD......ADFGL......KSDIW......
145535311	......NNTRC......IIELY......FLVME......ICHRD......VDFGV......EVDIW......
145535458	......AILKQ......IVKIH......YLMLN......ISHRD......IDFGF......AADIW......
145537950	......FLNTK......IVRTY......YLVME......LCHRD......IDFGV......LIDVW......
145537700	......EINNL......FVKIK......YIIMD......VMHRD......VDFGL......KCDIF......
145539003	......EVMKN......IVKIL......VLIME......LIHRD......IDFGI......SIDIW......
145540840	......EINNL......FVKIK......YIIMD......IMHRD......VDFGL......KCDIF......
145542436	......EVMKN......IIKIL......VLIME......LIHRD......IDFGI......AIDIW......
145539786	......FLNTS......ICKML......YLVME......LCHRD......IDFGV......KIDIW......
145541578	......SIHRR......AIKFY......FIILE......IIYRD......RKFSQ......KVDVF......
145542033	......QILKQ......IVHLI......ILVME......LIHRD......VDFGI......LSDVW......
145542035	......YLLKS......IVKFY......VIVME......LIHRD......VDFGI......HIDVW......
145541636	......TILQM......VVQLY......YLFME......IVHRD......VDFGL......QTDLW......
145538544	......LILKQ......IVRLY......IMVME......IIHCD......IDFGV......GQDVW......
145540601	......QAMVA......IAELL......YMVMQ......YMHRD......IDFGL......KCDIF......
145539938	......AIMKK......VVQLF......YLVME......VIHRD......ADFGV......PVDIW......
145541487	......AIMKK......LIQLY......TLVLE......VVHRD......ADFGV......AADIW......
145543184	......IINRE......IIQVF......YLVME......ICHRD......IDFGV......KIDIW......
145543657	......DILNL......IINLY......FLIME......ISHRD......IDFGY......SVDVW......
145543278	......STQAE......IIRII......YFVME......VVHRD......ADFGF......EADIF......
145544655	......HILKL......IIQLY......FLVME......VVHRD......VDFGL......QVDLW......
145546963	......NCLKK......IIQIY......YLIME......VMHRD......VDFGL......KADTW......
145545408	......NTLRI......ITKLY......NLVME......VCHRD......IDFGF......AADIW......
145546929	......HILKL......VIQLY......FIVME......IVHRD......VDFGL......MVDIW......
145545009	......HILKL......VIQLY......FLVME......IVHRD......VDFGL......LVDIW......
145548160	......RIQSK......IVELV......YLVQE......IVHRD......TDFNW......KVDSW......
145545059	......QILRQ......IIRLI......NLIME......IVHRD......IDFGF......ITDIW......
145545305	......NIHKY......ICKLY......YLIMQ......IVNRD......IDFST......KLDVW......
145547046	......--LQQ......IVKVF......YYLME......IIYRD......VDFDL......SVDYW......
145544048	......RILKI......FLNLK......YLVTD......IIHRD......VDFGL......KVDIF......
145547314	......EINNQ......FIKVK......YIVMD......IMHRD......VDFGL......VCDIF......
145544046	......NYLQC......IVEFI......YIVLE......IVHRD......CDFNW......GVDVW......
145545895	......SINND......FIKLH......YMVMD......IMHRD......VDFGL......VCDVF......
145544450	......NILKL......IIKLI......CIILE......ICHRD......ADFGL......KADIY......
145545584	......EILEK......IVKII......NIILE......IAHRD......IDFGF......KSDVW......
145548938	......SIMRK......LIKMH......FLVQD......ILHRD......ADFGL......KVDIF......
145550108	......QILRS......LVEIY......QIVTE......ILHRD......IDFGL......KVDVF......
145548866	......KILRL......VMTLI......FVVQE......VIHRD......SDFGL......KVDVF......
145550744	......SILKR......IIKLV......NLVME......IAHRD......IDFGF......CSDIW......
145553283	......SILKK......VIKLY......YLVME......ITHRD......ADFGL......KSDIW......
145552457	......EIHQR......IIKLH......YVVLE......VAHRD......CDFGS......LADVW......
145551109	......SILKR......IIKLI......NLVME......IAHRD......IDFGF......CSDIW......
145548852	......HILRK......IIQLY......YLITE......IVHRD......VDFGL......KTDIW......
145549245	......AIMKK......VVQLY......YLVME......VIHRD......ADFGV......PVDIW......
145550531	......IINRE......IIQVF......YLVME......ICHRD......IDFGV......KIDIW......
145550702	......----Q......IVRCK......YIIME......IIHRD......ADFGL......QVDAW......
145551061	......----Q......IVRCK......YIIME......IIHRD......ADFGL......QVDAW......
145551548	......EILNQ......IINLY......YLIME......ICHRD......IDFGY......SVDIW......
145551616	......KILKS......IIKIV......YFVCE......YVHRD......IDFGF......KSDIF......
145550435	......STQAE......IIRII......YFVME......VVHRD......ADFGF......EADIF......
145551943	......SINNE......FIKLH......YMVMD......IMHRD......VDFGL......VCDVF......
145473691	......LILSQ......IVRYH......YFILS......IIHRD......IDFGL......SVDIW......
145552701	......KNLKK......IVKME......YVVME......CCHRD......TDFNV......QVDLW......
145549962	......YTLKK......IIKFY......YICME......IVHRD......IDFGF......KCDIW......
124603212	......RTTAM......IASVH......YLVME......LVHRD......TDFGI......ASDVY......
125525490	......GTLKL......VVRLH......YMVLE......VYHRD......SDFGL......LSDIW......

```
125526301    ......--HKM......-----......FIILE......VYHRD......SDFGL......LADTW......
125527780    ......DVMRR......VIRLH......YFVME......VYHRD......TDFGL......KADIW......
125528214    ......SIMRL......VLQLF......YFVLE......VYHRD......SDFGL......KADVW......
125524845    ......AVLRR......IVQLY......YFVME......VFHRD......SDFGL......AADLW......
125539851    ......INHRS......IIRFK......AIVME......ICHRD......CDFGY......MADVW......
125537205    ......INHRS......IIRFK......AIVME......VCHRD......CDFGY......IADVW......
125537309    ......AAMRR......VLRLH......YLVME......VAHRD......SDFGL......KADAW......
125535689    ......STMKL......VVRIY......YIVLE......VYHRD......SDFGL......MADLW......
125538316    ......TAMKL......IVKIY......CLVME......VYHRD......SDFGL......AADVW......
125538180    ......SIMRL......ILQLF......YFVLE......VYHRD......SDFGL......KADIW......
125535596    ......TTMRL......IVQLH......YFVME......VYHRD......SDFGL......KSDIW......
125533281    ......STMKL......VVRIY......YIVLE......VYHRD......SDFGL......MADLW......
125550758    ......ATLKL......VVRLH......YMVLE......VYHRD......SDFGL......LSDIW......
125541541    ......AEQEP......VVALH......HLVVD......VAHRD......GDFGS......KVDVW......
125544162    ......MNHRS......IIRF-......-----......VCHRD......CDFGY......KVDVW......
125545822    ......INHRS......IIRFK......AIVME......VCHRD......CDFGY......TADVW......
125544980    ......AAMRR......VLRLH......YLVME......VSHRD......SDFGL......KADAW......
125542218    ......STMKL......VVQLH......YMVLE......VYHRD......SDFGL......AADVW......
125543650    ......CTMKL......VVRLF......FIVLE......VYHRD......SDFGL......AADIW......
125545469    ......SIMKM......IVNLI......YMVLE......IIHGD......GDFSV......AADTW......
125541539    ......AEQEP......VVALH......HLVVD......VAHRD......GDFGS......KVDVW......
125559305    ......ATLKL......VVRLH......YMVLE......VYHRD......SDFGL......SSD--......
125551861    ......AALRR......VVALL......YLVLE......VFHRD......TDFGL......KADVW......
125555659    ......AVMQR......VVRIH......CVVME......VFHRD......ADFGL......KADVW......
125552972    ......AILRR......IVRLF......YFVME......VFHRD......SDFGL......KADIW......
125551857    ......SVMKL......IVQLY......YFVLE......VYHRD......TDFGL......KVDTW......
125552707    ......SIMRL......VLQLF......YFALE......VYHRD......SDFGL......KADVW......
125555994    ......SIMKI......IVRLN......YIILE......VYHRD......SDFGL......AADVW......
125553081    ......KILRL......IIRLY......YVVME......VVHRD......ADFGL......EVDVW......
125557252    ......STMKL......VIRMH......YIVME......VYHRD......SDFGL......KADLW......
125559213    ......MNHRS......IVRFK......AIVME......ICHRD......CDFGY......KVDVW......
125570705    ......--HKM......-----......FIILE......VYHRD......SDFGL......LADTW......
125563737    ......SIMRM......VVGIR......FVVME......VAHRD......TDFGL......RADLW......
125563738    ......AVMKM......VVELH......YLALE......VYHRD......ADFGL......EADLW......
125561684    ......AVMKR......IVELH......YLALE......VYHRD......VDFGL......KADLW......
125570014    ......GTLKL......VVRLH......YMVLE......VYHRD......SDFGL......LSDIW......
125576106    ......STMKL......VVRIY......YIVLE......VYHRD......SDFGL......MADLW......
125572474    ......SIMRL......VLQLF......YFVLE......VYHRD......SDFGL......KADVW......
125579889    ......INHRS......IIRFK......AIVME......VCHRD......CDFGY......IADVW......
125578411    ......STMKL......VVRIY......YIVLE......VYHRD......SDFGL......MADLW......
125572094    ......NVLRR......IVQLF......YFVME......VFHRD......SDFGL......KADIW......
125582475    ......INHRS......IIRFK......AIVME......ICHRD......CDFGY......MADVW......
125586068    ......CTMKL......VVRLF......FIVLE......VYHRD......SDFGL......AADIW......
125586513    ......MNHRS......IIRF-......-----......VCHRD......CDFGY......KVDVW......
125581021    ......TAMKL......IVKIY......CLVME......VYHRD......SDFGL......AADVW......
125597500    ......AVMQR......VVRIH......CVVME......VFHRD......ADFGL......KADVW......
125592732    ......ATLKL......VVRLH......YMVLE......VYHRD......SDFGL......LSDIW......
125593800    ......-----......-CRSP......V----......VFHRD......TDFGL......KADVW......
125594865    ......AILRR......-----......YFVME......VFHRD......SDFGL......KADIW......
125599132    ......STMKL......VIRMH......YIVME......VYHRD......SDFGL......KADLW......
125594609    ......SIMRL......VLQLF......YFALE......VYHRD......SDFGL......KADVW......
125594976    ......KILRL......IIRLY......YVVME......VVHRD......ADFGL......EVDVW......
125603558    ......AVMKR......IVELH......YLALE......VYHRD......VDFGL......KADLW......
125601123    ......MNHRS......IVRFK......AIVME......ICHRD......CDFGY......KVDVW......
133901970    ......AIMKL......VLHLY......YLLLE......ICHRD......ADFGM......KADVW......
112253339    ......EILKS......VVRMF......FIILE......IIHRD......TDFGL......SSDLW......
112253341    ......EILKS......VVRMF......FIILE......IIHRD......TDFGL......SSELV......
125803911    ......AMMLK......IIGLH......ILSLE......VFHGD......IDFGC......PVNVW......
125803916    ......AMMLK......IIELH......ILSLE......VFHGD......IDFGC......PVNIW......
125804406    ......AMMLR......IIRLH......VLILE......VFHGD......IDFGC......PAYVW......
125804398    ......AMMLR......IIRLH......VLILE......VFHGD......IDFGC......PAYVW......
125804408    ......AMMLR......IIRLH......VLILE......VFHGD......IDFGC......PAYVW......
125805030    ......AMMLR......IIRLH......VLILE......VFHGD......IDFGC......PAYVW......
125805602    ......SLNLL......IVHMI......ILIME......IFHRD......IDFGL......PTTAW......
125807099    ......AILVR......VVKVL......QLVME......VLHRD......IDFGS......ELEMW......
125809357    ......SLNLL......IVHMI......ILIME......IFHRD......IDFGL......PTTAW......
125812670    ......AILKL......VLKLY......YLVLE......ICHRD......ADFGM......RADVW......
160333355    ......QNLKL......IIKLY......FMVME......VVHRD......ADFGL......EVDIW......
125816670    ......RIMKT......IVQLF......YLVME......IVHRD......ADFGF......EVDIW......
125817290    ......EIMKL......IIKLY......YLVTE......IVHRD......ADFGF......QLDIW......
125818500    ......ALNLL......VAHMI......ILIME......VFHRD......IDFGL......PTTVW......
125821350    ......RIMKL......IVKLF......YLVME......IVHRD......ADFGF......EVDVW......
125824929    ......GLMYL......IIQLL......TLVLE......VYHSD......IDFGV......PATVY......
125827075    ......AVMLK......IIGLH......ILSLE......VFHGD......IDFGC......PVNVW......
```

```
125827087    ......AVMLK......IIGLH......ILSLE......VFHGD......IDFGC......PVNVW......
125827089    ......AMMLK......IIGLH......ILSLE......VFHGD......IDFGC......PVNVW......
125828688    ......AMMLR......IIGLH......LLVLE......VFHGD......IDFGC......PANIW......
125828684    ......AMMLR......IIGLH......LLVLE......VFHGD......IDFGC......PANIW......
125828695    ......AMMLK......IIGLH......ILSLE......VFHGD......IDFGC......PVNVW......
125828698    ......AMMLK......IIGLH......ILSLE......VFHGD......IDFGC......PVNVW......
125828690    ......AMMLR......IIGLH......LLVLE......VFHGD......IDFGC......PANIW......
125828894    ......MCMEK......IVRLY......YLAME......IIHRD......GDFGF......YVDVW......
125830917    ......AMMLK......IIELY......VLILE......VFHGD......IDFGC......PAYVW......
125831040    ......AMMLK......IIELY......ILSLE......VFHGD......IDFGC......PAYVW......
125833241    ......AMMLR......IIRLH......VLILE......VFHGD......IDFGC......PAYVW......
125835205    ......AILKL......VLKLH......YLVLE......ICHRD......ADFGM......KADVW......
125835977    ......ALMLK......VIEMY......SIVME......IVHND......IDFG-......VMDTR......
125835979    ......AFMLK......VIEMY......SIVME......IVHND......IDFGC......AVSAW......
125837648    ......QIMKM......IIKLY......YLVTE......IVHRD......ADFGF......QLDIW......
125837830    ......RIMKG......IVQLF......YLVME......IVHRD......ADFGF......EVDIW......
125838924    ......ALLRK......IVQMI......ILIME......IFHKD......IDFGC......KEAIC......
125838960    ......ALLRK......IVQMI......ILIME......IFHKD......IDFGC......KEAIC......
125840037    ......QNLKL......IIKLY......FMVME......VVHRD......ADFGL......EVDIW......
125840275    ......AMMLR......IIRLH......VLILE......VFHGD......IDFGC......PAYVW......
125840283    ......AMMLR......IIRLH......VLILE......VFHGD......IDFGC......PAYVW......
125840447    ......AMMLR......IIRLH......VLILE......VFHGD......IDFGC......PAYVW......
125840499    ......AMMLR......IIRLH......VLILE......VFHGD......IDFGC......PAYVW......
125843764    ......AMMLK......IIGLH......ILSLE......VFHGD......IDFGC......PVNVW......
125843762    ......AMMLK......IIGLH......ILSLE......VFHGD......IDFGC......PVNVW......
125843928    ......VLTLM......IIQLL......VMIFE......VFHRD......IDFGC......PATVW......
125846224    ......AMMLR......IIGLH......YLILE......VFHGD......IDFGC......PAYVW......
125847919    ......SNMES......LLQLF......YLILE......IIHRD......ADFGF......PVDVW......
125848492    ......RIMKL......IVKLF......YLVME......IVHRD......ADFGF......EVDVW......
125849288    ......EIMSS......IISIY......VIVME......IVHRD......ADFGL......EVDSW......
125851181    ......AYMLI......LIELY......SLVME......ISHND......IDFGC......YVDVF......
125851179    ......AYMLM......LIELY......SLVME......ISHND......IDFGC......YVDIR......
125851582    ......EITSS......IIRLY......VMVME......VVHRD......ADFGL......EVDCW......
125854436    ......EIMSS......IISIY......VIVME......VVHRD......ADFGL......EVDSW......
125854815    ......AMMLR......IIRLH......VLVLE......VFHGD......IDFGC......PAYVW......
125854813    ......AMMLR......IIRLH......VLVLE......VFHGD......IDFGC......PAYVW......
125854811    ......AMMLR......IIRLH......VLILE......VFHGD......IDFGC......PAYVW......
125854912    ......AMMLR......IIRLH......VLILE......VFHGD......IDFGC......PAYVW......
125855542    ......AMMLR......IIRLH......VLILE......VFHGD......IDFGC......PAYVW......
125855808    ......ELMLM......VIEMY......SMVLE......IVHND......IDFG-......NLDTR......
125597803    ......SIMKI......IVRLN......YIILE......VYHRD......SDFGL......AADVW......
126138794    ......CISSS......IIDTL......CEVME......VCHRD......TDFGN......RVDIW......
126134373    ......ALHLK......VITIH......VILMD......VYHCD......IDFGL......AGDIW......
126649093    ......AVLKS......IIKIF......YIIME......IVHKD......IDFGL......ECDVW......
126649203    ......SLSEP......FPKIF......YIVME......IMHGD......LDFGL......-FDFW......
126654147    ......EIMKL......IVSII......KMVLE......IIHRD......ADFGL......EIDIW......
126644132    ......C-LKL......VMKIY......YLVME......MLHRD......VDFDT......ASDLW......
126649261    ......SILKS......IIRLY......YIIME......ICHRD......GDFGL......EIDVW......
126654507    ......KILQF......IAKLH......YLIFD......IVHRD......NDFNS......PADMW......
126650670    ......LIMRK......IPRVL......VLVHE......IVHRD......SDFNS......KRDIW......
126438857    ......ALCAR......IVALL......FAVFE......VVHRD......LDFGI......KSDFY......
126290438    ......RIMKI......IVKLF......YLIME......IVHRD......ADFGF......EVDVW......
126307104    ......RIMKI......IVKLF......YLVME......IVHRD......ADFGF......EVDVW......
126310559    ......RIQQM......IVQLY......YMVME......IVHRD......VDFGL......KVDVW......
126305549    ......-----......INQIT......YVFFE......LVLRD......EDAYI......AADVW......
126306879    ......EIMSS......IITIH......VIVME......IVHRD......ADFGL......EVDSW......
126305942    ......QNLKL......IIKLY......FMVME......VVHRD......ADFGL......EVDIW......
126321542    ......QNLKL......IIKLY......FMVME......VVHRD......ADFGL......EVDIW......
126321540    ......QNLKL......IIKLY......FMVME......VVHRD......ADFGL......EVDIW......
126322672    ......QLPS-......ITGIV......YVFFE......IVLGD......EDTHI......SADVW......
126323665    ......QLLRR......VIQLV......YMVME......IVHKD......SDLGV......KVDIW......
126325221    ......QIMKL......IIKLY......YIVTE......IVHRD......ADFGF......HLDIW......
126325273    ......QIQQM......ITQLL......YLVME......VVHRD......IDFGL......KIDVW......
126326660    ......QIMKM......IIKLY......YLVTE......IVHRD......ADFGF......QLDIW......
126326980    ......QIMKM......IIRLY......YLVTE......IVHRD......ADFGF......KVDIW......
126327255    ......CINKM......IVKFY......YLFLE......ITHRD......SDFGL......PVDVW......
126333689    ......RIMKV......IVKLF......YLVME......IVHRD......ADFGF......EVDVW......
126334070    ......DAMKN......ICRLY......FMVME......YAHRD......IDFGL......EADVW......
126338547    ......AILSQ......IIKVV......QLVME......ITHRD......VDFGS......ELEMW......
126339782    ......EIMSS......IISIY......VIIME......VVHRD......ADFGL......EVDSW......
126341479    ......RCMKL......IVRLY......YLILE......VVHRD......TDFGF......AVDIW......
126658984    ......VILED......IPQLY......YLVQE......CIHRD......LDFGA......ATDLY......
126658230    ......ETLGK......VPRLL......YLVQE......VIHRD......IDFGV......ASDIY......
```

126632511	......AMMLK......IIGLH......ILSLE......VFHGD......IDFGC......PVNVW......
126732835	......RTLRR......IVRYY......FLVME......VIHRD......IDFGI......ATDIY......
126544477	......QNLKL......IIKLY......FMVME......VVHRD......ADFGL......EVDIW......
146324560	......QLLRE......LMRMA......CLVMD......WVHRD......SDFGL......GVDVW......
139438755	......HTAAM......IVQVI......YLVME......VLHLD......TDFGM......RADVF......
133900707	......DALRN......ICRLY......FIVME......YAHRD......IDFGL......EADVW......
133900709	......DALRN......ICRLY......FIVME......YAHRD......IDFGL......EADVW......
134102348	......RTAGR......VVTVH......YIVME......IVHRD......ADFGI......AGDLW......
134103121	......RIAAR......AIVVF......VLVME......IVHRD......TDFGI......ASDVF......
134100628	......RALAA......CVHVY......AIVME......VLHRD......IDFGI......ASDAW......
134103120	......RIAAR......AISVY......VLVME......VVHRD......TDFGI......ASDVF......
134100369	......RSLAV......VVMLY......FVVME......IVHRD......GDFGI......AADLW......
134103045	......RILAR......APTLY......FQVMQ......ICHRD......LDFGL......RSDLY......
134096663	......RTTAS......IAAVH......YLVME......LVHRD......TDFGI......ASDVY......
134025733	......DAMKN......VCRLY......FMVLE......YAHRD......IDFGL......EADIW......
145230860	......SILRD......IVRLH......GIIME......IVHRD......TDFGF......KVDVW......
145235409	......QYLQL......IIKLY......VMVLE......IVHRD......ADFGL......EVDVW......
154332023	......HALRQ......CVRFI......VIVTE......IVHRD......VDFGL......RLDVY......
154332434	......AVMKM......IIELH......YLVLE......IAHRD......SDFGL......KADIW......
154338437	......AVMKF......VTKLK......YIIME......LYHRD......ADFGT......VVDSW......
154345129	......DVLSR......RLLLP......VIGME......CIHRD......IDFGL......AADVW......
154337523	......SVLRR......IVQLI......YIILE......VAHRD......SDFGL......RADIW......
154341178	......EILQL......ICRLY......YLIME......VVHRD......IDFGL......EVDVW......
154336062	......DALRR......VVQLH......YIVLQ......VVHRD......ADFGY......RMDIF......
154335515	......AIMRS......VVKLQ......YLVLE......FAHRD......SDFGL......SADIW......
154335070	......ETMSL......IVKLE......SIVME......IVHRD......ADFGL......PVDMW......
154344044	......EMMRK......VVRLL......NLVME......IVHRD......CDFGL......SCDMW......
154338129	......TILRS......VVRVV......ALVME......IIHCD......ADFGS......PVDVW......
154344445	......MVMQA......VVKFY......YFMME......VAHKD......CDFGF......--DLW......
154340583	......NALEI......VTGLV......YIVMD......VVHRD......SDFGF......KIDVW......
154340126	......TLAKS......LVRVF......SIVME......VMHRD......GDFGF......KCDVW......
154335810	......DVLRH......VVTFL......LLFFE......IIHRD......TDFGL......NADTW......
154344789	......AIMKK......CVSLY......YLIME......LVHHD......TDFGV......HTDVW......
146078894	......AVMKF......VVKLK......YIIME......LYHRD......ADFGT......VVDSW......
146101167	......AIMKK......CVSLY......YLIME......LVHHD......SDFGV......HTDVW......
146103233	......KIMRV......ITRLY......VLAME......VTHRD......SDFGL......EADVW......
146093233	......EILQL......ICRLY......YLIME......VVHRD......IDFGL......EVDVW......
146084842	......DTLRR......VVQLH......YIVLQ......VVHRD......ADFGY......RMDIF......
146076711	......HVLRQ......CVRFI......VIVTE......IVHRD......VDFGL......RLDVY......
146083145	......ETMSL......IVKLE......SIVME......IVHRD......SDFGL......PVDMW......
146100450	......MVMQA......VVKFY......YFVME......VAHKD......CDFGF......--DLW......
146097996	......EMMRK......VVRLL......NLVME......IVHRD......CDFGL......SCDMW......
146085419	......ALCRR......VVPMY......VIALP......VVHRD......LDFGL......ACDVW......
146102485	......TLMSH......IVKFY......YVVME......CIHRD......TDFGL......EVDTW......
146086611	......SILRR......IVQLI......YIILE......VAHRD......SDFGL......RADIW......
146101817	......DVLSR......RLLLP......VIGME......CIHRD......IDFGL......AADVW......
146092827	......DVLRH......VVTFL......LLFFE......IIHRD......TDFGL......NADTW......
146077578	......AVMKM......IIELH......YLVLE......IAHRD......SDFGL......KADIW......
154346474	......TLMSH......IVKFY......YVVME......CIHRD......TDFGL......EVDTW......
154345576	......KIMRV......ITRLY......VLVME......VTHRD......SDFGL......EADVW......
145237908	......IVARH......VVKTV......SIVME......IAHRD......TDFGF......RLDVW......
145238296	......HVLNY......IVEME......YIEMT......VVHRD......IDFGS......EQDIW......
145239611	......AIVSL......ICGMR......YMLFE......IVHRD......IDFGL......EVDVW......
145239767	......RVWRY......VLTLD......FCFTK......VVHRD......CDFGM......SVDMW......
145240979	......TVHKH......IISFF......WIAME......VGHRD......ADFGL......DVDIW......
145249546	......YLLLL......LDSVY......YSETE......LCHTD......IDLGL......TVQIW......
145250705	......KIMKD......IVQYV......YIIME......ITHRD......SDFGL......SVDMW......
145252496	......KLHHL......VVSLV......YVVIE......IYHRD......ADFGL......PNDVW......
145253669	......DCHRR......IVNLI......YLVLE......MYHRD......GDLGL......KADIW......
145253817	......CISSS......VIHTL......CEVME......VAHRD......TDFGN......AVDVW......
145255768	......HHHRQ......IARLY......WLVLE......CVHRD......CDFGF......KVDVW......
145254774	......CIGST......IIETL......YEVME......LAHRD......IDFGS......PTDVW......
146092235	......NALEI......VTGLV......YLVME......VVHRD......SDFGF......KVDVW......
134279930	......ALCAR......IVALL......FAVFE......VVHRD......LDFGI......KSDFY......
138894697	......QAATS......IVSIY......YIVME......IIHRD......TDFGI......KSDIY......
138895192	......DVLSR......IPARY......HLVMD......IVHRD......IDFGL......RSDFY......
145296137	......RSMAQ......LVNVY......YLVME......MVHRD......SDFGL......ASDVY......
145341516	......TNHRN......IVKFK......AVAME......ISHRD......CDFGF......ASDVW......
145341390	......VMMQQ......IVTIR......HVVMD......VIHRD......VDFGL......GVDVW......
145344720	......VNHRL......VVRFI......AIAME......VAHRD......CDFGY......TADVW......
145348195	......KILRL......IIRLY......FLVME......VVHRD......ADFGL......EVDVW......
145350929	......AIHKH......ILSKL......YTAME......VTHND......ADLGL......KVDVW......
145346292	......KYATK......VVRLM......VLVFE......LCHRD......IDFGL......KADVW......

145356449	......RMQSE.....VVQAY.....KIVME.....IAHYD.....GDFGC.....KADVW.....
145353208	......KAMAD.....IVDLK.....FMVMQ.....IYHRD.....GDFGL.....PADVW.....
145352610	......AVLKK.....IRTLH.....ILVLE.....IAHMD.....ADFGV.....KADIW.....
145351106	......ELLSQ.....VVRMV.....QVVME.....VVFRD.....VDFGR.....AADMW.....
145602057	......HILDF.....IIDME.....YIEME.....IVHRD.....IDFGS.....AQDVW.....
145602239	......RLHWL.....VVSML.....YRG-Q.....IYHRD.....ADFGL.....CADVW.....
145615888	......SLHSH.....IIEWF.....WIAME.....VAHRD.....ADFGM.....DVDIW.....
145613894	......AIVTL.....ICGLR.....YMLFE.....IVHRD.....IDFGL.....EVDVW.....
145609087	......EIASG.....VVSTL.....FSVME.....VAHCD.....SDFGC.....AADVW.....
145614018	......SILKQ.....IVRLH.....GIILE.....IVHRD.....TDFGF.....KVDVW.....
145614416	......KIMRS.....-----.....-----.....ITHRD.....TDFGL.....AVDLY.....
145333959	......SIMKL.....VVRLY.....YIILE.....VYHRD.....SDFGL.....VADIW.....
146282315	......WIARR.....VVKAC.....YTVSE.....MLHQD.....IDFGS.....RSDQF.....
146282748	......WFMRR.....FAELH.....YVQRE.....ILHRD.....LDFGL.....AQDLY.....
145579391	......CINKM.....VVKFY.....YLFLE.....ITHRD.....SDFGL.....PVDVW.....
146185038	......AIMKK.....VVRLY.....YMVID.....IIHRD.....ADFGV.....KADVW.....
146185336	......HILKI.....IIQLY.....YLIME.....IAHRD.....VDFGL.....CVDIW.....
146186314	......QILKK.....VLKLL.....FIVTE.....VLHRD.....IDFGI.....GSDLW.....
146186222	......-----.....-----.....-FIME.....LIHRD.....IDFGI.....AIDVW.....
146182520	......RIMNM.....IVELI.....QLVME.....ICHRD.....IDFSV.....KIDIW.....
146181416	......KIAKN.....IIKPY.....YYVME.....ICHRD.....IDFEI.....AVDIW.....
146181336	......SILKS.....ISQLY.....YLVLE.....ISHRD.....VDFGL.....YVDLW.....
146181045	......DLLKY.....ICKLY.....YLVMQ.....IVNRD.....IDFST.....AADIW.....
146174434	......KILQQ.....IIKYL.....MIVME.....IIHRD.....-DFGL.....SVDLW.....
146165798	......EGME-.....-----.....VFIME.....VIHRD.....IDFGI.....SIDIW.....
146165653	......EKLKQ.....IIRLE.....AIVME.....ICHRD.....CDLAM.....ATDIF.....
146164775	......RVHSL.....IIEYH.....FIVLD.....IVHRD.....CDFGW.....RIDIW.....
146164422	......KILNQ.....IVQLQ.....YYVMK.....ISHRD.....CDFGF.....PIDLF.....
146163794	......ELLKR.....IIRIF.....YIITE.....IVHRD.....IDWGL.....KCDLW.....
146162862	......HILKM.....LIQLY.....FLVME.....VVHRD.....VDFGL.....QVDIW.....
146161707	......NIMKE.....VIRLY.....YLSME.....ICHRD.....ADFGL.....LCDVW.....
146339837	......MILPK.....VPACF.....YVVIE.....VIHHD.....IDYGL.....RSDIF.....
147678116	......QAVAS.....IVSIY.....YLVME.....IVHRD.....TDFGI.....QSDIY.....
146386859	......CINKM.....VVKFY.....YLFLE.....ITHRD.....SDFGL.....PVDVW.....
146419327	......QIMSY.....IMRII.....YLETP.....IAHRD.....IDFGS.....PQDVW.....
146419705	......FYHRQ.....IVKLY.....WLVME.....LSHRD.....TDFGF.....AADIW.....
146423058	......LYLRL.....IIKLY.....IMVIE.....IVHRD.....ADFGL.....EVDVW.....
146413873	......CIGLT.....IIETV.....LQVME.....LAHRD.....IDFGS.....PVDVW.....
146412466	......ALGRL.....ICRLY.....YMLFE.....VVHRD.....IDFGL.....EVDVW.....
146416735	......KIGEI.....LIHTI.....YIVMD.....LAHRD.....IDFGS.....LVDVW.....
146416447	......CIGST.....IIKTI.....FEIME.....LAHRD.....IDFGS.....PVDLW.....
146413717	......CISLS.....IIDTL.....CEVME.....VSHRD.....TDFGN.....RVDIW.....
146415374	......IIMKL.....IMGLY.....YLILE.....ICHRD.....ADFGM.....PSDIW.....
146417039	......NSLKL.....IVNLV.....GIVLE.....LVHRD.....SDFGF.....KVDIW.....
146416673	......KIWEQ.....ILPLI.....FCFTD.....IVHGD.....CDFGM.....SADVW.....
146418834	......GIHKI.....ISTLV.....YLILE.....VYHRD.....CDWGL.....KVDLW.....
146412253	......YIMKR.....ITTLH.....AIVMD.....IYHCD.....IDFGL.....AGDIW.....
146413242	......DILKL.....IIAVY.....SLWFS.....WCHRD.....IDFAT.....AADAW.....
146415909	......IIMKL.....VLRLY.....YLVLE.....ICHRD.....ADFGM.....GSDIW.....
148261089	......QAAGR.....IVGIY.....YIVME.....VIHRD.....ADFGV.....RTDIY.....
146285383	......KILKL.....IIRLY.....YVVME.....VVHRD.....ADFGL.....EVDVW.....
149247990	......IIMKL.....VLRLY.....YLVLE.....ICHRD.....ADFGM.....ASDVW.....
149248714	......SVWKQ.....IIAMI.....FCIMN.....IVHGD.....CDFGM.....LADVW.....
149246101	......ALGRL.....ICRLY.....YMLFE.....VVHRD.....IDFGL.....EVDIW.....
149248274	......YYHKQ.....IVSLY.....WIALE.....LSHRD.....TDFGF.....AIDVW.....
149246183	......NSLKM.....IVNLV.....GIVLE.....LIHRD.....SDFGF.....KVDIW.....
149247327	......CVGST.....IIETV.....LQVME.....LAHRD.....IDFGS.....PVDVW.....
149242239	......QIMAF.....IMRIV.....YLETP.....IVHRD.....IDFGS.....PQDIW.....
149245934	......CIGST.....IIKTI.....FEVME.....LAHRD.....IDFGS.....PVDLW.....
149244558	......ALHLK.....VATIH.....VILLD.....VYHCD.....IDFGL.....AGDIW.....
149238780	......CISSS.....IIDTL.....CEVME.....VAHRD.....TDFGN.....RVDIW.....
149245831	......NILKQ.....VVKLY.....LLILE.....IIHRD.....SDFGV.....RIDIW.....
149238439	......IIMKL.....IMGLY.....YLILE.....ICHRD.....ADFGM.....PSDIW.....
149235728	......CILKE.....IITVY.....Y--ME.....ICHRD.....IDFAT.....AADMW.....
149237753	......SYLRL.....IIKLY.....IMVIE.....IVHRD.....ADFGL.....EVDVW.....
148984176	......RAMAD.....IVRIT.....YLAME.....IVHRD.....TDFGI.....QSDIY.....
149002035	......RAMAD.....IVRIT.....YLAME.....IVHRD.....TDFGI.....QSDIY.....
149006599	......RAMAD.....IVRIT.....YLAME.....IVHRD.....TDFGI.....QSDIY.....
147774797	......SIMKM.....IVNLV.....YMVLE.....VVHGD.....GDFGV.....AADTW.....
147772897	......KILRL.....IIRLY.....FVVME.....VVHRD.....ADFGL.....EVDVW.....
147783296	......INHRS.....IVRFK.....AIVME.....ICHRD.....CDFGY.....IADVW.....

147782993	......SVMKM......IVELH......YFAME......VYHRD......TDFGL......KADLW......
147788088	......INHRS......IVRFK......AIVME......VCHRD......CDFGY......IADVW......
147792424	......SILRR......IVQLF......YFVME......VYHRD......SDFGL......KVDIW......
147785830	......SVMKK......IVQLH......YLAME......VFHRD......TDFGL......KADIW......
147795318	......SVMKK......IVQLH......YLAME......VFHRD......TDFGL......KADIW......
147815805	......ATMKL......VVRLY......FIVLE......VYHRD......SDFGL......TADLW......
147815428	......STMKL......VIRMH......YIVLE......VFHRD......SDFGL......KADLW......
148273342	......RMLAG......LVTLF......FIVME......VVHRD......TDFGI......PTDVY......
147843535	......SVMRL......VVELY......YFVME......VYHRD......SDFGL......KADIW......
147846506	......SIMKI......IVRLH......YIILE......VYHRD......SDFGL......AADVW......
148241221	......ERLKA......IPRLG......WQVRE......LVHGD......IDFGL......WMDLY......
147860766	......LIMKM......IVNLI......YMVLE......IVHGD......ADFSV......AADTW......
147864363	......INHRS......IIRFK......AIVME......ICHRD......CDFGY......LADVW......
147865635	......SIMRR......IVKLY......YFVME......VFHRD......SDFGL......KVDIW......
148994051	......RAMAD......IVRIT......YLAME......IVHRD......TDFGI......QSDIY......
153791691	......QNLKL......IIKLY......FMIME......IVHRD......ADFGL......EVDVW......
148658672	......AAFAA......LPAVV......FLVFD......LIHRD......TNPGI......RTDLY......
148657411	......EITSQ......IVPIY......YTVME......VVHRD......SDFGI......LSDIY......
145105398	......SVMRL......IVELK......FFVME......VSHRD......SDFGL......RADIW......
148670244	......ELLMM......IISLL......YLIME......IVHRD......IDFGL......KIDVW......
148665972	......QIQQM......ITQLL......YLVME......VVHRD......IDFGL......KIDVW......
148666050	......EIQES......INQIT......YVFFE......LVLRD......EDAYI......AADVW......
148673961	......EIMKS......IIKLL......FIVLE......IVHRD......TDFGL......ATDVW......
148674014	......---RL......VARPT......YIFFT......LVLRD......VDACV......AADVW......
148670478	......DALKS......ICQLY......FMVLE......YAHRD......IDFGL......EADVW......
148673962	......EIMKS......IIKLL......FIVLE......IVHRD......TDFGL......ATDVW......
148670479	......DALKS......ICQLY......FMVLE......YAHRD......IDFGL......EADVW......
148671435	......QNLKL......IIKLY......FMVME......VVHRD......ADFGL......EVDIW......
148674015	......---RL......VARPT......YIFFT......LVLRD......VDACV......AADVW......
148686181	......AILKL......VLKLH......YLVLE......ICHRD......ADFGM......KADVW......
148681129	......RIMKI......IVKLF......YLVME......IVHRD......ADFGF......EVDVW......
148681128	......RIMKI......IVKLF......YLVME......IVHRD......ADFGF......EVDVW......
148693822	......QIMKM......IIKLY......YLVTE......VVHRD......ADFGF......QLDIW......
148686387	......SSMEK......IVRLY......HLVME......IIHRD......GDFGF......YVDIW......
148693728	......QIMKM......IIRLY......YLVTE......IVHRD......ADFGF......KVDIW......
148693729	......QIMKM......IIRLY......YLVTE......IVHRD......ADFGF......KVDIW......
148689452	......EIMSS......IISIY......VIIME......VVHRD......ADFGL......EVDSW......
148686388	......SSMEK......IVRLY......HLVME......IIHRD......GDFGF......YVDIW......
148691198	......RIMKG......IISLF......VI---......IVHRD......ADFGF......EVDIW......
148701352	......RIMKV......IVKLF......YLVME......IVHRD......ADFGF......EVDVW......
148699303	......AILKL......VLKLH......YLVLE......ICHRD......ADFGM......RADMW......
148706079	......NILKK......IVSLL......YLILE......IVHRD......IDFGL......KSDLG......
148699654	......QLLRR......VIQLV......YMVME......IVHKD......SDLGV......KVDIW......
148708393	......QLMKL......IIKLY......YIVTE......IVHRD......ANFGF......QLDVW......
148701351	......RIMKV......IVKLF......YLVME......IVHRD......ADFGF......EVDVW......
148725352	......SLNLL......IVHMI......ILIME......IFHRD......IDFGL......PTTAW......
148725351	......SLNLL......IVHMI......ILIME......IFHRD......IDFGL......PTTAW......
148725484	......EIMKL......IIKLY......YLVTE......IVHRD......ADFGF......QLDIW......
148725930	......AMMLR......IIRLH......VLILE......VFHGD......IDFGC......PAYVW......
148725933	......AMMLR......IIRLH......VLILE......VFHGD......IDFGC......PAYVW......
156094189	......RYRSC......ILSCD......YMIMP......LALRD......NDPGQ......KVDVF......
156093747	......EILAD......CLTCD......YLVNS......ICHRD......GDFGM......KADIW......
156102771	......QTLRG......IIKMY......CLIME......IVHRD......ADFGL......KLDIW......
156102064	......KYLSV......IIKFK......YIILE......IIHRD......IDFGM......SSDIW......
156099125	......INLSI......VIKLH......ILIQD......IVHRD......ADFGW......KIDNY......
156100501	......SISCR......VVKTT......IQIME......VSHRD......GDFG-......KADVW......
156100239	......KIHEQ......ICKLY......YMILE......VVHRD......IDFGL......LTDVW......
156098655	......NVLIY......IIKIF......YIVME......IAHKD......IDFGL......KCDIW......
156102929	......GIHKK......ILQMI......WVLLE......IIHCD......GDFGL......KVDMW......
156101317	......KYHSV......IIKMY......YHVLE......IFHRD......SDFGL......RSDIW......
156098292	......HILSM......LMKIY......YMIME......IIHRD......IDF--......LSDMW......
156098661	......SILRL......VIYLK......YISME......IIHRD......TDFGL......KVDAW......
156100323	......FIMKT......IVKIL......YIVLE......IMHRD......SDFGL......KIDIW......
149179274	......KSAAA......VVPIL......FLVMP......LVHRD......TDFGL......RSDLF......
149177536	......ELTAS......IVPVF......YYVMQ......ILHRD......TDFGL......QSDIY......
149178317	......KVAAA......LIHLH......YLLMD......WVHKD......IDFGL......AADIY......
149176905	......EALKK......VIEFY......YYVME......IIHRD......ADFGV......QSDLY......
149173084	......RAAAN......LCSVY......YITME......IVHRD......TDFGL......ASDVY......
149176046	......QLLTG......IVRVE......FMVMD......IIHCD......TDFGF......ATDNY......
149173775	......IAGAR......VVRTI......YIAME......IIHRD......LDFGL......RTDIY......
149175661	......EAIGS......IVAAH......YLVME......LIHRD......LDLGL......RADIY......
149016509	......QNLKL......IIKLY......FMVME......VVHRD......ADFGL......EVDIW......

```
149034597    ......QLLRR.....VIQLV.....YMVME.....IVHKD.....SDLGV.....KVDIW.....
149044623    ......QNLKL.....IIKLY.....FMVME.....VVHRD.....ADFGL.....EVDIW.....
157821861    ......DALKN.....ICQLY.....FMVLE.....YAHRD.....IDFGL.....EADVW.....
149045016    ......DIIKS.....IIKLF.....YMVME.....IAHRD.....CDFGL.....QADIW.....
149056768    ......RIMKG.....I----.....-----.....IVHRD.....ADFGF.....EVDIW.....
149062253    ......RIMKV.....IVKLF.....YLVME.....IVHRD.....ADFGF.....EVDVW.....
149062252    ......RIMKV.....IVKLF.....YLVME.....IVHRD.....ADFGF.....EVDVW.....
149057863    ......EIMKS.....IIKLL.....FIVLE.....IIHRD.....SDFGL.....AIDVW.....
149198972    ......KLVSN.....IVQIY.....FIVME.....IVHRD.....TDFGL.....RVDIY.....
149196327    ......TITAR.....IMPIY.....FFTMP.....VIHLD.....CDWGL.....RCDVY.....
149199974    ......RLSAS.....IVPVY.....WFTMK.....VIHLD.....VDWGL.....HTDIF.....
149200293    ......WLTSS.....IIKIH.....FFTMD.....IIHLD.....CDWGM.....AADIY.....
149196104    ......KLNAA.....IVPVY.....WFTMK.....VIHLD.....CDWGL.....HTDIF.....
149197064    ......RICAS.....IVPVY.....FFTMR.....ILHRD.....CDWGL.....TADIY.....
149196086    ......QLTSS.....IVPVY.....WFTMK.....VLHLD.....CDWGL.....ATDIF.....
149195918    ......KINAA.....IVPIY.....WFAMK.....VLHLD.....CDWGL.....ATDIF.....
149199835    ......RITAQ.....IVPVY.....FFTMK.....VVHLD.....GDWGL.....LCDIY.....
149197517    ......RLTAR.....IVPLY.....FFVMK.....VIHLD.....CDWGL.....RTDVY.....
149199937    ......RLTAA.....IIKVH.....YFTMD.....VLHLD.....CDWGL.....RSDIY.....
149198256    ......RITAM.....IVPLY.....YFTMK.....IAHLD.....CDWGL.....RSDIF.....
149196021    ......RLTAT.....IIPMH.....WFTMK.....VLHLD.....CDWGL.....HTDIF.....
149199006    ......RICAR.....IVPLH.....YFTMK.....IIHLD.....CDWGL.....QSDIY.....
149196110    ......RINAA.....IVPVY.....WFTMK.....ILHLD.....CDWGL.....HTDIF.....
149196819    ......KITGQ.....IIPLY.....YYVMK.....VVHLD.....TDWGL.....RSDVY.....
149195605    ......RLTAK.....IIKIH.....FFTMD.....VIHLD.....CDWGL.....RTDIY.....
149199069    ......EVAAR.....IMPVY.....FYTMK.....IIHRD.....LDWGL.....RADVY.....
149197497    ......RIMAR.....IVPVY.....FFTMK.....ILHLD.....CDWGL.....ETDIY.....
149199711    ......RLTAA.....IIPLY.....WFTMK.....ILHLD.....CDWGL.....YTDIF.....
149198388    ......RINSL.....IIPIY.....FFSMK.....IAHLD.....CDWGL.....HSDIF.....
149196507    ......RITAQ.....IIPMY.....HMAMK.....VIHRD.....MDWGI.....ASDQY.....
149195817    ......RLTAS.....IMPIY.....FFTMK.....VIHLD.....FDWGI.....KTDVY.....
149196980    ......RLTAL.....IITIH.....YFTME.....VLHLD.....CDWGL.....QTDIY.....
149198937    ......QSVAS.....LVQVF.....YLAME.....LIHRD.....TDMGL.....RADIY.....
149196009    ......QISAL.....IIPVY.....F-TMK.....VVHLD.....YDWGL.....RTDIF.....
149196453    ......RIAGQ.....IVVIF.....FIVME.....VVHRD.....SDFGV.....RADLY.....
149197397    ......KMSAL.....IIPVY.....YFTMK.....IIHLD.....HDWGL.....RTDIY.....
149263725    ......RMTSL.....FVKLF.....YLIME.....IVHRD.....ADFGF.....EVDVW.....
149256384    ......RIMKS.....IIKLF.....YLVME.....IVHRD.....CDFGL.....PVDIW.....
149258230    ......NLMKK.....IVSLL.....YLIME.....IVHRD.....IDFGL.....KSDLW.....
149260991    ......EILAT.....IICLF.....NFVLE.....IVHRD.....TDFGL.....KTDVW.....
149264217    ......DIIKS.....IIKLF.....YMVME.....IAHRD.....CDFGL.....QADIW.....
149267281    ......GIMKR.....IISLH.....FLILE.....IVHRD.....IDFGL.....KVDTW.....
149267752    ......GIMKR.....IISLH.....FLILE.....IVHRD.....IDFGL.....KVDTW.....
149268006    ......QIQQM.....ITQLL.....YLVME.....VVHRD.....IDFGL.....KIDVW.....
149268410    ......ELLMM.....IISLL.....YLIME.....IVHRD.....IDFGL.....KIDVW.....
149268414    ......ELLMM.....IISLL.....YLIME.....IVHRD.....IDFGL.....KIDVW.....
149268470    ......NIMKK.....IVSLL.....YLIME.....IVHRD.....IDFGL.....KSDLW.....
149268494    ......NIMKK.....IVSLL.....YLIME.....IVHRD.....IDFGL.....KSDLW.....
149268602    ......NLMKK.....IVSLL.....YLIME.....IVHRD.....IDFGL.....KSDLW.....
149268570    ......NLMKK.....IVSLL.....YLIME.....IVHRD.....IDFGL.....KSDLW.....
149268492    ......NIMKK.....IVSLL.....YLIME.....IVHRD.....IDFGL.....KSDLW.....
149268605    ......NLMKK.....IVSLL.....YLIME.....IVHRD.....IDFGL.....KSDLW.....
149269326    ......ATLEK.....IICLF.....NIVTE.....IVHQD.....IDFGL.....KADVW.....
149274510    ......RIMKS.....IIKLF.....YLVME.....IVHRD.....CDFGL.....PVDIW.....
149274970    ......ELLMM.....IISLL.....YLIME.....IVHRD.....IDFGL.....KIDVW.....
149275154    ......ELLMM.....IISLL.....YLIME.....IVHRD.....IDFGL.....KIDVW.....
149377287    ......WVGRR.....IVRFI.....YCVTE.....MVHQD.....IDLGA.....ASDRY.....
150863781    ......IIMKL.....VLRLY.....YLVLE.....ICHRD.....ADFGM.....ASDVW.....
150865810    ......IIMKL.....IMGLY.....YLILE.....ICHRD.....ADFGM.....PSDIW.....
150866505    ......SYLRL.....IIKLY.....IMVIE.....IVHRD.....ADFGL.....EVDVW.....
150864614    ......ALGRL.....ICRLY.....YMLFE.....VVHRD.....IDFGL.....EVDVW.....
150866211    ......RIHKI.....VSSLY.....YLILE.....VYHRD.....CDWGL.....KIDLW.....
150866167    ......QIHKA.....IIGLH.....YIIME.....VAHRD.....ADFGL.....MSDVW.....
150865388    ......YYHKQ.....IVSLY.....WLVLE.....LSHRD.....TDFGF.....ASDIW.....
150866126    ......NILRQ.....LIEVY.....RTFLE.....VCHRD.....IDFAL.....EADIW.....
150863980    ......QIMAY.....IMRII.....YLETP.....IVHRD.....IDFGS.....PQDIW.....
150864505    ......NSLKL.....IVNLV.....GIVLE.....LIHRD.....TDFGF.....KVDIW.....
150866593    ......KIGDY.....LICTI.....YIVME.....LAHRD.....IDFGS.....AADVW.....
150864583    ......NIWKE.....ILPLL.....FCITN.....IVHGD.....CDFGM.....SADIW.....
150865749    ......IISKK.....VIALV.....GIVLE.....IVHRD.....CDFGV.....KMDFW.....
150864174    ......CIGST.....IIKTI.....FEIME.....LAHRD.....IDFGS.....PVDLW.....
150865467    ......RILEM.....VCKLL.....ILVLE.....IFHRD.....CDWGL.....YIDYW.....
150864185    ......CIGST.....IIETV.....YQVME.....LAHRD.....IDFGS.....PVDVW.....
```

```
150865656   ......DTIRA......IVQIL......CLVYP......IIHRD......ADFGY......YVDYW......
149408639   ......RIMKI......IVKLF......YLIME......IVHRD......ADFGF......EVDVW......
149411471   ......LILSE......IIKVL......QLVME......ILHRD......IDFGS......ELEMW......
149412885   ......EAMKN......ICRLY......FMVME......YAHRD......IDFGL......EADVW......
149418969   ......EIMSS......IISIH......VIAME......VVHRD......ADFGL......EVDSW......
149441844   ......QNLKL......IIKLK......--IIK......-VHRD......ADFGL......EVDLW......
149514254   ......-----......INQIT......YVFFE......LVLRD......EDAYI......AADVW......
149526391   ......CINKM......IVKFY......YLFLE......ITHRD......SDFGL......PVDVW......
149539205   ......AILKL......VLKLH......YLVLE......ICHRD......ADFGM......RADMW......
149633859   ......QIMKL......IIKLY......YIVTE......IVHRD......ADFGF......LLDVW......
149634251   ......QNLKL......IIKLY......FMVME......VVHRD......ADFGL......EVDIW......
149634839   ......NILKS......IIHLE......YLVME......IVHRD......TDFGL......QCDIW......
149637923   ......EIMSS......IISIY......VIIME......VVHRD......ADFGL......EVDSW......
149638942   ......RIQQM......IVQLY......YMVME......IVHRD......IDFGL......KVDVW......
149639385   ......RCMKL......IVRLY......YLILE......VVHRD......TDFGF......AVDIW......
149639387   ......RCMKL......IVRLY......YLILE......VVHRD......TDFGF......AVDIW......
149641575   ......RIMKI......IVKLF......YLVME......IVHRD......ADFGF......EVDVW......
149707980   ......EIMSS......IIAIH......VIVME......VVHRD......ADFGL......EVDSW......
149711747   ......AILSR......IIKVL......QLVME......ILHRD......IDFGS......ELEMW......
149716655   ......CINKM......VVKFY......YLFLE......ITHRD......SDFGL......PVDVW......
149716917   ......QIMKM......IIRLY......YLVTE......IVHRD......ADFGF......KVDIW......
149716659   ......CINKM......VVKFY......YLFLE......ITHRD......SDFGL......PVDVW......
149716885   ......QIMKM......IIKLY......YLVTE......IVHRD......ADFGF......QLDIW......
149721785   ......QLPS-......ITGIV......YVFFE......IVLGD......EDTHI......AADVW......
149722602   ......AILKL......VLKLH......YLVLE......ICHRD......ADFGM......RADMW......
149725372   ......RIMKV......IVKLF......YLVME......IVHRD......ADFGF......EVDVW......
149728398   ......HSLKT......IVGLL......FLVTE......VVHRD......ADFGL......LADVW......
149728404   ......HSLKT......IVGLL......-----......VVHRD......ADFGL......LADVW......
149732765   ......SSMEK......IIRLY......HLVME......IIHRD......GDFGF......YVDIW......
149733157   ......----L......VARPT......YAFFP......LVLRD......VLENL......AADVW......
149737737   ......RIMKI......IVKLF......YLIME......IVHRD......ADFGF......EVDVW......
149739186   ......DALKN......ICQLY......FMVLE......YAHRD......IDFGL......EADVW......
149743874   ......RIMKI......IVKLF......YLVME......IVHRD......ADFGF......EVDVW......
153838643   ......QLLAR......IAKVF......YIVME......VLHAD......LDFNL......QSDVY......
153837254   ......QQTQR......VIRVF......FLVME......VVHAD......LDFGV......SDDMF......
149755292   ......QGLAR......ILRLV......FIISE......VIHRD......ADFGF......PADVW......
149755288   ......QGLAR......ILRLV......FIISE......VIHRD......ADFGF......PADVW......
149759119   ......QSLKT......VLKLL......FIVSE......VAHRD......SGFGS......PADVW......
149760327   ......QLLRR......VIQLV......YMVME......IVHKD......SDLGV......KVDIW......
149763247   ......QGLAR......ILRLV......FIISE......VIHRD......ADFGF......PADVW......
149922427   ......RIGAR......VVQVV......WLAME......IVHRD......LDFGI......AADVW......
149922550   ......QALAR......VVAIH......WIAME......LVHRD......MDFGL......AADQW......
149924963   ......RLSML......IVQVF......YMAME......IVHRD......MDFGV......TVDLF......
149922771   ......RTSCS......IVEVL......YIVME......VVHCD......VDFGI......RTDVY......
149922341   ......RATAE......VVEIS......FFVME......VVHRD......IDFGV......RVDVY......
149922421   ......HIASK......VVRVR......YVILE......VAHRD......ADFGI......ASDMF......
149922415   ......RALAD......LVALE......FFTME......FVHRD......LDFGL......PVDYY......
149922876   ......RALAR......VVQVF......YMVME......VVHRD......LDFGL......ASDLF......
149924409   ......RALAK......VVRIY......YIVME......LVHRD......MDLGV......RSDQF......
149924238   ......RAMAS......VVPVF......FLAME......LVHRD......TDFGL......RSDQF......
149923105   ......QALAR......VVTVH......YIAME......IIHRD......ADFGV......FTDQY......
149921680   ......QAMAR......VVTVY......FVAME......LVHRD......ADFGL......RSDQF......
149924911   ......RMLSQ......IVEVF......----E......LIHRD......VDFGL......RVDLY......
149921994   ......QAMAK......VVAVY......FIAME......LIHRD......SDFGL......AADQF......
149925265   ......LAASQ......IVEVL......YMVME......VIHRD......LDFGV......AADLY......
149924180   ......QLSGS......IVPIY......FFTMK......IIHRD......LDWGL......RTDVY......
149922297   ......KQVRG......LVPIF......FLVMP......VIHRD......MDFGL......RTDLY......
149921638   ......ETAAR......IVPVL......YLIMP......VIHRD......VDFGL......RTDLY......
149922732   ......KALAA......VVRII......WLILD......LVHRD......TDFGL......RSDQF......
149923368   ......RLTAV......IVTVH......FYTMQ......VIHRD......VDWGL......ATDVY......
149922137   ......QALAR......VVGVH......FVAME......LVHRD......MDFGI......RSDQF......
149916713   ......RILQN......VVHIL......YMVME......IVHRD......IDFGL......ASDVY......
149919734   ......LALAK......VVAIY......YVAME......LIHRD......MDFGL......RSDQF......
149916979   ......RLSSR......IVRVI......YLIME......LVHRD......FDFGL......QTDVY......
149919494   ......KTVAK......IVEIF......YIVCE......IVHRD......MDFGI......RIDIF......
149917700   ......QGLAR......VVSVF......WIAME......LVHRD......IDFGL......RSDQY......
149921594   ......QAMAR......VVTVY......FVAME......LAHRD......ADFGL......RSDQF......
149920088   ......ERIRD......VVRAR......VLIFD......IVHGD......ADFGI......RTDLY......
149919366   ......RALEA......LVELG......YFTME......VVHRD......LDFGL......AADIY......
149917312   ......RAMAR......VVTIY......YLAME......LVHRD......TDFGV......RSDQF......
```

```
149918417    ......DIVAH.....VVQII.....YLVME.....LIHRD.....LDFGL.....RTDIY.....
149919041    ......MTLAQ.....IVAVH.....YIVME.....ILHGD.....ADFGV.....RADQF.....
149920345    ......WALGR.....VVQVY.....FMVME.....ILHRD.....SDFGL.....PADQY.....
149920911    ......AAQGT.....IAGLY.....VFAME.....FIHRD.....IDFGL.....RTDLY.....
149918580    ......QALAR.....VVAIY.....SLAME.....LVHRD.....LDLGL.....AADQF.....
149920804    ......MTLAQ.....IVAVH.....YIVMD.....ILHGD.....ADFGV.....RADQF.....
149921139    ......RITAK.....IIKNA.....FVIVQ.....VIHCD.....ADFGI.....KSDVF.....
149920883    ......QALAQ.....VVAVH.....FVAME.....LVHRD.....MDFGL.....RSDQF.....
149918714    ......QAMAR.....VVAVH.....FVAME.....LVHRD.....LDLGL.....ASDIF.....
149918669    ......RLARR.....VARTY.....FLTME.....IIHRD.....TDFGV.....ATDLY.....
149918721    ......QAMAR.....LATVH.....YVAME.....LVHRD.....LDLGL.....ASDIF.....
149918325    ......SAARR.....VVKIH.....FLSME.....VVHRD.....IDFGL.....RTDLY.....
149916712    ......RILET.....VVHIH.....YMVME.....IVHRD.....IDFGL.....ASDVY.....
149917896    ......QALAR.....VVQIY.....YLAME.....LVHRD.....LDFGL.....AADQF.....
149917258    ......RLAMM.....IIDVF.....YIAME.....IVHRD.....ADFGV.....TVDLF.....
149920647    ......RALAD.....LISLV.....FFTME.....FLHRD.....LDFGL.....AADIY.....
149917044    ......QAMAR.....VVAVH.....FITME.....LIHRD.....TDFGL.....RADQW.....
149917959    ......RVTAR.....VVPIY.....YYTMK.....YVHCD.....MDWGG.....ATDIF.....
149919122    ......RLSMG.....IVQVF.....CMVME.....IIHRD.....MDFGI.....TIDLF.....
149919774    ......KRLDG.....VVEMY.....YIVME.....LMIRD.....IDFGL.....RVDVY.....
149921523    ......KAAGR.....ICEIL.....YIVME.....IVHRD.....MDFGV.....PADIF.....
149919998    ......QALAR.....VVAVH.....FVAME.....LIHRD.....LDFGL.....LSDQF.....
149921445    ......QAMAR.....VVPVF.....YIVME.....LVHRD.....MDFGL.....LADQF.....
149918215    ......RALAG.....LVELG.....FFTME.....CIHRD.....LDFGL.....AADYY.....
149919864    ......KVLGA.....IPRFI.....YIVSE.....VIHRD.....VDFGL.....RSDLY.....
149920153    ......MILAR.....IIAVH.....YMVMD.....VLHGD.....ADFGV.....RSDQY.....
149917099    ......RSLAR.....VVSIY.....YLAME.....IVHRD.....VDFGL.....RTDQF.....
149920901    ......QSLAK.....VVAVY.....FVAME.....LVHRD.....LDFGL.....RSDQF.....
149917411    ......QSAAR.....IIPVT.....YMVMQ.....VIHRD.....VDFGI.....RTDVY.....
149919557    ......RTLAD.....LVELR.....FFTME.....YVHRD.....LDFGL.....AADLY.....
149917781    ......RLSML.....VVQVF.....YMVME.....IVHRD.....ADFGL.....TVDLY.....
149917339    ......RVLAD.....LVHLH.....F-TME.....CVHRD.....LDFGL.....PADIY.....
149917146    ......KMLAA.....VVRIY.....WLVLE.....LVHRD.....LDFGL.....RGDQF.....
149927399    ......WVGQS.....VMRTH.....YYLGE.....MVHQD.....LDFGT.....KSDLF.....
149930903    ......QLLRR.....VIQLV.....YMVME.....IVHKD.....SDLGV.....KVDIW.....
150384711    ......RVTAQ.....IVPIY.....HLAMK.....VMHGD.....MDWGI.....RADIF.....
154285768    ......SIAKS.....IVKTF.....NHVME.....IAHRD.....TDFGV.....PLDIW.....
154284031    ......DILKD.....IVKLE.....YIFQE.....IVHRD.....TDFGC.....AVDMW.....
154280545    ......VIMKL.....IINLY.....YLVLE.....ICHRD.....ADFGM.....KADIW.....
154286918    ......RIWRY.....ILPLE.....FCFTK.....VVHRD.....CDFGM.....IVDIW.....
154279014    ......HHHRQ.....IARLY.....WLALE.....CVHRD.....CDFGF.....KVDVW.....
154283369    ......CIGST.....IIETM.....YEVME.....LAHRD.....IDFGS.....PTDLW.....
154287078    ......HIMSR.....IVKFY.....CLYMD.....IAHRD.....ADFGL.....KIDIW.....
154275668    ......HVLDG.....IVEMK.....YIEMH.....VVHRD.....IDFGS.....EQDVW.....
154295292    ......AILRE.....IVQLH.....GIILE.....IVHRD.....TDFGF.....KVDVW.....
154296275    ......AIMKL.....IMKIY.....YLVLE.....ICHRD.....ADFGM.....KVDIW.....
154290790    ......SCHAR.....IVNLI.....YIVLE.....MYHRD.....GDFGL.....EADVW.....
154295219    ......HVLDY.....IVEMS.....YIEMV.....VVHRD.....IDFGS.....PQDVW.....
154297100    ......HHHRQ.....IARLY.....WLVLE.....CVHRD.....VDFGF.....KVDVW.....
154311465    ......CISSS.....VIHTL.....CEVME.....VAHRD.....TDFGN.....AVDVW.....
154323125    ......NILSK.....IIRIE.....YMFTE.....IAHRD.....TDFGL.....AADMW.....
154319546    ......AIVTL.....ICGMR.....YMLFE.....IVHRD.....IDFGL.....EVDVW.....
154319544    ......AIVTL.....ICGMR.....YMLFE.....IVHRD.....IDFGL.....EVDVW.....
154320592    ......SLHSH.....IIEWF.....WIAME.....VGHRD.....ADFGL.....NVDIW.....
154298088    ......EYLQL.....IIKLY.....IMVLE.....IVHRD.....ADFGL.....EVDVW.....
154308291    ......SIAKA.....IVQTF.....NHVME.....IAHRD.....TDFGV.....PLDVW.....
154303609    ......CIGST.....IIETL.....YEVME.....LAHRD.....IDFGS.....PADIW.....
152984677    ......ALTRR.....VVRLF.....YIVME.....VLHGD.....FDFGL.....AADLY.....
63253969     ......SILRR.....IVLLI.....YLVME.....IVHRD.....GDFGL.....KVDIW.....
63253977     ......SILRR.....IIMLV.....FLVME.....IVHRD.....GDFGL.....KVDVW.....
63253975     ......SILRR.....IIMLV.....FLVME.....IVHRD.....GDFGL.....KVDVW.....
82706171     ......LIIQS.....IVKLH.....YLIME.....IVHRD.....ADFGL.....EVDMW.....
157134914    ......RVMKK.....IIQLI.....YLVLE.....IVHRD.....GDFGL.....KIDVW.....
109042288    ......LIIQS.....IVKLH.....YLILE.....IVHRD.....ADFGL.....EVDMW.....
109075873    ......SILRR.....IIMLV.....FLVME.....IVHRD.....GDFGL.....KVDIW.....
109120448    ......SILRR.....IVLLI.....YLVME.....IVHRD.....GDFGL.....KVDIW.....
109726701    ......SILRR.....IVLLI.....YLVME.....IVHRD.....GDFGL.....KVDIW.....
110755549    ......SILRQ.....IINLI.....FLVME.....IVHRD.....CDFGL.....KIDVW.....
112419335    ......SILRR.....IVLLI.....YLVME.....IVHRD.....GDFGL.....KVDIW.....
114596344    ......SILRR.....IIMLV.....FLVME.....IVHRD.....GDFGL.....KVDIW.....
114596346    ......SILRR.....IIMLV.....FLVME.....IVHRD.....GDFGL.....KVDIW.....
```

```
114596342    ......SILRR......IIMLV......FLVME......IVHRD......GDFGL......KVDIW......
114649473    ......SILRR......IVLLI......YLVME......IVHRD......GDFGL......KVDIW......
149589021    ......LIIQS......IVKLH......YLILE......IVHRD......ADFGL......EVDMW......
117606232    ......AILRR......IVLLI......YLVME......IVHRD......GDFGL......KVDIW......
115665242    ......AIMKH......IVRLY......YLVME......VVHRD......ADFGL......EVDMW......
115898485    ......SILRQ......IILLV......YLVME......IVHRD......GDFGL......KVDIW......
115951487    ......SILRQ......IILLV......YLVME......IVHRD......GDFGL......KVDIW......
117616934    ......LIIQS......IVKLH......YLIME......IVHRD......ADFGL......EVDMW......
118084966    ......SILRR......IVLLI......YLVME......IVHRD......GDFGL......KVDIW......
118086178    ......LIIRS......IVSLI......YLILE......IVHRD......ADFGL......EVDMW......
118089963    ......SILRR......IIMLI......YLVME......IVHRD......GDFGL......KVDIW......
119584880    ......LIIQS......IVKLH......YLILE......IVHRD......ADFGL......EVDMW......
119625407    ......SILRR......IIMLV......FLVME......IVHRD......GDFGL......KVDIW......
119908896    ......SILRR......IIMLI......FLVME......IVHRD......GDFGL......KVDVW......
119914300    ......LIIQS......IVKLH......YLIME......IVHRD......ADFGL......EVDMW......
123299521    ......AVLRR......IIMLI......YLVME......IVHRD......GDFGL......KVDIW......
123299366    ......AVLRR......IIMLI......YLVME......IVHRD......GDFGL......KVDIW......
123299354    ......AVLRR......IIMLI......YLVME......IVHRD......GDFGL......KVDIW......
123299360    ......AVLRR......IIMLI......YLVME......IVHRD......GDFGL......KVDIW......
126157519    ......AVLRR......IIMLI......YLVME......IVHRD......GDFGL......KVDIW......
123979978    ......SILRR......IIMLV......FLVME......IVHRD......GDFGL......KVDIW......
125843744    ......AVLRR......IIMLI......YLVME......IVHRD......GDFGL......KVDIW......
125845455    ......ALLRS......LIQLF......YLLME......IAHRD......ADFGL......GVDVW......
125852853    ......SILRR......IIMLI......YLVME......IVHRD......GDFGL......KVDIW......
126327453    ......SILRR......IVLLI......YLVME......IVHRD......GDFGL......KVDIW......
126336812    ......AVLQR......IVQLH......YLVLE......IVHRD......ADFGL......EVDLW......
126632013    ......SILRR......IVLLI......YLVME......IVHRD......GDFGL......KVDIW......
134026130    ......SILRR......IVLLI......YLVME......IVHRD......GDFGL......KVDIW......
148683424    ......SILRR......IIMLV......FLVME......IVHRD......GDFGL......KVDVW......
148683427    ......SILRR......IIMLV......FLVME......IVHRD......GDFGL......KVDVW......
148683426    ......SILRR......IIMLV......FLVME......IVHRD......GDFGL......KVDVW......
148703351    ......SILRR......IVLLI......YLVME......IVHRD......GDFGL......KVDIW......
148703356    ......SILRR......IVLLI......YLVME......IVHRD......GDFGL......KVDIW......
148703352    ......SILRR......IVLLI......YLVME......IVHRD......GDFGL......KVDIW......
149048216    ......SILRR......IIMLV......FLVME......IVHRD......GDFGL......KVDVW......
149064748    ......SILRR......IVLLI......YLVME......IVHRD......GDFGL......KVDIW......
149635824    ......SILRR......IVLLI......YLVME......IVHRD......GDFGL......KVDIW......
149637098    ......VIIQS......IVSLH......YLILE......IVHRD......ADFGL......EVDMW......
149640480    ......SILRQ......IIMLI......YLVME......IVHRD......GDFGL......KVDIW......
149729785    ......LIIQS......IVKLH......YLIME......IVHRD......ADFGL......EVDMW......
149730143    ......SILRR......IVLLI......YLVME......IVHRD......GDFGL......KVDIW......
157119087    ......QILKS......TVQLL......CMVFE......VIHTD......ADLGN......SADIW......
109067776    ......KLLKC......VVQLI......CMVFE......IIHTD......ADLGN......PADIW......
109067770    ......KLLKC......VVQLI......CMVFE......IIHTD......ADLGN......PADIW......
109067780    ......KLLKC......VVQLI......CMVFE......IIHTD......ADLGN......PADIW......
109067778    ......KLLKC......VVQLI......CMVFE......IIHTD......ADLGN......PADIW......
109067768    ......KLLKC......VVQLI......CMVFE......IIHTD......ADLGN......PADIW......
109070891    ......RLLKS......VVQLL......CMVFE......IIHTD......ADLGN......PADIW......
110736589    ......KILKQ......VVKLL......CMVFE......IIHTD......VDFGN......SADMW......
110765295    ......KLLKD......TVQLL......CMVFE......IIHTD......ADLGN......SADIW......
169614463    ......RHLKS......PGKVL......CLLFA......VVHTD......CDFGA......KIDMW......
115385773    ......GSSRH......IRRAL......CLVQK......LVHTD......SDFGS......PGDIW......
115397231    ......SDSSH......LRRLF......VLVFE......VVHTD......SDFGE......PVDIW......
115396672    ......KLLNH......VVSLL......CMVFE......IIHTD......ADLGN......STDVW......
114607108    ......RLLKS......VVQLL......CMVFE......IIHTD......ADLGN......PADIW......
114607104    ......RLLKS......VVQLL......CMVFE......IIHTD......ADLGN......PADIW......
114607106    ......RLLKS......VVQLL......CMVFE......IIHTD......ADLGN......PADIW......
114615296    ......KLLKC......VVQLI......CMVFE......IIHTD......ADLGN......PADIW......
114615304    ......KLLKC......VVQLI......CMVFE......IIHTD......ADLGN......PADIW......
114615294    ......KLLKC......VVQLI......CMVFE......IIHTD......ADLGN......PADIW......
114615298    ......KLLKC......VVQLI......CMVFE......IIHTD......ADLGN......PADIW......
114615306    ......KLLKC......VVQLI......CMVFE......IIHTD......ADLGN......PADIW......
114615308    ......KLLKC......VVQLI......CMVFE......IIHTD......ADLGN......PADIW......
116059700    ......EILTQ......VVRLH......CMVFD......IIHTD......CDLGN......SADIW......
169868972    ......KLLQH......VIQFL......CMVFE......VIHTD......ADLGN......SADIW......
169856744    ......KHLKS......TRPLI......G-VFR......VVHTD......ADLGE......KVDIW......
169859929    ......EILAR......CMKLL......CLVME......VVHTD......TDFGS......KIDIW......
169843728    ......KLLSR......IVSFL......CIVFE......LVHTD......ADLGN......RADVW......
118082070    ......KLLKC......VVQLI......CMVFE......IIHTD......ADLGN......PADIW......
118096959    ......SLLRC......IICLL......CLVFE......IIHAD......ADLGS......PADIW......
118102284    ......KLLKS......VVQLL......CMVFE......IIHTD......ADLGN......PADIW......
```

121699707	......QSNHA......IFQAQ......CLVHP......IIHTD......CDLGE......KVDIW......
121703027	......DIEEH......IRTHL......CLAYE......VVHTD......IDLGL......SADIW......
121708908	......DILRH......IRRPL......CLVFE......VIHTD......TDFDL......SADIW......
121705858	......QLQSD......YVRLV......CLVFE......IIHTD......SDFGN......SADIW......
121706332	......HLSKH......IRGLY......CLVHP......VVHSD......CDLGE......SADIW......
121704583	......EILRH......ISVLL......CLVME......IIHTD......CDWGS......EVDIW......
121714242	......CITEH......VATLL......CMVFD......VIHTD......TDFGL......SVDIW......
121710710	......GDL-H......IRRLL......VLVFE......VVHTD......SDFGE......PVDIW......
121714631	......KLLNK......VVSLL......CMVFE......IIHTD......ADLGN......STDIW......
121712134	......TADPS......LIRTL......CLVYP......TVHTD......ADFGR......PADIW......
121719412	......RLIEH......ISTLL......CMVFD......IIHTD......TDFGL......SVDIW......
119473693	......GTS-H......IRRLL......VLVFD......VVHTD......SDFGE......PVDIW......
119478027	......NALHC......VVQIL......CLVFP......IVHGD......TTLCK......RTEIW......
119499710	......GHLKA......VRSAL......CLIHP......IVHTD......CDFGQ......KVDIW......
119501773	......TQQNP......VITCL......CLVYE......VVHSD......ADFGL......KADIW......
119487407	......KLLNK......VVSLL......CMVFE......IIHTD......ADLGN......STDIW......
119593218	......KLLKC......IVQLI......CMVLE......IIHTD......ADLGN......PADIW......
119890945	......KLLKC......VVQLI......CMVFE......IIHTD......ADLGN......PADIW......
119624262	......RLLKS......VVQLL......CMVFE......IIHTD......ADLGN......PADIW......
119624261	......RLLKS......VVQLL......CMVFE......IIHTD......ADLGN......PADIW......
119624264	......RLLKS......VVQLL......CMVFE......IIHTD......ADLGN......PADIW......
119624259	......RLLKS......VVQLL......CMVFE......IIHTD......ADLGN......PADIW......
119915113	......RLLKS......VVQLL......CMVFE......IIHTD......ADLGN......PADIW......
123703035	......KLLKA......VVQLL......CMVFE......IIHTD......ADLGN......PADIW......
123436937	......LLMND......ITHFY......CFVLE......TIHTD......GDFGN......SADIW......
145489420	......EILKI......CVKMI......CVVME......IIHTD......VDFGN......NTDIW......
145489219	......ELLKD......CVEMV......CTVFE......IIHTD......VDFGN......STDIW......
145493645	......EILYK......TVQLL......CMVFE......VVHTD......ADLGN......TADIW......
145493571	......EILQK......VVQLL......CMVFE......VIHTD......ADLGN......TADLW......
145496812	......ELLKD......CVEMV......CTVFE......IIHTD......VDFGN......STDIW......
145502045	......EILQK......TVQLL......CMVFE......VIHTD......ADLGN......TADIW......
145520012	......DILKI......CVKMV......CVVME......IIHTD......VDFGN......NTDVW......
145529524	......EILQT......IVKLL......VLVFE......IIHAD......VDFSK......SADIW......
145532068	......EILQK......TVQLL......CMVFE......VIHTD......ADLGN......TADIW......
145532132	......EILQK......VVQLL......CMVFE......VIHTD......ADLGN......TADMW......
145547852	......EILQK......TVQLL......CMVFE......VIHTD......ADLGN......TADIW......
145552168	......EILQK......TVQLL......CMVFE......VIHTD......ADLGN......TADIW......
145473569	......EILQK......TVQLL......CMVFE......VIHTD......ADLGN......TADIW......
125558289	......EFLSE......IIQLI......CLVFE......IIHSD......VDFGN......PVDMW......
125564630	......ELLSA......AVQLL......CLVTE......IIHTD......VDFGN......SADMW......
125600189	......EFLSE......IIQLI......CLVFE......IIHSD......VDFGN......PVDMW......
125823096	......KLLKC......LVQLI......CMVLE......IIHTD......ADLGN......PADIW......
125828788	......KLLKA......VVQLL......CMVFE......IIHTD......ADLGN......PADIW......
125851680	......TLLRC......IVQLL......CLVLE......IIHTD......ADLGS......AADIW......
126340506	......KLLKC......VVQLI......CMVFE......IIHTD......ADLGN......PADIW......
146324765	......MERGS......VRSLL......CLVHP......IIHAD......CDFGS......SVDIW......
146323203	......APAEA......VTRLL......CLVFE......IAHGD......SDMGG......SIDVW......
134085886	......KLLKC......IVQLI......CMVLE......IIHTD......ADLGN......PADIW......
156717434	......KLLKC......IVQLI......CMVLE......IIHTD......ADLGN......PADIW......
145230449	......STADP......LLMLL......CLVYP......TVHTD......VDFGL......PADIW......
145231788	......HE-NH......IRLVL......CILYP......VIHTD......ADLGE......KVDIW......
154342065	......KLLTE......CARLN......CMLFD......IIHTD......ADFGN......SIDIW......
146094214	......KLLSE......CARLN......CMLFD......IIHTD......ADFGN......PIDIW......
145239213	......AAPDD......ILHLL......CLVFE......VGQID......FDLGE......RTDLW......
145239323	......RMQNE......LVTYL......VLVFP......IVHRD......KALGR......ACDMW......
145240685	......QISQH......VRLVQ......CLVFE......VMHTD......AVFGD......SADIW......
145245481	......RGSLH......IREVY......CLILQ......VVHTD......TDASR......AVDIW......
145245023	......KQWYT......TYMEL......KELYE......LTLAD......IRLGK......SSDVW......
145249020	......KLLNH......VVSLL......CMVFE......IIHTD......ADLGN......STDIW......
145254282	......VEVEH......VRRLF......CLVHE......IVHTD......SDFGE......KVDIW......
145350801	......EILKQ......VVCLY......CMVFD......IIHTD......CDLGN......SADIW......
145614791	......KLLNK......VVSLL......CMVFE......IIHTD......ADLGN......STDVW......
145328744	......ELLQA......VIRLI......CMVLE......MIHSD......VDFGN......SVDMW......
146422547	......KLLDK......VIQLL......VMVFE......VIHTD......ADLGN......LADLW......
146420200	......KILHT......IMTLY......CMVFE......IVHTD......ADMGN......STDIW......
149245248	......KLLDK......VIQLL......VMVFE......VIHTD......ADLGN......SSDLW......
147806137	......EVLSA......VVRLI......CMVLE......IIHTD......VDFGN......SVDMW......
148671224	......KLLKC......VVQLI......CMVFE......IIHTD......ADLGN......PADIW......
148690635	......RLLKS......VVQLL......CMVFE......IIHTD......ADLGN......PADIW......
156094019	......NYLKT......VVSFI......CMVFE......IIHSD......CDLGN......TADIW......

```
149029911   ......KLLKC.....IVQLI.....CMVLE.....IIHTD.....ADLGN.....PADIW.....
149046573   ......KLLKC.....VVQLI.....CMVFE.....IIHTD.....ADLGN.....PADIW.....
150951497   ......KLLDK.....VIQLL.....VMVFE.....VIHTD.....ADLGN.....SSDLW.....
149639283   ......KLLKC.....VVQLI.....CMVFE.....IIHTD.....ADLGN.....PADIW.....
149704561   ......KLLKC.....VVQLI.....CMVFE.....IIHTD.....ADLGN.....PADIW.....
149732461   ......RLLKS.....VVQLL.....CMVFE.....IIHTD.....ADLGN.....PADIW.....
149758787   ......KLLKC.....IVQLI.....CMVLE.....IIHTD.....ADLGN.....PADIW.....
149758789   ......KLLKC.....IVQLI.....CMVLE.....IIHTD.....ADLGN.....PADIW.....
154288142   ......GDPHC.....IPTLL.....CLVAE.....VCHGD.....FDNLT.....PHVIY.....
154287928   ......SALDH.....VIELL.....CLVLP.....V--ND.....GDLGG.....RIDIW.....
154272750   ......KLLNK.....VVSLL.....CMVFE.....IIHTD.....ADLGN.....STDVW.....
154276998   ......QSQSG.....LFQLY.....CLVQQ.....IVHTD.....TDFGE.....KVDIW.....
154310698   ......KLLNK.....VVSLL.....CMVFE.....IIHTD.....ADLGN.....STDVW.....
115451449   ......KILQN.....IVKLH.....SLIFE.....IMHRD.....IDWGL.....SLDMW.....
157131471   ......KILEN.....ITTLL.....ALIFE.....IMHRD.....IDWGL.....SLDMW.....
109092531   ......KILEN.....ITTLA.....ALVFE.....IMHRD.....IDWGL.....SLDMW.....
109128762   ......KILEN.....IIKLI.....ALVFE.....IMHRD.....IDWGL.....SLDMW.....
157817807   ......KILEN.....IIKLI.....ALVFE.....IMHRD.....IDWGL.....SLDMW.....
113374084   ......KILQN.....IIKLL.....SLIFE.....IMHRD.....IDWGL.....SLDMW.....
115491123   ......KILQN.....VVALL.....SLVFE.....IMHRD.....IDWGL.....SLDMW.....
114662857   ......KILEN.....IIKLI.....ALVFE.....IMHRD.....IDWGL.....SLDMW.....
115625818   ......KILEN.....IIALQ.....ALIFE.....IMHRD.....IDWGL.....SLDMW.....
116060876   ......KILQN.....VIKLL.....SLVFE.....IMHRD.....IDWGL.....SLDMW.....
169843411   ......KILQN.....IVALL.....SLITE.....IMHRD.....IDWGL.....SLDMW.....
121719783   ......KILQN.....VVALL.....SLVFE.....IMHRD.....IDWGL.....SLDMW.....
119484254   ......KILQN.....VVALL.....SLVFE.....IMHRD.....IDWGL.....SLDMW.....
123314319   ......KILTN.....IVRLL.....SFVFE.....IMHRD.....LDWGL.....SMDIW.....
123389842   ......KIMLN.....IVKLL.....IFVQE.....IMHRD.....IDWGL.....SIDMW.....
123410055   ......KILTI.....ALQLM.....ITSFE.....IMHRD.....ADWGL.....AVDMW.....
123404947   ......KILIN.....IIQLI.....SFVFE.....IMHRD.....IDWGL.....SVDIW.....
123439253   ......KILID.....IVNLY.....SFVFE.....IMHRD.....IDWGL.....SVDMW.....
123445158   ......AILNV.....ISQLL.....YLILN.....IMHRD.....IDWGL.....SLDIW.....
123439474   ......KILLN.....IVKLY.....SMVFE.....IMHRD.....IDWGL.....SIDMW.....
123455327   ......AILKK.....VVGFT.....SIVTE.....IMHRD.....IDWGL.....GIDVW.....
123471162   ......KILTI.....ILNLM.....SMITE.....IMHRD.....ADWGL.....SLDIW.....
123492560   ......RILEK.....ICNLL.....TLVLD.....IMHRD.....IDWGL.....TVDIW.....
123502980   ......KILEN.....IVKLK.....SFVFE.....VMHRD.....IDWGL.....SVDIW.....
154413432   ......CLLTK.....TVQLY.....SIITD.....IMHRD.....IDWGL.....GIDVW.....
154417828   ......KILTN.....ILQLY.....--VYS.....IMHRD.....LDWGL.....SMDIW.....
145475295   ......VFMQT.....LIPLV.....SLVY-.....VIHGD.....IDWGL.....QIDVY.....
145483745   ......RILQV.....IIDLY.....TLIFP.....IFHRD.....IDWGL.....SLDIW.....
145488874   ......DILNE.....IVRLV.....VLVFQ.....IMHLD.....IDWGV.....AVDVW.....
145508457   ......RILQI.....IIDLY.....TLIFP.....IFHRD.....IDWGL.....SLDIW.....
145509298   ......KILQN.....VLKLL.....SLILE.....IIHRD.....IDFGL.....SLDIW.....
145545093   ......LALKN.....ITELL.....SLIFE.....IFHRD.....LDWGL.....SIDSW.....
145544989   ......QVLKA.....TLQLI.....QNATF.....VMHRD.....IDWGL.....SIDMW.....
125545815   ......KILQN.....IVKLL.....SLIFE.....IMHRD.....IDWGL.....SLDLW.....
125545808   ......KILQN.....IVKLL.....SLIFE.....IMHRD.....IDWGL.....SLDMW.....
125542836   ......KILQN.....IVKLH.....SLIFE.....IMHRD.....IDWGL.....SLDMW.....
125557016   ......KILQN.....IVKLL.....SLIFE.....IMHRD.....IDWGL.....SLDMW.....
125588014   ......KILQN.....IVKLL.....SLIFE.....IMHRD.....IDWGL.....SLDLW.....
125585335   ......KILQN.....IVKLH.....SLIFE.....IMHRD.....IDWGL.....SLDMW.....
125828805   ......KILEN.....IISLI.....ALVFE.....IMHRD.....IDWGL.....SLDMW.....
125854853   ......KILEN.....IIQLM.....ALVFE.....IMHRD.....IDWGL.....SLDMW.....
126652641   ......SILKL.....IIKLL.....GLVME.....VFHRD.....IDWGL.....SVDSW.....
126649259   ......KILYN.....IVKLL.....SLVFE.....IMHRD.....IDWGL.....SLDIW.....
126276369   ......SILKN.....IVALL.....GLIFE.....IMHRD.....IDWGL.....SLDMW.....
126253861   ......KILQN.....IVKLL.....SLIFE.....IMHRD.....IDWGL.....SLDMW.....
126293917   ......KILEN.....ITTLA.....ALVFE.....IMHRD.....IDWGL.....SLDMW.....
126305189   ......KILEN.....IIKLI.....ALVFE.....IMHRD.....IDWGL.....SLDMW.....
154331468   ......SILRN.....VVRLL.....VLVTE.....IFHRD.....IDWGL.....SLDIW.....
154344663   ......MVLQT.....IVELF.....SFVFE.....IMHRD.....IDWGL.....RLDMW.....
146100964   ......MVLQT.....IVDLF.....SFVFE.....IMHRD.....IDWGL.....RLDMW.....
146075533   ......SILRN.....VVRLL.....VLVTE.....IFHRD.....IDWGL.....SLDIW.....
145253172   ......KILQN.....VVALL.....SLVFE.....IMHRD.....IDWGL.....SLDMW.....
145353055   ......KILQN.....VIKLL.....SLVFE.....IMHRD.....IDWGL.....SLDMW.....
146183969   ......SILKN.....IVQLL.....CTVFE.....IMHRD.....IDWGL.....SLDIW.....
146423613   ......SILKN.....IIGLF.....GLIFE.....IMHRD.....IDWGL.....SLDLW.....
146412716   ......KILKN.....IIGLL.....VLIFE.....IVHRD.....IDWGL.....SLDLW.....
149248112   ......KILQN.....IVGLL.....ALIFE.....IIHRD.....IDWGL.....SLDLW.....
```

```
149235019   ......SILKN......IITLL......GLIFE......IMHRD......IDWGL......SLDLW......
147815306   ......KILQN......VVKLL......SLIFE......IMHRD......IDWGL......SLDMW......
147841853   ......KILQN......IVKLL......SLIFE......IMHRD......IDWGL......SLDLW......
148679226   ......KILEN......IIKLI......ALVFE......IMHRD......IDWGL......SLDMW......
156091746   ......KILQN......IIKLL......SLIFE......IMHRD......IDWGL......SLDIW......
156098360   ......KILQN......IIKLL......SLIFE......IMHRD......IDWGL......SLDIW......
149031054   ......KILEN......IITLA......ALVFE......IMHRD......IDWGL......SLDMW......
149031055   ......KILEN......IITLA......ALVFE......IMHRD......IDWGL......SLDMW......
150951566   ......KILQN......VIGLL......ALIFE......IIHRD......IDWGL......SLDLW......
149388975   ......SILKN......IVALL......GLIFE......IMHRD......IDWGL......SLDMW......
149640718   ......KILEN......IIKLI......ALVFE......IMHRD......IDWGL......SLDMW......
149699606   ......KILEN......IIKLI......ALVFE......IMHRD......IDWGL......SLDMW......
149733508   ......KILEN......IITLA......ALVFE......IMHRD......IDWGL......SLDMW......
154283593   ......KILQN......VIPLL......SLVFE......IMHRD......IDWGL......SLDMW......
154320770   ......KILQN......IVALL......SLIFE......IMHRD......IDWGL......SLDMW......
157117894   ......NALEK......CVKML......CIAFE......LTHTD......IDFGS......PCDVW......
109080028   ......QVLEH......CVQML......CIVFE......LTHTD......VDFGS......PCDVW......
109080026   ......QVLEH......CVQML......CIVFE......LTHTD......VDFGS......PCDVW......
109081900   ......NVLKK......CVLMS......CIAFE......LTHTD......ADFGS......PCDVW......
109081902   ......NVLKK......CVLMS......CIAFE......LTHTD......ADFGS......PCDVW......
109081898   ......NVLKK......CVLMS......CIAFE......LTHTD......ADFGS......PCDVW......
109100536   ......QVLEH......CVQML......CIVFE......LTHTD......VDFGS......PCDVW......
109100534   ......QVLEH......CVQML......CIVFE......LTHTD......VDFGS......PCDVW......
109080030   ......QVLEH......CVQML......CIVFE......LTHTD......VDFGS......PCDVW......
110738363   ......DVLQK......CVQMK......CIVFE......LVHTD......IDFGS......QCDLW......
110761559   ......NALEK......CVKML......CIAFE......LTHTD......IDFGS......PCDVW......
114559997   ......NVLEK......CVQMF......CISFE......LTHTD......VDFGS......PCDVW......
115438078   ......DVLNR......CVQIQ......CIVFE......LIHTD......IDFGS......PCDLW......
115397407   ......RVLST......CIHLR......CIVTD......LIHTD......IDFGS......PCDIW......
114603755   ......QVLEH......CVQML......CIVFE......LTHTD......VDFGS......PCDVW......
114582497   ......QVLEH......CVQML......CIVFE......LTHTD......VDFGS......PCDVW......
114582503   ......QVLEH......CVQML......CIVFE......LTHTD......VDFGS......PCDVW......
114582511   ......QVLEH......CVQML......CIVFE......LTHTD......VDFGS......PCDVW......
114582505   ......QVLEH......CVQML......CIVFE......LTHTD......VDFGS......PCDVW......
114582509   ......QVLEH......CVQML......CIVFE......LTHTD......VDFGS......PCDVW......
114658081   ......NVLKK......CVLMS......CIAFE......LTHTD......ADFGS......PCDVW......
114658083   ......NVLKK......CVLMS......CIAFE......LTHTD......ADFGS......PCDVW......
114658079   ......NVLKK......CVLMS......CIAFE......LTHTD......ADFGS......PCDVW......
114658071   ......NVLKK......CVLMS......CIAFE......LTHTD......ADFGS......PCDVW......
114658069   ......NVLKK......CVLMS......CIAFE......LTHTD......ADFGS......PCDVW......
115529383   ......NVLEK......CVQML......CLSFE......LTHTD......VDFGS......PCDVW......
115738313   ......NVLEK......CVKMY......CISFE......ITHTD......IDFGS......PCDVW......
169859384   ......RVLQK......CIHLL......CLVSE......LIHTD......IDFGS......PCDAY......
117616322   ......NVLEK......CVQMF......CISFE......LTHTD......VDFGS......PCDVW......
148612855   ......NVLKK......CVLMS......CIAFE......LTHTD......ADFGS......PCDVW......
118097513   ......QVLEH......CVQML......CIVFE......LTHTD......VDFGS......PCDVW......
121700655   ......RVLST......CIHLR......CIVTD......LIHTD......IDFGS......PCDIW......
119494365   ......RVLST......CIHLR......CIVTD......LIHTD......IDFGS......PCDIW......
119574217   ......QVLEH......CVQML......CIVFE......LTHTD......VDFGS......PCDVW......
119573473   ......NVLEK......CVQMF......CISFE......LTHTD......VDFGS......PCDVW......
119590621   ......QVLEH......CVQML......CIVFE......LTHTD......VDFGS......PCDVW......
119590624   ......QVLEH......CVQML......CIVFE......LTHTD......VDFGS......PCDVW......
119889290   ......NVLEK......CVQMF......CISFE......LTHTD......VDFGS......PCDVW......
119619731   ......NVLKK......CVLMS......CIAFE......LTHTD......ADFGS......PCDVW......
145487746   ......DILFY......LVEII......CMVFE......LTHTD......IDLGG......KSDVW......
145496519   ......DVLFH......LVELY......CMIFE......IVHTD......IDLGG......KSDVW......
145500790   ......KILWY......IVRLF......FMVFE......ITHTD......IDLGG......RSDIW......
145504284   ......DILFY......LVEII......CMVFE......LTHTD......IDLGG......KSDVW......
145505796   ......DVLFH......LVELY......CMVFE......IVHTD......IDLGG......KSDVW......
145526531   ......KILWY......IVRLF......FMVFE......LTHTD......IDFGG......NSDIW......
125528311   ......DVLQR......CVQIR......CIVFE......LIHTD......IDFGS......SCDLW......
125536545   ......GMLEQ......CVQIR......CIVCE......LIHTD......PDYEG......ARRIF......
125581011   ......GMLEQ......CVQIR......CIVCE......LIHTD......PDYEG......ARRIF......
125834353   ......EVLEQ......CVRMY......CIAFE......LTHTD......VDFGN......SCDVW......
126649153   ......EILRD......CVMLY......CLVFE......LTHTD......IDFGA......SSDMW......
126276048   ......RILST......CIHLR......CIVTD......LIHTD......IDFGS......PCDMW......
126272254   ......NVLKK......CVLMS......CIAFE......LTHTD......ADFGS......PCDVW......
126291087   ......QVLEH......CVQML......CIVFE......LTHTD......VDFGS......PCDVW......
126337739   ......QVLEH......CVQML......CIVFE......LTHTD......VDFGS......PCDVW......
147906364   ......KVLEH......CVQML......CIVFE......LTHTD......VDFGS......PCDVW......
145230255   ......RVLST......CIHLR......CIVTD......LIHTD......IDFGS......PCDIW......
```

154332792	......QFMEK......LMKIQ......CIVMP......LMHTD......CDLGG......STDMW......
154339279	......SLLRY......ITNII......CMVIP......VVHTD......ADFGL......ATDVW......
146078244	......QFMEK......LMKIQ......CIVMP......LMHTD......CDLGG......STDMW......
146078248	......QFMEK......LMKIQ......CIVMP......LMHTD......CDLGG......STDMW......
145350632	......EVLKT......CIALR......CMVFD......LVHTD......IDFGS......PCDMW......
145601943	......RVLQT......CIHLR......CIVMD......LIHTD......IDFGS......PCDIW......
146179521	......EIIRK......IVKLE......CMIFE......LTHTD......IDFGG......VSDVW......
146416505	......RILST......CIHLR......CIVTD......LIHTD......IDFGS......PCDMW......
147767048	......EVLQQ......CVQIR......CIVFE......LIHTD......IDFGS......PCDIW......
147800314	......DVLQK......CVQIR......CIVFE......LIHTD......IDFGS......PCDLW......
148683287	......NVLEK......CVQMF......CISFE......LTHTD......VDFGS......PCDVW......
148701720	......QVLEH......CVQML......CIVFE......LTHTD......VDFGS......PCDVW......
153791637	......NVLEK......CVLMR......CIAFE......LTHTD......VDFGS......PCDVW......
156100421	......DILKK......IVKYH......CLVFE......LTHTD......IDFGC......SSDMW......
148887358	......NVLKK......CVLMS......CIAFE......LTHTD......ADFGS......PCDVW......
149052487	......QVLEH......CVQML......CIVFE......LTHTD......VDFGS......PCDVW......
149048090	......NVLEK......CVQMF......CISFE......LTHTD......VDFGS......PCDVW......
149052486	......QVLEH......CVQML......CIVFE......LTHTD......VDFGS......PCDVW......
149388932	......RILST......CIHLR......CIVTD......LIHTD......IDFGS......PCDMW......
149412112	......QVLEH......CVQML......CIVFE......LTHTD......VDFGS......PCDVW......
149425760	......QVLEH......CVQML......CIVFE......LTHTD......VDFGS......PCDVW......
149726026	......QVLEH......CVQML......CIVFE......LTHTD......VDFGS......PCDVW......
149751567	......NVLEK......CVQMF......CISFE......LTHTD......VDFGS......PCDVW......
149759559	......QVLEH......CVQML......CIVFE......LTHTD......VDFGS......PCDVW......
154282365	......RVLST......CIHLR......CIVTD......LVHTD......IDFGS......PCDIW......
154316297	......RVLST......CIHLR......CIVMD......LIHTD......IDFGS......PCDIW......
156121187	......QVLEH......CVQML......CIVFE......LTHTD......VDFGS......PCDVW......
108705774	......SLLKE......IVRLH......GLVFE......VLHRD......ADFGL......PVDMW......
108862876	......LILRR......VVSLD......YLVFD......VMHRD......ADFGL......SVDLW......
108862308	......KILRT......VIKLQ......YLVFE......VLHRD......ADFGL......AVDMW......
157108436	......SLLKE......IVTLH......TFVFE......VLHRD......ADFGL......SLDMW......
157132156	......SLLKD......IVELF......YMIFE......ILHRD......ADFGL......GVDIW......
157133875	......SLLKE......IVTLH......TFVFE......VLHRD......ADFGL......SLDMW......
157134220	......RPLTL......VVNLI......YLVFD......ILHRD......ADFGL......PVDMW......
157126107	......KVLQN......ILSLL......SLVFE......IMHRD......CDFGL......SIDMW......
157123020	......KILRQ......IVNLR......YLVFE......FLHRD......ADFGL......AIDVW......
157119348	......SLLKE......IVSLE......YLIFE......VLHRD......ADFGL......PVDIW......
157119357	......CLLKE......IVRLY......TLVFE......VLHRD......ADFGL......SIDMW......
157119359	......CLLKE......IVRLY......TLVFE......VLHRD......ADFGL......SIDMW......
157109037	......QILKN......VVKLK......FLVME......IIHRD......ADFGL......AVDMW......
157104530	......NTLLK......IVTVR......FIVMD......ILHRD......GDFGL......PIDIW......
157128583	......ALLRE......VINLI......WLLFD......VLHRD......ADMGF......AIDIW......
157118645	......ALLKQ......VVKLL......FLVFE......IIHRD......ADFGL......SVDIW......
157169491	......KLLHE......IIGLL......SLVFD......ILHRD......GDFGL......GVDIW......
108995587	......NTILK......IVTVR......YIVMN......ILHRD......GDFGL......AVDMW......
108995575	......NTILK......IVTVR......YIVMN......ILHRD......GDFGL......AVDMW......
108995589	......NTILK......IVTVR......YIVMN......ILHRD......GDFGL......AVDMW......
108995578	......NTILK......IVTVR......YIVMN......ILHRD......GDFGL......AVDMW......
108995584	......NTILK......IVTVR......YIVMN......ILHRD......GDFGL......AVDMW......
108995581	......NTILK......IVTVR......YIVMN......ILHRD......GDFGL......AVDMW......
108995592	......NTILK......IVTVR......YIVMN......ILHRD......GDFGL......AVDMW......
109067546	......SLLKG......IVLLH......TLVFE......ILHRD......ADFGL......CLDMW......
109077479	......KLLQE......IIGLL......SLVFD......ILHRD......ADFGL......GVDMW......
109089720	......SLLKE......IVSLQ......YLIFE......VLHRD......ADFGL......PVDIW......
109089724	......SLLKE......IVSLQ......YLIFE......VLHRD......ADFGL......PVDIW......
109097207	......SLLKE......IVKLL......YLVFE......VLHRD......ADFGL......AVDIW......
109097197	......SLLKE......IVKLL......YLVFE......VLHRD......ADFGL......AVDIW......
109097205	......SLLKE......IVKLL......YLVFE......VLHRD......ADFGL......AVDIW......
109097199	......SLLKE......IVKLL......YLVFE......VLHRD......ADFGL......AVDIW......
109100608	......SLLKG......IVLLH......TFVFE......VLHRD......ADFGL......ELDIW......
109112071	......KALQE......VVQLK......VLAFE......IVHRD......ADFGL......GVDLW......
109112159	......KILQL......VVNLI......YLVFD......ILHRD......ADFGL......PIDLW......
109114869	......KILRQ......VVNMK......YLVFE......FLHRD......ADFGL......AIDVW......
109114872	......KILRQ......VVNMK......YLVFE......FLHRD......ADFGL......AIDVW......
109118322	......SLLKE......IVQLL......YLVFE......VIHRD......ADFGL......AVDIW......
109118320	......SLLKE......IVQLL......YLVFE......VIHRD......ADFGL......AVDIW......
109118314	......SLLKE......IVQLL......YLVFE......VIHRD......ADFGL......AVDIW......
109130678	......SLLKD......IVTLH......TLVFE......VLHRD......ADFGL......QIDMW......
109465993	......KLLQE......IIGHY......STQVD......ILHRD......ADFGL......GVDMW......
166157480	......SLLKD......IVTLH......TLVFE......VLHRD......ADFGL......QIDMW......
109487210	......SLLKG......IVLLH......TFVFE......VLHRD......ADFGL......ELDIW......

```
109505017    ......KILRQ.....IINMK.....YLVFE.....FLHRD.....ADFGL.....AIDVW.....
109505848    ......KILRQ.....IINMK.....YLVFE.....FLHRD.....ADFGL.....AIDVW.....
109505850    ......KILRQ.....IINMK.....YLVFE.....FLHRD.....ADFGL.....AIDVW.....
109510558    ......NTILK.....IITVR.....YIVMN.....ILHRD.....GDFGL.....AVDMW.....
109097505    ......ALLRR.....VVRLM.....TLVFE.....IVHRD.....ADFGL.....PVDMW.....
157818145    ......ALLRE.....VISLQ.....WLLFD.....VLHRD.....ADMGF.....AIDIW.....
157824204    ......KILRQ.....VVNMK.....YLVFE.....FLHRD.....ADFGL.....AIDVW.....
110289666    ......MLLRE.....VVKLV.....YLAFD.....IIHRD.....ADFGL.....AVDMW.....
110611797    ......TLLLR.....IVELK.....FLVMG.....IIHRD.....ADFGL.....SIDMW.....
118404796    ......CLLKE.....IVRLH.....TLVFE.....VLHRD.....ADFGL.....SIDMW.....
110755289    ......KILRQ.....IVNLR.....YLVFE.....FLHRD.....ADFGL.....AIDVW.....
110760390    ......KILQQ.....IIELL.....IMVFE.....IIHRD.....ADFGL.....AIDMW.....
110761270    ......NTLLK.....IVTVR.....FIVMD.....ILHRD.....GDFGL.....PIDMW.....
110761543    ......SLLRE.....IVQLF.....YLVFE.....ILHRD.....ADFGL.....AVDVW.....
110765291    ......SLLKE.....IVTLH.....TFVFE.....VLHRD.....ADFGL.....SLDMW.....
110765116    ......SLLKE.....IVRLM.....YLIFE.....IFHRD.....ADFGL.....AIDIW.....
110766771    ......SVLLS.....IVHLR.....FLAME.....IVHRD.....ADFGL.....SVDMW.....
169598548    ......SILQK.....IVTLH.....VLVME.....IMHRD.....ADFGM.....EVDMW.....
111307039    ......CLLKE.....IVRLH.....TLVFE.....VLHRD.....ADFGL.....SIDMW.....
115441609    ......SILRL.....VVRLL.....YLVFE.....VLHRD.....ADLGL.....GVDIW.....
115435124    ......LILRR.....IIKLE.....YLVFE.....ILHRD.....ADFGL.....AVDLW.....
115441957    ......ALLRR.....IVRLR.....YLVFD.....VLHRD.....ADFGL.....GVDLW.....
115453015    ......LILRR.....VVKLE.....YLVFE.....VLHRD.....ADFGL.....GVDLW.....
115473955    ......LILRR.....VIKLD.....YLVFD.....VLHRD.....GDFGL.....GVDLW.....
115477248    ......SLLRM.....VVRLL.....YLVFE.....VLHRD.....ADLGL.....PVDIW.....
115481186    ......ACQHA.....IVQIK.....FLVLE.....VIHRD.....CDFGA.....AVDMW.....
115489258    ......LILRR.....VVSLD.....YLVFD.....VMHRD.....ADFGL.....SVDLW.....
115433166    ......KLLQH.....VVSLL.....FMVFE.....VLHRD.....ADFGL.....AVDVW.....
115398892    ......SLLKE.....IVRLF.....YLVFE.....ILHRD.....ADFGL.....GVDMW.....
115432970    ......SLMKE.....IVSLY.....MLVFE.....VLHRD.....GDFGL.....SIDIW.....
115492059    ......RLLRE.....VIPLL.....LLVLP.....IIHRD.....ADFGI.....ALDLW.....
115395888    ......-----......----D.....FLVMD.....IMHRD.....ADFGM.....EIDMW.....
115391413    ......SLCSE.....VVQLE.....FMVFE.....VLHRD.....GDLGL.....AVDLW.....
115389042    ......KYLQE.....IIALH.....NLVLE.....ILHRD.....ADFGL.....AVDVW.....
114215592    ......SLLKE.....VVRLL.....YLIFE.....VLHRD.....ADFGL.....PVDVW.....
114582670    ......SLLKG.....IVLLH.....TFVFE.....VLHRD.....ADFGL.....ELDIW.....
114582672    ......SLLKG.....IVLLH.....TFVFE.....VLHRD.....ADFGL.....ELDIW.....
114609033    ......ALLRE.....VIALQ.....WLLFD.....VLHRD.....ADMGF.....AIDIW.....
114613062    ......KILRQ.....IINMK.....YLVFE.....FLHRD.....ADFGL.....AIDVW.....
114613058    ......KILRQ.....IINMK.....YLVFE.....FLHRD.....ADFGL.....AIDVW.....
114613064    ......KILRQ.....IINMK.....YLVFE.....FLHRD.....ADFGL.....AIDVW.....
114613066    ......KILRQ.....IINMK.....YLVFE.....FLHRD.....ADFGL.....AIDVW.....
114613060    ......KILRQ.....IINMK.....YLVFE.....FLHRD.....ADFGL.....AIDVW.....
114614410    ......SLLKG.....IVLLH.....TLVFE.....ILHRD.....ADFGL.....CLDMW.....
114646391    ......SLLKD.....IVTLH.....TLVFE.....VLHRD.....ADFGL.....QIDMW.....
114646389    ......SLLKD.....IVTLH.....TLVFE.....VLHRD.....ADFGL.....QIDMW.....
114646393    ......SLLKD.....IVTLH.....TLVFE.....VLHRD.....ADFGL.....QIDMW.....
114646381    ......SLLKD.....IVTLH.....TLVFE.....VLHRD.....ADFGL.....QIDMW.....
114649080    ......ALLRE.....VISLQ.....WLLFD.....VLHRD.....ADMGF.....AIDKW.....
114670586    ......SLLKE.....IVRLL.....YLVFE.....VIHRD.....ADFGL.....AVDIW.....
114670584    ......SLLKE.....IVRLL.....YLVFE.....VIHRD.....ADFGL.....AVDIW.....
114670582    ......SLLKE.....IVRLL.....YLVFE.....VIHRD.....ADFGL.....AVDIW.....
114670580    ......SLLKE.....IVRLL.....YLVFE.....VIHRD.....ADFGL.....AVDIW.....
114688340    ......SLLKD.....IVTLH.....TLVFE.....VLHRD.....ADFGL.....QIDMW.....
118150570    ......ALLKR.....IVRLM.....TLVFE.....VLHRD.....ADFGL.....PVDIW.....
154147579    ......SLLKD.....IVTLH.....TLVFE.....VLHRD.....ADFGL.....QIDMW.....
115660790    ......KALQE.....VVKLK.....VLVFE.....IMHRD.....ADFGL.....GADLW.....
115682385    ......VTQSE.....LPSLH.....NKVNE.....VLHRD.....ADFGL.....SIDMW.....
115702469    ......ALLKE.....IVKLH.....YLVFE.....VLHRD.....ADFGL.....AVDIW.....
115707447    ......STLLK.....IVTVR.....YIVMD.....ILHRD.....GDFGL.....PIDMW.....
115722990    ......ALLRE.....VICLR.....YLLCD.....VLHRD.....ADMGF.....AIDIW.....
115964384    ......KALQE.....VVKLK.....VLVFE.....IMHRD.....ADFGL.....GADLW.....
115954076    ......STLLK.....IVTVR.....YIVMD.....ILHRD.....GDFGL.....PIDMW.....
116000498    ......NILLS.....IVNVN.....FMVME.....IMHRD.....CDFGL.....AIDVW.....
116000651    ......KILKK.....VVDLK.....YLVFE.....ILHRD.....ADFGL.....SVDMW.....
116058980    ......KILQR.....VISLK.....FLVFE.....VFHRD.....CDFGL.....AIDMW.....
116058692    ......KLLQE.....VIELV.....NLVFE.....VLHRD.....ADFGL.....GVDVW.....
116060672    ......TMLRA.....VVRLH.....SLAID.....IIHRD.....ADFGL.....AIDIW.....
118786221    ......RALQH.....VLELL.....SLVFE.....LMHRD.....ADFGL.....SIDLW.....
169864791    ......KILKA.....IVNIL.....YMVFP.....ILHRD.....ADFGL.....EVDIW.....
169867318    ......SLLKE.....IVKLL.....YLVFE.....ILHRD.....ADFGL.....AIDMW.....
```

```
169865137   ......ALNRE......VVALR......YMVFE......ILHRD......GDLGL......AIDCW......
169861129   ......KLLQS......VVQLY......FMVFE......VIHRD......ADFGL......EVDMW......
169849493   ......KFLRE......VIELL......NLVLE......ILHRD......ADFGL......GVDIW......
169844288   ......YALMT......VVRIR......-----......-LTQD......ADFGL......AVDMW......
169844504   ......SLMKE......IVRLH......VLIFE......VLHRD......GDFGL......SIDVW......
116668167   ......SLLKE......IVKLL......YLVFE......VLHRD......ADFGL......AVDIW......
116668171   ......SLLKE......IVKLL......YLVFE......VLHRD......ADFGL......AVDIW......
116812135   ......SLLKE......IVCLE......YLIFE......VLHRD......ADFGL......PVDIW......
117380776   ......KALQS......VVALL......YLVQE......IMHRD......ADCGL......AVDVW......
117644918   ......SLLKE......IVSLQ......YLIFE......VLHRD......ADFGL......PVDIW......
117645044   ......KLLQE......IIGLL......SLVFD......ILHRD......ADFGL......GVDMW......
117645398   ......SLLKE......IVSLQ......YLIFE......VLHRD......ADFGL......PVDIW......
117646652   ......KLLQE......IVGLL......SLVFD......ILHRD......ADFGL......GVDMW......
117616778   ......KALQE......VVQLK......VLAFE......IVHRD......ADFGL......GVDLW......
117616308   ......KILRQ......IINMK......YLVFE......FLHRD......ADFGL......AIDVW......
161086911   ......SLLKG......IVLLH......TLVFE......ILHRD......ADFGL......CLDMW......
117616822   ......KILQL......VVNLI......YLVFD......ILHRD......ADFGL......PIDLW......
118082519   ......SLLKD......IVTLH......TLVFE......VLHRD......ADFGL......QIDMW......
118085024   ......ALLRE......VISLQ......WLLFD......VLHRD......ADMGF......AIDIW......
118085807   ......SLLKG......IVLLH......TLVFE......ILHRD......ADFGL......CLDMW......
118088685   ......ALLRE......VIALQ......WLLFD......VLHRD......ADMGF......AIDIW......
118093448   ......SLLKH......IVLLH......TFVLE......ILHRD......ADFGL......DLDIW......
118102398   ......SLLKN......IVTLH......TLVFE......ILHRD......ADFGL......PIDMW......
118102964   ......KILRQ......VVNMK......YLVFE......FLHRD......ADFGL......AIDVW......
90074906    ......SLLKG......IVLLH......TLVFE......ILHRD......ADFGL......CLDMW......
90077252    ......SLLKN......IVTLH......TLVFE......ILHRD......ADFGL......PIDMW......
121699374   ......SLLKE......IVRLL......YLVCE......ILHRD......ADFGL......GVDMW......
121701259   ......RLLKE......VIGLL......ILVFP......IIHRD......ADFGI......ALDLW......
121706166   ......SLCSE......VVQLA......FMVFE......VLHRD......GDLGL......AVDLW......
121715334   ......QTLLE......IVYLR......FLVMD......IMHRD......ADFGM......EIDMW......
121711193   ......KYLQE......IIALH......NLVLE......VLHRD......ADFGL......AVDVW......
121712552   ......KLLKM......ILQLR......YMVTP......ILHRD......ADFGL......AIDMW......
121718847   ......KLLQH......VVSLL......FMVFE......VLHRD......ADFGL......AVDVW......
121717671   ......SLMKE......IVSLY......MLVFE......VLHRD......GDFGL......SIDIW......
119469234   ......SLLKE......IVRLL......YLVFE......ILHRD......ADFGL......GVDMW......
119482530   ......KYLQE......IIALH......NLVLE......VLHRD......ADFGL......AVDVW......
119480865   ......QTLLE......IVYLR......FLVMD......IMHRD......ADFGM......EIDMW......
119500086   ......SLLKE......VVQLF......YLVME......ILHRD......GDFGL......TVDMW......
119490685   ......ALCSE......VVQLA......FMVFE......VLHRD......GDLGL......AVDLW......
119498729   ......SLMKE......IVSLY......MLVFE......VLHRD......GDFGL......SIDIW......
119498639   ......KLLKM......ILQLK......YMVTP......ILHRD......ADFGL......AIDMW......
119498917   ......KLLQH......VVSLL......FMVFE......VLHRD......ADFGL......AVDVW......
119495217   ......RLLKE......VISLL......ILVFP......IIHRD......ADFGI......ALDLW......
119590702   ......SLLKG......IVLLH......TFVFE......VLHRD......ADFGL......ELDIW......
119574436   ......CLLKE......IVRLH......TLVFE......----D......ADFGL......SIDMW......
119574435   ......CLLKE......IVRLH......TLVFE......VLHRD......ADFGL......SIDMW......
119574589   ......SLLKE......IVSLQ......YLIFE......VLHRD......ADFGL......PVDIW......
119579702   ......SLLKD......IVTLH......TLVFE......VLHRD......ADFGL......QIDMW......
119579698   ......SLLKD......IVTLH......TLVFE......VLHRD......ADFGL......QIDMW......
119587113   ......TLLLR......IVELK......FLVMG......IIHRD......ADFGL......SIDMW......
119579699   ......SLLKD......IVTLH......TLVFE......VLHRD......ADFGL......QIDMW......
157817023   ......KILRQ......VVNMK......YLVFE......FLHRD......ADFGL......AIDVW......
157817073   ......KILRQ......VVNMK......YLVFE......FLHRD......ADFGL......AIDVW......
119590703   ......SLLKG......IVLLH......TFVFE......VLHRD......ADFGL......ELDIW......
119597279   ......SLLKG......IVLLH......TLVFE......ILHRD......ADFGL......CLDMW......
119597278   ......SLLKG......IVLLH......TLVFE......ILHRD......ADFGL......CLDMW......
119890699   ......AVLRH......VVRLF......TLVFE......VVHRD......ADFGL......PVDLW......
119891197   ......KILRQ......IINMK......YLVFE......FLHRD......ADFGL......AIDVW......
119891195   ......KILRQ......IINMK......YLVFE......FLHRD......ADFGL......AIDVW......
119891199   ......KILRQ......IINMK......YLVFE......FLHRD......ADFGL......AIDVW......
119892633   ......SLLKD......IVTLH......TLVFE......VLHRD......ADFGL......QIDMW......
119617265   ......SLLKE......IVKLL......YLVFE......VLHRD......ADFGL......AVDIW......
119617974   ......SLLKD......IVTLH......TLVFE......VLHRD......ADFGL......QIDMW......
145309300   ......KILRQ......IINMK......YLVFE......FLHRD......ADFGL......AIDVW......
119609759   ......SLLKE......IVRLL......YLVFE......VIHRD......ADFGL......AVDIW......
119614539   ......KILRQ......IINMK......YLVFE......FLHRD......ADFGL......AIDVW......
119901256   ......ALLRE......VIALQ......WLLFD......VLHRD......ADMGF......AIDIW......
119904670   ......ALLRE......VISLQ......WLLFD......VLHRD......ADMGF......AIDIW......
149642973   ......SLLKE......IVRLL......YLVFE......VIHRD......ADFGL......AVDIW......
119912236   ......KILRQ......VVNMK......YLVFE......FLHRD......ADFGL......AIDVW......
```

```
119921292   ......SLLKG.....IVLLH.....TFVFE.....VLHRD.....ADFGL.....ELDIW......
89275180    ......CLLKE.....IVRLH.....TLVFE.....----D.....ADFGL.....SIDMW......
148228730   ......TLLLK.....IVELK.....FLVMG.....IVHRD.....ADFGL.....AIDMW......
120537647   ......KILRQ.....VVNMK.....YLVFE.....FLHRD.....ADFGL.....AIDVW......
123392715   ......SNLQT.....IINLR.....YMVFD.....IVHRD.....GDFGL.....SSDIW......
123392124   ......CLLSE.....IIHAQ.....SIHFN.....IIHCD.....IDFGL.....AVDMW......
123401158   ......KSMHS.....IIHLN.....YAVLD.....YMHRD.....CDLGL.....KIDVW......
123433165   ......CLLSE.....IIHAQ.....SIHFN.....IIHCD.....IDFGL.....AVDMW......
123436532   ......RQLCE.....IVRYI.....YIATE.....LLHRD.....IDFGY.....ALDMW......
123426196   ......ETLKS.....IIQML.....FIIME.....YIHRD.....IDFGY.....KMDIW......
123431415   ......NVLQT.....IVSYK.....CLALE.....FVHRD.....TDFGL.....SIDIW......
123433704   ......NTLRA.....VLKIY.....AIVYS.....VIHRD.....VDFGL.....AIDIW......
123444396   ......FMHKA.....IVQLH.....TLLLE.....IMHRD.....GDLGL.....AIDIW......
123439023   ......GVLQN.....IVSYG.....CITLE.....YAHRD.....TDFGL.....AIDIW......
123975138   ......SAMKA.....IVKCL.....VIISE.....YLHRD.....ADFSL.....GIDSW......
123456898   ......QALSV.....IVKLH.....SLVME.....IIHRD.....CDLGL.....AVDTW......
123451223   ......KLLKQ.....IVLLK.....YLVFE.....VVHRD.....GDFGL.....EVDIW......
123472153   ......TLLSE.....IIHIK.....VLAFV.....IIHCD.....IDFGL.....SIDLW......
123490726   ......CILKS.....ILHFR.....IMVCE.....IVHRD.....GDFGT.....EIDIW......
154411717   ......SILKE.....VVGLN.....TLVFE.....IIHRD.....ADFGL.....PVDIW......
123486670   ......QTLRA.....VVHIL.....SVVYS.....VIHND.....IDFGL.....GVDIW......
123491839   ......CLLSE.....IIHAK.....TLNFH.....IIHCD.....IDFGL.....AVDIW......
123508697   ......ALMRN.....IVALK.....TMVSE.....IMHRD.....CDFGL.....SVDVW......
123503810   ......SILRS.....IARLI.....YLVFS.....ILHRD.....TDFGL.....EIDIW......
123487049   ......KVLQS.....IIQYF.....FISME.....FAHRD.....ADLGL.....SVDIW......
123509071   ......RLLQK.....IVKLQ.....YLVLE.....VIHRD.....GDFGL.....EVDIW......
123490526   ......RYMRE.....ILQLY.....SLVTD.....FVHRD.....GDFGS.....AIDVW......
123480258   ......QALSV.....IVKLH.....NLVME.....MIHRD.....CDLGL.....PIDIW......
123508241   ......SILKT.....IIGLK.....FLAFE.....IIHRD.....CDFGM.....SIDMW......
123499233   ......SNLQH.....IINLR.....YMVFD.....IIHRD.....GDFGL.....SSDIW......
123494026   ......RWLKN.....IVKLY.....TLVME.....VIHRD.....IDFGL.....LIDIW......
154417060   ......SILKK.....IVSLV.....TIVLE.....VIHRD.....CDFGL.....SADIW......
154418554   ......ALLKE.....IVQLY.....TLIFE.....IIHRD.....ADFGL.....PVDVW......
154421652   ......SILSE.....VVSVK.....ILIME.....IIHRD.....CDFGL.....SVDVW......
154422366   ......AAHCV.....IVKLK.....YLIFD.....VLHRD.....GDFGY.....PSDIW......
154419658   ......KCLKE.....IIRLR.....WLSFD.....ILHRD.....GDFGL.....EVDVW......
154413046   ......AILNN.....IVECK.....HVVFE.....IIHRD.....ADFGL.....EVDIW......
154421760   ......KILRA.....VVHLI.....YMVFE.....IYHRD.....GDFGL.....EIDNW......
154414435   ......SNLQT.....IINLR.....YMVFD.....IVHRD.....GDFGL.....SSDVW......
154412682   ......RQLNE.....IVKFN.....YLSTE.....IMHRD.....IDFGL.....EIDMW......
126165309   ......SLLKE.....IVKLL.....YLVFE.....VLHRD.....ADFGL.....AVDIW......
121543965   ......CLLKE.....IVRLY.....VLIFE.....VLHRD.....ADFGL.....SIDMW......
157816935   ......KILRQ.....VVNMK.....YLVFE.....FLHRD.....ADFGL.....AIDVW......
123228017   ......SLLKD.....IVTLH.....TLVFE.....VLHRD.....ADFGL.....QIDMW......
157816961   ......KILRQ.....VVNMK.....YLVFE.....FLHRD.....ADFGL.....AIDVW......
122893693   ......SLMKE.....IVRLY.....MLVFE.....VLHRD.....ADFGL.....SIDVW......
123994147   ......SLLKN.....IVTLH.....TLVFE.....ILHRD.....ADFGL.....PIDMW......
145479295   ......SLLKE.....IVPLK.....YLIFD.....VIHRD.....ADFGL.....PVDIW......
145476217   ......SLLKE.....IVKLM.....VLVFE.....ILHRD.....ADFGL.....SIDIW......
145484037   ......SILKE.....IVGLK.....YLVFE.....IIHRD.....ADFGL.....GVDIW......
145483853   ......EILRS.....IVLFK.....YIIME.....IIHRD.....CDLGM.....KVDVW......
145489482   ......SLLQR.....VVKIL.....RVIME.....CIHRD.....GDFGS.....AIDMW......
145490134   ......DILMQ.....VVKLI.....VIVLE.....IAHRD.....CDFGS.....SIDIW......
145486772   ......KLLKI.....ILRLR.....FLVFD.....IIHRD.....ADFGL.....QIDMW......
145492790   ......NLLLS.....IVKVK.....FMVME.....VMHRD.....CDFGL.....KIDIW......
145494588   ......SALSS.....IVPLQ.....YIVLP.....FMHRD.....SDFGI.....SVDVW......
145493457   ......KLLKI.....ILRLR.....FLVFD.....IIHRD.....ADFGL.....QIDMW......
145496670   ......SVLLQ.....IVELK.....YLIME.....IFHRD.....GDFGL.....ALDIF......
145503063   ......SILQT.....IVKLI.....RLVME.....CMHRD.....GDFGM.....AIDVW......
145503900   ......AILLK.....VISLF.....YLVFD.....VLHRD.....ADFGL.....AVDIW......
145508357   ......EILRS.....IVLFK.....YIIME.....IIHRD.....CDLGM.....KVDVW......
145507148   ......KFLTS.....VIKIK.....KTIKT.....IIHRD.....ADFGL.....QLDVW......
145507869   ......HLLSS.....IVSFQ.....YLVLE.....VYHRD.....CDFGM.....SVDVW......
145508131   ......SILKE.....IVGLK.....YLVFE.....IIHRD.....ADFGL.....GVDIW......
145509693   ......AALSS.....IVPLQ.....YIVLP.....FMHRD.....SDFGI.....SVDVW......
145510875   ......DILMQ.....VVKLI.....VIILE.....IAHRD.....CDFGS.....SIDIW......
145510857   ......RFMNT.....LNIPK.....SICFV.....ILHRD.....TDFGL.....ALDVW......
145514874   ......QILQA.....IVQMI.....TIIFT.....IMHRD.....IDWGL.....SIDVW......
145513973   ......QILKA.....IVQII.....TIIFA.....IMHRD.....IDWGL.....SIDVW......
145520365   ......SILKE.....IVGLK.....YLVFE.....IIHRD.....ADFGL.....GVDIW......
145520579   ......EILKS.....IVALK.....YLIME.....IVHRD.....CDLGM.....KVDVW......
```

145523678	......SILQT.....IVKLI.....RLVME.....CMHRD.....GDFGM.....AIDVW.....
145523960	......QLLTS.....ININIV.....CLTME.....ILHRD.....GDFGI.....SIDIW.....
145524890	......SLLKE.....IVKLM.....VLVFE.....ILHRD.....ADFGL.....SIDIW.....
145534283	......SLLKE.....IVPLK.....YLIFD.....VIHRD.....ADFGL.....PVDIW.....
145532204	......KLLKI.....ILRLR.....FLVFD.....IIHRD.....ADFGL.....QIDMW.....
145535253	......HLHEK.....VIKVY.....SIYME.....VVHRD.....GDFGS.....EVDIW.....
145537740	......SLLRE.....IVQLR.....QLVFE.....ILHRD.....ADFGL.....PVDIW.....
145538313	......HLLSS.....IVSFQ.....YLVLE.....VYHRD.....CDFGM.....SVDVW.....
145540489	......SILNQ.....IIKLI.....HLVMN.....IIHRD.....-DFGL.....AIDMW.....
145539344	......SLLKE.....IVPLK.....YLIFD.....VIHRD.....ADFGL.....PVDIW.....
145542664	......AILQK.....IINLL.....LLIFE.....VLHRD.....ADFGL.....GVDIW.....
145540802	......SLLRE.....IVQLR.....QLVFE.....ILHRD.....ADFGL.....PVDIW.....
145543097	......HLLSS.....IVSFK.....FLVLE.....VYHRD.....CDFGM.....SVDVW.....
145473727	......KLLKI.....ILRLR.....FLVFD.....IIHRD.....ADFGL.....QIDMW.....
145551478	......SCLTN.....VQNLH.....YLIQE.....IMHRD.....TEFHL.....KVDLF.....
145548231	......EILKS.....IVSFK.....YIIME.....IIHRD.....CDLGM.....QVDVW.....
145548455	......-----.....TSTSS.....--IFE.....IIHRD.....ADFGL.....GVDIW.....
145550497	......KILQR.....VVNLL.....HLVFE.....ILHRD.....ADFGL.....AIDIW.....
125579842	......LILRR.....VVSLD.....YLVFD.....VMHRD.....ADFGL.....SVDLW.....
125528707	......SILRL.....VVRLL.....YLVFE.....VLHRD.....ADLGL.....GVDIW.....
125526526	......EMLAA.....VVRLR.....HLVMD.....VIHRD.....CDLGL.....AVDMW.....
125524804	......LILRR.....IIKLE.....YLVFE.....ILHRD.....ADFGL.....AVDLW.....
125525936	......LILRK.....VIKLE.....YLVFE.....VLHRD.....ADFGL.....GVDLW.....
125529183	......KILKK.....VIQLK.....YMVFE.....VLHRD.....ADFGL.....AVDMW.....
125528934	......ALLRR.....IVRLC.....YLVFD.....VLHRD.....ADFGL.....GVDLW.....
125537147	......LILRR.....VVSLD.....YLVFD.....VMHRD.....ADFGL.....SVDLW.....
125531164	......ACQHA.....IVQIK.....FLVLE.....VIHRD.....CDFGA.....AVDMW.....
125536073	......KILRT.....VIKLQ.....YLVFE.....VLHRD.....ADFGL.....AVDMW.....
125531181	......DCLAA.....VVQLR.....FIVME.....MAHRD.....CDFGM.....VVDMW.....
125536439	......RALEA.....IVQLI.....YIVME.....LMHRD.....CDLGF.....TVDSW.....
125531186	......ACT--.....IVQIK.....FLVME.....VIHRD.....CDFGS.....GVDMW.....
125536457	......AAMEV.....IVQPR.....VLVME.....LMHRD.....CDLGL.....CVDAW.....
125531163	......ACQHA.....IVQIK.....FLVLE.....VIHRD.....CDFGA.....AVDMW.....
125533934	......LVLRK.....VIKLE.....YLVFE.....VLHRD.....ADFGL.....SVDMW.....
125536438	......RALEA.....IVQLI.....YIVME.....LMHRD.....CDLGM.....TIDMW.....
158512871	......NILLS.....IVEVK.....FMVME.....VLHRD.....CDFGL.....AIDMW.....
125531179	......ACT--.....IVQIK.....FLVME.....VIHRD.....CDFGS.....GVDMW.....
125539905	......HVLRR.....VVRLE.....YLVFE.....VLHRD.....ADFGL.....AVDLW.....
125536437	......MSLYA.....GVAHL.....-LVMD.....LLHRD.....CDLGM.....RVDTW.....
125530989	......GCLAA.....IVVVR.....FIVTD.....VMHRD.....CDFGA.....AVDMW.....
125536431	......RCLQA.....LVELR.....YVVME.....VMHRD.....CDFGM.....LVDTW.....
125539108	......MILRR.....VIRLD.....YLVFD.....ILHRD.....GDFGL.....GIDLW.....
125543857	......LILRR.....VVKLE.....YLVFE.....VLHRD.....ADFGL.....GVDLW.....
125542155	......SLLKE.....IVRLH.....YLVFE.....VLHRD.....ADFGL.....PVDMW.....
125542086	......SLLKE.....IVRLH.....GLVFE.....VLHRD.....ADFGL.....PVDMW.....
125547209	......HVLRR.....VVKLE.....YLVFE.....VLHRD.....ADFGL.....AVDLW.....
125559044	......QILRR.....VMKLE.....YLVFE.....IVHRD.....ADFGL.....AVDLW.....
158512937	......ALLAA.....VVALL.....VLVLE.....VVHRD.....ADLGQ.....EVDLW.....
125556593	......LYLAR.....IVHYH.....MLVME.....VVHRD.....CDLGL.....LVDAW.....
125559919	......HILRR.....VIKLE.....YLVFE.....VLHRD.....ADFGL.....AVDLW.....
158512927	......KLLKE.....IIELI.....HLVFE.....VLHRD.....ADFGL.....AVDIW.....
125569409	......LILRR.....IIKLE.....YLVFE.....ILHRD.....ADFGL.....AVDLW.....
125570390	......LILRK.....VIKLE.....YLVFE.....VLHRD.....ADFGL.....GVDLW.....
125562157	......SLLRM.....VVRLL.....YLVFE.....VLHRD.....ADLGL.....PVDIW.....
125573873	......-----.....-----.....FIVTD.....VMHRD.....CDFGS.....AVDMW.....
125579162	......RALEA.....IVQLI.....YIVME.....LMHRD.....CDLGF.....DISGW.....
125573379	......KILKK.....VIQLK.....YMVFE.....VLHRD.....ADFGL.....AVDMW.....
125578805	......KILRT.....VIKLQ.....YLVFE.....VLHRD.....ADFGL.....AVDMW.....
125576732	......LVLRK.....VIKLE.....YLVFE.....VLHRD.....ADFGL.....SVDMW.....
125580625	......SLLKE.....IVRLQ.....YLVFE.....VLHRD.....ADFGL.....PVDMW.....
125574053	......ACQHA.....IVQIK.....FLVLE.....VIHRD.....CDFGA.....AVDMW.....
125586243	......LILRR.....VVKLE.....YLVFE.....VLHRD.....ADFGL.....GVDLW.....
125590816	......NILLS.....IVDVK.....FMVME.....VLHRD.....CDFGL.....AIDMW.....
125584707	......SLLKE.....IVRLH.....YLVFE.....VLHRD.....ADFGL.....PVDMW.....
125582784	......NILLS.....IVEVK.....FMVME.....VLHRD.....CDFGL.....AIDMW.....
125582394	......HVLRR.....VVRLE.....YLVFE.....VLHRD.....ADFGL.....AVDLW.....
125581783	......MILRR.....VIRLD.....YLVFD.....ILHRD.....GDFGL.....GIDLW.....
125594134	......KLLKE.....IIELI.....HLVFE.....VLHRD.....ADFGL.....AVDIW.....
125594115	......KILKK.....VIQLK.....YMVFE.....VLHRD.....ADFGL.....AVDMW.....
125597088	......RILQE.....-TGTY.....SGVEP.....STHGD.....ADIGM.....EVDLW.....

```
125605880    ......SFMAA.....LVGLH.....SLVME.....IIHRD.....CDFGL.....RVDTW......
125601439    ......LILRR.....VIKLD.....YLVFD.....VLHRD.....GDFGL.....GVDLW......
125601964    ......HILRR.....VIKLE.....YLVFE.....VLHRD.....ADFGL.....AVDLW......
125600952    ......QILRR.....VMKLE.....YLVFE.....IVHRD.....ADFGL.....AVDLW......
125813011    ......ALLRK.....IVKLM.....TLVFE.....VIHRD.....ADFGL.....SVDMW......
125823089    ......SLLKD.....IVTLH.....TLVFE.....VLHRD.....ADFGL.....QIDMW......
125829878    ......SLLKN.....IVTLH.....TLVFE.....ILHRD.....ADFGL.....PIDMW......
125840783    ......SLLKG.....IVLLH.....TLVFE.....ILHRD.....ADFGL.....CLDMW......
125852996    ......KILRQ.....IINMK.....YLVFE.....FLHRD.....ADFGL.....AIDVW......
125863342    ......SLLRM.....VVRLI.....YLVFE.....VLHRD.....ADLGL.....AVDMW......
126138566    ......SLMKE.....IVTLY.....TLVFE.....VLHRD.....GDFGL.....SIDIW......
126138792    ......SLLKE.....IVRLY.....YLVFE.....VLHRD.....ADFGL.....GVDMW......
126643891    ......SILRE.....IVSVQ.....NIVFE.....IIHRD.....ADFGL.....SIDIW......
126644757    ......VLLRE.....IVALL.....WLIFE.....ILHRD.....ADFGL.....SVDLW......
126652097    ......KIMNE.....IMGLR.....NLVMD.....IIHRD.....GDFGL.....AVDMW......
126654357    ......ALLRK.....VVETY.....FIILE.....IAHND.....CDFGM.....ASDMW......
126282402    ......SLLKE.....IVSLQ.....YLIFE.....VLHRD.....ADFGL.....SVDIW......
126297584    ......KILQL.....VVNLI.....YLVFD.....ILHRD.....ADFGL.....PIDLW......
126306617    ......NTILK.....IVTVR.....YIVMN.....ILHRD.....GDFGL.....AIDMW......
126304988    ......TLLLR.....IVELK.....FLVMG.....IIHRD.....ADFGL.....SIDMW......
126306725    ......SLLKN.....IVTLH.....TLVFE.....ILHRD.....ADFGL.....PIDMW......
126306723    ......SLLKN.....IVTLH.....TLVFE.....ILHRD.....ADFGL.....PIDMW......
126306619    ......NTILK.....IVTVR.....YIVMN.....ILHRD.....GDFGL.....AIDMW......
126306615    ......NTILK.....IVTVR.....YIVMN.....ILHRD.....GDFGL.....AIDMW......
126306613    ......NTILK.....IVTVR.....YIVMN.....ILHRD.....GDFGL.....AIDMW......
126306621    ......NTILK.....IVTVR.....YIVMN.....ILHRD.....GDFGL.....AIDMW......
126308701    ......SLLKE.....IVRLL.....YLVFE.....VIHRD.....ADFGL.....AVDVW......
126308212    ......KILRQ.....VVNMK.....YLVFE.....FLHRD.....ADFGL.....AIDVW......
126306721    ......SLLKN.....IVTLH.....TLVFE.....ILHRD.....ADFGL.....PIDMW......
126310426    ......ALLRE.....VIALQ.....WLLFD.....VLHRD.....ADMGF.....AIDIW......
126315696    ......KLLQE.....IIGLL.....SLVFD.....ILHRD.....ADFGL.....GVDMW......
126324407    ......KILRQ.....VIHMR.....YLVLE.....FLHRD.....ADFGL.....SIDIW......
126327395    ......ALLRE.....VISLQ.....WLLFD.....VLHRD.....ADMGF.....AIDIW......
126338051    ......SLLKG.....IVLLH.....TFVFE.....ILHRD.....ADFGL.....DLDIW......
126339596    ......SLLKD.....IVTLH.....TLVFE.....VLHRD.....ADFGL.....QIDMW......
126341336    ......SLLKG.....IVLLH.....TLVFE.....ILHRD.....ADFGL.....CLDMW......
126341350    ......AVLRH.....VVRLF.....TLVFE.....VVHRD.....ADFGL.....PVDLW......
126342753    ......SLLKD.....IVTLH.....TLVFE.....VLHRD.....ADFGL.....QIDMW......
126342765    ......KALQE.....VVKLK.....VLAFE.....IVHRD.....ADFGL.....GVDLW......
86611379     ......SLLKE.....IVRLQ.....YLVFE.....VLHRD.....ADFGL.....PVDVW......
134085651    ......SLLRN.....IVTLH.....TLVFE.....ILHRD.....ADFGL.....PLDMW......
133902092    ......SLLRN.....IVSLH.....TFVFE.....ILHRD.....ADFGL.....SLDMW......
133981479    ......SLLRN.....IVSLH.....TFVFE.....ILHRD.....ADFGL.....SLDMW......
148224570    ......SLLKD.....IVTLH.....TLVFE.....VLHRD.....ADFGL.....QIDMW......
156717836    ......SLLKG.....IVLLH.....TLVFE.....ILHRD.....ADFGL.....CLDMW......
134047723    ......SLLKE.....IIKLL.....YMVFE.....VIHRD.....ADFGL.....AVDI-......
145229321    ......SLLRR.....ILTLH.....VLVFE.....ILHRD.....ADFGL.....SIDIW......
154332768    ......IALRH.....TVQLC.....VLVFA.....IIHRD.....TDFGL.....KVDVW......
154332107    ......SLLKE.....VVRLL.....CIVFE.....VVHRD.....ADFGM.....SVDMW......
154334911    ......CLLKE.....VVDLF.....TMIFE.....VVHRD.....GDFGL.....PVDIW......
154337206    ......SLLKE.....IVKLL.....TIVFE.....VLHRD.....GDFGL.....PVDVW......
154340012    ......DLLLR.....IMGAL.....FLVMN.....ILHRD.....CDFGL.....KMDVW......
154335762    ......ELLAA.....IVQVL.....VLLMP.....VLHRD.....IDFGW.....SLDMW......
154339097    ......LIASR.....LVKTI.....AVVME.....ILHRD.....GDFGL.....EVDVW......
154339738    ......TLLHD.....VVRLL.....YLVFE.....ILHRD.....ADFGL.....AADVW......
146082738    ......CLLKE.....VVDLF.....TMIFE.....VVHRD.....GDFGL.....PVDIW......
146089320    ......LIASR.....LVKTI.....AVVME.....ILHRD.....GDFGL.....EVDLW......
146076846    ......SLLKE.....VVRLL.....CIVFE.....VVHRD.....ADFGM.....SVDMW......
154345508    ......SILQE.....IVNLL.....YLVFE.....IIHRD.....ADFGL.....AVDIW......
146094254    ......SLLKE.....IVKLL.....TIVFE.....VLHRD.....GDFGL.....PVDVW......
146091071    ......DLLLR.....IMGAL.....FLVMD.....ILHRD.....CDFGL.....KMDVW......
146078194    ......IALRH.....AVQLC.....VLVFA.....IIHRD.....TDFGL.....KVDVW......
146094537    ......TLLHD.....VVKLL.....YLVFE.....ILHRD.....ADFGL.....AADVW......
146079631    ......ELLAA.....IVQVL.....VMLMP.....VVHRD.....IDFGW.....SLDIW......
145240273    ......QLLRE.....VVPLL.....ILVFP.....IIHRD.....ADFGI.....ALDLW......
145242270    ......ALCSE.....VVQLA.....FMVFE.....VLHRD.....GDLGL.....AVDLW......
145242790    ......SLMKE.....IVSLY.....MLVFE.....VLHRD.....GDFGL.....SIDIW......
145242548    ......KLLQH.....VVSLL.....FMVFE.....VMHRD.....ADFGL.....AVDVW......
145243582    ......SLMKE.....ILRLH.....MLVFE.....ILHRD.....ADFGL.....SIDIW......
145243550    ......SLLKE.....IVRLL.....YLVFE.....ILHRD.....ADFGL.....GVDMW......
145247801    ......KYLQE.....IIALH.....NLVLE.....ILHRD.....ADFGL.....AVDVW......
145252692    ......QTLLE.....VVYLR.....YLVMD.....IMHRD.....ADFGM.....EIDMW......
145253901    ......KLLKM.....ILHLK.....YMVTP.....ILHRD.....ADFGL.....AIDMW......
```

145341649	......KILKK......VVDLK......YLVFE......ILHRD......ADFGL......SVDMW......
145341494	......NILLS......IVNVN......FMVME......IMHRD......CDFGL......AIDVW......
145348413	......KLLQE......VIALV......NLVFE......VLHRD......ADFGL......GVDIW......
145345826	......SLLKE......VVSLL......YLVFE......VLHRD......ADFGL......PVDVW......
145349182	......RILQR......VVRLL......FLVFE......IFHRD......CDFGL......AIDMW......
145352855	......TTLRA......IVHLH......SLAID......IMHRD......ADFGL......AVDIW......
145353846	......SLLQM......IVRLL......YLVFE......VMHRD......ADLGL......PVDMW......
145609287	......KHLQE......IIALR......CLVLE......VLHRD......ADFGL......AVDVW......
145604178	......ALCSE......VIRLV......FMVFE......VLHRD......GDLGL......AVDMW......
145605697	......KLLKL......VLTLE......HMVFP......ILHRD......ADFGL......AIDMW......
145614510	......SLMKE......IVGLH......MLVFE......VLHRD......GDFGL......SIDIW......
145337866	......NTILK......IVTVR......YIVMN......ILHRD......GDFGL......AVDMW......
145559452	......NTILK......IVTVR......YIVMN......ILHRD......GDFGL......AVDMW......
146184947	......SIMQD......IVKLI......SLVME......IIHRD......GDFGS......EIDIW......
146175845	......KLLQK......VLKIN......FLVFD......VIHRD......GDFGL......KSDVW......
146170700	......STLKK......IVILK......LMVFE......IMHRD......SDFGL......TIDIW......
146170271	......SCLKA......VVKLV......YLVFE......IFHRD......ADFGL......PVDVW......
146164872	......SILNS......IIKLK......FMVFD......FMHRD......ADLGL......AVDIW......
146163269	......NILKR......LVKLL......FFVFE......IIHRD......ADFGL......KVDIW......
146161671	......RFMQQ......IVKIV......MIVMP......ILHRD......TDFGL......KSDMW......
146419727	......SLCRE......IIKVV......YLIFE......IFHRD......GDLGL......AIDLW......
146419725	......ALVKK......VLLYL......VLVTP......IIHRD......GDFGI......EVDMW......
146422716	......KLLQS......VLGLL......FMVSD......IIHRD......ADFGL......EVDIW......
146417586	......KYLQE......VIELI......NLVLE......ILHRD......ADFGL......AIDIW......
146412706	......TILKK......VIEII......YTVCL......YLHRD......ADFGL......AVDLW......
146413719	......SLLKE......IVRLY......YLVFE......VLHRD......ADFGL......GVDMW......
146413753	......SLMKE......IVTLY......TLVFE......VLHRD......GDFGL......SIDIW......
149248104	......TILKQ......ILTIK......YTVSP......YLHRD......ADFGL......SVDLW......
149247340	......KYLQE......VIELI......NLVLE......ILHRD......ADFGL......AVDIW......
149238782	......SLLKE......IVRLY......YLVFE......VLHRD......ADFGL......GVDIW......
149240669	......KLLQS......IVGLL......YMIFD......IIHRD......ADFGL......EVDIW......
149244914	......SLMKE......IVTLY......TLVFE......VLHRD......GDFGL......SIDIW......
147768420	......NILLS......IVDVK......YMVME......VLHRD......CDFGL......AIDMW......
147781538	......EALQV......VVVLH......VLVLE......IVHRD......ADFGQ......EIDLW......
147788188	......VILRR......VVKLE......YLVFE......VLHRD......ADFGL......GVDLW......
147806286	......HVLRK......VMKLE......YLVFE......VLHRD......GDFGL......SVDLW......
147816223	......SLLRM......VVRLM......YLVFE......VLHRD......ADLGL......AVDMW......
147838772	......KLLKE......IIELI......HLVFE......VLHRD......ADFGL......GVDVW......
147843679	......SLLKE......IVRLQ......YLVFE......VLHRD......ADFGL......PVDVW......
147852277	......HVLRR......IIKLE......YLVFE......VLYRD......ADFGL......VVDLW......
148707363	......KLLQK......TIGLL......SLVLD......ILHRD......ADFVL......GMDMW......
148668478	......KLLQE......IIGLL......SLVFD......----D......ADFGL......GVDMW......
148673007	......ALLRE......VIALQ......WLLFD......VLHRD......ADMGF......AIDIW......
148676621	......KILQL......VVNLI......YLVFD......ILHRD......ADFGL......PIDLW......
148683071	......NTILK......IVTVR......YIVMN......ILHRD......GDFGL......AVDMW......
148682688	......SLLKG......IVLLH......TLVFE......ILHRD......ADFGL......CLDMW......
148679773	......TLLLR......IVELK......FLVMG......IIHRD......ADFGL......SIDMW......
148679771	......TLLLR......IVELK......FLVMG......IIHRD......ADFGL......SIDMW......
148684175	......KILRQ......VVNMK......YLVFE......FLHRD......ADFGL......AIDVW......
148683072	......NTILK......IVTVR......YIVMN......ILHRD......GDFGL......AVDMW......
148689600	......SLLKD......IVTLH......TLVFE......VLHRD......ADFGL......QIDMW......
148725865	......AVLRQ......VVRLF......TLVFE......VVHRD......ADFGL......PVDLW......
148728180	......TLLLR......IVELK......FLVMG......IIHRD......ADFGL......SIDMW......
156100953	......SCLKN......ILRLI......FAAYE......MCHRD......GDLGL......NVDIW......
156098003	......QQLKQ......QTSLA......WMVFE......IIHRD......ADFGL......SVDMW......
156097013	......NIMRI......VVCLL......YLPLK......VIHRD......ADFGL......SVDIW......
156098111	......NILLQ......ILSVK......YLVME......VMHRD......CDFGM......KIDIW......
156096843	......KIMKE......IMTAL......NLVME......FMHRD......ADFGL......SVDMW......
149024815	......NTILK......IVTVR......YIVMN......ILHRD......GDFGL......AVDMW......
149032519	......KILRQ......IINMK......YLVFE......FLHRD......ADFGL......AIDVW......
149038436	......TLLLR......IVELK......FLVMG......IIHRD......ADFGL......SIDMW......
158254369	......TLLLR......IVELK......FLVMG......IIHRD......ADFGL......SIDMW......
149044383	......SLLKD......IVTLH......TLVFE......VLHRD......ADFGL......QIDMW......
149059202	......KLLQE......IIG--......VL---......ILHRD......ADFGL......GVDMW......
149067189	......SLLKD......IVTLH......TLVFE......VLHRD......ADFGL......QIDMW......
149241266	......ALLRR......VVRLM......TLVFE......IVHRD......ADFGL......PVDMW......
149242354	......SLLKE......IVKLL......YLVFE......VLHRD......ADFGL......AVDIW......
150951564	......TILRQ......ILTIV......YTVSP......FLHRD......ADFGL......AVDLW......
150865162	......KLLQS......IVGLL......YMIFD......VIHRD......ADFGL......EVDIW......
150864190	......KYLQE......VIELV......NLVLE......ILHRD......ADFGL......AVDIW......
150865392	......SLCRE......ITKLV......YMIFE......IFHRD......GDLGL......AIDLW......
149410017	......SLLKE......IVCLQ......YLIFE......VLHRD......ADFGL......PVDIW......

```
149417861   ......SLLKD......IVTLH......TLVFE......VLHRD......ADFGL......QIDMW......
149487257   ......NTILK......IVTVR......SLSIL......ILHRD......GDFGL......AIDMW......
149498658   ......SLLKN......IVTLH......TLVFE......ILHRD......ADFGL......PIDMW......
149584761   ......KLLQE......IIGLL......SLVFD......ILHRD......ADFGL......GVDMW......
149598956   ......ALLRE......VISLQ......WLLFD......VLHRD......ADMGF......AIDIW......
149614700   ......KALQE......VVKLK......VLAFE......IMHRD......ADFGL......GVDLW......
149620801   ......SLLKG......IVLLH......TLVFE......ILHRD......ADFGL......CLDMW......
149637958   ......SLLKD......IVTLH......TLVFE......VLHRD......ADFGL......QIDMW......
149690265   ......SLLKE......IVSLQ......YLIFE......VLHRD......ADFGL......PVDIW......
149701821   ......TLLLR......IVELK......FLVMG......IIHRD......ADFGL......SIDMW......
149705947   ......AVLRH......VVRLF......TLVFE......VVHRD......ADFGL......PVDLW......
149705453   ......SLLKG......IVLLH......TLVFE......ILHRD......ADFGL......CLDMW......
149715226   ......ALLRR......VVRLM......TLVFE......IVHRD......ADFGL......PVDMW......
149723546   ......SLLKE......IVRLL......YLVFE......VIHRD......ADFGL......AVDVW......
149730040   ......ALLRE......VISLQ......WLLFD......VLHRD......ADMGF......AIDIW......
149732672   ......KLLQE......IIGLL......SLVFD......ILHRD......ADFGL......GVDMW......
149743173   ......SLLKD......IVTLH......TLVFE......VLHRD......ADFGL......QIDMW......
149744592   ......SLLKD......IVTLH......TLVFE......VLHRD......ADFGL......QIDMW......
149754997   ......SLLKG......IVLLH......TFVFE......VLHRD......ADFGL......ELDIW......
149756563   ......SLLKE......IVKLL......YLVFE......VLHRD......ADFGL......AVDIW......
149758334   ......NTILK......IVTVR......YIVMN......ILHRD......GDFGL......AVDMW......
149758336   ......NTILK......IVTVR......YIVMN......ILHRD......GDFGL......AVDMW......
154278786   ......KYLQE......IIALH......NLVLE......VLHRD......ADFGL......AVDIW......
154285298   ......RLLQH......VVSLQ......FMVFE......VLHRD......ADFGL......AVDIW......
154270995   ......QILLE......IVHLR......YLVMD......IMHRD......ADFGM......EIDMW......
154280054   ......SLMKE......IVSLY......MLVFE......VLHRD......GDFGL......SIDIW......
154276474   ......RILSA......VIPLL......ILVFP......IIHRD......ADFGI......SLDMW......
154317886   ......KLLQS......IVTLQ......FMVFE......VLHRD......ADFGL......AVDIW......
154311513   ......QTLMD......IVRLR......FLVLD......ILHRD......ADFGM......SVDMW......
154292618   ......SLLKE......IVRLL......YLVME......VLHRD......ADFGL......GVDMW......
154294282   ......ALCSE......VIRLI......FMVFE......VLHRD......GDLGL......AIDMW......
154312796   ......SLMKE......IVSLH......MLVFE......VLHRD......ADFGL......SIDIW......
154321191   ......KHLQE......VISLL......NLVLE......VLHRD......ADFGL......AVDIW......
154312114   ......KTLKL......VLSLE......YMVTP......ILHRD......ADFGL......AIDIW......
157109150   ......QIMRR......IVKLK......NLVLE......ICHRD......CDFGS......KIDVW......
109033272   ......QIMRK......IVRLR......NLVLD......ICHRD......CDFGS......SIDVW......
109033269   ......QIMRK......IVRLR......NLVLD......ICHRD......CDFGS......SIDVW......
109033266   ......QIMRK......IVRLR......NLVLD......ICHRD......CDFGS......SIDVW......
109124957   ......QIMRK......IVRLR......NLVLE......VCHRD......CDFGS......SIDVW......
109124955   ......QIMRK......IVRLR......NLVLE......VCHRD......CDFGS......SIDVW......
169612169   ......QIMRI......IVELK......NLVLE......ICHRD......CDFGS......KIDVW......
110809647   ......QIMQD......IVQLQ......NVVME......VCHRD......CDFGS......SVDIW......
116007128   ......QIMRK......IVKLL......NLVLE......ICHRD......CDFGS......KIDVW......
115398504   ......QIMRI......IVELK......NLVLE......ICHRD......CDFGS......KIDVW......
114588709   ......QIMRK......IVRLR......NLVLD......ICHRD......CDFGS......SIDVW......
114588713   ......QIMRK......IVRLR......NLVLD......ICHRD......CDFGS......SIDVW......
114588711   ......QIMRK......IVRLR......NLVLD......ICHRD......CDFGS......SIDVW......
114677509   ......QIMRK......IVRLR......NLVLE......VCHRD......CDFGS......SIDVW......
114677511   ......QIMRK......IVRLR......NLVLE......VCHRD......CDFGS......SIDVW......
114677507   ......QIMRK......IVRLR......NLVLE......VCHRD......CDFGS......SIDVW......
115644344   ......QIMRR......IVKLK......NLVLE......ICHRD......CDFGS......DIDVW......
116057022   ......QIMKA......VVKLI......NLVLE......ICHRD......CDFGS......AIDIW......
169853815   ......QIMRL......VVDLK......NLVLE......ICHRD......CDFGS......NIDIW......
117646987   ......QTMRL......VVALK......NLVLE......VCHRD......CDFGS......AIDIW......
118083461   ......QIMRK......IVRLR......NLVLD......ICHRD......CDFGS......SIDVW......
90079059    ......QIMRK......IVRLR......NLVLE......VCHRD......CDFGS......SIDVW......
121703542   ......QIMRI......IVELK......NLVLE......ICHRD......CDFGS......KIDVW......
119467830   ......QIMRI......IVELK......NLVLE......ICHRD......CDFGS......KIDVW......
120538392   ......QIMRK......IVRLR......NLVLD......VCHRD......CDFGS......NIDIW......
123397881   ......AILKR......ILSLK......NLVTD......IVHRD......GDFGN......PIDIW......
123424134   ......TMIQA......CLKLH......YIFTD......ITHRD......CDFGS......KIDVW......
123426576   ......DIMKK......CIKLK......YIVME......ICHRD......CDFGS......SIDIW......
123435865   ......EILKI......CIKLK......NIVMD......IVHRD......TDFGS......AIDVW......
123455468   ......KVFTR......CIKMR......NIVME......YIHRD......CDFGS......PIDIW......
123456926   ......KVMLR......CVKLR......NIVME......YIHRD......CDFGS......PMDIW......
123975997   ......DILLS......IVKVY......QIVME......IAHCD......ADFGS......AVDIW......
123476292   ......QLVSR......LVYIG......ILITD......IAHRD......CDFGS......SIDVW......
123478209   ......EVLKA......CLSLH......FLVIE......IAHRD......CDFGS......EIDIW......
123493199   ......QITPL......CLHCY......HLVTD......LCHRD......CDFGS......KIDVW......
```

```
123478645    ......ETLQT......CLTLI......NLVTE......ICHRD......CDFGS......AIDIW......
154414715    ......DIIST......CITLR......NIVMD......IVHRD......CDFGS......PIDIW......
154414405    ......ETMQE......CIRLC......NLVMD......VTHRD......CDFGS......AIDIW......
145483037    ......LIIQE......VVMLR......NLVME......ICHRD......CDFGS......QIDMW......
145485648    ......EIHLN......IAPLR......HLVMD......VMHRD......ADFGS......KINIW......
145486652    ......LIIQE......VIKLK......NLVMD......ICHRD......CDFGS......AIDTW......
145494470    ......ANLKL......IIPLK......NFVME......MAHRD......CDFGS......NIDIW......
145495011    ......LIIQE......IVKLR......NLVMD......ICHRD......CDFGS......SIDIW......
145502412    ......QILQE......VLKMK......NVVME......ICHRD......CDFGS......QIDVW......
145500860    ......QILQE......VLKMK......NVVME......ICHRD......CDFGS......QIDVW......
145508139    ......LIIQE......VIKLK......NLVMD......ICHRD......CDFGS......AIDVW......
145506627    ......QILQE......IVETK......NVIMD......ICHRD......CDFGS......QVDMW......
145510522    ......YYLKQ......IIELK......NMLYE......ITHRD......CDFNS......QVDIW......
145510248    ......EILFE......IVKIL......NIVFE......IAHRD......CDFGS......SIDIW......
145509807    ......EILLL......IIQIE......NIIFE......VVHRD......CDFGS......EIDIW......
145514876    ......ANLKL......IVALK......NYVME......MAHRD......CDFGS......SIDIW......
145513969    ......ANLKL......IVALK......NYVME......MAHRD......CDFGS......SIDIW......
145517236    ......QILQE......VLKMK......HVVME......ICHRD......CDFGS......QVDVW......
145518884    ......QILQE......VLKMK......NVVME......ICHRD......CDFGS......QIDVW......
145518191    ......QILQE......IVETK......NVIMD......ICHRD......CDFGS......QVDMW......
145525160    ......QIMQE......IVETK......NVIMD......VCHRD......CDFGS......QVDMW......
145528959    ......EILMQ......VVKML......NIILE......IAHRD......CDFGS......SIDIW......
145530163    ......QILQE......VVKLK......NVVMD......ICHRD......CDFGS......QIDVW......
145528678    ......LIIQE......IVKLR......NLVMD......ICHRD......CDFGS......SIDIW......
145541203    ......QILQE......VVKLK......NVVMD......ICHRD......CDFGS......QIDVW......
145540716    ......LIIQE......VVMLR......NLVME......ICHRD......CDFGS......QIDMW......
145548940    ......EIHTN......IAPLR......HLVMD......IMHRD......ADFGS......KIDIW......
145553367    ......EILFE......VVKIL......NIVFE......IVHRD......CDFGS......SVDIW......
145473859    ......LIIQE......VIKLK......NLVMD......ISHRD......CDFGS......AIDVW......
145549047    ......LIIQE......VVMLR......NLVME......ICHRD......CDFGS......QIDMW......
145548451    ......LIIQE......VIKLK......NLVMD......ICHRD......CDFGS......AIDVW......
145549580    ......EILFE......IVKIL......NIVFE......IAHRD......CDFGS......SIDIW......
125524842    ......QLMRA......VICLK......NLVME......VCHRD......CDFGS......SIDIW......
125525508    ......QTMQV......VACLK......NLVLE......VCHRD......CDFGS......AIDVW......
125525208    ......QTMRL......VVALK......NLVLE......VCHRD......CDFGS......AIDIW......
125538742    ......QIMRS......VVSLK......NLVME......VCHRD......CDFGS......SIDIW......
125550739    ......QTMRV......VVSLK......NLVLE......VCHRD......CDFGS......AIDVW......
125555677    ......QIMRS......VISLK......NLVME......VCHRD......CDFGS......SIDIW......
125569752    ......QTMRL......VVALK......NLVLE......VCHRD......CDFGS......AIDIW......
125569452    ......QLMRA......VICLK......NLVME......VCHRD......CDFGS......SIDIW......
125570032    ......QTMQV......VACLK......NLVLE......VCHRD......CDFGS......AIDVW......
125575433    ......QTMQL......VVQLK......NLVLE......VCHRD......CDFGS......AIDIW......
125581429    ......QIMRS......VVSLK......NLVME......VCHRD......CDFGS......SIDIW......
125588588    ......QIMHM......IVGLK......NLVLE......ICHRD......CDFGS......AIDLW......
112253401    ......HILSE......IVRLN......NLVLE......VVHSD......CDFGS......AIDLW......
125804489    ......QIMRK......IVRLR......NLVMD......ICHRD......CDFGS......SIDIW......
125593192    ......QLMRA......VISLK......NLVME......VCHRD......CDFGS......SIDIW......
126645708    ......DIMKV......IIKLV......NVIME......ICHRD......CDFGS......SIDLW......
126325739    ......QIMRK......IVRLR......NLVLD......ICHRD......CDFGS......SIDVW......
126325737    ......QIMRK......IVRLR......NLVLD......ICHRD......CDFGS......SIDVW......
154335429    ......QIMQD......IVQLQ......NVVME......VCHRD......CDFGS......AVDIW......
146083815    ......QIMQD......IVQLQ......NVVME......VCHRD......CDFGS......SVDIW......
145250085    ......QIMRI......IVELK......NLVLE......ICHRD......CDFGS......KIDVW......
90797273     ......QTMRV......VVALK......NLVLE......VCHRD......CDFGS......AIDVW......
145345251    ......QIMKA......VVKLI......NLVLE......ICHRD......CDFGS......AIDIW......
143330602    ......NIIQR......IVEFR......HLVME......VCHRD......CDFGS......AIDVW......
145607095    ......QIMRI......IVQLK......NLVQE......ICHRD......CDFGS......KIDVW......
146185034    ......EILLD......YVDLY......NIVFE......IAHRD......CDFGG......KIDMW......
146181217    ......EIIER......IIKCK......NLVME......ICHRD......CDFGS......AIDMW......
146181519    ......EIIKS......LIKLK......NIVME......ICHRD......CDFGS......AIDTW......
146419721    ......QIMKL......VVDMK......NLILE......ICHRD......CDFGS......KIDVW......
146417859    ......ELLRV......IVSLR......NLVME......ISHRD......CDFGS......KIDIW......
149248260    ......QIMKL......IADLK......NLILE......ICHRD......CDFGS......KIDVW......
149236181    ......EILRV......IVSLR......NLVME......ISHRD......CDFGS......KIDIW......
147767229    ......QTMRL......VVSLK......NLVLE......VCHRD......CDFGS......AIDIW......
147785084    ......QTMRL......IVSLK......NLVLE......VCHRD......CDFGS......AIDMW......
148692338    ......QIMRK......IVRLR......NLVLE......VCHRD......CDFGS......SIDVW......
157909770    ......QIMRK......IVRLR......NLVLD......VCHRD......CDFGS......NIDIW......
```

```
156094209    ......IIMQS......IIFLK......NVVME......ICHRD......CDFGS......HIDLW......
156097835    ......EIMKK......IVKLK......HMIME......ITHRD......CDFNT......AIDTW......
156102791    ......DLLQK......IVNLK......NIVFK......LAHRD......CDLGS......DVDLW......
155372335    ......QIMRK......IVRLR......NLVLD......ICHRD......CDFGS......SIDVW......
150865505    ......EILRL......IVSLR......NLVME......ISHRD......CDFGS......KIDIW......
150865389    ......QIMKL......IADLK......NLILE......ICHRD......CDFGS......KIDVW......
149562735    ......QIMRK......IVRLR......NLVLD......ICHRD......CDFGS......SIDVW......
149731291    ......QIMRK......IVRLR......NLVLD......ICHRD......CDFGS......SIDVW......
149731289    ......QIMRK......IVRLR......NLVLD......ICHRD......CDFGS......SIDVW......
154294657    ......QIMRI......IVELK......NLVQE......ICHRD......CDFGS......KIDVW......
150408741    ......QTMQL......VVQLK......NLVLE......VCHRD......CDFGS......AIDIW......
110468069    ......QTMRL......VVSLK......NLVLE......VCHRD......CDFGS......AIDVW......
108860807    ......KLLRL......IVEIK......YVVFE......VYHRD......CDFGL......AIDIW......
115465609    ......KLLRL......IVEIK......YVIFE......VFHRD......CDFGL......AIDIW......
108864228    ......KLLRL......IVEIK......YVVFE......VFHRD......CDFGL......AIDIW......
157138129    ......KILTR......IIDIR......YIVQC......VLHRD......CDFGL......SIDIW......
157118743    ......RMLKH.....IIGLL.....YLVTH.....IIHRD.....LDFGL.....TVDIW......
157119344    ......KLMKL.....IIGLL.....YLVME.....IIHRD.....LDFGL.....NVDIW......
157119346    ......KLLQT.....IIKLL.....YLFTE.....IIHRD.....LDFGL.....NVDIW......
157107527    ......KMLCF.....VLSAL.....YVITE.....ILHRD.....CDFGL.....AVDVW......
109070894    ......RLLKH.....VIGLL.....YLVTH.....IIHRD.....LDFGL.....TVDIW......
109074888    ......VLMKC.....IISLL.....YLVME.....IIHRD.....LDFGL.....NAD--......
109074880    ......VLMKC.....IISLL.....YLVME.....IIHRD.....LDFGL.....NVDMW......
109081156    ......KIIRR.....IVKVF.....YIVQE.....VLHRD.....GDFGL.....AIDMW......
109089011    ......VLMKC.....IIGLL.....YIVME.....IIHRD.....LDFGL.....NVDIW......
109089017    ......VLMKC.....IIGLL.....YIVME.....IIHRD.....LDFGL.....NVDLW......
109089013    ......VLMKC.....IIGLL.....YIVME.....IIHRD.....LDFGL.....NVDLW......
109094636    ......RLLKH.....VIGLL.....YLVTT.....IIHRD.....LDFGL.....TVDIW......
109094634    ......RLLKH.....VIGLL.....YLVMP.....---RD.....LDFGL.....TVDIW......
109113587    ......KILKH.....IIAIK.....YVVLD.....VIHRD.....GDFGM.....AIDLW......
109113866    ......KMLCF.....VLSAL.....YVVTE.....ILHRD.....CDFGL.....AIDIW......
109113597    ......KILKH.....IIAIK.....YVVLD.....VIHRD.....GDFGM.....AIDLW......
109122049    ......KIIRR.....IVKVF.....YIVQE.....VLHRD.....GDFGL.....AIDMW......
109122202    ......KIIRR.....IVKVY.....YIVQE.....VLHRD.....GDFGL.....AIDMW......
109122200    ......KIIRR.....IVKVY.....YIVQE.....VLHRD.....GDFGL.....AIDMW......
109122204    ......KIIRR.....IVKVY.....YIVQE.....VLHRD.....GDFGL.....AIDMW......
109122196    ......KIIRR.....IVKVY.....YIVQE.....VLHRD.....GDFGL.....AIDMW......
109122051    ......KIIRR.....IVKVF.....YIVQE.....VLHRD.....GDFGL.....AIDMW......
109158061    ......RLLKH.....VIGLL.....YLVTH.....IIHRD.....LDFGL.....TVDIW......
109497729    ......KMLCF.....VLSAL.....YVVTE.....ILHRD.....CDFGL.....AIDIW......
109482717    ......RLLKH.....VIGLL.....YLVTT.....IIHRD.....LDFGL.....TVDIW......
109482715    ......RLLKH.....VIGLL.....YLVTT.....IIHRD.....LDFGL.....TVDIW......
109503050    ......VLMKC.....IIGLL.....YIVME.....IIHRD.....LDFGL.....IVDLW......
109503710    ......VLMKC.....IIGLL.....YIVME.....IIHRD.....LDFGL.....NVDTW......
109506789    ......KIIRR.....IVKVY.....YIVQE.....VLHRD.....GDFGL.....AIDMW......
109094632    ......RLLKH.....VIGLL.....YLVMP.....IIHRD.....LDFGL.....TVDIW......
110180210    ......KLLSH.....VIKIK.....YIVYE.....VLHRD.....CDFGL.....AIDIW......
110180218    ......KLLRL.....IVEIK.....YVVFE.....VFHRD.....CDLGL.....AIDMW......
110180222    ......KLLRL.....IVEIK.....YVVFE.....VYHRD.....CDFGL.....AIDIW......
110180206    ......KLLKH.....VIAII.....HIVYE.....VLHRD.....GDFGL.....AIDIW......
110180192    ......KLLKH.....VIAVK.....YIVYE.....VLHRD.....GDFGL.....AIDIW......
110180204    ......KLLRH.....IVAIR.....YIVNE.....VLHRD.....CDFGL.....AIDVW......
110180196    ......KLLRH.....IVAIR.....YIAYE.....VLHRD.....CDFGL.....AIDVW......
110180186    ......KLLRH.....VIALK.....YLVYE.....ILHRD.....CDFGL.....SIDVW......
110180200    ......KLLRL.....VVEIK.....YVVFE.....VFHRD.....CDFGL.....AIDIW......
110180202    ......KLLRL.....IVEIK.....YVVFE.....VFHRD.....CDFGL.....AIDIW......
110180194    ......KLLRH.....VVVIK.....YIVFE.....VLHRD.....TDFGL.....AIDVW......
110180190    ......KLLRH.....IIAIR.....YISTE.....IIHRD.....CDFGL.....AIDVW......
110180220    ......KLLRL.....IVEIK.....YVVFE.....VYHRD.....CDFGL.....AIDVW......
110180214    ......KLLRL.....VVEIK.....YVVFE.....VFHRD.....CDFGL.....AIDIW......
110180224    ......KLLRL.....IVEIK.....YVVFE.....VYHRD.....CDFGL.....AIDIW......
110180212    ......KLLRH.....VISLK.....YLVYE.....ILHRD.....CDFGL.....SIDVW......
110180208    ......KLLRH.....VITIK.....YIVYE.....ILHRD.....GDFGL.....AIDIW......
110180198    ......KLLRH.....VIALK.....YLVYE.....ILHRD.....CDFGL.....SIDVW......
110180216    ......KLLRL.....IVEIK.....YVVFE.....VFHRD.....CDFGL.....AIDIW......
110180188    ......KLLRH.....VVALK.....YLVYE.....ILHRD.....CDFGL.....SIDVW......
110590383    ......KILLR.....IIGIN.....YIVQD.....VLHRD.....CDFGL.....SIDIW......
110645378    ......VLMKC.....IISLL.....YLVME.....IIHRD.....LDFGL.....NVDIW......
110739389    ......KLLRL.....IVEIK.....YVVFE.....VFHRD.....CDLGL.....AIDMW......
110755131    ......KMLCF.....VLSAL.....YVITE.....ILHRD.....CDFGL.....AVDVW......
110756046    ......MFLLS.....IIRLI.....YLVFE.....VIHRD.....ADFGL.....GIDMW......
110758787    ......RMLKH.....IIGLL.....YLVTH.....IIHRD.....LDFGL.....TVDIW......
```

```
71980112     ......KLLRH.....VIGIR.....YISTE.....VIHRD.....CDFGL.....AIDIW......
110809637    ......DIMCF.....VIGYF.....HIVMP.....IIHRD.....ADMGL.....KIDIW......
110832261    ......KLLRL.....IVEIK.....YVVFE.....VYHRD.....CDFGL.....AIDIW......
110832259    ......KLLRH.....VIALK.....YLVYE.....ILHRD.....CDFGL.....SIDVW......
110832257    ......KVLRL.....IVVIK.....YVVFE.....VFHRD.....CDFGL.....AIDIW......
110832255    ......KLLRH.....IIGIR.....YIATE.....VIHRD.....CDFGL.....AIDVW......
118403626    ......KILKH.....IIAIK.....YVVLD.....VLHRD.....GDFGM.....AIDMW......
111380705    ......KLLRY.....IISIL.....YLIQE.....VLHRD.....CDFGL.....AIDVW......
113930697    ......KILKH.....IIAIK.....YVVLD.....VIHRD.....GDFGM.....AIDLW......
86279640     ......MFLQE.....IIKLL.....YLIFE.....VIHRD.....CDFGL.....GVDMW......
115439029    ......KLLRL.....IVEIK.....YVVFE.....VYHRD.....CDFGL.....AIDIW......
115465485    ......KLLRL.....IVEIK.....YVVFE.....VYHRD.....CDFGL.....AIDTW......
115465679    ......KLLRL.....IVEIK.....YVVFE.....VYHRD.....CDFGL.....AIDIW......
115495537    ......KIIQR.....IVKVY.....YIVQE.....VLHRD.....GDFGL.....AIDMW......
115400293    ......KLLKK.....LIGLC.....YLVTE.....VIHRD.....CDFGL.....EVDIW......
115389850    ......KLLQH.....ITCLY.....YLYEE.....VLHRD.....CDFGL.....AIDVW......
115389978    ......KLLRY.....IISIL.....YLIQE.....VLHRD.....CDFGL.....AIDVW......
114603870    ......VLLKC.....IISLL.....YLVME.....IIHRD.....LDFGL.....NVDIW......
114603876    ......VLLKC.....IISLL.....YLVME.....IIHRD.....LDFGL.....NVDIW......
114603878    ......VLLKC.....IISLL.....YLVME.....---RD.....LDFGL.....NVDIW......
114603882    ......VLLKC.....IISLL.....YLVME.....IIHRD.....LDFGL.....NGDL-......
114625405    ......KMLCF.....VLSAL.....YVVTE.....ILHRD.....CDFGL.....AIDIW......
114635091    ......VLMKC.....IIGLL.....YIVME.....IIHRD.....LDFGL.....NVDLW......
114635085    ......VLMKC.....IIGLL.....YIVME.....----D.....LDFGL.....NVDIW......
114635089    ......VLMKC.....IIGLL.....YIVME.....IIHRD.....LDFGL.....NVDIW......
114661963    ......QILLR.....VIGIR.....YIVQD.....VLHRD.....CDFGL.....SIDIW......
114668738    ......KILKH.....IIAIK.....YVVLD.....VIHRD.....GDFGM.....AIDLW......
114796146    ......KLLRH.....VICLK.....YLVSE.....ILHRD.....CDFGL.....SIDVW......
115311606    ......QILLR.....VIGIR.....YIVQD.....VLHRD.....CDFGL.....SIDIW......
121923978    ......KLLKH.....IISLS.....YFVTE.....VVHRD.....CDFGL.....EVDIW......
115383345    ......KILSR.....IINIQ.....YIVQC.....VLHRD.....CDFGL.....SIDIW......
115545488    ......VLMKC.....IISLL.....YLVME.....IIHRD.....LDFGL.....NVDIW......
115678918    ......ILMKL.....IIGLL.....YLVME.....IIHRD.....LDFGL.....NVDIW......
115737419    ......ICLQE.....IVKLL.....YLVFE.....VIHRD.....ADFGL.....GVDMW......
115741915    ......RLLKH.....VISLL.....YMVTH.....VIHRD.....LDFGL.....KVDIW......
115905867    ......KMLFF.....VSTCM.....YVVTE.....ILHRD.....CDFGL.....AVDMW......
115911473    ......KILRH.....VIAIR.....YVVFD.....VIHRD.....GDFGM.....AVDVW......
116055034    ......HLLRR.....VIKLV.....YLVYE.....VLHRD.....CDFGL.....SIDMW......
116057755    ......VALHR.....VCTLR.....YLSFE.....IWHRD.....CDFGS.....SVDVW......
116059075    ......TLLRH.....IVEVL.....FIVFE.....VFHRD.....CDFGL.....AIDIW......
116059852    ......KLLRH.....VIDII.....YLMYD.....VLHRD.....CDFGL.....AIDVW......
91718897f    ......QILLR.....VIGIR.....YIVQD.....VLHRD.....CDFGL.....SIDIW......
154310447    ......KLLQH.....ITCLY.....YLYEE.....VLHRD.....CDFGL.....AIDVW......
118780573    ......KLMKL.....IIGLL.....YLVME.....IIHRD.....LDFGL.....NVDIW......
169861842    ......RCLDH.....LTLFV.....YSVVC.....IIHTD.....IDFDW.....GIDIF......
169851340    ......KLLKF.....IISIL.....YLIQE.....VIHRD.....CDFGL.....AIDVW......
169844236    ......KLLKF.....IISIL.....YFIQE.....IVHRD.....CDFGL.....AIDIW......
169847319    ......RLLHH.....ITCLY.....YLYEE.....VLHRD.....CDFGL.....AIDVW......
169845357    ......KLLKH.....IISLS.....YFVTE.....VVHRD.....CDFGL.....AVDIW......
116666756    ......VLMKC.....IISLL.....YLVME.....IIHRD.....LDFGL.....NVDIW......
116668022    ......KIIRR.....IVKVF.....YIVQE.....VLHRD.....GDFGL.....AIDMW......
117616346    ......QILLR.....VIGIR.....YIVQD.....VLHRD.....CDFGL.....SIDIW......
117616348    ......KILLR.....IIGIN.....YIVQD.....VLHRD.....CDFGL.....SIDIW......
118082195    ......RLLKH.....VIGIL.....YLVMP.....IIHRD.....LDFGL.....TVDIW......
118087463    ......MFLQE.....IIKLL.....YLVFE.....VIHRD.....CDFGL.....GVDMW......
118090182    ......VLMKC.....IISLL.....YLVME.....IIHRD.....LDFGL.....NVDMW......
118092741    ......VLMKC.....IIGLL.....YIVME.....IIHRD.....LDFGL.....NVDLW......
118092739    ......VLMKC.....IIGLL.....YIVME.....IIHRD.....LDFGL.....NVDIW......
118100435    ......KMLCF.....VLSAL.....YVVTE.....ILHRD.....CDFGL.....AIDIW......
118102270    ......RLLKH.....VIGLL.....YLVTH.....IIHRD.....LDFGL.....TVDIW......
118102288    ......TLLKQ.....VIGLL.....YLVMP.....IIHRD.....LDFGL.....TVDIW......
118102268    ......RLLKH.....VIGLL.....YLVTH.....IIHRD.....LDFGL.....TVDIW......
118345322    ......KLLRY.....IISIL.....YLIQE.....VLHRD.....CDFGL.....AIDVW......
118600947    ......KIIRR.....IVKVY.....YIVQE.....VLHRD.....GDFGL.....AIDMW......
68303938     ......KLLKY.....IVSIV.....YLIQE.....VIHRD.....CEFGL.....AIDVW......
122692401    ......RLLKH.....VIGLL.....YLVTT.....IIHRD.....LDFGL.....TVDIW......
119389443    ......KILKH.....IITIF.....YIIQE.....VIHRD.....CDFGL.....AMDVW......
119389382    ......VLMKC.....IISLL.....YLVME.....IIHRD.....LDFGL.....NVDIW......
119389523    ......RLLKH.....VIGLL.....YLVTH.....IIHRD.....LDFGL.....TVDIW......
119389520    ......RLLKH.....VIGLL.....YLVTH.....IIHRD.....LDFGL.....TVDIW......
119389522    ......RLLKH.....VIGLL.....YLVTH.....IIHRD.....LDFGL.....TVDIW......
121700140    ......KLLRY.....IISIL.....YLIQE.....VLHRD.....CDFGL.....AIDVW......
```

121701399	......KLLKH......IISLS......YFVTE......VVHRD......CDFGL......EVDIW......
121708701	......KLLQH......ITCLY......YLYEE......VLHRD......CDFGL......AIDVW......
121713066	......KLLKY......LICLR......YIATE......VIHRD......CDFGL......QVDIW......
119479499	......RLLKY......LICLR......YIATE......VIHRD......CDFGL......QVDVW......
119472832	......KLLRY......IISIL......YLIQE......VLHRD......CDFGL......AIDVW......
119499844	......KLLQH......ITCLY......YLYEE......VLHRD......CDFGL......AIDVW......
119495366	......KLLKH......IISLS......YFVTE......VVHRD......CDFGL......EVDIW......
119370006	......RLLKH......VIGLL......YLVTH......IIHRD......LDFGL......TVDIW......
119370004	......RLLKH......VIGLL......YLVTH......IIHRD......LDFGL......TVDIW......
119571446	......KMLCF......VLSAL......YVVTE......ILHRD......CDFGL......AIDIW......
149408126	......KMLCF......VLSAL......YVVTE......ILHRD......CDFGL......AIDIW......
149642961	......RLLKH......VIGLL......YLVMP......VIHRD......LDFGL......TVDIW......
158138507	......QILLR......VIGIR......YIVQD......VLHRD......CDFGL......SIDIW......
119602609	......TLLQE......ISLL......YLVFE......VVHRD......CDFGL......GVDMW......
119613543	......VLMKC......IIGLL......YIVME......IIHRD......LDFGL......NVDLW......
119613537	......VLMKC......IIGLL......YIVME......IIHRD......LDFGL......NVDIW......
119624272	......RLLKH......VIGLL......YLVTH......---D......LDFGL......TVDIW......
119918672	......VLMKC......IIGLL......YIVME......IIHRD......LDFGL......NAD--......
119927467	......KIIRR......IVKVF......YIVQE......VLHRD......GDFGL......AIDMW......
154318036	......KLLKH......VISLS......YFVTE......VVHRD......CDFGL......EVDVW......
123425671	......RILSS......ITNIT......IVVSD......ILHRD......TDFGL......AIDIW......
123436996	......CILSH......ITNLI......IIIMD......VLHRD......TDFGL......ALDVW......
123446420	......KILSQ......ITNLV......AIVMD......ILHRD......ADFGL......PMDMW......
123480001	......TLLRQ......IVGLL......YLAFE......VIHRD......CDFGL......GIDMW......
123479755	......CILSH......ITNLI......IVVMD......VLHRD......GDFGL......QLDVW......
123481925	......TFLRQ......IVSLL......YLVFE......IIHRD......CDFGL......GVDMW......
154412658	......TFLRQ......IVKLL......YMVFE......VIHRD......CDFGL......SVDMW......
154416524	......TFLRQ......IVDLL......YVVFE......LIHRD......CDFGL......GVDMW......
122894104	......KLLRH......VIAVR......YIAAE......VIHRD......CDFGL......AIDVW......
123996459	......RLLKH......VIGLL......YLVTT......IIHRD......LDFGL......TVDIW......
76162777	......RLIRH......VLSLL......YMTVD......VLHRD......CDLGL......GVDLW......
76162772	......MYLQE......IIRLL......YLVFD......LLHRD......CDFGL......SVDMW......
76162770	......RLLGH......ITRLH......YIITE......VLHRD......CDFGL......AIDVW......
76162774	......RLMRH......VVSVL......YIVTD......IIHRD......CDFGL......AIDLW......
145475159	......CFLQQ......IVKLL......YMVFE......LIHRD......ADFGL......SVDMW......
145477295	......IFLEQ......IIKLT......YMVFD......LIHRD......ADFGL......AVDMW......
145479369	......KLLQF......IIGLV......YIVTE......VMHRD......CDLGL......AIDIW......
145476645	......KILRN......IVNLI......YCVTE......ILHRD......CDFGL......AVDIW......
145479753	......CILRE......LIQTI......YVVME......VLHRD......CDFGL......AIDVW......
145485456	......KILRN......IVNLI......YCVTE......ILHRD......CDFGL......AVDIW......
145486232	......CFLSQ......FVCLD......YLVFE......LIHRD......ADFGL......SVDMW......
145487468	......IYLQQ......IVKLL......YMIFE......LIHRD......ADFGL......SVDMW......
145489139	......RILRT......ILRLE......YLVTN......IVHRD......CDFGL......AIDMW......
145489237	......MFLQE......VVRLL......YLVFD......LIHRD......ADFGL......AVDMW......
145487302	......RLLRL......IIGIN......YVVQE......VVHRD......CDFGL......SVDVW......
145486377	......MFLQE......IVRLL......YLVFD......LIHRD......ADFGL......AVDMW......
145488322	......IFLEQ......IIKLN......YMVFE......LIHRD......ADFGL......AVDMW......
145494566	......KLLKF......IISLF......YIVTE......VIHRD......CDLGL......AVDIY......
145494169	......MFLQE......IVRLL......YLVFD......LIHRD......ADFGL......AVDMW......
145493954	......MFLQE......VIRMT......YIVFD......IIHRD......ADFGL......AVDMW......
145491251	......MFLQE......IVRLL......YLVFD......LIHRD......ADFGL......AVDMW......
145499431	......IFLEQ......IIRLT......YMVFD......VIHRD......ADFGL......AVDIW......
145497907	......CFLQQ......IVKLL......YMVFE......LIHRD......ADFGL......SVDMW......
145500052	......MFLEQ......IIKLT......YMVFD......LIHRD......ADFGL......AVDMW......
145496609	......KIQRS......ILKIY......YIVSQ......SIHRD......CDFGF......PVDIW......
145496818	......MFLQE......VVRLL......YLVFD......LIHRD......ADFGL......AVDMW......
145502383	......MFLQE......VIRMT......YIVFD......IIHRD......ADFGL......AVDMW......
145503097	......RILRA......IVAIK......YLVQE......ILHRD......CDFGL......PIDIW......
145504414	......KIQRS......ILKIY......YIVSP......SIHRD......CDFGF......PVDIW......
145505684	......KIQRS......ILKIY......YIVSP......SIHRD......CDFGF......PVDIW......
145509032	......MFLYE......IVKLY......YLVFE......LLHRD......ADFGL......GVDMW......
145505085	......LIQRQ......ILQIY......YIVSE......SIHRD......CDFGF......PVDVW......
145506234	......KILRL......IVELK......YMVTE......ILHRD......CDFGL......AVDIW......
145509673	......KLLKF......IISLL......YIVTE......VIHRD......CDLGL......AVDIY......
145513492	......SFLKQ......IIQLI......YLIFE......MIHRD......ADFGL......GVDMW......
145512231	......KILRL......VVDLK......YMVMD......ILHRD......CDFGL......AVDIW......
145512934	......KLLQF......VISLV......YIITE......VMHRD......CDLGL......AIDIW......
145513304	......SILRE......LIQII......YVVME......VLHRD......CDFGL......AIDVW......
145515557	......IYLQQ......IVKLL......YMTFE......LIHRD......ADFGL......AVDMW......

145515655	......KILRL......IVELK......YMVTE......ILHRD......CDFGL......AVDMW......
145521238	......KILRN......IVNLI......YCVTE......ILHRD......CDFGL......AVDIW......
145523642	......RILRA......IVAIK......YLVQE......IIHRD......CDFGL......PIDIW......
145527728	......CFLQQ......IVKLL......YMVFE......LIHRD......ADFGL......SVDMW......
145534183	......KLLQF......VISLF......YIITE......VMHRD......CDLGL......AIDIW......
145533845	......TILRE......LIQII......YVVME......VLHRD......CDFGL......AIDVW......
145533691	......SFLKQ......IIQLI......YLIFE......MIHRD......ADFGL......GVDMW......
145537690	......TFLKQ......IVSII......YIVFE......MIHRD......ADFGL......GIDLW......
145535989	......IFLEQ......IIKLL......YMVFE......IIHRD......ADFGL......AVDMW......
145536720	......KLLKF......IISLV......YIITD......VIHRD......CDLGL......AVDIY......
145536590	......MFLQE......IVRLL......YLVFD......LIHRD......ADFGL......AVDMW......
145535712	......MFLYE......IIKLY......YLVFE......LLHRD......ADFGL......GVDMW......
145541560	......IFLEQ......IIKLL......YMVFE......IIHRD......ADFGL......AVDMW......
145538680	......MFLQE......VIRMT......YIVFD......IIHRD......ADFGL......AVDMW......
145540850	......TFLKQ......IVSII......YIVFE......MIHRD......ADFGL......GIDLW......
145539878	......CILRE......LIQII......YVVME......VLHRD......CDFGL......AIDVW......
145540042	......SLLKK......IVSLI......YMAFE......MIHRD......ADFGL......GVDMW......
145546334	......RLLRL......IIGIN......YVVQE......VVHRD......CDFGL......SVDVW......
145544332	......KLLKF......IVSLL......YIITD......IIHRD......CDFGL......SVDIY......
145546378	......KILRL......IVELK......YMVTE......ILHRD......CDFGL......AVDMW......
145546063	......KLLKF......IVKLL......YLVFQ......IIHRD......GDLNL......AIDIW......
145544134	......MFLQE......IVRLL......YLVFD......LIHRD......ADFGL......AVDMW......
145551173	......RILRT......ILHLE......YLVTN......IVHRD......CDFGL......AIDIW......
145550802	......RILRT......ILHLE......YLVTN......IVHRD......CDFGL......AIDIW......
145548730	......KILRN......IVNLI......YCVTE......ILHRD......CDFGL......AVDIW......
145551803	......KLLRF......IVKLL......YLVFQ......IIHRD......GDLNL......AIDIW......
124294722	......KLVRH......VVQIM......YVVYE......ILHRD......CDFGL......AIDVW......
125527035	......KLLRL......IVEIK......YIIFE......VFHRD......CDFGL......AIDIW......
125527168	......KLLRL......IVEIK......YVVFE......VYHRD......CDFGL......AIDIW......
125526938	......KLLRL......IVEIK......YVVFE......VYHRD......CDFGL......AIDIW......
125537986	......KVLRL......IVVIK......YVVFE......VFHRD......CDFGL......AIDIW......
125538081	......KLLRH......VIALK......YLVYE......ILHRD......CDFGL......SIDVW......
125550810	......KLLRL......IVEIK......YVVFE......VFHRD......CDFGL......AIDIW......
125543399	......KLLRH......IIGIR......YIATE......VIHRD......CDFGL......AIDVW......
125553344	......KLLRL......IVEIK......YVVFE......VYHRD......CDFGL......AIDTW......
125553482	......KLLRL......IVEIK......YVVFE......VYHRD......CDFGL......AIDIW......
125560202	......KLLRH......VISIK......YIVYE......VLHRD......GDFGL......AIDIW......
125555287	......KLLRL......IVEIK......YVVFE......VYHRD......CDFGL......AIDIW......
125556702	......KLLRL......IVEIK......YVVFE......VFHRD......CDFGL......AIDIW......
125576836	......KLLRL......IVEIK......YVVFE......VFHRD......CDFGL......AIDIW......
125580809	......KLLRH......VIALK......YLVYE......ILHRD......CDFGL......SIDVW......
125571491	......KLLRL......IVEIK......YVVFE......VYHRD......CDFGL......AIDIW......
125571267	......KLLRL......IVEIK......YVVFE......VYHRD......CDFGL......AIDIW......
125596091	......KLLRH......IVAIR......YIAYE......VLHRD......CDFGL......AIDVW......
125595356	......KLLRL......IVEIK......YVVFE......VYHRD......CDFGL......AIDIW......
125602244	......KLLRH......VISIK......YIVYE......VLHRD......GDFGL......AIDIW......
160333575	......VLMKC......IIGLL......YLVME......IIHRD......LDFGL......NVDVW......
125838215	......KMLCF......VLSAL......YVVTE......ILHRD......CDFGL......AIDIW......
125851667	......RLLRH......VICLL......YMVMP......IIHRD......LDFGL......TVDVW......
126031620	......KILLR......IIGIN......YIVQD......VLHRD......CDFGL......SIDIW......
126031626	......VLMKC......IISLL......YLVME......IIHRD......LDFGL......NVDIW......
126644254	......AILSR......IVKIL......YLVLE......IYHRD......CDFGL......AIDMW......
126644803	......KLLRQ......ILGII......YIVTQ......IIHRD......CDFGL......AVDIW......
126644158	......MILTE......IVNLL......YLVFD......LLHRD......ADFGL......GIDMW......
126272855	......VLMKC......IIGLL......YIVME......IIHRD......LDFGL......NVDIW......
126277582	......KIIRR......IVKVF......YIVQE......VLHRD......GDFGL......AIDMW......
126272857	......VLMKC......IIGLL......YIVME......IIHRD......LDFGL......NVDLW......
126290058	......KILLR......INRIN......YIVQD......ILHRD......CDFGL......SIDIW......
126291096	......VLLKC......IISLL......YLVME......IIHRD......LDFGL......NVDIW......
126282977	......KIIRR......IVKVF......YIVQE......VLHRD......GDFGL......AIDMW......
126291093	......VLLKC......IISLL......YLVME......IIHRD......LDFGL......NVDIW......
126309779	......RLLKH......VIGLL......YLVTH......IIHRD......LDFGL......TVDIW......
126309913	......MLLKH......VIGLL......YLVMP......VVHRD......LDFGL......TVDIW......
126309777	......RLLKH......VIGLL......YLVTH......IIHRD......LDFGL......TVDIW......
126313889	......KMLCF......VLSAL......YVVTE......ILHRD......CDFGL......AIDIW......
126320715	......KIIRR......IVKVY......YIVQE......VLHRD......GDFGL......AIDMW......
126323017	......MFLQE......IIRLL......YLVFE......VIHRD......CDFGL......GVDMW......
126320713	......KIIRR......IVKVY......YIVQE......VLHRD......GDFGL......AIDMW......
126324784	......KILLR......IIGIN......YIVQD......VLHRD......CDFGL......SIDIW......
126334010	......KILKH......IIAIK......YVVLD......VIHRD......GDFGM......AIDLW......
126335546	......QILLR......VIGIC......YIVQD......VLHRD......CDFGL......SIDIW......
126338967	......RLLKH......VIGLL......YLVMP......IVHRD......LDFGL......TVDIW......

126338985	......RLLKH.....VIGLL.....YLVTT.....IIHRD.....LDFGL.....TVSCW.....
121483467	......KLLQH.....ITCLY.....YLYEE.....VLHRD.....CDFGL.....AIDVW.....
145230127	......KLLQH.....ITCLY.....YLYEE.....VLHRD.....CDFGL.....AIDVW.....
154331589	......EILQN.....VVGCR.....YVAMD.....VLHRD.....CDFGL.....ASDVW.....
154333061	......RLLNH.....ILGLR.....YLVTE.....VVHRD.....CDFNL.....LVDVW.....
154333141	......DMMRF.....LLNVV.....YVVTP.....VAHRD.....IDFGL.....AVDIW.....
154333896	......QLMMS.....VMSAT.....YVVMK.....VIHRD.....TDFGQ.....GVDVW.....
154341927	......LLLRI.....ILRLI.....YLVTD.....MVHRD.....CDFGL.....PIDIW.....
154343978	......KLLAH.....IIGLR.....YIVMD.....VIHRD.....CDFGL.....QIDVW.....
154336056	......EIMTS.....LIRLH.....YFVMD.....VMHRD.....CDFGL.....AVDVW.....
154344118	......DIMSF.....VIGYF.....HIVMP.....IIHRD.....ADMGL.....QIDIW.....
154341445	......KLLAY.....ILSLK.....YVVTD.....VLHRD.....GDFGL.....SADMW.....
154346712	......MLLQQ.....VVDIL.....YLVFE.....VIHRD.....GDFGL.....AMDMW.....
146094056	......LLLRI.....VLRLI.....YLVTD.....IVHRD.....CDFGL.....PIDIW.....
146075791	......EILQN.....VVGCR.....YVAMD.....VLHRD.....CDFGL.....ASDVW.....
146093544	......KLLAY.....ILSLK.....YVVTD.....VLHRD.....GDFGL.....SADMW.....
146080852	......QLMMA.....VMSAT.....YVVMK.....VIHRD.....TDFGQ.....GVDVW.....
146079138	......DMMRF.....LLNVV.....YVVTP.....VAHRD.....IDFGL.....AVDIW.....
146103309	......MFLHR.....IIKLL.....YLVFE.....ILHRD.....ADFGL.....GVDMW.....
146079023	......RLLNH.....ILGLR.....YLVTE.....VVHRD.....CDFNL.....LVDMW.....
146097823	......KLLAH.....IIGLR.....YIVMD.....VIHRD.....CDFGL.....QIDVW.....
146102593	......MLLQR.....IVGIL.....YLVFE.....IIHRD.....GDFGL.....AMDMW.....
154345540	......MFLHR.....IIKLL.....YLVFE.....ILHRD.....ADFGL.....GVDMW.....
146097931	......DIMCF.....VIGYF.....HIVMP.....IIHRD.....ADMGL.....RIDIW.....
145237666	......KLLKH.....LIGLS.....YLVTD.....VIHRD.....CDFGL.....QVDIW.....
145240111	......KLLKH.....IISLS.....YFVTE.....VVHRD.....CDFGL.....EVDIW.....
145241049	......KLLRY.....IISIL.....YLIQE.....VLHRD.....CDFGL.....AIDVW.....
145255493	......KLLKH.....LIGLC.....YLVTE.....VIHRD.....CDFGL.....EVDLW.....
134105020	......VLMKC.....IISLL.....YLVME.....IIHRD.....LDFGL.....NVDIW.....
134034948	......KLLKH.....VISLS.....YFVTE.....VVHRD.....CDFGL.....EVDIW.....
141795792	......VLMKC.....IISLL.....YLVME.....IIHRD.....LDFGL.....NVDIW.....
145349268	......TLLRH.....IVEVL.....FIVFE.....VFHRD.....CDFGL.....AIDIW.....
145350942	......KLLRH.....VIDII.....YLMYD.....VLHRD.....CDFGL.....AIDVW.....
145350146	......HLLRR.....VIKLE.....YLVYE.....VLHRD.....CDFGL.....SIDMW.....
145580327	......RLLKH.....VIGLL.....YLVTH.....IIHRD.....LDFGL.....TVDIW.....
146185358	......KLLRL.....VIGLE.....YCVSE.....VIHRD.....CDFGL.....AMDVW.....
146185355	......RLLRL.....IIGIN.....YVVSE.....VVHRD.....CDFGL.....KVDVW.....
146177486	......RILRY.....ILQLS.....YLVTN.....IIHRD.....CDFGL.....AVDIW.....
116242306	......KLLRH.....IVAIR.....YIAYE.....VLHRD.....CDFGL.....AIDVW.....
116242308	......KLLRH.....VIGLR.....YIATE.....VLHRD.....CDFGL.....AIDVW.....
134254740	......KLLRH.....VIAIK.....YIVYE.....VLHRD.....GDFGL.....AIDIW.....
134254744	......KLLSH.....IIKIK.....YIVYE.....VLHRD.....CDFGL.....AIDIW.....
134254742	......KLLRH.....VIALK.....YLVYE.....ILHRD.....CDFGL.....SIDVW.....
146414215	......KLLKH.....LITLD.....YFVTE.....VIHRD.....CDFGL.....EVDLW.....
146416277	......KLLKH.....IISVL.....YFIQE.....VLHRD.....CDFGL.....AIDVW.....
149246025	......KLLQF.....ITCLL.....YVYEE.....VLHRD.....GDFGL.....AIDVW.....
149248820	......KLLKH.....LITLD.....YFVNE.....VIHRD.....CDFGL.....EVDLW.....
149238758	......KLLRK.....IIRLY.....YLIQE.....VIHRD.....CDFGL.....AIDLW.....
149234583	......KLLKH.....IISIL.....YLIQE.....VLHRD.....CDFGL.....AIDVW.....
147781744	......KLLRH.....VIALK.....YLVYE.....ILHRD.....CDFGL.....SIDVW.....
147786894	......KLLRH.....VIVLK.....YIVYE.....VLHRD.....GDFGL.....AIDIW.....
147807972	......KLLRH.....VIALK.....YLVYE.....ILHRD.....CDFGL.....SIDVW.....
74231016	......KLLRH.....VIASK.....YLVYE.....ILHRD.....CDFGL.....SIDVW.....
148665019	......KILLR.....IIGIN.....YIVQD.....VLHRD.....CDFGL.....SIDIW.....
148665020	......KILLR.....IIGIN.....YIVQD.....VLHRD.....CDFGL.....SIDIW.....
148685478	......QILLR.....VIGIR.....YIVQD.....VLHRD.....CDFGL.....SIDIW.....
148692906	......VLMKC.....IIGLL.....YIVME.....IIHRD.....LDFGL.....NVDIW.....
148688303	......VLMKC.....IISLL.....YLVME.....IIHRD.....LDFGL.....NVDIW.....
148692911	......VLMKC.....IIGLL.....YIVME.....IIHRD.....LDFGL.....NVDLW.....
148690640	......RLLKH.....VIGLL.....YLVTH.....IIHRD.....LDFGL.....TVDIW.....
148692907	......VLMKC.....IIGLL.....YIVME.....IIHRD.....LDFGL.....NVDLW.....
148690639	......RLLKH.....VIGLL.....YLVTH.....IIHRD.....LDFGL.....TVDIW.....
148690637	......RLLKH.....VIGLL.....YLVTH.....IIHRD.....LDFGL.....TVDIW.....
148690643	......LLLKH.....VIGLL.....YLVMP.....IVHRD.....LDFGL.....TVDIW.....
148688304	......VLMKC.....IISLL.....YLVME.....IIHRD.....LDFGL.....NVDIW.....
148697564	......MLLKE.....IIRLL.....YLVFE.....VIHRD.....CDFGL.....GVDMW.....
149944747	......KILKH.....IIAIK.....YVVLD.....VIHRD.....GDFGM.....AIDLW.....
156101932	......MFLHQ.....IITLM.....YLVFD.....LLHRD.....GDFGL.....GVDMW.....
156098454	......TILNR.....IIRLR.....YIVLE.....IIHRD.....CDFGL.....SIDIW.....
148886592	......KLLKQ.....LIGLC.....YLVTE.....VIHRD.....CDFGL.....MVDIW.....
148886593	......KLLKK.....LIGLC.....YLVTE.....VIHRD.....CDFGL.....EVDIW.....
148886841	......KLLKH.....IISLS.....YFVTE.....VVHRD.....CDFGL.....EVDIW.....

148886844	......KLLKH.....IISLS.....YFVTE.....VVHRD.....CDFGL.....EVDVW......
148886843	......KLLKH.....VISLS.....YFVTE.....VVHRD.....CDFGL.....EVDIW......
149034130	......VLMKC.....IIGLL.....YIVME.....IIHRD.....LDFGL.....NIDQW......
149034131	......VLMKC.....IIGLL.....YIVME.....IIHRD.....LDFGL.....NIDQW......
149043483	......RLLKH.....VIGLL.....YLVTH.....IIHRD.....LDFGL.....TVDIW......
149046752	......VLMKC.....IISLL.....YLVME.....IIHRD.....LDFGL.....NVDIW......
149043487	......LLLKH.....VIGLL.....YLVMP.....IVHRD.....LDFGL.....TVDIW......
149053556	......KMLCF.....VLSAL.....YVVTE.....ILHRD.....CDFGL.....AIDIW......
149243200	......RLLKH.....VIGLL.....YLVTH.....IIHRD.....LDAGL.....TVDIW......
149243426	......RLLKH.....VIGLL.....YLVTH.....IIHRD.....LDRGL.....TVDIW......
150866354	......KLLSR.....IVQLY.....YIIQE.....VIHRD.....CDFGL.....EIDVW......
150951385	......KLLKH.....IISIL.....YLIQE.....VLHRD.....CDFGL.....AIDVW......
150866199	......KFMRH.....V--NL.....YCYQE.....VIHRD.....CDFGL.....AVDLW......
150866723	......KLLQF.....ITCLL.....YLYEE.....VLHRD.....CDFGL.....AIDIW......
150864140	......KLLKH.....LITLD.....YFVNE.....VIHRD.....CDFGL.....EVDLW......
149409754	......KIIRR.....IVKVY.....YVVQE.....VLHRD.....GDFGL.....AIDMW......
149411999	......VLMKC.....IISLL.....YLVME.....IIHRD.....LDFGL.....NVDMW......
149412116	......VLLKC.....IISLL.....YLVME.....IIHRD.....LDFGL.....NVDIW......
149412114	......VLLKC.....IISLL.....YLVME.....IIHRD.....LDFGL.....NVDIW......
149505461	......KIIRR.....IVKVF.....YIVQE.....VLHRD.....GDFGL.....AIDMW......
149632952	......KMLCF.....VLSAL.....YVVTE.....ILHRD.....CDFGL.....AIDIW......
149640591	......LLLKH.....VIGLL.....YLVMP.....VIHRD.....LDFGL.....TVDIW......
149691933	......KIIRR.....IVKVF.....YIVQE.....VLHRD.....GDFGL.....AIDMW......
149701504	......VLMKC.....IISLL.....YLVME.....IIHRD.....LDFGL.....NVDIW......
149720224	......KILLR.....IIGIN.....YIVQD.....VLHRD.....CDFGL.....SIDIW......
149726022	......VLLKC.....IISLL.....YLVME.....IIHRD.....LDFGL.....NVDIW......
149726020	......VLLKC.....IISLL.....YLVME.....IIHRD.....LDFGL.....NVDIW......
149732465	......RLLKH.....VIGLL.....YLVTH.....IIHRD.....LDFGL.....TVDIW......
149759303	......RLLKH.....VIGLL.....YLVTT.....IIHRD.....LDFGL.....TVDIW......
56417685	......LLLKH.....VIGLL.....YLVMP.....----D.....LDFGL.....TVDIW......
150387832	......KLLKH.....IISLS.....YFVTE.....VVHRD.....CDFGL.....EVDIW......
154271632	......KLLKH.....IISLS.....YFVTE.....VVHRD.....CDFGL.....EVDVW......
154275466	......KLLQH.....ITCLY.....YLYEE.....VLHRD.....CDFGL.....AIDVW......
154277902	......KLLRY.....IISIL.....YLIQE.....VLHRD.....CDFGL.....AIDVW......
157119189	......DTLKK.....CLRLF.....CMVLE.....ILHAD.....CDFGS.....GIDMW......
157136011	......NLLEM.....IVRLK.....CLVFE.....IIHCD.....VDFGS.....AIDMW......
157136013	......NLLEM.....IVRLK.....CLVFE.....IIHCD.....VDFGS.....AIDMW......
157123429	......RILKH.....IIHMY.....CITFE.....IIHCD.....IDFGS.....AIDMW......
157119423	......RILDE.....VIHML.....CISFE.....IIHCD.....IDFGS.....PIDMW......
109014457	......SILSR.....FVRSY.....CLVFE.....LIHAD.....IDFGS.....AIDMW......
109014451	......SILSR.....FVRSY.....CLVFE.....LIHAD.....IDFGS.....AIDMW......
109014463	......SILSR.....FVRSY.....CLVFE.....LIHAD.....IDFGS.....AIDMW......
109014466	......SILSR.....FVRSY.....CLVFE.....LIHAD.....IDFGS.....AIDMW......
109014454	......SILSR.....FVRSY.....CLVFE.....LIHAD.....IDFGS.....AIDMW......
109014460	......SILSR.....FVRSY.....CLVFE.....LIHAD.....IDFGS.....AIDMW......
109018589	......RILEH.....VIHML.....CMAFE.....IIHCD.....IDFGS.....PIDIW......
109018595	......RILEH.....VIHML.....CMAFE.....IIHCD.....IDFGS.....PIDIW......
109018591	......RILEH.....VIHML.....CMAFE.....IIHCD.....IDFGS.....PIDIW......
109065345	......RLLEL.....IVHLK.....CLVFE.....IIHCD.....VDFGS.....AIDMW......
109065343	......RLLEL.....IVHLK.....CLVFE.....IIHCD.....VDFGS.....AIDMW......
109065347	......RLLEL.....IVHLK.....CLVFE.....IIHCD.....VDFGS.....AIDMW......
109065341	......RLLEL.....IVHLK.....CLVFE.....IIHCD.....VDFGS.....AIDMW......
109065349	......RLLEL.....IVHLK.....CLVFE.....IIHCD.....VDFGS.....AIDMW......
109068454	......SILAR.....FVRAY.....CLVFE.....LIHAD.....IDFGS.....AIDMW......
109069479	......EFLKK.....CLRLF.....CLVFE.....ILHAD.....CDFGS.....GIDMW......
109095130	......KILEA.....VVHMK.....CITFE.....IIHCD.....IDFGS.....AIDMW......
109095132	......KILEA.....VVHMK.....CITFE.....IIHCD.....IDFGS.....AIDMW......
109097694	......RILEH.....VIHML.....CMTFE.....IIHCD.....IDFGS.....PIDMW......
109097698	......RILEH.....VIHML.....CMTFE.....IIHCD.....IDFGS.....PIDMW......
109106860	......SILAR.....FVRAY.....CLVFE.....LIHAD.....IDFGS.....AIDMW......
109106856	......SILAR.....FVRAY.....CLVFE.....LIHAD.....IDFGS.....AIDMW......
109106858	......SILAR.....FVRAY.....CLVFE.....LIHAD.....IDFGS.....AIDMW......
109124799	......KLLHC.....VIRFL.....YLVFE.....IIHAD.....IDFGS.....KVDVW......
109124707	......RLLEL.....IVHLK.....CLVFE.....IIHCD.....VDFGS.....AIDMW......
109124797	......KLLHC.....VIRFL.....YLVFE.....IIHAD.....IDFGS.....KVDVW......
109124705	......RLLEL.....IVHLK.....CLVFE.....IIHCD.....VDFGS.....AIDMW......
109124703	......RLLEL.....IVHLK.....CLVFE.....IIHCD.....VDFGS.....AIDMW......
158508564	......SILSR.....FVRSY.....CLVFE.....LIHAD.....IDFGS.....AIDMW......
109472656	......KILEA.....VVHMK.....CITFE.....IIHCD.....IDFGS.....AIDMW......
110751243	......SILSR.....FVRAY.....CLVFE.....LIHAD.....IDFGS.....AIDMW......
169609715	......NEEDP.....IIRLL.....CIVFE.....VIHLD.....ADFGT.....PVDMW......
118403794	......RLLEL.....IVHLK.....CLVFE.....IIHCD.....VDFGS.....AIDMW......
115454989	......KLLKY.....LLRLY.....LIVCE.....LIHCD.....IDLGS.....KIDMW......
115433362	......NLLQK.....VVNFT.....CISTE.....VIHCD.....IDFGS.....PIDMW......

115386532	......TADPS......IIRLV......CLVYE......IVHTD......VDFGL......QADIW......
115396242	......GILEQ......LIRFE......CMVFE......ILHAD......CDLGS......AIDVW......
115387751	......SVLDL......LLRLK......CLVFE......LIHCD......IDFGS......AIDMW......
114558707	......SILSR......FVRSY......CLVFE......LIHAD......IDFGS......AIDMW......
114558701	......SILSR......FVRSY......CLVFE......LIHAD......IDFGS......AIDMW......
114558709	......SILSR......FVRSY......CLVFE......LIHAD......IDFGS......AIDMW......
114558703	......SILSR......FVRSY......CLVFE......LIHAD......IDFGS......AIDMW......
114558705	......SILSR......FVRSY......CLVFE......LIHAD......IDFGS......AIDMW......
114572217	......RILEH......VIHML......CMAFE......IIHCD......IDFGS......PIDIW......
114572219	......RILEH......VIHML......CMAFE......IIHCD......IDFGS......PIDIW......
114572221	......RILEH......VIHML......CMAFE......IIHCD......IDFGS......PIDIW......
114572223	......RILEH......VIHML......CMAFE......IIHCD......IDFGS......PIDIW......
114572215	......RILEH......VIHML......CMAFE......IIHCD......IDFGS......PIDIW......
114605847	......EFLKK......CLRLF......CLVFE......ILHAD......CDFGS......GIDMW......
114616285	......SILAR......FVRAY......CLVFE......LIHAD......IDFGS......AIDMW......
114616283	......SILAR......FVRAY......CLVFE......LIHAD......IDFGS......AIDMW......
114616289	......SILAR......FVRAY......CLVFE......LIHAD......IDFGS......AIDMW......
114616287	......SILAR......FVRAY......CLVFE......LIHAD......IDFGS......AIDMW......
114616281	......SILAR......FVRAY......CLVFE......LIHAD......IDFGS......AIDMW......
114636845	......SILAR......FVRAY......CLVFE......LIHAD......IDFGS......AIDMW......
114636851	......SILAR......FVRAY......CLVFE......LIHAD......IDFGS......AIDMW......
114636847	......SILAR......FVRAY......CLVFE......LIHAD......IDFGS......AIDMW......
114642933	......KILEA......VVHMK......CITFE......IIHCD......IDFGS......AIDMW......
114642935	......KILEA......VVHMK......CITFE......IIHCD......IDFGS......AIDMW......
114642931	......KILEA......VVHMK......CITFE......IIHCD......IDFGS......AIDMW......
114643890	......RILEH......VIHML......CMTFE......IIHCD......IDFGS......PIDMW......
114643888	......RILEH......VIHML......CMTFE......IIHCD......IDFGS......PIDMW......
115683741	......RILEH......LIHMF......CITFE......IIHCD......IDFGS......PIDMW......
115694469	......RLLEL......IVKLK......CLVFE......IIHCD......IDFGS......AIDMW......
115735112	......RLLEL......IVKLK......CLVFE......IIHCD......IDFGS......AIDMW......
115751068	......KILDA......VVHMR......CITFE......IIHCD......IDFGS......PIDMW......
115757202	......EYLKR......CVRLF......CLVFE......ILHAD......CDFGS......AIDMW......
115908399	......KILDA......VVHMR......CITFE......IIHCD......IDFGS......PIDMW......
115945419	......RILEH......LIHMF......CITFE......IIHCD......IDFGS......PIDMW......
116000791	......DILQK......CVRFL......FMVFE......VVHAD......CDFGS......PMDLW......
116056100	......DFLRE......CVRLL......CLVFE......IIHCD......IDFGS......GIDMW......
116057150	......KLLKM......ILRML......FIVSE......LIHCD......IDFGS......KVDVW......
116057802	......KTLER......VVQMF......CVVLE......VVHCD......IDFGS......PIDMW......
116060261	......GILRT......IVELA......CLVFE......VVHCD......VDFGS......PIDMW......
118404398	......GILAR......FVRAY......CLVFE......LIHAD......IDFGS......AIDMW......
118404554	......SILAR......FVRAY......CLVFE......LIHAD......IDFGS......AIDMW......
169868904	......RALDP......IVKLE......CLVFE......IMHAD......CDLGS......ALDVW......
169857308	......QAVEV......FVRFT......FLVFE......ITHGD......CFFGS......KTDIF......
169847906	......TILEM......LLRLR......CLVFE......LIHCD......IDFGS......AIDMW......
169853631	......KILDN......VLKMT......CIATE......IVHCD......IDFGS......AIDMW......
117616786	......EFLKK......CLRLF......CLVFE......ILHAD......CDFGS......GIDMW......
117616740	......KILEA......VVHMK......CITFE......IIHCD......IDFGS......AIDMW......
117616990	......KLLRC......VIRFL......YLVFE......IIHAD......IDFGS......KVDVW......
118082890	......SILAR......FVRAY......CLVFE......LIHAD......IDFGS......AIDMW......
118086455	......EFLKK......CLRLF......CLVFE......ILHAD......CDFGS......GIDMW......
118091150	......SILAR......FVRAY......CLVFE......LIHAD......IDFGS......AIDMW......
118102503	......SILSR......FVRSY......CLVFE......LIHAD......IDFGS......AIDMW......
121698200	......KIYEH......STHLY......CLVLQ......ITHTD......CDFGE......AVDIW......
121704485	......KQLDH......IRKAL......CLVQK......LVHTD......VDFGS......PVDIW......
121705588	......AILEQ......VIRFE......CMVFE......ILHAD......CDLGS......AIDVW......
121710904	......NLLQK......VVNFT......CISTE......VIHCD......IDFGS......PIDMW......
121710522	......EILRH......ITILL......CLVME......IIHTD......CDWGS......EVDIW......
121714455	......SVLDL......LLRLK......CLVFE......LIHCD......IDFGS......AIDMW......
121720112	......RIEGG......AVRLL......CLVHP......IIHAD......CDFGS......SVDIW......
119473887	......EIYNH......CLRLI......CLVHP......VIHTD......CDLGE......KVDIW......
119497909	......NLLQK......VVNFT......CISTE......VIHCD......IDFGS......PIDMW......
119487130	......SVLDL......LLRLK......CLVFE......LIHCD......IDFGS......AIDMW......
119491823	......GILEQ......VIRFE......CMVFE......ILHAD......CDLGS......AIDVW......
119577322	......RLLEL......IVHLK......CLVFE......IIHCD......VDFGS......AIDMW......
119892358	......RILEH......VIHML......CMTFE......IIHCD......IDFGS......PIDMW......
119893121	......KILEA......VVHMK......CITFE......IIHCD......IDFGS......AIDMW......
119630109	......RLLEL......IVHLK......CLVFE......IIHCD......VDFGS......AIDMW......
164420691	......SILAR......FVRAY......CLVFE......LIHAD......IDFGS......AIDMW......
119604335	......SILAR......FVRAY......CLVFE......LIHAD......IDFGS......AIDMW......
119604332	......SILAR......FVRAY......CLVFE......LIHAD......IDFGS......AIDMW......
119604334	......SILAR......FVRAY......CLVFE......LIHAD......IDFGS......AIDMW......
119609248	......KILEA......VVHMK......CITFE......IIHCD......IDFGS......AIDMW......
119630111	......RLLEL......IVHLK......CLVFE......IIHCD......VDFGS......AIDMW......
119630108	......RLLEL......IVHLK......CLVFE......IIHCD......VDFGS......AIDMW......
119907687	......SILAR......FVRAY......CLVFE......LIHAD......IDFGS......AIDMW......

```
119907685    ......SILAR.....FVRAY.....CLVFE.....LIHAD.....IDFGS.....AIDMW.....
125630665    ......RLLEL.....IVHLK.....CLVFE.....IIHCD.....VDFGS.....AIDMW.....
119910539    ......KLLRC.....IIRFL.....YLVFE.....IIHAD.....IDFGS.....KVDVW.....
120537328    ......RLLEL.....IVHLK.....CLVFE.....IIHCD.....VDFGS.....AIDMW.....
123701398    ......RLLEL.....IVHLK.....CLVFE.....IIHCD.....VDFGS.....AIDMW.....
123373427    ......KRDKT.....ACKYV.....FTVTE.....MCHGD.....IDFGG.....KIDIW.....
123409976    ......EFLKI.....ICEIL.....FIVTK.....IVHSD.....IDFGC.....SIDVW.....
123399584    ......NFLKI.....ILELI.....FIVTK.....IIHSD.....IDFGC.....SIDVW.....
123411095    ......RFINN.....FPKIY.....VIVME.....CIHAD.....IDF-S.....SLDIW.....
123414780    ......KANSS.....VSDVH.....CIVLD.....IIHCD.....VDFGS.....KADVW.....
123424395    ......MIVQH.....IVRTF.....AVSFE.....VVHCD.....IDFGS.....PMDIW.....
123436158    ......EVNEA.....FAKYF.....VFVFE.....YIHCD.....IDLGC.....KIDVW.....
123446210    ......QILAR.....VVKAN.....CITFE.....IVHCD.....IDFGS.....PMDMW.....
123438931    ......QEADP.....IVPVI.....CFCFE.....LVHCD.....IDFGC.....AIDVW.....
123445226    ......YIATN.....IATYY.....CIVLE.....LIHSD.....IDFGA.....KIDIW.....
123439229    ......NLLTD.....IVQME.....VIIYE.....LIHAD.....VDLGG.....AADIW.....
123444985    ......RIQEK.....IITMI.....CIVSE.....IIHGD.....IDFGS.....KVDVW.....
123437716    ......SSLAK.....IVRAY.....CITYE.....IIHCD.....IDFGT.....EIDVW.....
123454032    ......QMQKA.....VGNPI.....CMVMD.....IIHCD.....IDFGS.....KIDIW.....
123448889    ......SILQH.....VVQNM.....CATFE.....VIHCD.....LDFGS.....PMDIW.....
123455333    ......NLMKD.....C-DFF.....CIVIE.....KLHGD.....IDMET.....EVDIW.....
123454689    ......QKLAT.....CIHFV.....CIVNE.....IVHSD.....VDFGS.....KIDIW.....
123473140    ......NQNAR.....YIPMH.....CIILD.....VIHTD.....IDFGN.....KIDIW.....
123460029    ......KIISR.....IIRYI.....CIVME.....IVHCD.....IDFGC.....EIDIW.....
123501212    ......LILPL.....TGRFL.....GFLME.....LVHGD.....IDWSS.....GVDIW.....
123488673    ......RFLET.....IIELI.....YIVTK.....IIHSD.....IDFGC.....SIDVW.....
123507614    ......MVLTE.....IGTMI.....VILFS.....YIHGD.....IDFGS.....KIDIW.....
123483682    ......DILKH.....ILHYI.....CIVME.....IIHCD.....IDFGC.....GIDVW.....
123494002    ......QILSR.....VVRAY.....CVTFE.....VVHCD.....IDFGS.....PMDVW.....
154420639    ......HQLDH.....IIMPI.....IIV-E.....VTHCD.....IDLGS.....KIDLW.....
154413949    ......HFLRR.....FVKFI.....CIVLE.....KIHTD.....VDFGS.....EADIW.....
154422402    ......AIIQR.....IVRTF.....AVSFE.....IVHCD.....IDFGS.....PMDIW.....
154416958    ......AYLTT.....IIRYK.....CIVME.....VIHSD.....IDFGC.....KIDIW.....
123857735    ......SILAR.....FVRAY.....CLVFE.....LIHAD.....IDFGS.....AIDMW.....
123857736    ......SILAR.....FVRAY.....CLVFE.....LIHAD.....IDFGS.....AIDMW.....
124297502    ......SILAR.....FVRAY.....CLVFE.....LIHAD.....IDFGS.....AIDMW.....
145477455    ......EILRI.....VIKML.....CMVFE.....IIHCD.....IDFGS.....QIDMW.....
145481497    ......EILKM.....LIKML.....CMVFE.....IIHCD.....IDFGS.....QIDMW.....
145484886    ......DILKV.....LVKML.....CIVFE.....IIHCD.....IDFGS.....AIDMW.....
145481045    ......QQFDP.....IVRFY.....CILLE.....ITHTD.....IDMGG.....SSDIW.....
145486519    ......NVLRT.....IIQLN.....CLVFP.....VVHAD.....IDFGS.....KIDIW.....
145490710    ......DLLKI.....LIKLM.....CMVFE.....IIHCD.....IDFGS.....QIDMW.....
145494047    ......NEADP.....ILRLI.....CLVFE.....IIHAD.....CDFGT.....NIDVW.....
145494063    ......RVLRT.....IIQLN.....CLVFP.....VVHAD.....IDFGS.....KIDIW.....
145497619    ......NILTY.....IVKIK.....CISFE.....IIHCD.....IDFGS.....NIDMW.....
145503368    ......KLIDH.....IVKVY.....CILME.....YLHTD.....IDFGN.....KQDIW.....
145500494    ......KLLKY.....VLKLY.....FLVTE.....LIHCD.....IDFGS.....KIDMW.....
145503392    ......QILTF.....IIKIK.....CLNFE.....IIHCD.....IDFGS.....SIDMW.....
145503846    ......QLLDP.....IVKFY.....CILLE.....ITHTD.....IDMGG.....SSDIW.....
145510478    ......QAKDP.....IVKLQ.....CLVFE.....IVHCD.....VDFGT.....EIDMW.....
145519994    ......REKDS.....IVRIE.....CIVLE.....IIHCD.....VDFGS.....EIDMW.....
145527430    ......NILTY.....IVKIK.....CISFE.....IIHCD.....IDFGS.....SIDMW.....
145526799    ......KLLRY.....VLRLY.....FLVTE.....LIHCD.....IDFGS.....KIDMW.....
145530315    ......KERDP.....IVRIE.....CIVFE.....IVHCD.....VDFGS.....EIDMW.....
145537698    ......GILRT.....IVRAY.....CIVFE.....YTHTD.....IDLGG.....LSDMW.....
145542418    ......EILKV.....LIKML.....CLVFE.....IIHCD.....IDFGS.....QIDMW.....
145539255    ......QLLDP.....IVKFY.....CILLE.....ITHTD.....ND--Q.....IIH-W.....
145540844    ......GILRT.....IVRAY.....CIVFE.....YTHTD.....IDLGG.....LSDMW.....
145541341    ......RERDP.....IVRIE.....CIVFE.....IVHCD.....VDFGS.....EIDMW.....
145545865    ......NILSF.....IVKIK.....CIICE.....IIHCD.....IDFGS.....GIDMW.....
145544757    ......NILQY.....IVKNI.....CIVFE.....IIHCD.....VDFGS.....EIDMW.....
145544997    ......KLIRF.....IIKLI.....CIVFP.....WVHCD.....IDFGS.....AIDMW.....
145551969    ......NILSF.....IVKIK.....CIICE.....IIHCD.....IDFGS.....GIDMW.....
145553122    ......EILQT.....IVKLL.....VLIFE.....ILLCD.....ADFGN.....SADIW.....
125528269    ......KLLKF.....VLRLY.....FIVTE.....IIHCD.....IDLGS.....RIDIW.....
125537568    ......SILEK.....CVRFI.....CLVFE.....VLHCD.....CDFGN.....PLDMW.....
125545535    ......KLLKY.....LLRLY.....LIVCE.....LIHCD.....IDLGS.....KIDMW.....
125552652    ......KLLKF.....ILRLY.....FIVTE.....IVHCD.....IDLGS.....KIDIW.....
125580226    ......SILEK.....CVRFI.....CLVFE.....VLHCD.....CDFGN.....PLDMW.....
125572529    ......KLLKF.....VLRLY.....FIVTE.....IIHCD.....IDLGS.....RIDIW.....
125583381    ......SLLSM.....IVRML.....CIAFE.....IIHCD.....IDFGS.....KVGRW.....
125594547    ......KLLKF.....ILRLY.....FIVTE.....IVHCD.....IDLGS.....KIDIW.....
```

125828790	......SILNR......FVRSY......CLVFE......LIHAD......IDFGS......AIDMW......
125837103	......RLLEL......IVHLK......CLVFE......IIHCD......VDFGS......AIDMW......
125840318	......RLLEL......IVHLK......CLVFE......IIHCD......VDFGS......AIDMW......
125854823	......SILAR......LVRAF......CLVFE......LIHAD......IDFGS......AIDMW......
125855579	......KILDA......VIHMK......CISFE......IIHCD......VDFGS......AIDMW......
126723770	......SILSR......FVRSY......CLVFE......LIHAD......IDFGS......AIDMW......
147904940	......EFLKK......CLRLF......CLVFE......ILHAD......CDFGS......GIDMW......
161333817	......KLLRC......VIRFL......YLVFE......IIHAD......IDFGS......KVDVW......
126654564	......SFVKK......IVQVQ......CIVFE......LVHAD......SDFGS......PIDIW......
126311629	......SILSR......FVRSY......CLVFE......LIHAD......IDFGS......AIDMW......
126306757	......KILEH......VIHML......CMAFE......IIHCD......IDFGS......PIDVW......
126322417	......EFLKK......CLRLF......CLVFE......ILHAD......CDFGS......GIDMW......
126325331	......RLLEL......IVHLK......CLVFE......IIHCD......VDFGS......AIDMW......
126329333	......KLLRC......IIRFL......YLVFE......IIHAD......IDFGS......KVDVW......
126329119	......RLLEL......IVHLK......CLVFE......IIHCD......VDFGS......AIDMW......
126329117	......RLLEL......IVHLK......CLVFE......IIHCD......VDFGS......AIDMW......
126332234	......SILSR......FVRAY......CLVFE......LIHAD......IDFGS......AIDMW......
126332232	......SILSR......FVRAY......CLVFE......LIHAD......IDFGS......AIDMW......
126340193	......KILEA......VVHMK......CITFE......IIHCD......IDFGS......AIDMW......
126340869	......SILAR......FVRAY......CLVFE......LIHAD......IDFGS......AIDMW......
145235457	......NLLQK......VVNFT......CISTE......VIHCD......IDFGS......PIDMW......
145257079	......SDVEH......VVELL......CLVFP......IVHCD......GDLGG......AIDIW......
154334438	......DILLR......VVRLL......VLVFE......IIHCD......IDFGS......PIDRW......
154334237	......HLNND......VVQLR......VLVFE......IIHCD......IDFGS......AIDRW......
154337324	......RALTC......VVRLL......VLVTE......LIHCD......LDLGS......AVDIW......
154344066	......GILQR......IVRMI......CISYE......IIHCD......ADLGS......AIDWW......
154335200	......GICEQ......CLSMA......IIVFP......ILHGD......IDLGH......AIDMW......
154340160	......HEHDG......VSKMY......CPVFI......MVHTD......CDFGS......AADMW......
154344691	......NTLEL......IVRLL......ILVFE......IVHCD......IDFGS......AIDMW......
146081498	......HLNND......VVQLR......VLVFE......IIHCD......IDFGS......AIDRW......
146091011	......HEHDK......VSKMY......CPVFI......MVHTD......CDFGS......AADMW......
146081806	......DILLR......VVRLL......VLVFE......IIHCD......IDFGS......PIDRW......
146098030	......HMLQH......IVRMT......CVSYE......IIHCD......IDLGS......AIDWW......
146101011	......KTLEL......IVRLL......ILVFE......IVHCD......IDFGS......AIDMW......
146086129	......RALTC......VVRLL......VLVTE......LIHCD......LDLGS......AVDIW......
154346264	......SALLC......SANIL......CIVSE......YMHCD......IDFGA......AIDSW......
146105275	......SALLC......SVNIL......CIVSE......YIHCD......IDFGA......AIDSW......
145238160	......NLLAD......IVSVL......CIVFE......LSHAD......LDFGE......RVDLW......
145242184	......NMYRL......PIHLL......CLVHP......IAHTD......CDFGS......SADIW......
145248790	......SVLDL......LLRLK......CLVFE......LIHCD......IDFGS......AIDMW......
145251161	......SIADH......IRKAL......CLVHP......IVHTD......CDFGQ......KVDIW......
145252488	......GILEQ......VIKFE......CMVFE......ILHAD......CDLGS......AIDVW......
134035221	......SILSR......FVRSY......CLVFE......LIHAD......IDFGS......AIDMW......
158513576	......SILSR......FVRSY......CLVFE......LIHAD......IDFGS......AIDMW......
145341102	......DILQK......CVRFV......FMVFE......VVHAD......CDFGS......PMDLW......
145347077	......SVLKR......VVKMF......CVVFE......VVHCD......IDFGS......PIDVW......
145345962	......KLLRL......LLRMF......FIVSE......LIHCD......IDFGS......KIDVW......
145351353	......GILRT......IVDLM......CLVFE......VVHCD......VDFGS......PIDMW......
145602241	......AILQK......IVKFE......CMAFE......IIHAD......CDLGT......AADMW......
145610979	......NILQK......MVNFT......CISTE......VIHCD......IDFGS......PIDMW......
145332685	......QILKK......CVRFL......CLVFE......VLHCD......CDFGN......PLDIW......
146186202	......NILSY......IVRIK......-----......IIHCD......IDFGS......SIDMW......
146179761	......KLLRY......VLRYF......FIITE......LIHCD......IDFGS......RIDIW......
146165548	......KLLKY......VLKVY......FIVTE......LIHCD......IDFGS......KIDMW......
146421063	......KILKN......ILQYF......CIVSE......IIHCD......IDFGS......LIDLW......
146419655	......RLLEF......FIRLL......CLVFE......IIHCD......IDFGS......SIDMW......
149247510	......KMLKL......VINFY......CIATE......IIHCD......IDFGS......KIDIW......
149245178	......RLLEF......FIRLL......CLVFE......MIHCD......IDFGS......SIDMW......
147784275	......VILKK......CVRFL......CLVFE......VLHCD......CDFGN......PMDIW......
148667430	......KILEA......VVHMK......CITFE......IIHCD......IDFGS......AIDMW......
148692205	......RLLEL......IVHLK......CLVFE......IIHCD......VDFGS......AIDMW......
148692234	......KLLRC......VIRFL......YLVFE......IIHAD......IDFGS......KVDVW......
148692437	......RILEH......VIHML......CMTFE......IIHCD......IDFGS......PIDMW......
148708948	......EFLKK......CLRLF......CLVFE......ILHAD......CDFGS......GIDMW......
153792057	......SILAR......FVRAY......CLVFE......LIHAD......IDFGS......AIDMW......
156098468	......SILKK......IVRLL......CLVFE......IMHAD......CDLGS......QIDVW......
157821775	......RILEH......VIHML......CMTFE......IIHCD......IDFGS......PIDMW......
149022796	......SILAR......FVRAY......CLVFE......LIHAD......IDFGS......AIDMW......
149017701	......RLLEL......IVHLK......CLVFE......IIHCD......VDFGS......AIDMW......
149045217	......EFLKK......CLRLF......CLVFE......ILHAD......CDFGS......GIDMW......
149056482	......RLLEL......IVHLK......CLVFE......IIHCD......VDFGS......AIDMW......
157819763	......RLLEL......IVHLK......CLVFE......IIHCD......VDFGS......AIDMW......
149049370	......KILEA......VVHMK......CITFE......IIHCD......IDFGS......AIDMW......
149058677	......RILEH......VIHML......CMAFE......IIHCD......IDFGS......PIDIW......

```
149065293    ......SILAR.....FVRAY.....CLVFE.....LIHAD.....IDFGS.....AIDMW.....
157820597    ......SILAR.....FVRAY.....CLVFE.....LIHAD.....IDFGS.....AIDMW.....
149065294    ......SILAR.....FVRAY.....CLVFE.....LIHAD.....IDFGS.....AIDMW.....
150865631    ......RLLEY.....FIRLL.....CLVFE.....MIHCD.....IDFGS.....SIDMW.....
150864334    ......KMLRL.....ILKYY.....CIVTE.....VIHCD.....IDFGS.....GIDVW.....
149409697    ......SILAR.....FVRAY.....CLVFE.....LIHAD.....IDFGS.....AIDMW.....
149409699    ......SILAR.....FVRAY.....CLVFE.....LIHAD.....IDFGS.....AIDMW.....
149481746    ......SILAR.....FVRAY.....CLVFE.....LIHAD.....IDFGS.....AIDMW.....
149523637    ......KILDA.....MVHMK.....CITFE.....IIHCD.....IDFGS.....AIDMW.....
149632269    ......RILEH.....VIHML.....CMTFE.....IIHCD.....IDFGS.....PIDMW.....
149633793    ......RLLEL.....IVHLK.....CLVFE.....IIHCD.....VDFGS.....AIDMW.....
149636482    ......EFLKK.....CLRLF.....CLVFE.....ILHAD.....CDFGS.....GIDMW.....
149708003    ......RILEH.....VIHML.....CMAFE.....IIHCD.....IDFGS.....PIDIW.....
149708775    ......SILSR.....FVRSY.....CLVFE.....LIHAD.....IDFGS.....AIDMW.....
149722289    ......KLLRC.....VIRFL.....YLVFE.....IIHAD.....IDFGS.....KVDVW.....
149731920    ......EFLKK.....CLRLF.....CLVFE.....ILHAD.....CDFGS.....GIDMW.....
149742131    ......RLLEL.....IVHLK.....CLVFE.....IIHCD.....VDFGS.....AIDMW.....
149742133    ......RLLEL.....IVHLK.....CLVFE.....IIHCD.....VDFGS.....AIDMW.....
149742135    ......RLLEL.....IVHLK.....CLVFE.....IIHCD.....VDFGS.....AIDMW.....
149747388    ......SILAR.....FVRAY.....CLVFE.....LIHAD.....IDFGS.....AIDMW.....
149747367    ......SILAR.....FVRAY.....CLVFE.....LIHAD.....IDFGS.....AIDMW.....
149758288    ......SILAR.....FVRAY.....CLVFE.....LIHAD.....IDFGS.....AIDMW.....
149758290    ......SILAR.....FVRAY.....CLVFE.....LIHAD.....IDFGS.....AIDMW.....
154284560    ......ELLEQ.....IIRFE.....CIVFE.....ILHAD.....CDLGS.....AIDMW.....
154271057    ......SVLDL.....LLRLR.....CLVFE.....LIHCD.....IDFGS.....AIDMW.....
154312350    ......SVLDL.....LLRLK.....CLVFE.....LIHCD.....IDFGS.....AIDMW.....
154314947    ......EILEK.....MIRLE.....CMVFE.....ILHAD.....CDLGS.....AIDIW.....
154304859    ......NILQK.....MVNFV.....CISTE.....VIHCD.....IDFGS.....PIDMW.....
157107065    ......RLLKN.....LVCLL.....HLVFE.....CLHRD.....CDFGF.....PVDVW.....
109078618    ......KFLKQ.....LVNLI.....HLVFE.....IIHRD.....CDFGF.....PVDIW.....
109078620    ......KFLKQ.....LVNLI.....HLVFE.....IIHRD.....CDFGF.....PVDIW.....
109083526    ......RMLKQ.....LVNLL.....HLVFE.....CIHRD.....CDFGF.....PVDVW.....
109102764    ......RMLKQ.....LVNLI.....HLVFE.....CIHRD.....CDFGF.....SVDIW.....
109478928    ......RMLKQ.....LVNLI.....HLVFE.....CIHRD.....CDFGF.....SVDIW.....
110756421    ......RLLKN.....LVNLL.....HLVFE.....CVHRD.....CDFGF.....PVDVW.....
114594220    ......KLLKQ.....LVNLL.....YLVFE.....IIERD.....-DFGS.....AVDVW.....
114594216    ......KLLKQ.....LVNLL.....YLVFE.....IIERD.....-DFGS.....AVDVW.....
114594218    ......KLLKQ.....LVNLL.....YLVFE.....IIERD.....-DFGS.....AVDVW.....
114601699    ......KFLKQ.....LVNLI.....HLVFE.....IIHRD.....CDFGF.....PVDIW.....
115696674    ......KLLRQ.....IVELK.....YLVFE.....VIHRD.....YNKGF.....AVDVW.....
115707274    ......RMLKQ.....LVNLI.....YLVFE.....IIHRD.....CDFGF.....AIDLW.....
118084096    ......KMLRT.....IVELK.....YLVFE.....IVHRD.....CDFGF.....AVDMW.....
118089977    ......KLLKQ.....LVSLL.....YLVFE.....IIHRD.....CDFGF.....AVDVW.....
118092339    ......RMLKQ.....LVNLL.....HLVFE.....CIHRD.....CDFGF.....PVDVW.....
119586118    ......RMLKQ.....LVNLL.....HLVFE.....CIHRD.....CDFGF.....PVDVW.....
166064025    ......KFLKQ.....LVNLI.....HLVFE.....IIHRD.....CDFGF.....PVDIW.....
119895479    ......KFLKQ.....LVNLI.....HLVFE.....IIHRD.....CDFGF.....PVDIW.....
119620754    ......RMLKQ.....LVNLI.....HLVFE.....CIHRD.....CDFGF.....SVDIW.....
119619346    ......KMLRT.....IVELK.....YLVFE.....IVHRD.....CDFGF.....SVDMW.....
155371961    ......RMLKQ.....LVNLI.....HLVFE.....CIHRD.....CDFGF.....PVDVW.....
123297771    ......KFLKQ.....LVNLI.....HLVFE.....IIHRD.....CDFGF.....PVDIW.....
145500664    ......KVLRL.....IVELK.....YLVFE.....IVHRD.....CDFGF.....GVDMW.....
145502353    ......KLLKM.....IVKIK.....YVVLE.....ILHRD.....CDFGF.....GVDIW.....
145510344    ......KMLRM.....IIQLK.....FLVFQ.....IAHRD.....CDFGF.....QVDIW.....
145517482    ......KMLRL.....IIDLF.....YLVFE.....IVHRD.....IDFGF.....GVDLW.....
145518662    ......KMLRL.....IIDLF.....YLVFE.....IVHRD.....IDFGF.....GVDLW.....
145526629    ......KVLRL.....IVELK.....YLVFE.....IVHRD.....CDFGF.....GVDMW.....
145529215    ......KVLRL.....IVELK.....YLVFE.....IVHRD.....CDFGF.....GVDMW.....
145534594    ......KVLRL.....IVELK.....YLVFE.....IVHRD.....CDFGF.....GVDMW.....
145537223    ......KMLRL.....IIQLK.....FLVFQ.....IVHRD.....CDFGF.....QVDIW.....
145538642    ......KLLKM.....IVKIK.....YVVLE.....ILHRD.....CDFGF.....GVDIW.....
145549672    ......KMLRM.....IIQLK.....FLVFQ.....IVHRD.....CDFGF.....QVDIW.....
125829693    ......KMLRT.....IVELK.....YLVFE.....IVHRD.....CDFGF.....AVDMW.....
126654525    ......ATLRR.....IIQLK.....YLSLE.....IIHRD.....CDFGF.....GIDVW.....
126290198    ......KFLKQ.....LVNLI.....HLVFE.....IIHRD.....CDFGF.....PVDIW.....
126304528    ......RLLKQ.....LVNLI.....HLVFE.....CIHRD.....CDFGF.....TVDVW.....
126330854    ......KLLKQ.....LVNLL.....YLVFE.....IIHRD.....CDFGF.....AVDIW.....
126336980    ......KMLRT.....IVELK.....YLVFE.....IVHRD.....CDFGF.....SVDMW.....
154343519    ......RMLQL.....VIRLE.....YLVFE.....VIHRD.....CDFGF.....PVDVW.....
154340271    ......RVEQL.....VTQLL.....YLVME.....IIHRD.....CDFGF.....AVDMW.....
146097043    ......RMLQL.....VIRLE.....YLVFE.....VIHRD.....CDFGF.....PVDVW.....
157074066    ......KLLKQ.....LVNLL.....YLVFE.....IIHRD.....CDFGF.....AVDVW.....
```

148701688 ......KFLKQ.....LVNLI.....HLVFE.....IIHRD.....CDFGF.....PVDIW.....
148701685 ......KFLKQ.....LVNLI.....HLVFE.....IIHRD.....CDFGF.....PVDIW.....
148701684 ......KFLKQ.....LVNLI.....HLVFE.....IIHRD.....CDFGF.....PVDIW.....
148706580 ......RMLKQ.....LVNLI.....HLVFE.....CIHRD.....CDFGF.....SVDVW.....
148701687 ......KFLKQ.....LVNLI.....HLVFE.....IIHRD.....CDFGF.....PVDIW.....
149033803 ......KLLKQ.....LVNLL.....YLVFE.....IIHRD.....CDFGF.....AVDIW.....
149033804 ......KLLKQ.....LVNLL.....YLVFE.....IIHRD.....CDFGF.....AVDIW.....
149052527 ......KFLKQ.....LVNLI.....HLVFE.....IIHRD.....CDFGF.....PVDIW.....
149052529 ......KFLKQ.....LVNLI.....HLVFE.....IIHRD.....CDFGF.....PVDIW.....
149052526 ......KFLKQ.....LVNLI.....HLVFE.....IIHRD.....CDFGF.....PVDIW.....
149410357 ......RMLKQ.....LVNLL.....HLVFE.....CIHRD.....CDFGF.....PVDVW.....
149636700 ......KFLKQ.....LVNLI.....HLVFE.....IIHRD.....CDFGF.....PVDIW.....
149638292 ......KMLRT.....IVELK.....YLVFE.....IVHRD.....CDFGF.....SVDMW.....
149692970 ......RMLKQ.....LVNLI.....HLVFE.....CIHRD.....CDFGF.....PVDVW.....
149726878 ......KFLKQ.....LVNLI.....HLVFE.....IIHRD.....CDFGF.....PVDIW.....
149727622 ......RMLKQ.....LVNLI.....HLVFE.....CIHRD.....CDFGF.....SVDVW.....
149727624 ......RMLKQ.....LVNLI.....HLVFE.....CIHRD.....CDFGF.....SVDVW.....
149744368 ......KMLRT.....IVELK.....YLVFE.....IVHRD.....CDFGF.....SVDMW.....
109128955 ......RVLRR.....IIQLV.....YMVME.....ITHRD.....TDFGL.....SVDMW.....
115658004 ......TILRR.....IIQLL.....YMIME.....ITHRD.....TDFGL.....SVDLW.....
118096146 ......SVLRR.....IIQLI.....YMVME.....ITHRD.....TDFGL.....SVDMW.....
125831923 ......RVLRR.....IIRLT.....YLVLE.....VIHRD.....TDFGL.....AVDMW.....
126304695 ......CVLRR.....IIQLI.....YMVME.....ITHRD.....TDFGL.....SVDMW.....
126322437 ......SVLRR.....IVQLI.....YIVME.....ITHRD.....TDFGL.....AVDMW.....
148726021 ......CVLRR.....IIQLM.....YMVME.....ITHRD.....TDFGL.....AVDMW.....
149699246 ......RVLRR.....IIQLV.....YMVME.....ITHRD.....TDFGL.....SVDMW.....
149721405 ......TVLKR.....IVQLM.....YIVME.....ITHRD.....TDFGL.....AVDMW.....
108712089 ......KALQK.....IVKLK.....FFIFE.....YFHRD.....ADFGL.....AIDMW.....
157114393 ......QVMKV.....VVSFV.....TLIME.....IFHRD.....ADFGS.....KMDIW.....
157107307 ......KSLKK.....VVKLK.....YFVFE.....FFHRD.....ADFGL.....AIDLW.....
109069610 ......KSLKK.....VIKLK.....YFIFE.....FFHRD.....ADFGL.....PIDVW.....
109084951 ......QALRR.....ILTLH.....ALICE.....IFHRD.....GDFGS.....KMDLW.....
110809633 ......QAVRR.....IVDLV.....ALVLE.....VFHRD.....ADFGS.....KMDLW.....
110809635 ......DVVRR.....VVKLR.....FFVFE.....YFHRD.....ADFGL.....AVDVW.....
118404318 ......KSLKK.....VVKLK.....FFVFE.....FFHRD.....ADFGL.....PIDIW.....
118404664 ......QALRR.....ILTLC.....SLICE.....IFHRD.....GDFGS.....KMDIW.....
115384204 ......IFLRT.....LVPAL.....HICME.....FFHRD.....ADFGL.....PVDMW.....
114605463 ......KSLKK.....VIKLK.....YFIFE.....FFHRD.....ADFGL.....PIDVW.....
114605457 ......KSLKK.....VIKLK.....YFIFE.....FFHRD.....ADFGL.....PIDVW.....
114607895 ......KSLKK.....VVKLK.....YFIFE.....FFHRD.....ADFGL.....PIEVW.....
114654813 ......-----.....-----.....ALICE.....IFHRD.....GDFGS.....KMDLW.....
114654815 ......-----.....-----.....ALICE.....IFHRD.....GDFGS.....KMDLW.....
114654811 ......QALRR.....ILMLH.....ALICE.....IFHRD.....GDFGS.....KMDLW.....
114654827 ......QALRR.....ILMLH.....ALICE.....IFHRD.....GDFGS.....KMDLW.....
115688419 ......KSLKK.....IVKLK.....YFVFE.....FFHRD.....ADFGL.....PIDMW.....
72004410f ......QAMKR.....ILELK.....SLVCE.....IFHRD.....ADFGS.....QMDMW.....
169857254 ......QSLAT.....----Y.....FLVFE.....IAHTD.....EDFQN.....PVDIF.....
169843972 ......ESLRA.....IIPLY.....YFVFE.....YFHRD.....ADFGL.....PVDLW.....
118086352 ......KSLKK.....VIKLK.....YFVFE.....FFHRD.....ADFGL.....PIDIW.....
118086350 ......KSLKK.....VIKLK.....YFVFE.....FFHRD.....ADFGL.....PIDIW.....
118088861 ......KSLKK.....VVKLK.....YFVFE.....FFHRD.....ADFGL.....PIDIW.....
118092188 ......QALRR.....ILMLH.....SLICE.....IFHRD.....ADFGS.....KMDIW.....
121715960 ......IFLRT.....LVPAL.....HICME.....FFHRD.....ADFGL.....PVDMW.....
119481489 ......IFLRT.....LVPAL.....HICME.....FFHRD.....ADFGL.....PVDMW.....
119575686 ......KSLKK.....VIKLK.....YFIFE.....FFHRD.....ADFGL.....PIDVW.....
119602180 ......QALRR.....ILMLH.....ALICE.....IFHRD.....GDFGS.....KMDLW.....
119602181 ......-----.....-----.....ALICE.....IFHRD.....GDFGS.....KMDLW.....
149643087 ......KSLKK.....VVKLK.....YFIFE.....FFHRD.....ADFGL.....PIDIW.....
123354649 ......KSLRK.....VVRLL.....YMVFE.....FFHRD.....ADFGL.....PVDIW.....
123367011 ......KSLRK.....VEKLL.....YLVFE.....FFHRD.....ADFGL.....PVDIW.....
123438110 ......LYLRA.....IIKLY.....ALRFE.....LFHRD.....CDFGS.....EVDIW.....
123455201 ......MCLRA.....IIRLE.....ALVFE.....LFHRD.....ADFGS.....AVDIW.....
123471874 ......QYLKA.....IIKLY.....YLLFE.....LFHRD.....CDFGS.....EVDIW.....
123484086 ......TSLRK.....VVRLL.....FLVFE.....FFHRD.....CDFGL.....PVDIW.....
154415594 ......KSLRK.....VVKLV.....YLAFE.....FFHRD.....IDFGL.....PVDIW.....
145477787 ......KALMK.....IVKLK.....CLVFE.....YFHRD.....IDFGL.....PVDIF.....
145476427 ......QALRK.....IIKLI.....ALVFE.....IFHRD.....ADLGS.....KMDIW.....
145482401 ......QALRK.....IIKLI.....ALVFE.....IFHRD.....ADFGS.....KMDLW.....
145490301 ......KALMK.....IVKLK.....CLVFE.....YFHRD.....IDLGC.....PVDIF.....
145493037 ......KALLK.....IVKLK.....CLVFE.....YFHRD.....IDLGC.....PVDIF.....
145495657 ......KALLK.....IVKLK.....CLVFE.....YFHRD.....IDLGC.....PVDIF.....

```
145498467    ......QILKK......IIKLR......AIVLD......YLHRD......IDFGQ......EIDIW......
145498078    ......QILKK......IIKLR......AIVLD......YLHRD......IDFGQ......EVDIW......
145502277    ......KALMK......IIKLL......YLIYE......YFHRD......IDFGL......QIDIF......
145512239    ......KSLLK......IVKLY......HFVFE......YFHRD......ADFGL......PIDIF......
145516466    ......KALQK......IIKLV......NLVFE......YFHRD......CDFGL......PVDIF......
145518163    ......QALRK......IIKLI......ALVFE......IFHRD......ADLGS......KMDIW......
145521801    ......KSLLK......IVKLY......HFVFE......YFHRD......ADFGL......PIDIF......
145524761    ......KALQK......IIKLV......NLVFE......YFHRD......CDFGL......PVDIF......
145525140    ......QALRK......IIKLI......ALVFE......IFHRD......ADLGS......KMDIW......
145529077    ......QALHQ......IVKLN......ALVCE......FFHRD......ADLGS......KMDLW......
145534712    ......QALHQ......IVKLH......ALVCE......FFHRD......ADLGS......KMDIW......
145532727    ......QALRK......IIKLI......ALVFE......IFHRD......ADFGS......KMDLW......
145538556    ......KALMK......IIKLL......YLIYE......YFHRD......IDFGL......SIDIF......
145547096    ......QALRK......IIKLI......ALVFE......IFHRD......ADFGS......KMDLW......
145546863    ......QALRK......IIRLI......ALVFE......IFHRD......ADFGS......KMDLW......
145545616    ......KALMK......IVKLK......CLVFE......YFHRD......IDLGC......PVDIF......
125540794    ......KSLRR......IVKLK......YFIME......YFHRD......ADFGL......AVDMW......
125553810    ......KSLRR......IVKLK......FFVFE......YFHRD......ADFGL......AVDMW......
125583369    ......KSLRR......IVKLK......YFIME......YFHRD......ADFGL......AVDMW......
125595836    ......KSLRR......IVKLK......FFVFE......YFHRD......ADFGL......AVDMW......
126133527    ......RIMAI......LVQIY......HISME......YFHRD......ADYGL......PIDIW......
126290468    ......QALRR......ILTLH......ALICE......IFHRD......GDFGS......KMDMW......
126310138    ......KSLKK......VVKLK......YFVFE......FFHRD......ADFGL......PIDIW......
126321998    ......KSLKK......VIKLK......YFVFE......FFHRD......ADFGL......PIDVW......
126321996    ......KSLKK......VIKLK......YFVFE......FFHRD......ADFGL......PIDVW......
145231725    ......IFLRS......LVPAL......HICME......FFHRD......ADFGL......PVDMW......
154339632    ......DVVRR......VVKLR......FFVFE......YFHRD......ADFGL......AVDVW......
154335818    ......QSLRK......LVKLK......FLIFE......FMHRD......ADFGL......PIDIW......
154345303    ......QAVRR......IVDLI......ALVLE......VFHRD......ADFGS......KMDLW......
146092811    ......QSLRK......LVKLK......FMIFE......FMHRD......ADFGL......PVDIW......
146102271    ......QAVRR......IVDLV......ALVLE......VFHRD......ADFGS......KMDLW......
146090641    ......DVVRR......VVKLR......FFVFE......YFHRD......ADFGL......AVDVW......
134276939    ......KSLKK......IIKLR......YFVFE......FFHRD......ADFGL......PIDMW......
145613231    ......VFLRT......LVPAL......HIAME......FFHRD......ADFGL......PVDIW......
146163216    ......KALRL......IIKIK......ICVYE......FFHRD......SSFTV......KVDIW......
146162383    ......KSLRK......LVKLK......MLVFE......YFHRD......CDFGL......PIDVF......
146421445    ......QFIQA......LLHIY......HFSME......YFHRD......ADYGL......PVDIW......
149246626    ......KILSV......LVQVF......HISME......YFHRD......GDYGL......PIDIW......
148686709    ......QALRR......ILALH......ALICE......IFHRD......GDFGS......KMDLW......
148709020    ......KSLKK......VIKLK......YFVFE......FFHRD......ADFGL......PIDVW......
148709021    ......KSLKK......VIKLK......YFVFE......FFHRD......ADFGL......PIDVW......
148709022    ......KSLKK......VIKLK......YFVFE......---RD......ADFGL......PIDVW......
149019107    ......KSLKK......IVKLK......YFIFE......FFHRD......ADFGL......PIDVW......
149045136    ......KSLKK......VIKLK......YFIFE......FFHRD......ADFGL......PIDVW......
149044116    ......QALRR......ILTLH......ALICE......IFHRD......GDFGS......KMDLW......
149044114    ......QALRR......ILTLH......ALICE......IFHRD......GDFGS......KMDLW......
149638578    ......KSLKK......VIKLK......YFVFE......FFHRD......ADFGL......PIDVW......
149638580    ......KSLKK......VIKLK......YFVFE......FFHRD......ADFGL......PIDVW......
149731794    ......KSLKK......VIKLK......YFIFE......FFHRD......ADFGL......PIDVW......
149737721    ......QALRR......ILMLH......ALICE......IFHRD......GDFGS......KMDLW......
154274972    ......IFLRT......LVPAL......HICME......FFHRD......ADFGL......PVDMW......
108707300    ......ALLSQ......IVQYY......YIFLE......IVHRD......ADFGL......EADIW......
108707610    ......RILRR......VVEYL......NLHME......VVHGD......ADFGA......ASDVW......
115455533    ......KFLSQ......IVQYY......YIYLE......IMHRD......ADFGM......AVDIW......
108864120    ......VLLSQ......IVQYY......SVYLE......TVHRD......ADFGM......SVDIW......
108864121    ......VLLSQ......IVQYY......SVYLE......TVHRD......ADFGM......SVDIW......
157126214    ......KIFEG......LVKYY......LIFME......VVHRD......GDFGS......AADIW......
157118478    ......KLHSQ......IVQYW......KIFME......IVHRD......SDFGT......AADIW......
109000008    ......ALHKR......IVRYL......KIFME......IVHRD......SDFGT......AADIW......
109000005    ......ALHKR......IVRYL......KIFME......IVHRD......SDFGT......AADIW......
109000011    ......ALHKR......IVRYL......KIFME......IVHRD......SDFGT......AADIW......
109073213    ......KIFEG......LVRYF......YIFME......IVHRD......GDFGC......AADIW......
109104423    ......DLLKA......IVAYL......SIFME......VVHRD......IDFGC......KSDIW......
109116803    ......QLLKN......IVQYY......TIFME......IVHRD......GDFGA......KADVW......
109157363    ......ALHKH......IVQYL......KIFME......IVHRD......SDFGT......AADIW......
109460509    ......ALHKH......IVQYL......KIFME......IVHRD......SDFGT......AADIW......
157816917    ......ALHKR......IVRYL......KIFME......IVHRD......SDFGT......AADIW......
109506412    ......QLLKN......IVQYY......SIFME......IVHRD......GDFGA......KADIW......
109510706    ......ALHKY......IVQYL......KIFME......IVHRD......SDFGT......PADIW......
109511783    ......ALHKY......IVQYL......KIFME......IVHRD......SDFGT......PADIW......
109730385    ......ALHKH......IVQYL......KIFME......IVHRD......SDFGT......AADIW......
```

171846249	......ALHKH......IVQYL......KIFME......IVHRD......SDFGT......AADIW......
110737157	......SILST......MVKYI......NILME......IVHCD......ADMGC......PADVW......
110738597	......SVFRS......IVKFL......NLYLE......FVHCD......ADFGS......ESDVW......
110758195	......QIFEG......LIRYY......LIFME......IVHRD......GDFGS......AADIW......
169604320	......DLLKN......IVKYN......YILLE......VIHRD......ADFGV......SSDIW......
111220232	......TAARH......VARVI......WLATE......VVHRD......IDFGV......AADIF......
153792015	......ALHKR......IVRYL......KIFME......IVHRD......SDFGT......AADIW......
111380701	......ELLQG......IVQYL......NIFLE......IIHRD......SDFGI......KADIW......
115439397	......RILSG......VLPCL......QLFLE......LVHGD......ADFGC......AADVW......
115439401	......RVMSG......IVPCL......QLFLE......LVHGD......ADFGC......AADVW......
115439399	......RVLSG......IVPCL......QLFLE......LVHGD......TDFGC......AADVW......
115439395	......GMMSG......VVPCI......NLFLE......LVHGD......ADFGC......AADVW......
115446691	......DMLKQ......IVQYY......SIYLE......TVHRD......ADFGM......AVDIW......
115445823	......GMLSA......VVPCV......DLFLE......IAHCD......ADFGC......AADIW......
115452407	......RILRR......VVEYL......NLHME......VVHGD......ADFGA......ASDVW......
115458518	......DMLRQ......IVQYY......SIYLE......TVHRD......ADFGM......PVDIW......
115465225	......GVMAG......VVPCI......QMFLE......LVHGD......ADFGC......AADVW......
115465223	......GILAS......VLPCF......GLLLE......MVHGD......ADFGC......AADVW......
115470339	......KFLSQ......IVQYY......YIYLE......IMHRD......ADFGM......AVDIW......
115478945	......KLLKN......IVRYL......NILLE......IIHRD......ADFGA......SADIW......
115484641	......VLLSQ......IVQYY......SVYLE......TVHRD......ADFGM......SVDIW......
115438224	......ELLQG......IVQYL......NIFLE......IIHRD......SDFGI......KADIW......
115398283	......DLLKN......IVKYQ......NIILE......VIHRD......ADFGV......ASDIW......
115491499	......GVLEV......IVSYH......YIFME......IIHRD......VDFGA......SVDIW......
115396346	......DTMQH......IVQYL......SIYLE......ILHRD......SDFGI......KVDIW......
114555005	......ALHRR......IVRYL......KIFME......IVHRD......SDFGT......AADIW......
114580714	......QLLKN......IVQYY......SIFME......IVHRD......GDFGA......KADIW......
114580718	......QLLKN......IVQYY......SIFME......IVHRD......GDFGA......KADIW......
114581130	......DLLKA......IVAYL......SIFME......VVHRD......IDFGC......KSDIW......
114609463	......ALHKH......IVQYL......KIFME......IVHRD......SDFGT......AADIW......
114609461	......ALHKH......IVQYL......KIFME......IVHRD......SDFGT......AADIW......
114609465	......ALHKH......IVQYL......KIFME......IVHRD......SDFGT......AADIW......
115644439	......KLMAT......IVRLM......NVFVE......VIHRD......ADLGT......SCDIW......
115668619	......QLLKT......IVGFL......NIFMQ......VIHRD......IDFGC......KSDIW......
115697792	......LLHSR......IVKYL......KIYME......IVHRD......SDFGT......PADIW......
115943016	......ELLRN......IVQYF......SIFME......IVHRD......ADFGA......RADVW......
116055854	......ELLSN......VVKYE......YVELE......VVHRD......ADFGA......AADVW......
116061057	......DVLSR......IVRYI......YIFLE......VLHRD......ADFGM......EADIW......
116248533	......ALHKY......IVQYL......KIFME......IVHRD......SDFGT......PADIW......
116311127	......SLLSR......IVQYY......YIYLE......TVHRD......ADFGM......AVDIW......
116310785	......DMLRQ......IVQYY......SIYLE......TVHRD......ADFGM......PVDIW......
116310036	......DLLKN......IVKYL......HIILE......VIHRD......ADFGV......ASDIW......
169858742	......DLLKN......IVKYK......YIILE......VIHRD......ADFGV......ASDIW......
169844593	......SVMEM......VVEYY......YIFEE......IVHRD......VDFGA......ATDIW......
169843834	......ETLKD......IVQYL......SIFLE......ILHRD......SDFGI......KIDIW......
169843856	......DTLKD......IVSYL......SIFLE......ILHRD......SDFGI......KVDIW......
169853015	......ELLKV......IVQYL......NIFLE......IIHRD......SDFGI......KADIW......
169849203	......DLVKS......IVKYE......SIVLE......VVHCD......SDFGV......KSDIW......
116643232	......SVLSR......IVQYY......YIYLE......TVHRD......ADFGM......AVDIW......
116643226	......ALLSQ......IVRYR......YIFLE......FVHRD......ADFGL......PADIW......
116643230	......NLLNQ......IVQYY......SVYLE......TVHRD......ADFGM......AVDIW......
116643224	......ALLSQ......ILRYR......YIFLE......FIHRD......ADFGL......PADIW......
116643234	......KLLSN......IVQYF......FIYLE......TVHRD......ADFGM......AVDIW......
116643222	......KLLSQ......IVRYR......YIFLE......FIHRD......ADFGL......PADIW......
116643244	......DLLKN......IVKYL......HIILE......VIHRD......ADFGV......ASDIW......
116643242	......DLLKN......IVKYL......HIILE......VIHRD......ADFGV......ASDIW......
116643238	......KLLKN......IVRYL......NILLE......IMHRD......ADFGA......SADIW......
116643236	......KLLKN......IVRYL......NILLE......IMHRD......ADFGA......SADIW......
116643240	......QLLKN......IVRYL......NILME......IMHRD......ADFGA......SADIW......
118088516	......ALHKH......IVQYL......KIFME......IVHRD......SDFGT......AADIW......
118088304	......KIFEG......LVRYF......YIFME......IVHRD......GDFGC......AADIW......
118093793	......QLLKN......IVQYY......SIFME......IVHRD......GDFGA......KADIW......
118093896	......DLLKT......IVTYL......SIFME......VVHRD......IDFGC......KSDIW......
118102844	......QLLKN......IVQYY......TIFME......IVHRD......GDFGA......KADVW......
118103813	......RMMSH......IIRML......NLFIE......IIHRD......ADFGA......SCDVW......
42540755	......KLLKN......IVRYL......NILLE......IMHRD......ADFGA......SADIW......
121701805	......GVLEV......IVSYH......YIFME......IVHRD......VDFGA......AVDIW......
121706594	......DLLKN......IVKYH......NIILE......VIHRD......ADFGV......ASDIW......
121705696	......DTMQH......IVQYL......SIYLE......ILHRD......SDFGI......KVDIW......
121712734	......ELLQG......IVQYL......NIFLE......IIHRD......SDFGI......KADIW......
119495794	......GVLEV......IVSYH......YIFME......IVHRD......VDFGA......AVDIW......

```
119501188   ......DLLKN......IVKYH......NIILE......VIHRD......ADFGV......ASDIW......
119498477   ......ELLQG......IVQYL......NIFLE......IIHRD......SDFGI......KADIW......
119491713   ......DTMQH......IVQYL......SIYLE......ILHRD......SDFGI......KVDIW......
119575348   ......RMMSH......IIRML......NLFIE......IIHRD......ADFGA......SCDVW......
119567972   ......KIFEG......LVRYF......YIFME......IVHRD......GDFGC......AADIW......
119575349   ......RMMSH......IIRML......NLFIE......IIHRD......ADFGA......SCDVW......
119619369   ......ALHKY......IVQYL......KIFME......IVHRD......SDFGT......PADIW......
121583542   ......ALHKY......IVQYL......KIFME......IVHRD......SDFGT......PADIW......
119901731   ......KIFEG......LVRYF......YIFME......IVHRD......GDFGC......AADIW......
119901572   ......ALHKH......IVQYL......KIFME......IVHRD......SDFGT......AADIW......
119912558   ......QLLKN......IVQYY......TIFME......IVHRD......GDFGA......KADVW......
119913105   ......RMMSH......IIRML......NLFIE......IIHRD......ADFGA......SCDVW......
119920534   ......ALHKY......IVQYL......KIFME......IVHRD......SDFGT......PADIW......
123121740   ......ALHKR......IVRYL......KIFME......IVHRD......SDFGT......AADIW......
160012147   ......ALHKY......IVQYL......KIFME......IVHRD......SDFGT......PADIW......
123187083   ......NVLEM......IVQYY......NIFME......IVHRD......VDFGA......SDDIW......
124267503   ......WLGRT......GQGFV......YAVFD......VVHRD......LDLGV......ASDLY......
124360510   ......RILSS......IVTYK......NLFME......VVHCD......GDFGC......PCDVW......
145475057   ......QMLSK......IVRYL......NIFLE......VIHRD......ADFGS......KADIW......
145477723   ......EILSQ......IVRYY......NIFLE......VIHRD......ADFGS......KADIW......
145479577   ......KLLKK......IIKYY......DIALE......VIHRD......SDFGT......ESDIW......
145477291   ......DLLSL......IVSYY......K----......IMHRD......ADFGS......KADIW......
145484651   ......EILSK......IVRYY......NIFLE......VIHRD......ADFGS......KADIW......
145488320   ......DLLSL......IVSYY......NIFLE......IMHRD......ADFGS......KADIW......
145490441   ......IILSK......IVQYY......NVYLE......VVHRD......ADFGS......RVDVW......
145493119   ......RVLSK......IVKYL......NLFLE......IIHRD......ADFGS......FADIW......
145497971   ......EMLSK......IVRYM......NIFLE......VIHRD......ADFGS......KADIW......
145497051   ......YLLKK......IVKYI......NIILE......IVHRD......ADFGV......SCDIW......
145503882   ......ELLKS......IIRYV......LLYLE......IIHRD......GDFGC......ASDIW......
145502697   ......KILQQ......IVEYY......SILLE......IIHRD......ADFGC......YSDIW......
145523369   ......QILSQ......IVEYY......SILLE......IIHRD......ADFGC......YSDIW......
145523986   ......KILQQ......IVEYY......SILLE......IIHRD......ADFGC......YSDIW......
145527812   ......EMLSR......IVRYI......NIFLE......VIHRD......ADFGS......KADIW......
145524998   ......QVLSL......IVEYY......SIFLE......IIHRD......ADFGC......YSDIW......
145531844   ......DLLSL......-AQEH......REDPE......IMHRD......ADFGS......KADIW......
145539688   ......KLLKK......IIKYY......DIALE......VIHRD......SDFGT......ESDIW......
145542686   ......DLLKR......IIRYA......LLYLE......IIHRD......GDFGC......ASDIW......
145539221   ......DLLKS......IIRYV......LLYLE......IIHRD......GDFGC......ASDIW......
145545772   ......IILSK......IVQYY......NVYLE......VVHRD......ADFGS......RVDVW......
145546131   ......SFLQI......IIKYI......LIYQE......ILHLD......SDFGC......PADIW......
145551743   ......SFLQI......IIKYI......LIYQE......ILHLD......SDFGC......PADIW......
125591268   ......SLLSR......IVQYY......YIYLE......TVHRD......ADFGM......AVDIW......
125527387   ......RILSG......VLPCL......QLFLE......LVHGD......ADFGC......AADVW......
125527385   ......GMMSG......VVPCI......NLFLE......LVHGD......ADFGC......AADVW......
125527388   ......RVLSG......IVPCL......QLFLE......LVHGD......TDFGC......AADVW......
125527389   ......RVMSG......IVPCL......QLFLE......LVHGD......ADFGC......AADVW......
125539881   ......DMLKQ......IVQYY......SIYLE......TVHRD......ADFGM......AVDIW......
125540601   ......LLLNR......IVRYY......YIYLE......TVHRD......ADFGM......AVDIW......
125531043   ......SVMSG......VLRCL......QLLLE......VVHGD......ADFGC......AADVW......
125531043   ......SVMSG......VLRCL......QLLLE......VVHGD......ADFGC......AADVW......
125533751   ......VLLSQ......IVQYY......SVYLE......TVHRD......ADFGM......SVDIW......
125531042   ......SVMSW......VLKCL......GLFLE......VVHGD......TDFGC......AADVW......
125539219   ......GMLSA......VVPCV......DLFLE......IAHCD......ADFGC......AADIW......
125548393   ......DMLRQ......IVQYY......SIYLE......TVHRD......ADFGM......PVDIW......
125549324   ......SLLSR......IVQYY......YIYLE......TVHRD......ADFGM......AVDIW......
125545819   ......KFLSQ......IVQYY......YIYLE......IMHRD......ADFGM......AVDIW......
125541283   ......SLLSR......IVQYF......YIFLE......VLHRD......ADFGL......PADIW......
125543194   ......ALLSQ......IVQYY......YIFLE......IVHRD......ADFGL......EADIW......
125547787   ......ALLSR......IVQYY......YVFLE......IVHRD......ADFGL......AADIW......
125550085   ......DLLKN......IVKYL......HIILE......VIHRD......ADFGV......ASDIW......
125543449   ......RILRR......VVEYL......NLHVE......VVHGD......ADFGA......ASDVW......
125553189   ......GILAS......VLPCF......GLLLE......MVHGD......ADFGC......AADVW......
125553190   ......GVMAG......VVPCI......QMFLE......LVHGD......ADFGC......AADVW......
125563562   ......KLLKN......IVRYL......NILLE......IIHRD......ADFGA......SADIW......
125576542   ......VLLSQ......IVQYY......SVYLE......TVHRD......ADFGM......SVDIW......
125571704   ......RILSG......VLPCL......QLFLE......LVHGD......ADFGC......AADVW......
125571707   ......RVMSG......IVPCL......QLFLE......LVHGD......ADFGC......AADVW......
125590477   ......DMLRQ......IVQYY......SIYLE......TVHRD......ADFGM......PVDIW......
125588018   ......KFLSQ......IVQYY......YIYLE......IMHRD......ADFGM......AVDIW......
125583824   ......SLLSR......IVQYF......YIFLE......VLHRD......ADFGL......PADIW......
```

```
125582508    ......DMLKQ.....IVQYY.....SIYLE.....TVHRD.....ADFGM.....AVDIW.....
125585897    ......RILRR.....VVEYL.....NLHME.....VVHGD.....ADFGA.....RADA-.....
125585675    ......ALLSQ.....IVQYY.....YIFLE.....IVHRD.....ADFGL.....EADIW.....
125583181    ......LLLNR.....IVRYY.....YIYLE.....TVHRD.....ADFGM.....AVDIW.....
125595089    ......GVMAG.....VVPCI.....QMFLE.....LVHGD.....ADFGC.....AADVW.....
125591937    ......DLLKN.....IVKYL.....HIILE.....VIHRD.....ADFGV.....ASDIW.....
125598939    ......KFLSQ.....IVQYY.....YIYLE.....IMHRD.....ADFGM.....AVDIW.....
125605546    ......KLLKN.....IVRYL.....NILLE.....IIHRD.....ADFGA.....SADIW.....
125812677    ......QLLKN.....IVQYY.....TIFME.....IVHRD.....GDFGA.....KADVW.....
125825889    ......DLLKN.....IVGFL.....SIFME.....VIHRD.....IDFGC.....KSDIW.....
125832196    ......RMMGL.....IIRML.....NLFVE.....IIHRD.....ADFGA.....SCDVW.....
125833646    ......KIFEG.....LVRYF.....YIFME.....IVHRD.....GDFGC.....AADIW.....
125845732    ......ALHKH.....IVQYL.....KIFME.....IVHRD.....SDFGT.....PADIW.....
125858064    ......ALHRR.....IVRYL.....KIFME.....IVHRD.....SDFGT.....AADIW.....
125858938    ......ALHRR.....IVRYL.....KIFME.....IVHRD.....SDFGT.....AADIW.....
126311262    ......KIFEG.....LVRYF.....YIFME.....IVHRD.....GDFGC.....AADIW.....
126311126    ......ALHKH.....IVQYL.....KIFME.....IVHRD.....SDFGT.....AADIW.....
126308646    ......QLLKN.....IVQYY.....TIFME.....IVHRD.....GDFGA.....KADVW.....
126316703    ......RMMSH.....IIRML.....NLFIE.....IIHRD.....ADFGA.....SCDVW.....
126325607    ......ALHKY.....IVQYL.....KIFME.....IVHRD.....SDFGT.....PADIW.....
126326077    ......QLLKN.....IVQYY.....SIFME.....IVHRD.....GDFGA.....KADIW.....
126328697    ......ALHKR.....IVQYL.....KIFME.....IIHRD.....SDFGT.....AADIW.....
146324065    ......ELLQG.....IVQYL.....NIFLE.....IIHRD.....SDFGI.....KADIW.....
145232891    ......DTMQH.....IVQYL.....SIYLE.....ILHRD.....SDFGI.....KVDIW.....
145256666    ......DLLKN.....IVKYQ.....NIILE.....VIHRD.....ADFGV.....ASDIW.....
154332488    ......DVLCA.....IIRFL.....SIFTE.....IAHRD.....SDFGS.....AADIW.....
154331117    ......SVLSK.....IVQYY.....NILLE.....IVHSD.....TDFGT.....AGDIW.....
154332075    ......ALMKR.....CVQYY.....NIFME.....IVHRD.....ADFGC.....KSDIW.....
154332396    ......EVLTA.....IVGFF.....LMYLE.....YAHLD.....ADLGC.....PADVW.....
154334363    ......TLLKH.....IVRYL.....RIYMD.....IIHRD.....ADLGT.....AADIW.....
154331498    ......EIMSS.....IVRYY.....SIFME.....IVHRD.....TDFGT.....ACDVW.....
154334630    ......STMCN.....IIHYF.....RVFME.....YVHGD.....GDFGT.....PSDIW.....
154338874    ......AYMRE.....LVKYY.....QIIME.....VCHRD.....ADFGV.....SADIW.....
154336994    ......LILQR.....VVQLL.....VIFME.....IIHRD.....SDFGA.....ESDIW.....
154335812    ......EIHRK.....IIRYL.....YVYLE.....VAHRD.....ADFDQ.....KADIW.....
154339902    ......RAMSS.....CVRYL.....YIIME.....IVHRD.....VDFGC.....KSDIW.....
154341499    ......RVMRR.....VVTYL.....RIFME.....ILHRD.....GDFGQ.....EADVW.....
154341955    ......NMLRE.....IIRYF.....LVFME.....VVHRD.....ADFGC.....KADIW.....
154342997    ......RMWSM.....VITFY.....LLVME.....VIHGD.....SDFGC.....KSDVW.....
154342863    ......ALLKS.....IVSYF.....RIAME.....IFHRD.....CDFGC.....KADIW.....
154338080    ......SIMCS.....IVHYF.....SLFME.....IAHRD.....TDFGT.....SCDIW.....
154338361    ......NMMHR.....LCTFK.....SIFME.....IIHRD.....VDFGA.....QADVW.....
154335130    ......TVLTE.....IVRVV.....RIYME.....IIHRD.....TDFGT.....ASDIW.....
154343003    ......CTCAK.....IVPYI.....AIIME.....TTHGD.....SDFGS.....ASDIY.....
154345578    ......EALSQ.....IVGYD.....FIICE.....WLHCD.....ADFGA.....QADIY.....
146085119    ......LAMTT.....IIQYF.....LLYME.....YVHGD.....GDFGT.....KADIW.....
146083342    ......NLMRS.....IVTYY.....EVFLE.....VVHRD.....ADFGC.....AADIW.....
146096311    ......CTCAK.....IVPYI.....AIIME.....TTHGD.....SDFGS.....ASDIY.....
146089951    ......SIMCS.....IVHYF.....SLFME.....IAHRD.....TDFGT.....SCDIW.....
146090842    ......RAMSS.....CVRYL.....YIIME.....ILHRD.....VDFGC.....KSDVW.....
146075612    ......EIMSS.....IVRYY.....SIFME.....IVHRD.....TDFGT.....ACDVW.....
146092823    ......EIHRK.....IIRYL.....YVYLE.....VAHRD.....ADFDQ.....KADIW.....
146077490    ......EVLAA.....IVGFF.....LMYLE.....YAHLD.....ADLGC.....PADVW.....
146103228    ......ETLSQ.....IVGYD.....FIICE.....WLHCD.....ADFGA.....QADIY.....
146096069    ......ALLKS.....IVSYF.....RIAME.....IFHRD.....CDFGC.....KADIW.....
146103798    ......GILCK.....IIHYF.....NLFMA.....IIHRD.....SDFGT.....ESDIW.....
146081701    ......TLLKQ.....IVRYL.....RIYMD.....IIHRD.....ADLGT.....AADIW.....
146089113    ......AYMRE.....LVEYY.....QIIME.....VCHRD.....ADFGV.....SADIW.....
146093592    ......RVMRR.....VVTYL.....RIFME.....ILHRD.....GDFGQ.....EADVW.....
146077702    ......DVLCA.....IIRFL.....RVFTE.....IAHRD.....SDFGS.....AVDIW.....
146088036    ......NMMHR.....LCTFK.....CMFME.....IIHRD.....VDFGA.....KADVW.....
146094088    ......NMLRE.....IIRYF.....LVFME.....VVHRD.....ADFGC.....KADIW.....
146096305    ......QMWSM.....VITFY.....LLVME.....VIHGD.....SDFGC.....KSDVW.....
146085694    ......LILQR.....VVQLL.....VIFME.....VIHRD.....SDFGA.....ESDIW.....
146083302    ......TVLTE.....IVRVV.....RIYME.....VIHRD.....TDFGT.....ASDIW.....
146076785    ......ALMRR.....CVQYY.....NIFME.....IVHRD.....ADFGC.....KSDIW.....
154346148    ......EILCQ.....IIHYF.....NLFMA.....IIHRD.....SDFGT.....ESDIW.....
145239589    ......GVLEV.....IVSYH.....YIFME.....IIHRD.....VDFGA.....AVDIW.....
145253689    ......ELLQG.....IVQYL.....NIFLE.....IIHRD.....SDFGI.....KADIW.....
145355589    ......ELLSR.....IVKYE.....YIMLE.....VVHRD.....ADFGV.....AADIW.....
145353562    ......DVLSR.....IVRYI.....YIFLE.....VVHRD.....ADFGM.....EADIW.....
```

```
145607237    ......DLLKN.....LAHTL.....NILE.....VIHRD.....ADFGV.....ASDIW.....
145611712    ......RVLES.....VVSYY.....YIFME.....IAHRD.....VDFGA.....AVDVW.....
145610819    ......ETMQH.....IVQYL.....SIFLE.....ILHRD.....SDFGI.....KVDIW.....
145608830    ......SLLRD.....IVQYL.....NIFLE.....IIHRD.....SDFGI.....KADIW.....
145336768    ......KLLKN.....IVRYL.....NILLE.....IMHRD.....ADFGA.....SADIW.....
146184771    ......HLLKK.....IVTYI.....AIILE.....IVHRD.....TDFGV.....SCDIW.....
146417099    ......ETMKD.....IVQYL.....SLFLE.....ILHRD.....SDFGI.....KVDVW.....
146416847    ......TVLEM.....IVQYF.....YIFME.....VVHRD.....VDFGA.....SVDIW.....
146416671    ......SLLKV.....IVRYL.....NIFLE.....IIHRD.....SDFGI.....KADIW.....
146412586    ......DLLKI.....IVKYH.....NVFLE.....VVHRD.....ADFGV.....VSDIW.....
149248712    ......MLLKE.....IVRYF.....NIFLE.....IIHRD.....GDFGI.....KADIW.....
149247406    ......DLLKI.....IVKYH.....NLVLE.....VVHRD.....ADFGV.....SSDIW.....
149237208    ......TVLEM.....VVQYF.....YIFME.....VVHRD.....VDFGA.....SVDIW.....
149235842    ......KNMKD.....IVQYL.....CLFME.....IIHRD.....SDFGI.....KVDIW.....
147776082    ......EILHR.....IVDCF.....LVYLE.....FVHCD.....ADFGH.....PKDIW.....
147778640    ......GVLRR.....VVRYL.....NLHVE.....LVHCD.....ADFGA.....ESDVW.....
147792548    ......VLLSR.....IVQYY.....YIYLE.....TVHRD.....ADFGM.....AVDIW.....
147788138    ......ECLKQ.....-----.....SVYLE.....TVHRD.....ADFGM.....AVDIW.....
147816313    ......SFLSQ.....IVKYL.....NVFME.....LVHCD.....ADLGC.....PADVW.....
147834028    ......EILGV.....VVRCY.....NLLLE.....YVHCD.....GDFGL.....LSDIW.....
147828248    ......DLLKN.....IVKYL.....HIILE.....VIHRD.....ADFGV.....ASDIW.....
147841887    ......RILSS.....IVGYK.....NLFIE.....LVHCD.....ADFGC.....PSDVW.....
147866345    ......TTSNA.....AQRYL.....NILLE.....IMHRD.....ADFGA.....DPDIW.....
148671498    ......ALHKH.....IVQYL.....KIFME.....IVHRD.....SDFGT.....AADIW.....
148670127    ......KIFEG.....LVRYF.....YIFME.....IVHRD.....GDFGC.....AADIW.....
148686472    ......RMMGH.....IIRML.....NLFIE.....IIHRD.....ADFGA.....SCDVW.....
148686473    ......RMMGH.....IIRML.....NLFIE.....IIHRD.....ADFGA.....SCDVW.....
148702316    ......QLLKN.....IVQYY.....TIFME.....IVHRD.....GDFGA.....KADVW.....
148698129    ......ALHKR.....IVRYL.....KIFME.....IVHRD.....SDFGT.....AADIW.....
148887396    ......RMMSH.....IIRML.....NLFIE.....IIHRD.....ADFGA.....SCDVW.....
148922282    ......KIFEG.....LVRYF.....YIFME.....IVHRD.....GDFGC.....AADIW.....
157822757    ......KIFEG.....LVRYF.....YIFME.....IVHRD.....GDFGC.....AADIW.....
149039630    ......ALHKH.....IVQYL.....KIFME.....IVHRD.....SDFGT.....AADIW.....
149059334    ......RMMSH.....IIRML.....NLFIE.....IIHRD.....ADFGA.....SCDVW.....
149059333    ......RMMSH.....IIRML.....NLFIE.....IIHRD.....ADFGA.....SCDVW.....
149260603    ......ALHKH.....IVQYL.....KIFME.....IVHRD.....SDFGT.....AADIW.....
150951556    ......DLLKI.....IVKYH.....NILLE.....VVHRD.....ADFGL.....ASDIW.....
150951062    ......TVLEM.....VVQYF.....YIFME.....VVHRD.....VDFGA.....NVDIW.....
150864582    ......TLLKS.....IVRYY.....NIFLE.....IIHRD.....GDFGI.....KADIW.....
150864689    ......ETMKD.....IVQYL.....SLFLE.....ILHRD.....SDFGI.....KIDIW.....
149614491    ......QLLKN.....IVQYY.....SIFME.....IVHRD.....GDFGA.....KADIW.....
149638284    ......ALHKY.....IVRYL.....KIFME.....IVHRD.....SDFGT.....PADIW.....
149639468    ......RMMSH.....IIRML.....NLFIE.....IIHRD.....ADFGA.....SCDVW.....
149642474    ......ALHKH.....IVQYL.....KIFME.....IVHRD.....SDFGT.....AADIW.....
149695025    ......ALHKR.....IVRYL.....KIFME.....IVHRD.....SDFGT.....AADIW.....
149723655    ......QLLKN.....IVQYY.....TIFME.....IVHRD.....GDFGA.....KADVW.....
149730546    ......DLLKA.....IVAYL.....SIFME.....VVHRD.....IDFGC.....KSDIW.....
149744067    ......KIFEG.....LVRYF.....YIFME.....IVHRD.....GDFGC.....AADIW.....
149744374    ......ALHKY.....IVQYL.....KIFME.....IVHRD.....SDFGT.....PADIW.....
149755280    ......QLLKN.....IVQYY.....SIFME.....IVHRD.....GDFGA.....KADVW.....
55961173     ......ALHRR.....IVRYL.....KIFME.....IVHRD.....SDFGT.....AADIW.....
150036250    ......KLLKD.....IVRYL.....NILLE.....IMHRD.....ADFGA.....SADIW.....
154287882    ......GVLEV.....IISYY.....YIFME.....IVHRD.....VDFGA.....HMDIW.....
154270317    ......DLLQG.....IVQYL.....NIFLE.....IIHRD.....SDFGI.....KADIW.....
154276278    ......DTMQH.....IVQYL.....SIYLE.....ILHRD.....SDFGI.....KVDIW.....
154278701    ......--LRV.....IVKYH.....NIILE.....VIHRD.....ADFGV.....ASDIW.....
154315134    ......HVLEV.....VVSYH.....YIFME.....IVHRD.....VDFGA.....SVDVW.....
154300312    ......DTMQH.....IVQYL.....SIFLE.....ILHRD.....SDFGI.....KVDIW.....
115450285    ......QTMSL.....VIRAY.....WVIMP.....HIHRD.....GDFGV.....KADIW.....
108711026    ......RMLGA.....IVEIY.....TIMME.....VIHRD.....SDFDN.....EVDIW.....
157129773    ......QAMSS.....VVTYH.....WLVLR.....QIHRD.....ADFGV.....KADIW.....
157124824    ......LVLRD.....IPDFA.....WFVLE.....CMHRD.....VDFGV.....RCDVW.....
157124822    ......LVLRD.....IPDFA.....WFVLE.....CMHRD.....VDFGV.....RCDVW.....
157117354    ......VIMRD.....IVETY.....WVVME.....VIHRD.....SDFGF.....EVDIW.....
157114193    ......NVLKK.....IATYY.....WLVME.....VIHRD.....VDFGV.....RSDLW.....
157113643    ......SIMQQ.....VVKYY.....WIVME.....KIHRD.....ADFGV.....VADIW.....
157130361    ......LVMRE.....VVNYL.....WVVME.....VIHRD.....TDFGF.....KVDLW.....
157106155    ......RILRD.....LLDFY.....WFILE.....VIHRD.....CDFGL.....RSDVW.....
157104977    ......MVLSQ.....VTKYF.....WIIME.....KLHRD.....ADFGV.....KADIW.....
157137060    ......NVLKD.....LVNFL.....WVILE.....IIHRD.....TDFGF.....KVDIW.....
157117698    ......RFLRQ.....TIEYK.....WLVME.....RIHRD.....ADFG-.....KVDVW.....
109044311    ......NMLKK.....IATYY.....WLVME.....VIHRD.....VDFGV.....KSDLW.....
```

```
109044299     ......NMLKK......IATYY......WLVME......VIHRD......VDFGV......KSDLW......
109044320     ......NMLKK......IATYY......WLVME......VIHRD......VDFGV......KSDLW......
109044296     ......NMLKK......IATYY......WLVME......VIHRD......VDFGV......KSDLW......
109044290     ......NMLKK......IATYY......WLVME......VIHRD......VDFGV......KSDLW......
109044287     ......NMLKK......IATYY......WLVME......VIHRD......VDFGV......KSDLW......
109044305     ......NMLKK......IATYY......WLVME......VIHRD......VDFGV......KSDLW......
109044302     ......NMLKK......IATYY......WLVME......VIHRD......VDFGV......KSDLW......
109044293     ......NMLKK......IATYY......WLVME......VIHRD......VDFGV......KSDLW......
109044314     ......NMLKK......IATYY......WLVME......VIHRD......VDFGV......KSDLW......
109044308     ......NMLKK......IATYY......WLVME......VIHRD......VDFGV......KSDLW......
109044332     ......NMLKK......IATYY......WLVME......VIHRD......VDFGV......KSDLW......
109044329     ......NMLKK......IATYY......WLVME......VIHRD......VDFGV......KSDLW......
109044326     ......NMLKK......IATYY......WLVME......VIHRD......VDFGV......KSDLW......
109044317     ......NMLKK......IATYY......WLVME......VIHRD......VDFGV......KSDLW......
109044323     ......NMLKK......IATYY......WLVME......VIHRD......VDFGV......KSDLW......
109054049     ......LVMKE......IVNFL......FVVME......VIHRD......TDFGF......KVDIW......
109054046     ......LVMKE......IVNFL......FVVME......VIHRD......TDFGF......KVDIW......
109080618     ......VIMRD......VVEMY......WVLME......VIHRD......SDFGF......EVDIW......
109083529     ......FMVKE......IVAYF......WICME......KMHRD......ADFGV......LCDIW......
109087126     ......SIMQQ......VVKYY......WIVME......KIHRD......ADFGV......VADIW......
109088453     ......NILKL......VVRFY......WLVLE......TIHRD......VDFGV......RCDTW......
109090486     ......DILAS......IVKLL......WILIE......IIHRD......ADFGV......KADVW......
109090484     ......DILAS......IVKLL......WILIE......IIHRD......ADFGV......KADVW......
109092911     ......VIMRD......VVDMY......WVVME......VIHRD......SDFGF......EVDIW......
109098928     ......KFLRQ......TIEYK......WLVME......LIHRD......ADFG-......KVDIW......
109099935     ......QAMSQ......VVTYY......WLVMK......QIHRD......ADFGV......KADMW......
109100024     ......NILQF......VVKFY......WLVLE......IIHRD......VDFGV......RCDVW......
109100020     ......NILQF......VVKFY......WLVLE......IIHRD......VDFGV......RCDVW......
109100026     ......NILQF......VVKFY......WLVLE......IIHRD......VDFGV......RCDVW......
109100022     ......NILQF......VVKFY......WLVLE......IIHRD......VDFGV......RCDVW......
109101727     ......TVLSQ......ITRYF......WIIME......KIHRD......ADFGV......KADIW......
109101729     ......TVLSQ......ITRYF......WIIME......KIHRD......ADFGV......KADIW......
109101721     ......TVLSQ......ITRYF......WIIME......KIHRD......ADFGV......KADIW......
109102780     ......IMMKD......IVAYF......WICME......KMHRD......ADFGV......LCDLW......
109104112     ......NMLKK......IATYY......WLVME......VIHRD......VDFGV......RSDLW......
109108017     ......LVMRE......IVNYL......WVVME......VIHRD......TDFGF......KVDIW......
109108019     ......LVMRE......IVNYL......WVVME......VIHRD......TDFGF......KVDIW......
109108027     ......LVMRE......IVNYL......WVVME......VIHRD......TDFGF......KVDIW......
109108031     ......LVMRE......IVNYL......WVVME......VIHRD......TDFGF......KVDIW......
109108021     ......LVMRE......IVNYL......WVVME......VIHRD......TDFGF......KVDIW......
109108029     ......LVMRE......IVNYL......WVVME......VIHRD......TDFGF......KVDIW......
109113001     ......NMLKK......IATYY......WLVME......VIHRD......VDFGV......RSDIW......
109113828     ......KFLQR......SIEYK......WLVME......MIHRD......ADFG-......KVDVW......
109113830     ......KFLQR......SIEYK......WLVME......MIHRD......ADFG-......KVDVW......
109113832     ......KFLQR......SIEYK......WLVME......MIHRD......ADFG-......KVDVW......
109121156     ......TVLSQ......VTKYY......WIIME......KIHRD......ADFGV......KADIW......
109121162     ......TVLSQ......VTKYY......WIIME......KIHRD......ADFGV......KADIW......
109121158     ......TVLSQ......VTKYY......-----......KIHRD......ADFGV......KADIW......
109121172     ......TVLSQ......VTKYY......WIIME......KIHRD......ADFGV......KADIW......
109121164     ......TVLSQ......VTKYY......WIIME......KIHRD......ADFGV......KADIW......
109121166     ......TVLSQ......VTKYY......WIIME......KIHRD......ADFGV......KADIW......
109121168     ......TVLSQ......VTKYY......WIIME......KIHRD......ADFGV......KADIW......
109121160     ......TVLSQ......VTKYY......WIIME......KIHRD......ADFGV......KADIW......
109124668     ......VIMRD......VVEMY......WVVME......VIHRD......SDFGF......EVDIW......
109124612     ......LILKT......IVAYH......WICME......KIHRD......GDFAL......LCDIW......
109128106     ......RFLQK......TIQYR......WLVME......MIHRD......GDFG-......KVDVW......
109131920     ......LVMRE......IVNYL......WVVME......VIHRD......TDFGF......KVDIW......
109131922     ......LVMRE......IVNYL......WVVME......VIHRD......TDFGF......KVDIW......
109131924     ......LVMRE......IVNYL......WVVME......VIHRD......TDFGF......KVDIW......
109131926     ......LVMRE......IVNYL......WVVME......VIHRD......TDFGF......KVDIW......
109132321     ......TVLSQ......VTKYY......WIIME......KIHRD......ADFGV......KADIW......
157819679     ......VIMRD......VVEMY......WVVME......VIHRD......SDFGF......EVDIW......
157817492     ......VIMRD......VVEMY......WVLME......VIHRD......SDFGF......EVDIW......
149048594     ......NMLKK......IATYY......WLVME......VIHRD......VDFGV......KSDLW......
109468272     ......NILQF......VVKFY......WLVLE......IIHRD......VDFGV......RCDVW......
109470060     ......NILQF......VVKFY......WLVLE......IIHRD......VDFGV......RCDVW......
109477864     ......IMMKD......IVAYF......WICME......KMHRD......ADFGV......LCDLW......
109478244     ......FMVKE......IVAYF......WICME......KMHRD......ADFGV......LCDIW......
109487956     ......EILAT......IVKLL......WIMIE......IIHRD......ADFGV......KADIW......
109488332     ......NMLKK......IATYY......WLVME......VIHRD......VDFGV......RSDIW......
109490494     ......EILAT......IVKLL......WIMIE......IIHRD......ADFGV......KADIW......
109490492     ......EILAT......IVKLL......WIMIE......IIHRD......ADFGV......KADIW......
109491165     ......NMLKK......IATYY......WLVME......VIHRD......VDFGV......RSDIW......
```

```
109491174    ......NMLKK.....IATYY.....WLVME.....VIHRD.....VDFGV.....RSDIW.....
109491177    ......NMLKK.....IATYY.....WLVME.....VIHRD.....VDFGV.....RSDIW.....
109491169    ......NMLKK.....IATYY.....WLVME.....VIHRD.....VDFGV.....RSDIW.....
109491179    ......NMLKK.....IATYY.....WLVME.....VIHRD.....VDFGV.....RSDIW.....
109491172    ......NMLKK.....IATYY.....WLVME.....VIHRD.....VDFGV.....RSDIW.....
109491167    ......NMLKK.....IATYY.....WLVME.....VIHRD.....VDFGV.....RSDIW.....
109502040    ......TVLSQ.....VTKYY.....WIIME.....KIHRD.....ADFGV.....KADIW.....
109502964    ......TVLSQ.....VTKYY.....WIIME.....KIHRD.....ADFGV.....KADIW.....
109505313    ......NILRT.....VVRFY.....WLVLE.....TIHRD.....VDFGV.....RCDTW.....
109506253    ......NILRT.....VVRFY.....WLVLE.....TIHRD.....VDFGV.....RCDTW.....
115495963    ......VIMRD.....VVEMY.....WVVME.....VIHRD.....SDFGF.....EVDIW.....
109511135    ......NLLRK.....IVTFY.....WMVME.....VIHRD.....VDFGV.....RVSLK.....
109511211    ......TVLSQ.....VTKYY.....WIIME.....KIHRD.....ADFGV.....KADIW.....
109512396    ......NLLRK.....IVTFY.....WMVME.....VIHRD.....VDFGV.....RSDVW.....
115482966    ......QIMSL.....VIRAY.....WVVMP.....QIHRD.....GDFGV.....KADIW.....
110331883    ......LILKT.....IVAYH.....WICME.....KIHRD.....ADFGI.....LCDIW.....
110760264    ......SIMQQ.....VVKYY.....WIVME.....KIHRD.....ADFGV.....VADIW.....
110760400    ......NVLKR.....IATYY.....WLVME.....VIHRD.....VDFGV.....RSDLW.....
110762094    ......MVLSQ.....VTKYY.....WIIME.....KLHRD.....ADFGV.....KADIW.....
110762628    ......LVLRD.....IPLFY.....WFVME.....CMHRD.....VDFGV.....RCDVW.....
110763592    ......DILSE.....VVELH.....WMLIE.....VIHRD.....ADFGV.....KVDIW.....
110764306    ......LVMRE.....VVNYL.....WVVME.....VIHRD.....TDFGF.....KVDIW.....
110765948    ......VIMRD.....IVEMY.....WVVME.....VIHRD.....SDFGF.....EVDIW.....
111021377    ......QTLAQ.....IVRVF.....RLLMQ.....VLHRD.....ADFNI.....RSDIF.....
111017527    ......DHAAR.....IVSVF.....WIAMQ.....VLHRD.....TDFGI.....RADVY.....
111018124    ......RSVAR.....LVAVY.....FLVME.....LVHRD.....ADFGL.....RSDVY.....
111025322    ......DHAAR.....IVAVY.....WIAMQ.....VLHRD.....ADFGI.....RADVY.....
110611908    ......LILKT.....IVAYH.....WICME.....KIHRD.....ADFGI.....LCDIW.....
111054911    ......NILKL.....VVRFY.....WLVLE.....TIHRD.....VDFGV.....RCDTW.....
169602102    ......MILSG.....VTRYY.....WIVME.....KLHRD.....ADFGV.....KADIW.....
111120334    ......NILQF.....VVKFY.....WLVLE.....IIHRD.....VDFGV.....RCDVW.....
111220804    ......QISAA.....VVTTL.....WIVMD.....VVHRD.....TDFGA.....VADVF.....
111222679    ......AAAKL.....TAPVV.....WLATQ.....IVHRD.....IDFGV.....AADVF.....
111223812    ......DAARR.....VATVL.....WMATE.....VVHRD.....IDFGI.....PADIF.....
111221665    ......RAAAA.....VARLV.....WLATE.....VVHRD.....IDFGI.....AADVF.....
112181194    ......LVMRE.....IVNYL.....WVVME.....VIHRD.....TDFGF.....KVDIW.....
111380697    ......LVMKD.....IVNFL.....WVVME.....VIHRD.....TDFGF.....KVDIW.....
115455273    ......RMLGA.....IVEIY.....TIMME.....VIHRD.....SDFDN.....EVDIW.....
145593410    ......RAIAR.....VVRIF.....WIVME.....VMHRD.....TDFGL.....EADLW.....
159899485    ......ITAGN.....IVTIF.....YIAME.....AVHRD.....TDFGI.....RSDLY.....
145593389    ......RAAAA.....VVQVY.....WIVME.....VLHRD.....TDFGV.....PSDLF.....
114049948    ......EAARR.....TAELV.....WLATL.....VVHRD.....IDFGV.....ATDVF.....
114052104    ......NMLKK.....IATYY.....WLVME.....VIHRD.....VDFGV.....RSDIW.....
118404680    ......EILAT.....IVKLL.....WIMIE.....MIHRD.....ADFGV.....KADIW.....
148224626    ......AIMQQ.....VVKYY.....WIVME.....KIHRD.....ADFGV.....VADIW.....
115399248    ......LVMKD.....IVNFL.....WVVME.....VIHRD.....TDFGF.....KVDIW.....
115438438    ......IVMKD.....IVNFL.....WVVME.....IIHRD.....TDFGF.....KVDCW.....
115395210    ......AILSE.....VTRYH.....WIIME.....KLHRD.....ADFGV.....KADIW.....
115384336    ......KVMKQ.....INELI.....WLVCE.....IIHRD.....CDFGV.....EIDVW.....
114581624    ......NILPF.....VVKFY.....WLVLE.....IIHRD.....VDFGV.....RCDVW.....
114584361    ......TVLSQ.....ITRYF.....WIIME.....KIHRD.....ADFGV.....KADIW.....
114577097    ......IMMKD.....IVAYF.....WICME.....KMHRD.....ADFGV.....LCDLW.....
114579265    ......NMLKK.....IATYY.....WLVME.....VIHRD.....VDFGV.....RSDLW.....
114581576    ......QAMSQ.....VVTYY.....WLVMK.....QIHRD.....ADFGV.....KADMW.....
114590382    ......NMLKK.....IATYY.....WLVME.....VIHRD.....VDFGV.....KSDLW.....
114590366    ......NMLKK.....IATYY.....WLVME.....VIHRD.....VDFGV.....KSDLW.....
114590352    ......NMLKK.....IATYY.....WLVME.....VIHRD.....VDFGV.....KSDLW.....
114590368    ......NMLKK.....IATYY.....WLVME.....VIHRD.....VDFGV.....KSDLW.....
114590376    ......NMLKK.....IATYY.....WLVME.....VIHRD.....VDFGV.....KSDLW.....
114591275    ......LVMKE.....IVKLL.....FVVME.....VIHRD.....TDFGF.....KVDIW.....
114590356    ......NMLKK.....IATYY.....WLVME.....VIHRD.....VDFGV.....KSDLW.....
114590362    ......NMLKK.....IATYY.....WLVME.....VIHRD.....VDFGV.....KSDLW.....
114590360    ......NMLKK.....IATYY.....WLVME.....VIHRD.....VDFGV.....KSDLW.....
114590384    ......NMLKK.....IATYY.....WLVME.....VIHRD.....VDFGV.....KSDLW.....
114590358    ......NMLKK.....IATYY.....WLVME.....VIHRD.....VDFGV.....KSDLW.....
114586046    ......QAMSQ.....IVSYY.....WLVMK.....QIHRD.....ADFGV.....KADIW.....
114591281    ......LVMKE.....IVNCI.....FVVME.....VIHRD.....TDFGF.....KVDIW.....
114590372    ......NMLKK.....IATYY.....WLVME.....VIHRD.....VDFGV.....KSDLW.....
114590354    ......NMLKK.....IATYY.....WLVME.....VIHRD.....VDFGV.....KSDLW.....
114591283    ......LVMKE.....IVNCI.....FVVME.....VIHRD.....TDFGF.....KVDIW.....
114590378    ......NMLKK.....IATYY.....WLVME.....VIHRD.....VDFGV.....KSDLW.....
114590380    ......NMLKK.....IATYY.....WLVME.....VIHRD.....VDFGV.....KSDLW.....
114591277    ......LVMKE.....IVXXX.....FVVME.....VIHRD.....TDFGF.....KVDIW.....
114590374    ......NMLKK.....IATYY.....WLVME.....VIHRD.....VDFGV.....KSDLW.....
```

```
114586044    ......QAMSQ......IVSYY......WLVMK......QIHRD......ADFGV......KADIW......
114590370    ......NMLKK......IATYY......WLVME......VIHRD......VDFGV......KSDLW......
114590350    ......NMLKK......IATYY......WLVME......VIHRD......VDFGV......KSDLW......
114590364    ......NMLKK......IATYY......WLVME......VIHRD......VDFGV......KSDLW......
114603379    ......EILAT......IVKLL......WLLVL......IIHRD......ADFGV......KADIW......
114603377    ......EILAT......IVKLL......WLQAQ......IIHRD......ADFGV......KADIW......
114621084    ......SIMQQ......VVKYY......WIVME......KIHRD......ADFGV......VADIW......
114621086    ......SIMQQ......VVKYY......WIVME......KIHRD......ADFGV......VADIW......
114629747    ......NILKL......VVRFY......WLVLE......TIHRD......VDFGV......RCDTW......
114632697    ......DILAS......IVKLL......WILIE......IIHRD......ADFGV......KADVW......
114632695    ......DILAS......IVKLL......WILIE......IIHRD......ADFGV......KADVW......
114632691    ......DILAS......IVKLL......WILIE......IIHRD......ADFGV......KADVW......
114632693    ......DILAS......IVKLL......WILIE......IIHRD......ADFGV......KADVW......
114638367    ......TILRE......VVAYI......WICME......KIHRD......ADFGV......LCDVW......
114638365    ......TILRE......VVAYI......WICME......KIHRD......ADFGV......LCDVW......
114638371    ......TILRE......VVAYI......WICME......KIHRD......ADFGV......LCDVW......
114639476    ......LVMRE......IVNYL......WVVME......VIHRD......TDFGF......KVDIW......
114647343    ......KFLRQ......TIEYK......WLVME......LIHRD......ADFG-......KVDIW......
114650422    ......TVLSQ......VTKYY......WIIME......KIHRD......ADFGV......KADIW......
114650412    ......TVLSQ......VTKYY......WIIME......KIHRD......ADFGV......KADIW......
114650428    ......TVLSQ......VTKYY......WIIME......KIHRD......ADFGV......KADIW......
114650414    ......TVLSQ......VTKYY......-----......KIHRD......ADFGV......KADIW......
114650416    ......TVLSQ......VTKYY......WIIME......KIHRD......ADFGV......KADIW......
114650418    ......TVLSQ......VTKYY......WIIME......KIHRD......ADFGV......KADIW......
114650420    ......TVLSQ......VTKYY......WIIME......KIHRD......ADFGV......KADIW......
114650426    ......TVLSQ......VTKYY......WIIME......KIHRD......ADFGV......KADIW......
114650410    ......TVLSQ......VTKYY......WIIME......KIHRD......ADFGV......KADIW......
114652950    ......FMVKE......IVAYF......WICME......KMHRD......ADFGV......LCDIW......
114652952    ......FMVKE......IVAYF......WICME......KMHRD......ADFGV......LCDIW......
114652938    ......FMVKE......IVAYF......WICME......KMHRD......ADFGV......LCDIW......
114652942    ......FMVKE......IVAYF......WICME......KMHRD......ADFGV......LCDIW......
114662014    ......RFLQK......TIQYR......WLVME......MIHRD......GDFG-......KVDVW......
114665828    ......NMLKK......IATYY......WLVME......VIHRD......VDFGV......RSDIW......
114665850    ......NMLKK......IATYY......WLVME......VIHRD......VDFGV......RSDIW......
114665840    ......NMLKK......IATYY......WLVME......VIHRD......VDFGV......RSDIW......
114665838    ......NMLKK......IATYY......WLVME......VIHRD......VDFGV......RSDIW......
114665848    ......NMLKK......IATYY......WLVME......VIHRD......VDFGV......RSDIW......
114665844    ......NMLKK......IATYY......WLVME......VIHRD......VDFGV......RSDIW......
114665824    ......NMLKK......IATYY......WLVME......VIHRD......VDFGV......RSDIW......
114665836    ......NMLKK......IATYY......WLVME......VIHRD......VDFGV......RSDIW......
114665832    ......NMLKK......IATYY......WLVME......VIHRD......VDFGV......RSDIW......
114665830    ......NMLKK......IATYY......WLVME......VIHRD......VDFGV......RSDIW......
114665842    ......NMLKK......IATYY......WLVME......VIHRD......VDFGV......RSDIW......
114665846    ......NMLKK......IATYY......WLVME......VIHRD......VDFGV......RSDIW......
114665834    ......NMLKK......IATYY......WLVME......VIHRD......VDFGV......RSDIW......
114665826    ......NMLKK......IATYY......WLVME......VIHRD......VDFGV......RSDIW......
114668377    ......KFLQR......SIEYK......WLVME......MIHRD......ADFG-......KVDVW......
114668373    ......KFLQR......SIEYK......WLVME......MIHRD......ADFG-......KVDVW......
114681031    ......VIMRD......VVDMY......WVVME......VIHRD......SDFGF......EVDIW......
114682137    ......SIMQQ......VVKYY......WIVME......KIHRD......ADFGV......VADIW......
114682141    ......SIMQQ......VVKYY......WIVME......KIHRD......ADFGV......VADIW......
114689709    ......NLLRK......IVSFY......WMVME......VIHRD......VDFGV......RSDVW......
114690199    ......TVLSQ......VTKYY......WIIME......KIHRD......ADFGV......KADIW......
114794045    ......RFLQK......TIQYR......WLVME......MIHRD......GDFG-......KVDVW......
116004015    ......LVMRE......IVNYL......WVVME......VIHRD......TDFGF......KVDIW......
115430083    ......NMLKK......IATYY......WLVME......VIHRD......VDFGV......KSDLW......
115374562    ......YAMTK......TLQVF......FITME......YVHCD......MDFGV......RADLY......
115430252    ......QAMSQ......VVTYY......WLVMK......QIHRD......ADFGV......KADMW......
118600963    ......NLLRK......IVSFY......WMVME......VIHRD......VDFGV......RSDVW......
133951926    ......SILRQ......IVRFF......WVVME......VIHRD......TDFGF......RADIW......
115732986    ......TVLSQ......VTKYH......WIIME......KIHRD......ADFGV......KADMW......
115749104    ......QAMCL......VVQYF......WLVMR......QLHRD......ADFGV......KADIW......
115751502    ......LMMKD......IVGYF......WIAME......KMHRD......ADFGV......QCDIW......
115762690    ......VIMRD......IVEMY......WVVME......VIHRD......SDFGF......EVDIW......
115889596    ......SIMQQ......VVKYY......WIVME......KIHRD......ADFGV......KADIW......
115940697    ......NILKL......VVRFY......WLVLE......TIHRD......VDFGV......RCDTW......
115951619    ......RFIHR......CIEFK......WLAME......TIHRD......GDFG-......KVDVW......
116058170    ......ATLRS......VPEYL......YVLVQ......VIHRD......VDFGS......RSDQY......
116061143    ......QTMSM......LVKYH......WVVMP......NIHRD......ADFGV......HADIW......
116061740    ......GILAQ......VTKYL......AIVME......KIHRD......ADFGV......KADIW......
```

```
119114174     ......MVLSQ.....VTKYF.....WIIME.....KLHRD......ADFGV......KADIW......
118404452     ......NMLKK.....IATYY.....WLVME.....VIHRD......VDFGV......RSDIW......
118404384     ......KFLQK.....TIEYK.....WLVME.....MIHRD......GDFG-......KVDVW......
169855369     ......LVMKE.....IVNFL.....WVVME.....IIHRD......TDFGF......KVDIW......
169849179     ......LVMRS.....IVNYI.....WVVME.....VIHRD......TDFGF......KVDIW......
169847639     ......ALLTQ.....ITKYF.....WIVME.....VIHRD......CDFGV......KADIW......
169854982     ......GLMKG.....VIGLD.....WIRME.....IAHRD......TDFAN......KVDVW......
169843361     ......TLMSL.....VLRVR.....YIALR.....FIHRD......GDLGV......SADIW......
118601812     ......SIMQQ.....VVKYY.....WIVME.....KIHRD......ADFGV......VADIW......
147905953     ......QAMSQ.....IVSYY.....WLVMK.....QIHRD......ADFGV......KADIW......
147904114     ......VIMRD.....VVEMY.....WVIME.....VIHRD......SDFGF......EVDIW......
147901101     ......SIMQQ.....VVKYY.....WIVME.....KIHRD......ADFGV......VADIW......
117644566     ......NLLRK.....IVSFY.....WMVME.....VIHRD......VDFGV......RSDVW......
117645510     ......NLLRK.....IVSFY.....WMVME.....VIHRD......VDFGV......RSDVW......
117928194     ......KAAAA.....VVAVF.....YLVME.....LIHRD......ADFGL......RSDVY......
117616416     ......VIMRD.....VVEMY.....WVLME.....VIHRD......SDFGF......EVDIW......
117616478     ......EILAT.....IVKLL.....WIMIE.....IIHRD......ADFGV......KADIW......
117616422     ......LMLKT.....IVAYH.....WICME.....KIHRD......ADFGI......LCDIW......
117616550     ......NMLKK.....IATYY.....WLVME.....VIHRD......VDFGV......RSDLW......
156743320     ......KAVTA.....IVPLY.....FMVMR.....SIHRD......ADFGV......LMDVY......
118084302     ......NMLKK.....IATYY.....WLVME.....VIHRD......VDFGV......RSDLW......
118085268     ......LVMRE.....IVNYL.....WVVME.....VIHRD......TDFGF......KVDIW......
118085670     ......NILKL.....VVKFY.....WLVLE.....TIHRD......VDFGV......RCDAW......
118087722     ......VIMRD.....VVDMY.....WVVME.....VIHRD......SDFGF......EVDIW......
118089619     ......LVMRE.....IVNYL.....WVVME.....VIHRD......TDFGF......KVDIW......
118091019     ......VIMRD.....VVEMY.....WVLME.....VIHRD......SDFGF......EVDIW......
118092341     ......YMVKE.....IVAYF.....WICME.....KMHRD......ADFGV......LCDIW......
118092958     ......DILAS.....IVKLL.....WILIE.....IIHRD......ADFGV......KADIW......
118092960     ......DILAS.....IVKLL.....WILIE.....IIHRD......ADFGV......KADIW......
118093669     ......QAMSQ.....VVTYY.....WLVMK.....QIHRD......ADFGV......KADMW......
118093591     ......NILQS.....VVRFY.....WLVLE.....IIHRD......VDFGV......RCDVW......
118094908     ......LVMKE.....IVNFL.....FVVME.....VIHRD......TDFGF......KVDIW......
118094906     ......LVMKE.....IVNFL.....FVVME.....VIHRD......TDFGF......KVDIW......
118095294     ......NMLKK.....IATYY.....WLVME.....VIHRD......VDFGV......KSDLW......
118097318     ......EILAT.....IVKLL.....WIMIE.....IIHRD......ADFGV......KADIW......
118100308     ......KFLQR.....SIEYK.....WLVME.....MIHRD......ADFG-......KVDVW......
118118945     ......VIMRD.....VVEMY.....WVVME.....VIHRD......SDFGF......EVDIW......
118138635     ......DILAS.....IVKLL.....WILIE.....IIHRD......ADFGV......KADVW......
118142813     ......RFLQK.....TIQYR.....WLVME.....MIHRD......GDFG-......KVDVW......
118466868     ......RAVAR.....LVAVY.....FLVME.....LVHRD......ADFGV......RSDVY......
118462529     ......DLAAT.....IVGVH.....WISMD.....LLHRD......ADFGV......RADQY......
118468993     ......DMAAT.....IVGVH.....WISMD.....LLHRD......TDFGI......RADQY......
118472179     ......RAVAR.....LVAVY.....FLVME.....LVHRD......ADFGL......RSDVY......
118467944     ......EFACR.....VVAMY.....WLAME.....IVHSD......IDFGV......ATDQY......
118619584     ......DLAAT.....IVGVH.....WISMD.....LLHRD......ADFGI......RADQY......
118616323     ......DVAAA.....IVRVN.....WISMD.....LLHRD......GDFGI......RADQY......
118618813     ......RTVAR.....LVAVY.....FLVME.....LVHRD......ADFGV......RSDVY......
118617740     ......ELAAT.....IVGVH.....WISMD.....LLHRD......TDFGI......RADQY......
90080966      ......VIMRD.....VVDMY.....WVVME.....VIHRD......SDFGF......EVDIW......
90085313      ......LVMRE.....IVNYL.....WVVME.....VIHRD......TDFGF......KVDIW......
119390433     ......EILAT.....IVKLL.....WIMIE.....IIHRD......ADFGV......KADIW......
121710228     ......LVMKD.....IVNFL.....WVVME.....VIHRD......TDFGF......KVDIW......
121712622     ......IVMKD.....IVNFL.....WVVME.....IIHRD......TDFGF......KVDCW......
121714238     ......T---P.....IEEFR.....-LILR.....FVHTD......ADFGS......ATDIW......
121711315     ......AILSE.....VTKYH.....WIIME.....KLHRD......ADFGV......KADIW......
121716369     ......KVMKQ.....INELI.....WLVCE.....IIHRD......CDFGV......EIDVW......
121719196     ......SVLAT.....VTQYK.....WIVME.....KIHRD......ADFGV......KADIW......
119482654     ......AILSE.....VTKYH.....WIVME.....KLHRD......ADFGV......KADIW......
119481593     ......KVMKQ.....INELI.....WLVCE.....IIHRD......CDFGV......EVDVW......
119480119     ......LVMKD.....IVNFL.....WVVME.....VIHRD......TDFGF......KVDIW......
119498577     ......IVMKD.....IVNFL.....WVVME.....IIHRD......TDFGF......KVDCW......
119499105     ......SVLAT.....VTQYK.....WIVME.....KIHRD......ADFGV......KADIW......
119595434     ......LVMRE.....IVNYL.....WVVME.....VIHRD......TDFGF......KVDIW......
119584928     ......QAMSQ.....IVSYY.....WLVMK.....QIHRD......ADFGV......KADIW......
119574019     ......LVMKE.....IVNFL.....FVVME.....VIHRD......TDFGF......KVDIW......
119577266     ......VIMRD.....VVEMY.....WVVME.....VIHRD......SDFGF......EVDIW......
119586114     ......FMVKE.....IVAYF.....WICME.....KMHRD......ADFGV......LCDIW......
119586116     ......FMVKE.....IVAYF.....WICME.....KMHRD......ADFGV......LCDIW......
119586115     ......FMVKE.....IVAYF.....WICME.....KMHRD......ADFGV......LCDIW......
119598893     ......NMLKK.....IATYY.....WLVME.....VIHRD......VDFGV......KSDLW......
119598896     ......NMLKK.....IATYY.....WLVME.....VIHRD......VDFGV......KSDLW......
119598897     ......NMLKK.....IATYY.....WLVME.....VIHRD......VDFGV......KSDLW......
119598894     ......NMLKK.....IATYY.....WLVME.....VIHRD......VDFGV......KSDLW......
```

```
119598892    ......NMLKK......IATYY......WLVME......VIHRD......VDFGV......KSDLW......
159036455    ......RAIAR......VVRIF......WIVME......VMHRD......TDFGL......EADLW......
159036432    ......RAAAA......VVQVY......WIVME......VLHRD......TDFGV......PSDLF......
119884864    ......NMLKK......IATYY......WLVME......VIHRD......VDFGV......KSDLW......
119887533    ......NILQF......VVKFY......WLVLE......IIHRD......VDFGV......RCDVW......
119606511    ......NILKL......VVRFY......WLVLE......TIHRD......VDFGV......RCDTW......
119622213    ......NMLKK......IATYY......WLVME......VIHRD......VDFGV......RSDLW......
119622210    ......NMLKK......IATYY......WLVME......VIHRD......VDFGV......RSDLW......
119622215    ......NMLKK......IATYY......WLVME......VIHRD......VDFGV......RSDLW......
119622211    ......NMLKK......IATYY......WLVME......VIHRD......VDFGV......RSDLW......
119623154    ......NLLRK......IVSFY......WMVME......VIHRD......VDFGV......RSDVW......
119622212    ......NMLKK......IATYY......WLVME......VIHRD......VDFGV......RSDLW......
119631649    ......NILQF......VVKFY......WLVLE......IIHRD......VDFGV......RCDVW......
119631650    ......NILQF......VVKFY......WLVLE......IIHRD......VDFGV......RCDVW......
119631648    ......NILQF......VVKFY......WLVLE......IIHRD......VDFGV......RCDVW......
119631651    ......NILQF......VVKFY......WLVLE......IIHRD......VDFGV......RCDVW......
119903370    ......IMMKD......IVAYF......WICME......KMHRD......ADFGV......LCDLW......
119902422    ......FMVKE......IVAYF......WICME......KMHRD......ADFGV......LCDIW......
119901473    ......LVMKE......IVNFL......FVVME......VIHRD......TDFGF......KVDIW......
119903372    ......IMMKD......IVAYF......WICME......KMHRD......ADFGV......LCDLW......
119905034    ......VIMRD......VVDMY......WVVME......VIHRD......SDFGF......EVDIW......
119906735    ......SIMQQ......VVKYY......WIVME......KIHRD......ADFGV......VADIW......
119905232    ......SILKL......VVRFY......WLVLE......TIHRD......VDFGV......RCDTW......
119905036    ......VIMRD......VVDMY......WVVME......VIHRD......SDFGF......EVDIW......
119909323    ......KFLQQ......TIEYK......WLVME......LIHRD......ADFG-......KVDIW......
119909321    ......KFLQQ......TIEYK......WLVME......LIHRD......ADFG-......KVDIW......
119919305    ......TILRE......VVAYI......WICME......KIHRD......ADFGV......LCDVW......
119919662    ......TVLSQ......VTKYY......WIIME......KIHRD......ADFGV......KADIW......
119916915    ......RFLQK......TIQYR......WLVME......MIHRD......GDFG-......KVDVW......
119912956    ......EILAT......IVKLL......WIMIE......IIHRD......ADFGV......KADIW......
119917718    ......DILAS......IVKLL......WILIE......IIHRD......ADFGV......KADVW......
119917716    ......DILAS......IVKLL......WILIE......IIHRD......ADFGV......KADVW......
119920101    ......LVMRE......IVNYL......WVVME......VIHRD......TDFGF......KVDIW......
119920099    ......LVMRE......IVNYL......WVVME......VIHRD......TDFGF......KVDIW......
119920019    ......NLLKK......IVSFY......WMVME......VIHRD......VDFGV......RSDVW......
120404511    ......RAVAR......LVAVY......FLVME......LVHRD......ADFGL......RGDVY......
123403534    ......DLLKK......TVQYF......MILME......VLHRD......TDFGI......PADIW......
123416811    ......TLLKR......IVPYL......HIVME......IIHRD......ADFGL......KCDIW......
123416209    ......RLMST......IIEFY......WILME......KIHRD......ADFGY......SVDIW......
123431208    ......NNVID......VVNYY......YLEME......RIHRD......CDFGV......KVDIW......
123438906    ......AFWST......VVKYH......YIVME......QIHRD......GDFGI......KADIW......
123447684    ......DLMAP......VVKYI......YIVME......IIHRD......SDFGI......KCDIW......
123454568    ......SFWSS......VVGYY......YILME......QIHRD......GDFGV......KADIW......
123454836    ......DMLRV......VVSCE......WIVME......KIHRD......ADFGV......KCDIW......
123977044    ......RLMAT......IIKFY......WILME......KIHRD......ADFGY......GVDIW......
123464257    ......ELIKK......TIQFY......MLLME......KIHRD......TDFGL......PADVW......
123468059    ......DLLRK......TVQYY......MILLE......VLHRD......TDFGI......PADIW......
123501763    ......SYWSS......IISYY......YILMS......QIHRD......GDFGV......KADIW......
123500044    ......SFWAT......LIQYY......WFLSE......QIHRD......GSLGC......KADIW......
123479975    ......AFWAT......QIQYY......WFLYE......QIHRD......GAIGS......KADIW......
123504925    ......RLMYS......IVKFY......WIIME......KIHRD......ADFGY......AVDIW......
123485137    ......DMLRA......VIRYE......WIVME......KVHRD......ADFGV......KCDLW......
154420412    ......RLMYT......IIRFY......WIIME......KIHRD......ADFGY......AVDIW......
154415027    ......KILRT......IIKYY......MLLME......IIHRD......CDFGA......ASDVW......
154414954    ......NNMCS......IVKIY......YIILQ......IIHRD......GDFGS......KADIW......
122890900    ......NMLKK......IATYY......WLVME......VIHRD......VDFGV......RSDIW......
123121730    ......TVLSQ......VTKYY......WIIME......KIHRD......ADFGV......KADIW......
123232294    ......NILQF......VVKFY......WLVLE......IIHRD......VDFGV......RCDVW......
123997255    ......VIMRD......VVEMY......WVLME......VIHRD......SDFGF......EVDIW......
124360373    ......KTLSL......ILKAH......WVVMP......HLHRD......ADFGV......KADIW......
145479077    ......ALMKM......IVKYY......FMVVE......KIHRD......ADFGF......KVDIW......
145484284    ......ALMKM......IVKYY......FMVVE......KIHRD......ADFGF......KVDIW......
145487408    ......QILKQ......I----......WLVME......KIHRD......ADFGV......LTDIW......
145489275    ......QILRD......IVKYF......WLVME......KIHRD......ADFGV......KTDIW......
145496712    ......QILRD......IVKYF......WLVME......KIHRD......ADFGV......KTDIW......
145499916    ......KILKD......IVSFL......WLIME......KIHRD......ADFGV......LTDIW......
145546440    ......QILKQ......IVQYY......WLVME......KIHRD......ADFGV......LTDIW......
145551005    ......QILRD......IVKYF......WLIME......KIHRD......ADFGV......KTDIW......
125538326    ......KMMST......LLGAY......WIIMP......LVHRD......ADFGA......KADIW......
125532654    ......QIMSL......VIRAY......WVVMP......QIHRD......GDFGV......KADIW......
125545252    ......KAMAL......VLRAH......WVVMP......RIHRD......ADFGV......KADIW......
125542127    ......QTMSL......VIRAY......WVIMP......HIHRD......GDFGV......KKYIW......
```

125545761	......EMLQQ.....VVRYF.....WIVME.....KVHRD.....GDFGV.....KVDVW.....
125541427	......QTMIL.....VMKAH.....WVVMP.....HIHRD.....GDFGV.....-ADIW.....
125545687	......RMLGA.....IVEIY.....TIMME.....VIHRD.....SDFDN.....EVDIW.....
125558463	......SVLSQ.....ITDYY.....WIVME.....KIHRD.....ADFGV.....KADIW.....
125555378	......QTMSL.....LLRAY.....WVIMP.....HIHRD.....GDFGV.....KADIW.....
125575414	......QIMSL.....VIRAY.....WVVMP.....QIHRD.....GDFGV.....KADIW.....
125587886	......RMLGA.....IVEIY.....TIMME.....VIHRD.....SDFDN.....EVDIW.....
125587959	......EMLQQ.....VVRYF.....WIVME.....KVHRD.....GDFGV.....KVDVW.....
125581029	......KMMST.....LLGAY.....WIIMP.....LVHRD.....ADFGA.....KADIW.....
125584680	......QTMSL.....VIRAY.....WVIMP.....HIHRD.....GDFGV.....EQDII.....
125597270	......QTMSL.....LLRAY.....WVIMP.....HIHRD.....GDFGV.....KADIW.....
125600370	......SVLSQ.....ITDYY.....WIVME.....KIHRD.....ADFGV.....KADIW.....
125804475	......DILAS.....IVKLL.....WLLIE.....VIHRD.....ADFGV.....KADIW.....
148922935	......QAMSQ.....IVSYY.....WLVMK.....QIHRD.....ADFGV.....KADLW.....
125806466	......LVMKE.....IVNFL.....FVVME.....VIHRD.....TDFGF.....KVDIW.....
125812164	......KFLQK.....TIEYK.....WLVME.....MIHRD.....GDFG-.....KVDVW.....
125816359	......KFLEQ.....TIEYK.....WLVME.....MIHRD.....ADFG-.....KVDIW.....
125816538	......NMLKK.....IATYY.....WLVME.....VIHRD.....VDFGV.....RSDIW.....
125827340	......DILAA.....ILSLL.....WILLE.....IIHRD.....ADFGV.....KADIW.....
125830425	......KFLQK.....TVEYR.....WLVME.....MIHRD.....GDFG-.....KVDVW.....
125833244	......DILAS.....IVKLL.....WLLIE.....IIHRD.....ADFGV.....KADIW.....
125837988	......KFLQR.....SIEYK.....WLVME.....MIHRD.....ADFG-.....KVDIW.....
125847100	......VIMRD.....VVEMY.....WVIME.....VIHRD.....SDFGF.....EVDIW.....
125848922	......TVLSQ.....VTKYY.....WIIME.....KIHRD.....ADFGV.....KADIW.....
125950024	......KFLQR.....SIEYK.....WLVME.....MIHRD.....ADFG-.....KVDIW.....
126136577	......LVMKG.....IVNFI.....WVVME.....VIHRD.....TDFGF.....KVDIW.....
126290830	......EILAT.....IVKLL.....WIMIE.....IIHRD.....ADFGV.....KADIW.....
126290827	......EILAT.....IVKLL.....WIMIE.....IIHRD.....ADFGV.....KADIW.....
126302937	......SIMQQ.....VVKYY.....WIVME.....KIHRD.....ADFGV.....VADIW.....
126302572	......NILQF.....VVKFY.....WLVLE.....IIHRD.....VDFGV.....RCDVW.....
126304542	......IMMKD.....IVAYF.....WICME.....KMHRD.....ADFGV.....LCDLW.....
126304345	......VIMRD.....VVDMY.....WVVME.....VIHRD.....SDFGF.....EVDIW.....
126309281	......NMLKK.....IATYY.....WLVME.....VIHRD.....VDFGV.....RSDIW.....
126324704	......KFLRQ.....TIEYK.....WLVME.....LIHRD.....ADFG-.....KVDVW.....
126325799	......LVMKE.....IVNFL.....FVVME.....VIHRD.....TDFGF.....KVDIW.....
126325801	......LVMKE.....IVNFL.....FVVME.....VIHRD.....TDFGF.....KVDIW.....
126325797	......LVMKE.....IVNFL.....FVVME.....VIHRD.....TDFGF.....KVDIW.....
126326279	......QAMSQ.....VVTYY.....WLVMK.....QIHRD.....ADFGV.....KADMW.....
126326550	......NILQF.....VVKFY.....WLVLE.....IIHRD.....VDFGV.....RCDVW.....
126327740	......LVMRE.....IVNYL.....WVVME.....VIHRD.....TDFGF.....KVDIW.....
126327738	......LVMRE.....IVNYL.....WVVME.....VIHRD.....TDFGF.....KVDIW.....
126329193	......VIMRD.....VVQMY.....WVVME.....VIHRD.....SDFGF.....EVDIW.....
126329159	......FIVRN.....IVAYH.....WICME.....KIHRD.....ADFGI.....LCDIW.....
126335583	......RFLQK.....TIQYR.....WLVME.....MIHRD.....GDFG-.....KVDVW.....
126336695	......QAMSQ.....IVSYY.....WLVMR.....QIHRD.....ADFGV.....VSGQW.....
126337519	......TVLSQ.....VTKYY.....WIIME.....KIHRD.....ADFGV.....KADIW.....
126338365	......NMLKK.....IATYY.....WLVME.....VIHRD.....VDFGV.....KSDLW.....
126342397	......TVLSQ.....VTKYY.....WIIME.....KIHRD.....ADFGV.....KADIW.....
126342730	......LVMRE.....IVNYL.....WVVME.....VIHRD.....TDFGF.....KVDIW.....
160333867	......TVLSQ.....ITKYF.....WIIME.....KIHRD.....ADFGV.....KADIW.....
146322801	......LVMKD.....IVNFL.....WVVME.....VIHRD.....TDFGF.....KVDIW.....
132626321	......DILAS.....IVKLL.....WILIE.....IIHRD.....ADFGV.....KADVW.....
134102978	......RAIAV.....VVTLY.....FVVME.....IVHRD.....SDFGI.....SADLW.....
145233655	......SVLAT.....VTQYK.....WIVME.....KIHRD.....ADFGV.....KADIW.....
145232489	......TVMKQ.....INEII.....WLVCE.....VIHRD.....CDFGV.....EIDVW.....
154334775	......EILQE.....IVRFY.....WIVME.....VIHRD.....ADFGV.....RADIW.....
154337364	......EIMER.....IVAYI.....RIVME.....IVHGD.....ADFGI.....ACDIW.....
154337020	......DALHS.....VVKYV.....FLVEE.....VVHGD.....NDFAL.....ASDIW.....
146078073	......EIMER.....IVAYI.....RIVME.....IVHGD.....ADFGI.....ACDIW.....
146085731	......DALHT.....VVKYL.....FLVEE.....VVHGD.....TDFAL.....ASDIW.....
146084886	......DASRN.....LAEYL.....WLLSN.....MVHAD.....GDLES.....--DIW.....
146082467	......EILQE.....IVRFY.....WIVME.....VIHRD.....ADFGV.....RADMW.....
145243830	......LVMKD.....IVNFL.....WVVME.....VIHRD.....TDFGF.....KVDIW.....
145247963	......AILSE.....VTRYH.....WIIME.....KLHRD.....ADFGV.....KADIW.....
145253831	......IVMKD.....IVNFL.....WVVME.....IIHRD.....TDFGF.....KVDCW.....
134093062	......SIMQQ.....VVKYY.....WIVME.....KIHRD.....ADFGV.....VADIW.....
148877259	......SIMQQ.....VVKYY.....WIVME.....KIHRD.....ADFGV.....VADIW.....
148877255	......SIMQQ.....VVKYY.....WIVME.....KIHRD.....ADFGV.....VADIW.....
134093126	......SIMQQ.....VVKYY.....WIVME.....KIHRD.....ADFGV.....VADIW.....
148877253	......SIMQQ.....VVKYY.....WIVME.....KIHRD.....ADFGV.....VADIW.....
134093087	......SIMQQ.....VVKYY.....WIVME.....KIHRD.....ADFGV.....VADIW.....

```
148877258 ......SIMQQ.....VVKYY.....WIVME.....KIHRD.....ADFGV.....VADIW.....
148877254 ......SIMQQ.....VVKYY.....WIVME.....KIHRD.....ADFGV.....VADIW.....
148877256 ......SIMQQ.....VVKYY.....WIVME.....KIHRD.....ADFGV.....VADIW.....
148877257 ......SIMQQ.....VVKYY.....WIVME.....KIHRD.....ADFGV.....VADIW.....
134093114 ......SIMQQ.....VVKYY.....WIVME.....KIHRD.....ADFGV.....VADIW.....
134104915 ......DILAS.....IVKLL.....WILIE.....IIHRD.....ADFGV.....KADVW.....
134104916 ......DILAS.....IVKLL.....WILIE.....IIHRD.....ADFGV.....KADVW.....
145342059 ......STLAQ.....VTKYL.....AIVME.....KIHRD.....ADFGV.....KADIW.....
145347880 ......ATLRA.....VPEYV.....VLVQR.....VVHRD.....VDFGG.....KSDQY.....
145353332 ......QTMSM.....LVKYY.....WVVMP.....NIHRD.....ADFGV.....HADIW.....
145601265 ......AILSE.....VTKYY.....WIVME.....KLHRD.....ADFGV.....KADIW.....
145607087 ......SVLST.....VTH--......----......KIHRD.....ADFGV.....KADIW.....
145223559 ......RAVAR.....LVAVY.....FLVME.....LVHRD.....ADFGL.....RGDVY.....
145279211 ......NILRT.....VVRFY.....WLVLE.....TIHRD.....VDFGV.....RCDTW.....
145279237 ......NMLKK.....IATYY.....WLVME.....VIHRD.....VDFGV.....RSDLW.....
145324923 ......SVLSQ.....ITEYY.....WIIME.....KIHRD.....ADFGV.....KADIW.....
145337763 ......QTMML.....VLKSH.....WVIMP.....HIHRD.....GDFGV.....KADIW.....
145340080 ......QTMTL.....VIKSF.....WVVMP.....HIHRD.....GDFGV.....KADIW.....
145580573 ......DILAS.....IVKLL.....WILIE.....IIHRD.....ADFGV.....KADVW.....
146184686 ......RMLKE.....VVKYY.....WLIME.....IIHRD.....ADFGV.....KADIW.....
146180978 ......SILQN.....IVGYF.....WLVTE.....KIHRD.....ADFGV.....KTDIW.....
146231908 ......TVLSQ.....VTKYY.....WIIME.....KIHRD.....ADFGV.....KADIW.....
146420305 ......LVMKD.....IVNFL.....WVIME.....IIHRD.....TDFGF.....KVDVW.....
146423433 ......QFLSE.....VTHYY.....WIIMD.....VIHRD.....CDFGV.....KADIW.....
146417867 ......QIISE.....ITSFY.....WVVME.....KIHRD.....TDFGV.....KADLW.....
146418255 ......LVMKG.....IVNFI.....WVVME.....VIHRD.....TDFGF.....KVDIW.....
146416649 ......QFLSL.....IVQYL.....WIIME.....TVHRD.....ADFGV.....KADIW.....
149248184 ......QFLSE.....ITHYY.....WIVMD.....VIHRD.....CDFGV.....KADIW.....
149246880 ......LVMKD.....IVNFL.....WVIME.....IIHRD.....TDFGF.....KVDVW.....
149244382 ......QFLSK.....IIQYI.....FIVMD.....VVHRD.....ADFGV.....KADIW.....
149236173 ......QIISE.....ITSYY.....WVVME.....KIHRD.....TDFGV.....KADVW.....
149238001 ......LVMKG.....IVNYI.....WVIME.....VIHRD.....TDFGF.....KVDIW.....
148273054 ......RSAAR.....VVNVF.....YLVME.....IVHRD.....GDFGL.....RSDIY.....
147859270 ......QTMSL.....LLQAH.....WVVMP.....HIHRD.....ADFGV.....KADIW.....
109745521 ......LVMKE.....IVNFL.....FVVME.....VIHRD.....TDFGF.....KVDIW.....
148676188 ......NILRT.....VVRFY.....WLVLE.....TIHRD.....VDFGV.....RCDTW.....
148676187 ......NILRT.....VVRFY.....WLVLE.....TIHRD.....VDFGV.....RCDTW.....
148668278 ......TVLSQ.....VTKYY.....WIIME.....KIHRD.....ADFGV.....KADIW.....
148685522 ......RFLQK.....TIQYR.....WLVME.....MIHRD.....GDFG-.....KVDVW.....
148677282 ......QAMSQ.....IVSYY.....WLVMK.....QIHRD.....ADFGV.....KADIW.....
148682620 ......NMLKK.....IATYY.....WLVME.....VIHRD.....VDFGV.....RSDLW.....
148685525 ......RFLQK.....TIQYR.....WLVME.....MIHRD.....GDFG-.....KVDVW.....
148680633 ......NMLKK.....IATYY.....WLVME.....VIHRD.....VDFGV.....RSDIW.....
148682621 ......NMLKK.....IATYY.....WLVME.....VIHRD.....VDFGV.....RSDLW.....
148684378 ......LVMRE.....IVNYL.....WVVME.....VIHRD.....TDFGF.....KVDIW.....
148695071 ......QAMSQ.....VVTYY.....WLVMK.....QIHRD.....ADFGV.....KADMW.....
148691804 ......EILAT.....IVKLL.....WIMIE.....IIHRD.....ADFGV.....KADIW.....
148695072 ......QAMSQ.....VVTYY.....WLVMK.....QIHRD.....ADFGV.....KADMW.....
148692147 ......LMLKT.....IVAYH.....WICME.....KIHRD.....ADFGI.....LCDIW.....
148691955 ......NLLRK.....IVTFY.....WMVME.....VIHRD.....VDFGV.....RSDVW.....
148702990 ......NMLKK.....IATYY.....WLVME.....VIHRD.....VDFGV.....KSDLW.....
148697152 ......TVLSQ.....VTKYY.....WIIME.....KIHRD.....ADFGV.....-ADIW.....
148704646 ......FMVKE.....IVAYF.....WICME.....KMHRD.....ADFGV.....LCDIW.....
148702993 ......NMLKK.....IATYY.....WLVME.....VIHRD.....VDFGV.....KSDLW.....
148706583 ......IMMKD.....IVAYF.....WICME.....KMHRD.....ADFGV.....LCDLW.....
148710091 ......DILAS.....IVKLL.....WILIE.....IIHRD.....ADFGV.....KADVW.....
148710090 ......DILAS.....IVKLL.....WILIE.....IIHRD.....ADFGV.....KADVW.....
148702992 ......NMLKK.....IATYY.....WLVME.....VIHRD.....VDFGV.....KSDLW.....
148704648 ......FMVKE.....IVAYF.....WICME.....KMHRD.....ADFGV.....LCDIW.....
148724961 ......VIMRD.....VVEMY.....WVIME.....VIHRD.....SDFGF.....EVDIW.....
148726518 ......LVMKE.....IVNFL.....FVVME.....VIHRD.....TDFGF.....KVDIW.....
148745103 ......RFLQK.....TIQYR.....WLVME.....MIHRD.....GDFG-.....KVDVW.....
156093838 ......HFLQI.....IVKYH.....VIVCE.....LVHRD.....VDFGS.....SADIW.....
150247103 ......NMLKK.....IATYY.....WLVME.....VIHRD.....VDFGV.....RSDIW.....
148887437 ......LVMKD.....IVNFI.....WVVME.....VIHRD.....TDFGF.....KVDIW.....
148921436 ......RFLQK.....TIQYR.....WLVME.....MIHRD.....GDFG-.....KVDVW.....
149033203 ......NLLRK.....IVTFY.....WMVME.....VIHRD.....VDFGV.....RSDVW.....
149028601 ......NILRT.....VVRFY.....WLVLE.....TIHRD.....VDFGV.....RCDTW.....
149040344 ......DILAS.....IVKLL.....WILIE.....IIHRD.....ADFGV.....KADVW.....
149040345 ......DILAS.....IVKLL.....WILIE.....IIHRD.....ADFGV.....KADVW.....
149052243 ......EILAT.....IVKLL.....WIMIE.....IIHRD.....ADFGV.....KADIW.....
149053475 ......KFLQR.....SIEYK.....WLVME.....MIHRD.....ADFG-.....KVDVW.....
```

149051360	......FMVKE.....IVAYF.....WICME.....KMHRD.....ADFGV.....LCDIW.....
158749606	......NMLKK.....IATYY.....WLVME.....VIHRD.....VDFGV.....KSDLW.....
149056428	......LMLKS.....IVAYH.....WICME.....KIHRD.....ADFGI.....LCDIW.....
149053203	......NMLKK.....IATYY.....WLVME.....VIHRD.....VDFGV.....RSDIW.....
149052242	......EILAT.....IVKLL.....WIMIE.....IIHRD.....ADFGV.....KADIW.....
149066538	......SIMQQ.....VVKYY.....WIVME.....KIHRD.....ADFGV.....VADIW.....
149060125	......TVLSQ.....VTKYY.....WIIME.....KIHRD.....ADFGV.....-ADIW.....
149068911	......LVMRE.....IVNYL.....WVVME.....VIHRD.....TDFGF.....KVDIW.....
149067798	......RFLQK.....TIQYR.....WLVME.....MIHRD.....GDFG-.....KVDVW.....
149067799	......RFLQK.....TIQYR.....WLVME.....MIHRD.....GDFG-.....KVDVW.....
150865228	......KFLSE.....VTQYY.....WIIMD.....VIHRD.....CDFGV.....KADIW.....
150863909	......LVMKD.....IVNFL.....WVIME.....IIHRD.....TDFGF.....KVDVW.....
150866045	......QFLSR.....ITSYI.....FIVME.....KVHRD.....ADFGV.....KADIW.....
150865759	......QIISE.....ITQYF.....WVIME.....KIHRD.....TDFGV.....KADIW.....
149410489	......FMVKE.....IVSYS.....WICME.....KMHRD.....ADFGV.....LCDIW.....
149410969	......NMLKK.....IATYY.....WLVME.....VIHRD.....VDFGV.....RSDLW.....
149411461	......TVLSQ.....ITRYF.....WIIME.....KIHRD.....ADFGV.....KADIW.....
149411800	......TVLSQ.....VTKYY.....WIIME.....KIHRD.....ADFGV.....KADIW.....
149441288	......RFLQK.....TIQYR.....WLVME.....MIHRD.....GDFG-.....KVDVW.....
149451947	......SIMQQ.....VVKYY.....WIVME.....KIHRD.....ADFGV.....VADIW.....
149469310	......SIMQQ.....VVKYY.....WIVME.....KIHRD.....ADFGV.....VADIW.....
149536168	......LVMRE.....IVNYL.....WVVME.....VIHRD.....TDFGF.....KVDIW.....
149602583	......QAMSQ.....IVSYY.....WLVMR.....QIHRD.....ADFGV.....KADIW.....
149633183	......NMLKK.....IATYY.....WLVME.....VIHRD.....VDFGV.....RSDLW.....
149634716	......NILKL.....VVKFY.....WLVLE.....TIHRD.....VDFGV.....RCDAW.....
149639631	......QAMSQ.....VVTYY.....WLVMK.....QIHRD.....ADFGV.....KADMW.....
149640949	......VIMRD.....VVDMY.....WVVME.....VIHRD.....SDFGF.....EVDIW.....
149641760	......KFLQR.....SIEYK.....WLVME.....MIHRD.....ADFG-.....KVDVW.....
149689690	......DILAS.....IVKLL.....WILIE.....IIHRD.....ADFGV.....KADVW.....
149689692	......DILAS.....IVKLL.....WILIE.....IIHRD.....ADFGV.....KADVW.....
149692486	......VIMRD.....VVEMY.....WVLME.....VIHRD.....SDFGF.....EVDIW.....
149711760	......TVLSQ.....ITRYF.....WIIME.....KIHRD.....ADFGV.....KVDIW.....
149720561	......KFLRQ.....TIEYK.....WLVME.....LIHRD.....ADFG-.....KVDIW.....
149720559	......KFLRQ.....TIEYK.....WLVME.....LIHRD.....ADFG-.....KVDIW.....
149720557	......KFLRQ.....TIEYK.....WLVME.....LIHRD.....ADFG-.....KVDIW.....
149721703	......SIMQQ.....VVKYY.....WIVME.....KIHRD.....ADFGV.....VADIW.....
149725860	......RFLQK.....TIQYR.....WLVME.....MIHRD.....GDFG-.....KVDVW.....
149727280	......NMLKK.....IATYY.....WLVME.....VIHRD.....VDFGV.....RSDLW.....
149729620	......QAMSQ.....IVSYY.....WLVMK.....QIHRD.....ADFGV.....KADIW.....
149730901	......NILQF.....VVKFY.....WLVLE.....IIHRD.....VDFGV.....RCDVW.....
149730887	......QAMSQ.....VVTYY.....WLVMK.....QIHRD.....ADFGV.....KADMW.....
149731245	......LVMKE.....IVNFL.....FVVME.....VIHRD.....TDFGF.....KVDIW.....
149731098	......NMLKK.....IATYY.....WLVME.....VIHRD.....VDFGV.....KSDLW.....
149731247	......LVMKE.....IVNFL.....FVVME.....VIHRD.....TDFGF.....KVDIW.....
149733075	......VIMRD.....VVDMY.....WVVME.....VIHRD.....SDFGF.....EVDIW.....
149733680	......SIMQQ.....VVKYY.....WIVME.....KIHRD.....ADFGV.....VADIW.....
149743507	......NILKL.....VVKFY.....WLVLE.....TIHRD.....VDFGV.....RCDTW.....
149744936	......LVMRE.....IVNYL.....WVVME.....VIHRD.....TDFGF.....KVDIW.....
149745593	......TVLSQ.....VTKYY.....WIIME.....KIHRD.....ADFGV.....KADIW.....
149922712	......RLCGT.....LVRTL.....WFAMD.....FVHCD.....IDFGV.....RSDLY.....
149924825	......RALAA.....VVRIY.....WIVME.....LAHRD.....VDFGL.....RSDQF.....
149924562	......RALAA.....VVRIY.....WIVME.....LVHRD.....VDFGL.....RSDQF.....
149923389	......WITGR.....IIPVY.....-IVLK.....ILHLD.....ADWGV.....RTDVY.....
154284105	......IVMKG.....IVNYL.....WVVME.....IIHRD.....TDFGF.....RVDIW.....
154285018	......AVLAT.....VTQYK.....WIVME.....KIHRD.....ADFGV.....KADIW.....
154270420	......LVMKD.....VVNFM.....WVIME.....VIHRD.....TDFGF.....KVDIW.....
154279930	......KIMQQ.....VNMII.....WLICE.....IIHRD.....CDFGV.....EIDVW.....
154314114	......LVMKD.....IVNFI.....WVIME.....VIHRD.....TDFGF.....KVDIW.....
154311331	......MVMKD.....IVNFL.....WVVME.....IIHRD.....TDFGF.....KVDIW.....
154319089	......SVLST.....VTQYK.....WIVME.....KIHRD.....ADFGV.....KADIW.....
151554011	......LILKT.....IVAYH.....WICME.....KIHRD.....ADFGI.....LCDIW.....
108743310	......SILRR.....VIKCH.....GILME.....IIHRD.....ADFGV.....NADIW.....
157133330	......KVLHD.....IVGFY.....SICME.....IMHRD.....CDFGV.....QSDIW.....
157136077	......DVLKS.....IVKCL.....WICME.....VIHRD.....CDFGI.....RADVW.....
157167917	......EVMKS.....IVTFY.....WICME.....IIHRD.....CDFGI.....RSDVW.....
157120754	......DIMRA.....TVHFY.....WICME.....VIHRD.....CDFGI.....KSDVW.....
109081614	......EILYK.....IIGFY.....SICTE.....ILHRD.....CDFGV.....HSDVW.....
109081572	......QVLHE.....IVGFY.....SICME.....IMHRD.....CDFGV.....QSDIW.....
109081612	......EILYK.....IIGFY.....SICTE.....ILHRD.....CDFGV.....HSDVW.....
109081610	......EILYK.....IIGFY.....SICTE.....ILHRD.....CDFGV.....HSDVW.....
109113360	......DVMRS.....IVQFY.....WICME.....IIHRD.....CDFGI.....RSDVW.....

109122953	......QVLHE......IVGFY......SICME......IMHRD......CDFGV......QSDIW......
109659371	......QVLHE......IVGFY......SICME......IMHRD......CDFGV......QSDIW......
109727200	......KINQA......IVLCH......YLVLE......VIHRD......TDFGV......KSDIW......
110083393	......EILRD......VVRCH......QVLLE......IVHRD......ADFGV......AGDIW......
110763741	......EVVMK......IVQFY......WICME......IIHRD......CDFGI......RSDVW......
169617479	......SFNKN......ICKYY......SIAME......IIHRD......CDFGV......TSDVW......
110809643	......QVLHT......MVKVY......DIVME......YIHQD......SDFGL......PADIW......
110809639	......ETLHA......LVSFY......FIAME......LIHRD......SDFGV......GADIW......
111380703	......QVGHD......IVTFY......VLCME......IMHRD......CDFGV......RSDVW......
115449123	......AILRT......VVRCH......QILLE......IVHRD......ADFGV......AGDIW......
115466858	......AILRT......VVRCH......QILLE......IVHRD......ADFGV......AGDIW......
115402919	......QVGHD......IVTFY......VLCME......IMHRD......CDFGV......RSDVW......
115397547	......EILHR......IIDFY......YICVE......IIHRD......CDFGV......QSDIW......
115389428	......NFNKD......ICRYY......SIAME......IIHRD......CDFGV......TSDVW......
114657799	......EILYK......IIGFY......SICTE......ILHRD......CDFGV......HSDVW......
114657805	......EILYK......IIGFY......SICTE......ILHRD......CDFGV......HSDVW......
114657803	......EILYK......IIGFY......SICTE......ILHRD......CDFGV......HSDVW......
114668626	......DIMRT......TVTFY......WICME......VIHRD......CDFGI......KSDVW......
114669021	......DVMRS......IVQFY......WICME......IIHRD......CDFGI......RSDVW......
114669023	......DVMRS......IVQFY......WICME......IIHRD......CDFGI......RSDVW......
114674708	......QVLHE......IVGFY......SICME......IMHRD......CDFGV......QSDIW......
114675073	......DVLKS......IVQCF......FIAME......VIHRD......CDFGI......RADVW......
115299610	......KISLS......VVACC......SIVLE......IIHRD......SDFGV......MSDIW......
115299612	......KISLS......VVACC......SIVLE......IIHRD......SDFGV......MSDIW......
115345159	......QVLHE......IVGFY......SICME......IMHRD......CDFGV......QSDVW......
115374939	......KLTVR......IVRTY......LMVQE......IVHRD......TDFGV......RSDLY......
115720219	......-AMRS......TVEFY......WICME......VIHRD......CDFGI......KSDVW......
115738296	......KVLHE......IVGFF......CICME......IMHRD......CDFGV......QSDIW......
115770468	......DVVKS......IIKFF......WICME......IIHRD......CDFGI......RSDVW......
116055435	......SRRKS......IVRCL......NFALE......VVHRD......CDFGL......KSDVW......
116057376	......RTLHK......IVRSS......SLVME......VVHRD......SDFGV......TADVW......
116487676	......DVMRS......IVQFY......WICME......IIHRD......CDFGI......RSDVW......
169857879	......HIMHD......IISFY......CICME......IIHRD......CDFGV......KSDVW......
169855605	......DIMHE......IISCF......CICME......IMHRD......CDFGV......KSDVW......
169847271	......TIMSS......IITFY......KIIME......TIHRD......CDFGV......RSDVW......
116830891	......EILRR......VVRCQ......SILME......IVHRD......ADFGV......AGDIW......
116830942	......DILKR......IIKCY......CFVME......IVHGD......ADFGA......AGDVW......
117616492	......DIMRT......TVTFY......WICME......VIHRD......CDFGI......KSDVW......
117616508	......DVLKS......IVQCF......FIAME......VIHRD......CDFGI......RADVW......
117616502	......DVLKS......IVQCF......FIAME......VIHRD......CDFGI......RADVW......
117616512	......DVLKS......IVQCF......FIAME......VIHRD......CDFGI......RADVW......
118099542	......DVMRS......IVQFY......WICME......IIHRD......CDFGI......RSDVW......
118425889	......DILHR......IVDFY......YICME......IIHRD......CDFGV......QSDIW......
118425887	......DILHR......IVDFY......YICME......IIHRD......CDFGV......QSDIW......
118425890	......DILHR......IVDFY......YICME......IIHRD......CDFGV......QSDIW......
90079635	......DIMRT......TVTFY......WICME......VIHRD......CDFGI......KSDIW......
90082457	......DVMRS......IVQFY......WICME......IIHRD......CDFGI......RSDVW......
121702619	......NFNKD......ICRYY......SIAME......IIHRD......CDFGV......TSDVW......
121700791	......DILHR......IIDFY......YMCVE......IIHRD......CDFGV......QSDIW......
121704694	......QVGHD......IVTFY......VLCME......IMHRD......CDFGV......RSDVW......
119494513	......DILHR......IIDFY......YMCVE......IIHRD......CDFGV......QSDIW......
119496629	......NFNKD......ICRYY......SIAME......IIHRD......CDFGV......TSDVW......
119493338	......QVGHD......IVTFY......VLCME......IMHRD......CDFGV......RSDVW......
119598172	......QVLHE......IVGFY......SICME......IMHRD......CDFGV......QSDIW......
119589371	......DVLKS......IVQCF......FIAME......VIHRD......CDFGI......RADVW......
119589369	......DVLKS......IVQCF......FIAME......VIHRD......CDFGI......RADVW......
119598174	......QVLHE......IVGFY......SICME......IMHRD......CDFGV......QSDIW......
119589669	......QVLHE......IVGFY......SICME......IMHRD......CDFGV......QSDIW......
119598173	......QVLHE......IVGFY......SICME......IMHRD......CDFGV......QSDIW......
119894881	......DVLKS......IVQCF......FIAME......VIHRD......CDFGI......RADVW......
119609490	......DIMRT......TVTFY......WICME......VIHRD......CDFGI......KSDIW......
119610379	......DVMRS......IVQFY......WICME......IIHRD......CDFGI......RSDVW......
119901930	......QVLHE......IVGFY......SICME......IMHRD......CDFGV......QSDIW......
149642561	......DVMRS......IVQFY......WICME......IIHRD......CDFGI......RSDVW......
123410068	......HSLRK......VVQLF......HILMD......ILHRD......ADFGL......KSDVW......
123447762	......HSLRI......VVHLF......HILMD......IIHRD......ADFGL......KSDVW......
123470200	......DCMNT......VLRLY......HIIMP......YIHRD......ADFGL......KSDVW......
123227426	......DVMRT......TVTFY......WICME......VIHRD......CDFGI......KSDIW......
145485879	......DALIS......IVQCY......CIWLE......IIHRD......ADFGI......DADIW......
145486658	......STLNG......LIKFY......RLVLE......HLHRD......TDFGI......ASDIW......

145500145	......KALID.....VVQCY.....YIVME.....IIHRD.....GDFGI.....DCDIW.....
145515786	......ETLVS.....IIRCY.....AIALE.....IIHRD.....SDFGV.....NTDVW.....
145515249	......ETLVS.....IIRCY.....AIALE.....IIHRD.....SDFGV.....NTDVW.....
145531239	......KALID.....-----.....YIVME.....IIHRD.....GDFGI.....DCDIW.....
145540585	......KLALE.....VVKCY.....HIILE.....IIHRD.....TDFGI.....DSDIW.....
145543927	......KLALE.....VVKCY.....HIILE.....IIHRD.....TDFGI.....DSDIW.....
145548828	......KILLH.....LVNLY.....KVVLE.....QIHRD.....TDFGI.....LSDIW.....
145473853	......STLNG.....LIKFY.....RLVLE.....HLHRD.....TDFGI.....ASDIW.....
125526807	......EALRR.....VVRCH.....AMLLE.....VVHLD.....GDFSV.....AADVW.....
125526139	......KINQA.....IVLCH.....YLVLE.....VIHRD.....TDFGV.....KSDIW.....
125541403	......AILRT.....VVRCH.....QILLE.....IVHRD.....ADFGV.....AGDIW.....
125542956	......EILRA.....VVRLH.....ALALE.....IVHRD.....ADFGA.....AADVW.....
125545497	......DVMRR.....VVRCH.....ALLLE.....VVHRD.....ADFGI.....AADVW.....
125545496	......DILSR.....VVRCH.....ALLLE.....VAHLD.....ADFGI.....AADVW.....
125555322	......RTL-C.....IVEFQ.....SIALE.....LVHRD.....TDFGV.....AADIW.....
125554372	......D----.....LVRCH.....IVLLE.....VVHGD.....AGFG-.....AGDIW.....
125597221	......RTL-C.....IVEFQ.....SIALE.....LVHRD.....TDFGV.....AADIW.....
125596323	......D----.....LVRCH.....IVLLE.....VVHGD.....AGFG-.....AGDIW.....
125663933	......RTL-C.....IVEFQ.....SIALE.....LVHRD.....TDFGV.....AADIW.....
126654562	......HSWRL.....LVQFL.....VVCME.....RIHRD.....SDFGI.....SSDIW.....
126277073	......QVLHE.....IVGFY.....SICME.....IMHRD.....CDFGV.....QSDIW.....
126277504	......EILYK.....IIGFY.....SICTE.....ILHRD.....CDFGV.....HSDVW.....
126309424	......DVMRS.....IVQFY.....WICME.....IIHRD.....CDFGI.....RSDVW.....
126308812	......DIMRT.....TVTFY.....WICME.....VIHRD.....CDFGI.....KSDIW.....
126323127	......QVLHE.....IVGFY.....SICME.....IMHRD.....CDFGV.....QSDIW.....
126323125	......QVLHE.....IVGFY.....SICME.....IMHRD.....CDFGV.....QSDIW.....
126323863	......DVLKS.....IVQCF.....FIAME.....VIHRD.....CDFGI.....RADVW.....
126334604	......DIMRT.....TVTFY.....WICME.....VIHRD.....CDFGI.....KSDVW.....
133778746	......DVMRS.....IVQFY.....WICME.....IIHRD.....CDFGI.....RSDVW.....
139949238	......DVMRT.....TVTFY.....WICME.....VIHRD.....CDFGI.....KSDVW.....
145230427	......EILHR.....IIDFY.....YICVE.....IIHRD.....CDFGV.....QSDVW.....
154332296	......QVLHT.....MVKVY.....DIVME.....YIHRD.....SDFGL.....PADIW.....
154341238	......QVMAR.....VMNNY.....VIPME.....VLHRD.....ADFGV.....SSDVW.....
154336411	......RNIFT.....TVNLY.....RLVME.....QIHRD.....ADFGI.....PSDIW.....
154338557	......RQVAA.....IVSSY.....YIVLE.....MIHRD.....SDFGV.....NSDIW.....
146088261	......RQVAA.....IVSSY.....YVVLE.....MIHRD.....SDFGV.....NSDIW.....
146093307	......QVMAR.....VMNNY.....VIPME.....VLHRD.....ADFGV.....SSDVW.....
146103249	......ETLHA.....LVGFY.....FIAME.....LIHRD.....SDFGV.....GADIW.....
146099095	......RNIFS.....TVNLY.....RLVME.....QIHRD.....ADFGI.....PSDIW.....
154345568	......EALHA.....LVHFY.....FIAME.....LIHRD.....SDFGV.....GADIW.....
146077285	......QVLHT.....MVKVY.....DIVME.....YIHQD.....SDFGL.....PADIW.....
145245517	......QVGHD.....IVTFY.....VLCME.....IMHRD.....CDFGV.....RSDVW.....
145255007	......NFNKD.....ICRYY.....SIAME.....IIHRD.....CDFGV.....TSDVW.....
145345653	......RTLHK.....IVRSL.....NLVME.....VVHRD.....SDFGV.....TADVW.....
145603860	......VILHE.....IIDFY.....YMCIE.....IIHRD.....CDFGV.....QSDIW.....
148222531	......DVMRS.....IVQFY.....WICME.....IIHRD.....CDFGI.....RSDVW.....
145617259	......KISLS.....VVACC.....SIVLE.....IIHRD.....SDFGV.....MSDIW.....
146164019	......IILQE.....FVFMY.....QIILE.....QVHRD.....TDFGV.....PSDIW.....
146423372	......QYNRL.....IVRYY.....GIAME.....IIHRD.....CDFGV.....TCDVW.....
146414928	......EILHK.....IVDFY.....YMCIE.....IIHRD.....CDFGV.....QLDVW.....
146416979	......KIMHE.....IIDFY.....VICME.....ILHRD.....CDFGV.....KSDVW.....
149248212	......EIARK.....IVKYY.....GITME.....IIHRD.....CDFGV.....SSDIW.....
149241038	......DILHK.....IVDFY.....YMCIE.....IIHRD.....CDFGV.....QSDVW.....
149239138	......RILHE.....IIEFY.....VICME.....IIHRD.....CDFGV.....KSDVW.....
147770515	......EILRF.....VVQCH.....AILME.....IIHRD.....ADFGV.....SGDIW.....
147852632	......KINQA.....IVVCY.....SLVLE.....VIHRD.....TDFGV.....SSDIW.....
148678460	......DVMRS.....IVQFY.....WICME.....IIHRD.....CDFGI.....RSDVW.....
148694090	......EILYK.....IIGFY.....SICTE.....ILHRD.....CDFGV.....HSDVW.....
148702439	......DVMRT.....TVTFY.....WICME.....VIHRD.....CDFGI.....KSDIW.....
148702438	......DVMRT.....TVTFY.....WICME.....VIHRD.....CDFGI.....KSDIW.....
148702437	......DVMRT.....TVTFY.....WICME.....VIHRD.....CDFGI.....KSDIW.....
148706743	......DIMRT.....TVTFY.....WICME.....VIHRD.....CDFGI.....KSDVW.....
148699513	......QVLHE.....IVGFY.....SICME.....IMHRD.....CDFGV.....QSDIW.....
148726469	......QVLHE.....IVGFY.....SICME.....IMHRD.....CDFGV.....QSDIW.....
149015605	......DVLKS.....IVQCF.....FIAME.....VIHRD.....CDFGI.....RADVW.....
149015610	......DVLKS.....IVQCF.....FIAME.....VIHRD.....CDFGI.....RADVW.....
149015607	......DVLKS.....IVQCF.....FIAME.....VIHRD.....CDFGI.....RADVW.....
149015609	......DVLKS.....IVQCF.....FIAME.....VIHRD.....CDFGI.....RADVW.....
149015611	......DVLKS.....IVQCF.....FIAME.....VIHRD.....CDFGI.....RADVW.....
149015606	......DVLKS.....IVQCF.....FIAME.....VIHRD.....CDFGI.....RADVW.....

```
149034454   .....QVLHE.....IVGFY.....SICME.....IMHRD.....CDFGV.....QSDIW.....
149054673   .....DVMRT.....TVTFY.....WICME.....VIHRD.....CDFGI.....KSDIW.....
149052856   .....DIMRT.....TVTFY.....WICME.....VIHRD.....CDFGI.....KSDVW.....
150864994   .....QYNRL.....IVQYY.....GIAME.....IIHRD.....CDFGV.....TSDVW.....
150866944   .....RILHE.....IIEFY.....VICME.....IIHRD.....CDFGV.....KSDVW.....
150864371   .....DILHK.....IVDFY.....YMCIE.....IIHRD.....CDFGV.....QSDIW.....
149409144   .....DIMRT.....TVTFY.....WICME.....VIHRD.....CDFGI.....KSDVW.....
149414677   .....EILYK.....IIGFY.....SICTE.....ILHRD.....CDFGV.....HSDVW.....
149635064   .....DIMRT.....TVTFY.....WICME.....VIHRD.....CDFGI.....KSSLE.....
149691834   .....EILYK.....IIGFY.....SICTE.....ILHRD.....CDFGV.....HSDVW.....
149692305   .....QVLHE.....IVGFY.....SICME.....IMHRD.....CDFGV.....QSDIW.....
149691832   .....EILYK.....IIGFY.....SICTE.....ILHRD.....CDFGV.....HSDVW.....
149723349   .....DIMRT.....TVTFY.....WICME.....VIHRD.....CDFGI.....KSDIW.....
149724977   .....DVMRS.....IVQFY.....WICME.....IIHRD.....CDFGI.....RSDVW.....
149758255   .....DVMRT.....TVTFY.....WICME.....VIHRD.....CDFGI.....KSDVW.....
149759460   .....QVLHE.....IVGFY.....SICME.....IMHRD.....CDFGV.....QSDIW.....
154281103   .....QVGHD.....IVTVY.....VLCME.....IMHRD.....CDFGV.....RSDVW.....
154282507   .....DILHR.....IIDFY.....YICVE.....IIHRD.....CDFGV.....QSDIW.....
154316783   .....QIMYD.....IVNFY.....IMCME.....IMHRD.....CDFGV.....KSDVW.....
154298932   .....GFNKG.....ICRYY.....SIAME.....IIHRD.....CDFGV.....TSDVW.....
154305637   .....DILHR.....IIDFY.....YICIE.....IIHRD.....CDFGV.....QSDIW.....
109003438   .....EVLCK.....IINLL.....YIAIE.....FIHRD.....ADFGL.....KSDVW.....
109111321   .....EVLCK.....IINLL.....YLAIE.....FIHRD.....ADFGL.....NSDVW.....
109111323   .....EVLCK.....IINLL.....YLAIE.....FIHRD.....ADFGL.....NSDVW.....
157787147   .....EVLCK.....IINLL.....YLAIE.....FIHRD.....ADFGL.....NSDVW.....
109477041   .....EVLCK.....IINLL.....YIAIE.....FIHRD.....ADFGL.....KSDVW.....
112180440   .....EVLCK.....IINLL.....YLAIE.....FIHRD.....ADFGL.....NSDVW.....
113912159   .....EVLCK.....IINLL.....YLAIE.....FIHRD.....ADFGL.....NSDVW.....
114555926   .....EVLCK.....IINLL.....YIAIE.....FIHRD.....ADFGL.....KSDVW.....
114555928   .....EVLCK.....IINLL.....YIAIE.....FIHRD.....ADFGL.....KSDVW.....
114555930   .....EVLCK.....IINLL.....YIAIE.....FIHRD.....ADFGL.....KSDVW.....
114555932   .....EVLCK.....IINLL.....YIAIE.....FIHRD.....ADFGL.....KSDVW.....
114623991   .....EVLCK.....IINLL.....YLAIE.....FIHRD.....ADFGL.....NSDTV.....
118094506   .....EVLCK.....IINLL.....YIAIE.....FIHRD.....ADFGL.....KSDVW.....
118104503   .....EVLCK.....IINLL.....YLAIE.....FIHRD.....ADFGL.....NSDVW.....
119627512   .....EVLCK.....IINLL.....YIAIE.....FIHRD.....ADFGL.....KSDVW.....
123982614   .....EVLCK.....IINLL.....YLAIE.....FIHRD.....ADFGL.....NSDVW.....
125826777   .....EVLCK.....IINLI.....YIAIE.....FIHRD.....ADFGL.....KSDVW.....
126334229   .....EVLCK.....IINLL.....YLAIE.....FIHRD.....ADFGL.....NSDVW.....
145580114   .....EVLCK.....IINLL.....YLAIE.....FIHRD.....ADFGL.....NSDVW.....
148698985   .....EVLCK.....IINLL.....YLAIE.....FIHRD.....ADFGL.....NSDVW.....
149637085   .....EVLCK.....IVNLL.....YIAIE.....FIHRD.....ADFGL.....KSDVW.....
149694543   .....EVLCK.....IINLL.....YIAIE.....FIHRD.....ADFGL.....KSDVW.....
149737053   .....EVLCK.....IINLL.....YLAIE.....FIHRD.....ADFGL.....NSDVW.....
157169517   .....GVMTT.....LVKFI.....YLVTE.....VIHRD.....SDFGL.....KTDMW.....
109081911   .....SVMTQ.....LVQLL.....YIVTE.....FVHRD.....SDFGL.....KSDVW.....
112419735   .....DTMTR.....LVQLI.....MIVSE.....FVHRD.....ADFGL.....KSDVW.....
114658101   .....SVMTQ.....LVQLL.....YIVTE.....FVHRD.....SDFGL.....KSDVW.....
115698861   .....SVMTT.....LVQLL.....YIVLE.....FVHRD.....SDFGL.....MSDVW.....
21450842f   .....AVMTK.....LVRLL.....YIVME.....LVHRD.....SDFGL.....KSDVW.....
117616330   .....SVMTQ.....LVQLL.....YIVTE.....FVHRD.....SDFGL.....KSDVW.....
118103089   .....AAMTK.....LVRLL.....YIVME.....LVHRD.....SDFGL.....KSDVW.....
119589690   .....AVMTK.....LVRLL.....YIVME.....LVHRD.....SDFGL.....KSDVW.....
119589693   .....AVMTK.....LVRLL.....YIVME.....LVHRD.....SDFGL.....KSDVW.....
126272256   .....SVMTQ.....LVQLL.....YIVTE.....FVHRD.....SDFGL.....KSDVW.....
148693971   .....SVMTQ.....LVQLL.....YIVTE.....FVHRD.....SDFGL.....KSDVW.....
148693972   .....SVMTQ.....LVQLL.....YIVTE.....FVHRD.....SDFGL.....KSDVW.....
148699491   .....AVMTK.....LVRLL.....YIVME.....LVHRD.....SDFGL.....KSDVW.....
148699494   .....AVMTK.....LVRLL.....YIVME.....LVHRD.....SDFGL.....KSDVW.....
148726069   .....SVMTN.....LVPLL.....HIVTE.....LLHRD.....SDFGL.....QSDVW.....
149691774   .....SVMTQ.....LVQLL.....YIVTE.....FVHRD.....SDFGL.....KSDVW.....
157130674   .....KQLSR.....VAKLI.....CIVLD.....VVHRD.....CTLGT.....KSDVW.....
109017889   .....KIMSR.....IIHLL.....CMITE.....FVHRD.....ADFGM.....ASDVW.....
110758281   .....RFLSS.....VARIL.....WTIIE.....LVHKD.....TDIAM.....SSSVW.....
111120292   .....KIMSR.....IIRLL.....CMITD.....FVHRD.....ADFGM.....ASDVW.....
118404968   .....KIISR.....IIRLL.....CMITE.....FVHRD.....ADFGM.....CSDAW.....
116007087   .....KQLAQ.....VARLV.....CIVQD.....FVHRD.....CSIGT.....KSDVW.....
114561021   .....KIMSR.....IIHLL.....CMITE.....FVHRD.....ADFGM.....ASDVW.....
114561035   .....KIMSR.....IIHLL.....CMITE.....FVHRD.....ADFGM.....ASDVW.....
114561033   .....KIMSR.....IIHLL.....CMITE.....FVHRD.....ADFGM.....ASDVW.....
115724315   .....KIMSQ.....IVRLL.....CMIVE.....FVHRD.....ADFGM.....KTDVW.....
116283854   .....KIMSR.....IIRLL.....CMITD.....FVHRD.....ADFGM.....ASDVW.....
118094070   .....KIMSR.....IIRLL.....CMITE.....FVHRD.....ADFGM.....ASDVW.....
```

```
119623745   ......KIMSR......IIRLL......CMITD......FVHRD......ADFGM......ASDVW......
119623742   ......KIMSR......IIRLL......CMITD......FVHRD......ADFGM......ASDVW......
125805312   ......RIISR......IIRLL......CMITE......FVHRD......ADFGM......ASDVW......
125822566   ......KIMSR......IIRLL......CMITE......FVHRD......ADFGM......SSDVW......
125846714   ......KIMSR......IIQLL......CMVTE......FVHRD......SDFGM......ASDVW......
126306249   ......KIMSR......IIRLL......CMITE......FVHRD......ADFGM......ASDVW......
139949224   ......KIMSR......IIRLL......CMITE......FVHRD......ADFGM......ASDVW......
148691281   ......KIMSR......IIRLL......CMITD......FVHRD......ADFGM......ASDVW......
148691280   ......KIMSR......IIRLL......CMITD......FVHRD......ADFGM......ASDVW......
148691279   ......KIMSR......IIRLL......CMITD......FVHRD......ADFGM......ASDVW......
148707217   ......KIMSR......IIRLL......CMITE......FVHRD......ADFGM......ASDVW......
148725482   ......RIISR......IIRLL......CMITE......FVHRD......ADFGM......ASDVW......
149567694   ......KIMSR......IIRLL......CMITE......FVHRD......ADFGM......ASDVW......
149754666   ......KIMSR......IIRLL......CMITD......FVHRD......ADFGM......ASDVW......
149754668   ......KIMSR......IIRLL......CMITD......FVHRD......ADFGM......ASDVW......
149754670   ......KIMSR......IIRLL......CMITD......FVHRD......ADFGM......ASDVW......
149755764   ......KIMSR......IIRLL......CMITE......FVHRD......ADFGM......ASDVW......
123281395   ......KIMSR......IIRLL......CMITD......FVHRD......ADFGM......ASDVW......
157105492   ......YIMAS......LLKLL......MLITQ......LVHRD......TDFGL......KSDVW......
109066534   ......YVMAS......VCRLL......QLITQ......LVHRD......TDFGL......QSDVW......
109114897   ......YVMAG......VSRLL......QLVTQ......LVHRD......TDFGL......QSDVW......
109114894   ......YVMAG......VSRLL......QLVTQ......LVHRD......TDFGL......QSDVW......
109631108   ......YVMAS......VCRLL......QLITQ......LVHRD......TDFGL......QSDVW......
147906005   ......LIMAS......LVRLL......QLVTQ......LVHRD......TDFGL......QSDVW......
148229369   ......YVMAG......VCRLL......QLVTQ......LVHRD......TDFGL......QSDVW......
110590405   ......YVMAS......VCRLL......QLITQ......LVHRD......TDFGL......QSDVW......
110590401   ......YVMAS......VCRLL......QLITQ......LVHRD......TDFGL......QSDVW......
110825958   ......LIMAS......LVRLL......QLVTQ......LVHRD......TDFGL......QSDVW......
114583090   ......LIMAS......LVRLL......QLVTQ......LVHRD......TDFGL......QSDVW......
114583088   ......LIMAS......LVRLL......QLVTQ......LVHRD......TDFGL......QSDVW......
114613405   ......YVMAS......VCRLL......QLITQ......LVHRD......TDFGL......QKKVW......
114613399   ......YVMAS......VCRLL......QLITQ......LVHRD......TDFGL......QKKVW......
114613403   ......YVMAS......VCRLL......QLITQ......LVHRD......TDFGL......QKKVW......
114613401   ......YVMAS......VCRLL......QLITQ......LVHRD......TDFGL......QKKVW......
114613407   ......YVMAS......VCRLL......QLITQ......LVHRD......TDFGL......QKKVW......
118785876   ......YIMAS......LLKLL......MLITQ......LVHRD......TDFGL......KSDVW......
119920730   ......YVMAS......VCRLL......QLITQ......LVHRD......TDFGL......QSDVW......
122065178   ......LIMAS......LVRLL......QLVTQ......LVHRD......TDFGL......QSDVW......
126308198   ......YVMAG......VSRLL......QLVTQ......LVHRD......TDFGL......QSDVW......
126336558   ......YVMAS......VCRLL......QLITQ......LVHRD......TDFGL......QSDVW......
126337964   ......LIMAS......LVRLL......QLVTQ......LVHRD......TDFGL......QSDVW......
134104655   ......YVMAS......VCRLL......QLITQ......LVHRD......TDFGL......QSDVW......
134104654   ......YVMAS......VCRLL......QLITQ......LVHRD......TDFGL......QSDVW......
145579713   ......YVMAS......VCRLL......QLITQ......LVHRD......TDFGL......QSDVW......
145579717   ......YVMAS......VCRLL......QLITQ......LVHRD......TDFGR......QSDVW......
146741286   ......YVMAS......VCRLL......QLITQ......LVHRD......TDFGL......QSDVW......
148692639   ......LAVGS......IVRLL......QLVTQ......MVHRD......ADFGV......QSDVW......
149044710   ......YVMAS......VCRLL......QLITQ......LVHRD......TDFGL......QSDVW......
149029663   ......LAVGS......IVRLL......QLVTQ......MVHRD......ADFGV......QSDVW......
149029664   ......LAVGS......IVRLL......QLVTQ......MVHRD......ADFGV......QSDVW......
149054114   ......YVMAG......VSRLL......QLVTQ......LVHRD......TDFGL......QSDVW......
149054113   ......YVMAG......VSRLL......QLVTQ......LVHRD......TDFGL......QSDVW......
149411341   ......LIMAT......LVRLL......QLVTQ......LVHRD......TDFGL......QSDVW......
149588160   ......YVMAS......VCRLL......QLITQ......LVHRD......TDFGL......QSDVW......
149704865   ......YVMAS......VCRLL......QLITQ......LVHRD......TDFGL......QSDVW......
149710107   ......LIMAS......LVRLL......QLVTQ......LVHRD......TDFGL......QSDVW......
149723876   ......YVMAG......VSRLL......QLVTQ......LVHRD......TDFGL......QSDVW......
108998935   ......SIMGQ......IIRLE......MIVTE......YVHRD......SDFGL......ASDVW......
109029836   ......SIMGQ......IIRLE......MIVTE......YVHRD......SDFGL......ASDVW......
109029839   ......SIMGQ......IIRLE......MIVTE......YVHRD......SDFGL......ASDVW......
109032572   ......SIMGQ......IIRLE......MIVVE......YVHRD......SDFGL......ASDAW......
109032575   ......SIMGQ......IIRLE......MIVVE......YVHRD......SDFGL......ASDAW......
109042392   ......SIMGQ......IIRLE......MILTE......YVHRD......SDFGL......ASDVW......
109049319   ......SIMGQ......IIRLE......MIITE......YVHRD......SDFGL......ASDVW......
109049310   ......SIMGQ......IIRLE......MIITE......YVHRD......SDFGL......ASDVW......
109049316   ......SIMGQ......IIRLE......MIITE......YVHRD......SDFGL......ASDVW......
109049307   ......SIMGQ......IIRLE......MIITE......YVHRD......SDFGL......ASDVW......
109068709   ......TIMGQ......ILHLE......MIITE......YVHRD......SDFGL......ASDVW......
```

```
109068711     ......TIMGQ.....ILHLE.....MITE.....YVHRD.....SDFGL.....ASDVW.....
109074683     ......SIMGQ.....IIHLE.....MIVTE.....YVHRD.....SDFGL.....ASDVW.....
109074675     ......SIMGQ.....IIHLE.....MIVTE.....YVHRD.....SDFGL.....ASDVW.....
109074677     ......SIMGQ.....IIHLE.....MIVTE.....YVHRD.....SDFGL.....ASDVW.....
109074679     ......SIMGQ.....IIHLE.....MIVTE.....YVHRD.....SDFGL.....ASDVW.....
109074681     ......SIMGQ.....IIHLE.....MIVTE.....YVHRD.....SDFGL.....ASDVW.....
109074687     ......SIMGQ.....IIHLE.....MIVTE.....YVHRD.....SDFGL.....ASDVW.....
109074685     ......SIMGQ.....IIHLE.....MIVTE.....YVHRD.....SDFGL.....ASDVW.....
109074689     ......SIMGQ.....IIHLE.....MIVTE.....YVHRD.....SDFGL.....ASDVW.....
109074695     ......SIMGQ.....IIHLE.....MIVTE.....YVHRD.....SDFGL.....ASDVW.....
109074691     ......SIMGQ.....IIHLE.....MIVTE.....YVHRD.....SDFGL.....ASDVW.....
109074697     ......SIMGQ.....IIHLE.....MIVTE.....YVHRD.....SDFGL.....ASDVW.....
109074699     ......SIMGQ.....IIHLE.....MIVTE.....YVHRD.....SDFGL.....ASDVW.....
109101183     ......SIMGQ.....IIHLE.....MIITE.....YVHRD.....SDFGM.....ASDVW.....
109101189     ......SIMGQ.....IIHLE.....MIITE.....YVHRD.....SDFGM.....ASDVW.....
109101179     ......SIMGQ.....IIHLE.....MIITE.....YVHRD.....SDFGM.....ASDVW.....
109101193     ......SIMGQ.....IIHLE.....MIITE.....YVHRD.....SDFGM.....ASDVW.....
109101177     ......SIMGQ.....IIHLE.....MIITE.....YVHRD.....SDFGM.....ASDVW.....
109101185     ......SIMGQ.....IIHLE.....MIITE.....YVHRD.....SDFGM.....ASDVW.....
109101181     ......SIMGQ.....IIHLE.....MIITE.....YVHRD.....SDFGM.....ASDVW.....
109101187     ......SIMGQ.....IIHLE.....MIITE.....YVHRD.....SDFGM.....ASDVW.....
109101191     ......SIMGQ.....IIHLE.....MIITE.....YVHRD.....SDFGM.....ASDVW.....
109475684     ......SIMGQ.....VIHLE.....MIITE.....YVHRD.....SDFGL.....ASDVW.....
109475686     ......AIMGQ.....IIRLE.....MIVTE.....YIHRD.....SDFGL.....ASDVW.....
157822929     ......SIMGQ.....IIRLE.....MIITE.....YVHRD.....SDFGL.....ASDVW.....
109477533     ......SIMGQ.....VIHLE.....MIITE.....YVHRD.....SDFGL.....ASDVW.....
109493030     ......INMGN.....CESLP.....MIVVE.....YVHRD.....SDFGL.....ASDAW.....
109494196     ......SIMGQ.....IIRLE.....MIVVE.....YVHRD.....SDFGL.....ASDAW.....
109497070     ......SIMGQ.....IIRLE.....MILTE.....YVHRD.....SDFGL.....ASDAW.....
109497068     ......SIMGQ.....IIRLE.....MILTE.....YVHRD.....SDFGL.....ASDAW.....
109497066     ......SIMGQ.....IIRLE.....MILTE.....YVHRD.....SDFGL.....ASDAW.....
109497064     ......SIMGQ.....IIRLE.....MILTE.....YVHRD.....SDFGL.....ASDAW.....
110590407     ......SIMGQ.....IIRLE.....MIVTE.....YVHRD.....SDFGL.....ASDVW.....
111118978     ......SIMGQ.....VIHLE.....MIITE.....YVHRD.....SDFGL.....ASDVW.....
111154065     ......SIMGQ.....IIHLE.....MIVTE.....YVHRD.....SDFGL.....SSDVW.....
111185530     ......SIMGQ.....IIRLE.....MIVTE.....YVHRD.....SDFGL.....ASDVW.....
111598956     ......SIMGQ.....IIRLE.....MIITE.....YVHRD.....SDFGL.....ASDVW.....
114554226     ......AHLPP.....PLHLP.....MIITE.....YVHRD.....SDFGL.....ASDVW.....
114554617     ......SIMGQ.....VIHLE.....MIITE.....YVHRD.....SDFGL.....ASDVW.....
114583543     ......SIMGQ.....IIHLE.....MIITE.....YVHRD.....SDFGM.....ASDVW.....
114583545     ......SIMGQ.....IIHLE.....MIITE.....YVHRD.....SDFGM.....ASDVW.....
114583531     ......SIMGQ.....IIHLE.....MIITE.....YVHRD.....SDFGM.....ASDVW.....
114583535     ......SIMGQ.....IIHLE.....MIITE.....YVHRD.....SDFGM.....ASDVW.....
114583549     ......SIMGQ.....IIHLE.....MIITE.....YVHRD.....SDFGM.....ASDVW.....
114583547     ......SIMGQ.....IIHLE.....MIITE.....YVHRD.....SDFGM.....ASDVW.....
114583537     ......SIMGQ.....IIHLE.....MIITE.....YVHRD.....SDFGM.....ASDVW.....
114583539     ......SIMGQ.....IIHLE.....MIITE.....YVHRD.....SDFGM.....ASDVW.....
114594541     ......SIMGQ.....IIHLE.....MIVTE.....YVHRD.....SDFGL.....ASDVW.....
114588027     ......SIMGQ.....IIRLE.....MIVTE.....YVHRD.....SDFGL.....ASDVW.....
114594553     ......SIMGQ.....IIHLE.....MIVTE.....YVHRD.....SDFGL.....ASDVW.....
114590829     ......SIMGQ.....IIRLE.....MILTE.....YVHRD.....SDFGL.....ASDVW.....
114588033     ......SIMGQ.....IIRLE.....MIVTE.....YVHRD.....SDFGL.....ASDVW.....
114594559     ......SIMGQ.....IIHLE.....MIVTE.....YVHRD.....SDFGL.....ASDVW.....
114589292     ......SIMGQ.....IIRLE.....MIITE.....YVHRD.....SDFGL.....ASDVW.....
114589294     ......SIMGQ.....IIRLE.....MIITE.....YVHRD.....SDFGL.....ASDVW.....
114594561     ......SIMGQ.....IIHLE.....MIVTE.....YVHRD.....SDFGL.....ASDVW.....
114589284     ......SIMGQ.....IIRLE.....MIITE.....YVHRD.....SDFGL.....ASDVW.....
114588029     ......SIMGQ.....IIRLE.....MIVTE.....YVHRD.....SDFGL.....ASDVW.....
114588089     ......SIMGQ.....IIRLE.....MIVVE.....YVHRD.....SDFGL.....ASDAW.....
114589290     ......SIMGQ.....IIRLE.....MIITE.....YVHRD.....SDFGL.....ASDVW.....
114594549     ......SIMGQ.....IIHLE.....MIVTE.....YVHRD.....SDFGL.....ASDVW.....
114594551     ......SIMGQ.....IIHLE.....MIVTE.....YVHRD.....SDFGL.....ASDVW.....
114589298     ......SIMGQ.....IIRLE.....MIITE.....YVHRD.....SDFGL.....ASDVW.....
114594547     ......SIMGQ.....IIHLE.....MIVTE.....YVHRD.....SDFGL.....ASDVW.....
114588031     ......SIMGQ.....IIRLE.....MIVTE.....YVHRD.....SDFGL.....ASDVW.....
114594555     ......SIMGQ.....IIHLE.....MIVTE.....YVHRD.....SDFGL.....ASDVW.....
```

114589286	......SIMGQ.....IIRLE.....MIITE.....YVHRD.....SDFGL.....ASDVW......
114589308	......SIMGQ.....IIRLE.....MIITE.....YVHRD.....SDFGL.....ASDVW......
114589296	......SIMGQ.....IIRLE.....MIITE.....YVHRD.....SDFGL.....ASDVW......
114594545	......SIMGQ.....IIHLE.....MIVTE.....YVHRD.....SDFGL.....ASDVW......
114589304	......SIMGQ.....IIRLE.....MIITE.....YVHRD.....SDFGL.....ASDVW......
114589302	......SIMGQ.....IIRLE.....MIITE.....YVHRD.....SDFGL.....ASDVW......
114594543	......SIMGQ.....IIHLE.....MIVTE.....YVHRD.....SDFGL.....ASDVW......
114589300	......SIMGQ.....IIRLE.....MIITE.....YVHRD.....SDFGL.....ASDVW......
114589306	......SIMGQ.....IIRLE.....MIITE.....YVHRD.....SDFGL.....ASDVW......
114615050	......SIMGQ.....IIRLE.....MILTE.....YVHRD.....SDFGL.....ASDAW......
114616533	......TIMGQ.....ILHLE.....MIITE.....YVHRD.....SDFGL.....ASDVW......
114616531	......TIMGQ.....ILHLE.....MIITE.....YVHRD.....SDFGL.....ASDVW......
115688582	......SIMGQ.....VIKLL.....MIVTE.....FVHRD.....ADFGL.....ASDVW......
115948839	......SIMGQ.....VIKLL.....MIVTE.....FVHRD.....ADFGL.....ASDVW......
117644922	......SIMGQ.....IIHLE.....MIVTE.....YVHRD.....SDFEL.....ASDVW......
117645796	......SIMGQ.....IIHLE.....MIVTE.....YVHRD.....SDFEP.....ASDVW......
118083672	......SIMGQ.....IIRLE.....MIVVE.....YVHRD.....SDFGL.....ASDAW......
118094889	......SIMGQ.....IIRLE.....MIITE.....YVHRD.....SDFGL.....ASDVW......
118095239	......SIMGQ.....IIHLE.....MIITE.....YVHRD.....SDFGL.....ASDVW......
119568903	......SIMGQ.....VVHLE.....MIVIE.....YVHRD.....SDFGL.....ASDVW......
119598642	......SIMGQ.....IIRLE.....MILTE.....YVHRD.....SDFGL.....ASDVW......
119878724	......SIMGQ.....IIRLE.....MIVTE.....YVHRD.....SDFGL.....ASDVW......
119879066	......SIMGQ.....IIRLE.....MIVVE.....YVHRD.....SDFGL.....ASDAW......
119878741	......SIMGQ.....IIRLE.....MIVTE.....YVHRD.....SDFGL.....ASDVW......
119888835	......SIMGQ.....IIRLE.....MIVTE.....YVHRD.....SDFGL.....ASDVW......
119888953	......SIMGQ.....IIRLE.....MIITE.....YVHRD.....SDFGL.....ASDVW......
119893979	......SIMGQ.....IIHLE.....MIVTE.....YVHRD.....SDFGL.....ASDVW......
119625941	......SIMGQ.....IIHLE.....MIVTE.....YVHRD.....SDFGL.....ASDVW......
119600292	......SIMGQ.....IIRLE.....MIVVE.....YVHRD.....SDFGL.....ASDAW......
119599535	......SIMGQ.....IIRLE.....MIITE.....YVHRD.....SDFGL.....ASDVW......
119600293	......SIMGQ.....IIRLE.....MIVVE.....YVHRD.....SDFGL.....ASDAW......
119615431	......SIMGQ.....VIHLE.....MIITE.....YVHRD.....SDFGL.....ASDVW......
119615429	......SIMGQ.....VIHLE.....MIITE.....YVHRD.....SDFGL.....ASDVW......
119615430	......SIMGQ.....VIHLE.....MIITE.....YVHRD.....SDFGL.....ASDVW......
119625943	......SIMGQ.....IIHLE.....MIVTE.....YVHRD.....SDFGL.....ASDVW......
119625942	......SIMGQ.....IIHLE.....MIVTE.....YVHRD.....SDFGL.....ASDVW......
119901379	......SIMGQ.....VVHLE.....MIVIE.....YVHRD.....SDFGL.....ASDVW......
119917187	......SIMGQ.....IIRLE.....MILTE.....YVHRD.....SDFGL.....ASDAW......
122891068	......SIMGQ.....IIRLE.....MIITE.....YVHRD.....SDFGM.....ASDVW......
122891069	......SIMGQ.....IIRLE.....MIITE.....YVHRD.....SDFGM.....ASDVW......
125844167	......SIMGQ.....IIRLE.....MIVTE.....YVHRD.....SDFGL.....ASDVW......
122891188	......SIMGQ.....VIHLE.....MIITE.....YVHRD.....SDFGL.....ASDVW......
123704646	......SIMGQ.....VVHLE.....MIVIE.....YVHRD.....SDFGL.....ASDVW......
123285543	......SIMGQ.....VVHLE.....MIVIE.....YVHRD.....SDFGL.....ASDVW......
125803990	......SIMGQ.....IIHLE.....MIVVE.....YVHRD.....SDFGL.....ASDAW......
125806276	......SIMGQ.....IIRLE.....MIITE.....YVHRD.....SDFGM.....ASDVW......
125828173	......SIMGQ.....IIHLE.....MIVVE.....YVHRD.....SDFGL.....ASDAW......
125838407	......SVIGQ.....IIHLE.....MIITE.....YVHRD.....SDFGM.....ASDVW......
125840137	......SIMGQ.....IIRLE.....MIVTE.....YVHRD.....SDFGL.....ASDVW......
125847121	......SIMAQ.....VIHLE.....MIITE.....YVHRD.....SDFGL.....SSDVW......
125851906	......SIMGQ.....IIRLE.....MIVTE.....YVHRD.....SDFGL.....ASDVW......
126310341	......SIMGQ.....VVHLE.....MIVIE.....YVHRD.....SDFGL.....ASDVW......
126326021	......SIMGQ.....IIRLE.....MIITE.....YVHRD.....SDFGL.....ASDVW......
126331651	......SIMGQ.....IIHLE.....MIVTE.....YVHRD.....SDFGL.....ASDVW......
126336946	......SIMGQ.....IIRLE.....MIVVE.....YVHRD.....SDFGL.....ASDAW......
126336936	......SIMGQ.....IIRLE.....MIVTE.....YVHRD.....SDFGL.....ASDVW......
126338198	......SIMGQ.....IIHLE.....MIITE.....YVHRD.....SDFGM.....ASDVW......
126340975	......TIMSQ.....ILHLE.....MIITE.....YVHRD.....SDFGL.....ASDVW......
154240697	......AIMGQ.....IIRLE.....MIVTE.....YIHRD.....SDFGL.....ASDVW......
134104446	......SIMGQ.....IIHLE.....MIITE.....AVHRD.....SDFGM.....ASDVW......
144228147	......SIMGQ.....VIFLE.....MIVTE.....FVHRD.....ADFGL.....ASDVW......
145312251	......SIMGQ.....IIRLE.....MIVVE.....YVHRD.....SDFGL.....ASDAW......
145312274	......SIMGQ.....IIRLE.....MIVVE.....YVHRD.....SDFGL.....ASDAW......
148665816	......SIMGQ.....IIRLE.....MIVVE.....YVHRD.....SDFGL.....ASDAW......
148665185	......SIMGQ.....IIRLE.....MILTE.....YVHRD.....SDFGL.....ASDVW......
148665184	......SIMGQ.....IIRLE.....MILTE.....YVHRD.....SDFGL.....ASDVW......

148665822 ......SIMGQ.....IRLE.....MIVTE.....YVHRD.....SDFGL.....ASDVW.....
148689100 ......SIMGQ.....IRLE.....MIITE.....YVHRD.....SDFGL.....ASDVW.....
148687327 ......SIMGQ.....IRLE.....MILTE.....YVHRD.....SDFGL.....ASDAW.....
148697983 ......SIMGQ.....VIHLE.....MIITE.....YVHRD.....SDFGL.....ASDVW.....
148706002 ......SIMGQ.....IHLE.....MIVTE.....YVHRD.....SDFGL.....SSDVW.....
148697978 ......AIMGQ.....IRLE.....MIVTE.....YIHRD.....SDFGL.....ASDVW.....
148706001 ......SIMGQ.....IHLE.....MIVTE.....YVHRD.....SDFGL.....SSDVW.....
148706003 ......SIMGQ.....IHLE.....MIVTE.....YVHRD.....SDFGL.....SSDVW.....
149016216 ......SIMGQ.....IHLE.....MIITE.....YVHRD.....SDFGM.....ASDVW.....
157787046 ......SIMGQ.....IRLE.....MILTE.....YVHRD.....SDFGL.....ASDVW.....
149024325 ......SIMGQ.....VIHLE.....MIITE.....YVHRD.....SDFGL.....ASDVW.....
149024331 ......AIMGQ.....IRLE.....MIVTE.....YIHRD.....SDFGL.....ASDVW.....
149060271 ......SIMGQ.....IRLE.....MIVVE.....YVHRD.....SDFGL.....ASDAW.....
149408815 ......SIMGQ.....IHLE.....MIITE.....YVHRD.....SDFGM.....ASDVW.....
149445096 ......SIMGQ.....IRLE.....MIVVE.....YVHRD.....SDFGL.....ASDAW.....
149572970 ......SIMGQ.....IRLE.....MIITE.....YVHRD.....SDFGL.....ASDVW.....
149602994 ......SIMGQ.....IRLE.....MILTE.....YVHRD.....SDFGL.....ASDAW.....
149632896 ......SIMGQ.....IRLE.....MIVTE.....YVHRD.....SDFGL.....ASDVW.....
149635594 ......SIMGQ.....IHLE.....MIVTE.....YVHRD.....SDFGL.....ASDVW.....
149640520 ......SIMGQ.....VVHLE.....MIVIE.....YVHRD.....SDFGL.....ASDVW.....
149695183 ......SIMGQ.....VIHLE.....MIITE.....YVHRD.....SDFGL.....ASDVW.....
149695187 ......SIMGQ.....IRLE.....MIVTE.....YVHRD.....SDFGL.....ASDVW.....
149695472 ......SIMGQ.....IRLE.....MIITE.....YVHRD.....SDFGL.....ASDVW.....
149706693 ......TIMGQ.....ILHLE.....MIITE.....YVHRD.....SDFGL.....ASDVW.....
149711526 ......SIMGQ.....IHLE.....MIITE.....YVHRD.....SDFGM.....ASDVW.....
149722808 ......SIMGQ.....VVHLE.....MIVIE.....YVHRD.....SDFGL.....ASDVW.....
149729747 ......SIMGQ.....IRLE.....MIITE.....YVHRD.....SDFGL.....ASDVW.....
149731442 ......SIMGQ.....IRLE.....MILTE.....YVHRD.....SDFGL.....ASDVW.....
149731740 ......SIMGQ.....IRLE.....MIVVE.....YVHRD.....SDFGL.....ASDAW.....
149742246 ......SIMGQ.....IRLE.....MIVTE.....YVHRD.....SDFGL.....ASDVW.....
149742248 ......SIMGQ.....IRLE.....MIVTE.....YVHRD.....SDFGL.....ASDVW.....
149751615 ......SIMGQ.....IHLE.....MIVTE.....YVHRD.....SDFGL.....ASDVW.....
149751619 ......SIMGQ.....IHLE.....MIVTE.....YVHRD.....SDFGL.....ASDVW.....
149751617 ......SIMGQ.....IHLE.....MIVTE.....YVHRD.....SDFGL.....ASDVW.....
149757782 ......SIMGQ.....IRLE.....MILTE.....YVHRD.....SDFGL.....ASDAW.....
151358090 ......SIMGQ.....VIHLE.....MIITE.....YVHRD.....SDFGL.....ASDVW.....
151358092 ......SIMGQ.....VIHLE.....MIITE.....YVHRD.....SDFGL.....ASDVW.....
157108016 ......EVMKM.....INLL.....YVIVE.....CIHRD.....ADFGL.....QSDVW.....
157167533 ......EVMKM.....INLL.....YVIVE.....CIHRD.....ADFGL.....QSDVW.....
108994961 ......EMMKM.....INLL.....YVLVE.....CIHRD.....ADFGL.....QSDVW.....
108994958 ......EMMKM.....INLL.....YVLVE.....CIHRD.....ADFGL.....QSDVW.....
109079866 ......EVMKL.....INLL.....YVIVE.....CIHRD.....ADFGL.....QSDVW.....
109086174 ......EMMKM.....INLL.....YVIVE.....CIHRD.....ADFGL.....QSDVW.....
109090842 ......EMMKM.....INLL.....PAFRA.....CIHRD.....ADFGL.....QSDVW.....
149067597 ......EMMKM.....INLL.....YVIVE.....CIHRD.....ADFGL.....QSDVW.....
110278396 ......EMMKM.....INLL.....YVIVE.....CIHRD.....ADFGL.....QSDVW.....
110763144 ......EMMKM.....INLL.....YVVVE.....CIHRD.....ADFGL.....QSDVW.....
113197970 ......EMMKM.....INLL.....YVLVE.....CIHRD.....ADFGL.....QSDVW.....
114619755 ......EMMKM.....INLL.....YVIVE.....CIHRD.....ADFGL.....QSDVW.....
114619739 ......EMMKM.....INLL.....YVIVE.....CIHRD.....ADFGL.....QSDVW.....
114619743 ......EMMKM.....INLL.....YVIVE.....CIHRD.....ADFGL.....QSDVW.....
114619757 ......EMMKM.....INLL.....YVIVE.....CIHRD.....ADFGL.....QSDVW.....
114619735 ......EMMKM.....INLL.....YVIVE.....CIHRD.....ADFGL.....QSDVW.....
114619725 ......EMMKM.....INLL.....YVIVE.....CIHRD.....ADFGL.....QSDVW.....
114619741 ......EMMKM.....INLL.....YVIVE.....CIHRD.....ADFGL.....QSDVW.....
114619731 ......EMMKM.....INLL.....YVIVE.....CIHRD.....ADFGL.....QSDVW.....
114619759 ......EMMKM.....INLL.....YVIVE.....CIHRD.....ADFGL.....QSDVW.....
114619745 ......EMMKM.....INLL.....YVIVE.....CIHRD.....ADFGL.....QSDVW.....
114619729 ......EMMKM.....INLL.....YVIVE.....CIHRD.....ADFGL.....QSDVW.....
114619753 ......EMMKM.....INLL.....YVIVE.....CIHRD.....ADFGL.....QSDVW.....
114619751 ......EMMKM.....INLL.....YVIVE.....CIHRD.....ADFGL.....QSDVW.....
114619749 ......EMMKM.....INLL.....YVIVE.....CIHRD.....ADFGL.....QSDVW.....
114619727 ......EMMKM.....INLL.....YVIVE.....CIHRD.....ADFGL.....QSDVW.....
114633092 ......EMMKM.....INLL.....YVIVE.....CIHRD.....ADFGL.....QSDVW.....
114633068 ......EMMKM.....INLL.....YVIVE.....CIHRD.....ADFGL.....QSDVW.....
114633086 ......EMMKM.....INLL.....YVIVE.....CIHRD.....ADFGL.....QSDVW.....

```
114633084    ......EMMKM.....IINLL.....YVIVE.....CIHRD.....ADFGL.....QSDVW.....
114633078    ......EMMKM.....IINLL.....YVIVE.....CIHRD.....ADFGL.....QSDVW.....
114633070    ......EMMKM.....IINLL.....YVIVE.....CIHRD.....ADFGL.....QSDVW.....
114633072    ......EMMKM.....IINLL.....YVIVE.....CIHRD.....ADFGL.....QSDVW.....
114633080    ......EMMKM.....IINLL.....YVIVE.....CIHRD.....ADFGL.....QSDVW.....
114633082    ......EMMKM.....IINLL.....YVIVE.....CIHRD.....ADFGL.....QSDVW.....
114633090    ......EMMKM.....IINLL.....YVIVE.....CIHRD.....ADFGL.....QSDVW.....
114633088    ......EMMKM.....IINLL.....YVIVE.....CIHRD.....ADFGL.....QSDVW.....
114633074    ......EMMKM.....IINLL.....YVIVE.....CIHRD.....ADFGL.....QSDVW.....
108735559    ......AMMKM.....IINLL.....YVIVE.....CIHRD.....CDFGL.....QSDVW.....
119569733    ......EMMKM.....IINLL.....YVIVE.....CIHRD.....ADFGL.....QSDVW.....
119569726    ......EMMKM.....IINLL.....YVIVE.....CIHRD.....ADFGL.....QSDVW.....
119569731    ......EMMKM.....IINLL.....YVIVE.....CIHRD.....ADFGL.....QSDVW.....
119569739    ......EMMKM.....IINLL.....YVIVE.....CIHRD.....ADFGL.....QSDVW.....
119569730    ......EMMKM.....IINLL.....YVIVE.....CIHRD.....ADFGL.....QSDVW.....
119569724    ......EMMKM.....IINLL.....YVIVE.....CIHRD.....ADFGL.....QSDVW.....
119569740    ......EMMKM.....IINLL.....YVIVE.....CIHRD.....ADFGL.....QSDVW.....
119569729    ......EMMKM.....IINLL.....YVIVE.....CIHRD.....ADFGL.....QSDVW.....
119583719    ......EMMKM.....IINLL.....YVIVE.....CIHRD.....ADFGL.....QSDVW.....
119583708    ......EMMKM.....IINLL.....YVIVE.....CIHRD.....ADFGL.....QSDVW.....
119583714    ......EMMKM.....IINLL.....YVIVE.....CIHRD.....ADFGL.....QSDVW.....
119895168    ......EVMKL.....IINLL.....YVIVE.....CIHRD.....ADFGL.....QSDVW.....
119605440    ......EVMKL.....IINLL.....YVIVE.....CIHRD.....ADFGL.....QSDVW.....
119602968    ......EMMKM.....IINLL.....YVLVE.....CIHRD.....ADFGL.....QSDVW.....
119917855    ......EMMKM.....IINLL.....YVIVE.....CIHRD.....ADFGL.....QSDVW.....
119675394    ......EMMKM.....IINLL.....YVIVE.....CIHRD.....ADFGL.....QSDVW.....
119675396    ......EMMKM.....IINLL.....YVIVE.....CIHRD.....ADFGL.....QSDVW.....
119675402    ......EMMKM.....IINLL.....YVIVE.....CIHRD.....ADFGL.....QSDVW.....
119675418    ......EMMKM.....IINLL.....YVIVE.....CIHRD.....ADFGL.....QSDVW.....
119675400    ......EMMKM.....IINLL.....YVIVE.....CIHRD.....ADFGL.....QSDVW.....
119675424    ......EMMKM.....IINLL.....YVIVE.....CIHRD.....ADFGL.....QSDVW.....
119675416    ......EMMKM.....IINLL.....YVIVE.....CIHRD.....ADFGL.....QSDVW.....
119675420    ......EMMKM.....IINLL.....YVIVE.....CIHRD.....ADFGL.....QSDVW.....
119675430    ......EMMKM.....IINLL.....YVIVE.....CIHRD.....ADFGL.....QSDVW.....
119675428    ......EMMKM.....IINLL.....YVIVE.....CIHRD.....ADFGL.....QSDVW.....
119675398    ......EMMKM.....IINLL.....YVIVE.....CIHRD.....ADFGL.....QSDVW.....
119675404    ......EMMKM.....IINLL.....YVIVE.....CIHRD.....ADFGL.....QSDVW.....
119675410    ......EMMKM.....IINLL.....YVIVE.....CIHRD.....ADFGL.....QSDVW.....
119675406    ......EMMKM.....IINLL.....YVIVE.....CIHRD.....ADFGL.....QSDVW.....
119675412    ......EMMKM.....IINLL.....YVIVE.....CIHRD.....ADFGL.....QSDVW.....
119675422    ......EMMKM.....IINLL.....YVIVE.....CIHRD.....ADFGL.....QSDVW.....
119675426    ......EMMKM.....IINLL.....YVIVE.....CIHRD.....ADFGL.....QSDVW.....
119675408    ......EMMKM.....IINLL.....YVIVE.....CIHRD.....ADFGL.....QSDVW.....
122891104    ......EMMKI.....IINLL.....YVIVE.....CIHRD.....ADFGL.....QSDVW.....
148725237    ......EMMKI.....IINLL.....YVIVE.....CIHRD.....ADFGL.....QSDVW.....
124487848    ......EVMKM.....ILNLL.....FVITE.....CVHRD.....ADFGL.....KSDVW.....
125828582    ......EMMKM.....IINLL.....YVIVE.....CIHRD.....ADFGL.....QSDVW.....
126015615    ......DTMKN.....IINLI.....YVVVE.....CIHRD.....ADFGL.....QSDVW.....
126015617    ......EVMKT.....IINLI.....FVVVE.....CIHRD.....ADFGL.....QSDVW.....
126273351    ......EMMKM.....IINLL.....YVIVE.....CIHRD.....ADFGL.....QSDVW.....
126291579    ......EMMKL.....IINLL.....YVIVE.....CIHRD.....ADFGL.....QSDVW.....
126303365    ......EMMKM.....IINLL.....YVIVE.....CIHRD.....ADFGL.....QSDVW.....
126303367    ......EMMKM.....IINLL.....YVIVE.....CIHRD.....ADFGL.....QSDVW.....
126303363    ......EMMKM.....IINLL.....YVIVE.....CIHRD.....ADFGL.....QSDVW.....
126332238    ......EMMKM.....IINLL.....YVLVE.....CIHRD.....ADFGL.....QSDVW.....
133740950    ......EMMKI.....IINLL.....YVIVE.....CIHRD.....ADFGL.....QSDVW.....
133740938    ......EMMKI.....IINLL.....YVIVE.....CIHRD.....ADFGL.....QSDVW.....
157278533    ......EMMKI.....IINLL.....YVIVE.....CIHRD.....ADFGL.....QSDVW.....
133740953    ......EMMKI.....IINLL.....YVIVE.....CIHRD.....ADFGL.....QSDVW.....
133740952    ......EMMKI.....IINLL.....YVIVE.....CIHRD.....ADFGL.....QSDVW.....
133740939    ......EMMKI.....IINLL.....YVIVE.....CIHRD.....ADFGL.....QSDVW.....
133740940    ......EMMKI.....IINLL.....YVIVE.....CIHRD.....ADFGL.....QSDVW.....
133740951    ......EMMKI.....IINLL.....YVIVE.....CIHRD.....ADFGL.....QSDVW.....
156717572    ......EMMKI.....IINLL.....YVIVE.....CIHRD.....ADFGL.....QSDVW.....
134054352    ......EMMKM.....IINLL.....YVIVE.....CIHRD.....ADFGL.....QSDVW.....
134054480    ......EMMKM.....IINLL.....YVLVE.....CIHRD.....ADFGL.....QSDVW.....
```

116089355	......EMMKM......IINLL......YVIVE......CIHRD......ADFGL......QSDVW......
116089349	......EMMKM......IINLL......YVIVE......CIHRD......ADFGL......QSDVW......
141795158	......EMMKM......IINLL......YVIVE......CIHRD......ADFGL......QSDVW......
153792303	......EMMKM......IINLL......YVIVE......CIHRD......ADFGL......QSDVW......
148705486	......EMMKM......IINLL......YVLVE......CIHRD......ADFGL......QSDVW......
148725236	......EMMKI......IINLL......YVIVE......CIHRD......ADFGL......QSDVW......
149039891	......EMMKL......IINLL......YVIVE......CIHRD......ADFGL......QSDVW......
149047445	......EMMKM......IINLL......YVLVE......CIHRD......ADFGL......QSDVW......
149047446	......EMMKM......IINLL......YVLVE......CIHRD......ADFGL......QSDVW......
149057813	......EMMKM......IINLL......YVIVE......CIHRD......ADFGL......QSDVW......
149689623	......EMMKM......IINLL......YVIVE......CIHRD......ADFGL......QSDVW......
149689627	......EMMKM......IINLL......YVIVE......CIHRD......ADFGL......QSDVW......
149689621	......EMMKM......IINLL......YVIVE......CIHRD......ADFGL......QSDVW......
149689631	......EMMKM......IINLL......YVIVE......CIHRD......ADFGL......QSDVW......
149689629	......EMMKM......IINLL......YVIVE......CIHRD......ADFGL......QSDVW......
149689633	......EMMKM......IINLL......YVIVE......CIHRD......ADFGL......QSDVW......
149726621	......EVMKL......IINLL......YVIVE......CIHRD......ADFGL......QSDVW......
149742565	......EMMKM......IINLL......YVIVE......CIHRD......ADFGL......QSDVW......
149742563	......EMMKM......IINLL......YVIVE......CIHRD......ADFGL......QSDVW......
149742561	......EMMKM......IINLL......YVIVE......CIHRD......ADFGL......QSDVW......
149756980	......EMMKM......IINLL......YVLVE......CIHRD......ADFGL......QSDVW......
157105531	......RILKQ......IVKLI......MIVME......CIHRD......SDFGM......LCDVW......
109085443	......RILKQ......IVRLI......YIVME......VSPRD......SDFGM......ESDVW......
109085449	......RSSPS......LCR--......-----......VSPRD......SDFGM......ESDVW......
109085447	......RILKQ......IVRLI......YIVME......VSPRD......SDFGM......ESDVW......
109085445	......RILKQ......IVRLI......YIVME......VSPRD......SDFGM......ESDVW......
109085441	......RILKQ......IVRLI......YIVME......VSPRD......SDFGM......ESDVW......
157817059	......RILKQ......IVRLI......YIVME......CIHRD......SDFGM......ESDVW......
157817710	......KILKQ......IVKLI......YIIME......CIHRD......SDFGM......ESDVW......
133930931	......RFIRN......VVRLY......YILLE......CIHRD......TDFGL......ATDVY......
110749814	......DLLHK......VVKLI......YMILE......LVHKD......SLPCM......KSDVY......
114601071	......KILKQ......IVKLI......YIIME......CIHRD......SDFGM......ESDVW......
114658913	......RILKQ......IVRLI......YIVME......CIHRD......SDFGM......ESDVW......
114658903	......RILKQ......IVRLI......YIVME......CIHRD......SDFGM......ESDVW......
114658905	......RILKQ......IVRLI......YIVME......CIHRD......SDFGM......ESDVW......
114658925	......R----......-TSSW......-----......CIHRD......SDFGM......ESDVW......
114658917	......RILKQ......IVRLI......YIVME......CIHRD......SDFGM......ESDVW......
114658921	......RILKQ......IVRLI......YIVME......CIHRD......SDFGM......ESDVW......
115717936	......NILKQ......IVKLI......YIVME......CIHRD......SDFGM......MSDVW......
169855659	......EIWKT......VLELY......FFVSP......VLHGD......TDFGQ......EADVY......
118104229	......RILKQ......IVKLI......YIVME......CIHRD......SDFGM......ESDVW......
118572319	......KILKQ......IVKLI......YIIME......CIHRD......SDFGM......ESDVW......
119896002	......KILKQ......IVKLI......YIIME......CIHRD......SDFGM......ESDVW......
119622518	......RILKQ......IVRLI......YIVME......CIHRD......SDFGM......ESDVW......
123232989	......RILKQ......IVKLI......YIVME......CIHRD......SDFGM......ESDVW......
125817240	......RILKQ......IVKLI......YIVME......CIHRD......SDFGM......ESDVW......
125863903	......RILKQ......IVKLI......YIIME......CIHRD......SDFGM......ESDVW......
126273771	......RILKQ......IVRLI......YIVME......CIHRD......SDFGM......ESDVW......
126320586	......KILKQ......IVKLI......YIIME......CIHRD......SDFGM......ESDAW......
146232078	......RVMQL......IVKFY......LLVLE......CIHRD......ADFGM......NTDVY......
147776747	......QFLGI......LVKLI......LLVYE......VIYRD......SDFGL......KSDVW......
148675045	......RILKQ......IVRLI......YIVME......CIHRD......SDFGM......ESDVW......
148706338	......KILKQ......IVKLI......YIIME......CIHRD......SDFGM......ESDVW......
148706336	......KILKQ......IVKLI......YIIME......CIHRD......SDFGM......ESDVW......
149037400	......KILKQ......IVKLI......YIIME......CIHRD......SDFGM......ESDVW......
149566766	......RILKQ......IVRLI......YIVME......CIHRD......SDFGM......ESDVW......
149642271	......RILKQ......IVKLI......YIIME......CIHRD......SDFGM......ESDAW......
149642269	......RILKQ......IVKLI......YIIME......CIHRD......SDFGM......ESDAW......
149690944	......RILKQ......IVRLI......YIVME......CIHRD......SDFGM......ESDVW......
149726470	......KILKQ......IVKLI......YIIME......CIHRD......SDFGM......ESDVW......
149726468	......KILKQ......IVKLI......YIIME......CIHRD......SDFGM......ESDVW......
157127985	......SVMKQ......VVRLL......LVIME......FVHRD......GDFGM......SSDVF......
157124987	......EAMKR......IVKLL......YTIME......YVHRD......GDFGM......ASDVW......
109082458	......SVMKE......VVRLL......LVIME......FVHRD......GDFGM......YSDVW......
109658490	......SVMKG......VVRLL......LVVME......FVHRD......GDFGM......SSDMW......
110751262	......SVMKG......VVKLL......LVIME......FVHRD......GDFGM......YSDVW......
110759786	......SVMKN......IIKLI......FVIME......YVHRD......GDFGM......DSDVW......

110762270	......EVMKQ......IIKLL......LTVME......YVHRD......GDFGM......ASDVW......
114659080	......SVMKE......VVRLL......LVIME......FVHRD......GDFGM......YSDVW......
114659078	......SVMKE......VVRLL......LVIME......FVHRD......GDFGM......YSDVW......
114674965	......SVMKG......VVRLL......LVVME......FVHRD......GDFGM......SSDMW......
115673158	......EVMKR......IVNLL......YAVME......YVHRD......GDFGM......MSDVW......
115927306	......SVMKL......VVRLL......YVIME......YVHRD......ADFGL......SSDVW......
118792063	......TIMKE......VVRLY......LVIME......FVHRD......GDFGM......SSDVF......
118103311	......SVMKG......VVRLL......LVVME......FVHRD......GDFGM......YSDVW......
118103313	......SVMKG......VVRLL......LVVME......FVHRD......GDFGM......YSDVW......
118103315	......SVMKG......VVRLL......LVVME......FVHRD......GDFGM......YSDVW......
119395738	......SVMKG......VVRLL......LVVME......FVHRD......GDFGM......SSDMW......
119395736	......SVMKG......VVRLL......LVVME......FVHRD......GDFGM......SSDMW......
119589453	......SVMKG......VVRLL......LVVME......FVHRD......GDFGM......SSDMW......
119573289	......SVMKA......VVRLL......LVIME......FVHRD......GDFGM......HSDVW......
119589449	......SVMKG......VVRLL......LVVME......FVHRD......GDFGM......SSDMW......
119589451	......SVMKG......VVRLL......LVVME......FVHRD......GDFGM......SSDMW......
119589450	......SVMKG......VVRLL......LVVME......FVHRD......GDFGM......SSDMW......
119589452	......SVMKG......VVRLL......LVVME......FVHRD......GDFGM......SSDMW......
119589448	......SVMKG......VVRLL......LVVME......FVHRD......GDFGM......SSDMW......
119889338	......SVMKA......VVRLL......LVIME......FVHRD......GDFGM......HSDVW......
119622627	......SVMKE......VVRLL......LVIME......FVHRD......GDFGM......YSDVW......
119913553	......SVMKE......VVRLL......LVIME......FVHRD......GDFGM......HSDVW......
126277183	......SVMKE......VVRLL......LVIME......FVHRD......GDFGM......HSDVW......
126307856	......SVMKA......VVRLL......LVIME......FVHRD......GDFGM......HSDVW......
126323895	......SVMKG......VVRLL......LVVME......FVHRD......GDFGM......YSDVW......
112983656	......SVMKE......VVRLL......LVIME......FVHRD......GDFGM......HSDVW......
146386625	......SVMKE......VVRLL......LVIME......FVHRD......GDFGM......YSDVW......
148675224	......SVMKE......VVRLL......LVIME......FVHRD......GDFGM......HSDVW......
148675225	......SVMKE......VVRLL......LVIME......FVHRD......GDFGM......HSDVW......
160333073	......SVMKA......VVRLL......LVIME......FVHRD......GDFGM......HSDVW......
148689954	......SVMKG......VVRLL......LVVME......FVHRD......GDFGM......SSDMW......
149015543	......SVMKG......VVRLL......LVVME......FVHRD......GDFGM......SSDMW......
149015542	......SVMKG......VVRLL......LVVME......FVHRD......GDFGM......SSDMW......
149057149	......SVMKE......VVRLL......LVIME......-THRD......ADFGM......HSDVW......
149410821	......SVMKE......VVRLL......LVIME......FVHRD......GDFGM......HSDVW......
149691073	......SVMKE......VVRLL......LVIME......FVHRD......GDFGM......HSDVW......
149716743	......SVMKG......VVRLL......LVVME......FVHRD......GDFGM......SSDMW......
150034869	......SVMKE......VVRLL......LVIME......FVHRD......GDFGM......NSDVW......
151425900	......SVMKE......VVRLL......LVIME......FVHRD......GDFGM......HSDVW......
151425898	......SVMKE......VVRLL......LVIME......FVHRD......GDFGM......HSDVW......
109008250	......EILRN......IVKYK......KLIME......YVHRD......GDFGL......ASDVW......
109008254	......EILRN......IVKYK......KLIME......YVHRD......GDFGL......ASDVW......
109111641	......EILKS......IVKYK......KLIME......YIHRD......GDFGL......ASDVW......
109123904	......QILKA......IVKYR......RLVME......CVHRD......ADFGL......QSDVW......
109475184	......EILRN......IVKYK......KLIME......YVHRD......GDFGL......ASDVW......
111607496	......EILRN......IVKYK......KLIME......YVHRD......GDFGL......ASDVW......
114556983	......EILRN......IVKYK......KLIME......YVHRD......GDFGL......ASDVW......
114556989	......EILRN......IVKYK......KLIME......YVHRD......GDFGL......ASDVW......
114623672	......EILKS......IVKYK......KLIME......YIHRD......GDFGL......ASDVW......
114675359	......DILRT......IIKYK......QLVME......YIHRD......GDFGL......ASDVW......
114675363	......DILRT......IIKYK......QLVME......YIHRD......GDFGL......ASDVW......
114675361	......DILRT......IIKYK......QLVME......YIHRD......GDFGL......ASDVW......
114676079	......QILKA......IVKYR......RLVME......CVHRD......ADFGL......QSDVW......
115976934	......RAMSY......IVKVL......SLVME......MVHRD......TDFGL......ESDVW......
114326478	......EILKS......IVKYK......RLIME......YIHRD......GDFGL......ASDVW......
115764984	......RAMSY......IVKVL......SLVME......MVHRD......TDFGL......ESDVW......
148222248	......EILKS......IVRYK......RLIME......YIHRD......GDFGL......ASDVW......
166157490	......DILRK......IVKYK......QLVME......YVHRD......GDFGL......ASDVW......
119605043	......QILKA......IVKYR......RLVME......CVHRD......ADFGL......QSDVW......
119626953	......EILRN......IVKYK......KLIME......YVHRD......GDFGL......ASDVW......
119900352	......EILKS......IVKYK......RLIME......YIHRD......GDFGL......ASDVW......
124297739	......QILKA......IVKYR......RLVME......CVHRD......ADFGL......QSDVW......
126305995	......EILKK......IVKYK......KLIME......YVHRD......GDFGL......ASDVW......
126322899	......EILRT......IVKYK......ILIME......YIHRD......GDFGL......ASDVW......
126335718	......EILKS......IVKYK......RLIME......YIHRD......GDFGL......ASDVW......
126717429	......EILRN......IVKYK......KLIME......YVHRD......GDFGL......ASDVW......
148693212	......EILRT......IVKYK......QLVME......YIHRD......GDFGL......ASDVW......
148697007	......QILKA......IVKYR......RLVME......CVHRD......ADFGL......QSDVW......
148697008	......QILKA......IVKYR......RLVME......CVHRD......ADFGL......QSDVW......
148697010	......QILKA......IVKYR......RLVME......CVHRD......ADFGL......QSDVW......
149036095	......QILKA......IVKYR......RLVME......CVHRD......ADFGL......QSDVW......
149044565	......EILRN......IVKYK......KLIME......YVHRD......GDFGL......ASDVW......
149736849	......EILKS......IVKYK......RLIME......YIHRD......GDFGL......ASDVW......
109119005	......QPYRA......LLQCL......LLVME......YVHSD......GDYGL......SGNVW......

```
109489490 ......QPYRA.....LLQCL.....LLVME.....YVHSD.....GDYGL.....TSNVW.....
109495182 ......EPYYI.....VLQCV.....LLVFE.....FLHSD.....GDYGI.....YSNIW.....
110764546 ......TPYLK.....ILTLI.....LLLFE.....FVHTD.....GDYGT.....EANMW.....
122937191 ......QPYRA.....LLQCL.....LLVME.....FVHSD.....GDYGL.....SGNVW.....
114149222 ......QPYRA.....LLQCL.....LLVME.....FVHSD.....GDYGL.....SGNVW.....
114614765 ......EPYYI.....ILQCV.....LLVFE.....FLHSD.....GDYGI.....YSNIW.....
114614763 ......EPYYI.....ILQCV.....LLVFE.....FLHSD.....GDYGI.....YSNIW.....
115896439 ......QPYRE.....VLMLL.....LLVLE.....FIHID.....GDYGL.....KSNIW.....
117949603 ......QPYRS.....VLQCL.....LLIME.....YVHSD.....GDYGL.....ESNIW.....
118097709 ......EPYYI.....VLQCI.....LLVFE.....FVHSD.....GDYGI.....YSNIW.....
118573331 ......EPYYI.....VLQCV.....LLVFE.....FLHSD.....GDYGI.....YSNIW.....
119597126 ......EPYYI.....ILQCV.....LLVFE.....FLHSD.....GDYGI.....YSNIW.....
119572738 ......QPYRS.....VLQCL.....LLIME.....YVHSD.....GDYGL.....ESNIW.....
119917250 ......EPYYI.....VLQCV.....LLVFE.....FLHSD.....GDYGI.....YSNIW.....
119912672 ......QPYRA.....LLQCL.....LLVME.....FVHSD.....GDYGL.....ASNVW.....
125810902 ......QPYRV.....LLQCL.....LLVME.....YIHSD.....GDYGL.....ASNVW.....
125813436 ......DPYRV.....ILQCL.....LLVFE.....FLHSD.....GDYGF.....PSNVW.....
126308910 ......QPYRA.....LLQCL.....LLVME.....YVHSD.....GDYGL.....ASNVW.....
134024500 ......QPYRS.....ILQCL.....LLIME.....YIHSD.....GDYGI.....ESNVW.....
148690950 ......QPYRS.....VLQCL.....LLIME.....YVHSD.....GDYGL.....ESNVW.....
148687077 ......EPYYI.....VLQCV.....LLVFE.....FLHSD.....GDYGI.....YSNIW.....
149054993 ......QPYRA.....LLQCL.....LLVME.....YVHSD.....GDYGL.....TSNVW.....
149055865 ......QPYRS.....VLQCL.....LLIME.....YVHSD.....GDYGL.....ESNVW.....
149850244 ......QPYRS.....VLQCL.....LLIME.....YVHSD.....GDYGL.....ESNVW.....
109039581 ......LVEME.....-VGQA.....P---P.....FVHRD.....ADFGL.....KSDVW.....
109067950 ......IIMKD.....VLSLL.....LVVLP.....FVHRD.....ADFGL.....KSDVW.....
109067948 ......IIMKD.....VLSLL.....LVVLP.....FVHRD.....ADFGL.....KSDVW.....
115502262 ......IIMKD.....VLSLL.....LVVLP.....FVHRD.....ADFGL.....KSDVW.....
114615578 ......IIMKD.....VLSLL.....LVVLP.....FVHRD.....ADFGL.....KSDVW.....
118572794 ......IIMKD.....VLSLL.....LVVLP.....FVHRD.....ADFGL.....KSDVW.....
118572781 ......IIMKD.....VLSLL.....LVVLP.....FVHRD.....ADFGL.....KSDVW.....
162287029 ......IIMKD.....VLSLL.....LVVLP.....FVHRD.....ADFGL.....KSDVW.....
118572788 ......IIMKD.....VLSLL.....LVVLP.....FVHRD.....ADFGL.....KSDVW.....
114586951 ......LLMRG.....VLALI.....HVLLP.....FVHRD.....ADFGL.....KSDVW.....
118572784 ......IIMKD.....VLSLL.....LVVLP.....FVHRD.....ADFGL.....KSDVW.....
115299257 ......IIMKD.....VLSLL.....LVVLP.....FVHRD.....ADFGL.....KSDVW.....
118572791 ......IIMKD.....VLSLL.....LVVLP.....FVHRD.....ADFGL.....KSDVW.....
118572789 ......IIMKD.....VLSLL.....LVVLP.....FVHRD.....ADFGL.....KSDVW.....
118572783 ......IIMKD.....VLSLL.....LVVLP.....FVHRD.....ADFGL.....KSDVW.....
118572780 ......IIMKD.....VLSLL.....LVVLP.....FVHRD.....ADFGL.....KSDVW.....
118572787 ......IIMKD.....VLSLL.....LVVLP.....FVHRD.....ADFGL.....KSDVW.....
115636690 ......IMMKD.....VLSLI.....YVVLP.....FVHRD.....GDFGL.....RTDVW.....
119226186 ......VMMRG.....VLALI.....LIILP.....FVHRD.....ADFGL.....KSDVW.....
148887173 ......IIMKD.....VLSLL.....LVVLP.....FVHRD.....ADFGL.....KSDVW.....
119585440 ......LLMRG.....VLALI.....HVLLP.....FVHRD.....ADFGL.....KSDVW.....
148887171 ......IIMKD.....VLSLL.....LVVLP.....FVHRD.....ADFGL.....KSDVW.....
119603913 ......IIMKD.....VLSLL.....LVVLP.....FVHRD.....ADFGL.....KSDVW.....
119914682 ......LLMRR.....VLALI.....RVLLP.....FVHRD.....ADFGL.....KSDVW.....
125819093 ......LFMKA.....VLSLL.....LVILP.....FVHRD.....ADFGM.....KSDVW.....
125829807 ......ILMKG.....VLSLL.....LVVLP.....FVHRD.....ADFGM.....KSDVW.....
126336050 ......LLMRS.....VLSLI.....RVVLP.....FVHRD.....ADFGL.....KSDVW.....
126340641 ......IIMKD.....VLSLL.....LVVLP.....FVHRD.....ADFGL.....KSDVW.....
133778035 ......IIMKD.....VLSLL.....LVVLP.....FVHRD.....ADFGL.....KSDVW.....
133778041 ......IIMKD.....VLSLL.....LVVLP.....FVHRD.....ADFGL.....KSDVW.....
148681934 ......IIMKD.....VLSLL.....LVVLP.....FVHRD.....ADFGL.....KSDVW.....
149638791 ......IIMKD.....VLSLL.....LVVLP.....FVHRD.....ADFGL.....KSDVW.....
149728888 ......LLMRG.....VLALI.....CVLLP.....FVHRD.....ADFGL.....KSDVW.....
157118308 ......CLLAE.....IVKLL.....CLLFE.....FVHRD.....ADFGL.....ESDVW.....
109110605 ......ALMAE.....IVKLL.....CLLFE.....FVHRD.....ADFGL.....ESDVW.....
109110601 ......ALMAE.....IVKLL.....CLLFE.....FVHRD.....ADFGL.....ESDVW.....
109110603 ......ALMAE.....IVKLL.....CLLFE.....FVHRD.....ADFGL.....ESDVW.....
109110599 ......ALMAE.....IVKLL.....CLLFE.....FVHRD.....ADFGL.....ESDVW.....
110761061 ......CLLAE.....IVKLL.....CLLFE.....FVHRD.....ADFGL.....ESDVW.....
114626105 ......ALMAE.....IVKLL.....CLLFE.....FVHRD.....ADFGL.....ESDVW.....
114626117 ......ALMAE.....IVKLL.....CLLFE.....FVHRD.....ADFGL.....ESDVW.....
114626109 ......ALMAE.....IVKLL.....CLLFE.....FVHRD.....ADFGL.....ESDVW.....
114626113 ......ALMAE.....IVKLL.....CLLFE.....FVHRD.....ADFGL.....ESDVW.....
115691065 ......QMIAR.....IVKLL.....CLLLE.....FVHRD.....SDFGL.....ASDVW.....
119579470 ......ALMAE.....IVKLL.....CLLFE.....FVHRD.....ADFGL.....ESDVW.....
119579469 ......ALMAE.....IVKLL.....CLLFE.....FVHRD.....ADFGL.....ESDVW.....
119579471 ......ALMAE.....IVKLL.....CLLFE.....FVHRD.....ADFGL.....ESDVW.....
119900983 ......ALMAE.....IVKLL.....CLLFE.....FVHRD.....ADFGL.....ESDVW.....
119900985 ......ALMAE.....IVKLL.....CLLFE.....FVHRD.....ADFGL.....ESDVW.....
```

119900981	......ALMAE.....IVKLL.....CLLFE.....FVHRD.....ADFGL.....ESDVW......
126334859	......ALMAE.....IVRLL.....CLLFE.....FVHRD.....ADFGL.....ESDVW......
148670284	......ALMAE.....IVKLL.....CLLFE.....FVHRD.....ADFGL.....ESDVW......
148670285	......ALMAE.....IVKLL.....CLLFE.....FVHRD.....ADFGL.....ESDVW......
148670283	......ALMAE.....IVKLL.....CLLFE.....FVHRD.....ADFGL.....ESDVW......
148670281	......ALMAE.....IVKLL.....CLLFE.....FVHRD.....ADFGL.....ESDVW......
148670282	......ALMAE.....IVKLL.....CLLFE.....FVHRD.....ADFGL.....ESDVW......
149037125	......ALMAE.....IVKLL.....CLLFE.....FVHRD.....ADFGL.....ESDVW......
149037126	......ALMAE.....IVKLL.....CLLFE.....FVHRD.....ADFGL.....ESDVW......
149037127	......ALMAE.....IVKLL.....CLLFE.....FVHRD.....ADFGL.....ESDVW......
149412999	......ALMAE.....IVKLL.....CLLFE.....FVHRD.....ADFGL.....ESDIW......
149739681	......ALMAE.....IVKLL.....CLLFE.....FVHRD.....ADFGL.....ESDVW......
109074808	......KVLSY.....IVNLL.....LVITE.....CIHRD.....CDFGL.....ESDVW......
109079335	......KIMSH.....IVNLL.....LVITE.....CIHRD.....GDFGL.....QSDVW......
109079327	......KIMSH.....VVNLL.....YIITE.....CVHRD.....CDFGL.....LSDVW......
109079329	......KIMSH.....VVNLL.....YIITE.....CVHRD.....CDFGL.....LSDVW......
109079333	......KIMSH.....IVNLL.....LVITE.....CIHRD.....GDFGL.....QSDVW......
109120355	......KMMTQ.....IVNLL.....YLIFE.....CVHRD.....CDFGL.....KSDVW......
109495097	......KMMTH.....IVNLL.....YLIFE.....CVHRD.....CDFGL.....KSDVW......
110613446	......KIMTH.....IVNLL.....YIITE.....CVHRD.....CDFGL.....LSDVW......
114594738	......KVLSY.....IVNLL.....LVITE.....CIHRD.....CDFGL.....ESDVW......
114602769	......KIMSH.....VVNLL.....YIITE.....CVHRD.....CDFGL.....LSDVW......
114649291	......KMMTQ.....IVNLL.....YLIFE.....CVHRD.....CDFGL.....KSDVW......
116043978	......KIMSH.....VVNLL.....YIITE.....CVHRD.....CDFGL.....LSDVW......
116043976	......KIMSH.....VVNLL.....YIITE.....CVHRD.....CDFGL.....LSDVW......
116497195	......KMMTQ.....IVNLL.....YLIFE.....CVHRD.....CDFGL.....KSDVW......
117130316	......KVLSY.....IVNLL.....LVITE.....CIHRD.....CDFGL.....ESDVW......
118097472	......KIMSH.....IVNLL.....LVITE.....CIHRD.....CDFGL.....QSDVW......
118097470	......KIMSH.....IVNLL.....YIITE.....CVHRD.....CDFGL.....LSDVW......
119390020	......KIMSH.....IVNLL.....LVITE.....CIHRD.....GDFGL.....QSDVW......
119390010	......KIMSH.....IVNLL.....LVITE.....CIHRD.....GDFGL.....QSDVW......
119582150	......KIMSH.....VVNLL.....YIITE.....CVHRD.....CDFGL.....LSDVW......
119893893	......KVLSY.....IVNLL.....LVITE.....CIHRD.....CDFGL.....ESDVW......
119904649	......KMMTH.....IVNLL.....YLIFE.....CLHRD.....ADFGL.....KSDVW......
119923655	......KIMTH.....IVNLL.....YIITE.....CVHRD.....CDFGL.....LSDVW......
121114304	......KMMTQ.....IVNLL.....YLIFE.....CVHRD.....CDFGL.....KSDVW......
125803386	......KVLSH.....LLNLL.....FIIFS.....CIHRD.....CDFGL.....ESDVW......
126031591	......KIMSH.....IVNLL.....LVITE.....CIHRD.....GDFGL.....QSDVW......
126291209	......KIMSH.....IVNLL.....LVITE.....CIHRD.....GDFGL.....QSDVW......
126327599	......KMMTH.....IVNLL.....YLIFE.....CVHRD.....CDFGL.....KSDVW......
126331629	......KIMTH.....IVNLL.....YIITE.....CVHRD.....CDFGL.....LSDVW......
126331737	......KVLSY.....IVNLL.....LVITK.....CIHRD.....CDFGL.....ESDVW......
156717500	......KIMTH.....IVNLL.....YIITE.....CVHRD.....CDFGL.....LSDVW......
134085174	......KIMTH.....IVNLL.....YIITE.....CVHRD.....CDFGL.....LSDVW......
148673885	......KMMTH.....IVNLL.....YLIFE.....CVHRD.....CDFGL.....KSDVW......
148673886	......KMMTH.....IVNLL.....YLIFE.....CVHRD.....CDFGL.....KSDVW......
148677841	......KIMSH.....VVNLL.....YIITE.....CVHRD.....CDFGL.....LSDVW......
148677842	......KIMSH.....VVNLL.....YIITE.....CVHRD.....CDFGL.....LSDVW......
148677840	......KIMSH.....VVNLL.....YIITE.....CVHRD.....CDFGL.....LSDVW......
148705936	......KIMTH.....IVNLL.....YIITE.....CVHRD.....CDFGL.....LSDVW......
148726416	......KIMTH.....IVNLL.....YIITE.....CVHRD.....CDFGL.....LSDVW......
148726415	......KIMTH.....IVNLL.....YIITE.....CVHRD.....CDFGL.....LSDVW......
149034842	......KMMTH.....IVNLL.....YLIFE.....CVHRD.....CDFGL.....KSDVW......
149064384	......KIMSH.....VVNLL.....YIITE.....CVHRD.....CDFGL.....LSDVW......
149064385	......KIMSH.....IVNLL.....LVITE.....CIHRD.....GDFGL.....QSDVW......
149599038	......KMMTH.....IVNLL.....YLIFE.....CVHRD.....CDFGL.....KSDVW......
149634850	......KVLSY.....IVNLL.....LVITE.....CIHRD.....CDFGL.....ESDVW......
149702964	......KIMTH.....IVNLL.....YIITE.....CVHRD.....CDFGL.....LSDVW......
149702961	......KVLSY.....IVNLL.....LVITE.....CIHRD.....CDFGL.....ESDVW......
149726184	......KIMSH.....IVNLL.....LVITE.....CIHRD.....GDFGL.....QSDVW......
149726738	......KIMSH.....VVNLL.....YIITE.....CVHRD.....CDFGL.....LSDVW......
149730114	......KMMTH.....IVNLL.....YLIFE.....CVHRD.....CDFGL.....KSDVW......
157119750	......AIMKE.....LVQLI.....YIITE.....FIHRD.....ADFGL.....KSDVW......
109019222	......QILRG.....ILQFL.....YIVTE.....FIHRD.....ADFGL.....KSDVW......
109019224	......QILRG.....ILQFL.....YIVTE.....FIHRD.....ADFGL.....KSDVW......
109019226	......QILRG.....ILQFL.....YIVTE.....FIHRD.....ADFGL.....KSDVW......
109019220	......QILRG.....ILQFL.....YIVTE.....FIHRD.....ADFGL.....KSDVW......
109157754	......AVMKE.....LVQLL.....YIITE.....FIHRD.....ADFGL.....KSDVW......
109157762	......AVMKE.....LVQLL.....YIITE.....FIHRD.....ADFGL.....KSDVW......
109468008	......AVMKE.....LVQLL.....YIITE.....FIHRD.....ADFGL.....KSDVW......
109468010	......AVMKE.....LVQLL.....YIITE.....FIHRD.....ADFGL.....KSDVW......
109469721	......AVMKE.....LVQLL.....YIITE.....FIHRD.....ADFGL.....KSDVW......
114568177	......AVMKE.....LVQLL.....YIVTE.....FIHRD.....ADFGL.....KSDVW......

```
114568179    ......AVMKE.....LVQLL.....YIVTE.....FIHRD.....ADFGL.....KSDVW......
114568175    ......AVMKE.....LVQLL.....YIVTE.....FIHRD.....ADFGL.....KSDVW......
114627222    ......AVMKE.....LVQLL.....YIITE.....FIHRD.....ADFGL.....KSDVW......
114627224    ......AVMKE.....LVQLL.....YIITE.....FIHRD.....ADFGL.....KSDVW......
114794378    ......AVMKE.....LVQLL.....YIITE.....FIHRD.....ADFGL.....KSDVW......
116875856    ......AVMKE.....LVQLL.....YIVTE.....FIHRD.....ADFGL.....KSDVW......
118094194    ......AVMKE.....LVQLL.....YIVTE.....FIHRD.....ADFGL.....KSDVW......
118099341    ......AVMKE.....LVQLL.....YIITE.....FIHRD.....ADFGL.....KSDVW......
118099343    ......AVMKE.....LVQLL.....YIITE.....FIHRD.....ADFGL.....KSDVW......
119389607    ......AVMKE.....LVQLL.....YIITE.....FIHRD.....ADFGL.....KSDVW......
153266757    ......AVMKE.....LVQLL.....YIVTE.....FIHRD.....ADFGL.....KSDVW......
119608353    ......AVMKE.....LVQLL.....YIITE.....FIHRD.....ADFGL.....KSDVW......
119908618    ......AVMKE.....LVQLL.....YIVTE.....FIHRD.....ADFGL.....KSDVW......
125817700    ......AVMKE.....LVQLL.....YIITE.....FIHRD.....ADFGL.....KSDVW......
125817698    ......AVMKE.....LVQLL.....YIITE.....FIHRD.....ADFGL.....KSDVW......
170015995    ......SVMKE.....LVQLL.....YIVTE.....FIHRD.....ADFGL.....KSDVW......
126030689    ......AVMKE.....LVQLL.....YIITE.....FIHRD.....ADFGL.....KSDVW......
126030694    ......AVMKE.....LVQLL.....YIITE.....FIHRD.....ADFGL.....KSDVW......
126030685    ......AVMKE.....LVQLL.....YIITE.....FIHRD.....ADFGL.....KSDVW......
126306282    ......AVMKE.....LVQLL.....YIVTE.....FIHRD.....ADFGL.....KSDVW......
126306280    ......AVMKE.....LVQLL.....YIVTE.....FIHRD.....ADFGL.....KSDVW......
126306278    ......AVMKE.....LVQLL.....YIVTE.....FIHRD.....ADFGL.....KSDVW......
148707429    ......AVMKE.....LVQLL.....YIVTE.....FIHRD.....ADFGL.....KSDVW......
149039047    ......AVMKE.....LVQLL.....YIITE.....FIHRD.....ADFGL.....KSDVW......
157821685    ......AVMKE.....LVQLL.....YIVTE.....FIHRD.....ADFGL.....KSDVW......
149241245    ......AVMKE.....LVQLL.....YIITE.....FIHRD.....ADFGL.....KSDVW......
149410314    ......AVMKE.....LVQLL.....YIITE.....FIHRD.....ADFGL.....KSDVW......
149636169    ......AVMKE.....LVQLL.....YIVTE.....FIHRD.....ADFGL.....KSDVW......
149707930    ......AVMKE.....LVQLL.....YIVTE.....FIHRD.....ADFGL.....KSDVW......
149707926    ......AVMKE.....LVQLL.....YIVTE.....FIHRD.....ADFGL.....KSDVW......
55662017     ......AVMKE.....LVQLL.....YIVTE.....FIHRD.....ADFGL.....KSDVW......
157115161    ......QLLQE.....VIRLL.....LLIIE.....LVHRD.....SDFGL.....KSDVW......
110767634    ......QLLKE.....VIRLL.....YLIIE.....LVHRD.....SDFGL.....KSDVW......
114794793    ......NVLKQ.....VIKLY.....LLIVE.....LVHRD.....SDFGL.....QSDVW......
114794791    ......NVLKQ.....VIKLY.....LLIVE.....LVHRD.....SDFGL.....QSDVW......
114794789    ......NVLKQ.....VIKLY.....LLIVE.....LVHRD.....SDFGL.....QSDVW......
119606990    ......NVLKQ.....VIKLY.....LLIVE.....LVHRD.....SDFGL.....QSDVW......
119606985    ......NVLKQ.....VIKLY.....LLIVE.....LVHRD.....SDFGL.....QSDVW......
119606984    ......NVLKQ.....VIKLY.....LLIVE.....LVHRD.....SDFGL.....QSDVW......
148667176    ......NLLKQ.....VIKLY.....LLIVE.....LVHRD.....SDFGL.....QSDVW......
158534062    ......NLLKQ.....VIKLY.....LLIVE.....LVHRD.....SDFGL.....QSDVW......
158534064    ......NLLKQ.....VIKLY.....LLIVE.....LVHRD.....SDFGL.....QSDVW......
149632184    ......NLLKQ.....VIKLY.....YLIVE.....LVHRD.....SDFGL.....QSDVW......
157126057    ......ELISD.....IVCIL.....CMLFE.....YVHRD.....SDFGL.....ESDVW......
109005871    ......SLMAE.....IVCLL.....CMLFE.....FVHKD.....SDLGL.....DSDIW......
109112284    ......MLRAR.....IVCLL.....SMIFS.....VVHKD.....SDLGL.....DSDIW......
157822115    ......SLMAE.....IVCLL.....CMLFE.....FVHKD.....SDLGL.....DSDIW......
157823843    ......MLRAR.....IVCLL.....SMVFS.....VVHKD.....SDLGL.....DSDIW......
110748871    ......DIMST.....ILSLK.....SMVFE.....FVHRD.....ADFGM.....ESDVW......
110758504    ......DLMTD.....IICLL.....CMLFE.....YVHRD.....SDFGL.....ESDVW......
114557175    ......SLMAE.....IVCLL.....CMLFE.....FVHKD.....SDLGL.....DSDIW......
114625503    ......MLRAR.....IVCLL.....SMIFS.....VVHKD.....SDLGL.....DSDIW......
115921052    ......SVMAS.....IITLL.....CMLFE.....FVHRD.....TDFGL.....ESDIW......
123959766    ......MMRSR.....IVCLL.....SMIFS.....VVHKD.....SDLGL.....DSDIW......
119583202    ......MLRAR.....VVCLL.....SMIFS.....VVHKD.....SDLGL.....DSDIW......
119890108    ......SLMAE.....IVCLL.....CMLFE.....FVHKD.....SDLGL.....DSDIW......
119626962    ......SLMAE.....IVCLL.....CMLFE.....FVHKD.....SDLGL.....DSDIW......
120660404    ......MLRAR.....VVCLL.....SMIFS.....VVHKD.....SDLGL.....DSDIW......
121582134    ......EAWSK.....IITIV.....CVLYE.....IVHGD.....TDVAL.....ETDIW......
124504273    ......SLMAE.....IVCLL.....CMLFE.....FVHKD.....SDLGL.....DSDIW......
125818690    ......SVLAE.....VVCLL.....CMLFE.....FIHKD.....SDLGL.....DSDVW......
125826902    ......MLRSR.....IVCLL.....SMIFS.....VVHKD.....LDLGL.....DSDIW......
126316162    ......MMRSR.....IVCLL.....SMIFG.....VVHKD.....SDLGL.....DSDIW......
148709174    ......MLRAR.....IVCLL.....SMIFS.....VVHKD.....SDLGL.....DSDIW......
149507357    ......SLTAE.....IVCLL.....CMLFE.....FVHKD.....SDLGL.....DSDIW......
149709772    ......SLMAE.....IVCLL.....CMLFE.....FVHKD.....SDLGL.....DSDIW......
149758051    ......MLRAR.....IVCLL.....SMIFS.....VVHKD.....SDLGL.....DSDIW......
157123527    ......STLCG.....ILPVA.....KVAYP.....VLHKD.....CDNAL.....SSDIW......
157130072    ......MSLYG.....ILSVL.....PFLLY.....VIHKD.....ADNSL.....ASDTW......
109049440    ......CKLRG.....LLPIT.....MVILP.....VIHKD.....TDNAL.....ASDVW......
```

```
110681702      ......CKLRG......LLPIT......MVVLP......VIHRD......TDNAL......ASDVW......
110760450      ......SQLAG......IASLA......LLAYT......LLHRD......ADTAL......ATDVW......
115732965      ......CLLQG......ILPLL......FILEA......LVHKD......TDNAL......ASDVW......
118094904      ......CKLRG......LLPIT......MVLLP......VIHKD......TDNAL......ASDVW......
119886983      ......CKLRG......LLPIT......MVILP......VIHKD......TDNAL......ASDVW......
119599561      ......CKLRG......LLPIT......MVILP......VIHKD......TDNAL......ASDVW......
139948989      ......CKLRG......LLPIS......MVLLP......VIHKD......TDNAL......ASDVW......
126326011      ......CKLRG......LLPIT......MVLLP......VIHKD......TDNAL......ASDVW......
148689113      ......CKLRG......LLPIT......MVVLP......VIHRD......TDNAL......ASDVW......
149018754      ......CKLRG......LLPIT......MVISP......VIHKD......TDNAL......ASDVW......
157106088      ......QAMHT......LVRLH......MMVTE......FLHRD......GDFGL......ASDTW......
157128141      ......AIMHS......IVRLY......MLVTE......LIHRD......SDFGL......ASDVW......
157124162      ......QAMHT......LVRLH......MMVTE......FLHRD......GDFGL......ASDTW......
109054420      ......NAMHS......LIRLY......KMVTE......FIHRD......GDFGL......ASDTW......
109054417      ......NAMHS......LIRLY......KMVTE......FIHRD......GDFGL......ASDTW......
109113088      ......SVMMN......VLRLH......QMVME......LVHRD......ADFGL......ASDVW......
157822447      ......SVMMK......VLRLH......QMVME......LVHRD......ADFGL......ASDVW......
109521356      ......NAMHS......LIRLY......KMVTE......FIHRD......GDFGL......ASDTW......
164420757      ......NAMHS......LIRLY......KTVTE......FIHRD......GDFGL......ASDTW......
109729623      ......NAMHS......LIRLY......KMVTE......FIHRD......GDFGL......ASDTW......
110759083      ......QVMHT......LIRLY......MMVTE......FLHRD......GDFGL......ASDVW......
114666152      ......SVMMN......VLRLH......QMVME......LVHRD......ADFGL......ASDVW......
114666154      ......SVMMN......VLRLH......QMVME......LVHRD......ADFGL......ASDVW......
115637393      ......AIMHS......LVKLY......MLVTE......IIHRD......SDFGL......ASDVW......
115958895      ......AIMHS......LVKLY......MLVTE......IIHRD......SDFGL......ASDVW......
56549666f      ......NAMHS......LIRLY......KMVTE......FIHRD......GDFGL......ASDTW......
58331191f      ......NAMHS......LIRLY......KMVTE......FIHRD......GDFGL......ASDTW......
116242821      ......SVMMN......VLRLH......QMVME......LVHRD......ADFGL......ASDVW......
121583968      ......TSMQS......IIHLY......KMVTE......FIHRD......GDFGL......ASDVW......
119570720      ......NAMHS......LIRLY......KMVTE......FIHRD......GDFGL......ASDTW......
119570719      ......NAMHS......LIRLY......KMVTE......FIHRD......GDFGL......ASDTW......
119570721      ......NAMHS......LIRLY......KMVTE......FIHRD......GDFGL......ASDTW......
119610610      ......SVMMN......VLRLH......QMVME......LVHRD......ADFGL......ASDVW......
119610609      ......SVMMN......VLRLH......QMVME......LVHRD......ADFGL......ASDVW......
125853655      ......NAMHS......LIRLY......KMVTE......FIHRD......GDFGL......ASDTW......
126325979      ......NAMHS......LIRLY......KMVTE......FIHRD......GDFGL......ASDTW......
156717326      ......NAMHS......LIRLY......KMVTE......FIHLD......GDFGL......ASDTW......
148665389      ......NAMHS......LIRLY......KMVTE......FIHRD......GDFGL......ASDTW......
158711692      ......NAMHS......LIRLY......KMVTE......FIHRD......GDFGL......ASDTW......
149724249      ......SIMMN......VLRLH......QMVME......LVHRD......ADFGL......ASDVW......
157116740      ......HLMSN......ILKLI......YIVME......FVHRD......GDFGL......QSDIW......
109072890      ......HLMSK......ILKQL......YIILE......FIHRD......GDFGL......QSDVW......
110749902      ......RLMSH......VLRLL......LLVLE......FVHRD......GDFGL......QSDVW......
114609055      ......HLMSK......ILKQL......YIILE......FIHRD......GDFGL......QSDVW......
14424434       ......QLMSN......IVCLV......SLIME......FVHRD......GDFGL......QSDVW......
119568593      ......HLMSK......ILKQL......YIILE......FIHRD......GDFGL......QSDVW......
119568594      ......HLMSK......ILKQL......YIILE......FIHRD......GDFGL......QSDVW......
125841522      ......HLMSQ......ILRLL......YLILE......FVHRD......GDFGL......YSDVW......
148673127      ......HLMSK......ILKQL......YIILE......FIHRD......GDFGL......QSDVW......
148673126      ......HLMSK......ILKQL......YIILE......FIHRD......GDFGL......QSDVW......
149038653      ......HLMSK......ILKQL......YIILE......FIHRD......GDFGL......QSDVW......
149615041      ......HLMSK......ILKQL......YLILE......FIHRD......GDFGL......RSDVW......
149722908      ......HLMSK......ILRQL......YIILE......FIHRD......GDFGL......QSDVW......
157106710      ......AIMKK......LVALY......YIVQE......LIHRD......CDFGL......KSDVW......
157129304      ......QIMKK......LIQLY......YIITE......YIHRD......ADFGL......KSDVW......
109000043      ......QVMKL......LVQLY......YIVTE......YIHRD......ADFGL......KSDVW......
109000046      ......QVMKL......LVQLY......YIVTE......YIHRD......ADFGL......KSDVW......
109000025      ......QVMKL......LVQLY......YIVTE......YIHRD......ADFGL......KSDVW......
109000034      ......QVMKL......LVQLY......YIVTE......YIHRD......ADFGL......KSDVW......
109000040      ......QVMKL......LVQLY......YIVTE......YIHRD......ADFGL......KSDVW......
109000037      ......QVMKL......LVQLY......YIVTE......YIHRD......ADFGL......KSDVW......
109072957      ......QIMKN......LIQLY......YIITE......YIHRD......ADFGL......KSDVW......
109072955      ......QIMKN......LIQLY......YIITE......YIHRD......ADFGL......KSDVW......
109073003      ......QIMKK......LVQLY......YIVTE......YIHRD......ADFGL......KSDVW......
109085588      ......NVMKA......LVRLY......YIVTE......SIHRD......ADFGL......KADVW......
109085590      ......NVMKA......LVRLY......YIVTE......SIHRD......ADFGL......KADVW......
109085592      ......NVMKA......LVRLY......YIVTE......SIHRD......ADFGL......KADVW......
109085594      ......NVMKA......LVRLY......YIVTE......SIHRD......ADFGL......KADVW......
109085596      ......NVMKA......LVRLY......YIVTE......SIHRD......ADFGL......KADVW......
109085598      ......NVMKA......LVRLY......YIVTE......SIHRD......ADFGL......KADVW......
```

109086465	......NLMKT.....LVRLY.....YIITE.....YIHRD.....ADFGL.....KSDVW.....
109091342	......QTLKG.....LIRLH.....YIVTE.....IVHRD.....ADFGL.....KSDVW.....
109092120	......QVMKK.....LVQLY.....YIVTE.....YVHRD.....ADFGL.....KSDVW.....
109121713	......QIMKK.....LVPLY.....YIVTE.....YIHRD.....ADFGL.....KSDVW.....
109121705	......QIMKK.....LVPLY.....YIVTE.....YIHRD.....ADFGL.....KSDVW.....
109121715	......QIMKK.....LVPLY.....YIVTE.....YIHRD.....ADFGL.....KSDVW.....
109475545	......NLMKQ.....LVRLY.....YIITE.....YIHRD.....ADFGL.....KSDVW.....
110591271	......QIMKK.....LVQLY.....YIVTE.....YIHRD.....ADFGL.....KSDVW.....
110765971	......QIMKK.....LIQLY.....YIITE.....YIHRD.....ADFGL.....KSDVW.....
112419737	......AVMKR.....LIQLF.....YIVSE.....FIHRD.....GDFGL.....KSDVW.....
112419739	......AVMKK.....LIRLY.....YIITE.....FIHRD.....ADFGL.....KSDVW.....
112419733	......AVMKK.....LIQLF.....YIITE.....FIHRD.....GDFGL.....KSDVW.....
114608995	......QIMKN.....LIQLY.....YIITE.....YIHRD.....ADFGL.....KSDVW.....
114608997	......QIMKN.....LIQLY.....YIITE.....YIHRD.....ADFGL.....KSDVW.....
114608944	......QIMKK.....LVQLY.....YIVTE.....YIHRD.....ADFGL.....KSDVW.....
114608946	......QIMKK.....LVQLY.....YIVTE.....YIHRD.....ADFGL.....KSDVW.....
114672463	......QIMKK.....LVPLY.....YIVTE.....YIHRD.....ADFGL.....KSDVW.....
114672461	......QIMKK.....LVPLY.....YIVTE.....YIHRD.....ADFGL.....KSDVW.....
114672459	......QIMKK.....LVPLY.....YIVTE.....YIHRD.....ADFGL.....KSDVW.....
114681916	......QVMKK.....LVQLY.....YIVTE.....YVHRD.....ADFGL.....KSDVW.....
114681918	......QVMKK.....LVQLY.....YIVTE.....YVHRD.....ADFGL.....KSDVW.....
114681908	......QVMKK.....LVQLY.....YIVTE.....YVHRD.....ADFGL.....KSDVW.....
114681910	......QVMKK.....LVQLY.....YIVTE.....YVHRD.....ADFGL.....KSDVW.....
114681752	......NVMKT.....LVKLH.....YIITE.....YIHRD.....ADFGL.....KSDVW.....
114681912	......QVMKK.....LVQLY.....YIVTE.....YVHRD.....ADFGL.....KSDVW.....
114681914	......QVMKK.....LVQLY.....YIVTE.....YVHRD.....ADFGL.....KSDVW.....
114683138	......QTLKG.....LIRLH.....YIVTE.....VVHRD.....ADFGL.....KSDVW.....
114794401	......NVMKT.....LVKLH.....YIITE.....YIHRD.....ADFGL.....KSDVW.....
115496864	......QIMKR.....LISLF.....YIITE.....SIHRD.....ADFGL.....KSDVW.....
115616565	......NLMKE.....LVNLY.....YIITE.....YVHRD.....ADFGL.....KSDVW.....
115682433	......NVMKK.....IVQLY.....YIVTE.....VVHRD.....ADFGR.....KSDVW.....
115687298	......NIMKK.....LVALL.....YIVTE.....FVHRD.....ADFGL.....KSDVW.....
115943912	......NIMKK.....LVALL.....YIVTE.....FVHRD.....ADFGL.....KSDVW.....
116283984	......NLMKT.....LVRLY.....YIITE.....YIHRD.....ADFGL.....KSDVW.....
116284012	......NVMKA.....LVRLY.....YIVTE.....SIHRD.....ADFGL.....KADVW.....
118088653	......QIMKN.....LIQLY.....YIVTE.....YIHRD.....ADFGL.....KSDVW.....
118089189	......NLMKK.....LVRLY.....YIVTE.....SIHRD.....ADFGL.....KSDVW.....
118100777	......NLMKS.....LVRLH.....YIITE.....YIHRD.....ADFGL.....KSDVW.....
118100717	......QNLKR.....LIQLH.....YIITE.....IVHRD.....ADFGL.....KSDVW.....
118137342	......QVMKK.....LVQLY.....YIVTE.....YVHRD.....ADFGL.....KSDVW.....
119508804	......QIMKK.....LVPLY.....YIVTE.....YIHRD.....ADFGL.....KSDVW.....
119389672	......QVMKK.....LVQLY.....YIVTE.....YVHRD.....ADFGL.....KSDVW.....
119568665	......QIMKK.....LVQLY.....YIVTE.....YIHRD.....ADFGL.....KSDVW.....
119568669	......QIMKK.....LVQLY.....YIVTE.....YIHRD.....ADFGL.....KSDVW.....
119595669	......QAMKK.....ILALY.....YIITE.....YIHRD.....GDFGL.....KSDVW.....
119595668	......QAMKK.....ILALY.....YIITE.....YIHRD.....GDFGL.....KSDVW.....
119607172	......NLMKT.....LVRLY.....YIITE.....YIHRD.....ADFGL.....KSDVW.....
119901215	......QIMKN.....LIQLY.....YIITE.....YIHRD.....ADFGL.....KSDVW.....
119905607	......QTLKS.....LIRLH.....YIVTE.....IVHRD.....ADFGL.....KSDVW.....
147902372	......NLMKQ.....LVRLN.....YIVTE.....YIHRD.....ADFGL.....KSDVW.....
122891858	......QIMKK.....LVQLY.....YIVTE.....YIHRD.....ADFGL.....KSDVW.....
125825232	......QILKK.....LIALF.....YIITE.....SIHRD.....ADLGL.....KSDVW.....
125845693	......QIMKK.....LIQLY.....YIITE.....YIHRD.....ADFGL.....KSDVW.....
125851621	......NLMKS.....LVRLN.....YIITE.....YIHRD.....ADFGL.....KSDVW.....
126291286	......QVMKK.....LVQLY.....YIVTE.....YVHRD.....ADFGL.....KSDVW.....
126291289	......QVMKK.....LVQLY.....YIVTE.....YVHRD.....ADFGL.....KSDVW.....
126293861	......NLMKT.....LVKLH.....YIITE.....YIHRD.....ADFGL.....KSDVW.....
126303011	......EAMKK.....ILSLY.....YIITE.....YVHRD.....GDFGL.....KSDVW.....
126303009	......QNLKS.....LIRLH.....YIVTE.....IVHRD.....ADFGL.....KSDVW.....
126310438	......QIMKK.....LVQLY.....YIVTE.....YIHRD.....ADFGL.....KSDVW.....
126310434	......QIMKK.....LVQLY.....YIVTE.....YIHRD.....ADFGL.....KSDVW.....
126303989	......NLMKT.....LVRLY.....YIVTE.....SIHRD.....ADFGL.....KSDVW.....
126310462	......QIMKN.....LIQLY.....YIITE.....YIHRD.....ADFGL.....KSDVW.....
126321787	......QIMKK.....LVPLY.....YIVTE.....YIHRD.....ADFGL.....KSDVW.....
126330231	......NLMKQ.....LVRLY.....YIITE.....YIHRD.....ADFGL.....KSDVW.....
127802117	......NVMKT.....LVKLH.....YIITE.....YTHRD.....ADFGL.....KSDVW.....
134104465	......QVMKK.....LVQLY.....YIVTE.....YVHRD.....ADFGL.....KSDVW.....
134105148	......NLMKQ.....LVRLY.....YIITE.....YIHRD.....ADFGL.....KSDVW.....

```
134105147   ......NLMKQ......LVRLY......YIITE......YIHRD......ADFGL......KSDVW......
134105153   ......NLMKQ......LVRLY......YIITE......YIHRD......ADFGL......KSDVW......
134105145   ......NLMKQ......LVRLY......YIITE......YIHRD......ADFGL......KSDVW......
143811392   ......QIMKK......LVQLY......YIVTE......YIHRD......ADFGL......KSDVW......
145580052   ......QVMKK......LVQLY......YIVTE......YVHRD......ADFGL......KSDVW......
145586946   ......NLMKT......LVRLF......YIITE......YIHRD......ADFGL......KSDVW......
145586944   ......NLMKA......LVRLF......YIITE......YIHRD......ADFGL......KSDVW......
146232051   ......KTLTK......VIHLL......LIITE......IVHRD......ADFGL......KTDVW......
148672975   ......QIMKK......LVQLY......YIVTE......YIHRD......ADFGL......KSDVW......
148675448   ......EALKS......LIRLH......YIVTE......VVHRD......ADFGL......KSDVW......
148672946   ......QIMKS......LIQLY......YIITE......YIHRD......ADFGL......KSDVW......
148705449   ......QIMKK......LVPLY......YIVTE......YIHRD......ADFGL......KSDVW......
148704117   ......NVMKT......LVRLY......YIVTE......SIHRD......ADFGL......KADVW......
148705448   ......QIMKK......LVPLY......YIVTE......YIHRD......ADFGL......KSDVW......
148726499   ......QIMKR......LVQLY......YIITE......YIHRD......ADFGL......KSDVW......
149030991   ......NLMKT......LVKLH......FIVTE......YIHRD......ADFGL......KSDVW......
149043104   ......QVMKK......LVQLY......YIVTE......YVHRD......ADFGL......KSDVW......
149411118   ......NLMKT......LVRLY......YIITE......YIHRD......ADFGL......KSDVW......
149411122   ......NLMKT......LVRLY......YIITE......YIHRD......ADFGL......KSDVW......
149411120   ......NLMKT......LVRLY......YIITE......YIHRD......ADFGL......KSDVW......
149427478   ......QIMKQ......LVQLY......YIVTE......YIHRD......ADFGL......KSDVW......
149635250   ......QIMKK......LVPLY......YIVTE......YIHRD......ADFGL......KSDVW......
149635681   ......QIMKK......LVQLY......YIVTE......YIHRD......ADFGL......KSDVW......
149636054   ......QNLKS......LIRLH......YIVTE......IVHRD......ADFGL......KSDVW......
149636052   ......QVMKE......ILALY......YIITE......YIHRD......GDFGL......KSDVW......
149694184   ......QVMKL......LVQLY......YIVTE......YIHRD......ADFGL......KSDVW......
149694740   ......NLMKQ......LVRLY......YIITE......YIHRD......ADFGL......KSDVW......
149698297   ......NLMKT......LVRLY......YIVTE......SIHRD......ADFGL......KADVW......
149721012   ......QIMKK......LVPLY......YIVTE......YIHRD......ADFGL......KSDVW......
149721393   ......NLMKT......LVRLY......YIITE......YIHRD......ADFGL......KSDVW......
149721391   ......NLMKT......LVRLY......YIITE......YIHRD......ADFGL......KSDVW......
149723117   ......QIMKN......LIQLY......YIITE......YIHRD......ADFGL......KSDVW......
149733631   ......QVMKK......LVQLY......YIVTE......YVHRD......ADFGL......KSDVW......
149734289   ......QAMKQ......ILALY......YIVTE......YIHRD......GDFGL......KSDVW......
149734291   ......QTLKS......LIRLH......YIVTE......IVHRD......ADFGL......KSDVW......
157119639   ......SVMIR......IVKLI......MMVEE......FVHRD......SDFGL......ASDVW......
109103910   ......QIMHQ......IVRLI......MLVME......FVHRD......SDFGL......RSDVW......
109103908   ......QIMHQ......IVRLI......MLVME......FVHRD......SDFGL......RSDVW......
109103912   ......QIMHQ......IVRLI......MLVME......FVHRD......SDFGL......RSDVW......
109112266   ......NVMQQ......IVRMI......MLVME......FVHRD......SDFGL......KSDVW......
109112262   ......NVMQQ......IVRMI......MLVME......FVHRD......SDFGL......KSDVW......
109112264   ......NVMQQ......IVRMI......MLVME......FVHRD......SDFGL......KSDVW......
109112260   ......NVMQQ......IVRMI......MLVME......FVHRD......SDFGL......KSDVW......
109112256   ......NVMQQ......IVRMI......MLVME......FVHRD......SDFGL......KSDVW......
114579056   ......QIMHQ......IVRLI......MLVME......FVHRD......SDFGL......RSDVW......
114625493   ......NVMQQ......IVRMI......MLVME......FVHRD......SDFGL......KSDVW......
115738266   ......KVMCG......IVKLM......MLVQE......FVHRD......SDFGL......SSDVW......
115924184   ......ELMAG......IVRMI......-----......FVHRD......SDFGM......KSDVW......
118103133   ......QIMHQ......IVRMI......MLVME......FVHRD......SDFGL......KSDVW......
119622324   ......QIMHQ......IVRLI......MLVME......FVHRD......SDFGL......RSDVW......
126316168   ......NVMQR......IVRMI......MLVME......FVHRD......SDFGL......KSDVW......
126323793   ......HIMHQ......IVRII......MLVME......FVHRD......SDFGL......RSDVW......
149044990   ......NVMQQ......IVRMI......MLVME......FVHRD......SDFGL......KSDVW......
149044986   ......NVMQQ......IVRMI......MLVME......FVHRD......SDFGL......KSDVW......
149758047   ......NVMQQ......IVRMI......MLVME......FVHRD......SDFGL......KSDVW......
157103777   ......AIMAK......IVHLI......FIVLE......FIHRD......ADFGM......KTDVW......
109080787   ......LIIRC......QGWKA......-----......FIHRD......GDFGM......KTDSW......
149023031   ......LIISK......IVRCV......LILLE......FIHRD......GDFGM......KTDSW......
109479030   ......LIISK......IVRCI......FILLE......FIHRD......GDFGM......KTDTW......
110347475   ......LIISK......IVRCI......FILLE......FIHRD......GDFGM......KTDTW......
110750737   ......LIMSK......IVHFI......YIVLE......FIHRD......ADFGM......KTDVW......
114656474   ......LIISK......IVRCV......LILLE......FIHRD......GDFGM......KTDSW......
114656472   ......LIISK......IVRCV......LILLE......FIHRD......GDFGM......KTDSW......
118087613   ......LIISK......IVRCI......FILLE......FIHRD......GDFGM......KTDTW......
118091617   ......LIISK......IVQCI......FILLE......FIHRD......GDFGM......KTDTW......
119612904   ......LIISK......IVRCV......LILLE......FIHRD......GDFGM......KTDSW......
119612907   ......LIISK......IVRCV......LILLE......FIHRD......GDFGM......KTDSW......
119620912   ......LIISK......IVRCI......FILLE......FIHRD......GDFGM......KTDTW......
146328566   ......LIISK......IVRCI......FILLE......FIHRD......GDFGM......KTDTW......
119903780   ......LIISK......IVRCI......FILLE......FIHRD......GDFGM......KTDTW......
```

```
159032530    ......LIISK......IVRCV......LILLE......FIHRD......GDFGM......KTDSW......
159032532    ......LIISK......IVRCV......LILLE......FIHRD......GDFGM......KTDSW......
125842088    ......LIMSK......IVRCI......FILLE......FIHRD......GDFGM......KTDTW......
125842196    ......LIMSK......IVRCI......FILLE......FIHRD......GDFGM......KTDTW......
125842613    ......LIISK......IVRCI......FILLE......FIHRD......GDFGM......KTDTW......
126278417    ......LIISK......IVKCV......LILLE......FIHRD......GDFGM......KTDSW......
126303631    ......LIISK......IVRCI......FILLE......FIHRD......GDFGM......KTDTW......
29029632f    ......LIISK......IVRCI......FILLE......FIHRD......GDFGM......KTDTW......
148696023    ......LIISK......IVRCV......LILLE......FIHRD......GDFGM......KTDSW......
149728028    ......LIISK......IVRCI......FILLE......FIHRD......GDFGM......KTDTW......
157103605    ......KVMTK......LVQLY......YITTE......YIHRD......ADFGL......KSDVW......
109074187    ......KVMMK......LVQLY......YIVTE......YIHRD......SDFGM......KSDVW......
109074192    ......KVMMK......LVQLY......YIVTE......FIHRD......SDFGM......KSDVW......
109079556    ......EVMMK......LVQLY......CLVFE......VIHRD......SDFGM......KSDVW......
109129993    ......QTMTK......LVKFY......YIVTE......FIHRD......SDFGM......KSDVW......
109129997    ......QTMTK......LVKFY......YIVTE......FIHRD......SDFGM......KSDVW......
109129995    ......QTMTK......LVKFY......YIVTE......FIHRD......SDFGM......KSDVW......
157818821    ......EVMMK......LVQLY......CLVFE......VIHRD......SDFGM......KSDVW......
109500475    ......KVMMK......LVQLY......YIVTE......FIHRD......SDFGM......KSDVW......
109500471    ......KVMMK......LVQLY......YIVTE......FIHRD......SDFGM......KSDVW......
109500477    ......KVMMK......LVQLY......YIVTE......FIHRD......SDFGM......KSDVW......
109500473    ......KVMMK......LVQLY......YIVTE......FIHRD......SDFGM......KSDVW......
157824055    ......QTMMK......LVKFY......YIVTE......FIHRD......SDFGM......KSDVW......
110764017    ......KVMTK......LVQLY......YIVTE......YIHRD......ADFGL......KSDVW......
111306647    ......KVMMK......LVQLY......YIVTE......YIHRD......SDFGM......KSDVW......
114594882    ......KVMMK......LVQLY......YIVTE......FIHRD......SDFGM......KSDVW......
114603096    ......EVMMK......LVQLY......CLVFE......VIHRD......SDFGM......KSDVW......
114689455    ......KVMMN......LVQLY......FIITE......FLHRD......SDFGL......KSDIW......
114689449    ......KVMMN......LVQLY......FIITE......FLHRD......SDFGL......KSDIW......
114689451    ......KVMMN......LVQLY......FIITE......FLHRD......SDFGL......KSDIW......
114689447    ......KVMMN......LVQLY......FIITE......FLHRD......SDFGL......KSDIW......
114689441    ......KVMMN......LVQLY......FIITE......FLHRD......SDFGL......KSDIW......
114689445    ......KVMMN......LVQLY......FIITE......FLHRD......SDFGL......KSDIW......
148596974    ......KVMMK......LVQLY......YIVTE......YIHRD......SDFGM......KSDVW......
118084117    ......QTMMK......LVRLY......YLVME......LIHRD......SDFGM......KSDVW......
118090547    ......KLMMK......LVQLY......YVVTE......FIHRD......SDFGM......KSDIW......
118097426    ......KVLMK......LVQLY......CLVFE......VIHRD......SDFGM......KSDVW......
118764043    ......EVMMK......LVQLY......CLVFE......VIHRD......SDFGM......KSDVW......
119893849    ......KVMMK......LVQLY......YIVTE......FIHRD......SDFGM......KSDVW......
119893843    ......KVMMK......LVQLY......YIVTE......FIHRD......SDFGM......KSDVW......
119613457    ......KVMMK......LVQLY......YIVTE......YIHRD......SDFGM......KSDVW......
119623263    ......KVMMN......LVQLY......FIITE......FLHRD......SDFGL......KSDIW......
119619296    ......QTMMK......LVKFY......YIVTE......FIHRD......SDFGM......KSDVW......
125836368    ......KVMMK......LVQLY......YIVTE......FIHRD......TDFGL......KSDIW......
125852859    ......KIMTR......LVQLY......CIVTE......FIHRD......CDFGM......KSDVW......
126291353    ......EVMMK......LVQLY......CLVFE......VIHRD......SDFGM......KSDVW......
126331769    ......KVMTK......LVQLY......YIVTE......FIHRD......SDFGM......KSDVW......
126331767    ......KVMMK......LVQLY......YIVTE......FIHRD......SDFGM......KSDVW......
126342940    ......KVMMN......LVQLY......FIITE......FLHRD......SDFGL......KSDIW......
148705887    ......KVMMK......LVQLY......YIVTE......FIHRD......SDFGM......KSDVW......
148705886    ......KVMMK......LVQLY......YIVTE......YIHRD......SDFGM......KSDVW......
148701875    ......EVMMK......LVQLY......CLVFE......VIHRD......SDFGM......KSDVW......
148705888    ......KVMMK......LVQLY......YIVTE......FIHRD......SDFGM......KSDVW......
170295809    ......KVMMK......LVQLY......YIVTE......YIHRD......SDFGM......KSDVW......
148705884    ......KVMMK......LVQLY......YIVTE......YIHRD......SDFGM......KSDVW......
148705885    ......KVMMK......LVQLY......YIVTE......YIHRD......SDFGM......KSDVW......
149035282    ......KVMMK......LVQLY......YIVTE......YIHRD......SDFGM......KSDVW......
149035278    ......KVMMK......LVQLY......YIVTE......FIHRD......SDFGM......KSDVW......
149412562    ......EVMMK......LVQLY......CLVFE......VIHRD......SDFGM......KSDVW......
149618599    ......KVMMK......LVQLY......YIVTE......FIHRD......SDFGM......KSDVW......
149638364    ......QTMMK......LVKLY......YIVTE......FLHRD......TDFGM......KSDVW......
149640013    ......KVMMN......LVQLY......FIITE......FLHRD......SDFGL......KSDIW......
149702760    ......KVMTK......LVQLY......YIVTE......FIHRD......SDFGM......KSDVW......
149702998    ......KVMMK......LVQLY......YIVTE......FIHRD......SDFGM......KSDVW......
149726699    ......EVMMK......LVQLY......CLVFE......VIHRD......SDFGM......KSDVW......
149744269    ......QTMMK......LVKFY......YIVTE......FIHRD......SDFGM......KSDVW......
149755196    ......KVMMN......LVQLY......FIITE......FLHRD......SDFGL......KSDIW......
109017577    ......ELLTM......IVRFF......LMVFE......FVHRD......GDFGM......ESDVW......
```

109082274	......ELLTN......IVKFY......IMVFE......FVHRD......GDFGM......ESDVW......
109082270	......ELLTN......IVKFY......IMVFE......FVHRD......GDFGM......ESDVW......
109082266	......ELLTN......IVKFY......IMVFE......FVHRD......GDFGM......ESDVW......
109082262	......ELLTN......IVKFY......IMVFE......FVHRD......GDFGM......ESDVW......
109082272	......ELLTN......IVKFY......IMVFE......FVHRD......GDFGM......ESDVW......
109111970	......ELLTN......IVKFY......IMVFE......FVHRD......GDFGM......ESDVW......
109111968	......ELLTN......IVKFY......IMVFE......FVHRD......GDFGM......ESDVW......
109111966	......ELLTN......IVKFY......IMVFE......FVHRD......GDFGM......ESDVW......
112422314	......RVLQD......IVKLY......LLVFE......IVHRD......SDFGM......NSDIW......
118404588	......ELLTN......IVKFY......IMVFE......FVHRD......GDFGM......ESDVW......
119573285	......ELLTM......IVRFF......LMVFE......FVHRD......GDFGM......ESDVW......
119573286	......ELLTM......IVRFF......LMVFE......FVHRD......GDFGM......ESDVW......
119573287	......ELLTM......IVRFF......LMVFE......FVHRD......GDFGM......ESDVW......
119913609	......ELLTN......IVKFY......IMVFE......FVHRD......GDFGM......ESDVW......
125817371	......ELLTN......IVKFY......IMVFE......FVHRD......GDFGM......ESDVW......
125824969	......ELLTN......IVTFY......IMVFE......FVHRD......GDFGM......ESDVW......
125840574	......ELLTV......IVRFY......AMVFE......FVHRD......GDFGM......ESDIW......
125854606	......ELLTN......IVKFY......IMVFE......FVHRD......GDFGM......ESDVW......
126273734	......ELLTN......IVKFY......IMVFE......FVHRD......GDFGM......ESDVW......
126273737	......ELLTN......IVKFY......IMVFE......FVHRD......GDFGM......ESDVW......
126273731	......ELLTN......IVKFY......IMVFE......FVHRD......GDFGM......ESDVW......
133901966	......RVLQD......IVKLY......LLVFE......IVHRD......SDFGM......NSDIW......
148683400	......ELLTM......IVRFF......LMVFE......FVHRD......GDFGM......ESDVW......
148709326	......ELLTN......IVKFY......IMVFE......FVHRD......GDFGM......ESDVW......
149634164	......ELLTN......IVKFY......IMVFE......FVHRD......GDFGM......ESDVW......
149690978	......ELLTN......IVKFY......IMVFE......FVHRD......GDFGM......ESDVW......
149751604	......ELLTM......IVRFF......LMVFE......FVHRD......GDFGM......ESDVW......
149755253	......ELLTN......IVKFY......IMVFE......FVHRD......GDFGM......ESDVW......
149755251	......ELLTN......IVKFY......IMVFE......FVHRD......GDFGM......ESDVW......
109074853	......KILIH......VVNLL......MVIVE......CIHRD......CDFGL......QSDVW......
109080164	......KILIH......VVNLL......MVIVE......CIHRD......CDFGL......QSDVW......
109120359	......KILTH......VVNLL......MVIVE......CIHRD......CDFGL......KSDVW......
114594733	......KILIH......VVNLL......MVIVE......CIHRD......CDFGL......QSDVW......
114603893	......KILIH......VVNLL......MVIVE......CIHRD......CDFGL......QSDVW......
114649218	......KILTH......VVNLL......MVIVE......CIHRD......CDFGL......KSDVW......
115772526	......LKTKR......EPDLL......LEVGD......CIHRD......CDFGL......YSDVW......
118089586	......KILIH......VVNLL......MVIVE......CIHRD......CDFGL......QSDVW......
118097479	......KILIH......VVNLL......MVIVE......CIHRD......CDFGL......QSDVW......
158508551	......KILIH......VVNLL......MVIVE......CIHRD......CDFGL......QSDVW......
119625869	......KILIH......VVNLL......MVIVE......CIHRD......CDFGL......QSDVW......
119904625	......KILTH......VVNLL......MVIVE......CIHRD......CDFGL......KSDVW......
120538623	......KILIH......VVNLL......MIIVE......CIHRD......CDFGL......QSDVW......
122890731	......KILIH......VVNLL......MVIVE......CIHRD......CDFGL......QSDVW......
124297528	......KILIH......VVNLL......MVIVE......CIHRD......CDFGL......QSDVW......
114186831	......KILTH......VVNLL......MVIVE......CIHRD......CDFGL......KSDVW......
125836133	......KILIH......VVNLL......MIIVE......CIHRD......CDFGL......QSDVW......
126291662	......KILIH......VVNLL......MVIVE......CIHRD......CDFGL......QSDVW......
126327603	......KILTH......VVNLL......MVIVE......CIHRD......CDFGL......KSDVW......
126331735	......KILIH......VVNLL......MVIVE......CIHRD......CDFGL......QSDVW......
126342276	......KILIH......VVNLL......MVIVE......CIHRD......CDFGL......QSDVW......
144226988	......KMLIH......IVNLL......YVISE......CIHRD......CDFGL......YSDVW......
145580439	......KILIH......VVNLL......MVITE......CIHRD......CDFGL......QSDVW......
145580440	......KILIH......VVNLL......MVIVE......CIHRD......CDFGL......QSDVW......
148673892	......KILTH......VVNLL......MVIVE......CIHRD......CDFGL......KSDVW......
148673891	......KILTH......VVNLL......MVIVE......CIHRD......CDFGL......KSDVW......
148701798	......KILIH......VVNLL......MVIVE......CIHRD......CDFGL......QSDVW......
149034835	......KILTH......VVNLL......MVIVE......CIHRD......CDFGL......KSDVW......
149454165	......KILIH......VVNLL......MVIVE......CIHRD......CDFGL......RLPIL......
149599042	......KILTH......VVNLL......MVIVE......CIHRD......CDFGL......QSDVW......
149634852	......KILVH......VVNLL......MVIVE......CIHRD......CDFGL......QSDVW......
149702733	......KILIH......VVNLL......MVIVE......CIHRD......CDFGL......QSDVW......
149730119	......KILTH......VVNLL......MVIVE......CIHRD......CDFGL......KSDVW......
109104214	......ACMKD......VIRLL......MVILP......FLHRD......ADFGL......KSDVW......
109124924	......VCMKE......VMRLI......VVILP......FIHRD......ADFGL......KSDVW......
109461495	......VCMKE......VMRLI......VVILP......FIHRD......ADFGL......KSDVW......
110238735	......VCMKE......VMRLI......VVILP......FIHRD......ADFGL......KSDVW......
114579573	......ACMKD......VIRLL......MVILP......FLHRD......ADFGL......KSDVW......
118404376	......AFMKE......VCKLI......MVILP......FIHRD......ADFGL......HSDVW......
147904872	......VCMKE......VMRLI......VVILP......FIHRD......ADFGL......KSDVW......
119572482	......ACMKD......VIRLL......MVILP......FLHRD......ADFGL......KSDVW......
119572481	......ACMKD......VIRLL......MVILP......FLHRD......ADFGL......KSDVW......
119612913	......ACMKE......VAKLV......MVILP......FIHRD......ADFGL......QSDVW......
119612914	......ACMKE......VAKLV......MVILP......FIHRD......ADFGL......QSDVW......

119902363	.....ACMKE.....VAKLV.....MVILP.....FIHRD.....ADFGL.....HSDVW.....
119903129	.....ACMKD.....VIRLL.....MVILP.....FLHRD.....ADFGL.....KSDVW.....
119910557	.....VCMKE.....VMRLI.....VVILP.....FIHRD.....ADFGL.....KSDVW.....
123232208	.....ACMKE.....VAKLV.....MVILP.....FIHRD.....ADFGL.....HSDVW.....
126304251	.....ACMKD.....VITLL.....MVILP.....FLHRD.....ADFGL.....KSDVW.....
126329372	.....VCMKE.....VMRLI.....VVILP.....FIHRD.....ADFGL.....KSDVW.....
148696027	.....ACMKE.....VAKLV.....MVILP.....FIHRD.....ADFGL.....HSDVW.....
148696270	.....ACMKD.....VIRLL.....MVILP.....FLHRD.....ADFGL.....KSDVW.....
149023245	.....ACMKD.....VIRLL.....MVILP.....FLHRD.....ADFGL.....KSDVW.....
149023034	.....ACMKE.....VAKLV.....MVILP.....FIHRD.....ADFGL.....HSDVW.....
149056564	.....VCMKE.....VMRLI.....VVILP.....FIHRD.....ADFGL.....KSDVW.....
149242798	.....ACMKD.....VIRLL.....MVILP.....FLHRD.....ADFGL.....KSDVW.....
149692453	.....ACMKE.....VAKLV.....MVILP.....FIHRD.....ADFGL.....HSDVW.....
149722150	.....VCMKE.....VMRLI.....VVILP.....FIHRD.....ADFGL.....KSDVW.....
149722152	.....VCMKE.....VMRLI.....VVILP.....FIHRD.....ADFGL.....KSDVW.....
157106946	.....YIMQK.....IIKLI.....WIVME.....FVHRD.....ADFGL.....ASDVW.....
109085964	.....VIMKN.....IVRLI.....WIIME.....CVHRD.....GDFGL.....ASDVW.....
109085978	.....VIMKN.....IVRLI.....WIIME.....CVHRD.....GDFGL.....ASDVW.....
109085976	.....VIMKN.....IVRLI.....WIIME.....CVHRD.....GDFGL.....ASDVW.....
109087589	.....LRMRT.....GVPLS.....WIIVK.....FVHRD.....GDFGL.....ASDVW.....
109087585	.....LRMRT.....GVPLS.....WIIVK.....FVHRD.....GDFGL.....ASDVW.....
109087583	.....LRMRT.....GVPLS.....WIIVK.....FVHRD.....GDFGL.....ASDVW.....
109087601	.....LRMRT.....GVPLS.....WIIVK.....FVHRD.....GDFGL.....ASDVW.....
109087587	.....LRMRT.....GVPLS.....WIIVK.....FVHRD.....GDFGL.....ASDVW.....
110756271	.....YIMQQ.....IIRLI.....WLVME.....FVHRD.....ADFGL.....SSDVW.....
114619388	.....VIMKN.....IVKLI.....WIIME.....CVHRD.....GDFGL.....ASDVW.....
114619404	.....VIMKN.....IVKLI.....WIIME.....CVHRD.....GDFGL.....ASDVW.....
114619402	.....VIMKN.....IVKLI.....WIIME.....CVHRD.....GDFGL.....ASDVW.....
114619406	.....VIMKN.....IVKLI.....WIIME.....CVHRD.....GDFGL.....ASDVW.....
114619412	.....VIMKN.....IVKLI.....WIIME.....CVHRD.....GDFGL.....ASDVW.....
114619410	.....VIMKN.....IVKLI.....WIIME.....CVHRD.....GDFGL.....ASDVW.....
114619408	.....VIMKN.....IVKLI.....WIIME.....CVHRD.....GDFGL.....ASDVW.....
114619400	.....VIMKN.....IVKLI.....WIIME.....CVHRD.....GDFGL.....ASDVW.....
114621899	.....LTMRQ.....IVKLI.....WIIME.....FVHRD.....GDFGL.....ASDVW.....
114621913	.....LTMRQ.....IVKLI.....WIIME.....FVHRD.....GDFGL.....ASDVW.....
114621893	.....LTMRQ.....IVKLI.....WIIME.....FVHRD.....GDFGL.....ASDVW.....
114621937	.....LTMRQ.....IVKLI.....WIIME.....FVHRD.....GDFGL.....ASDVW.....
114621953	.....LTMRQ.....IVKLI.....WIIME.....FVHRD.....GDFGL.....ASDVW.....
114621901	.....LTMRQ.....IVKLI.....WIIME.....FVHRD.....GDFGL.....ASDVW.....
114621909	.....LTMRQ.....IVKLI.....WIIME.....FVHRD.....GDFGL.....ASDVW.....
114621895	.....LTMRQ.....IVKLI.....WIIME.....FVHRD.....GDFGL.....ASDVW.....
114621911	.....LTMRQ.....IVKLI.....WIIME.....FVHRD.....GDFGL.....ASDVW.....
114621961	.....LTMRQ.....IVKLI.....WIIME.....FVHRD.....GDFGL.....ASDVW.....
114621903	.....LTMRQ.....IVKLI.....WIIME.....FVHRD.....GDFGL.....ASDVW.....
114621959	.....LTMRQ.....IVKLI.....WIIME.....FVHRD.....GDFGL.....ASDVW.....
114621907	.....LTMRQ.....IVKLI.....WIIME.....FVHRD.....GDFGL.....ASDVW.....
114621905	.....LTMRQ.....IVKLI.....WIIME.....FVHRD.....GDFGL.....ASDVW.....
114621931	.....LTMRQ.....IVKLI.....WIIME.....FVHRD.....GDFGL.....ASDVW.....
114621935	.....LTMRQ.....IVKLI.....WIIME.....FVHRD.....GDFGL.....ASDVW.....
114621897	.....LTMRQ.....IVKLI.....WIIME.....FVHRD.....GDFGL.....ASDVW.....
114621949	.....LTMRQ.....IVKLI.....WIIME.....FVHRD.....GDFGL.....ASDVW.....
114621939	.....LTMRQ.....IVKLI.....WIIME.....FVHRD.....GDFGL.....ASDVW.....
114621933	.....LTMRQ.....IVKLI.....WIIME.....FVHRD.....GDFGL.....ASDVW.....
27886588f	.....VIMKN.....IVKLI.....WIIME.....CVHRD.....GDFGL.....ASDVW.....
117616356	.....LTMRQ.....IVKLI.....WIIME.....FVHRD.....GDFGL.....ASDVW.....
118137641	.....LTMRQ.....IVKLI.....WIIME.....FVHRD.....GDFGL.....ASDVW.....
90076004	.....LTMRQ.....IVKLI.....WIIME.....FVHRD.....GDFGL.....ASDVW.....
119612618	.....VTMRQ.....IVKLI.....WIIME.....FVHRD.....GDFGL.....ASDVW.....
119612617	.....LTMRQ.....IVKLI.....WIIME.....FVHRD.....GDFGL.....ASDVW.....
125841174	.....LIMKK.....IVRLI.....WIVME.....MVHRD.....GDFGL.....ASDVW.....
126303019	.....VLMKN.....IVKLI.....WIIME.....CVHRD.....GDFGL.....ASDVW.....
126303017	.....VLMKN.....IVKLI.....WIIME.....CVHRD.....GDFGL.....ASDVW.....
126322369	.....LTMRQ.....IVKLI.....WIIME.....FVHRD.....GDFGL.....ASDVW.....
148704054	.....VIMKN.....IVKLI.....WIIME.....CVHRD.....GDFGL.....ASDVW.....
148704056	.....VIMKN.....IVKLI.....WIIME.....CVHRD.....GDFGL.....ASDVW.....
148697470	.....LTMRQ.....IVKLI.....WIIME.....FVHRD.....GDFGL.....ASDVW.....
148704055	.....VIMKN.....IVKLI.....WIIME.....CVHRD.....GDFGL.....ASDVW.....
148697468	.....LTMRQ.....IVKLI.....WIIME.....FVHRD.....GDFGL.....ASDVW.....
148697469	.....LTMRQ.....IVKLI.....WIIME.....FVHRD.....GDFGL.....ASDVW.....
149030325	.....VIMKN.....IVKLI.....WIVME.....CVHRD.....GDFGL.....ASDVW.....

```
149066245    ......LTMRQ.....IVKLI.....WIIME.....FVHRD.....GDFGL.....ASDVW......
149066246    ......LTMRQ.....IVKLI.....WIIME.....FVHRD.....GDFGL.....ASDVW......
149410118    ......LTMRQ.....IVKLI.....WIIME.....FVHRD.....GDFGL.....ASDVW......
149452390    ......VLMKN.....IVKLI.....WIIME.....CVHRD.....GDFGL.....ASDVW......
149721678    ......LTMRQ.....IVKLI.....WIIME.....FVHRD.....GDFGL.....ASDVW......
149721680    ......LTMRQ.....IVKLI.....WIIME.....FVHRD.....GDFGL.....ASDVW......
149721682    ......LTMRQ.....IVKLI.....WIIME.....FVHRD.....GDFGL.....ASDVW......
156121149    ......VIMKN.....IVKLI.....WIIME.....CVHRD.....GDFGL.....ASDVW......
108711198    ......QMLSK.....LVSLI.....ILVYE.....IIHRD.....ADFGL.....KSDVY......
115450435    ......EAIGR.....LVRLL.....MLVYE.....VVHRD.....SDFGL.....RSDVY......
115450539    ......KGVGK.....LANLI.....LLVAE.....LYH-D.....SCFGL.....ESVIF......
108706407    ......NYLGQ.....LVKLI.....LLVYE.....VIYRD.....SDFGL.....KADVY......
108707199    ......SLLLG.....ILLPF.....MLVYE.....VIHGD.....SDFGL.....KCDIY......
115452117    ......EILGS.....LVNLR.....LLIYD.....IIHRD.....SDFGL.....KTDVY......
108707451    ......NYLGQ.....LVKLI.....LLVYE.....VIYRD.....SDFGL.....KSDVY......
115452207    ......NYLGQ.....LVKLI.....LLVYE.....VIYRD.....SDFGL.....KSDVY......
115453471    ......IFLGQ.....LVKLI.....LLVYE.....VIYRD.....SDFGL.....KSDVY......
115455669    ......DIISR.....LVSLV.....VLVYE.....IIHRD.....ADFGL.....KSDVF......
108706408    ......NYLGQ.....LVKLI.....LLVYE.....VIYRD.....SDFGL.....KADVY......
115456259    ......NYLGQ.....LVKLF.....LLVYE.....VIYRD.....SDFGL.....KSDVY......
115450419    ......EVLSR.....LVSLI.....ILVYE.....IIHRD.....ADFGL.....RSDVY......
115453823    ......DTISR.....LVTLV.....LLVYE.....IIHRD.....ADFGL.....RSDVF......
115452155    ......EIITR.....LVSLV.....LLVYE.....IIHRD.....ADFGL.....KADVF......
108705682    ......EVLGK.....LVMLL.....CLVYE.....IIHRD.....GDVGL.....KSDTY......
115452843    ......EMLSK.....LVSLI.....ILVYD.....IIHRD.....SDFGL.....KSDVY......
115453141    ......IFLGQ.....LVKLL.....LLVYE.....VIYRD.....SDFGL.....KSDVY......
115451111    ......IFLGQ.....LVKLI.....LLVYE.....VIYRD.....SDFGL.....KSDVY......
115488020    ......HLCSM.....IVKVL.....LLVEE.....IVHLD.....GDFGL.....KNDVY......
108862069    ......TLLSR.....LVSFL.....ILVYE.....IIHRD.....ADFGL.....KSDMY......
115488842    ......SVIGR.....LVRMW.....LLVFE.....LIHCD.....ADFGL.....KVDVY......
115486924    ......EAIGH.....LVRLL.....MLVYE.....VVHRD.....SDFGL.....KSDVY......
108862905    ......KLISS.....LVRLL.....ILVYE.....ILHRD.....ADFGL.....KSDVY......
108862906    ......KLISS.....LVRLL.....ILVYE.....ILHRD.....ADFGL.....KSDVY......
108864089    ......EIVSS.....IISLA.....LLVYE.....VIHRD.....SDFGL.....KIDVY......
108863918    ......EAIGH.....LVRLL.....MLVYE.....VVHRD.....SDFGL.....KSDVY......
108864366    ......KLISN.....LVRLL.....LLVYE.....IIHRD.....ADFGL.....KVDTY......
108864162    ......GILID.....IVKLI.....LLCYE.....IIHLD.....ADFGL.....KSDIF......
108864483    ......VLVSK.....LVRLV.....LLVYE.....IIHRD.....SDFGL.....KSDVF......
157116538    ......ELLSK.....IVELI.....CLVYE.....VVHRD.....CDFGL.....ALDVY......
109096213    ......KVMAK.....LVELL.....CLVYV.....HIHRD.....SDFGL.....KSDIY......
109289920    ......ILIAK.....LVRVL.....MLIYE.....VIHRD.....ADFGL.....KSDVF......
109289920    ......ILIAK.....LVRVL.....MLIYE.....VIHRD.....ADFGM.....KSDVF......
109462334    ......EQLSR.....IVDFA.....CLVYG.....LIHGD.....GDFGL.....DTDTF......
109638225    ......RLIAR.....LVRIL.....ILIYE.....IIHRD.....SDFGM.....KTDVF......
109638227    ......RLIAR.....LVRIL.....ILIYE.....IIHRD.....SDFGM.....KTDVF......
109716229    ......ETIGK.....LVPLL.....LLMYD.....IIHRD.....SDFGM.....KGDVY......
110288802    ......HHLMG.....IVQLI.....CLCYE.....IIHLD.....ADFGL.....KSDIY......
115480832    ......HLISK.....LVTLI.....CLVYE.....IVHGD.....ADCGI.....KSDIY......
110288705    ......SILSQ.....IVKLF.....LLVYD.....IFHRD.....SDFGA.....KSDVY......
110288534    ......IIQSR.....IIKLL.....MLVYE.....IRHGD.....SDFGT.....KSDVF......
110289142    ......NVLGV.....LVKLI.....LLVYE.....IIFRD.....SDFGL.....KNDIW......
110288690    ......ILQSE.....IIQLF.....MLVYE.....IRHGD.....SDFGT.....KSDIY......
110288706    ......SILSQ.....IVKLF.....LLVYD.....IFHRD.....SDFGA.....KSDVY......
110341803    ......LMLSL.....LVTLL.....ILVYE.....VVYRD.....ADFGL.....MSDIY......
110341792    ......LMLSL.....LVTLL.....ILVYE.....VVYRD.....ADFGL.....MSDIY......
110736139    ......LLVAK.....LVKLL.....LLVYE.....IIHRD.....ADFGM.....KTDVY......
110737406    ......EILSK.....LLLLL.....SLVYE.....IVHRD.....GDVGL.....ESDIY......
110738078    ......EILGK.....VLKLY.....YLVFE.....IIHRD.....ADFGV.....KSDVY......
110737903    ......KLISK.....LLRLL.....LLVYE.....VVHRD.....SDFGL.....KSDIY......
110738228    ......GIISH.....TALLL.....-LVFR.....IIHRD.....TDFGL.....KTDIY......
110738232    ......ILIAK.....LVRLL.....MLVYE.....IIHRD.....SDFGM.....KSDVY......
110738226    ......EAIGH.....LVRLL.....MLVYE.....VVHRD.....SDFGL.....KSDIY......
110738595    ......ETLSR.....LVLLR.....LLIYS.....ILHRD.....ADFGL.....KGDVY......
110739664    ......ELLSR.....LVSLI.....MLVYE.....VFHRD.....ADFGL.....KSDVY......
110739551    ......VVISK.....LVKLL.....MLVYE.....IIHRD.....SDFGL.....KSDVF......
110740019    ......EILGS.....LVNLR.....LLLYD.....IIHRD.....SDFGL.....KTDVY......
110741352    ......EILKS.....LVILY.....LLVYE.....IIHRD.....ADFGL.....KSDVY......
110741486    ......EMISM.....LLRLR.....LLVYP.....IIHRD.....GDFGL.....KTDVF......
145333847    ......VLVAK.....LVRLL.....IIVYE.....IIHRD.....ADFGT.....KSDVY......
113205211    ......NYLGQ.....LVKLI.....LLVYE.....VIYRD.....SDFGL.....KSDVY......
10177416     ......EMLSR.....LVNLI.....SLVYE.....VIHRD.....SDFGL.....KSDVY......
115457910    ......AILSQ.....IVKLF.....LLVYD.....VLHRD.....SDFGV.....KSDVY......
115460798    ......AVMAG.....LLRLL.....ILVYA.....VIHRD.....ADFGT.....KCDVY......
```

```
115460788      ......VLIAK.....LVKLL.....LLIYE.....IIHRD.....SDFGM.....KSDTY.....
115460790      ......VLIAK.....LVRLL.....LLIYE.....IIHRD.....SDFGM.....KSDIY.....
115438258      ......ESIGH.....LVRLL.....MLVYE.....VIHRD.....SDFGL.....KSDVY.....
115434126      ......QTIGS.....LVRLI.....LLVYE.....IAHLD.....SDFGL.....KADVY.....
115434126      ......ATIGT.....IVRLL.....ALVYE.....ILHFD.....SDFGL.....KSDVY.....
115440679      ......ELLSR.....LVSLV.....MLVYE.....IIHRD.....ADFGL.....KSDVY.....
115436194      ......IVHSQ.....VVRLL.....MIVME.....VLHGD.....SDFGI.....KSDVY.....
115434162      ......ATIGR.....IVRLL.....ALIYE.....ILHFD.....SDFGL.....KSDVY.....
115438737      ......EAIGH.....LVRLL.....MLVYE.....VVHRD.....SDFGL.....KSDVY.....
115439299      ......ASISR.....VVTLL.....ALIYE.....IVHFD.....SDFGM.....KSDVY.....
115435700      ......EMLSK.....LVSLI.....VLVYD.....IIHRD.....SDFGL.....KSDVY.....
115434144      ......ATIGQ.....IARLL.....ILIYE.....ILHFD.....SDFGL.....KSDVY.....
115434184      ......STIGR.....IVRLL.....ALIYE.....ILHFD.....SDFGL.....KSDVY.....
115434176      ......ATIGR.....IVRLL.....ALIYE.....ILHFD.....SDFGL.....KSDVY.....
115435382      ......QTVGM.....LVRLL.....LLVYE.....IIHCD.....CDFGM.....KADVY.....
115440611      ......QILSK.....LVTFL.....ALVYD.....IVHRD.....SDFGL.....KADVY.....
115434158      ......STIGR.....VVHLV.....ALVYE.....ILHFD.....ADFGL.....KSDVY.....
115439075      ......TLIGR.....LVRMW.....LLVYE.....LVHCD.....ADFGL.....KVDVY.....
115439291      ......AILSR.....LVMFY.....LLVYE.....IVHRD.....ADFGL.....KSDVY.....
115434130      ......STIGS.....VVRLV.....ALVYE.....ILHFD.....ADFGL.....KSDVY.....
115440615      ......EIFSC.....ISTAA.....CFPYQ.....IVHRD.....SDFGL.....KTDVY.....
115435062      ......EMLGR.....LVKLL.....CLVYE.....VIHRD.....SDFGL.....KSDVY.....
115434140      ......ATIGR.....IVRLL.....ALIYE.....ILHFD.....SDFGL.....KSDVY.....
115434186      ......STIGR.....VVRLV.....ALVYE.....ILHFD.....ADFGL.....KSDVY.....
115434164      ......ATIGR.....VVRLV.....ALVYE.....ILHFD.....ADFGL.....KSDVY.....
115435378      ......QTVGM.....LVRLL.....LLVYE.....IIHCD.....ADFGM.....KADVY.....
115435640      ......DTMGD.....IVPLC.....LLIYE.....VIHRD.....SDFGL.....KGDVY.....
115434188      ......ATIGR.....IVRLL.....ALIYE.....ILHFD.....SDFGL.....KSDVY.....
115435858      ......AVLAA.....VVNLV.....MLVME.....VIHRD.....ADFSL.....ESDVF.....
115444253      ......QHLTR.....LVSLI.....ALVYE.....LIHRD.....ADFGL.....KSDVY.....
115447425      ......ETLSK.....LVLLQ.....LLIYS.....ILHRD.....ADFGL.....KGDVY.....
115447449      ......VILSQ.....VVKIF.....LLVYE.....AIHRD.....SDFGA.....KSDVY.....
115447445      ......AILSQ.....VVKLF.....LLVYE.....AIHRD.....SDFGA.....KSDVY.....
115443749      ......IILSQ.....VVRLL.....MLVYE.....IVHGD.....TDFGA.....KSDVY.....
115447515      ......ESITE.....LVRLL.....ILIYE.....IVHRD.....GDFGI.....KADVY.....
115449837      ......EAVGR.....LVTLV.....AVVHE.....TVHGD.....AGLGM.....ERDVY.....
115444291      ......DALST.....LVPLW.....LLIYS.....IVHRD.....ADFGL.....RGDMY.....
115452809      ......ELLSR.....LVGLV.....MLVYE.....IIHRD.....ADFGL.....KSDVY.....
115451015      ......NYLGQ.....LVKLI.....LLVYE.....VIYRD.....SDFGL.....KADVY.....
115453591      ......SILRQ.....IITFI.....ALVYE.....LVHGD.....CNFGM.....LSDVY.....
115452079      ......NVISN.....LVKLI.....LLVYE.....IVHRD.....GDFGL.....KADIY.....
115451945      ......SLLLG.....ILLPF.....MLVYE.....VIHGD.....SDFGL.....KCDIY.....
115451697      ......EAIGK.....LVGLV.....MLVYE.....VVHRD.....SDFGM.....SSDIY.....
115450101      ......EVLGK.....LVMLL.....CLVYE.....IIHRD.....GDVGL.....KSDTY.....
115457366      ......EVLRS.....IMRPL.....ALIFK.....LVHCD.....GDFGS.....GGDVY.....
115457624      ......ETIGS.....LVRLI.....LLVYE.....IAHLD.....ADFGL.....KVDVY.....
115460994      ......VILSQ.....VVKLY.....LLVYE.....VLHRD.....SDFGA.....KSDIY.....
115461030      ......GTIGR.....LVRLY.....ALVYE.....IVHYD.....ADFGL.....KCDVY.....
115459594      ......VSIGH.....LVQLL.....LLVYD.....VIHRD.....GDFGL.....VTDVF.....
115457922      ......VILCR.....IVKLL.....MLVYE.....HLHGD.....SDFGC.....RNDVY.....
115461416      ......KSVGQ.....LANLL.....LLVAE.....LYH-D.....SCFGL.....ESVIY.....
115460588      ......ATLSR.....LARVV.....ALVLD.....VVHCD.....SDFGT.....KVDVF.....
115457584      ......ATISA.....LVRLH.....LLVYE.....IVHRD.....SDFGL.....KADVF.....
115460552      ......ETISR.....LVTLY.....LLVYE.....IVHRD.....SDFGL.....KVDVF.....
115457552      ......AILSQ.....IVRLF.....LLVYD.....VFHRD.....SDFGA.....KSDVY.....
115458430      ......EAIGR.....LVRLL.....MLVYE.....VVHRD.....SDFGL.....TSDVY.....
115461022      ......GTVGH.....VTALL.....-LIFE.....IIHRD.....SDFGL.....KTDVF.....
115457620      ......ETIGS.....LVRLI.....LLVYE.....IAHLD.....ADFGL.....KVDIY.....
115461060      ......NMITS.....LVRLV.....LLVYE.....IVHRD.....SDFGL.....KADTY.....
115459592      ......VSIGR.....LVQLL.....LLVYD.....VVHRD.....GDFGL.....ASDVF.....
115458100      ......LMLSL.....LVNLV.....LLVYE.....VIYRD.....SDFGL.....KSDVY.....
115463169      ......EMLSK.....LVSLI.....ILVYD.....IIHRD.....SDFGL.....KSDVY.....
115462683      ......EIISR.....LVSLV.....LLVYE.....IIHRD.....ADFGL.....KSDVF.....
115462713      ......AILGN.....LSV--......----.....IIHRD.....GDFGL.....GTDVF.....
115464993      ......QTLTK.....LVALI.....ALVYE.....FVHRD.....ADFGL.....KIDVY.....
115461943      ......AILSQ.....VVKLF.....LLVYE.....AIHRD.....SDFGA.....KSDVY.....
115462881      ......ATISA.....LVKLH.....LLVYE.....IVHRD.....SDFGL.....KADVF.....
115464555      ......ELLSR.....LVSLI.....MLVYE.....IIHRD.....ADFGL.....KSDVY.....
115463163      ......RVLSQ.....LVRLL.....LMVYE.....IYHRD.....SDFGL.....KSDVY.....
115463649      ......RILSS.....LVNLL.....LLVVE.....VIHRD.....GDFGL.....KTDVF.....
115462875      ......ATISA.....LVKLH.....LLVYE.....IVHRD.....SDFGL.....KADVF.....
115466866      ......EVLSC.....MVLLL.....CLVYE.....LVHRD.....SDVGL.....KSDVY.....
115466662      ......LILSQ.....IVKLL.....MLVYE.....IIHGD.....SDFGA.....KSDVY.....
```

115466340	......IMLSQ.....VVRLI......ILVYE.....IIHGD.....TDFGA......KSDVY......
115466310	......EVLGK.....LLLML......CLVYE.....IIHRD.....GDVGL......KSDVY......
115472135	......QLIAK.....LVNLR......LLIYE.....IIHRD.....SDFGL......KSDVF......
115471117	......ECLSH.....LVRPI......LLLHH.....TIHLD.....GEVEI......PGNVY......
115470455	......VSIGH.....LVQLL......LLVYD.....VIHRD.....GDFGL......LTDVF......
115470447	......ASIGR.....LVQLL......LLVYD.....VIHRD.....GDFGL......LTDVF......
115472855	......RLLSA.....LVPLI......ILVYP.....IIHRD.....ADFGF......KSDVF......
115473507	......VLIAK.....LVRLV......LLVYE.....IIHRD.....SDFGL......KSDVY......
115470225	......EALSQ.....LVSLR......LLIYS.....IIHRD.....ADFGL......KGDVY......
115472577	......LLLAE.....LVRLH......LLVYE.....IIHRD.....ADFGL......KIDIF......
115472531	......QLIAK.....LVKLL......MLVYE.....VVHRD.....SDFGM......KSDVF......
115472567	......LLLAE.....LVRLQ......LLVYE.....IIHRD.....ADFGL......KIDIF......
115472701	......STIGT.....LVRLL......LLVYE.....IIHCD.....SDFGL......KADVF......
115470469	......VSIGR.....IVQLH......LLVYD.....VVHRD.....GDFGL......LTDVF......
115470449	......VSIGR.....LVQLL......LLVYE.....IVHRD.....GDFGL......LTDIF......
115472597	......LLVAK.....LVRLI......ILAYE.....IVHRD.....SDFGL......KLDVY......
115470461	......VSIGH.....LVQLH......ILVYD.....ILHRD.....GDFGL......LTDVF......
115470461	......VSIGR.....LVQLL......LLVYE.....VIHRD.....GDFGL......LTDVY......
115473155	......EVLAT.....LVTLY......ILVYE.....VVHRD.....TDFGL......KGDVY......
115470445	......VSLGR.....LVRLL......MLVYE.....VIHRD.....GDFGL......LTDVF......
115472555	......LVLAE.....LVRLQ......LLVYE.....IIHRD.....ADFGL......KIDIF......
115477138	......AILSQ.....VVKLL......LLVYE.....IFYRD.....SDFRA......KSDVF......
115475249	......ATISA.....LVKLH......LLVYE.....IVHRD.....SDFGL......KADVF......
115478745	......QSLTK.....LVSLV......ALAYE.....IIHGD.....ADFGL......SSDVY......
115478759	......QSLSK.....LVSLV......ALVYE.....IIHRD.....ADFGL......SSDIY......
115478727	......QSLTT.....LVSLF......ALVYE.....IIHGD.....ADFGL......SSDVY......
115480117	......SIVSR.....FVEML......LVAYE.....IVHRD.....ADFNP......KSDVY......
115477549	......KVLTH.....LVRLI......-LVYE.....VIHRD.....ADFGL......KVDVY......
115478172	......TIIAD.....LVHLQ......LLVYE.....VIHRD.....GDFGL......KSDVY......
115475255	......ATISS.....LVKLH......LLVYE.....IVHRD.....SDFGL......KVDVY......
115480773	......EILGR.....LVTLI......ALVYE.....IAHGD.....GDFGI......QYDVY......
115478773	......DFLGN.....LVRLI......LLVYE.....VIYRD.....SDFGL......KSDVY......
115478749	......ESLTT.....LVFLV......ALIYE.....IIHRD.....SDFGL......SSDVF......
115475231	......TAISD.....LITLV......ILVYN.....IIHRD.....SDFGL......KSDIY......
115478723	......QSLTK.....LVSLI......ALVYE.....IIHGD.....ADFGL......SSDVY......
115475251	......ATISA.....LVKLY......LLVYE.....IVHRD.....SDFGL......KADVF......
115480777	......EILSR.....LVTLI......ALVYE.....IVHSD.....SGFGV......LSDVY......
115478685	......GMISA.....LVKLY......LLVYE.....IVHRD.....SDFGL......KADVY......
115478725	......QSLTK.....LVSLV......ALVYE.....IIHGD.....ADFGL......SSDVY......
115478721	......QSLTK.....LVCLV......ALVYE.....IIHGD.....ADFG-......SSDVY......
115476142	......AILSP.....IVRLL......LLVYE.....VIHRD.....SDFGL......ASDVY......
115480541	......ILIAK.....LVRLL......LLIYE.....IIHRD.....ADFGM......KSDVY......
115480541	......ILIAK.....LVRLL......LLIYE.....IIHRD.....ADFGM......KSDVY......
115477136	......AVLSQ.....VVKLL......LLVYE.....IFHRD.....SDFGA......RSDVF......
115478635	......KIISR.....LVQLI......LIVYE.....VVHGD.....GDFGL......ESDVY......
115481412	......HHLMG.....IVQLI......CLCYE.....IIHLD.....ADFGL......KSDIY......
115481172	......IIQSR.....IIKLL......MLVYE.....IRHGD.....SDFGT......KSDVY......
115482584	......KLISN.....LVRLL......LLVYE.....HIHRD.....ADFGL......KVDTY......
115481200	......KILNT.....IVRMA......LILYE.....IVHRD.....TDFGM......KSDVY......
115483266	......AILSR.....LVTLI......ALVYE.....VVHGD.....SDFGI......QSDTY......
115484791	......GILID.....IVKLI......LLCYE.....IIHLD.....ADFGL......KSDIF......
115485457	......KLISN.....LVRLL......LLVYE.....IIHRD.....ADFGL......KVDTY......
115486543	......QGMEV.....IVPLY......LLVYE.....IIHRD.....TDFGY......TIDIY......
115483717	......TLLSR.....LVSFL......ILVYE.....IIHRD.....ADFGL......KSDMY......
115484557	......EIVSS.....IISLA......LLVYE.....VIHRD.....SDFGL......KIDVY......
115486721	......TVQLQ.....LVCLI......MLVFE.....IFHGD.....SDFGS......KSDVY......
115487070	......RFLGQ.....LVKLI......LLVYE.....IIYRD.....SDFGL......RSDVY......
115484015	......SVIGR.....LVRMW......ILVYE.....IIHCD.....TDFGL......KVDVY......
115486862	......TLLSR.....LVSFL......ILVYE.....IIHRD.....ADFGL......KSDMY......
115485851	......VLVSK.....LVRLV......LLVYE.....IIHRD.....SDFGL......KSDVF......
115489720	......GTLSS.....VVKLL......LLVYE.....ILHRD.....ADFGL......KSDVY......
114645208	......KVMAK.....LVELL......CLVYV.....HIHRD.....SDFGL......KSDIY......
114645212	......KVMAK.....LVELL......CLVYV.....HIHRD.....SDFGL......KSDIY......
114649545	......KVLTH.....LVRLI......-LVYE.....VIHRD.....ADFGL......DLLVV......
114690655	......EQLSR.....IVDFA......CLVYG.....LIHGD.....GDFGL......DTDTF......
115900705	......EALTK.....IVSLT......CLIFD.....LIHQD.....ADFGL......KLDVY......
116008311	......ATIGL.....IVQLL......ALIYE.....ILHFD.....SDFGL......KSDVY......
82582807	......ATIGL.....IVRLL......ALIYE.....ILHFD.....SDFGL......KSDVY......
82582805	......ATIGL.....IVRLL......ALIYE.....ILHFD.....SDFGL......KSDVY......
162459964	......SLASK.....LVEML......ILAYE.....IIHRD.....ADFNL......KSDVY......
162459810	......SMVSR.....VVELL......VLAYE.....IIHRD.....ADFDL......KSDVY......

109657910	......SIASK......FVEML......LVVYE......IVHRD......ADFNL......KSDVY......
118783059	......DILSK......VVSLL......CLVYE......IIHRD......CDFGL......ALDIF......
118781894	......RFLNS......IVPLF......CLVYQ......FIHGD......GDFGL......KVDTY......
116312039	......VSIGH......LVQLL......LLVYD......VIHRD......GDFGL......VTDVF......
116312038	......VSIGR......LVQLL......LLVYD......VVHRD......GDFGL......ASDVF......
116309379	......LMLSL......LVNLV......LLVYE......VIYRD......SDFGL......KSDVY......
116309653	......SVIGR......LVRIW......LLVSE......IVHCD......TDFGL......KADVY......
116309700	......NFLGR......LVRLI......LLVYE......IIYRD......SDFGL......KSDVY......
116308957	......ETIGS......LVKVI......LLVYE......IAHLD......ADFGL......KVDVY......
116309574	......LILSR......IVKLL......VLVYE......HLHGD......SDFGK......KSDVY......
116309549	......AILLR......IVKLF......LLVYD......VFHRD......SDFGT......KSDVY......
116309112	......ETIGS......LVRLI......LLVYE......IAHLD......ADFGL......KVDVY......
116309805	......ALLSR......LVRLL......ILVYE......IIHRD......SDFGL......KSDVY......
116309655	......GTIGR......LVRLY......ALVYE......IVHYD......ADFGL......KCDVY......
116309309	......ELLGR......LVGLK......FQVYE......LCHGD......AVSGL......KSDVY......
116309300	......LILSR......IVKLL......VLVYE......HLHGD......SDFGK......KSDVY......
116309943	......EMLSK......LVAMI......ILVYE......IIHRD......ADFGL......KSDVY......
116310289	......QTIGW......LVRLL......ILVYE......IIHCD......SDFGL......KVDVY......
116310852	......NMITS......LVRLV......LLVYE......IVHRD......SDFGL......KADTY......
116311967	......VILSQ......VVKLY......LLVYE......VLHRD......SDFGA......KSDIY......
116311954	......ETISR......LVKLY......LLVYE......VVHRD......SDFGL......KVDVF......
116311987	......ELLAR......LVTLK......FLVYE......LCHRD......ADFGL......KSDIY......
116317907	......QTISR......LVELL......CLVYE......HLHRD......ADFGL......SSDVF......
116317803	......EILST......LVKLV......ALVYE......VVHCD......GDFGL......SGDVY......
116831353	......FRLSL......LANLI......LLVHE......VIYRD......SDFGL......KSDVY......
116831407	......ELLLT......LASFV......ALIYE......IVHRD......ADFGL......KSDVY......
116831497	......LMLSL......LVNLI......LLVYE......VIYRD......SDFGL......KSDVY......
117646774	......EQLSR......IVDFA......CLVYG......LIHGD......GDFGL......DTDTF......
117616446	......EQLSR......IVDFA......CLVYG......LIHGD......GDFGL......DTDTF......
119467424	......EVLSL......ITSLL......DMAVP......IVHGD......SDFGV......RFDAY......
119638451	......EVLGK......LLLML......CLVYE......IIHRD......GDAGL......KSDVY......
119638466	......IILSQ......VVRLL......ILVYE......IVHGD......TDFGA......KSDVY......
162464025	......SMVSR......VVELL......VLAYE......IIHRD......ADFDL......KSDVY......
121308607	......EIISR......LVSLV......LLVYE......IIHRD......ADFGL......KSDVF......
122920986	......KVMAK......LVELL......CLVYV......HIHRD......SDFGL......KSDIY......
122920981	......KVMAK......LVELL......CLVYV......HIHRD......SDFGL......KSDIY......
124221924	......RILCQ......LLRIL......LLVYE......IYHRD......SDFGL......KSDVY......
124360779	......EMLSR......LVKLI......CLVYE......VIHRD......SDFGL......KSDVY......
125569577	......QTVGM......LVRLL......LLVYE......IIHCD......CDFGM......KADVY......
125570941	......EVLSC......MVLLL......CLVYE......LVHRD......SDVGL......KSDIY......
125568766	......QTIGS......LVRLI......LLVYE......IAHLD......SDFGL......KADVY......
125571978	......RILGN......IVRLL......LLVYD......IVHRD......ADFGL......KVDVY......
125568765	......ATIGT......IVRLL......ALVYE......ILHFD......SDFGL......KSDVY......
125596013	......IMLSQ......VVRLI......ILVYE......IIHGD......TDFGA......KSDVY......
125571523	......SVIGR......LVRTW......LLVYD......LVHCD......ADFGL......KVDVF......
125592152	......ELLAR......LVTLK......FLVYE......LCHRD......ADFGL......KSDIY......
125548819	......SVASR......FVRLL......VLVYE......VTHKD......ADFSQ......KSDVY......
125592079	......DVITN......LVELI......ILVYE......IVHRD......GDFGL......RADIY......
125590038	......LILCR......VVKLL......ILVYE......ILHGD......ADFGC......KSDVY......
125589797	......ETIGS......LVRLI......LLVYE......IAHLD......ADFGL......KVDVY......
125578200	......TLLSR......LVSFL......ILVYE......IIHRD......ADFGL......KSDMY......
125527654	......KVLCK......LVELI......YLIYE......YVHRD......SDFGL......KNDVY......
125527509	......ETIGK......LVPLL......LLVYD......IIHRD......SDFGM......KGDVY......
125527200	......TLIGR......LVRMW......LLVYE......LVHCD......ADFGL......KVDVY......
125526046	......EVVAS......LVAIR......MIVCD......IIHRD......ADFGL......KSDVY......
125527202	......TVLGR......LVRIW......LLVYE......LIHCD......ADFGL......KVDVY......
125528724	......ALVSR......FVDML......LVAYE......IVHRD......ADFNL......KSDVY......
125526624	......DFLGN......LVKLV......LLVYE......VIYRD......SDFGL......KSDVY......
125524154	......QTIGS......LVRLI......LLVYE......IAHLD......SDFGL......KADVY......
125528581	......NTLGK......ILKLH......FLVYE......IIHRD......ADFGI......KSDVY......
125527201	......SIIGR......LVRMW......LLVYE......LVHCD......ADFGL......KVDVY......
125526330	......KTIGR......LVSLI......LLVYE......IAHLD......SDFGL......KADTY......
125525215	......EMLSK......LVSLI......VLVYD......IIHRD......SDFGL......KSDVY......
125524486	......EMLSG......LVSLI......ILLYE......VIHRD......ADFGL......KSDVY......
125524148	......QTIGS......LVRLI......LLVYE......IAHLD......SDFGL......KADVY......
125528000	......LMLSL......LVKLL......ILVYE......VIYRD......SDFGL......TSDIY......
125525658	......SAVSR......IIQLI......VLAYE......VVHRD......GDFDV......KSDVY......
125529203	......ELLSR......LVSLL......MLVYE......IFHRD......ADFGL......KSDVY......
125524321	......DILSR......LVILY......LLVYE......IIHRD......ADFGL......KSDVY......
125524969	......QTVGM......LVRLL......LLVYE......HIHCD......ADFGM......KADVY......
125528172	......ELLSR......LVSLV......MLVYE......IFHRD......ADFGL......KSDVY......
125524125	......EIISR......LVSLV......LLVYD......IIHRD......ADFGL......RSDVF......
125524827	......ETIGR......LVRLI......LLVYE......IAHLD......CDFGL......KVDVY......
125527205	......SVIGR......LVRTW......LLVYD......LVHCD......ADFGL......KVDVF......

```
125524260    ......RLLLK.....LVSLL.....MLVYP.....IIHRD.....SDFGM.....KTDVF.....
125526953    ......EAIGH.....LVRLL.....MLVYE.....VVHRD.....SDFGL.....KSDVY.....
125528111    ......QTLSK.....LVTLV.....ALVYD.....IVHRD.....SDFGL.....KTDVY.....
125526170    ......EAIGK.....IVTLR.....LLIYD.....YVHGD.....SDFGL.....KWDVF.....
125524765    ......EMLGR.....LVKLL.....CLVYE.....VIHRD.....SDFGL.....KSDVY.....
125526715    ......ESIGH.....LVRLL.....MLVYE.....VIHRD.....SDFGL.....KSDVY.....
125528614    ......EIISH.....LVPLR.....FLVYD.....IYHRD.....ADFGL.....KSDVY.....
125528156    ......ELLSR.....LVSLV.....MLVYE.....IIHRD.....ADFGL.....KSDVY.....
125527318    ......AILSR.....LVMFY.....LLVYE.....IVHRD.....ADFGL.....KSDVY.....
125525594    ......IVHSQ.....VVRLL.....MIVME.....VLHGD.....SDFGI.....KSDVY.....
125525257    ......LMLSL.....LVKLF.....LLIYE.....VIYRD.....SDFGL.....KSDIY.....
125526551    ......EVLSC.....MVLLL.....CLVYE.....LVHRD.....SDVGL.....KSDIY.....
125524069    ......GRVAK.....LVAVK.....FIVYE.....VVHRD.....MGVGL.....KSDVY.....
125529302    ......NYLGQ.....LVKLV.....LLVYE.....VIYRD.....SDFGL.....KSDVY.....
125524233    ......QILTR.....LVSMI.....ALVYE.....IIHRD.....ADFGL.....KSDVY.....
125524592    ......GVLPR.....IVRLL.....LLFYD.....ILHRD.....ADFGL.....KSDVY.....
125524992    ......EIISR.....LVTLV.....LLVYE.....IIHRD.....ADFGL.....KSDVF.....
125526358    ......ELISQ.....LVKLL.....LLVYE.....IIHRD.....TDFGL.....KADVY.....
125527639    ......DTISR.....LVSLV.....MLVYD.....IIHRD.....SDFGL.....KSDVY.....
125527447    ......EMLSR.....LVKLI.....CLVYE.....VIHRD.....SDFGL.....KSDVY.....
125525168    ......DTMGD.....IVPLC.....LLIYE.....VIHRD.....SDFGL.....KGDVY.....
125527660    ......RILGN.....IVRLL.....LLVYN.....IVHRD.....ADFGL.....KVDVY.....
125538126    ......ETLSM.....LVPLL.....LLIYS.....IVHRD.....ADFGL.....KGDVY.....
125533574    ......NALRN.....LVPIL.....ALVYK.....IIHCD.....GDFGL.....AADVY.....
125535127    ......AILKR.....LITLR.....ALMYE.....IAHGD.....SDFGI.....QSDVF.....
125539078    ......ASIGR.....LVQLF.....LLVYD.....VIHRD.....GDFGL.....LTDVF.....
125533027    ......AGVGK.....LVNLI.....LLVAE.....KIYHD.....SSFGL.....ESVIY.....
125532955    ......EVLGK.....LLLLI.....CLVYE.....IIHRD.....GDVGL.....KSDTY.....
125540441    ......ESITE.....LVRLL.....ILIYE.....IVHRD.....GDFGV.....KADVY.....
125532305    ......KALTE.....IVKLH.....YLVYE.....IVHRD.....CDFGT.....KCDVY.....
125532859    ......AILSR.....LVTLI.....ALVYE.....VVHGD.....SDFGI.....QSDTY.....
125535304    ......IVQSR.....VVRLI.....MLVFE.....IIHGD.....SDFGS.....KSDVY.....
125540687    ......KILQK.....LVKLE.....YLVYE.....VVHKD.....ANFGL.....KMDVF.....
125538124    ......DALST.....LVPLW.....LLIYS.....IVHRD.....ADFGL.....RGDMY.....
125538111    ......ELLNN.....MVHLL.....CLVYE.....FVHRD.....SDVGL.....KSDVY.....
125540856    ......ELIAK.....LVRLL.....MLIYE.....VIHRD.....SDFGM.....KSDVF.....
125531854    ......DYLGQ.....LVKLI.....LLVYE.....VIYRD.....SDFGL.....KADVY.....
125537267    ......KLISS.....LVRLL.....ILVYE.....ILHRD.....ADFGL.....KSDVY.....
125540597    ......AVLSR.....LVTLI.....GLVYE.....IVHGD.....GDFGI.....RSDVY.....
125540452    ......VSIGQ.....LVQLL.....LLVYD.....VLHRD.....GDFGL.....ASDIF.....
125539628    ......LMLSL.....LVNLI.....LLVYE.....VIYRD.....SDFGL.....KSDVY.....
125538636    ......DILSQ.....LVLLL.....CLVYE.....IVHRD.....GDAGF.....KSDLF.....
125535982    ......EIVSS.....AMPLA.....MLVYD.....VIHRD.....CDFGL.....KIDVY.....
125531180    ......KILNT.....IVRMA.....LILYE.....IVHRD.....TDFGM.....KSDVY.....
125538722    ......GIISA.....LVRLF.....LLIYE.....IIHRD.....SDFGL.....KADVY.....
125531120    ......N-LPE.....IIQLL.....ILVYE.....ILHGD.....SGFGS.....KSDVY.....
125530988    ......TVQLQ.....LVCLI.....MLVFE.....IFHGD.....SDFGS.....KSDVY.....
125534595    ......QSVGQ.....LVRMI.....MLVFE.....IIHCD.....TDFGI.....KADVY.....
125531076    ......QLIAK.....LVRLL.....ILVYE.....VIHRD.....SDFGL.....KSDVF.....
125531208    ......IIQSR.....IVKLI.....ILVYE.....ILHGD.....SDFGI.....KSDVY.....
125537340    ......RIISR.....LVQLI.....LLVYE.....VVHRD.....GDFGL.....ESDVY.....
125537520    ......GTLSS.....VVKLL.....LLVYE.....ILHRD.....ADFGL.....KSDVY.....
125540513    ......QFLGV.....LVKLI.....LLVYE.....VIYRD.....SDFGL.....KSDVW.....
125539938    ......AMVSK.....FLELL.....MLAYQ.....IVHRD.....ADFNL.....KSDVY.....
125534329    ......KLISN.....LVRLL.....LLVYE.....IIHRD.....ADFGL.....KVDTY.....
125538741    ......ELIGL.....LLRLY.....LLVYP.....IIHRD.....GDFGL.....KTDVY.....
125539074    ......VSIGR.....IVQLL.....LLVYD.....VVHRD.....GDFGL.....LTDVF.....
125538128    ......EALTM.....LVPLW.....LLIYS.....IVHRD.....ADFGL.....RGDIY.....
125532757    ......ATIGR.....LVSLL.....LLVYE.....IVHRD.....SDFGL.....RSDVY.....
125536850    ......SVIGR.....LVRMW.....LLVFE.....LIHCD.....ADFGL.....KVDVY.....
125530998    ......KIHLQ.....LVRLI.....QLVLE.....IIHGD.....SDFGL.....KSDVY.....
125536100    ......ELLSR.....VVSLV.....MLVYE.....IIHRD.....SDFGL.....RSDVY.....
125539076    ......ASIGR.....LVQLL.....LLVYD.....VIHRD.....GDFGL.....LTDVF.....
125538699    ......RILGE.....IIDLL.....LLVYE.....IMHRD.....ADFGL.....KVDVY.....
125530884    ......HLISK.....LVTLI.....CLVYE.....IVHGD.....ADCGI.....KSDIY.....
125532512    ......EAIGH.....LVRLL.....MLVYE.....VVHRD.....SDFGL.....KSDVY.....
125535807    ......KLISN.....LVRLL.....LLVYE.....HIHRD.....ADFGL.....KVDTY.....
125532862    ......AVLSR.....LVTLV.....-LVYE.....VIHGD.....GDFGI.....RSDIY.....
125538383    ......EVLAT.....LLSLR.....LIVYD.....IIHRD.....ADFGF.....SCDVF.....
125535455    ......TLLSR.....LVSFL.....ILVYE.....IIHRD.....ADFGL.....KSDMY.....
125538123    ......DALSM.....LVPLW.....FLIYS.....IVHRD.....ADFGL.....RGDMY.....
125535676    ......SVIGR.....LVRMW.....ILVYE.....IIHCD.....TNFGL.....KVDVY.....
125538101    ......QHLTR.....LVSLI.....ALVYE.....LIHRD.....ADFGL.....KSDVY.....
```

125532824	......RGVGK......MANLI......LLVAE......LYH-D......SCFGL......ESVVF......
125538689	......TTLGM......IILLL......VILYE......IIHGD......SGFSY......KVDVY......
125533288	......SVIGR......LVRMW......ILVYE......IIHCD......TDFGL......KVDVY......
125535122	......GILKR......LVILL......ALMYE......IAHGD......SDFGI......QSDVF......
125533796	......QNLMR......IVRLV......ALCFE......IYHLD......ADFGL......KLDIF......
125532766	......LVLSS......LVSLI......ILVYE......IIHRD......ADFGL......RSDVY......
125534752	......RIHAQ......IVKVI......MMVTE......ICHGD......ADFGL......KSDIY......
125534708	......VLVSK......LVRLV......LLVYE......IIHRD......SDFGL......KSDVF......
125532121	......NVLGV......LVKLI......LLVYE......IIFRD......SDFGL......KNDIW......
125538588	......KVQSL......VVTLL......TLILE......IVHGD......SDFGS......KSDVY......
125532638	......DALLR......IVRLL......VLVLE......VVHGD......CDFGS......KSDVY......
125533866	......GILID......IVKLI......LLCYE......IIHLD......ADFGL......KSDIF......
125530997	......KAQLQ......VVRLI......MLVFE......IVHGD......SDFGS......KSDVY......
125537347	......RIISR......LVQLI......LLVYE......VVHRD......GDFGL......ESDVY......
125532050	......IFLGH......LVRLV......LLVYE......VIYRD......SDFGL......MSDVY......
125538690	......RILGE......IIDLL......LLVYE......IMHRD......ADFGL......KVDVY......
125530946	......ETLGK......LVPLL......FLVYE......IIHRD......ADFGM......KGDVY......
125538457	......ETVGK......LVPLL......FLVYE......VIHRD......SDFGL......KGDVY......
125539838	......EAIGR......LVRLL......MLVYE......VVHRD......SDFGL......TSDVY......
125531077	......QLIAK......LVRLL......ILVYE......VIHRD......SDFGL......KSDVF......
125540503	......DVLLS......VVRLL......VLVFE......VIHGD......CDFGF......KSDVY......
125532762	......ASLGR......LVELR......LLVYE......VVHRD......GDFGL......ATDVF......
125534749	......RIHAQ......IVKLI......MMVTE......ICHGD......SDFGI......KSDVY......
125538127	......DALSM......LVPFW......LLIYS......IVHRD......ADFGL......RGDMY......
125538992	......ELISV......LLKLI......LLVYP......IIHRD......GDFGL......RTDVF......
125531177	......KILNT......IVRMA......LILYE......IIHRD......TDFGM......KSDVY......
125534712	......VLVGK......LVRLV......LLVYE......IIHRD......SDFGL......KSDVF......
125538118	......DALST......LVPLW......LLIYS......IVHRD......ADFGL......RGDMY......
125537767	......ELLSR......VVPLL......LLVFE......ILHRD......TDLGM......KSDVF......
125535511	......EAIGH......LVRLL......MLVYE......VVHRD......SDFGL......KSDVY......
125547920	......VILCR......IVKLL......MLVYE......HLHGD......SDFGC......KNDVY......
125544996	......DLLSR......LLNLL......LLVYE......VIHRD......ADFGL......KSDVY......
125542984	......EAIGK......LVGLV......MLVYE......VVHRD......SDFGM......SSDIY......
125548986	......EMLSR......LVKLI......CLVFE......VIHRD......ADFGL......KSDVY......
125550068	......NMITS......LVRLV......LLVYE......IVHRD......SDFGL......KADTY......
125543236	......EILGS......LVNLR......LLIYD......IIHRD......SDFGL......KTDVY......
125543366	......ELLSQ......LVSLI......ILVYE......IIHRD......ADFGL......KSDVY......
125545568	......DLFAK......LVPIM......KLVYI......MVHRD......GSLSE......SYDVY......
125546361	......ATISS......LVRLI......LLVYE......IVHCD......SDFGL......KSDVY......
125546264	......RAVGQ......LANLI......LLVAE......LYH-D......SCFGL......ESVVY......
125542577	......NYLGQ......LVKLI......LLVYE......VIYRD......SDFGL......KADVY......
125542637	......IFLGQ......LVKLI......LLVYE......VIYRD......SDFGL......KSDVY......
125542199	......EVLSR......LVSLI......ILVYE......IIHRD......ADFGL......RSDVY......
125550218	......EIVSS......IISLA......LLVYE......VIHRD......SDFGL......KIDVY......
125548332	......EAIGR......LVRLL......MLVYE......VVHRD......SDFGL......TSDVY......
125550557	......NYLGQ......LVRLV......LLVYE......VIYRD......SDFGL......KSDVY......
125546248	......ASMSR......LVQLL......VLVYD......VVHRD......SDFGL......STDVF......
125550755	......SMVSR......VVELL......VLAYE......IIHRD......ADFDL......KSDVY......
125550040	......GTIGR......LVRLY......ALVYE......IVHYD......ADFGL......KCDVY......
125541090	......GTIGR......LVMLY......ALVYE......IIHYD......GDFGL......KCDVY......
125547684	......ETIGS......LVRLI......LLVYE......IAHLD......ADFGL......KVDVY......
125545937	......S-LLS......IVRLL......LIVYE......VIHRD......TDFGL......ASDVY......
125547939	......LILSR......IVKLL......VLVYE......HLHGD......SDFGK......KSDVY......
125541612	......EMLSR......LVKLI......CLVYE......VIHRD......TDFGL......KSDVY......
125549880	......AVMAG......LLRLL......ILVYA......VIHRD......ADFGT......KCDVY......
125545860	......VSIGR......LVQLL......ILVYD......VIHRD......GDFGL......LTDVF......
125543068	......DIITK......IVPLI......ISVYS......VIHRD......SDFGL......KVDVY......
125545516	......AVLTK......LVSLL......ILVYE......FIHRD......ADFGL......KADVF......
125547679	......ETIGS......LVKVI......LLVYE......IAHLD......ADFGL......KVDVY......
125550689	......AFLSR......LVGFV......LLVYE......IIHRD......SDFGL......KSDVY......
125546373	......EIATS......IVSLR......VLVYD......VIHGD......CDFGL......KIDVY......
125544378	......SILRQ......IITFI......ALVYE......LVHGD......CNFGM......LSDVY......
125550711	......RLVSQ......LVKLL......LLVYE......IIHRD......ADFGL......KADIY......
125541663	......KAMAR......LVRFY......VIVVE......-MYAD......GDFGF......RSDVY......
125547985	......ELLSG......LVSLI......ILVYE......IIHRD......TDFGI......KSDVY......
125547149	......AAVAR......LVRLR......LLVYD......IIHRD......GDFGL......KSDVF......
125544501	......KLIAK......LVRLL......MLIYE......IIHRD......SDFGV......KSDVF......
125543211	......NVISN......LVKLI......LLVYE......IVHRD......GDFGL......KADIY......
125546965	......QVIIR......IVQLI......LLVYE......HELED......GDFGL......RTDVY......
125545357	......SIQSQ......VVRLL......ILVYE......ILHGD......SDFGI......KSDVY......
125545415	......ELISV......LLRLI......LLVYP......IIHRD......GDFGL......RTDVF......
125547150	......DVLRK......LVALL......LLVYE......FIHRD......SDFGL......KVDVY......
125541650	......NVLGI......LVKLI......LLVYE......VIFRD......SDFGL......KSDIW......

```
125541402    ......EVLSC.....MVLLL.....CLVYE.....LVHRD.....SDVGL.....KSDVY......
125548644    ......TSLSH.....LVSLR.....FLVYE.....IVHRD.....SDFGL.....AGDVY......
125542963    ......KLVSS.....LVRLL.....ILVYE.....IYHRD.....ADFGL.....KSDVY......
125549878    ......EMMSN.....LVELL.....ILVYE.....VIHRD.....ADFGT.....KCDVY......
125549741    ......ETISR.....LVKLY.....LLVYE.....VVHRD.....SDFGL.....KVDVF......
125549741    ......ETISR.....LVKLY.....LLVYE.....VIHRD.....SDFGL.....KVDVF......
125541344    ......VYLGM.....LVKLI.....MLVYE.....VIYRD.....SDFGL.....KSDVY......
125547035    ......EILST.....LVKLV.....ALVYE.....VVHCD.....GDFGL.....SGDVY......
125549881    ......IVLSK.....LVRLF.....MLLYE.....IIHRD.....SDFGM.....KSDIF......
125545961    ......TILSP.....IVPLY.....LLVYE.....IIHRD.....TDFGL.....SVDIY......
125543722    ......ELLSR.....LVGLV.....MLVYE.....IIHRD.....ADFGL.....KSDVY......
125543263    ......EIITR.....LVSLV.....LLVYE.....IIHRD.....ADFGL.....KADVF......
125545791    ......QMLSK.....LVSLI.....ILVYE.....IIHRD.....ADFGL.....KSDVY......
125548698    ......QTIGW.....LVRLL.....LLVYE.....IIHCD.....SDFGL.....KVDVY......
125550483    ......EIISR.....LVSLV.....LLVYD.....IIHRD.....ADFGL.....RSDVF......
125543749    ......EMLSK.....LVSLI.....ILVYD.....IIHRD.....SDFGL.....KSDVY......
125545355    ......SIQSQ.....VVRLL.....-----.....ILHGD.....SDFGI.....KSDLY......
125549738    ......ETISR.....LVTLY.....LLVYE.....IVHRD.....SDFGL.....KVDVF......
125548409    ......ALLSR.....LVRLL.....ILVYE.....IIHRD.....SDFGL.....KSDVY......
125544645    ......DIITR.....LVSLV.....LLVYE.....IIHRD.....ADFGL.....KADVF......
125544270    ......IFLGQ.....LVKLI.....LLVYE.....VIYRD.....SDFGL.....KSDVY......
125547639    ......ELISI.....ILRLI.....LLVYP.....IIHRD.....GDFGL.....KTDIF......
125547107    ......QTISR.....LVELL.....CLVYE.....HLHRD.....ADFGL.....SSDVF......
125549764    ......ATLSR.....LARVV.....ALVLD.....VVHCD.....SDFGT.....KVDVF......
125546584    ......EVLRS.....LMRPL.....ALIFK.....LVHCD.....GDFGS.....GGDVY......
125549870    ......VLIAK.....LVRLL.....LLIYE.....IIHRD.....SDFGM.....KSDIY......
125543139    ......SLLLG.....ILLPF.....MLVYE.....VIHGD.....SDFGL.....KCDIY......
125545862    ......VSLGR.....IVQLL.....LLVYD.....VIHRD.....GDFGI.....SSDVF......
125554197    ......SILSR.....LVTLL.....TLVYE.....VVHGD.....SDFGI.....QSDVY......
125558363    ......QLIAK.....LVNLR.....LLIYE.....IIHRD.....SDFGL.....KSDVF......
125551566    ......TTISA.....LVKLH.....LLVYE.....IVHRD.....SDFGL.....KVDVF......
125556003    ......KCMNK.....AFCWS.....SS--S.....LMYGD.....SDFG-.....ESDVY......
125557234    ......EILSR.....LVSLI.....ILVYE.....IIHRD.....ADFGI.....SSDVY......
125557128    ......VSIGH.....LVQLH.....ILVYD.....ILHRD.....GDFGL.....LTDVF......
125557128    ......VSIGR.....LVQLL.....LLVYE.....VIHRD.....GDFGL.....LTDVY......
125557129    ......VSIGH.....VVKLL.....LLVYD.....VIHRD.....GDFGL.....LTDVF......
125554618    ......EIISM.....LLRLQ.....LLVYP.....IIHRD.....GDFGL.....KTDVF......
125557131    ......VSIGR.....IVQLL.....LLVYD.....VIHRD.....GDFGL.....LTDVF......
125555693    ......QTIGS.....LVRMI.....LLVYE.....IVHLD.....SDFGV.....KADVY......
125559260    ......VLIAK.....LVRLV.....LLVYE.....IIHRD.....SDFGL.....KSDVY......
125553989    ......EVLGK.....LLLML.....CLVYE.....IIHRD.....GDVGL.....KSDVY......
125551809    ......ELLSK.....LVSLI.....ILVYE.....IIHRD.....SDFGL.....KSDVY......
125553530    ......EVLSC.....VVMLI.....LLVYE.....IIHRD.....GDFGL.....KADVY......
125551584    ......ATISA.....LVKLH.....LLVYE.....IVHRD.....SDFGL.....KADVF......
125560509    ......ATISA.....LVKLH.....LLVYE.....IVHRD.....SDFGL.....KADVF......
125551811    ......EMLSK.....LVSLI.....ILVYD.....IIHRD.....SDFGL.....KSDVF......
125555374    ......EIISR.....LVSLV.....MLIYE.....IIHRD.....ADFGL.....RSDVF......
125551812    ......EMLSK.....LVSLI.....ILVYD.....IIHRD.....SDFGL.....KSDVY......
125558635    ......QLIAK.....LVKLL.....ILVYE.....VVHRD.....SDFGM.....KSDVF......
125559049    ......EVLAT.....LVTLY.....ILVYE.....VVHRD.....TDFGL.....KGDVY......
125555970    ......SVISK.....LVRVW.....MLVSE.....LIHCD.....TDFGL.....KVDVY......
125556581    ......Q-LVR.....LIRLL.....ILVYE.....IVHRD.....ADFGS.....KTDVF......
125557127    ......VSIGR.....IVQLL.....LLVYD.....VIHRD.....GDFGL.....LTDVF......
125556085    ......QSIGR.....LVRMV.....MLVFE.....IIHCD.....TDFGI.....KVDVY......
125557141    ......ITLGR.....LVRLL.....LLVYD.....IVHRD.....GDFGL.....ATDVF......
125557639    ......ECLSH.....LVRPI.....LLLHH.....TIHLD.....GEVEI.....PGNVY......
125552887    ......MLIAK.....LVQLI.....MLLYE.....IIHRD.....SDFGM.....KSDVF......
125555899    ......EIISK.....LVSII.....CLLSE.....IVHND.....SNFNL.....KDDIY......
125554210    ......SVIGR.....LVRVW.....LLVSE.....IVHCD.....ADFGL.....KADVF......
125557969    ......VSIGR.....LVQLL.....FLVYD.....VIHRD.....GDFGL.....LTDVF......
125552396    ......EIISH.....LVPLR.....FLVYD.....IYHRD.....ADFGL.....KSDVY......
125558741    ......ILIAK.....LVRLL.....ILVYE.....VVHRD.....SDFGM.....RSDVY......
125555179    ......ELLSR.....LVSLI.....ILVYE.....IIHRD.....ADFGL.....KSDVY......
125557117    ......VSIGQ.....LVQLL.....LLVYD.....VIHRD.....GDFGL.....ATDIF......
125556372    ......NYLGQ.....LVELI.....LLVYE.....IIYRD.....SDFGL.....RSDVY......
125558645    ......QLIAK.....LVRLV.....MLVYE.....IIHRD.....SDFGM.....KSDVF......
125558652    ......VLVAK.....LVRLV.....LLVYE.....IIHRD.....SDFGL.....KSDVF......
125553031    ......DILGH.....IVKLL.....LLVYE.....IVHRD.....ADFGL.....KVDVY......
125556766    ......TTLGR.....IVRLL.....LLLYE.....IIHRD.....ADFGL.....KSDVY......
125552774    ......ELLSR.....LVSLI.....MLVYE.....IIHRD.....ADFGL.....KSDVY......
125554212    ......SIIGR.....LVRIW.....LLVSE.....IVHCD.....ADFGL.....KADVY......
125556578    ......EALSA.....LVPLL.....LLIYP.....IVHRD.....ADFGL.....RGDVY......
125555815    ......EILSQ.....LVLLL.....CLVYE.....IVHRD.....GDVGL.....KSDVY......
```

```
125551463   ......IIQSR.....IVKLI......MLVYE.....ILHGD......SDFGI......KSDVY......
125557733   ......AVLAK.....LTTLV......ALVYE.....VVHRD......SDFGV......AGDVY......
125552821   ......VLVAK.....LVRLL......ILVYE.....IIHRD......SDFGL......KSDVF......
125551438   ......QSIGP.....LVRRI......MLVFE.....IIHCD......TDFGI......KVDVY......
125556684   ......LILSQ.....IVKLL......MLVYE.....ILHGD......SDFGL......KSDVY......
125557123   ......VSIGR.....LVQLL......LLVYE.....IVHRD......GDFGL......LTDIF......
125557134   ......VSIGR.....IVQLH......LLVYD.....VVHRD......GDFGL......LTDVF......
125558649   ......VLVAK.....LVRLL......LLVYE.....IIHRD......SDFGL......KSDVF......
125556573   ......EALSM.....LVPLW......LLIYS.....IVHRD......ADFGL......RGDIY......
125552773   ......RLLSG.....LVRII......CLVNE.....HIHRD......ADFGL......KIDVY......
125552773   ......ELLSR.....LVRLI......MLVYE.....IIHRD......ADFGL......KSDVY......
125558907   ......VSIGR.....IVRLL......LLVYD.....VIHRD......GDFGL......LTDVF......
125559154   ......IFLGQ.....LVKLV......LLVYE.....VIYRD......SDFGL......KSDVY......
125557136   ......VSMGR.....LVQLL......LLVYE.....VIHRD......GDFGL......ATDVF......
125552739   ......ELLSR.....LVALV......MLVYE.....IFHRD......ADFGL......KSDVY......
125558905   ......VSIGR.....LVQLL......LLVYD.....VIHRD......GDFGL......LTDVF......
125551587   ......ATISA.....LVRLH......LLVYE.....IVHRD......SDFGL......KADVF......
125556378   ......GIIAH.....AAQLL......-LVLQ.....IIHRD......SDFGL......KTDVF......
125553222   ......AILSR.....LVLFY......LLVYE.....IVHRD......ADFGL......KSDVY......
125555901   ......QILGH.....IVKLL......LLLYN.....ILHRD......ADFGL......KSDVY......
125559658   ......TYLGQ.....LVKLV......LLVYE.....IIFRD......SDFGL......KSDVY......
125557296   ......IVQSQ.....VVRLI......MLVFE.....IIHGD......SDFGS......KSDVY......
125557120   ......VSIGR.....LVQLL......LLVYA.....VVHRD......GDFGL......LTDVF......
125552467   ......EAIGR.....LVRLL......ILVYE.....VVHRD......SDFGL......RSDVY......
125552252   ......GVLSR.....LVGLL......LLVFE.....VIHRD......SDFGM......KSDVY......
125550949   ......ELLSG.....LVSLI......ILVYE.....IIHRD......SDFGL......KSDVY......
125558399   ......IMQSR.....VAKLF......VLVYK.....VVHGD......AGYGT......EYDVY......
125556820   ......NFLGR.....LVKLL......LLVYE.....IIYRD......SDFGL......KSDVY......
125557841   ......DV---.....-----......MLVYE.....IRHGD......SDIGT......KSDVY......
125560758   ......GIQGH.....TAYLL......-LVFE.....IIHRD......SDFGL......KTDIF......
125553038   ......QILTR.....LVSMI......ALVYE.....LIHRD......ADFGL......KSDVY......
125553042   ......QILTR.....LVSMI......ALVYE.....LIHRD......ADFGL......KSDVY......
125555440   ......ELIAK.....LVRLL......LLVYE.....VVHRD......SDFGM......KSDVF......
125558847   ......QMLSA.....LVPLI......ILVYP.....IIHRD......ADFGF......KSDVF......
125552177   ......RILSS.....LVNLL......LLVVE.....VIHRD......GDFGL......KTDVF......
125558658   ......ALVAK.....LVSFV......LLVYE.....VVHRD......SDFGL......KSDAF......
125560507   ......ATISA.....LVKLH......LLVYE.....IVHRD......SDFGL......KADVF......
125553044   ......QTLTK.....LVALI......ALVYE.....FVHRD......ADFGL......KIDVY......
125558677   ......VLVAK.....LVSLV......LLVYE.....VVHRD......SDFGL......KSDAF......
125557138   ......VSIGR.....LVQLL......LLVYE.....VVHRD......GDFGL......LTDVF......
125560513   ......ATISS.....LVKLH......LLVYE.....IVHRD......SDFGL......KVDVY......
125560513   ......ATISS.....LVKLY......LLVYE.....IVHRD......SDFGL......KVDVF......
125554376   ......EVLSC.....MVLLL......CLVYE.....LVHRD......SDVGL......KSDVY......
125556587   ......GTVGH.....VCALL......-LVFH.....IIHRD......SDFGL......KTDVF......
125551580   ......ATISA.....LVKLH......LLVYE.....IVHRD......SDFGL......KADVF......
125558684   ......VLVAK.....LVRLV......LLVYE.....IVHRD......GDFGL......KSDVF......
125558678   ......VLVAK.....LVSLV......LLVYE.....VVHRD......SDFGL......KSDAF......
125553040   ......QILTR.....LVSMI......ALVYE.....LIHRD......ADFGL......KSDVY......
125553909   ......ETIGS.....LVSLH......LLFYD.....IIHRD......SDFGI......KSDVY......
125557121   ......VSLGR.....LVRLL......MLVYE.....VIHRD......GDFGL......LTDVF......
125558636   ......QLIAK.....LVKLL......MLVYE.....VVHRD......SDFGM......KSDVF......
125557124   ......VSIGR.....LVQLL......LLVYD.....VIHRD......GDFGL......LTDVF......
125551582   ......ATISA.....LVKLH......LLVYE.....IVHRD......SDFGL......KADVF......
125560511   ......ATISA.....LVKLH......LLVYE.....IVHRD......SDFGL......KVDVF......
125558906   ......ASIGR.....LVQLL......LLVYD.....VIHRD......GDFGL......VTEQW......
125560503   ......ATIST.....LVKLH......LLVYE.....IVHRD......SDFGL......KADVF......
125568804   ......STIGR.....V---Y......-----.....ILHFD......ADFGL......KSDVY......
125569757   ......EMLSK.....LVSLI......VLVYD.....IIHRD......SDFGL......KSDVY......
125564768   ......EILSR.....LVTLI......ALVYE.....IVHSD......SGFGV......LSDVY......
125561346   ......AILSP.....IVRLL......LLVYE.....VIHRD......SDFGL......ASDVY......
125564769   ......VVLSR.....IVRLM......CLVYE.....IIHAD......GDFGT......ESDVY......
125564615   ......VLIAK.....LVRLV......LLIYE.....IIHRD......SDFGM......KSDVY......
125568783   ......ATIGR.....IVRLL......ALIYE.....ILHFD......SDFGL......KSDVY......
125563408   ......DFLGN.....LVRLI......LLVYE.....VIYRD......SDFGL......KSDVY......
125568736   ......EIISR.....LVSLV......LLVYD.....IIHRD......ADFGL......RSDVF......
125569574   ......QTVGM.....LVRLL......LLVYE.....IIHCD......ADFGM......KADVY......
125570764   ......ELISQ.....LVKLL......LLVYE.....IIHRD......TDFGL......KADVY......
125568758   ......ATIGR.....VVRLL......ALIYE.....ILHFD......SDFGL......KSDVF......
125563359   ......QSLTK.....LVSLV......ALVYE.....IIHGD......ADFGL......SSDVY......
125563884   ......FFLGQ.....LVKLI......MLVYE.....VIYRD......SDFGL......KSDVY......
125561181   ......ETIGK.....LVPLL......LLVYE.....IIHRD......SDFGM......KGDVY......
125563381   ......QSLTK.....LVSLV......ALAYE.....IIHGD......SDFGL......SSDVY......
```

125569575	......QTVGM.....LVRLL.....LLVYE.....IIHCD.....ADFGM.....KADVY.....
125568796	......STIGR.....VVRLV.....ALVYE.....ILHFD.....ADFGL.....KSDVY.....
125568934	......DILSR.....LVILY.....LLVYE.....IIHRD.....ADFGL.....KSDVY.....
125569725	......DTMGD.....IVPLC.....LLIYE.....VIHRD.....SDFGL.....KGDVY.....
125563305	......RIISQ.....LVQLH.....LLVYE.....ILHGD.....GDFGL.....ESDVY.....
125561696	......EMISL.....LLRLV.....VLVYP.....IIHRD.....GDFGL.....KTDVF.....
125568789	......ATIGR.....IVRLL.....ALIYE.....ILHFD.....SDFGL.....KSDVY.....
125569598	......EIISR.....LVTLV.....LLVYE.....IIHRD.....ADFGL.....KSDVF.....
125564281	......RVQSR.....VVKLL.....KLVYE.....ILHGD.....SDFGL.....LSDVY.....
125562017	......AALGS.....IVRLL.....LLFYT.....ILHGD.....ADFGL.....KSDVY.....
125569087	......EMLSG.....LVSLI.....ILLYE.....VIHRD.....ADFGL.....KSDVY.....
125570098	......IVHSQ.....VVRLL.....MIVME.....VLHGD.....SDFGI.....KSDVY.....
125564353	......SIVSR.....FVEML.....LVAYE.....IVHRD.....ADFNP.....KSDVY.....
125568949	......MSIGR.....IVSLF.....ALLYE.....IIHFD.....ADFGL.....KSDVY.....
125570482	......EVVAS.....LVAIR.....MIVCD.....IIHRD.....ADFGL.....KSDVY.....
125564767	......EILGR.....LVTLI.....ALVYE.....IAHGD.....GDFGI.....QYDVY.....
125563504	......DILSR.....LVTLI.....FVVYE.....VVHRD.....SDFGL.....QSDVY.....
125563368	......QSLTT.....LVSLF.....ALVYE.....IIHGD.....ADFGL.....SSDVY.....
125569371	......EMLGR.....LVKLL.....CLVYE.....VIHRD.....SDFGL.....KSDVY.....
125569917	......ELISI.....ILRLI.....LLVYP.....IIHRD.....GDFGL.....KTDIF.....
125561716	......RTLGQ.....LIRLN.....LILYD.....IIHRD.....SDFGI.....EFDVY.....
125568849	......QILTR.....LVSMI.....ALVYE.....IIHRD.....ADFGL.....KSDVY.....
125568947	......MSIGR.....IVSLF.....ALVYE.....IIHFD.....ADFGL.....KSDVY.....
125568872	......RLLLK.....LVSLL.....MLVYP.....IIHRD.....SDFGM.....KTDVF.....
125568773	......ATIGR.....VLRLL.....TLIYE.....ILHFD.....SDFGL.....KSDVY.....
125563366	......QSLTK.....LVSLV.....ALVYE.....IIHGD.....ADFGL.....SSDVY.....
125568800	......ATIGR.....VVRLV.....ALVYE.....ILHFD.....ADFGL.....KSDVY.....
125562358	......KVLTH.....LVRLI.....-LVYE.....VIHRD.....ADFGL.....KVDVY.....
125563363	......QSLTK.....LVSLI.....ALVYE.....IIHGD.....ADFGL.....SSDVY.....
125564473	......EILGR.....LLSLR.....LIVYD.....IIHRD.....ADFGF.....SCDVY.....
125568763	......QTIGS.....LVRLI.....LLVYE.....IAHLD.....SDFGL.....KADVY.....
125570599	......EAIGK.....IVTLR.....LLIYD.....YIHGD.....SDFGL.....KWDVY.....
125564620	......ILIAK.....LVRLL.....LLIYE.....IIHRD.....ADFGM.....KSDVY.....
125564621	......ILIAK.....LVRLL.....LLIYE.....IIHRD.....ADFGM.....KSDVY.....
125563310	......RIITR.....LVQLL.....MLVYE.....VLHGD.....GDFGL.....ESDVY.....
125568802	......STIGR.....VVRLV.....ALVYE.....ILHFD.....ADFGL.....KSDVY.....
125563333	......GMISA.....LVKLY.....LLVYE.....IVHRD.....SDFGL.....KADVY.....
125562917	......TIIAD.....LVHLQ.....LLVYE.....VIHRD.....GDFGL.....KSDVY.....
125568787	......ATIGR.....VVRLV.....ALVYE.....ILHFD.....ADFGL.....KSDVY.....
125569791	......LMLSL.....LVKLF.....LLIYE.....VIYRD.....SDFGL.....KSDIY.....
125563288	......KIISR.....LVQLI.....LIVYE.....VVHGD.....GDFGL.....ESDVY.....
125568943	......-----.....-----.....ALIYD.....IVHFD.....ADFGL.....KSDVY.....
125570378	......AILSQ.....VVKLF.....LLVYE.....IFHKD.....SDFGA.....KSDVY.....
125570100	......IVHSQ.....VVRLL.....MIVME.....VLHGD.....SDFGI.....KSDVY.....
125563399	......QSLSK.....LVSLV.....ALVYE.....IIHRD.....ADFGL.....SSDIY.....
125564625	......ILIAK.....LVRLL.....LLIYE.....IIHRD.....ADFGM.....KSDVY.....
125574182	......AILSL.....IVKLF.....LLVYD.....IFHRD.....SDFGA.....KSDVY.....
125572164	......EMLSK.....LVSLI.....ILVYD.....IIHRD.....SDFGL.....KSDVY.....
125571646	......ASISR.....VVTLL.....ALIYE.....IVHFD.....SDFGM.....KSDVY.....
125572385	......EIFSC.....ISTAA.....CFPYQ.....IVHRD.....SDFGL.....KTDVY.....
125571955	......DTISR.....LVSLV.....MLVYD.....IIHRD.....SDFGL.....KSDVY.....
125580177	......GTLSS.....VVKLL.....LLVYE.....ILHRD.....ADFGL.....KSDVY.....
125572440	......ELLSR.....LVSLV.....MLVYE.....IFHRD.....ADFGL.....KSDVY.....
125574065	......KILNT.....IVRMA.....LILYE.....IVHRD.....TDFGM.....KSDVY.....
125571518	......TLIGR.....LVRMW.....LLVYE.....LVHCD.....ADFGL.....KVDVY.....
125572983	......ALVSR.....FVDML.....LVAYE.....IVHRD.....ADFNL.....KSDVY.....
125577813	......IVQSQ.....VVRLI.....MLVFE.....IIHGD.....SDFGS.....KSDVY.....
125573396	......ELLSR.....LVSLL.....MLVYE.....IFHRD.....ADFGL.....KSDVY.....
125574228	......AVASH.....ILLPF.....MLVYD.....VVHGD.....ADFGL.....RCDIY.....
125578833	......ELLSR.....VVSLV.....MLVYE.....IIHRD.....SDFGL.....RSDVY.....
125574115	......IIQSR.....IVKLI.....ILVYE.....ILHGD.....SDFGI.....KSDVY.....
125575399	......DALLR.....IVRLL.....VLVLE.....VVHGD.....CDFGS.....KSDVY.....
125575175	......KLISN.....LVRLL.....LLVYE.....HIHRD.....ADFGL.....KVDTY.....
125575514	......LVLSS.....LVSLI.....ILVYE.....IIHRD.....ADFGL.....RSDVY.....
125580531	......ELLSR.....VVPLL.....LLVFE.....ILHRD.....TDLGM.....KSDVF.....
125575764	......AGVGK.....LVNLI.....LLVAE.....KIYHD.....SSFGL.....ESVIY.....
125580852	......ETLSM.....LVPLW.....LLIYS.....IVHRD.....ADFGL.....KGDVY.....
125574045	......IIQSR.....IIKLL.....MLVYE.....IRHGD.....SDFGT.....KSDVY.....
125572290	......LMLSL.....LVKLL.....ILVYE.....VIYRD.....SDFGL.....TSDIY.....
125575914	......TLLSR.....LVSFL.....ILVYE.....IIHRD.....ADFGL.....KSDMY.....
125574114	......IIQSR.....IVKLI.....ILVYE.....ILHGD.....SDFGI.....KSDVY.....
125573489	......NYLGQ.....LVKLV.....LLVYE.....VIYRD.....SDFGL.....KSDVY.....

```
125571972   ......KVLCK.....LVELI.....YLIYE.....YVHRD.....SDFGL.....KNDVY.....
125571074   ......ESIGH.....LVKLY.....CLVYE.....VIHRD.....SDFGL.....KSDVY.....
125575504   ......ATIGR.....LVSLL.....LLVYE.....IVHRD.....SDFGL.....RSDVY.....
125580838   ......ELLNN.....MVHLL.....CLVYE.....FVHRD.....SDVGL.....KSDVY.....
125571638   ......AILSR.....LVMFY.....LLVYE.....IVHRD.....ADFGL.....KSDVY.....
125578000   ......DILPT.....IIRLL.....SLVYE.....VIHRD.....ADFGL.....AMDVY.....
125574729   ......DYLGQ.....LVKLI.....LLVYE.....VIYRD.....SDFGL.....KADVY.....
125572383   ......QILSK.....LVTFL.....ALVYD.....IVHRD.....SDFGL.....KADVY.....
125571642   ......ASISR.....VVTLL.....VLIYE.....IVHFD.....SDFGM.....KSDVY.....
125576653   ......GILID.....IVKLI.....LLCYE.....IIHLD.....ADFGL.....KSDIF.....
125578397   ......SVIGR.....LVRMW.....ILVYE.....IIHCD.....TDFGL.....KVDVY.....
125571769   ......EMLSR.....LVKLI.....CLVYE.....VIHRD.....SDFGL.....KSDVY.....
125574064   ......KILNT.....IVRMA.....LILYE.....IIHRD.....TDFGM.....KSDVY.....
125570996   ......DFLGN.....LVKLV.....LLVYE.....VIYRD.....SDFGL.....KSDVY.....
125577546   ......TSQAR.....LVRLV.....MLVLE.....VVHGD.....SDFGS.....KSDVY.....
125577094   ......KLISN.....LVRLL.....LLVYE.....IIHRD.....ADFGL.....KVDTY.....
125580850   ......EALTM.....LVPLW.....LLIYS.....IVHRD.....ADFGL.....RGDIY.....
125574376   ......LLVAK.....LAKLL.....LLVYE.....IIHRD.....SDFGL.....KVDVY.....
125578699   ......EIVSS.....AMPLT.....MLVYD.....VIHRD.....CDFGL.....KIDVY.....
125577462   ......VLVSK.....LVRLV.....LLVYE.....IIHRD.....SDFGL.....KSDVF.....
125573812   ......IIQSQ.....IIKLL.....MLVYE.....IRHGD.....SDFGL.....KSDVY.....
125579554   ......SVIGR.....LVRMW.....LLVFE.....LIHCD.....ADFGL.....KVDVY.....
125576552   ......SSLGL.....LVKLI.....LLVYE.....IIHCD.....ADFGM.....KVDVY.....
125574880   ......IFLGH.....LVRLV.....LLVYE.....VIYRD.....SDFGL.....MSDVY.....
125575602   ......AILSR.....LVTLI.....ALVYE.....VVHGD.....SDFGI.....QSDTY.....
125575605   ......AVLSR.....LVTLV.....-LVYE.....VIHGD.....GDFGI.....RSDIY.....
125577504   ......RIHAQ.....IVKLI.....MMVTE.....ICHGD.....SDFGI.....KSDVY.....
125576747   ......ELLSR.....LVSLV.....MLVYE.....IVHRD.....SDFGL.....KSDVY.....
125571252   ......SIVSR.....FVEML.....LVAYE.....IVHRD.....ADFNP.....KSDVY.....
125578254   ......EAIGH.....LVRLL.....MLVYE.....VVHRD.....SDFGL.....KSDVY.....
125583945   ......EVLSC.....MVLLL.....CLVYE.....LVHRD.....SDVGL.....KSDVY.....
125582971   ......VILSQ.....VVKIF.....LLVYE.....AIHRD.....SDFGA.....KSDVY.....
125582972   ......AILSQ.....VVKLF.....LLVYE.....AIHRD.....SDFGA.....KSDVY.....
125586129   ......ELLSR.....LVGLV.....MLVYE.....IIHRD.....ADFGL.....KSDVY.....
125584085   ......AILSQ.....VVKLF.....LLIYE.....IIHRD.....SDFGA.....KSDVF.....
125586821   ......KLIAK.....LVRLL.....MLIYE.....IIHRD.....SDFGV.....KSDVF.....
125582461   ......EAIGR.....LVRLL.....MLVYE.....VVHRD.....SDFGL.....TSDVY.....
125581758   ......ASIGR.....LVQLL.....LLVYD.....VIHRD.....GDFGL.....LTDVF.....
125584817   ......KGVGK.....LANLI.....LLVAE.....LYH-D.....SCFGL.....ESVIF.....
125585078   ......NYLGQ.....LVKLI.....LLVYE.....VIYRD.....SDFGL.....KADVY.....
125587711   ......AVLTK.....LVSLL.....ILVYE.....FIHRD.....ADFGL.....KADVF.....
125584140   ......EMLSR.....LVKLI.....CLVYE.....VIHRD.....TDFGL.....KSDVY.....
125583010   ......ESITE.....LVRLL.....ILIYE.....IVHRD.....GDFGI.....KADVY.....
125590049   ......AT-NN.....IVGKG.....MLIYE.....ILHGD.....SDFGC.....KNDVY.....
125585477   ......EAIGK.....LVGLV.....MLVYE.....VVHRD.....SDFGM.....SSDIY.....
125587625   ......ELISV.....LLRLI.....LLVYP.....IIHRD.....GDFGL.....RTDVF.....
125584087   ......FILSQ.....VVKLL.....MLVYE.....ILHGD.....SDFGL.....KSDVY.....
125583260   ......KILQK.....LVKLE.....YLVYE.....VVHKD.....ANFGL.....KMDVF.....
125586947   ......DIITR.....LVSLV.....LLVYE.....IIHRD.....ADFGL.....KADVF.....
125587761   ......DLFAK.....LVPIM.....KLVYI.....MVHRD.....GSLSE.....SYDVY.....
125581419   ......ITIGS.....LVRLI.....LLVYE.....IAHLD.....SDFGL.....KADIY.....
125581375   ......TTLGM.....IIILL.....LILYE.....IIHGD.....SGFSY.....KVDVY.....
125583879   ......VYLGM.....LVKLI.....MLVYE.....VIYRD.....SDFGL.....KSDVY.....
125584197   ......EAVGR.....LVTLV.....AVVHE.....TVHGD.....AGLGM.....ERDVY.....
125588094   ......DIISR.....LVSLV.....VLVYE.....IIHRD.....ADFGL.....KSDVF.....
125581756   ......VSIGR.....IVQLL.....LLVYD.....VVHRD.....GDFGL.....LTDVF.....
125585628   ......SLLLG.....ILLPF.....MLVYE.....VIHGD.....SDFGL.....KCDIY.....
125586615   ......IFLGQ.....LVKLI.....LLVYE.....VIYRD.....SDFGL.....KSDVY.....
125584170   ......NVLGI.....LVKLI.....LLVYE.....VIFRD.....SDFGL.....KSDIW.....
125589318   ......AAVAR.....LVRLR.....LLVYD.....IIHRD.....GDFGL.....KSDVF.....
125590050   ......NNLMR.....IIGLV.....ALCFE.....IVHLD.....ADFGL.....KFDVF.....
125581322   ......DILSQ.....LVLLL.....CLVYE.....IVHRD.....GDAGF.....KSDLF.....
125590213   ......LMLSL.....LVNLV.....LLVYE.....VIYRD.....SDFGL.....KSDVY.....
125585139   ......IFLGQ.....LVKLI.....LLVYE.....VIYRD.....SDFGL.....KSDVY.....
125590426   ......EAIGR.....LVRLL.....MLVYE.....VVHRD.....SDFGL.....TSDVY.....
125589689   ......LILSR.....IVKLL.....VLVYE.....HFHGD.....SDFGC.....KSDVY.....
125585837   ......SVLSK.....LVKLI.....LLVYE.....VINRD.....SDFGL.....QSDIY.....
125586150   ......EMLSK.....LVSLI.....ILVYD.....IIHRD.....SDFGL.....KSDVY.....
125589727   ......AILSQ.....IVRLF.....LLVYD.....VFHRD.....SDFGA.....KSDVY.....
125584762   ......EAIGR.....LVRLL.....MLVYE.....VVHRD.....SDFGL.....RSDVY.....
125585693   ......NVISN.....LVKLI.....LLVYE.....IVHRD.....GDFGL.....KADIY.....
125588735   ......EVLRS.....IMRPL.....ALIFK.....LVHCD.....GDFGS.....D-DTF.....
125581428   ......ELIGL.....LLRLY.....LLVYP.....IIHRD.....GDFGL.....KTDVY.....
125581408   ......GIISA.....LVRLF.....LLIYE.....IIHRD.....SDFGL.....KADVY.....
```

```
125590063    ......LILSR......IVKLL......VLVYE......HLHGD......SDFGK......KSDVY......
125584804    ......NFLGR......LVKLL......LLVYE......IIYRD......SDFGL......KSDVY......
125589327    ......DVLRK......LVALL......LLVYE......FIHRD......SDFGL......KVDVY......
125582968    ......AILSQ......VVKLF......LLVYE......AIHRD......SDFGA......KSDVY......
125594679    ......ELLSR......LVSLI......MLVYE......IIHRD......ADFGL......KSDVY......
125592117    ......--VSY......PFALL......CLV-P......LYH-D......SCFGL......ESVIY......
125594937    ......QILTR......LVSMI......ALVYE......LIHRD......ADFGL......KSDVY......
125598857    ......EALSQ......LVSLR......LLIYS......IIHRD......ADFGL......KGDVY......
125596893    ......RTVGH......LLPLA......YLVYE......IIHRD......ADFGL......KCDVY......
125593517    ......ATISA......LVKLH......LLVYE......IVHRD......SDFGL......KADVF......
125592563    ......NYLGQ......LVRLV......LLVYE......VIYRD......SDFGL......KSDVY......
125596326    ......EVLSC......MVLLL......CLVYE......LVHRD......SDVGL......KSDVY......
125600309    ......QFLAV......LVKLI......LLVYE......VIYRD......SDFGL......RSDVW......
125600559    ......VLVAK......LVRLV......LLVYE......IIHRD......SDFGL......KSDVF......
125593525    ......ATISA......LVRLH......LLVYE......IVHRD......SDFGL......KADVF......
125591635    ......ETISR......LVKLY......LLVYE......VVHRD......SDFGL......KVDVF......
125598497    ......QFLGV......LVRLV......LLVYE......VIYRD......SDFGL......KSDVW......
125591632    ......ETISR......LVTLY......LLVYE......IVHRD......SDFGL......KVDVF......
125591656    ......ATLSR......LARVV......ALVLD......VVHCD......SDFGT......KVDVF......
125592704    ......RLVSQ......LVKLL......LLVYE......IIHRD......ADFGL......KADIY......
125594939    ......QTLTK......LVALI......ALVYE......FVHRD......ADFGL......KIDVY......
125593507    ......TTISA......LVKLH......LLVYE......IVHRD......SDFGL......KVDVF......
125593740    ......EMLSK......LVSLI......ILVYD......IIHRD......SDFGL......KSDVY......
125591902    ......GTIGR......LVRLY......ALVYE......IVHYD......ADFGL......KCDVY......
125600768    ......QMLSA......LVPLI......ILVYP......IIHRD......ADFGF......KSDVF......
125594295    ......EIISH......LVPLR......FLVYD......IYHRD......ADFGL......KSDVY......
125600654    ......ILIAK......LVRLL......ILVYE......VVHRD......SDFGM......KSDIY......
125600822    ......VSIGR......LVQLL......LLVYD......VIHRD......GDFGL......LTDVF......
125599841    ......VSIGR......LVQLL......FLVYD......VIHRD......GDFGL......LTDVF......
125598569    ......NFLGR......LVKLL......LLVYE......IIYRD......SDFGL......KSDVY......
125592680    ......AFLSR......LVGFV......LLVYE......IIHRD......SDFGL......KSDVY......
125591881    ......VILSQ......VVKLY......LLVYE......VLHRD......SDFGA......KSDIY......
125600543    ......QLIAK......LVKLL......MLVYE......VVHRD......SDFGM......KSDVF......
125595924    ......ETIGS......LVSLH......LLFYD......IIHRD......SDFGI......KSDVY......
125600574    ......LLLAE......LVRLH......LLVYE......IIHRD......ADFGL......KIDIF......
125600550    ......QLIAK......LVRLV......MLVYE......IIHRD......SDFGM......KSDVF......
125591757    ......AVMAG......LLRLL......ILVYA......VIHRD......ADFGT......KCDVY......
125591756    ......EMMSN......LVELL......ILVYE......VIHRD......ADFGT......KCDVY......
125598331    ......EALSA......LVPLL......LLIYP......IVHRD......ADFGL......RGDVY......
125593734    ......ELLSK......LVSLI......ILVYE......IIHRD......SDFGL......KSDVY......
125597087    ......ELLSR......LVSLI......ILVYE......IIHRD......ADFGL......KSDVY......
125600585    ......VLVAK......LVSLV......LLVYE......VVHRD......SDFGL......KSDAF......
125600587    ......LLVAK......LVRLI......ILAYE......IVHRD......SDFGL......KLDVY......
125600780    ......KIISR......LVQLV......LLVYE......VVHRD......GDFGL......ETDVY......
125600824    ......VSIGR......IVRLL......LLVYD......VIHRD......GDFGL......LTDVF......
125598337    ......GTVGH......VCALL......-LVFH......IIHRD......SDFGL......KTDVF......
125597652    ......EILSQ......LVLLL......CLVYE......IVHRD......GDVGL......KSDVY......
125591758    ......IVLSK......LVRLF......MLLYE......IIHRD......SDFGM......KSDIF......
125597718    ......QILGH......IVKLL......LLLYN......ILHRD......ADFGL......KSDVY......
125591637    ......ETISR......LVKLY......LLVYE......VVHRD......SDFGL......KVDVF......
125594641    ......ELLSR......LVALV......MLVYE......IFHRD......ADFGL......KSDVY......
125600565    ......ALVAK......LVSFV......LLVYE......VVHRD......SNFGL......KSDAF......
125599003    ......VSLGR......LVRLL......MLVYE......VIHRD......GDFGL......LTDVF......
125594934    ......QILTR......LVSMI......ALVYE......LIHRD......ADFGL......KSDVY......
125593530    ......ATISA......LVILH......LLVYE......IVHRD......SDFGL......KADIF......
125594723    ......VLVAK......LVRLL......ILVYE......IIHRD......SDFGL......KSDVF......
125598516    ......TTLGR......IVRLL......LLLYE......IIHRD......ADFGL......RRPVG......
125594364    ......EAIGR......LVRLL......ILVYE......VVHRD......SDFGL......RSDVY......
125592730    ......SMVSR......VVELL......VLAYE......IIHRD......ADFDL......KSDVY......
125596194    ......LILSQ......IVKLL......MLVYE......IIHGD......SDFGA......KSDVY......
125600556    ......VLVAK......LVRLL......LLVYE......IIHRD......SDFGL......KSDVF......
125593521    ......ATISA......LVKLH......LLVYE......IVHRD......SDFGL......KADVF......
125596143    ......SILSR......LVTLL......TLVYE......VVHGD......SDFGI......QSDVY......
125597318    ......ELIAK......LVRLL......LLVYE......VVHRD......SDFGM......KSDVF......
125603218    ......AILSP......IVRLL......LLVYE......VIHRD......SDFGL......ASDVY......
125605363    ......QSLTK......LVSLV......ALVYE......IIHGD......ADFGL......SSDVY......
125603476    ......AAIGA......LPRLL......LLVYE......PIHGD......TDYGL......SSDVY......
125606039    ......LILSQ......IVKLL......ILVYE......ILHGD......SDFGA......KSD--......
125604877    ......TIIAD......LVHLQ......LLVYE......VIHRD......GDFGL......KSDVY......
125602520    ......ATISA......LVKLH......LLVYE......IVHRD......SDFGL......KADVF......
125602044    ......KVISR......LVQLV......LLVYE......VVHRD......DDFGL......ESDVY......
125603918    ......AILSQ......VVKLL......LLVYE......IFYRD......SDFRA......KSDVF......
125605325    ......GMISA......LVKLY......LLVYE......IVHRD......SDFGL......KADVY......
```

```
125606548   ......TLIAK.....LVRLL.....LLIYE.....IIHRD.....ADFGM.....KTDIY.....
125603066   ......ETIGK.....LVPLL.....LLVYE.....IIHRD.....SDFGM.....KGDVY.....
125606551   ......ILIAK.....LVRLL.....LLIYE.....IIHRD.....ADFGM.....KSDVY.....
125606551   ......ILIAK.....LVRLL.....LLIYE.....IIHRD.....ADFGM.....KSDVY.....
125606551   ......ILIAK.....LVRLL.....LLIYE.....IIHRD.....ADFGM.....KSDVY.....
125605365   ......QSLTT.....LVSLF.....ALVYE.....IIHGD.....ADFGL.....SSDVY.....
125602518   ......STIST.....LVKLY.....LLVYE.....IVHRD.....SDFGL.....KADVF.....
125605394   ......QSLTK.....LVSLV.....ALVYE.....IIHRD.....ADFGL.....NSDVY.....
125606555   ......ILIAK.....LVRLL.....LLIYE.....IIHRD.....ADFGM.....KSDVY.....
125603569   ......EMISL.....LLRLV.....LLVYP.....IIHRD.....GDFGL.....KTDVF.....
125602515   ......ATISA.....LVKLH.....LLVYE.....IVHRD.....SDFGL.....KADVF.....
125602524   ......ATISS.....LVKLH.....LLVYE.....IVHRD.....SDFGL.....KVDVY.....
125602524   ......ATISS.....LVKLY.....LLVYE.....IVHRD.....SDFGL.....KVDVF.....
125605855   ......FFLGQ.....LVKLI.....MLVYE.....VIYRD.....SDFGL.....KSDVY.....
125606631   ......EV---.....-----.....LLVYE.....IIHRD.....SDFGA.....KSDIY.....
125605414   ......DFLGN.....LVRLI.....LLVYE.....VIYRD.....SDFGL.....KSDVY.....
125606298   ......KVLTH.....LVRLI.....FLVYE.....YVHRD.....ADFGL.....KVDVY.....
125605067   ......ETIGK.....LVPLL.....LLVYE.....IIHRD.....SDFGM.....KGDVY.....
125604168   ......KVLTH.....LVRLI.....-LVYE.....VIHRD.....ADFGL.....KVDVY.....
125603586   ......QTLGQ.....LIRLN.....LILYD.....IIHRD.....SDFGI.....EFDVY.....
125605359   ......QSLTK.....LVSLI.....ALVYE.....IIHGD.....ADFGL.....SSDVY.....
125605386   ......QSLTK.....LVSLV.....ALAYE.....IIHGD.....ADFGL.....SSDVY.....
125602726   ......GIQGH.....TAYLL.....-LVFE.....IIHRD.....SDFGL.....KTDIF.....
125606036   ......-----.....-----.....MLVYE.....IIHGD.....SDFGL.....KSDVY.....
125606700   ......VVLSR.....IVRLM.....CLVYE.....IIHAD.....GDFGT.....ESDVY.....
125603238   ......TILSQ.....VVKLL.....LLVYE.....IFHRD.....SDFGT.....KSDVF.....
125605381   ......ESLTT.....LVLLV.....GLVYE.....IVHRD.....SDFGL.....SSDVF.....
125605400   ......QSLTK.....LVSLV.....ALVYE.....IIHRD.....ADFGL.....NNDVY.....
125601175   ......VLIAK.....LVRLV.....LLVYE.....IIHRD.....SDFGL.....KSDVY.....
125606546   ......VLIAK.....LVRLV.....LLIYE.....IIHRD.....SDFGM.....KSDVY.....
125606547   ......LLIAK.....LVRLV.....LLIYE.....VIHRD.....SDFGM.....KSDVY.....
125601066   ......IFLGQ.....LVKLV.....LLVYE.....VIYRD.....SDFGL.....KSDVY.....
125605298   ......RIISQ.....LVQLH.....LLVYE.....ILHGD.....GDFGL.....ESDVY.....
125606421   ......EILGR.....LLSLR.....LIVYD.....IIHRD.....ADFGF.....SCDVY.....
125606634   ......AILSQ.....VVKLF.....LLVYE.....IIHRD.....SNFGA.....KSDVF.....
125602306   ......TSQAR.....LVRLV.....MLVLE.....VVHGD.....SDFGS.....KSDVY.....
125606632   ......AILSQ.....VVKLF.....LLVYE.....IIHRD.....SDFGA.....KSDVF.....
125606632   ......AILSQ.....VVKLF.....LLVYE.....IIHRD.....SDFGA.....KSDVF.....
125605500   ......DILSR.....LVTLI.....FVVYE.....VVHRD.....SDFGL.....QSDVY.....
125605348   ......QSLTK.....LVSLI.....ALVYE.....IIHRD.....ADFGL.....SSDVY.....
125605280   ......KIISR.....LVQLI.....LIVYE.....VVHGD.....GDFGL.....ESDVY.....
125597532   ......QTIGS.....LVRLI.....LLVYE.....IAHLD.....SDFGL.....KVDVY.....
125599702   ......DV---.....-----.....MLVYE.....IRHGD.....SDFGT.....KSDVY.....
125595125   ......ASISR.....VVTLL.....ALIYE.....IVHFD.....SDFGL.....KSDVY.....
125592024   ......ETLSR.....LVLLE.....LLIYA.....ILHRD.....ADFGL.....KGDVY.....
125593733   ......ETLSK.....LVSLI.....ILVYE.....IIHRD.....SDFGL.....RSDVF.....
125596159   ......SVIGR.....LVRVW.....LLVSE.....IVHCD.....ADFGL.....KADVF.....
125594927   ......DILGH.....IVKLL.....LLVYE.....IVHRD.....ADFGL.....KVDVY.....
125600584   ......VLVAK.....LVSLV.....LLVYE.....VVHRD.....SDFGL.....KSDAF.....
125594786   ......MLIAK.....LVQLI.....MLLYE.....IIHRD.....SDFGM.....KSDVF.....
125590955   ......EMLSR.....LVKLI.....CLVFE.....VIHRD.....ADFGL.....KSDVY.....
125594166   ......GVLSR.....LVGLL.....LLVFE.....VIHRD.....SDFGM.....KSDVY.....
126013404   ......NYLGQ.....LVRLV.....LLVYE.....VIYRD.....SDFGL.....KSDVY.....
126340173   ......KILAK.....LVQLL.....CLVYV.....HVHRD.....SDFGL.....KSDIY.....
146324309   ......EVLSL.....ITSLL.....DMAVP.....IVHGD.....SDFGV.....RFDAY.....
126843151   ......ETIGK.....LVPLL.....LLVYE.....IIHRD.....SDFGM.....KGDVY.....
126843180   ......ETIGK.....LVPLL.....LLVYE.....IIHRD.....SDFGM.....KGDVY.....
126843144   ......ETIGK.....LVPLL.....LLVYE.....IIHRD.....SDFGM.....KGDVY.....
134142350   ......DTLGK.....IVKLW.....LLVYE.....IVHRD.....ADFGV.....KSDIY.....
134142356   ......ETLGR.....IVKLW.....LLVYE.....IVHRD.....ADFGV.....KSDIY.....
134142354   ......DTLGK.....IVKLW.....LLVYE.....IVHRD.....ANSPL.....KSDIY.....
134142352   ......ETLGR.....IVKLW.....LLVYE.....IVHRD.....ADFGV.....KSDIY.....
145324006   ......DILSR.....LVSLI.....FLVYE.....IVHRD.....SDFGL.....QSDIY.....
145334855   ......VSMRC.....LVPLF.....LLVSE.....VLHRD.....GDFGM.....GTDVY.....
145334223   ......NIISS.....ISPLL.....ISVYN.....VIHRD.....SDFGL.....KVDVY.....
145335397   ......VVISK.....LVKLL.....MLVYE.....IIHRD.....SDFGL.....KSDVF.....
145335397   ......VVISK.....LVKLF.....MLVYE.....IIHRD.....SDFGL.....KSDVF.....
145357530   ......ELLSR.....LVSLI.....MLVYE.....VFHRD.....ADFGL.....KSDVY.....
145358545   ......MCLGK.....LVKLI.....LLVYE.....IIYRD.....SDFGL.....KSDVY.....
145351606   ......DTISR.....LVSLV.....LLVYE.....IIHRD.....SDFGL.....KSDVY.....
145580038   ......KVMAK.....LVELL.....CLVYV.....HIHRD.....SDFGL.....KSDIY.....
166008042   ......EVLSC.....MVLLL.....CLVYE.....LVHRD.....SDVGL.....KSDVY.....
145698410   ......RLIAR.....LVRIL.....MLIYE.....IIHRD.....SDFGM.....KSDVF.....
```

```
145666466    ......ESLGK.....LTVLR.....LLVYD.....MVHGD.....SEFGL.....ESDAY......
147767572    ......NLISG.....LVKLL.....LLVYE.....IIHRD.....ADFGL.....KVDVY......
147773745    ......ATIGR.....VVKLV.....ALIYE.....ILHFD.....SDFGL.....KADVY......
147766422    ......KTLGS.....IVRFL.....LLMYD.....IVHRD.....ADFGL.....KSDVY......
147768199    ......KILST.....IVKLL.....LLVYE.....IIHRD.....ADFGL.....KIDVY......
147765964    ......KIISR.....LVQLI.....LLVYE.....VLHRD.....GDFGL.....QSDIY......
147765961    ......VILCQ.....VVKLL.....LLVYE.....SIHRD.....ADFGI.....KSDVY......
147769927    ......ASISR.....IVSLL.....ALIYE.....ILHLD.....SDFGL.....KSDVY......
147768374    ......ATIGR.....VVRLV.....ALIYD.....ILHFD.....SDFGL.....KADVY......
147767540    ......ATISA.....LVKLY.....SLVYE.....IVHRD.....SDFGL.....KADVF......
147773344    ......VLIAK.....L----.....CF--......IIHRD.....SDFGL.....KSDVF......
147765625    ......NLISG.....LVKLL.....LLVYE.....IIHRD.....ADFGL.....KADVY......
147772215    ......IFLGQ.....LVKLI.....LLVYE.....VIYRD.....SDFGL.....KSDVY......
147765769    ......ELLSR.....VVSLI.....ILIYE.....IIHRD.....GDFGL.....KSDVY......
147765770    ......ELLSR.....VVGLV.....MLVYE.....IIHRD.....ADFGL.....KSDVY......
147765626    ......DIIGS.....LKDT-.....----......IIHRD.....ADFGL.....KVDVY......
147766865    ......GMISA.....LVKLY.....LLIYE.....IVHRD.....SDFGL.....KADVY......
147766839    ......KIFCR.....LVQLM.....LLVYE.....VVHRD.....RDFGL.....ESDMY......
147768020    ......MLIAK.....LVKVL.....ILIYE.....VIHRD.....SDFGM.....KSDVF......
147770821    ......VILSQ.....VVKLL.....LLVYE.....IYHRD.....SDFGI.....KSDVY......
147770298    ......TIIAR.....LVRLW.....ILVYE.....LLHCD.....SDFGL.....KADVY......
147770958    ......ATIGR.....VVRLV.....ALVYE.....ILHFD.....SDFGL.....KADVY......
147770086    ......SALTD.....LVTLQ.....FLVYD.....IIHRD.....SDFGL.....KSDVY......
147769069    ......ASIGR.....VIRLL.....ALIYE.....ILHLD.....SDFGL.....KSDVY......
147772798    ......DILSK.....LVTLI.....ALIYE.....IVHGD.....SDFGI.....KSDVY......
147769591    ......RLIAK.....LVRIL.....MLIYE.....MVHRD.....SDFGT.....KTDVY......
147772916    ......QILTR.....LVSLY.....LLVYE.....VVHRD.....ADFGL.....KSDVY......
147765333    ......NIISS.....LVRLL.....LLVYE.....IIHRD.....ADFGL.....KXDVY......
147767461    ......ATIGR.....VVRLV.....ALVYD.....ILHFD.....SDFGL.....KVDVY......
147769070    ......VTIGR.....VIRLL.....ALVYE.....ILHLD.....SDFGL.....KSDVY......
147768429    ......NIMSS.....IAPLL.....ISVYE.....VIHRD.....ADFGL.....KIDVY......
147784827    ......ATIGR.....IVQLV.....ALIYD.....ILHFD.....SDFGL.....KADVY......
147778645    ......TVISQ.....FVGLL.....HLVYE.....IIHCD.....SDFGL.....KVDVY......
147780497    ......TLLSR.....LVQFL.....MLVYE.....IIHRD.....SDFGL.....KSDVY......
147784474    ......AAISA.....LVKLY.....LLVYE.....IVHRD.....SDFGL.....KADVF......
147778584    ......IVLTK.....LVSLI.....ILVYE.....IIHRD.....ADFGL.....KSDVY......
147781106    ......ILIAK.....LVRLL.....----......IIHRD.....SDFGI.....KSDVF......
147780241    ......EAIGH.....LVRLL.....MLVYE.....VVHRD.....SDFGL.....KSDVY......
147778506    ......EMLSQ.....LVSLI.....ILVYE.....IIHRD.....SDFGL.....KSDVY......
147777971    ......AAISA.....LVKLY.....LLVYE.....TVHRD.....SDFGL.....KADAF......
147783438    ......IFLGQ.....LVKLI.....VLVYE.....VIYRD.....SDFGL.....RSDVY......
147780897    ......VLIAK.....LVRLL.....CML--.....IIHRD.....SDFGL.....KSDVF......
147784082    ......TLIAK.....LVRLL.....MLVYE.....IIHRD.....SDFGL.....KSDVY......
147783087    ......SSIGT.....LVRLR.....CLVFD.....IIHCD.....ADFGL.....KADVF......
147783665    ......LILSQ.....IVKLL.....LLVYE.....ICHRD.....SDFGL.....KSDVY......
147782227    ......LMLSL.....LVNLI.....LLVYE.....VIYRD.....SDFGL.....KSDIY......
147776918    ......DILSR.....LVSLI.....FLVYE.....IVHRD.....SDFGL.....QSDVY......
147778593    ......HAFSK.....VVKLL.....FLVYE.....IIHRD.....GDFGL.....KTDVY......
147782422    ......TIIGR.....LVRLL.....LLVYE.....IIHGD.....SDFRL.....EADVY......
147781733    ......GMISA.....LVRLY.....ILVYE.....IVHRD.....SDFGL.....KADVY......
147777060    ......FFAGR.....IVPVI.....ILVYE.....AIHGD.....GDFGL.....KCDVY......
147791345    ......RLLLK.....LVRLF.....MLVYE.....IIHRD.....SDFGL.....KIDVF......
147792365    ......ASISK.....IVTLL.....ALIYE.....IVHFD.....SDFGL.....KSDVY......
147790445    ......RILCQ.....LVRLL.....IMIYE.....IYHRD.....SDFGL.....KSDVY......
147793834    ......GTIGR.....VVXLI.....ALIYE.....ILHFD.....SDFGL.....KSDVY......
147793833    ......ATIGR.....VVKLV.....ALIYD.....ILHFD.....SDFGL.....KADVY......
147787085    ......EAIGK.....IVTLR.....LLIYD.....YVHGD.....SDFGL.....KWDVY......
147787336    ......FILSQ.....IVRLL.....LLVYE.....SIHRD.....SDFGL.....KSDVY......
147788840    ......TLIAK.....LVKLL.....----......IIHRD.....SDFGM.....KSDVY......
147789396    ......ELLSR.....LVSLI.....MLVYE.....IFHRD.....ADFGL.....KSDVY......
147795288    ......GMISA.....LVKLY.....SLVYE.....IVHRD.....SDFGL.....KADVY......
147791818    ......STIGK.....LVRMW.....LVVYE.....VIHCD.....ADFGL.....KVDVY......
147788621    ......ASISR.....IVTLL.....ALIYE.....ILHLD.....CDFGL.....KSDVY......
147790217    ......EGLSR.....LVCLF.....YLVYE.....IVHRD.....SDFGL.....ASDIY......
147790481    ......NLISQ.....LVKLL.....LLVYE.....IIHRD.....ADFGL.....KADVY......
147792868    ......ILILK.....LVRLL.....ILVYE.....VIHRD.....SDFGM.....KSDVF......
147794285    ......ATIGR.....VVRLV.....ALVYD.....ILHFD.....SDFGL.....KADVY......
147789104    ......GILSQ.....VIQLL.....LLVYE.....IVHRD.....CDFGA.....KSDVY......
147797614    ......KHLMR.....VTSLI.....GLIYE.....IIHRD.....ADFGL.....KSDVF......
147801082    ......ATIGR.....VVQLI.....ALVYD.....ILHFD.....SDFGL.....KADVY......
147802844    ......EILSR.....LVNLL.....KLVVE.....VIHRD.....GDFGL.....KSDVF......
147801768    ......IILSR.....LVSLI.....ILVYE.....IIHRD.....ADFGL.....KSDIY......
147804692    ......ELLAK.....LVKLL.....LIITE.....IIHRD.....ADFGF.....KSDVY......
147804673    ......TLIAK.....LVRLL.....MLVYE.....IIHRD.....SDFGL.....KSDVY......
```

147802484	......ETLGS.....IVKLY.....LLVYE.....IIHRD.....CRLRH.....KCDVY.....
147798220	......RLIVN.....LIRLL.....MLIYE.....MVHGD.....SDFGT.....KTDVY.....
147795963	......ASISR.....IVSLL.....ALIYE.....ILHLD.....SDFGL.....KSDVY.....
147801125	......ELLAR.....LVALR.....FLMYE.....LCHRD.....ADFGL.....KSDVY.....
147798319	......IVLSK.....LVSLI.....ILVYE.....IIHRD.....ADFGL.....KSDVY.....
147798321	......KVLSK.....LVSLI.....ILVYE.....IIHRD.....SDFGL.....KSDVY.....
147799241	......IYISK.....LVKLL.....MLIYE.....IIHRD.....SDFGM.....KSDVF.....
147801769	......LVLSK.....LVSLV.....ILVYE.....IIHRD.....ADFGL.....KSDVY.....
147798019	......AVLTK.....LVALL.....LLVYE.....FIHRD.....ADFGL.....KVDVY.....
147797909	......EVLSC.....MVLLL.....CLVYE.....LVHRD.....SDVGL.....KSDVY.....
147797625	......GIIAH.....TAKLI.....-LVLQ.....IIHRD.....CDFGL.....KTDIF.....
147799284	......SIIAC.....LVQLQ.....LLVYE.....VIHRD.....GDFGL.....KTDVF.....
147799980	......GMISA.....LVKLY.....LLIYE.....IVHRD.....SDFGL.....KADVY.....
147797812	......WGVGK.....LANLI.....LLVAE.....LYH-D.....SCFGM.....ESVIF.....
147805140	......EALSR.....LVSLQ.....LLIYS.....VVHRD.....ADFGL.....KGDVY.....
147814876	......GILSQ.....VIQLL.....LLVYE.....IIHRD.....CDFGA.....KSDVY.....
147811982	......GAIXR.....LVQLL.....LLVYE.....AIHGD.....SDFGL.....KVDVY.....
147815100	......SSLGR.....LVSLR.....MLVYD.....VLHRD.....GDFGL.....QTDVF.....
147807566	......GMISA.....LVKLY.....LLIYE.....IVHRD.....SDFGL.....KADVY.....
147812634	......ASFSR.....IVTLV.....ALIYE.....ILHFD.....SDFGL.....KSDVY.....
147806197	......NMISN.....LVELI.....ILVYE.....IVHRD.....GDFGL.....KADVY.....
147807268	......RMLSK.....LVSMI.....ILVYD.....IIHRD.....SDFGL.....KSDVY.....
147805682	......ATISA.....LVKLY.....SLVYE.....IVHRD.....SDFGL.....KADVF.....
147811712	......QILTR.....LVSLY.....LLVYE.....VVHRD.....ADFGL.....KSDVY.....
147811981	......KAIGR.....LVQLL.....LLVYE.....IIHCD.....SDFGL.....KVDVY.....
147809868	......GMISA.....LVKLY.....LLIYE.....IVHRD.....SDFGL.....KADVY.....
147811984	......SAIVR.....LVQLL.....LLVYE.....TIHCD.....SDFGL.....KVDVY.....
147805741	......RYLSA.....LVTLL.....FLIYE.....LVHKD.....ADAGL.....KSDVY.....
147808029	......VSIGR.....IVSLL.....LLVYD.....VVHRD.....GDFGL.....RTDVY.....
147821256	......ATIGR.....VVRLV.....ALIYD.....ILHFD.....SDFGL.....KADVY.....
147821305	......EMLSR.....LVKLV.....LLCYE.....VIHRD.....ADFGL.....KSDVY.....
147816099	......ET---.....-----.....-LVYE.....IIHRD.....ADFGL.....RGDVY.....
147815409	......RVLSC.....VVLLI.....VLVYE.....IVHRD.....ADFGL.....KVDVY.....
147820255	......LMLSL.....LVNMI.....LLVYE.....VIYRD.....SDFGF.....KSDTY.....
147816692	......EMLSQ.....LVSLI.....ILIYE.....VIHRD.....ADFGL.....KSDVY.....
147821075	......ATISA.....LVKLY.....LLVYE.....IVHRD.....SDFGL.....KADVF.....
147822535	......FMLSL.....LVNMV.....ILVYE.....VIYRD.....SDFGL.....MSDVY.....
147818022	......EMLSK.....LVSMI.....ILVYE.....IIHRD.....ADFGL.....KSDVY.....
147815663	......KLLTR.....LVRLI.....VLIYE.....IVHRD.....ADFGM.....KSDVY.....
147818758	......DVLNK.....LVPLL.....LLVYK.....LVHRD.....GSLSE.....AYDVY.....
147828268	......EIISR.....LVSLV.....MLVYE.....IIHRD.....ADFGL.....KSDVF.....
147826815	......TLIAK.....LVRLL.....MLVYE.....IIHRD.....SDFGL.....KSDVY.....
147834674	......ESIAK.....LVKLF.....CQMQS.....IIHRD.....SDFGI.....KSDVF.....
147832573	......ATIGR.....VVGLV.....ALVYD.....ILHFD.....SDFGL.....KANIY.....
147828025	......GILSQ.....LVKLL.....LMIYN.....HTHRD.....ADFGL.....KSDVY.....
147834997	......ELLSR.....VVPLL.....LLVFE.....ILHRD.....TDLGM.....MSDVF.....
147832546	......EALST.....LVSLQ.....LLIYS.....IVHRD.....ADFGL.....RGDVY.....
147833902	......EILAR.....LLSLR.....LIVYD.....IIHRD.....ADFGF.....SCDVY.....
147833188	......LMLSL.....LVNLM.....ILVYE.....VIYRD.....SDFGL.....KSDVY.....
147833460	......DTLGK.....IVKLW.....LLVYE.....IVHRD.....ADFGV.....KSDLY.....
147826993	......IFLGQ.....LVKLI.....VLIYE.....VIFRD.....SDFGL.....MSDVY.....
147828214	......SMVSR.....VVELV.....VVAYE.....IIHRD.....ADFDL.....KSDVY.....
147833640	......HLLSQ.....LVSLE.....ILVYE.....IIHRD.....CDFGL.....KSDVY.....
147827610	......KIIGR.....LVRLL.....LLVYE.....IIHCD.....ADFGL.....KVDIY.....
147833187	......KVLTH.....LVRLI.....-IVYE.....VVHRD.....ADFGL.....KIDVY.....
147833187	......KVLTH.....LVRLI.....-LVYE.....VIHRD.....ADFGL.....KIDVF.....
147834523	......LVLSK.....LVSLI.....ILVYE.....IIHRD.....ADFGL.....KSDVY.....
147840288	......MVISK.....LVRLL.....LLIYE.....IIHRD.....XDFGM.....KSDVF.....
147838634	......GMISG.....LVRLY.....LLVYE.....IVHRD.....SDFGL.....KADVY.....
147842358	......FLMAK.....LQRLL.....MLIYE.....IIHCD.....SNFGM.....QSDVY.....
147840589	......DMISN.....LVRLI.....MLVYE.....IIHRD.....GDFGL.....KADVY.....
147835472	......FILSQ.....IVKLL.....LLVYE.....ILHRD.....SDFGL.....KSDVY.....
147840283	......VLISK.....LVRLL.....ILIYE.....IIHRD.....SDFGM.....KSDVF.....
147838007	......GMISC.....LVKLR.....LLVYE.....IVHRD.....SDFGL.....KADVY.....
147840284	......VVISK.....LVRLL.....MLIYE.....IIHRD.....SDFGM.....KSDVF.....
147839162	......EVLSC.....VVMLI.....LLVYE.....IVHRD.....GDFGL.....KADVY.....
147840285	......VVISK.....LVRLV.....MLIYE.....IIHRD.....SDFGM.....RSDVF.....
147843648	......EAPAS.....VL-LR.....IHAAR.....VIYRD.....SDFGL.....MSDVY.....
147843772	......ATIGR.....IVQLV.....AVIYD.....ILHFD.....SDFGL.....KADVY.....
147844250	......EILSK.....LVNLV.....LLVVE.....VIHRD.....GDFGL.....KTDVF.....
147844883	......GLISA.....LVKLY.....LLIYE.....IVHRD.....SDFGL.....KADVY.....
147844727	......ATIGR.....VVRLV.....ALIYD.....ILHFD.....SDFGL.....KADVY.....
147852023	......VILSQ.....VVKLL.....LLVYE.....SIHRD.....ADFGT.....KSDVY.....
147852668	......QILTR.....LVSLY.....LLVYE.....VVHRD.....ADFGL.....KSDVY.....

```
147853264   ......GLLSR......LVSLI......MLVYE......IIHRD......SDFGI......KSDVY......
147854008   ......KLLTR......LVRLI......ALVYE......IVHRD......ADFGM......KSDVY......
147854553   ......TIIHR......LVRLV......LLVYD......VVHRD......GDFGL......ESDVY......
147854730   ......SSLGR......LVSLR......MLVYD......VLHRD......GDFGL......QTDVF......
147856335   ......GMISA......LVKLY......LLIYE......IVHRD......SDFGL......KADVY......
147856526   ......NTIGS......LVRLC......LLVYE......IIHCD......SDFGL......KADVY......
147856630   ......TLIAK......LVRLL......MLVYE......IIHRD......SDFGL......KSDVY......
147856745   ......EVLSA......MARLL......ALVYE......IFHHD......AGFGL......RNDVY......
147857565   ......QVLLR......VACVR......SLVSK......VIYRD......SDFGL......RSDVY......
147857978   ......NFLGD......LVKLI......LLVYE......VIYRD......SDFGL......RSDVY......
147858097   ......EVLSK......LLLLL......CLVYE......IIHRD......GDVGL......KSDVY......
147858923   ......KLISN......LIRLL......LLVYE......HIHRD......ADFGL......KVDTY......
147859294   ......AGVGK......LVNLI......LLVAE......KIYHD......SSFGL......ESVIY......
147859761   ......TVLSK......VVMLL......LLVYE......IIHRD......GDFGL......RTDVY......
147860573   ......EVLSC......MVLLL......CLVYE......LVHRD......SDVGL......KSDIY......
147860501   ......ISMGR......LVQLH......ILVYD......VVHRD......GDFGL......SIDVY......
147860684   ......TVIGR......LVSLL......LLVYE......IIHCD......SDFGL......KVDVY......
147861240   ......ATIGR......VVKLV......ALIYD......ILHFD......SDFGL......KADVY......
147861521   ......KMITS......LVRLL......LLVYE......IVHRD......GDFGL......KADIY......
147862348   ......KAIGQ......LVQLL......LLVYE......IIHCD......SDFGL......KVDVY......
147862350   ......SAIGR......LVQLL......LLVYE......TIHCD......SDFGL......KVDVY......
147862349   ......KAIGR......LVQPL......LLVYE......IIQCD......SDFGL......KVDVY......
147863736   ......ASMGR......LVQMR......MLVYD......VIHRD......GDFGL......ASDVY......
147865107   ......ETIGK......LVPLL......FLIYE......IIHRD......SDFGL......KGDVY......
147866269   ......ETIGS......LVSLH......LLFYD......IIHRD......SDFGI......KSDVY......
147867243   ......FILSQ......IVKLL......LLVYE......ILHRD......SDFGL......RSDVY......
147867400   ......GMISG......LVRLY......LLVYE......IVHRD......SDFGL......KADVY......
148362061   ......KILQK......LVKLM......CFVYE......IVHRD......ANFSM......KIDVF......
148362063   ......KVLCK......LVELI......FLIYE......YVHQD......SDFGL......KSDVY......
148362069   ......KVLTH......LVRLI......-LVYE......VIHRD......ADFGL......KIDVY......
148362073   ......KILQK......LVKLM......CFVYE......IVHRD......ANFSM......KIDVF......
148672317   ......KVMAT......LVELL......CLVYA......HIHRD......SDFGL......KSDIY......
148697900   ......EQLSR......IVDFA......CLVYG......LIHGD......GDFGL......DTDTF......
148697902   ......EQLSR......IVDFA......CLVYG......LIHGD......GDFGL......DTDTF......
148697901   ......EQLSR......IVDFA......CLVYG......LIHGD......GDFGL......DTDTF......
149029884   ......EQLSR......IVDFA......CLVYG......LIHGD......GDFGL......DTDTF......
148923083   ......ETIGK......LVPLL......LLVYE......IIHRD......SDFGM......KGDVY......
149637309   ......EKLSR......IVDFA......CLVYV......LIHGD......GDFGL......EIDTY......
149714359   ......KVMAK......LVELL......CLVYV......HIHRD......SDFGL......KSDIY......
149758845   ......EQLSR......IVDFA......CLVYG......LIHGD......GDFGL......DTDTF......
149770633   ......KVLTH......LVRLI......-LVYE......VIHRD......ADFGL......KIDVY......
149770636   ......KVLTH......LVRLI......-LVYE......VIHRD......ADFGL......KIDVY......
157118817   ......QLLNK......ILRFM......HALTE......IFHRD......GDFGL......TSDVF......
157117746   ......AVLRS......VLRFI......HLVTE......IIHRD......ADFGL......KVDIF......
109003866   ......QLMNR......ILRFM......HALTE......IFHRD......ADFGL......KADVF......
109111096   ......QLMNR......ILRFM......HALTE......VFHRD......GDFGL......KADVF......
110757948   ......AVLRS......VLRFI......HLVTE......IIHRD......ADFGL......KVDIF......
110763901   ......QLMNK......ILRFM......HALTE......LFHRD......GDFGL......RSDVF......
111548682   ......KVMRC......VLKFI......NFIAE......IIHRD......ADFGL......KVDLF......
118405054   ......KVMRC......VLKFI......NFITE......IIHRD......ADFGL......SVDVF......
115437018   ......EMLAK......IVKLY......FLVCQ......IIHRD......SDFGI......KCDVY......
115457010   ......RTISE......LVKFF......FLVYE......ILHRD......ADFGL......SSDVY......
115457256   ......QTIGR......LVRML......LLVYE......IIHCD......SDFGL......KVDVY......
115457254   ......QTIGQ......LVRLL......LLVYE......IIHCD......SDFGL......KVDVY......
115456994   ......RTLGC......IVRFL......LLMYD......IVHRD......ADFGL......KSDVY......
115475041   ......EM---......-----......EDIEH......IIHRD......GNFCL......KTDVY......
114556237   ......QLMNR......ILRFM......HALTE......IFHRD......ADFGL......KADVF......
114556235   ......QLMNR......ILRFM......HALTE......IFHRD......ADFGL......KADVF......
114613983   ......KVMRC......VLKFI......NFITE......IIHRD......ADFRL......KVDVF......
114613985   ......KVMRC......VLKFI......NFITE......IIHRD......TDLGL......KVDVF......
114613987   ......KVMRC......VLKFI......NFITE......IIHRD......TDLGL......KVDVF......
114624598   ......QLMNR......ILRFM......HALTE......VFHRD......GDFGL......KADVF......
115528367   ......TLLQK......IVRYL......HPILE......IYHRD......TDFGL......KVDVF......
115628101   ......ELLNK......VVRYM......HPVLE......VCHRD......TDFGL......QVDVF......
115971269   ......ELLNK......VVRYM......HPVLE......VCHRD......TDFGL......QVDVF......
169855118   ......RLMKE......ICLFI......FIVSE......CIHRD......TDFGF......PTDVF......
117645004   ......KVMRS......VLKFI......NLLTE......IIHRD......ADFGL......TVDIF......
117616474   ......KVMRS......VLKFI......NLLTE......IIHRD......ADFGL......TVDVF......
118094558   ......QLMNR......ILRFM......HALTE......IFHRD......GDFGL......KADVF......
122889965   ......QLMNR......ILRFM......HALTE......IFHRD......ADFGL......KADVF......
124360648   ......QVLEA......DQYII......CAIKK......IVHRD......ADFGT......KSDVY......
```

160333917	......QLMNR......ILRFM......HALTE......IFHRD......GDFGL......KADVF......
125589398	......QTIGQ......LVRLL......LLVYE......IIHCD......SDFGL......KVDVY......
125538659	......QILGE......IVKLL......LLIYE......IVHRD......ADFGL......KIDVY......
125538661	......QILGE......IVKLL......LLVYE......IVHRD......ADFGL......KIDVY......
125547086	......STISE......LVRFY......FLVYE......ILHRD......ADFGL......SSDVY......
125547221	......QTIGQ......LVRLL......LLVYE......IIHCD......SDFGL......KVDVY......
125547076	......RTLGC......IVRFL......LLMYD......IVHRD......ADFGL......KSDVY......
125547084	......ITISE......LVKFY......FLVHE......ILHRD......ADFGL......NCDVY......
125547223	......QTIGQ......LVRLL......LLVYE......IIHCD......SDFGL......KVDVY......
125547109	......RTISE......LVKFF......FLVYE......ILHRD......ADFGL......SSDVY......
125572262	......KLIAK......LVRLL......ILLYE......IIHRD......SDFGI......RVCNG......
125581346	......QILGE......IVKLL......LLIYE......IVHRD......ADFGL......KIDVY......
125589229	......QPSSE......LLKFY......FLVYE......ILHRD......ADFGL......YSDVF......
125821362	......QLMNR......ILRFV......HALTE......IFHRD......GDFGL......KVDVF......
125848834	......KVMRC......VLKFI......NLISE......VIHRD......ADFGL......RVDIF......
126302848	......SLLQK......IVRYL......YPILE......IYHRD......TDFGL......KVDVF......
126305796	......QLMNR......ILRFM......HALTE......IFHRD......ADFGL......KADVF......
126314544	......KVMRC......VLKFI......NFITE......IIHRD......ADFGL......KVDVF......
126324828	......KVMRS......VLKFI......NLLTE......IIHRD......ADFGL......TVDVF......
126334772	......QLMNR......ILRFM......HALTE......VFHRD......GDFGL......KADVF......
134032232	......QILNK......VIRLS......YLVYE......SIHKD......ANLSL......KLDVY......
147901229	......SLLQK......IVRYL......YPILE......IYHRD......TDFGL......KVDVF......
147843348	......LTLGK......IVKLY......LLLYE......IIHRD......GDFGL......KCDIY......
147859823	......RVLSK......IVKLY......FLVYE......ILHRD......SDFGT......KCDVY......
148362072	......EILNK......VIRLS......YLVYE......SIHKD......TNLSL......KLDVY......
148708465	......KVMRS......VLKFI......NLLTE......IIHRD......ADFGL......TVDVF......
148708464	......KVMRS......VLKFI......NLLTE......IIHRD......ADFGL......TVDVF......
158186665	......KVMRC......VLKFI......NFITE......IIHRD......ADFGL......KVDVF......
149047496	......KVMRS......VLKFI......NLLTE......IIHRD......ADFGL......TVDVF......
149047499	......KVMRS......VLKFI......NLLTE......IIHRD......ADFGL......TVDVF......
149047500	......KVMRS......VLKFI......NLLTE......IIHRD......ADFGL......TVDVF......
149047503	......KVMRS......VLKFI......NLLTE......IIHRD......ADFGL......TVDVF......
149632373	......QLMNR......ILRFM......HALTE......IFHRD......GDFGL......KVRFG......
149641032	......KVMRA......VLKFI......NLLTE......IIHRD......ADFGL......TVDVF......
149693698	......QLMNR......ILRFM......HALTE......IFHRD......ADFGL......KADVF......
149720259	......KVMRS......VLKFI......NLLTE......IIHRD......ADFGL......TVDVF......
149741224	......QLMNR......ILRFM......HALTE......VFHRD......GDFGL......KADVF......
149756077	......KVMRC......VLKFI......NFITE......IIHRD......ADFGL......KVDVF......
108707264	......VSMGR......LVQLL......LLVYE......VVHRD......GDFGL......QVDW-......
108707264	......VSLGR......VVQLL......LLVYD......VIHRD......GDFGL......YTDVF......
115451705	......QTLGR......IVRLL......LLVYE......ILHRD......ADFGL......KSDVY......
108708887	......RIIGK......LVRIW......MLVTE......VIHCD......ADFGL......KVDVY......
108862138	......SVIGR......LVRMW......ILVSE......IVHCD......TDFGL......KVDVY......
109082544	......SMLHA......IVALI......CFALE......IIFCD......SDYGI......KVDMF......
109481160	......VVLCH......LISLL......MLVME......IIYRD......ADYGI......QADVY......
109482771	......VVLCH......LISLL......MLVME......IIYRD......ADYGI......QADVY......
109658494	......VVLCH......LISLL......MLVME......IIYRD......ADYGI......QADVY......
161078441	......AVLLT......IVPLV......ALVLE......IIYRD......ADYGI......KVDCF......
110738055	......MIASS......IVPLL......FLVYK......VVHRD......CDFGL......KTDVY......
145350358	......VILSQ......VVKLL......MLVYE......IYHRD......ADFGT......KSDVY......
110738585	......VILSQ......VVKLL......ILVYE......IYHRD......ADFGT......KSDVY......
115460768	......VLIAR......LVKLV......LLIYE......IIHRD......SDFGM......KSDIY......
115439089	......SVIGK......LVRIW......LLVYE......VFHCD......ADFGL......KVDVY......
115440357	......MLIAK......LVRLL......ILIYE......IIHRD......SDFGM......KSDVF......
115441307	......STIGN......LIRLL......LLVYE......IIHCD......ADFGL......KADVF......
115439127	......DLLSR......FINLL......MMVLE......ITHPD......ADMSV......PVNVC......
115440745	......NTLAS......LCKLI......MLVYE......AMYND......SGYGI......KSNVW......
115449021	......ETVGS......LVSLQ......LLFYD......IIHRD......ADFGI......KSDVY......
115449399	......FILSQ......VVKLL......MLVYE......ILHGD......ADFGA......KSDVY......
115453579	......RIIGK......LVRIW......MLVTE......VIHCD......ADFGL......KVDVY......
115455635	......QTLGR......IVRLL......LLVYE......ILHRD......ADFGL......KSDVY......
115457908	......VILSQ......IVKLF......LLVYD......VLHRD......SDFGA......KSDVY......
115460292	......IVLSQ......VVKIL......LLVYE......IIHRD......SDFGA......KSDVY......
115457250	......ETIGQ......LVRLL......LLVYE......IIHCD......SDFGL......KVDVY......
115460782	......VLIDK......LVRLL......LLIFE......VIHRD......SDFGM......KSDTY......
115469634	......Q-LVR......LIRLL......ILVYE......IVHRD......GDFGV......KTDVF......
115467594	......ALLSR......LVNLV......ILIYE......VIHRD......ADFGL......KSDVY......
115466614	......SIIGR......LVRIW......LLVSE......IVHCD......ADFGL......KADVY......
115468174	......TIIGS......LVKLR......LLAYE......IVHCD......SDFGL......KSDVY......
115472595	......ILVAK......LVRLI......ILVYE......IVHRD......SDFGL......KSDVF......
115472589	......VLVAK......LVRLI......ILVYE......IVHRD......SDFGL......KLDVF......
115470375	......KALRS......LLPVL......ALVYK......IIHCD......GDFGI......SGDVY......

```
115471653    ......DIICK.....LVHLL.....LLVYE.....IIHRD.....SDFGI.....MQDVY......
115481112    ......RVHSQ.....VVRLI.....MMVME.....VLHGD.....TDFGI.....KSDVY......
115480896    ......SIHSQ.....VVELI.....MIVTK.....ICHGD.....SDFGV.....RSDVY......
115486327    ......EALKN.....LIRVI.....ALILE.....MVHCD.....SDFGL.....EGDVY......
114645336    ......VVLCH.....LISLL.....MLVME.....IIYRD.....ADYGI.....QADVY......
114659198    ......SMLHA.....IVALI.....CFALE.....IIFCD.....SDYGI.....KVPHF......
115373123    ......ELCST.....IVQVF.....FLAME.....LVHRD.....SDFGI.....RMDLF......
115844303    ......TVLRH.....LVSME.....ILVME.....IVYRD.....SDYGI.....EVDIY......
116059111    ......WYLTT.....VTRVY.....HMVME.....HVHRD.....TDFGL.....SSDMY......
90991702f    ......SMLHA.....IVALI.....CFALE.....IIFCD.....SDYGI.....KVDMF......
116256118    ......VLLSQ.....IVKLL.....ILVYE.....IFHRD.....SDFGT.....KSDVY......
116308956    ......ETIGS.....LVRLI.....LLVYE.....IAHLD.....AYFGL.....KVDIY......
116309301    ......LILSR.....IVKLL.....ILVYE.....ILHGD.....SDFGC.....KSDVY......
116309219    ......VILSQ.....IVKLF.....LLVYD.....VLHRD.....SDFGA.....KSDVY......
116309658    ......ATAGR.....LVRLY.....ALVYE.....IIHYD.....ADFGL.....KCDVY......
116309224    ......AILSQ.....IVKLF.....LLVYD.....VLHRD.....ADFGA.....KSDVY......
116309298    ......VILCR.....IVKLL.....MLVYE.....ILHGD.....SDFGC.....KNDVY......
116311017    ......LILSR.....IVKLL.....VLVYE.....IFHGD.....SDFGC.....KSDVY......
116310263    ......RSIGI.....LVRLI.....LLVYE.....IIHCD.....ADFGM.....KVDVY......
116643248    ......TLLEK.....VIQFV.....MIVVE.....IIHCD.....SGFGM.....RVDAH......
117621383    ......VSLAA.....LVGVE.....ALVMA.....ILHGD.....GDFG-.....RLEVR......
118469133    ......VAMGK.....IVNIV.....-IVMQ.....TLHRD.....TDFGI.....ASDVY......
121703884    ......LSLCN.....IVSLL.....PVLFV.....IVHGD.....SDFGE.....KFDAY......
94717653     ......SMLHA.....IVALI.....CFALE.....IIFCD.....SDYGI.....KVDMF......
119578219    ......VVLCH.....LISLL.....MLVME.....IIYRD.....ADYGI.....QADVY......
119578218    ......VVLCH.....LISLL.....MLVME.....IIYRD.....ADYGI.....QADVY......
119892297    ......VVLCH.....LISLL.....MLVME.....IIYRD.....ADYGI.....QADVY......
119622693    ......SMLHA.....IVALI.....CFALE.....IIFCD.....SDYGI.....KVDMF......
119622694    ......SMLHA.....IVALI.....CFALE.....IIFCD.....SDYGI.....KVDMF......
119622692    ......SMLHA.....IVALI.....CFALE.....IIFCD.....SDYGI.....KVDMF......
119913520    ......SMLHA.....IVSLI.....CFALE.....IIFCD.....SDYGI.....KVDMF......
123366883    ......LLMRA.....LNPPI.....IIASN.....IVHGD.....SDFGI.....ASDVY......
123437585    ......SVMAK.....VLGLR.....CILMD.....GIHRD.....ADFGL.....KVDVY......
123976989    ......WTMAT.....ILRLI.....CIITE.....IIHRD.....CDFGL.....KVDVY......
123452995    ......LLMRA.....LNPPI.....IIATN.....IIHGD.....SDFGI.....ASDVY......
123472416    ......WTMAT.....ILRLV.....CIVTE.....IIHRD.....CDFGL.....KVDVY......
154412199    ......EILVR.....VIPFV.....AVATR.....IIHRD.....CDFGV.....SADIF......
123482570    ......ETLAK.....LVPFI.....TIVTE.....IIHRD.....CDFGI.....KVDVY......
154419335    ......VTMAK.....ILPFV.....SIITE.....IVHRD.....CDFGI.....KVDIY......
154413209    ......HTMAL.....CMPML.....CLVLE.....VYHRD.....CDFGL.....KVDVY......
124359368    ......STIGR.....LVKLY.....ALVYE.....IIHYD.....ADFGL.....KCDVY......
124359371    ......ITIGR.....LVKLY.....ALVYE.....IIHYD.....ADFGL.....KCDVY......
125571548    ......DLLSR.....FINLL.....MMVLE.....ITHPD.....ADMSV.....PVNVC......
125571524    ......SVIGK.....LVRIW.....LLVYE.....VFHCD.....ADFGL.....KVDVY......
125589395    ......ETIGQ.....LVRLL.....LLVYE.....IIHCD.....SDFGL.....KVDVY......
125591754    ......VLIAK.....LVRLL.....LLIYE.....IIHRD.....SDFGM.....KSDTY......
125527231    ......DLLSR.....FINLL.....MMVLE.....ITHPD.....ADMSV.....PVNVC......
125526228    ......EALRN.....LIKII.....ALVFE.....VIHCD.....ADFGL.....VGDAY......
125528685    ......TVLEK.....VVQFV.....MIISE.....IIHCD.....AGFGL.....SVDAF......
125533274    ......SVIGR.....LVRMW.....ILVSE.....IVHCD.....TDFGL.....KVDVY......
125540272    ......EILRS.....LLPIL.....ALIYE.....IIHCD.....GDFGI.....YGDVY......
125534812    ......EALKN.....LVKVI.....AIVFD.....VVHCD.....GDFGL.....NGDIY......
125535056    ......EALKS.....LVRVI.....ALILE.....LVHCD.....SDFGL.....EGDVY......
125531120    ......RVHSQ.....VVRLI.....MMVME.....VLHGD.....TDFGI.....KSDVY......
125533544    ......EVLGT.....LARLL.....LLVYE.....ILHRD.....ADFGL.....KADVY......
125535033    ......EAFRN.....LIRVI.....ALILE.....LVHCD.....SDFGL.....EGDIY......
125535026    ......VALRN.....LIRVI.....ALVLE.....LVHCD.....SDFGL.....EGDIY......
125537555    ......EILGS.....LVNLR.....LLIYD.....IVHRD.....SDFGL.....KSDVY......
125530937    ......SIHSQ.....VVELI.....MIVTK.....ICHGD.....SDFGV.....RSDVY......
125533580    ......NALKN.....LVTIL.....ALVYE.....IVHSD.....GDFGL.....ASDVY......
125531434    ......EALRD.....LVKII.....AIVLD.....VVHCD.....GDFGL.....NGDIY......
125536740    ......QTLRS.....LVSVL.....AIVYE.....VVHCD.....GDFGL.....AGDVY......
125534814    ......EALRD.....LVKII.....AIVLD.....VVHCD.....GDFGL.....NGDIY......
125535678    ......SVIGR.....LVRMW.....ILVYE.....IIHCD.....TDFGL.....KVDVY......
125533567    ......NALRN.....LVPIF.....ALVYE.....IIHCD.....GDFGL.....ASDVY......
125538350    ......DLLGS.....IVSLL.....FIVYE.....VIHRD.....SDFGL.....KSDVY......
125534706    ......VLVGK.....LVRVL.....LLVYE.....IIHRD.....SDFGL.....KSDVF......
125546293    ......RVIAR.....LLRLL.....LLVYE.....IIHCD.....ADFGL.....KADVY......
125549866    ......VLIAR.....LVKLV.....LLIYE.....IIHRD.....SDFGM.....KSDIY......
125547414    ......STIGM.....LVKLI.....MLVYE.....IIHCD.....ADFGL.....KVDVY......
125547546    ......LILSR.....IVKLL.....VLVYE.....IFHGD.....SDFGC.....KSDVY......
125544230    ......AIISK.....VLALY.....ATVTE.....IVHFD.....ADFGL.....KIDVY......
125543172    ......VSLGR.....VVQLL.....LLVYD.....VIHRD.....GDFGL.....YTDVF......
125542967    ......SMVSR.....FIRLM.....ILVYQ.....IVHRD.....ADFNL.....KSDVY......
```

125547864	......EALKS.....LIPLI.....ALIYA.....IIHCD.....GDFGI.....YGDVY......
125542989	......QTLGR.....IVRLL.....LLVYE.....ILHRD.....ADFGL.....KSDVY......
125549875	......VLIAK.....LVRLL.....LLIYE.....IIHRD.....SDFGM.....KSDTY......
125545537	......SVVSR.....FTQLL.....IVLYE.....IVHRD.....GDFNL.....KSDVY......
125545869	......QTLGR.....IVRLL.....LLVYE.....ILHRD.....ADFGL.....KSDVY......
125550037	......SVIGR.....LVRIW.....LLVSE.....IVHCD.....TDFGL.....KADVY......
125548268	......SSIGV.....LVKLI.....LLVYE.....IIHCD.....ADFGM.....KVDVY......
125541342	......ETVGS.....LVSLQ.....LLFYD.....IIHRD.....ADFGI.....KSDVY......
125547934	......LILSR.....IVKLL.....ILVYE.....ILHGD.....SDFGC.....KSDVY......
125549869	......VLIAK.....LVKLL.....LLIYE.....IIHRD.....SDFGM.....KSDTY......
125549867	......VLIDK.....LVRLL.....LLIFE.....VIHRD.....SDFGM.....KSDTY......
125552679	......EVLSK.....LMNLV.....MMVFE.....VLLRD.....SDISF.....HSTVY......
125560657	......RAIAR.....LVRLL.....RLVYE.....IIHCD.....ADFGL.....KVDIY......
125551818	......DLLGR.....IVSLL.....YIVYE.....VIHRD.....ADFGL.....KSDVY......
125558425	......KILGR.....LVKLL.....MLVYE.....VVHRD.....GDFGL.....KSDVY......
125553293	......ESLSR.....FINLL.....TMVFE.....NVHPD.....ADLSV.....--NVY......
125558655	......LLLAE.....LVKLQ.....LLVYE.....IIHRD.....ADFGL.....KIDVL......
125554466	......ETVGS.....LVSLQ.....LLFYD.....IIHRD.....TDFGI.....KSDVY......
125558676	......VLVAK.....LVRLI.....ILVYE.....IVHRD.....SDFGL.....KLDVF......
125558683	......VLVAK.....LVRLV.....LLVYE.....IVHRD.....GDFGL.....KSDVF......
125557071	......KALRS.....LLPVL.....ALVYK.....IIHCD.....GDFGI.....SGDVY......
125558651	......VLVAK.....LVRLV.....LLV--.....IIHRD.....SDFGL.....KSDVF......
125556666	......SYLGQ.....LVELI.....LLVYE.....IIYRD.....SDFGL.....MSDVY......
125560089	......LLLGR.....LVNLV.....MLLYA.....VVHRD.....ADFGL.....KSDVY......
125558672	......VLVAK.....LVRLV.....MLVYE.....IVHRD.....SDFGL.....KSDVF......
125555407	......DVIPK.....IIELI.....----E.....IVHRD.....SDFGL.....KTDVY......
125557146	......QTLGR.....IVRLL.....LLVYE.....ILHRD.....ADFGL.....KSDVY......
125554931	......ALLSR.....LVNLV.....ILIYE.....VIHRD.....ADFGL.....KSDVY......
125552352	......STIRT.....LVRLL.....LLVCE.....IIHCD.....ADFGL.....KADVF......
125558680	......ILVAK.....LVRLI.....ILVYE.....IVHRD.....SDFGL.....KSDVF......
125555820	......SVISR.....LVRVW.....ILVSE.....VIHCD.....ADFGL.....KVDVY......
125569757	......EMLSR.....LVKLI.....-----.....VIHRD.....SDFGL.....KSDVY......
125561607	......AAIGA.....LPRLL.....LLVYE.....PIHGD.....TDYGL.....SADVY......
125563199	......EALKN.....LIRVI.....ALILE.....MVHCD.....SDFGL.....EGDVY......
125561357	......ATLGR.....IVKLW.....LLVYE.....IVHRD.....ADFGV.....KSDVY......
125569458	......DTLSR.....FINLL.....MMVFE.....VAITD.....ADVGI.....-SNVY......
125570640	......EALRN.....LIKII.....ALVFE.....VIHCD.....ADFGL.....VGDAY......
125570383	......ELIAK.....LVRLL.....ILVYE.....VIHRD.....SDFGL.....KSDVF......
125562786	......EWLAK.....LVRLL.....IIITE.....IIHRD.....SDFGF.....KSDVF......
125573857	......-----.....-VKLY.....FLVYD.....IIHRD.....SDFGT.....KCDVY......
125578401	......SVIGR.....LVRMW.....ILVSE.....IVHCD.....TDFGL.....KVDVY......
125580206	......EILGS.....LVNLR.....LLIYD.....IVHRD.....SDFGL.....KSDVY......
125574162	......ILQSE.....IIQLF.....MLVYE.....IRHGD.....SDFGT.....KSDIY......
125572943	......TVLEK.....VVQFV.....MIISE.....IIHCD.....AGFGL.....SVDAF......
125572924	......STIGK.....LIRLL.....LLVYE.....IIHCD.....ADFGL.....KADVF......
125576693	......RTLGS.....IVRLL.....VLVYE.....IVHRD.....ADFGL.....KSDVY......
125572796	......STIGN.....LIRLL.....LLVYE.....IIHCD.....ADFGL.....KADVF......
125573822	......SIHSQ.....VVELI.....MIVTK.....ICHGD.....SDFGV.....RSDVY......
125577668	......QLIAK.....LVKLL.....ILIYE.....VIHRD.....SDFGL.....KSDVF......
125572794	......STIGN.....LIRLL.....LLVYE.....IIHCD.....ADFGL.....KADVF......
125572925	......STIGN.....LIRLL.....LLVYE.....IIHCD.....ADFGM.....KADVF......
125590061	......VILCR.....IVKLL.....MLVYE.....ILHGD.....SDFGC.....KNDVY......
125584088	......FILSQ.....IIKLL.....MLIYE.....ILHGD.....SDFGA.....KSDVY......
125581052	......DLLGS.....IVSLL.....FIVYE.....VIHRD.....SDFGL.....KSDVY......
125583877	......ETVGS.....LVSLQ.....LLFYD.....IIHRD.....ADFGI.....KSDVY......
125588076	......QTLGR.....IVRLL.....LLVYE.....ILHRD.....ADFGL.....KSDVY......
125584086	......FILSQ.....VVKLL.....MLVYE.....ILHGD.....ADFGA.....KSDVY......
125586577	......AIISK.....VLALY.....ATVTE.....IVHFD.....ADFGL.....KIDVY......
125590037	......AILSQ.....IVKLF.....LLVYD.....VLHRD.....ADFGA.....KSDVY......
125584105	......FILSQ.....VVKLL.....MLVYE.....ILHGD.....SDFGA.....KSDVY......
125582883	......EVLRS.....LLPVL.....ALIYE.....IVHCD.....GDFGI.....YGDVY......
125597656	......SVISR.....LVRVW.....ILVSE.....VIHCD.....ADFGL.....KVDVY......
125600583	......VLVAK.....LVRLI.....ILVYE.....IVHRD.....SDFGL.....KLDVF......
125590932	......RSIGI.....LVRLI.....LLVYE.....IIHCD.....ADFGM.....KVDVY......
125593746	......DLLGR.....IVSLL.....YIVYE.....VIHRD.....ADFGL.....KSDVY......
125600568	......LLLAE.....LVRLQ.....LLVYE.....IIHRD.....ADFGL.....KIDIF......
125600590	......VLVAK.....LVQLV.....MLVYE.....IVHRD.....GDFGL.....KSDVF......
125597303	......TIIGS.....LVKLR.....LLAYE.....IVHCD.....SDFGL.....KSDVY......
125596860	......ALLSR.....LVNLV.....ILIYE.....VIHRD.....ADFGL.....KSDVY......
125598958	......KALRS.....LLPVL.....ALVYK.....IIHCD.....GDFGI.....SGDVY......
125600591	......VLVAK.....LVRLV.....LLVYK.....IIHRD.....GDFGL.....KSDVF......
125591748	......VLIAR.....LVKLV.....LLIYE.....IIHRD.....SDFGM.....KSDIY......
125598335	......Q-LVR.....LIRLL.....ILVYE.....IVHRD.....ADFGV.....KTDVF......

```
125596161    ......SIIGR.....LVRIW.....LLVSE.....IVHCD.....ADFGL.....KADVY.....
125600595    ......VLVTK.....LVRLV.....LLVYE.....IVHRD.....GDFGL.....KSDVF.....
125600577    ......VLVAK.....LVRLV.....MLVYE.....IVHRD.....SDFGL.....KSDVF.....
125594576    ......EVLSK.....LMNLV.....MMVFE.....VLLRD.....SDISF.....HITVY.....
125603917    ......AILSQ.....VVKLF.....-----.....IIHRD.....SDFGA.....KSDVY.....
125604761    ......ELLAK.....LVRLL.....IIITE.....IIHRD.....SDFGF.....KSDVF.....
125601631    ......EALKS.....LVRVI.....ALILE.....LVHCD.....SDFGL.....EGDVY.....
125855485    ......AVLGR.....LVGLL.....ILVME.....IIYRD.....TDYGI.....QADVY.....
125599019    ......RVLRM.....LMRIL.....ALLLE.....VLHCD.....ADFGI.....KSDVF.....
126644801    ......RIMNL.....IVQYM.....FLICE.....IIHRD.....GDFGL.....HIDVY.....
126277216    ......SMLHS.....IVSLI.....CFALE.....IIFCD.....SDYGI.....KVDMF.....
126340191    ......VVLCH.....LISLL.....MLVME.....IIYRD.....ADYGI.....QADVY.....
163644492    ......SLLSR.....VIGLV.....SLVVE.....IIYRD.....GDYGI.....KVDCF.....
145298855    ......ASLAA.....LIRVL.....VMALN.....IMHGD.....GDFGA.....RIEVR.....
145349744    ......WYLNA.....ITRVY.....HMVME.....HVHRD.....TDFGL.....SSDIY.....
145337237    ......ASMSQ.....IVSLL.....AIIYE.....IVHFD.....SDFGL.....KSDVY.....
145340395    ......DTLSR.....FVNLI.....MMVFE.....MAHTD.....SEFNL.....EANVH.....
145361901    ......EILSK.....IISLL.....FIVYE.....IIHRD.....SDFGL.....KSDVY.....
146231954    ......VVLCH.....LISLL.....MLVME.....IIYRD.....ADYGI.....QADVY.....
147770228    ......QTLGN.....IVRLI.....LLVYE.....IVHRD.....ADFGL.....KSDVY.....
147771638    ......QILSE.....LVRMI.....AIVLE.....VVHCD.....ADFGI.....RGDVY.....
147770087    ......EVLCK.....IVNLL.....LLVYE.....IIHRD.....ADFGL.....KSDVY.....
147768482    ......VLIAK.....LVRLL.....CML--.....IIHRD.....SDFGL.....KSDVF.....
147772402    ......RALTE.....IVKLL.....FLVYE.....IVHRD.....SDFGT.....KTDVF.....
147777441    ......HALTQ.....IVKLY.....FLVYE.....IVHRD.....SDFGT.....KTDVY.....
147782461    ......ATLAR.....IVRLL.....LLFYD.....IIHRD.....ADFGL.....KSDVY.....
147788157    ......KTIGS.....LVRLI.....LLVFE.....IVHLD.....SDFGL.....KVDIY.....
147790158    ......ASLGK.....LVHLQ.....LLVYD.....VVHRD.....GDFGL.....SSDVF.....
147787796    ......TLTAT.....LVKLL.....MLIYE.....IIHRD.....ADFGI.....KSDAY.....
147788158    ......ETIGS.....LVRLI.....LLVYE.....IVHLD.....SDFGL.....KVDIY.....
147802196    ......TLLEK.....VVQFV.....MIVSE.....VIHCD.....AGFGL.....SVDSF.....
147802220    ......KTLGR.....LVTLI.....FLIYN.....IVHRD.....SDFGL.....KADVY.....
147815186    ......ATISS.....LVRLI.....LLVYE.....IVHCD.....SDFGL.....KSDVY.....
147821362    ......IYIAK.....LVRLL.....SM---.....IIHRD.....SDFGM.....KVGTY.....
147816247    ......VILSQ.....IVKLL.....LLVYE.....IFHRD.....ADFGT.....KSDVY.....
147821313    ......DVLGN.....IVRLL.....MLLYE.....IVHRD.....ADFGV.....FVDW-.....
147826449    ......EILGS.....LVNLR.....LLIYD.....IVHRD.....SDFGL.....KSDVY.....
147853795    ......EVMRN.....LAKII.....ALVLE.....VVHCD.....SDFGI.....KCDTY.....
147855067    ......EVMRN.....LAKII.....ALVLE.....VVHCD.....SDFGI.....KGDIY.....
147856314    ......AVLSK.....--EMR.....CL---.....FIHRD.....SDFGL.....SQYLA.....
147856468    ......EILGT.....IVKLL.....CISNE.....IIHRD.....ADFGL.....KIDVY.....
147856780    ......DWLSK.....IVSLL.....FLVYE.....VIHRD.....SDFGL.....KSDVY.....
147857053    ......STIGT.....LIRLR.....FLVYE.....IIHCD.....SDFGL.....KADVF.....
147857736    ......KTLSR.....LVKVL.....ALVLE.....IVHCD.....SDFGT.....KVDVF.....
148362058    ......KVLTH.....LVRLI.....FLVYE.....VIHRD.....ADFGL.....KVDVY.....
148672362    ......VVLCH.....LISLL.....MLVME.....IIYRD.....ADYGI.....QADVY.....
149017601    ......VVLCH.....LISLL.....MLVME.....IIYRD.....ADYGI.....QADVY.....
149596247    ......SMLHS.....IVSLI.....CFALE.....IIFCD.....SDYGI.....KVDMF.....
149691041    ......SMLHA.....IVSLI.....CFALE.....IIFCD.....SDYGI.....KVDMF.....
115456177    ......NMMCK.....LVKFI.....VIVSE.....IIHRD.....TDFGL.....KVDVY.....
108707639    ......LQLMS.....ILQFH.....CIITR.....ITYRD.....GDMGI.....SSNVY.....
157134213    ......KLFWS.....IVELK.....CLVME.....VIHRD.....TDFGL.....ASDVW.....
157127548    ......--LRK.....IVKFK.....CIIME.....IIHRD.....SDFGT.....KVDIW.....
157114995    ......CQLSR.....IIGLY.....CLVME.....MIHRD.....CDFGT.....KCDVF.....
109008598    ......SILCQ.....VIQFV.....FIVTQ.....IIHRD.....ADFGE.....KADVF.....
109019961    ......RLFAM.....IIELR.....CLVLE.....ILHRD.....TDFGL.....GSDIW.....
109019963    ......RLFAM.....IIELR.....CLVLE.....ILHRD.....TDFGL.....GSDIW.....
109072089    ......RQLSR.....IVKLY.....CLVME.....LIHRD.....CDFGT.....KCDVF.....
109072081    ......RQLSR.....IVKLY.....CLVME.....LIHRD.....CDFGT.....KCDVF.....
109084171    ......KLFTM.....IIALR.....CLVME.....IIHRD.....TDFGL.....GSDVW.....
109084169    ......KLFTM.....IIALR.....CLVME.....IIHRD.....TDFGL.....GSDVW.....
109096938    ......--LRK.....IITFK.....CILME.....IIHRD.....SDFGT.....KVDIW.....
109100121    ......EILSV.....IIQFY.....GIVTE.....VIHRD.....CDFGA.....TCDTY.....
109105483    ......RLFAM.....IIALK.....CLVME.....VIHRD.....TDFGL.....GSDVW.....
109124738    ......RLFGA.....IIALR.....CLVME.....IIHRD.....CEASA.....RISNW.....
109124736    ......RLFGA.....IIALR.....CLVME.....IIHRD.....SSTDI.....QGALC.....
109458437    ......RLFGA.....IIALR.....CLVME.....IIHRD.....TDFGL.....SSDVW.....
109463388    ......RLFAM.....IIALK.....CLVME.....VIHRD.....TDFGL.....GSDVW.....
109478448    ......KLFAM.....IIALR.....CLVME.....VIHRD.....TDFGL.....GSDVW.....
109494652    ......--LRK.....IIAFK.....CIIME.....IIHRD.....SDFGT.....KVDIW.....
109508285    ......RLFAM.....IIQLR.....CLVLE.....ILHRD.....TDFGL.....GSDIW.....
109727320    ......MMLAN.....IVRFI.....CIVTE.....LIHRD.....ADFGV.....KVDVY.....
110180236    ......SILSR.....VILLL.....SLVTE.....IVHRD.....CDFGL.....KSDIF.....
```

```
110180232   ......LLLSS......VVSFY......ATVAE......IVHFD......GDLGL......KIDVY......
110180230   ......EILSK......VVAFY......ATVTE......TVHFD......GDFGL......KVDVF......
110739152   ......EILSK......VVAFY......TLVTE......IVHFD......GDFGL......KVDVF......
110760864   ......KLFWL......IVQLE......CLVME......LIHRD......TDFGL......ASDVW......
110766558   ......RHLRK......IVQFK......CIIME......IIHRD......SDFGT......KVDIW......
110772451   ......RQLSR......IVKLY......CLVME......LIHRD......CDFGT......KCDVF......
11994655    ......QILSN......VVAFY......ATVTE......IVHFD......GDLGL......KVDVF......
115436556   ......LILSQ......IVKLL......MLVYE......ILHGD......SDFGA......KSDVY......
115439117   ......YILSR......VILFL......SMVTE......IVHRD......CDFGL......KCDIF......
115446655   ......DALMS......IVKLF......FLVYE......IVHRD......SDFGI......KCDVY......
115446661   ......DALTR......IVKLF......FLVYE......IVHRD......SDFGI......KCDVY......
115445179   ......KALKN......LVKVI......GLVFE......VVHCD......CDFGL......EGDVY......
115447387   ......LILSQ......IVKLY......MLVYK......IIHGD......SDFGA......KSDVY......
115454263   ......AVWHK......VTKFV......CVVVE......IVHRD......ADFGV......KCDVY......
115452449   ......LQLMS......ILQFH......CIITR......ITYRD......GDMGI......SSNVY......
115459444   ......LILSP......IIKLL......MLVYE......ILHGD......TDFGA......KSDVY......
115464923   ......TILAR......VIKLI......CVITE......VIHRD......VDFGV......KVDVY......
115466756   ......DKLAS......VVAFY......ATVTE......IVHFD......GDLGL......KVDVF......
115472181   ......IIQSQ......IVRLL......MLVTE......ILHGD......CDFGI......KCDVY......
115479695   ......VILSQ......IVKLL......ILVYE......ILHGD......SDFGA......KSDVY......
115479693   ......LILSQ......VVKLL......MLVYE......ILHGD......SDFGA......KSDVY......
115480683   ......AILSQ......VVKLF......LLVYE......IIHRD......SDFGA......KSDVY......
115479699   ......LILSQ......IVKLL......ILVYE......ILHGD......SDFGA......KSDVY......
115487494   ......YILRE......VVRFI......CIITE......IIHRD......ADFGV......KADVF......
115489568   ......IILSQ......IVRLL......MLVYE......ILHGD......ADFGA......RSDVY......
115489382   ......QILSK......VVAFY......ATVTE......IVHFD......GDFGL......KVDVF......
106733448   ......RQLSR......IVKLY......CLVME......LIHRD......CDFGT......KCDIF......
159896768   ......QILAR......LPRVY......YLIMD......VIHRD......LDFGL......RSDVY......
159896769   ......QILAR......LPRVY......FLIMD......VIHRD......VDFGL......RSDVY......
114205587   ......KLFAM......IIALR......CLVME......IIHRD......TDFGL......GSDVW......
114557316   ......SILCQ......VIQFV......FIVTQ......IIHRD......ADFGE......KADVF......
114590841   ......--LRK......IIAFK......CIIME......IIHRD......SDFGT......KVDIW......
114608523   ......RQLSR......IVKLY......CLVME......LIHRD......CDFGT......KCDVF......
114638588   ......RLFAM......IIALK......CLVME......VIHRD......TDFGL......GSDVW......
114644516   ......--LRK......IITFK......CILME......IIHRD......SDFGT......KVDIW......
114653741   ......KLFAM......IIALR......CLVME......IIHRD......TDFGL......GSDVW......
114653743   ......KLFAM......IIALR......CLVME......IIHRD......TDFGL......GSDVW......
115374659   ......RLAAQ......IVQIF......YIAME......IVHRD......LDFGI......RSDIF......
116050447   ......ACLAA......LIEVL......GLVLE......ISHGD......GDFGA......RLEVR......
115631812   ......SILCR......VIRFV......AIVTQ......IIHRD......ADFGE......KADIF......
115655516   ......MQLSR......IVKLY......CLVME......LIHRD......CDFGT......KCDIF......
115706416   ......SILCR......VIRFV......AIVTQ......IIHRD......ADFGE......KADIF......
115707213   ......AVHKR......IVQLV......MIVMQ......ILHLD......CDLGL......ASDVW......
115724402   ......AIHKR......IVQLV......MIVMQ......ILHLD......CDLGL......ASDVW......
115920158   ......MIQQL......FPKFW......CQAVE......LLHND......IDYGM......ASDVF......
115926271   ......KLFSL......IISLR......CIVME......LIHRD......TDFGL......SSDVW......
116055320   ......KIMQQ......IVQFL......TIVSE......VIHRD......GDFGL......AVDVY......
116057881   ......QVLSK......IVRMF......CIVEE......IVHRD......ADFGI......KCDVY......
116057805   ......SILRR......IIQLI......CLVTE......IIHRD......ADFGV......KADVY......
116059538   ......RILRK......VIGYV......CLITE......MMHRD......CDFGL......SADVY......
116060363   ......RMMRG......IVLFI......GIVSE......VVHHD......SDFGM......RSDVY......
116060512   ......AIMAR......VLAFY......DVVVE......VMHRD......ADFGL......RADVF......
116242625   ......KLFAM......IIALR......CLVME......IIHRD......TDFGL......GSDVW......
116256119   ......VVLAQ......IVKLL......VLVYE......IYHRD......SDFGT......KSDVY......
116256120   ......ILLSQ......IVKLI......ILVYE......IYHRD......SDFGT......KSDVY......
116309013   ......EVLRS......LMRPL......FLIFK......LVHCD......GDFGS......GGDVY......
116310216   ......LILSQ......IIKLL......MLVYE......ILHGD......TDFGA......KSDVY......
169866105   ......VIWSQ......VLPFY......CLVSP......MVHGD......ADFGL......YTDVY......
169860683   ......EIWAK......IVPFY......YMVSP......IVHGD......TDFGL......MSDMY......
169859400   ......VIWCQ......VLPFL......ILVSP......VVHGD......ADFGL......ESDVY......
169863351   ......ATWHK......VSQFF......GMVSP......IVHGD......TDFGL......MSDVY......
169852966   ......KLLGG......IVRFL......MIVSE......VIHRD......ADFGL......KVDVF......
169853811   ......KIWRK......VLTLY......YLVSP......IVHGD......ADFGL......KSDIW......
169851235   ......ILWSQ......VLPFY......CLLSP......FIHGD......ADFGL......QTDVY......
169854654   ......RIWRT......ILPLY......F----......VLHGD......SDFGQ......AIDVY......
115432146   ......VILSQ......VVKLL......LLVYE......IYHRD......ADFGT......KSDVY......
116643216   ......SILSR......VILFL......SLITE......IVHRD......CDFGL......KCDIF......
116643220   ......EILSK......VMAFY......ATVTE......IVHFD......GDFGL......KVDVF......
116643204   ......EILSK......VVAFY......TLVTE......IVHFD......GDFGL......KVDVF......
116643252   ......ALLQR......IVQFL......MIVTE......IIHRD......ADFGV......KADVF......
```

116643246	......DLLVK.....IVQFL.....MLITE.....IIHRD.....GDFGL.....KVDVF.....
116643254	......ALLQK.....VVQFL.....MIVTE.....IIHCD.....ADFGV.....KVDVF.....
116643256	......FIMRK.....VVQFL.....CIVTE.....IIHRD.....ADFGV.....KADVF.....
116643258	......YIMRK.....VVQFI.....CIVTE.....IIHRD.....ADFGV.....RADVF.....
116643268	......LVLSS.....IVRFV.....MIVTE.....IIHRD.....ADFGL.....KIDVY.....
116643270	......LLLSK.....IVKFV.....IIVTE.....IIHRD.....ADFGI.....KADIY.....
116643276	......TLLSR.....VIKFV.....CVLTQ.....IIHRD.....ADFGI.....KADVY.....
116643274	......TLLSR.....VVKFV.....CIITE.....IVHQD.....ADFGI.....KCDVY.....
116643284	......AVWHK.....VTKFI.....CVVVE.....IVHRD.....ADFGV.....KCDVY.....
116643286	......AVWHK.....VTKFI.....CVVVE.....IVHRD.....ADFGV.....KCDVY.....
116643282	......SMLAF.....IVRFI.....CIVTE.....FIHRD.....ADFGV.....KVDVY.....
116643292	......AVWQK.....VTKFI.....CVVVE.....IVHRD.....ADFGV.....KCDVY.....
118151106	......SILCR.....IIQFV.....FIVTQ.....IIHRD.....ADFGE.....KADVF.....
118088735	......RQLSR.....IVKLY.....CLVME.....LIHRD.....CDFGT.....KCDVF.....
118088739	......RQLSR.....IVKLY.....CLVME.....LIHRD.....CDFGT.....KCDVF.....
118088737	......RQLSR.....IVKLY.....CLVME.....LIHRD.....CDFGT.....KCDVF.....
118093629	......EILSV.....IIQFY.....GIVTE.....VIHRD.....CDFGA.....TCDTY.....
118094766	......SILCR.....VIQFV.....FIVTQ.....IIHRD.....ADFGE.....KADVF.....
118094861	......--LRK.....IIAFK.....CIIME.....IIHRD.....SDFGT.....KVDIW.....
161520187	......ACLHA.....MIPVI.....GLVME.....IMHGD.....GDFG-.....RLEVR.....
119637821	......RLFAM.....IIQLR.....CLVLE.....ILHRD.....TDFGL.....GSDIW.....
119890000	......SILCR.....IIQFV.....FIVTQ.....IIHRD.....ADFGE.....KADVF.....
119617121	......--LRK.....IITFK.....CILME.....IIHRD.....SDFGT.....KVDIW.....
119918343	......RLFSM.....IIELR.....CLVLE.....ILHRD.....TDFGL.....GSDIW.....
119927714	......KLFAM.....IIALR.....CLVME.....IIHRD.....TDFGL.....GSDVW.....
120612910	......QILPA.....APRFV.....YLAME.....VCHLD.....LDFGL.....RSDVF.....
153004262	......RLASR.....VVQVF.....YIAME.....LVHRD.....ADFGI.....RADVF.....
123394166	......YILSK.....LLQLL.....CIATE.....IMHRD.....CDFGI.....RVDIY.....
123417368	......SLHAS.....LVPFI.....CIVTQ.....IIHRD.....IDFGT.....SVDVY.....
123413816	......ETMAS.....VLELV.....RIITR.....IVHRD.....ADFGL.....KVDTF.....
123417280	......TILAS.....LLPFI.....TIVTE.....IIHRD.....CDFGI.....KVDVY.....
123413162	......FAMST.....LLPFC.....CLATK.....VIHRD.....SDFGF.....KIDIY.....
123431774	......ETMAS.....VLELV.....RIITR.....IVHRD.....ADFGL.....NVDTY.....
123428848	......KILAQ.....ILGFV.....SIITI.....VIHRD.....CDFGL.....KVDVY.....
123424874	......VTMGK.....IIPFI.....CIITE.....IIHRD.....CDFGI.....KVDVF.....
123429497	......YTNAV.....CIKLV.....SIITT.....IVHRD.....CDFGV.....QVDVY.....
123434767	......SILAT.....ILKFI.....CVVTQ.....IIHRD.....SDFGF.....KVDVY.....
123432043	......AILAH.....ILPFV.....CIITK.....MVHRD.....ADFGM.....KSDVY.....
123424576	......NCLMN.....CIKFY.....SIITK.....VLHRD.....CDFGL.....SCDTY.....
123434055	......EILAV.....LLPFF.....SIITE.....IIHRD.....CDFGL.....KIDVY.....
123430527	......EILIK.....LLDLI.....SILTS.....IIHRD.....ADFGL.....SVDVY.....
123438871	......AILSS.....LIEFV.....WIITD.....IIHRD.....CDFGI.....KADSF.....
123437528	......ANMAN.....VLELV.....RIITR.....IVHRD.....ADFGL.....KVDSY.....
123439284	......ENLAE.....ILPFL.....TIITK.....IMHRD.....CDFGL.....KVDVY.....
123439337	......NAYSR.....IVKFH.....MLITE.....SIHGD.....CDFGS.....AADVY.....
123455691	......ATMAK.....FVKFM.....SLLTE.....IIHRD.....CDFGI.....PVDVY.....
123471241	......ENLAI.....CLAFL.....SIITQ.....IIHRD.....CDFGL.....KVDVY.....
123474701	......EIQSK.....ILELV.....YIATE.....YIHRD.....CDFGM.....KCDVY.....
123477613	......ATMAK.....ILELV.....RIITR.....IVHRD.....ADFGL.....KVDTY.....
123474263	......AILAT.....VLKFI.....SIVTE.....IIHRD.....CDFGF.....KVDVY.....
123477963	......DSMLA.....LLKLA.....CIATG.....MVHRD.....CDFGL.....CVDVY.....
123476799	......EILAR.....LLPFY.....SIAME.....IINRD.....CDFGI.....KVDVY.....
123474038	......YFLTI.....LTKFC.....YICTE.....VIHRD.....CDFGM.....RVDVY.....
123472791	......STQIQ.....VIHLI.....TIIEE.....FIHRD.....CDFGI.....PSDVY.....
123475550	......VTMGK.....VIPFI.....CIITE.....IIHRD.....CDFGI.....KADVF.....
154412292	......GIMAK.....ILPFL.....LIITE.....ILHRD.....CDFGL.....LVDVY.....
123500391	......EIQAS.....ILELV.....YIATE.....YIHRD.....CDFGM.....KADVY.....
154411956	......SVFSR.....IVGFV.....CIITD.....IIHRD.....CDFGL.....KIDVY.....
123491938	......RILTS.....ITPFV.....YIITQ.....IMHRD.....CDFGC.....KVDIY.....
123497021	......SILAS.....LVHFV.....CIVTE.....IIHRD.....CDFGY.....MIDVY.....
123493371	......GILAG.....ILPFV.....CIVTE.....MLHRD.....CDFGM.....KADVY.....
123977179	......TLMKK.....ILELV.....CIITR.....IVHRD.....ADFGL.....KVDVY.....
123490093	......QIFSS.....ILPFV.....CLVTE.....IMHRD.....CDFGM.....KADVY.....
123505885	......RILAK.....LLHFT.....LIVTE.....IMHRD.....CDFGA.....AVDVY.....
123486416	......TTLST.....ILKLL.....CLIIE.....LIHRD.....CDFGF.....KVDVY.....
154413583	......SVLAE.....LLPFL.....SIITQ.....VIHRD.....CDFGL.....KVDVY.....
154420831	......FMTAC.....VIRLM.....CIITQ.....IVHRD.....CDFGI.....YADVF.....
154414271	......KILSR.....LIPFY.....IIAMK.....VIHRD.....CDFGI.....KVDIY.....
154421034	......YALSV.....ILKFC.....YILTE.....VIHRD.....CDFGF.....KVDVY.....
154415592	......SIFTK.....LLPFV.....YIVTE.....IIHRD.....CDFGM.....KADVY.....
154415835	......TIFSK.....ILPFV.....YIVTE.....IVHRD.....CDFGM.....KADVY.....
154416791	......DALMK.....TIRLI.....CIVTN.....LMHRD.....CDFGL.....SVDVY.....

```
154417504    ......RILAK.....LLHLN.....IIVTD.....IMHRD.....CDFGI.....KVDVH.....
123230058    ......RQLSR.....IVKLY.....CLVME.....LIHRD.....CDFGT.....KCDVF.....
123230057    ......RQLSR.....IVKLY.....CLVME.....LIHRD.....CDFGT.....KCDVF.....
145479981    ......EMLSL.....IVLLM.....VIAIE.....VVHRD.....CDFGL.....SVDLF.....
145529225    ......SAFVT.....LVQLM.....YIVTE.....LIHRD.....ADFGL.....KADVY.....
145534586    ......SAFVT.....LVQLM.....YIVTE.....LIHRD.....ADFGL.....KADVY.....
145536275    ......EMLSL.....IVLLM.....VIATE.....VVHRD.....CDFGL.....GVDLF.....
145540094    ......EMLSL.....IVLLM.....VIATE.....VVHRD.....CDFGL.....SVDLF.....
125991936    ......RQLSR.....IVKLY.....CLVME.....LIHRD.....CDFGT.....KCDVF.....
125600204    ......YIMKK.....VVQFI.....CIVTE.....IVHRD.....ADFGV.....RADVF.....
125580073    ......IILSQ.....IVRLL.....MLVYE.....ILHGD.....ADFGA.....RSDVY.....
125527702    ......DVLQL.....VVQFL.....MIVME.....IIHRD.....ADFDL.....KVDVF.....
125527690    ......TTLSR.....VIKLI.....CVITE.....VVHRD.....VDFGI.....KVDVY.....
125524806    ......NALSH.....VIKLV.....YIITE.....VVHRD.....ADFGI.....KVDVY.....
125533565    ......NTLRN.....LVPIL.....ALVYE.....IVHCD.....ADFGL.....ASDVF.....
125533573    ......NALRN.....LVPIL.....GLVYK.....IVHCD.....GDFGL.....ASDVY.....
125538717    ......KALKN.....LVKVI.....GLVFE.....VVHCD.....CDFGL.....EGDVY.....
125539863    ......DALTR.....IVKLF.....FLVYE.....IVHRD.....SDFGI.....KCDVY.....
125540113    ......TMLSR.....LVKFI.....VVVTE.....IIHRD.....VDLGL.....KVDVY.....
125538971    ......EVLTK.....IVKLH.....FLVYD.....IVHRD.....SDFGM.....KCDVY.....
125537252    ......QILSK.....VVAFY.....ATVTE.....IVHFD.....GDFGL.....KVDVF.....
125534757    ......EIMRK.....LVRLI.....YLVSE.....YVHMD.....RNFGG.....KADVY.....
125530974    ......EILTQ.....IVKLY.....FLVYE.....IIHRD.....SDFGT.....KCDVY.....
125535882    ......YILRE.....VVRFI.....CIITE.....IIHRD.....ADFGV.....KADVF.....
125534752    ......VIMSP.....ITKLL.....TLVYE.....IAHGD.....TAFTL.....KSDVY.....
125541394    ......QILSS.....VVSFY.....ATVTE.....IVHFD.....GDLGL.....KIDVY.....
125544723    ......AVWQK.....VTKFV.....VVVVE.....IVHRD.....ADFGV.....KCDVY.....
125545003    ......AVWHK.....VTKFV.....CVVVE.....IVHRD.....ADFGV.....KCDVY.....
125545484    ......IVHTQ.....VVRLV.....MIVFE.....IVHCD.....SDFGI.....KSGVY.....
125554309    ......DKLAS.....VVAFY.....ATVTE.....IVHFD.....GDLGL.....KVDVF.....
125556191    ......AFLSR.....IVQFI.....CIITE.....VIHRD.....ADFGT.....KVDVY.....
125556267    ......SKLAD.....VVAFY.....ATVTE.....IVHFD.....GDLGL.....KVDVF.....
125558147    ......EILSK.....VVAFY.....ATVTE.....IVHFD.....GDFGL.....KVDVF.....
125556373    ......MMLST.....IVRFI.....CIITE.....FIHRD.....ADFGV.....KVDVY.....
125558301    ......YIMKK.....VVQFI.....CIVTE.....IVHRD.....ADFGV.....RADVF.....
125559274    ......NLLIK.....IVQFL.....MLVTE.....VIHRD.....GDFGL.....KVDVF.....
125560587    ......MMLAT.....IVKFV.....CIVTE.....FIHRD.....ADFGV.....KVDVY.....
125570374    ......LILSQ.....IVKLL.....MLVYE.....ILHGD.....SDFGA.....KSDVY.....
125563895    ......DALSR.....VLRLM.....CLVTE.....VVHRD.....ADFGH.....KCDVY.....
125563245    ......ETLSK.....IVKLF.....LLVYE.....IIHRD.....ADFGV.....KSDVY.....
125570376    ......LILSQ.....IVKLL.....MLVYE.....ILHGD.....SDFGA.....KSDVY.....
125579933    ......QILSK.....VVAFY.....ATVTE.....IVHFD.....GDFGL.....KVDVF.....
125577512    ......EIMRK.....LVRLI.....YLVSE.....YVHMD.....RNFGG.....KADVY.....
125579377    ......ADFAA.....-----.....-----.....IVHRD.....ADFGV.....KCDVY.....
125571346    ......AVWHK.....VTKFI.....CVVVE.....IVHRD.....ADFGV.....KCDVY.....
125578603    ......YILRE.....VVRFI.....CIITE.....IIHRD.....ADFGV.....KADVF.....
125589818    ......AILSQ.....IVKLF.....LLVYD.....VFHRD.....ADFGA.....KSDVY.....
125585917    ......LQLMS.....ILQFH.....CIITR.....ITYRD.....GDMGI.....ACNVY.....
125587227    ......AVWHK.....VTKFV.....CVVVE.....IVHRD.....ADFGV.....KCDVY.....
125581645    ......EVLTK.....IVKLH.....FLVYD.....IVHRD.....SDFGM.....KCDVY.....
125583938    ......QILSS.....VVSFY.....ATVTE.....IVHFD.....GDLGL.....KIDVY.....
125582933    ......LILSQ.....IVKLY.....MLVYK.....IIHGD.....SDFGG.....-SDVY.....
125582489    ......DALTR.....IVKLF.....FLVYE.....IVHRD.....SDFGI.....KCDVY.....
125600305    ......IIQSQ.....IVRLL.....MLVTE.....ILHGD.....CDFGI.....KCDVY.....
125596260    ......DKLAS.....VVAFY.....ATVTE.....IVHFD.....GDLGL.....KVDVF.....
125598034    ......SKLAD.....VVAFY.....ATVTE.....IVHFD.....GDLGL.....KVDVF.....
125592137    ......MILRS.....VVQFY.....LIVTE.....IIHRD.....ADFGV.....KADVF.....
125598444    ......LILSQ.....IVKLL.....MLVYE.....ILHGD.....SDFGA.....KSDVY.....
125600038    ......EILSK.....VVAFY.....ATVTE.....IVHFD.....GDFGL.....KVDVF.....
125598136    ......MMLST.....IVRFI.....CIITE.....FIHRD.....ADFGV.....KVDVY.....
125591003    ......LILSQ.....IIKLL.....MLVYE.....ILHGD.....TDFGA.....KSDVY.....
125606041    ......LILSQ.....IVKLL.....MLVYE.....ILHGD.....SDFGA.....KSDVY.....
125601198    ......NLLIK.....IVQFL.....MLVTE.....VIHRD.....GDFGL.....KVDVF.....
125606638    ......AILSQ.....VVKLF.....LLVYE.....IIHRD.....SDFGA.....KSDVY.....
125606034    ......VILSQ.....IVKLL.....ILVYE.....ILHGD.....SDFGA.....KSDVY.....
125823570    ......SILCR.....VIQFV.....FIVTQ.....IIHRD.....ADFGE.....KADMF.....
125825487    ......EILSV.....IIKFY.....GIVTE.....VIHRD.....CDFGA.....TCDTF.....
125826685    ......--LRK.....IISFK.....CIIME.....IIHRD.....SDFGT.....KVDIW.....
125833227    ......KLFSM.....IIKLE.....CLVME.....IIHRD.....TDFGL.....GSDVW.....
125833621    ......KLFAM.....IMALL.....CLVME.....VIHRD.....TDFGL.....GSDVW.....
125843789    ......RLFWM.....IIALR.....CLVME.....IIHRD.....TDFGL.....SSDVW.....
```

```
126282421    ......KLFAM.....IIALR.....CLIME.....IIHRD.....TDFGL.....GSDVW.....
126310335    ......RQLSR.....IVKLY.....CLVME.....LIHRD.....CDFGT.....KCDVF.....
126310331    ......RQLSR.....IVKLY.....CLVME.....LIHRD.....CDFGT.....KCDVF.....
126310337    ......RQLSR.....IVKLY.....CLVME.....LIHRD.....CDFGT.....KCDVF.....
126310333    ......RQLSR.....IVKLY.....CLVME.....LIHRD.....CDFGT.....KCDVF.....
126305949    ......SILCR.....VIQFV.....FIVTQ.....IIHRD.....SDFGE.....KADVF.....
126314760    ......--LRK.....IIAFK.....CIIME.....IIHRD.....SDFGT.....KVDIW.....
126326325    ......EILSV.....IIQFY.....GIVTE.....VIHRD.....CDFGA.....TCDTY.....
126326323    ......EILSV.....IIQFY.....GIVTE.....VIHRD.....CDFGA.....TCDTY.....
126329325    ......RLFGA.....IIALR.....CLVME.....IIHRD.....TDFGL.....SSDVW.....
126338920    ......RLFAM.....IIALK.....CLVME.....VIHRD.....TDFGL.....GSDVW.....
126632512    ......RLFWM.....IIALR.....CLVME.....IIHRD.....TDFGL.....SSDVW.....
154147628    ......RQLSR.....IVKLY.....CLVME.....LIHRD.....CDFGT.....KCDVF.....
145347149    ......AVLSA.....IVRMF.....CIVEE.....IVHRD.....ADFGI.....KCDVF.....
145346568    ......NILRR.....IIQLI.....CLVTE.....IIHRD.....ADFGV.....KADVY.....
145350181    ......KMLRT.....LVGYV.....CLVTE.....VIHRD.....SDFGL.....KADVF.....
145351986    ......EIMAR.....VLAFY.....SVVCE.....VMHRD.....ADFGL.....RADVF.....
145355792    ......EIMRA.....IVKIY.....CLMLQ.....IIHRD.....SDFGV.....KVDVY.....
145352240    ......QILSR.....IVLWL.....TIVLE.....IVHCD.....TDFGL.....ASDVF.....
145354528    ......KIMQQ.....IVQFL.....TIVSE.....VIHRD.....GDFGL.....AVDVY.....
145351834    ......RMMRA.....IVLFL.....GIVSE.....VVHHD.....SDFGM.....LSDVF.....
147772468    ......DILSK.....VVAFY.....TLVTE.....IVHFD.....GDFGL.....KVDVF.....
147768303    ......AVWHK.....VTKFI.....CVVVE.....IVHRD.....ADFGV.....KCDVY.....
147777440    ......HALTQ.....IVKLY.....FLVYE.....IVHRD.....SDFGT.....KTDVY.....
147789160    ......VVWHK.....VTKIF.....---IE.....IVHRD.....ADFGV.....KCDVY.....
147790565    ......VILCQ.....VVKLL.....LLVYE.....IYHRD.....ADFGT.....KSDVY.....
147815532    ......MILSQ.....ILGLL.....LLVYE.....IYHRD.....SDFGT.....KSDVY.....
147832778    ......HALTH.....IVKLY.....FLVYE.....IVHRD.....FDFGT.....KTDVY.....
147827247    ......STLGK.....IVKLH.....LLLYE.....IIHRD.....GDFGL.....KCDIY.....
147839295    ......SMLSR.....LAKFI.....VIVTE.....IIHRD.....VDFGL.....KVDAY.....
147839316    ......MMLAT.....IVRFI.....CIVTE.....FIHRD.....ADFGV.....KVDVY.....
147841871    ......RILSN.....VVAFY.....ATVTE.....IVHFD.....CDFGL.....KVDVF.....
147844711    ......AILRE.....VVRFI.....CIVTE.....IIHRD.....ADFGV.....KADVF.....
147846115    ......AVWHK.....VTKFI.....CVVVE.....IVHRD.....ADFGV.....KCDVY.....
147856644    ......IKLAD.....VVAFY.....ATVTE.....IVHFD.....GDLGL.....KVDVF.....
147859359    ......TILSR.....IVKFI.....MIITE.....IIHRD.....CDFGL.....KVDVY.....
148670775    ......KLFAM.....IIALR.....CLVME.....IIHRD.....TDFGL.....GSDVW.....
148672025    ......--LRK.....IITFK.....CILME.....IIHRD.....SDFGT.....KVDIW.....
148679860    ......RLFAM.....IIQLR.....CLVLE.....ILHRD.....TDFGL.....GSDIW.....
156096474    ......KILYS.....VIKLL.....SLILQ.....FFHRD.....SDFGL.....ESDVW.....
156101111    ......EIYKN.....ICTFY.....MLLLE.....VINGD.....CDFGK.....KIDIW.....
149031915    ......--LRK.....IITFK.....CILME.....IIHRD.....SDFGT.....KVDIW.....
149043233    ......RLFAM.....IIQLR.....CLVLE.....ILHRD.....TDFGL.....GSDIW.....
149062096    ......RLFAM.....IIALK.....CLVME.....VIHRD.....TDFGL.....GSDVW.....
149263376    ......KLFAM.....IIALR.....CLVME.....IIHRD.....TDFGL.....GSDVW.....
149435971    ......--LRR.....IITFK.....CILME.....IIHRD.....SQMGL.....KVDIW.....
149577193    ......--LRK.....IIAFK.....CIIME.....IIHRD.....SDFGT.....KVDIW.....
149639683    ......EILSV.....VIQFY.....GIVTE.....VIHRD.....CDFGA.....TCDTY.....
149640552    ......RQLSR.....IVKLY.....CLVME.....LIHRD.....CDFGT.....KCDVF.....
149714959    ......--LRK.....IITFK.....CILME.....IIHRD.....SDFGT.....KVDIW.....
149722804    ......RQLSR.....IVKLY.....CLVME.....LIHRD.....CDFGT.....KCDVF.....
149722806    ......RQLSR.....IVKLY.....CLVME.....LIHRD.....CDFGT.....KCDVF.....
149722800    ......RQLSR.....IVKLY.....CLVME.....LIHRD.....CDFGT.....KCDVF.....
149722802    ......RQLSR.....IVKLY.....CLVME.....LIHRD.....CDFGT.....KCDVF.....
149725577    ......RLFAM.....IIALK.....CLVME.....VIHRD.....TDFGL.....GSDVW.....
149730730    ......EILSV.....IIQFY.....GIVTE.....VIHRD.....CDFGA.....TCDTY.....
149731180    ......--LRK.....IIAFK.....CIIME.....IIHRD.....SDFGT.....KVDIW.....
149737487    ......KLFAM.....IIALR.....CLVME.....IIHRD.....TDFGL.....GSDVW.....
149924437    ......RLAAR.....VVQTF.....YLTME.....VVHRD.....VDFGI.....RADVF.....
149922853    ......KSLAR.....IIEIY.....FLALE.....VMHRD.....TDFGL.....RADQF.....
149923746    ......KALAK.....IVQVY.....YIAME.....LVHRD.....MDFGL.....FSDQF.....
149923917    ......RLARR.....AARTY.....FLTME.....VVHRD.....TDFGI.....RTDIY.....
149923702    ......QCLAR.....VVAVY.....FLTME.....LEHGD.....ADFGV.....RSDQF.....
149922954    ......QALAR.....VIQVY.....YIAME.....LVHRD.....LDFGI.....RADQF.....
149922163    ......QSLAR.....VIAVY.....WIAME.....LVHRD.....IDFGL.....LADQY.....
149923530    ......QALAK.....VIPVY.....WIAME.....LVHRD.....IDFGL.....RADQY.....
149922154    ......KITGQ.....VVPVY.....FYTMR.....IIHRD.....LDWGL.....QTDVY.....
149921170    ......QALAK.....VVGVH.....WIAMQ.....LLHRD.....MDFGL.....TTDQF.....
149920057    ......KVVVR.....LVQVF.....YLAME.....LVHRD.....TDFGL.....RTDIY.....
149919882    ......QALAK.....VVQIY.....FIAME.....LVHRD.....MDFGL.....RTDQF.....
```

149920652	......RALAR......VVAVY......YIVLE......LIHRD......ADFGL......ASDQF......
149920693	......QLLAQ......VVKVH......FLVLE......LSHRD......IDFGL......RSDQF......
149919007	......MVLAK......VVTVY......YIVMD......ILHGD......ADFGV......RSDQF......
156120631	......RLFAM......IIALK......CLVME......VIHRD......TDFGL......GSDVW......
157142160	......AMLKK......ILLFM......AIVTQ......IIHRD......GDFGL......QSDVY......
157119220	......ATFKK......VVLFM......AIVTS......IVHKD......TDFGL......SSDIF......
109034321	......AVLRK......ILLFM......AIVTQ......IIHRD......GDFGL......QSDVY......
109098922	......MAYRQ......VVLFM......AIITS......ILHKD......TDFGL......HSDVF......
109113709	......MNYRQ......VVLFM......AIITS......IVHKD......TDFGL......AADVY......
109471941	......GVLRK......ILLFM......AIVTQ......IIHRD......GDFGL......QSDVY......
109473380	......GVLRK......ILLFM......AIVTQ......IIHRD......GDFGL......QSDVY......
109497448	......MAYRQ......VVLFM......AIITS......ILHKD......TDFGL......HSDVF......
109496040	......MAYRQ......VVLFM......AIITS......ILHKD......TDFGL......HSDVF......
114585482	......AVLRK......ILLFM......AIVTQ......IIHRD......GDFGL......QSDVY......
114585478	......AVLRK......ILLFM......AIVTQ......IIHRD......GDFGL......QSDVY......
114585472	......AVLRK......ILLFM......AIVTQ......IIHRD......GDFGL......QSDVY......
114585480	......AVLRK......ILLFM......AIVTQ......IIHRD......GDFGL......QSDVY......
114616352	......GVLRK......ILLFM......AIVTQ......IIHRD......GDFGL......QSDVY......
114616356	......GVLRK......ILLFM......AIVTQ......IIHRD......GDFGL......QSDVY......
114647294	......MAYRQ......VVLFM......AIITS......ILHKD......TDFGL......HSDVF......
115292039	......GVLRK......ILLFM......RLVTQ......IIHRD......GDFGL......QSDVY......
115953064	......AVLRK......ILLFM......AIVTQ......IIHRD......GDFGL......QSDVY......
118100376	......MNYRQ......VVLFM......AIITS......IVHKD......TDFGL......AADIY......
90075856	......QVLRK......ILLFM......AIITQ......IIHRD......GDFGL......QSDVY......
119571420	......MNYRQ......VVLFM......AIITS......IVHKD......TDFGL......AADVY......
119891390	......GVLRK......ILLFM......AIVTQ......IIHRD......GDFGL......QSDVY......
119618518	......MAYRQ......VVLFM......AIITS......ILHKD......TDFGL......HSDVF......
119909298	......MAYRQ......VVLFM......AIITS......ILHKD......TDFGL......HSDVF......
122890770	......MAYRN......VVLFM......AIITS......ILHKD......TDFGL......QSDVF......
122920151	......GVLRK......ILLFM......AIVTQ......IIHRD......GDFGL......QSDVY......
125815601	......MAYRN......VVLFM......AIITS......ILHKD......TDFGL......QSDVF......
125838149	......MNYRQ......VVLFM......AIITS......IVHKD......TDFGL......AADVY......
126314007	......MNYRQ......VVLFM......AIITS......IVHKD......TDFGL......AADVY......
126324698	......MAYRQ......VVLFM......AIITS......ILHKD......TDFGL......HSDVF......
126336205	......AVLRK......ILLFM......AIVTQ......IIHRD......GDFGL......QSDVY......
147647674	......MAYRQ......VVLFM......AIITS......ILHKD......TDFGL......HSDVF......
104745831	......MAYRQ......VVLFM......AIITS......ILHKD......TDFGL......HSDVF......
148668415	......QVLRK......ILLFM......AIITQ......IIHRD......GDFGL......QSDVY......
148667117	......AVLRK......ILLFM......AIVTQ......IIHRD......GDFGL......QSDVY......
148683655	......MNYRQ......VVLFM......AIITS......IVHKD......TDFGL......AADVY......
148681648	......GVLRK......ILLFM......AIVTQ......IIHRD......GDFGL......QSDVY......
148886599	......MAYRQ......VVLFM......AIITS......ILHKD......TDFGL......HSDVF......
149044395	......QVLRK......ILLFM......AIITQ......IIHRD......GDFGL......QSDVY......
149044389	......QVLRK......ILLFM......AIITQ......IIHRD......GDFGL......QSDVY......
157818965	......MNYRQ......VVLFM......AIITS......IVHKD......TDFGL......AADVY......
149065321	......GVLRK......ILLFM......AIVTQ......IIHRD......GDFGL......QSDVY......
149618766	......GVLRK......ILLFM......AIVTQ......IIHRD......GDFGL......QSDVY......
149728262	......AVLRK......ILLFM......AIVTQ......IIHRD......GDFGL......QSDVY......
149729652	......AVLHK......VLLFM......AIVTQ......IIHRD......GDFGL......QSDVY......
149744596	......QVLRK......ILLFM......AIITQ......IIHRD......GDFGL......QSDVY......
149747615	......GVLRK......ILLFM......AIVTQ......IIHRD......GDFGL......QSDVY......
109065252	......KKMEM......ILPVY......GLVME......LLHLD......SDFGL......KHDVY......
109069441	......KMMNR......VVKLL......SLVME......VIHKD......ADLGL......KSDVY......
109083189	......KAMAS......VLRLE......ALVTK......LLHRD......ADFGL......ASDVY......
109086891	......EILHK......ILPIL......GIVTE......LLHHD......ADFGL......KHDIY......
109476182	......EILHK......ILPIL......GIVTE......LLHHD......ADFGL......KHDIY......
109474681	......EILHK......ILPIL......GIVTE......LLHHD......ADFGL......KHDIY......
157819567	......KKMEM......ILPVY......GLVME......LLHLD......SDFGL......KHDVY......
157824041	......KMMHR......VVKLL......SLVME......VIHKD......ADLGV......KSDVY......
112821694	......SLMSR......VVKLL......SLVME......VIHKD......ADLGL......KSDVY......
118404966	......KIMHM......VVKLL......SLVME......VVHKD......ADLGL......KSDVY......
114605235	......KMMNR......VVKLL......SLVME......VIHKD......ADLGL......KSDVY......
114605229	......KMMNR......VVKLL......SLVME......VIHKD......ADLGL......KSDVY......
114605233	......KMMNR......VVKLL......SLVME......VIHKD......ADLGL......KSDVY......
114620835	......EILHK......ILPIL......GIVTE......LLHHD......ADFGL......KHDIY......
114652412	......KAMAS......VLRLE......ALVTK......LLHRD......ADFGL......ASDVY......
114652410	......KAMAS......VLRLE......ALVTK......LLHRD......ADFGL......ASDVY......
114684393	......KKMEM......ILPVY......GLVME......LLHLD......SDFGL......KHDVY......
118083928	......RKMEM......ILPVY......GLVME......LLHLD......SDFGL......KHDVY......

```
118102013    ......TKMEK.....IVTIY.....GIVME.....LLHLD.....SDFGL.....KYDVY......
119575522    ......KMMNR.....VVKLL.....SLVME.....VIHKD.....ADLGL.....KSDVY......
119587620    ......AKMKK.....IVSIY.....GIVME.....LLHLD.....SDFGL.....KYDVY......
119629996    ......KKMEM.....ILPVY.....GLVME.....LLHLD.....SDFGL.....KHDVY......
119629994    ......KKMEM.....ILPVY.....GLVME.....LLHLD.....SDFGL.....KHDVY......
148235198    ......KIMHM.....VVKLL.....SLVME.....VVHKD.....ADLGV.....KSDVF......
125820276    ......ELMFD.....VVRVL.....GLVLE.....ILHLD.....TDFGL.....AFDVY......
126322447    ......KILHK.....ILPIL.....GIVTE.....LLHHD.....ADFGL.....KHDIY......
126322419    ......KIMHR.....VVKFL.....SLVME.....VIHKD.....ADLGV.....KSDVY......
126325259    ......KKMEM.....ILPVY.....GLVME.....LLHLD.....SDFGL.....KHDVY......
126327014    ......SKMEK.....IVSIY.....GIVME.....LLHLD.....SDFGL.....KYDVY......
146455171    ......EVLHK.....IIQIF.....CIITE.....LLHHD.....ADFGL.....KYDMY......
148673643    ......EILHK.....ILPIL.....GIVTE.....LLHHD.....ADFGL.....KHDIY......
148704288    ......KAMVN.....VLLLL.....SLVTR.....LLHRD.....ADFGL.....ASDVY......
148708934    ......KMMHR.....VVKLL.....SLVME.....VIHKD.....ADLGV.....KSDVY......
148704289    ......KAMVN.....VLLLL.....SLVTR.....LLHRD.....ADFGL.....ASDVY......
157823567    ......VKMEK.....IVSIY.....GIVME.....LLHLD.....SDFGL.....EYDVY......
149064015    ......KAMVN.....VLLLL.....ALVTG.....LLHRD.....ADFGL.....ASDVY......
149633829    ......KKMEM.....ILPVY.....GLVME.....LLHLD.....SDFGL.....KHDVY......
149636491    ......KIMHK.....VVKLL.....SLVME.....VIHKD.....ADLGV.....KSDVY......
149731912    ......RMMHR.....VVKLL.....ALVME.....VIHKD.....ADLGV.....KSDVY......
149755703    ......EILHK.....ILPIL.....GIVTE.....LLHHD.....ADFGL.....KHDIY......
149756212    ......KAMAN.....ILLLL.....SLVTA.....LLHRD.....ADFGL.....ASDVY......
157131637    ......EIYST.....ILGYI.....WLITH.....IAHRD.....ADFGL.....KADIY......
157122986    ......EIYQT.....ILGFI.....WLITD.....ISHRD.....ADFGL.....QADMY......
157167387    ......--LPR.....ILQFI.....WLITA.....IAHRD.....ADFGL.....RIDVY......
157115961    ......--LPR.....ILQFI.....WLITA.....IAHRD.....ADFGL.....RIDVY......
157104776    ......DIYPL.....LLSYF.....LLVLS.....VCHRD.....CDLGF.....QIDVY......
157123443    ......EIYQM.....ILGFI.....WLVTD.....IAHRD.....GDLGL.....RADVY......
108755896    ......EIYQM.....ILGFI.....WLVSD.....IAHRD.....ADLGL.....RADIY......
108755894    ......EIYQM.....ILGFI.....WLVSD.....IAHRD.....ADLGL.....RADIY......
108755900    ......EIYQM.....ILGFI.....WLVSD.....IAHRD.....ADLGL.....RADIY......
67773459     ......--VPG.....ILQYY.....WLVTE.....IAHRD.....ADFGL.....RIDVY......
109041732    ......---PG.....LLQFI.....WLITA.....IAHRD.....ADFGL.....RIDMY......
109052596    ......N----.....ILQFL.....WLITA.....IVHRD.....CDFGL.....QTDVY......
109052590    ......N----.....ILQFL.....WLITA.....IVHRD.....CDFGL.....QTDVY......
109052593    ......N----.....ILQFL.....WLITA.....IVHRD.....CDFGL.....QTDVY......
109075025    ......EIYQT.....ILGFI.....WLITD.....IAHRD.....ADLGL.....MADMY......
109089085    ......EIYQT.....ILGFI.....WLITD.....IAHRD.....ADLGL.....MADIY......
109089089    ......EIYQT.....ILGFI.....WLITD.....IAHRD.....ADLGL.....MADIY......
109096731    ......EIYQM.....ILGFI.....WLVSD.....IAHRD.....ADLGL.....CADIY......
109096723    ......EIYNT.....ILGFI.....WLITH.....IAHRD.....ADLGL.....WTDIW......
109096930    ......ALYPG.....IVRFI.....LLVLE.....IAHRD.....GDLGL.....RADIY......
109096729    ......EIYQM.....ILGFI.....WLVSD.....IAHRD.....ADLGL.....CADIY......
109099640    ......YSLPG.....ILQFI.....WLITA.....ISHRD.....ADFGL.....RIDMY......
109099648    ......YSLPG.....ILQFI.....WLITA.....ISHRD.....ADFGL.....RIDMY......
109099768    ......EIYQM.....ILGFI.....WLVSE.....IAHRD.....ADLGL.....RADIY......
109099774    ......ECCFT.....ITLHL.....WLVSE.....IAHRD.....ADLGL.....RADIY......
109099776    ......ELYNM.....ILGFI.....WLITH.....IAHRD.....ADLGL.....RVDIW......
109099782    ......ELYNM.....ILGFI.....WLITH.....IAHRD.....ADLGL.....RVDIW......
109099772    ......EIYQM.....ILGFI.....WLVSE.....IAHRD.....ADLGL.....RADIY......
109099770    ......EIYQM.....ILGFI.....WLVSE.....IAHRD.....ADLGL.....RADIY......
109100616    ......NIYPL.....IARFI.....LLVME.....ISHRD.....SDFGL.....QVDMY......
109100614    ......NIYPL.....IARFI.....LLVME.....ISHRD.....SDFGL.....QVDMY......
109110805    ......EIYQM.....ILGFI.....WLVSD.....IAHRD.....ADLGL.....RADIY......
109110803    ......EIYQM.....ILGFI.....WLVSD.....IAHRD.....ADLGL.....RADIY......
108947450    ......EIYQT.....ILGFI.....WLITD.....IAHRD.....ADLGL.....MADMY......
109486138    ......NIYPL.....IARFI.....LLVME.....ISHRD.....SDFGL.....ESTVY......
109487151    ......NIYPL.....IARFI.....LLVME.....ISHRD.....SDFGL.....QVDMY......
110293552    ......EIYQT.....ILGFI.....WLITD.....IAHRD.....ADLGL.....MADMY......
118572826    ......AVYQL.....IVRFI.....LLVLE.....IAHRD.....GDLGL.....RADVY......
110755721    ......--LAH.....ILRFI.....WLITS.....VAHRD.....ADFGL.....RIDMY......
118404660    ......EIYQT.....ILGFI.....WLITD.....IAHRD.....ADLGL.....MADMY......
114581315    ......EIYQM.....ILGFI.....WLVSE.....IAHRD.....ADLGL.....RADIY......
114581313    ......EIYQM.....ILGFI.....WLVSE.....IAHRD.....ADLGL.....RADIY......
114581317    ......ECCFT.....ITLHL.....WLVSE.....IAHRD.....ADLGL.....RADIY......
114582697    ......NIYPL.....IARFI.....LLVME.....ISHRD.....SDFGL.....QVDMY......
114581321    ......ELYNM.....ILGFI.....WLITH.....IAHRD.....ADLGL.....RVDIW......
114585824    ......N----.....ILQFL.....WLITA.....IVHRD.....CDFGL.....QTDVY......
114586062    ......---PG.....LLQFI.....WLITA.....IAHRD.....ADFGL.....RIDMY......
114595226    ......EIYQT.....ILGFI.....WLITD.....IAHRD.....ADLGL.....MADMY......
```

114609276	......EIYQT.....ILGFI......YLITD.....IAHRD......ADLGL......MAGIY......
114609274	......EIYQT.....ILGFI......YLITD.....IAHRD......ADLGL......MAGIY......
114625816	......EIYQM.....ILGFI......WLVSD.....IAHRD......ADLGL......RADIY......
114625822	......EIYQM.....ILGFI......WLVSD.....IAHRD......ADLGL......RADIY......
114625818	......EIYQM.....ILGFI......WLVSD.....IAHRD......ADLGL......RADIY......
114625820	......EIYQM.....ILGFI......WLVSD.....IAHRD......ADLGL......RADIY......
114631588	......EIYQT.....ILGFI......WLITD.....IAHRD......ADLGL......MADIY......
114631584	......EIYQT.....ILGFI......WLITD.....IAHRD......ADLGL......MADIY......
114644477	......ALYPG.....IVRFI......LLVLE.....IAHRD......GDLGL......RADIY......
114644617	......EIYQM.....ILGFI......WLVSD.....IAHRD......ADLGL......CADIY......
114644619	......EIYQM.....ILGFI......WLVSD.....IAHRD......ADLGL......CADIY......
114644629	......EIYNT.....ILGFI......WLITH.....IAHRD......ADLGL......WTDIW......
116003817	......EIYQT.....ILGFI......WLITD.....IAHRD......ADLGL......MADIY......
115618702	......YSLPH.....LLHFI......WLISE.....VAHRD......ADFGL......RIDMY......
115722921	......EIYQM.....ILGFI......WLVSD.....IAHRD......ADLGL......RADVY......
115728478	......EIYNM.....ILAFF......WLIME.....IAHRD......ADLGL......RVDVY......
115728544	......DIMPH.....LLQYV......LLVVD.....VVHRD......GDFGF......QVDVY......
115944926	......YSLPH.....LLHFI......WLISE.....VAHRD......ADFGL......RIDMY......
115976785	......SV--M.....IVQFI......WLITK.....VAHRD......ADFGL......QMDTY......
118789116	......EIYQM.....ILGFI......WLVTD.....IAHRD......GDLGL......RADVY......
118781140	......--LPR.....ILEFI......WLITA.....IAHRD......ADFGL......RIDVY......
118777191	......EIYQM.....ILGFI......WLVTD.....IAHRD......GDLGL......RADVY......
117616720	......N----.....ILQFL......WLITA.....IVHRD......CDFGL......QTDVY......
117616204	......EIYQM.....ILGFI......WLVSE.....IAHRD......ADLGL......RADIY......
117616196	......EIYNT.....ILGFI......WLITH.....IAHRD......ADLGL......WTDIW......
117650789	......-LTPG.....LLRYI......WLISE.....IAHRD......GDFGL......RIDMY......
118093959	......EIYQM.....ILGFI......WLVSE.....IAHRD......ADLGL......RADIY......
118129619	......EIYQM.....ILGFI......WLVSD.....IAHRD......ADLGL......CADIY......
45825344	......--LCG.....VLTFI......WLITD.....IAHRD......SDFGL......RIDMY......
98978984	......DIYPL.....IAHFL......FVVVE.....VAHRD......CDFGC......QADIY......
116110422	......EIYST.....ILGFI......WLMTH.....MAHRD......ADFGL......RTDIY......
116110460	......EIYST.....ILGFI......WLMTH.....MAHRD......ADFGL......RTDIY......
116110420	......EIYST.....ILGFI......WLMTH.....MAHRD......ADFGL......RTDIY......
116110424	......EIYST.....ILGFI......WLMTH.....MAHRD......ADFGL......RTDIY......
119578618	......EIYNT.....ILGFI......WLITH.....IAHRD......ADLGL......WTDIW......
119578616	......EIYNT.....ILGFI......WLITH.....IAHRD......ADLGL......WTDIW......
119887678	......EIYQM.....ILGFI......WLVSE.....IAHRD......ADLGL......RADIY......
119888299	......NIYPL.....IARFI......LLVME.....ISHRD......SDFGL......QVDMY......
119892128	......EIYQM.....ILGFI......WLVSD.....IAHRD......ADLGL......CADIY......
157427784	......EIYQT.....ILGFI......WLITD.....IAHRD......ADLGL......MADMY......
161333831	......EIYQM.....ILGFI......WLVSE.....IAHRD......ADLGL......RADIY......
122890144	......ECCFT.....ITLHL......WLVSE.....IAHRD......ADLGL......RADIY......
125991744	......ELYNM.....ILGFI......WLITH.....IAHRD......ADLGL......RVDIW......
125808355	......DIYLT.....ILHYI......WLVTE.....IAHRD......ADFGL......RIDMY......
125818018	......EIYNG.....LLHFI......WLITT.....IAHRD......ADFGL......RIDMY......
125820034	......EIYQM.....ILGFI......WLVSD.....IAHRD......ADLGL......CADIY......
125844349	......E----.....VLQFL......WLITA.....IAHRD......CDFGL......QADVY......
125863346	......N----.....ILQFL......WLITA.....IVHRD......CDFGL......QTDVY......
126272348	......EIYQT.....ILGFI......WLITD.....IAHRD......ADLGL......MADIY......
126326331	......YSLPG.....ILQFI......WLITA.....ISHRD......ADFGL......RIDMY......
126326424	......EIYQM.....ILGFI......WLVSE.....IAHRD......ADLGL......RSDIY......
126326430	......ELYNT.....ILGFI......WLITH.....ISHRD......ADLGL......RVDIW......
126330686	......EIYQT.....ILGFI......WLITD.....IAHRD......ADLGL......MADMY......
126335117	......EIYQM.....ILGFI......WLVSD.....IAHRD......ADLGL......RADIY......
126336552	......---PG.....LLQFI......WLITA.....IAHRD......ADFGL......RIDMY......
126338037	......NIYPL.....IARFI......LLVME.....ISHRD......SDFGL......QVDMY......
126341742	......N----.....ILQFL......WLITA.....IVHRD......CDFGL......QTDVY......
134085683	......EIYNT.....ILGFI......WLITH.....IAHRD......ADLGL......WTDIW......
157278531	......DIYPL.....IAHFL......FVVVE.....VAHRD......CDFGC......QADIY......
148672036	......AVYQL.....IVRFI......LLVLE.....IAHRD......GDLGL......RADVY......
148672117	......EIYNT.....ILGFI......WLITH.....IAHRD......ADLGL......WTDIW......
148672115	......EIYNT.....ILGFI......WLITH.....IAHRD......ADLGL......WTDIW......
148672037	......AVYQL.....IVRFI......LLVLE.....IAHRD......GDLGL......RADVY......
148672113	......EIYQM.....ILGFI......WLVSD.....IAHRD......ADLGL......CADIY......
148677269	......---PG.....LLQFI......WLITA.....IAHRD......ADFGL......RIDMY......
148677270	......---PG.....ILQFI......WLITA.....IAHRD......ADFGL......RIDMY......
148677271	......---PG.....LLQFI......WLITA.....IAHRD......ADFGL......RIDMY......
148692933	......EIYQT.....ILGFI......WLITD.....IAHRD......ADLGL......MADIY......
148694995	......ELYNM.....ILGFI......WLITH.....IAHRD......ADLGL......RVDIW......

149018272	......---PG.....LLQFI.....WLITA.....IAHRD.....ADFGL.....RIDMY.....
149018271	......---PG.....LLQFI.....WLITA.....IAHRD.....ADFGL.....RIDMY.....
149020220	......EIYQM.....ILGFI.....WLVSD.....IAHRD.....ADLGL.....RADIY.....
149018316	......N----.....ILQFL.....WLITA.....IVHRD.....CDFGL.....QTDVY.....
149018315	......N----.....ILQFL.....WLITA.....IVHRD.....CDFGL.....QTDVY.....
149031998	......EIYNT.....ILGFI.....WLITH.....IAHRD.....ADLGL.....WTDIW.....
149031997	......EIYQM.....ILGFI.....WLVSD.....IAHRD.....ADLGL.....CADIY.....
149034102	......EIYQT.....ILGFI.....WLITD.....IAHRD.....ADLGL.....MADIY.....
149047788	......EIYQM.....ILGFI.....WLVSE.....IAHRD.....ADLGL.....RADIY.....
149047786	......ELYNM.....ILGFI.....WLITH.....IAHRD.....ADLGL.....RVDIW.....
149584431	......EIYQM.....ILGFI.....WLVSD.....IAHRD.....ADLGL.....RADIY.....
149635743	......EIYQT.....ILGFI.....WLITD.....IAHRD.....ADLGL.....MADIY.....
149635741	......EIYQT.....ILGFI.....WLITD.....IAHRD.....ADLGL.....MADIY.....
149639405	......N----.....VLQFL.....WLITA.....IVHRD.....CDFGL.....QTDVY.....
149639480	......ELYNT.....ILGFI.....WLITH.....ISHRD.....ADLGL.....RVDIW.....
149639482	......ELYNT.....ILGFI.....WLITH.....ISHRD.....ADLGL.....RVDIW.....
149639586	......EIYQM.....ILGFI.....WLVSE.....IAHRD.....ADLGL.....RADIY.....
149690866	......EIYQT.....ILGFI.....WLITD.....IAHRD.....ADLGL.....MADIY.....
149701574	......EIYQT.....ILGFI.....WLITD.....IAHRD.....ADLGL.....MADMY.....
149714887	......EIYQM.....ILGFI.....WLVSD.....IAHRD.....ADLGL.....CADIY.....
149714723	......EIYNT.....ILGFI.....WLITH.....IAHRD.....ADLGL.....WTDIW.....
149729815	......N----.....ILQFL.....WLITA.....IVHRD.....CDFGL.....QTDVY.....
149729765	......---PG.....LLQFI.....WLITA.....IAHRD.....ADFGL.....RIDMY.....
149730855	......EIYQM.....ILGFI.....WLVSE.....IAHRD.....ADLGL.....RADIY.....
149730637	......ELYNM.....ILGFI.....WLITH.....IAHRD.....ADLGL.....RVDIW.....
149740375	......EIYQM.....ILGFI.....WLVSD.....IAHRD.....ADLGL.....RADIY.....
149755031	......NIYPL.....IAHFV.....LLVME.....ISHRD.....SDFGL.....QVDMY.....
157141863	......KIEKF.....LKHMP.....FLVMP.....YVHAD.....VDFGL.....RGDME.....
157110088	......KIEKF.....LKHMP.....FLVMP.....YVHAD.....VDFGL.....RGDME.....
157116563	......KLYKV.....IPHIR.....VLVMD.....FIHRD.....IDFGL.....RDDME.....
157116475	......KIEKF.....LKHMP.....FLVMP.....YVHAD.....VDFGL.....RGDME.....
157105688	......RFYKL.....IPEVY.....ALVME.....LIYRD.....IDFGL.....RDDLE.....
157105686	......RFYKL.....IPEVY.....ALVME.....LIYRD.....IDFGL.....RDDLE.....
157105690	......RFYKL.....IPEVY.....ALVME.....LIYRD.....IDFGL.....RDDLE.....
157130000	......KRIES.....MPEYI.....FLILK.....YVHSD.....IDFGL.....RSDLE.....
157130002	......KRIES.....MPEYI.....FLILK.....YVHSD.....IDFGL.....RSDLE.....
113462010	......RFYKQ.....IPQVY.....AMVLE.....LIYRD.....IDFGL.....RDDLE.....
109078390	......RFYKQ.....IPQVY.....AMVLE.....LIYRD.....IDFGL.....RDDLE.....
109078388	......RFYKQ.....IPQVY.....AMVLE.....LIYRD.....IDFGL.....RDDLE.....
109078386	......RFYKQ.....IPQVY.....AMVLE.....LIYRD.....IDFGL.....RDDLE.....
109079304	......KLYKI.....IPHIR.....VLVMD.....FIHRD.....IDFGL.....RDDME.....
109079312	......KLYKI.....IPHIR.....VLVMD.....FIHRD.....IDFGL.....RDDME.....
109081486	......RFYKQ.....LPQVY.....AMVLE.....LIYRD.....IDFGL.....RDDLE.....
109081490	......RFYKQ.....LPQVY.....AMVLE.....LIYRD.....IDFGL.....RDDLE.....
109081482	......RFYKQ.....LPQVY.....AMVLE.....LIYRD.....IDFGL.....RDDLE.....
109081488	......RFYKQ.....LPQVY.....AMVLE.....LIYRD.....IDFGL.....RDDLE.....
109081484	......RFYKQ.....LPQVY.....AMVLE.....LIYRD.....IDFGL.....RDDLE.....
109081492	......RFYKQ.....LPQVY.....AMVLE.....LIYRD.....IDFGL.....RDDLE.....
109084814	......HKLKY.....VPKYW.....FMIMD.....YVHGD.....VDYGL.....RGDLE.....
109084812	......HKLKY.....VPKYW.....FMIMD.....YVHGD.....VDYGL.....RGDLE.....
109103047	......KQLDY.....IPLFY.....FMVIE.....YVHGD.....ADYGL.....RSDVE.....
109119253	......KIYKM.....IPTIR.....VMVME.....FIHRD.....IDFGL.....RDDLE.....
109120496	......KLYTI.....IPHMH.....VLVMD.....FLHRD.....IDFGL.....RDDME.....
115482508	......KVYMQ.....IPHMK.....VMVID.....FIHRD.....IDYGL.....RDDLE.....
110591041	......RFYKQ.....LPQVY.....AMVLE.....LIYRD.....IDFGL.....RDDLE.....
110751147	......RFYKL.....VPEVY.....ALVME.....LIYRD.....IDFGL.....RDDLE.....
110760677	......KFYRM.....IPTIK.....VMVME.....FIHRD.....IDFGL.....RDDLE.....
169595176	......RVYKS.....IPFVR.....AMVLD.....FIHRD.....IDFGL.....RDDME.....
169604014	......RTYKI.....IPNVY.....ILVID.....LIYRD.....VDFGM.....RDDLE.....
118151288	......KFYKM.....IPSIK.....VMVME.....FIHRD.....IDFGL.....RDDLE.....
113951753	......RFYKQ.....VPQVF.....AMVLE.....LIYRD.....IDFGL.....RDDLE.....
113195362	......KKSHH.....VITCK.....FLVIN.....YSHGD.....VDYGL.....RGDLE.....
90660416	......KKSHN.....LITCK.....FLVIN.....YSHGD.....VDYGL.....RGDLE.....
94484020	......KKSHH.....VITCK.....FLVIN.....YSHGD.....VDYGL.....RGDLE.....
94487454	......KKSHH.....VITCK.....FLVIN.....YSHGD.....VDYGL.....RGDLE.....
94486238	......KKSHH.....VITCK.....FLVIN.....YSHGD.....VDYGL.....RGDLE.....
113462019	......RFYKQ.....IPQVY.....AMVLE.....LIYRD.....IDFGL.....RDDLE.....
113462008	......RFYKQ.....IPQVY.....AMVLE.....LIYRD.....IDFGL.....RDDLE.....
115439889	......QVYNA.....VPRVH.....VIVMD.....YVHGD.....VDLGL.....RDDLE.....
115435096	......QVYNT.....VPRVH.....IMVMD.....YVHGD.....VDLGL.....RDDLE.....

115462563	......QVYNT......VPRVH......IMIMD......YVHGD......VDLGL......RDDLE......
115386142	......RVYKS......IPFVR......AMVID......FIHRD......IDFGL......RDDME......
115389170	......RTYKI......IPNVY......ILVID......LIYRD......VDFGM......RDDLE......
114577474	......KQLDY......IPLFY......FMVME......YVHGD......ADYGL......RSDVE......
114602751	......KLYKI......IPHIR......VLVMD......FIHRD......IDFGL......RDDME......
114654672	......RKLKY......VPKYW......FMIMD......YVHGD......VDYGL......RGDLE......
114654675	......RKLKY......VPKYW......FMIMD......YVHGD......VDYGL......RGDLE......
114657556	......RFYKQ......LPQVY......AMVLE......LIYRD......IDFGL......RDDLE......
114657568	......RFYKQ......LPQVY......AMVLE......LIYRD......IDFGL......RDDLE......
114657564	......RFYKQ......LPQVY......AMVLE......LIYRD......IDFGL......RDDLE......
114657570	......RFYKQ......LPQVY......AMVLE......LIYRD......IDFGL......RDDLE......
114657554	......RFYKQ......LPQVY......AMVLE......LIYRD......IDFGL......RDDLE......
114657566	......RFYKQ......LPQVY......AMVLE......LIYRD......IDFGL......RDDLE......
114657572	......RFYKQ......LPQVY......AMVLE......LIYRD......IDFGL......RDDLE......
114671106	......KIYKM......IPTIR......VMVME......FIHRD......IDFGL......RDDLE......
114671110	......KIYKM......IPTIR......VMVME......FIHRD......IDFGL......RDDLE......
114671104	......KIYKM......IPTIR......VMVME......FIHRD......IDFGL......RDDLE......
114686378	......KFYKM......IPSIK......VMVME......FIHRD......IDFGL......RDDLE......
114794862	......RFYKQ......IPQVY......AMVLE......LIYRD......IDFAL......RDDLE......
115503220	......LIEKW......IPTFY......FIIID......YTHSD......LDYGL......RGDLE......
115658185	......KVYRI......IPKIR......VLVME......FIHRD......IDFGL......RDDLE......
115675584	......RFYKM......IPQVH......AMVME......LIYRD......IDFGL......RDDLE......
116061767	......SVYNS......VPKVH......VMVMD......FVHGD......VDLGL......RDDLE......
116061773	......RLYKI......VPNVR......IMVMD......FIHRD......IDFGL......RDDLE......
116283282	......KLYKI......IPHIR......VLVMD......FIHRD......IDFGL......RDDME......
116283643	......KQLDY......IPLFY......FMVME......YVHGD......ADYGL......RSDVE......
169858043	......YLYPY......MPTLW......YLAID......VLHRD......IDFGF......RDDLE......
169845140	......KVYKT......VPFVR......AMVLD......FIHRD......IDFGL......RDDLE......
117616266	......RFYKQ......LPQVY......AMVLE......LIYRD......IDFGL......RDDLE......
118099670	......KIYKM......IPTIK......VMVME......FIHRD......IDFGL......RDDLE......
90078168	......KIYKM......IPTIR......VMVME......FIHRD......IDFGL......RDDLE......
121710678	......RVYKS......IPFVR......AMVID......FIHRD......IDFGL......RDDME......
121709972	......RTYKI......IPNVY......ILVID......LIYRD......VDFGM......RDDLE......
119467996	......RTYKI......IPNVY......ILVID......LIYRD......VDFGM......RDDLE......
119498129	......RVYKS......IPFVR......AMVID......FIHRD......IDFGL......RDDME......
119569254	......RFYKQ......IPQVY......AMVLE......LIYRD......IDFGL......RDDLE......
119580635	......KFYKM......IPSIK......VMVME......FIHRD......IDFGL......RDDLE......
119598081	......RFYKQ......LPQVY......AMVLE......LIYRD......IDFGL......RDDLE......
119589832	......RFYKQ......VPQVY......AMVLE......LIYRD......IDFGL......RDDLE......
119589837	......RFYKQ......VPQVY......AMVLE......LIYRD......IDFGL......RDDLE......
119589835	......RFYKQ......VPQVY......AMVLE......LIYRD......IDFGL......RDDLE......
155371843	......RFYKQ......VPQVY......AMVLE......LIYRD......IDFGL......RDDLE......
119602060	......RKLKY......VPKYW......FMIMD......YVHGD......VDYGL......RGDLE......
119620468	......KQLDY......IPLFY......FMVME......YVHGD......ADYGL......RSDVE......
148228468	......RFYKQ......VPQVY......AMVLE......LIYRD......IDFGL......RDDLE......
123365071	......TVIKR......FPRIL......YFVME......YIHRD......LDFGI......RDDLI......
123389731	......KLYLI......IPKFF......ILVME......FIHRD......IDFGL......RDDLE......
123393862	......RLYQI......VPRLH......TIVID......YIHRD......IDYGL......RDDME......
123409704	......RLYRY......FSSVK......VLVMD......FVHRD......IDFGL......RDDME......
123407554	......KLYTL......VPRMH......AMVID......FIHND......IDFGL......RDDME......
123419125	......KLYKK......IPNIH......IMVMD......YIHRD......IDFGL......RDDLE......
123414789	......TFMCG......FPHMI......YLVQE......FIHND......IDFGL......RDDMY......
123420570	......KLYTL......IPKVY......VLVME......YLHRD......IDFGI......RDDLE......
123414316	......TVMKL......FPKFY......FIIME......FVHRD......IDFGL......KDDII......
123432193	......ELTKQ......IVCVY......GFTME......VLHHD......IDFGL......REDLV......
123437140	......AFLEQ......FPKLL......YFVMD......FVHRD......IDFGL......RDDML......
123427521	......KCYKT......IPKIY......VLAME......LLHRD......IDFGV......RDDLE......
123436290	......RALKC......IPKVY......AFTME......FIHRD......IDFGL......RDDME......
123428390	......KLYML......VPKFY......YMVID......FVHRD......IDFGL......RDDME......
123428829	......RIYKL......FPNCY......CLAMD......FIHQD......IDFGL......RDDIE......
123446775	......KLYVL......VPRLY......IMVID......FIHND......IDFGL......RDDME......
123448046	......QYYKM......FPKIF......YIAME......LIHRD......IDFGL......RDDLI......
123438465	......RVYQY......FAVQH......ILVLE......FIHRD......ADFGL......KDDLE......
123437923	......QMYNI......IPHIY......YIVID......FIHRD......IDFGL......RDDLE......
123438716	......CIYKL......IPSIK......VMVME......LIHRD......IDMGL......RDDLE......
123451377	......KFYRL......FPEFK......VMAME......YIHRD......FDFGL......RDDLE......
123455056	......KIYSI......IPKVF......VLAME......ILHRD......IDFGL......RDDLE......
123474475	......KLYMY......MPKIR......VMVMD......FIHQD......IDYGL......RDDME......
123471961	......KDQKY......IPKFY......WLAME......YIHRD......IDYGL......RDDMF......
123469523	......EISKQ......FAIQY......YMVME......YIHRD......VDFGL......RDDLI......
123468305	......RIYNI......VPEIF......VLVMD......FIHRD......IDFGL......RDDLE......

```
123496124     ......KFLEK.....FPKII......YLAME.....FIHRD......IDYGI......RDDLY......
123499654     ......AIYRL.....IPNIK......ALVMD.....LIHRD......IDMGI......RDDLE......
123501767     ......KILTH.....FPRCG......FYSME.....FVHRD......IDFGL......KDDLI......
123501005     ......KLYSM.....IPVVY......YMAME.....FIHRD......IDFGL......RDDME......
123487063     ......KCYQA.....IPRVY......FLVME.....ILHRD......IDFGV......RDDLE......
123495479     ......ETLLK.....FPKIN......YLVMT.....FIHRD......IDFGL......RDDLI......
123479561     ......QVLKK.....IPLFK......YCAME.....YVHRD......IDFGI......RDDLI......
123503309     ......DIYQS.....IPQIY......VMVME.....YIHHD......IDFGL......RDDIE......
154412057     ......KCYSA.....IPKVY......FLVME.....ILHRD......IDFGV......RDDLE......
123503356     ......KMYGV.....VPKMY......LLAID.....FIHRD......IDFGL......RDDME......
123506850     ......KLYGV.....VPRLY......VMAID.....YIHRD......IDFGL......RDDME......
123480496     ......KIYKT.....IPDIK......VMVIE.....ILHRD......IDFGL......RDDLE......
123495566     ......KIYKL.....FPSMK......VMVMD.....LIHRD......IDFGL......RDDME......
123501642     ......NELEQ.....FPKLV......YYIME.....FIHRD......IDFGL......RDDML......
154418791     ......KLYKV.....IPNSI......VLVMD.....YIHRD......IDFGL......RDDME......
154420789     ......RIYQI.....IPRLF......VIVIE.....YIHRD......IDYGL......RDDIE......
154420843     ......RLYQI.....IPRVY......GMAMD.....FIHRD......IDLGL......RDDLE......
154421772     ......KLYTL.....ISRVY......AMIMD.....LIHRD......IDLGL......RDDLE......
121501708     ......KLYRI.....IPNVR......VLVMD.....FLHRD......IDFGL......RDDLE......
124007198     ......KFYKM.....IPSIK......VMVME.....FIHRD......IDFGL......RDDLE......
124361134     ......KLYRM.....IPNIK......VLVMD.....LLHRD......IDFGL......RDDLE......
124365563     ......QVYSN.....IPGVH......ILVMD.....FVHGD......IDLGL......RDDLE......
145475723     ......EILKQ.....TPKLI......LMVIQ.....VIHRD......VDFGI......KDDIE......
145478521     ......KLYQH.....IPQVY......IMVMD.....FLHRD......IDFGL......RDDLE......
145480019     ......QIIEE.....IPKLY......CMAMQ.....VVHRD......VDFGI......RDDLE......
145476561     ......EILKL.....IPELY......VMITN.....VIHRD......IDYGI......KDDLE......
145478815     ......SILNR.....TPKLY......VIVIQ.....VIHRD......VDYGV......KDDVE......
145476071     ......QIFKH.....IPKLI......VLVMQ.....VIHRD......LDFGI......KDDLE......
145479941     ......EILKT.....VPKVI......IMIQN.....IIHRD......IDFGI......KDDLE......
145477491     ......KLYTY.....IPRIF......YIVID.....FLHRD......LDFGL......RDDLE......
145476497     ......LILKQ.....FPELI......YYMST.....FIHRD......IDFGL......RDDLE......
145483743     ......QILQR.....IPKLY......IMVIQ.....IIHRD......VDFGI......GDDLE......
145486048     ......KLYNY.....IPRIY......YIVMD.....FLHRD......LDFGL......RDDLE......
145481345     ......KVLLE.....FPQIQ......YCMIT.....IIHRD......IDFGL......KDDLE......
145484675     ......TILNR.....VPKLY......VIVIQ.....VIHRD......VDYGV......KDDVE......
145483407     ......EILSR.....VPQML......VIVME.....VLHRD......IDFGI......KDDLE......
145483799     ......KVLQD.....FPQLK......FFMES.....LIHRD......IDFGL......RDDIE......
145483935     ......QILRS.....VPRLK......VMVIQ.....VIHRD......VDFGI......KDDLE......
145483297     ......QVLER.....VPKLF......VIILQ.....VVHRD......VDFGI......KDDIE......
145483439     ......QIIEE.....VPKLL......CMAMQ.....VIHRD......VDYGI......RDDLE......
145481767     ......KVLLE.....FPQIQ......YCMIT.....IIHRD......IDFGL......KDDLE......
145485382     ......EMLSK.....IPELV......IMITK.....IIHRD......IDFGI......KDDLE......
145483079     ......SSLSR.....IPQIY......VLVMQ.....ILHRD......VDFGI......KDDLE......
145489297     ......DILIE.....VPELI......IMITN.....IIHRD......IDFGI......KDDLE......
145487456     ......QILKK.....FPKYL......ILVIQ.....VIHRD......IDFGI......KDDLE......
145486363     ......KVYAF.....IPKVF......YLVME.....FIHRD......VDMGL......RDDLE......
145488231     ......QRLQE.....IPQLI......YIITP.....ILHLD......IDFGL......KDDLE......
145488478     ......KLYSY.....IPRIY......YIVID.....FLHRD......LDFGL......RDDLE......
145486547     ......KILQH.....IPRLY......LMVIS.....VLHRD......VDFGI......RDDLE......
145488147     ......KILQN.....IPSML......FLVMD.....YVHRD......IDFGL......RDDLE......
145493140     ......KILNV.....IPRLL......FLIMP.....ILHLD......IDFGL......KDDLE......
145494402     ......KVLIE.....FPQIR......YCIMQ.....FLHRD......IDFGL......RDDLE......
145490662     ......KLYQY.....IPNVY......IMVMD.....FLHRD......IGFGF......RDDLE......
145494181     ......KVYAF.....IPKIF......YLVME.....FIHRD......VDMGL......RDDLE......
145493796     ......QILYQ.....FTQLF......YFVMN.....VIHRD......IDFGL......RDDLE......
145491341     ......QIIKI.....FPKYY......ILVIQ.....VIHRD......IDFGI......KDDLE......
145490742     ......KLYNY.....IPRIY......YIVMD.....FLHRD......LDFGL......RDDLE......
145494041     ......KILQH.....IPRLF......LMVIS.....VLHRD......VDFGI......RDDLE......
145491572     ......KKLKE.....IPQLI......SYLIT.....ILHLD......IDFGL......KDDLE......
145491806     ......KILHL.....FTQLI......YFIMN.....IIHRD......IDFGL......RDDLE......
145497389     ......EILKQ.....TPKLI......IMVIQ.....VVHRD......VDFGI......KDDLE......
145498463     ......NTFQQ.....VPQPY......LIIMP.....YVHRD......IDFGL......RDDLE......
145496220     ......KLYQH.....IPQVY......IMVMD.....FLHRD......IDFGL......RDDLE......
145496945     ......KILVM.....FTQLI......YFIMN.....IIHRD......IDFGL......RDDLE......
145498230     ......QILQI.....FTQLF......YFVMN.....VIHRD......IDFGL......RDDLE......
145499514     ......KLYQY.....IPNVY......IMVMD.....FLHRD......IDFGL......RDDLE......
145498759     ......QIFKR.....VPQLL......VLVMQ.....VVHRD......IDFGI......KDDLE......
145496615     ......YLLTK.....ITKLY......VMVVE.....IVHRD......VDFGV......KDDLE......
145498074     ......NTLMQ.....IPQPY......LIIMP.....FVHRD......IDFGL......RDDLE......
145497118     ......KVLIE.....FPQIK......YCIMT.....FLHRD......IDFGL......KDDLE......
```

```
145498634   ......KIMME.....FPQIL.....YYIMN.....LIHRD.....IDFGL.....RDDLE.....
145502535   ......KVLQK.....IPKIK.....YFVLP.....ILHLD.....IDFGL.....KDDLE.....
145500756   ......AMLIQ.....IPKLI.....LMAIE.....IIHRD.....VDFGI.....KDDLE.....
145508263   ......QILRS.....VPRLK.....VMVIQ.....VIHRD.....VDFGI.....KDDLE.....
145508283   ......DIIRR.....VPQLI.....VLVLQ.....VLHRD.....IDFGI.....KDDLE.....
145506661   ......VLYKL.....FPRLY.....FIVQT.....YIHRD.....IDFGF.....ASDLE.....
145508459   ......QILQR.....IPKLH.....IMVIQ.....IVHRD.....VDFGI.....GDDLE.....
145507292   ......YLLTK.....ITKLY.....VMVIE.....IVHRD.....VDFGV.....KDDLE.....
145506122   ......QILKK.....FPKYF.....ILVIQ.....VIHRD.....IDFGI.....KDDLE.....
145508403   ......KVLQD.....FPQLK.....FFMES.....LIHRD.....IDFGL.....RDDIE.....
145506563   ......LILKQ.....FPQLI.....FYMST.....FIHRD.....IDFGL.....RDDLE.....
145505081   ......YLLTK.....ITKLY.....VMVIE.....IVHRD.....VDFGV.....KDDLE.....
145511762   ......KNLKI.....-----.....YFMQT.....LIHRD.....IDFGL.....RDDIE.....
145509449   ......QMLDE.....IPKII.....CLVMQ.....IIHRD.....ADFGI.....RDDLE.....
145512018   ......KLYQY.....IPKYY.....FLVME.....FLHRD.....VDYGL.....RDDLE.....
145513582   ......HILRL.....VPKLI.....ILVQQ.....VVHRD.....VDFGI.....KDDLE.....
145511081   ......RILNQ.....VPQVY.....YIVME.....IRHRD.....IDFGI.....IDDIE.....
145511950   ......EILRR.....IPKLY.....VLVIQ.....VLHRD.....VDFGI.....RDDME.....
145511508   ......EILKR.....IPKLH.....VLVIQ.....VLHRD.....VDFGI.....RDDLE.....
145516440   ......KLYHY.....IPKYY.....FLVME.....FIHRD.....VDYGL.....RDDLE.....
145515026   ......KKLPG.....IPKII.....CLVMQ.....IIHRD.....ADFGI.....KDDLE.....
145515219   ......QIMTL.....IPKVY.....FMSME.....VVHRD.....IDFGI.....LDDIE.....
145514942   ......KVLIE.....FPQIK.....YCIMT.....FLHRD.....IDFGL.....KDDLE.....
145517272   ......QILSA.....IPKVH.....VMVME.....YLYRD.....IDFGL.....RDDME.....
145513901   ......KVLIE.....FPQIK.....YCIMT.....FLHRD.....IDFGL.....KDDLE.....
145517017   ......EILRK.....IPKLY.....YLVIQ.....VIHRD.....VDFGI.....RDDME.....
145521152   ......EILTL.....IPELY.....VMITR.....IIHRD.....IDFGI.....KDDLE.....
145521025   ......TILKQ.....IPNVY.....VIILN.....IIHRD.....IDFGI.....KDDLE.....
145520507   ......DIIQK.....VPKLL.....VLVLQ.....VLHRD.....IDYGI.....KDDLE.....
145518846   ......QILSA.....IPKVH.....VMVME.....YIYRD.....IDFGL.....RDDME.....
145521620   ......KLYQY.....IPNVY.....IMVMD.....FLHRD.....IDFGL.....RDDLE.....
145519289   ......KIYKV.....IPQSY.....ILVME.....LIHRD.....IDFGL.....RDDLE.....
145520325   ......KILKS.....VPKFH.....IIILE.....IIHRD.....IDYGI.....KDDIE.....
145521027   ......QIIEE.....VPKLY.....CMAMQ.....VIHRD.....VDFGI.....KDDLE.....
145520493   ......QILKN.....VPKLK.....VMVIQ.....VIHRD.....VDYGI.....KDDLE.....
145518772   ......TMLIQ.....IPKLI.....LMALQ.....IIHRD.....VDFGV.....KDDLE.....
145518157   ......VLYKL.....FPRLY.....FIVQT.....YIHRD.....IDFGF.....ASDLE.....
145524737   ......KLYHY.....IPKYY.....FLVME.....FIHRD.....VDYGL.....RDDLE.....
145522458   ......QILKI.....FPKFY.....ILVIQ.....VIHRD.....IDFGI.....KDDLE.....
145526452   ......EILKR.....IPKLH.....VLVIQ.....VLHRD.....VDFGI.....RDDLE.....
145526012   ......EILKR.....IPKLY.....YLVIQ.....VIHRD.....VDFGI.....RDDME.....
145527196   ......EILKQ.....TPKLI.....IMVIQ.....VVHRD.....VDFGI.....KDDLE.....
145528103   ......KIYKV.....IPQSY.....ILVME.....LIHRD.....IDFGL.....RDDLE.....
145525541   ......KIMKK.....IPKVY.....YLSME.....IVHRD.....IDFGI.....RDDIE.....
145525968   ......KLYQY.....IPKYY.....FLVME.....FLHRD.....VDYGL.....RDDLE.....
145525224   ......LILKQ.....FPDLI.....YYMST.....FIHRD.....IDFGL.....RDDLE.....
145525136   ......VLYKL.....FPRLY.....FIVQT.....YIHRD.....IDFGF.....ASDLE.....
145530992   ......SILNR.....TPKLY.....VIVIQ.....VIHRD.....VDYGV.....KDDVE.....
145529654   ......QIIEE.....VPKLY.....CMAMQ.....VIHRD.....VDFGI.....RDDLE.....
145529626   ......EILSR.....IPKLL.....TIVME.....VLHRD.....IDFGI.....KDDIE.....
145529902   ......KLYNY.....IPKIH.....YIVMD.....FLHRD.....VDFGL.....RDDLE.....
145533915   ......QVLQR.....VPNLY.....IIILQ.....VVHRD.....VDFGI.....KDDLE.....
145533605   ......HILRL.....VPKLI.....ILVQQ.....VVHRD.....VDYGI.....KDDLE.....
145534895   ......KLYQY.....IPKYY.....FLVME.....FLHRD.....VDYGL.....RDDLE.....
145536139   ......KVLSE.....FPQIK.....FFMET.....LIHRD.....IDFGL.....RDDIE.....
145536365   ......KVTKA.....IPALH.....VMVID.....FIHRD.....LDFGL.....RDDLE.....
145537512   ......QVLER.....VPKIY.....VIVMQ.....VVHRD.....VDFGI.....KDDIE.....
145537552   ......EIYKR.....IPHVY.....FMVMD.....IIHRD.....LDFGL.....RDDIE.....
145536323   ......KILLE.....FPQIL.....FFMIT.....LIHRD.....IDFGL.....RDDLE.....
145536241   ......EVLKL.....VPKIY.....IMIQN.....IIHRD.....IDFGI.....KDDLE.....
145535664   ......HILTR.....VPKLI.....VLVQQ.....VVHRD.....VDFGI.....KDDLE.....
145534949   ......EILKR.....IPKLY.....VLVIQ.....VLHRD.....VDFGI.....RDDME.....
145535377   ......EILRR.....IPKLY.....VLVIQ.....VLHRD.....VDFGI.....KDDLE.....
145535137   ......KVLNI.....FPQIV.....YFMQT.....LIHRD.....IDFGL.....KDDIE.....
145541949   ......KVLIE.....FPQIK.....YCIMT.....FLHRD.....IDFGL.....KDDLE.....
145541912   ......KVTKA.....IPTLH.....VMIID.....FIHRD.....LDFGL.....RDDLE.....
145542237   ......RVNDN.....FPQIV.....YFMQT.....LIHRD.....IDFGL.....KDDIE.....
```

```
145541712   ......KVLSE.....FPQIK.....FFMET.....LIHRD.....IDFGL.....RDDIE.....
145541008   ......LVLER.....VPKMF.....VIVMQ.....VVHRD.....IDFGI.....KDDIE.....
145541872   ......KILLV.....FPQIL.....FFMIT.....LIHRD.....IDFGL.....RDDLE.....
145541552   ......SRLKQ.....VPQLI.....YLITP.....ILHLD.....IDFGL.....KDDLE.....
145539926   ......KIYRI.....IPNLH.....VLVMD.....FIHRD.....IDFGL.....RDDLE.....
145540164   ......QIIEE.....IPKLY.....CMAMQ.....VIHRD.....VDFGI.....RDDLE.....
145540060   ......EILKL.....IPRVI.....IMIQN.....IIHRD.....IDFGI.....KDDLE.....
145546991   ......EILKK.....IPRLY.....VLVET.....YIHRD.....IDFGL.....ASDLE.....
145548142   ......KILQI.....FTQLI.....YFIMN.....IIHRD.....IDFGL.....RDDLE.....
145545440   ......KIMME.....FPQIL.....YYIMN.....LIHRD.....IDFGL.....RDDLE.....
145546478   ......QILKK.....FPKYF.....ILVIQ.....VIHRD.....IDFGI.....KDDLE.....
145544939   ......YLLTK.....VTKLF.....VMVIE.....IVHRD.....VDFGV.....KDDLE.....
145548662   ......EMLGK.....IPELL.....IMVMK.....IIHRD.....IDFGI.....KDDLE.....
145549091   ......QALSR.....IPQIY.....ILVMQ.....ILHRD.....VDFGI.....KDDLE.....
145552992   ......QIIEE.....VPKLY.....CMAMQ.....VIHRD.....VDYGI.....RDDLE.....
145549327   ......QVLER.....VPKLL.....VIILQ.....VVHRD.....VDFGI.....KDDIE.....
145553020   ......EILSR.....VPQIF.....VIVME.....VLHRD.....IDFGI.....KDDLE.....
145548333   ......QILRN.....VPKLK.....VMVIQ.....IIHRD.....VDYGI.....KDDLE.....
145550981   ......KILKK.....VPQLL.....VMITK.....ILHRD.....IDYGI.....RDDLE.....
145473577   ......KVLID.....FPEIK.....FCVMN.....YLHRD.....IDFGL.....RDDLQ.....
145550626   ......NFEEI.....GTLIT.....VMITK.....IIHRD.....IDFGI.....RDDLE.....
125527438   ......KIYRI.....IPNVR.....VLVMD.....FLHRD.....IDFGL.....RDDLE.....
125525033   ......KLYNA.....IANVK.....VLVID.....YLHRD.....IDFGL.....RDDLE.....
125527876   ......QVYSA.....IPWVH.....VLVMD.....FVHGD.....IDLGL.....RDDLE.....
125527674   ......RIYSA.....VPRVH.....VMVMD.....YVHGD.....VDLGL.....RDDLE.....
125524785   ......QVYNT.....VPRVH.....IMVMD.....YVHGD.....VDLGL.....RDDLE.....
125526493   ......KLYRI.....IPNVK.....VLVMD.....FLHRD.....IDFGL.....RDDLE.....
125532354   ......KVYMQ.....IPHMK.....VMVID.....FIHRD.....IDYGL.....RDDLE.....
125540342   ......KLYML.....IPHLK.....VMVID.....FLHRD.....IDYGL.....RDDLE.....
125546013   ......QVYHT.....IPSVH.....ILVMD.....FVHGD.....IDLGL.....RDDLE.....
125551174   ......QVYNT.....VPRVH.....IMIMD.....YVHGD.....VDLGL.....RDDLE.....
125557386   ......QVYHN.....IPAVH.....ILVMD.....FVHGD.....IDLGL.....RDDLE.....
125570876   ......KLYRI.....IPNVK.....VLVMD.....FLHRD.....IDFGL.....RDDLE.....
125569628   ......KLYNA.....IANVK.....VLVID.....YLHRD.....IDFGL.....RDDLE.....
125569389   ......QVYNT.....VPRVH.....IMVMD.....YVHGD.....VDLGL.....RDDLE.....
125571761   ......KIYRI.....IPNVR.....VLVMD.....FLHRD.....IDFGL.....RDDLE.....
125571990   ......-----.....-----.....WQVMD.....YVHGD.....VDLGL.....RDDLE.....
125575129   ......KVYMQ.....IPHMK.....VMVID.....FIHRD.....IDYGL.....RDDLE.....
125572187   ......QVYSA.....IPWVH.....VLVMD.....FVHGD.....IDLGL.....RDDLE.....
125582924   ......KLYML.....IPHLK.....VMVID.....FLHRD.....IDYGL.....RDDLE.....
125584099   ......KLYML.....IPHLK.....VMVID.....FLHRD.....IDYGL.....RDDLE.....
125803368   ......KFYMR.....VPKYW.....FMVMD.....YVHAD.....VDYGL.....RADLE.....
126644363   ......KVLKL.....IANVY.....IMAME.....FIHRD.....IDFGL.....RDDLE.....
126644763   ......KLYKI.....VPTVH.....VMILD.....FIHRD.....IDFGL.....RDDLE.....
126277048   ......RFYKQ.....LPQVY.....AMVLE.....LIYRD.....IDFGL.....RDDLE.....
126277054   ......RFYKQ.....LPQVY.....AMVLE.....LIYRD.....IDFGL.....RDDLE.....
126277045   ......RFYKQ.....LPQVY.....AMVLE.....LIYRD.....IDFGL.....RDDLE.....
126277051   ......RFYKQ.....LPQVY.....AMVLE.....LIYRD.....IDFGL.....RDDLE.....
126290539   ......HKLKY.....IPKYW.....FMVMD.....YVHGD.....VDYGL.....RGDLE.....
126290560   ......KLYKI.....IPHIR.....VLVMD.....FIHRD.....IDFGL.....RDDME.....
126304412   ......KAFKQ.....IPRFW.....FMVME.....YVHGD.....ADYGL.....RGDLE.....
126308856   ......KIYKM.....IPTIK.....VMVME.....FIHRD.....IDFGL.....RDDLE.....
126323502   ......RFYKQ.....IPQVY.....AMVLE.....LIYRD.....IDFGL.....RDDLE.....
126323500   ......RFYKQ.....IPQVY.....AMVLE.....LIYRD.....IDFGL.....RDDLE.....
126330884   ......KLYKI.....IAHMR.....VLVMD.....FIHRD.....IDFGL.....GDDME.....
126337648   ......RLYKA.....IPHMR.....VLVMD.....FIHRD.....IDFGL.....GDDLE.....
126342667   ......KLYKV.....IPHMR.....VLVMD.....FIHRD.....IDFGL.....RDDME.....
126342685   ......RLYKL.....IPRTR.....ILVMD.....FIHRD.....IDFGL.....RDDVE.....
154331976   ......RVMQD.....IATLK.....MLIMS.....YVHRD.....IDFGL.....RDDLE.....
154344539   ......RFYRI.....IPMMF.....VMVIE.....VLHRD.....IDFGL.....RDDME.....
154339044   ......LNYSG.....IPQIY.....IMVME.....FVHRD.....IDYGL.....RDDLE.....
154344537   ......RCYSI.....MPTIF.....LLTME.....ILHRD.....IDFGL.....RDDIE.....
154342049   ......RIYKK.....IPKSY.....VMVMD.....YLHRD.....IDVGL.....RDDLE.....
154339974   ......KVLQE.....IAKLK.....VLIMS.....YVHAD.....VDFGL.....RDDIE.....
146094192   ......RIYKK.....IPKSY.....VMVMD.....YLHRD.....IDVGL.....RDDLE.....
146100666   ......RYYTL.....MPTIF.....VMAME.....ILHRD.....IDFGL.....RDDME.....
146100670   ......RFYRI.....IPMMF.....VMVIE.....VLHRD.....IDFGL.....RDDME.....
146076579   ......RVMQD.....IATLK.....MLIMS.....YVHRD.....IDFGL.....RDDLE.....
146091004   ......KVMML.....IAKLK.....VLIMS.....YVHAD.....VDFGL.....RDDIE.....
146088972   ......SNHSV.....IPQIY.....IMVME.....FVHRD.....IDYGL.....RDDLE.....
```

145238078	......RVYKS......IPFVR......AMVID......FIHRD......IDFGL......RDDME......
145243206	......VFYAK......MLKLH......VMGID......IIHRD......VDMGL......RDDME......
145255705	......RTYKI......IPNVY......ILVID......LIYRD......VDFGM......RDDLE......
145342020	......RLYKI......VPNVR......IMVMD......FIHRD......IDFGL......RDDLE......
145343446	......SVYNS......VPKVH......VMVMD......FVHGD......VDLGL......RDDLE......
145609976	......RVYKS......IPFVR......AMVLD......FIHRD......IDFGL......RDDME......
145612111	......RTYKI......IPNVY......ILVID......LIYRD......VDFGM......RDDLE......
146186258	......KIFKV......IPTLY......VMVID......FIHRD......IDYGL......RDDME......
146184983	......KILYD......FTRMY......IMVTT......YVHRD......IDFGL......RDDLE......
146183776	......KIMKA......IPKLY......VMVLD......YIHRD......IDYGL......RDDLE......
146182625	......YIMKQ......FPRLI......FIIMS......FLHRD......IDFGL......RDDLE......
146165828	......KLYQY......IPNVY......IMVMD......FLHRD......IDFGL......RDDLE......
146161387	......QILTR......VPKLY......IMVLN......IIHRD......IDYGI......KDDLE......
146420773	......RTYKH......VPNAY......ILVID......LIYRD......IDFGM......RDDLE......
146419855	......RSYRI......IPQAY......ILVID......LIYRD......VDFGM......RDDLE......
146412077	......KVYKA......IPFVR......AMVID......FIHRD......IDFGL......RDDLE......
149240846	......RTYKH......IPNAY......ILVID......LIYRD......IDFGM......RDDLE......
149240353	......RSYRI......IPQAY......ILIID......LIYRD......VDFGM......RDDLE......
149236319	......KVYKA......IPFVR......AMVID......FIHRD......IDFGL......RDDLE......
147766588	......QVYNT......VPRVH......VMVMD......YVHGD......VDLGL......RDDLE......
147802057	......QVYNA......VPRVH......VMVMD......YVHGD......VDLGL......RDDLE......
147807924	......QVYNT......VPRVH......VMVMD......YVHGD......VDLGL......RDDLE......
147805658	......KLYML......IPHLK......VMVID......FFTVD......IDFGL......RDDLE......
147816350	......QVYNT......IPWVH......ILVMD......FVHGD......IDLGL......RDDLE......
147859066	......KLYRI......IPNVR......VLVMD......FLHRD......IDFGL......RDDLE......
149270087	......RFYKQ......IPQVY......AMVLE......LIYRD......IDFGL......RDDLE......
148672704	......KFYKM......IPSIK......VMVME......FIHRD......IDFGL......RDDLE......
148677821	......KLYKI......IPHIR......VLVMD......FIHRD......IDFGL......RDDME......
148677820	......KLYKI......IPHIR......VLVMD......FIHRD......IDFGL......RDDME......
149270093	......RFYKQ......IPQVY......AMVLE......LIYRD......IDFGL......RDDLE......
148677819	......KLYKI......IPHIR......VLVMD......FIHRD......IDFGL......RDDME......
148684317	......KFYRK......IPVFY......FMVME......YVHGD......-DF--......RSDVE......
148686795	......HKLKY......VPKYW......FMIMD......YVHGD......VDYGL......RGDLE......
148686794	......HKLKY......VPKYW......FMIMD......YVHGD......VDYGL......RGDLE......
148686796	......HKLKY......VPKYW......FMIMD......YVHGD......VDYGL......RGDLE......
148694162	......RFYKQ......LPQVY......AMVLE......LIYRD......IDFGL......RDDLE......
156098931	......KLYKI......VPKVY......IMVLD......FIHRD......IDFGL......RDDIE......
148913016	......VFYNN......IPVFY......FIIIN......YTHSD......LDYGL......RGDLE......
149034516	......RFYKQ......VPQVY......AMVLE......LIYRD......IDFGL......RDDLE......
149044209	......HKLKY......VPKYW......FMIMD......YVHGD......VDYGL......RGDLE......
149041999	......RFYKQ......LPQVY......AMVLE......LIYRD......IDFGL......RDDLE......
149044208	......HKLKY......VPKYW......FMIMD......YVHGD......VDYGL......RGDLE......
149064402	......KLYKI......IPHIR......VLVMD......FIHRD......IDFGL......RDDME......
149065931	......KFYKM......IPSIK......VMVME......FIHRD......IDFGL......RDDLE......
149065932	......KFYKM......IPSIK......VMVME......FIHRD......IDFGL......RDDLE......
149262023	......KFYRK......IPVFY......FMVME......YVHGD......ADYFI......RSDVE......
149270095	......RFYKQ......IPQVY......AMVLE......LIYRD......IDFGL......RDDLE......
149270097	......RFYKQ......IPQVY......AMVLE......LIYRD......IDFGL......RDDLE......
150865330	......RSYRI......IPQAY......ILIID......LIYRD......VDFGM......RDDLE......
150864368	......RTYKH......IPNAY......ILVID......LIYRD......IDFGM......RDDLE......
150865254	......KVYKA......IPFVR......AMVID......FIHRD......IDFGL......RDDLE......
149632217	......RFYKQ......LPQVY......AMVLE......LIYRD......IDFGL......RDDLE......
149636916	......THSHK......VPKYW......FMVMD......YVHGD......VDYGL......RGDLE......
149691878	......RFYKQ......LPQVY......AMVLE......LIYRD......IDFGL......RDDLE......
149691882	......RFYKQ......LPQVY......AMVLE......LIYRD......IDFGL......RDDLE......
149691880	......RFYKQ......LPQVY......AMVLE......LIYRD......IDFGL......RDDLE......
149726422	......RFYKQ......IPQVY......AMVLE......LIYRD......IDFGL......RDDLE......
149726418	......RFYKQ......IPQVY......AMVLE......LIYRD......IDFGL......RDDLE......
149726416	......RFYKQ......IPQVY......AMVLE......LIYRD......IDFGL......RDDLE......
149726420	......RFYKQ......IPQVY......AMVLE......LIYRD......IDFGL......RDDLE......
149727556	......KTLKQ......IPLFY......FMVME......YVHGD......ADYGL......RSDLE......
149737654	......HKLKY......VPKYW......FMIMD......YVHGD......VDYGL......RGDLE......
149743042	......KFYKM......IPSIK......VMVME......FIHRD......IDFGL......RDDLE......
149758772	......KIYKM......IPTIR......VMVME......FIHRD......IDFGL......RDDLE......
154278146	......RVYKS......IPFVR......AMVID......FIHRD......IDFGL......RDDME......
157128234	......AVLKK......ICRFI......YVVMQ......FLHRD......LDFGL......QDDLW......
109071262	......AVLKK......VCRFI......YVVMQ......FLHRD......LDFGL......HDDLW......
109485777	......AVLKK......VCRFI......YVVMQ......FLHRD......LDFGL......HDDLW......
118403710	......AVLKK......VCRFI......YVVMQ......FLHRD......LDFGL......HDDLW......
114656574	......AVLKK......VCRFI......YVVMQ......FLHRD......LDFGL......HDDLW......
114656576	......AVLKK......VCRFI......YVVMQ......FLHRD......LDFGL......HDDLW......
115675630	......AVLKK......ICKFI......YVVMT......FLHRD......LDFGL......HDDLW......

```
116056000          ......RVMRA.....FPELL.....FLAME.....YVHRD.....ID-GL.....RDDLY.....
116812624          ......AVLKK.....VCRFI.....YVVMQ.....FLHRD.....LDFGL.....HDDLW.....
133930875          ......TVMRL.....IPKCV.....FFVMT.....FIHRD.....IDFGL.....KDDLW.....
118087763          ......AVLKK.....VCRFI.....YVVMQ.....FLHRD.....LDFGL.....HDDLW.....
119624574          ......AVLKK.....VCRFI.....YVVMQ.....FLHRD.....LDFGL.....HDDLW.....
119624573          ......AVLKK.....VCRFI.....YVVMQ.....FLHRD.....LDFGL.....HDDLW.....
119624572          ......AVLKK.....VCRFI.....YVVMQ.....FLHRD.....LDFGL.....HDDLW.....
123389204          ......NILKS.....FATVI.....YFVMP.....FVHCD.....IDFGL.....RDDVI.....
123420975          ......LINQD.....FTKFY.....YMVIE.....YVHRD.....IDFGL.....RDDLW.....
123426769          ......QILKK.....FPVFH.....WLVLE.....LVHRD.....IDFGL.....CDDLF.....
123431838          ......YIMRK.....FPKYI.....FLVME.....IIHRD.....IDFGL.....RDDLI.....
123446561          ......KILRR.....FPKLF.....YVVME.....FIHRD.....LDFGL.....RDDMI.....
123447884          ......QVLDK.....FPRVG.....FFSME.....YVHRD.....IDFGL.....RDDVI.....
123448484          ......KLYPT.....IPRFI.....YLIIE.....VIHRD.....IDFGL.....CDDLF.....
123455026          ......VIQKK.....RKRFL.....NVHIS.....IVHGD.....IDFGL.....CDDIY.....
123976122          ......TFIKS.....FPKIY.....YAVMD.....YIHRD.....IDFGL.....ADDLA.....
123478066          ......SILSR.....FPRIK.....YFIME.....FIHRD.....IDFGL.....KDDLI.....
123472485          ......KVLRK.....YPTVL.....WFVMD.....YIHRD.....IDFGF.....CDDLM.....
123470167          ......SILQE.....FPQII.....YLVME.....YVHRD.....IDFGL.....RDDII.....
123476607          ......SVLKR.....FPKFR.....WLVME.....FIHRD.....IDFGL.....RDDLI.....
123478316          ......YFLEK.....FPKLI.....YCIME.....FVHRD.....IDFGL.....RDDLM.....
123501275          ......DVMEE.....CFPLV.....YIIME.....FVHRD.....IDFGL.....CDDLI.....
123480951          ......NLMRR.....LFHFQ.....FLVMG.....FIHRD.....IDYGL.....KDDLI.....
123498930          ......KVLER.....FPQIR.....WLTLE.....FIHRD.....IDFGL.....RDDLI.....
154422448          ......QIIAQ.....FPRLG.....FFSME.....IVHRD.....IDFGL.....RDDLI.....
154415338          ......QIIAQ.....FPRLG.....FFSME.....IVHRD.....IDFGL.....RDDLI.....
123238561          ......AVLKK.....VCRFI.....YVVMQ.....FLHRD.....LDFGL.....HDDLW.....
123858224          ......AVLKK.....VCRFI.....YVVMQ.....FLHRD.....LDFGL.....HDDLW.....
125821965          ......AVLKK.....VCRFV.....YVVME.....FLHRD.....LDFGL.....HDDLW.....
125829661          ......AVLKK.....VCKFI.....YVVMQ.....FLHRD.....LDFGL.....HDDLW.....
125841748          ......AVLKK.....VCKFI.....YVVMQ.....FLHRD.....LDFGL.....HDDLW.....
125841806          ......AVLKK.....VCKFI.....YVVMQ.....FLHRD.....LDFGL.....HDDLW.....
125846368          ......AVLKR.....VCRFV.....YVVME.....FLHRD.....LDFGL.....HDDLW.....
126253819          ......AVLKK.....VCRFI.....YVVMQ.....FLHRD.....LDFGL.....HDDLW.....
154336611          ......LALKK.....VVRYL.....FLVME.....LVHRD.....IDFGL.....VDDLW.....
146099450          ......LALKK.....VVRYL.....YLVME.....LVHRD.....IDFGL.....VDDLW.....
145355968          ......RVMRA.....FAELL.....FLAME.....YVHRD.....IDFGL.....RDDLF.....
148472907          ......DVLEA.....SAHII.....FTVMT.....FIHRD.....LDFGE.....VDDLW.....
148696073          ......AVLKK.....VCRFI.....YVVMQ.....FLHRD.....LDFGL.....HDDLW.....
148696074          ......AVLKK.....VCRFI.....YVVMQ.....FLHRD.....LDFGL.....HDDLW.....
148691560          ......AVLKK.....VCRFI.....YVVMQ.....FLHRD.....LDFGL.....HDDLW.....
149069383          ......AVLKK.....VCRFI.....YVVMQ.....FLHRD.....LDFGL.....HDDLW.....
149268725          ......AVLKK.....VCRFI.....YVVMQ.....FLHRD.....LDFGL.....HDDLW.....
149269324          ......AVLKK.....VCRFI.....YVVMQ.....FLHRD.....LDFGL.....HDDLW.....
149692027          ......AVLKK.....VCRFI.....YVVMQ.....FLHRD.....LDFGL.....HDDLW.....
149732552          ......AVLKK.....VCRFI.....YVVMQ.....FLHRD.....LDFGL.....HDDLW.....
157132820          ......KK-NL.....CLPFQ.....LIIRE.....ICHGD.....SDFAS.....EMDIF.....
110749853          ......RS-KL.....CLPFQ.....SIMRE.....VCHGD.....TDFAS.....MMDIF.....
111306263          ......KIR-L.....CLPFQ.....LLFRQ.....VRHGD.....TDFAS.....AMDIF.....
115397269          ......ERKVL.....ALSYQ.....YLVRQ.....LFHGD.....TDFAS.....AMDIF.....
114589145          ......KIR-L.....CLPFQ.....MLFRQ.....VRHGD.....TDFAS.....AMDIF.....
116056769          ......RDALT.....CWPFT.....YLMRQ.....VVHGD.....CDFAS.....AADIY.....
169862094          ......KE-SL.....VYNYQ.....YIIRQ.....VSHGD.....TDFAS.....AMDCF.....
117558519          ......KIR-L.....CLPFQ.....MLFRQ.....VRHGD.....TDFAS.....AMDIF.....
118085976          ......KIRLH.....CLPFQ.....MLFRQ.....VRHGD.....TDFAS.....AMDIF.....
121699902          ......RKL-L.....ALSHQ.....YLVRQ.....VFHGD.....TDFSS.....AMDIF.....
119471870          ......KLLS-.....ALSHQ.....YLVRQ.....VFHGD.....TDFSS.....AMDIF.....
154152111          ......KIRLH.....CLPFQ.....MLFRQ.....VRHGD.....TDFAS.....AMDIF.....
123455842          ......SL---.....IVKYT.....FLIRP.....LYHSD.....TDLAP.....AADLF.....
125541468          ......RNFEG.....VWPFQ.....YLLRQ.....ICHGD.....ADFAS.....SMDIF.....
125584011          ......RNFEG.....VWPFQ.....YLLRQ.....ICHGD.....ADFAS.....SMDIF.....
145240449          ......KLLS-.....ALSYQ.....YLVRQ.....VFHGD.....SDFSS.....AMDIF.....
145345011          ......REALT.....CWPFT.....YLMRQ.....VEHGD.....SDYAS.....SADIF.....
145603362          ......KPKAL.....ALAYQ.....YLVRQ.....IYHGD.....SDFSS.....AMDIF.....
146416897          ......EASLL.....VLPWH.....YLIRQ.....LHHGD.....SDFSN.....AMDLF.....
149236053          ......QSSLL.....TLTWH.....YLIRQ.....IHHGD.....TDFAG.....EMDLF.....
148689162          ......KIRLH.....CLPFQ.....MLFRQ.....VRHGD.....TDFAS.....AMDIF.....
157817604          ......KIRLH.....CLPFQ.....MLFRQ.....VRHGD.....TDFAS.....AMDIF.....
150865736          ......TELLV.....VLPWH.....YIIRQ.....VRHGD.....ADFAE.....EMDLF.....
```

149632003	......KIRLH.....CLPFQ.....MLFRQ.....VRHGD.....TDFAS.....AMDIF.....
149632001	......KIRLH.....CLPFQ.....MLFRQ.....VRHGD.....TDFAS.....AMDIF.....
149729718	......KIRLH.....CLPFQ.....MLFRQ.....VRHGD.....TDFAS.....AMDIF.....
154309079	......KA--L.....ALPYQ.....YLVRQ.....VFHGD.....SDFSS.....AMDVF.....
157137399	......GILKQ.....IVGYR.....YIAME.....ILHGD.....CDFGV.....KADIF.....
109085988	......KILKS.....IVGYR.....CLAME.....LLHGD.....CDVGV.....KADIF.....
121583770	......KILKN.....IVGYR.....CLAME.....LLHGD.....CDVGV.....KADMF.....
110755185	......EILRK.....IIGFR.....CLAME.....YLHGD.....CDFGV.....KADIW.....
114619467	......KILKS.....IVGYR.....CLAME.....LLHGD.....CDVGV.....KADIF.....
126303471	......IPEKN.....FIGYR.....CLAME.....LLHGD.....CDVGV.....KADIF.....
126335599	......KILKD.....IVGYQ.....CRAME.....LLHGD.....CDVGV.....KADIF.....
148704068	......KILKN.....IIGYR.....CLAME.....LLHGD.....CDVGV.....KADVF.....
149633075	......KILKN.....IVGYR.....CLAME.....LLHGD.....CDVGV.....KADIF.....
149746463	......KILKS.....IVGYR.....CLAME.....LLHGD.....CDVGV.....KADIF.....
156121003	......KILKT.....IIGYR.....CLAME.....LLHGD.....CDVGV.....KADIF.....
157127321	......AVLKK.....VVKLV.....YLVFE.....IIHGD.....ADLGV.....AADIW.....
109099013	......AILKK.....VVKLV.....YMVFE.....IIHRD.....ADFGV.....ALDVW.....
109099023	......AILKK.....VVKLV.....YMVFE.....IIHRD.....ADFGV.....ALDVW.....
109099009	......AILKK.....VVKLV.....YMVFE.....IIHRD.....ADFGV.....ALDVW.....
109099031	......AILKK.....VVKLV.....YMVFE.....IIHRD.....ADFGV.....ALDVW.....
109099007	......AILKK.....VVKLV.....YMVFE.....IIHRD.....ADFGV.....ALDVW.....
109099027	......AILKK.....VVKLV.....YMVFE.....IIHRD.....ADFGV.....ALDVW.....
109099017	......AILKK.....VVKLV.....YMVFE.....IIHRD.....ADFGV.....ALDVW.....
109112805	......AILKK.....VVKLI.....YLVFD.....IVHRD.....ADFGV.....ALDVW.....
109112801	......AILKK.....VVKLI.....YLVFD.....IVHRD.....ADFGV.....ALDVW.....
115495061	......AILKK.....VVKLV.....YMVFE.....IIHRD.....ADFGV.....ALDVW.....
115389198	......AILKK.....VVSLL.....YIVLE.....IIHRD.....SDFGV.....AIDVW.....
115709857	......AILKK.....IVKLF.....YMVFE.....VIHRD.....ADFGV.....ALDVW.....
169864143	......AIMKK.....VVRLY.....YMAME.....IIHRD.....GDFGV.....SIDIW.....
169847052	......AVMKQ.....LAMLY.....YLIME.....ILHRD.....IDFGV.....AVDIW.....
117616258	......AILKK.....VVKLV.....YMVFE.....IIHRD.....ADFGV.....ALDVW.....
118098481	......AILKK.....VVKLV.....YMVFE.....IIHRD.....ADFGV.....ALDVW.....
118100065	......AILKK.....VVKLI.....YMVFD.....IIHRD.....ADFGV.....ALDVW.....
90082535	......AILKK.....VVKLV.....YMVFE.....IIHRD.....ADFGV.....ALDVW.....
121702405	......AILKK.....VVSLL.....YIVLE.....IIHRD.....SDFGV.....AIDVW.....
121712642	......AIMKK.....LVSLI.....YMVME.....IVHRD.....VDFGV.....AADIW.....
119498561	......AIMKK.....LVSLI.....YMVME.....IVHRD.....VDFGV.....AADIW.....
119496399	......AILKK.....VVSLL.....YIVLE.....IIHRD.....SDFGV.....AIDVW.....
119610882	......AILKK.....VVKLI.....YLVFD.....IVHRD.....ADFGV.....ALDVW.....
119618653	......AILKK.....VVKLV.....YMVFE.....IIHRD.....ADFGV.....ALDVW.....
119610880	......AILKK.....VVKLI.....YLVFD.....IVHRD.....ADFGV.....ALDVW.....
119911712	......AILKK.....VVKLI.....YLVFD.....IIHRD.....ADFGV.....ALDVW.....
123231748	......AILKK.....IVKLV.....HMVFE.....IVHRD.....ADFGV.....ALDVW.....
125837338	......AILKK.....VVKLV.....HMVFE.....IIHRD.....ADFGV.....ALDIW.....
126275077	......AIMKK.....IVQLK.....YLVLE.....IVHRD.....SDFGV.....KIDIW.....
126343449	......AILKK.....IVKLI.....YMVFD.....IVHRD.....ADFGV.....ALDVW.....
145253805	......AIMKK.....LVSLI.....YMVME.....IVHRD.....VDFGV.....ATDIW.....
145255686	......AILKK.....VVSLL.....YIVLE.....IIHRD.....SDFGV.....AIDVW.....
145605601	......AILKK.....VVALL.....YMVLE.....VVHRD.....SDFGV.....QIDVW.....
146416343	......QIMKE.....VVKLF.....LLVLE.....IIHRD.....SDFGV.....RIDIW.....
146413785	......AIMKR.....IVSLR.....YLVLE.....IVHRD.....SDFGV.....KIDIW.....
149237114	......AIMKK.....IVALK.....YMVLE.....VVHRD.....SDFGV.....RIDIW.....
148687718	......AILKK.....VVKLV.....YMV--.....IIHRD.....ADFGV.....ALDVW.....
148687716	......AILKK.....VVKLV.....YMEIR.....IIHRD.....ADFGV.....ALDVW.....
148687717	......AILKK.....VVKLV.....YMV--.....IIHRD.....ADFGV.....ALDVW.....
148687719	......AILKK.....VVKLV.....YMEIR.....IIHRD.....ADFGV.....ALDVW.....
148806859	......AILKK.....VVKLI.....YLVFD.....IVHRD.....ADFGV.....ALDVW.....
149053315	......AILKK.....VVKLI.....YLVFD.....IVHRD.....ADFGV.....ALDVW.....
149063344	......AILKK.....VVKLV.....YMV--.....IIHRD.....ADFGV.....ALDVW.....
149063343	......AILKK.....VVKLV.....YMV--.....IIHRD.....ADFGV.....ALDVW.....
149063342	......AILKK.....VVKLV.....YMVFE.....IIHRD.....ADFGV.....ALDVW.....
149389000	......AIMKK.....IVQLK.....YLVLE.....IVHRD.....SDFGV.....KIDIW.....
150863844	......EIMKE.....VVKLY.....LLILE.....IIHRD.....SDFGV.....RIDIW.....
149480039	......AILKK.....IVKLI.....YLVFD.....IIHRD.....ADFGV.....ALDVW.....
149724188	......AILKK.....VVKLI.....YLVFD.....IIHRD.....ADFGV.....ALDVW.....
154284091	......-----.....GARVP.....YMVME.....IVHRD.....VDFGV.....AADIW.....
154311449	......AIMKK.....LVSLI.....YMVLE.....VVHRD.....VDFGV.....AADIW.....
154297741	......AILKK.....VVGLL.....YMVLE.....IVHRD.....SDFGV.....QIDVW.....
157106206	......RCMML.....VVGVD.....AFVMP.....VIHRD.....VDFGL.....AVDVW.....
109010209	......QCLTV.....VMGVK.....VIAMP.....IVHRD.....VDFGL.....AIDMW.....
145340316	......RMLER.....CIIKH.....CIILE.....VVHRD.....IDFNL.....KIDVW.....
169600479	......ELLFD.....VCPII.....VAVLP.....IIHRD.....VDFGL.....SIDMW.....
115402981	......ELLHD.....VCPLI.....VAVLP.....ILHRD.....VDFGL.....KIDMW.....
115774559	......SCLVR.....VMGVK.....VIVMP.....VIHRD.....VDFGL.....AVDMW.....
169865005	......FIMTC.....VSQLI.....AIVMP.....IIHRD.....CDFGL.....AVDVW.....

118094384	......QCLTV......VMGVK......VIVMP......IVHRD......VDFGL......AIDMW......
121704630	......ELLHD......VCPLI......VAVLP......ILHRD......VDFGL......KIDMW......
119493402	......ELLHD......VCPLI......VAVLP......ILHRD......VDFGL......KIDMW......
123498616	......QWLNK......IVQLY......TLVMG......IIHRD......IDFGL......AIDMW......
154418957	......QWICY......IVKCH......TLVME......IIHRD......IDFGL......TIDIW......
145482703	......GFLKL......LPQLV......YIVTE......IYHRD......IDYGL......EVDIW......
145486796	......GFLKL......MPQLV......YIVIE......IYHRD......IDYGL......EVDMW......
145493483	......GFLKL......MPQLV......YIVIE......IYHRD......IDYGL......EVDMW......
145473719	......GFLKL......MPQLV......YIVIE......IYHRD......IDYGL......EVDMW......
145532194	......GFLKL......MPQLV......YIVIE......IYHRD......IDYGL......EVDMW......
145551594	......GFLKL......LPQLV......YIVTE......IYHRD......IDYGL......EVDIW......
125575710	......KMLER......VIKYE......CFVLE......IVHRD......IDFNL......KVDVW......
126305839	......QCLTV......VMGVK......VIAMP......IVHRD......VDFGL......AIDMW......
145245599	......ELLHD......VCPLI......VAVLP......ILHRD......VDFGL......KIDMW......
146181421	......AFLTL......TPQMV......HIVIE......IYHRD......IDYGL......QVDIW......
146412640	......NLLYM......VAPLL......VAILP......IIHRD......VDFGL......KLDIW......
149247370	......NLLYM......VAPLL......LAILP......IIHRD......VDFGL......QIDVW......
147845401	......KMLER......IIKYE......CFVLE......VVHRD......IDFNL......KVDIW......
147854522	......RMLER......IIKYE......YCVLE......IVHRD......IDFNL......KVDIW......
148688225	......QCLTV......VMGLK......VIAMP......IVHRD......VDFGL......AIDMW......
150951632	......NLLYM......VAPLL......LAILP......VIHRD......VDFGL......KIDIW......
149709612	......QCLTV......VMGVK......VIAMP......IVHRD......VDFGL......AIDMW......
154304778	......DLLND......VCPLI......VAVLP......ILHRD......VDFGL......KIDIW......
157109668	......EVLRK......IVKLL......VIVME......LVHRD......TDFGA......NVDLW......
109018618	......EVLRK......IIKLF......VLVME......IVHRD......TDFGA......TVDLW......
109097635	......EVLKK......IVKLF......VLIME......IVHRD......TDFGA......TVDLW......
157817588	......EVLKK......IVKLF......VLIME......IVHRD......TDFGA......TVDLW......
157819637	......EVLRR......IVKLF......VLVME......IVHRD......SDFGA......TVDLW......
110758221	......EVLKK......IVQLL......VIVME......LVHRD......TDFGA......TVDLW......
112735201	......EVLKK......IVKLF......VLVME......IVHRD......TDFGA......TVDLW......
114572160	......EVLRK......IVKLF......VLVME......IVHRD......TDFGA......TVDLW......
114643875	......EVLKK......IVKLF......VLIME......IVHRD......TDFGA......TVDLW......
115684879	......QVLLR......IVCLE......ILIME......VVHRD......ADFGA......RIDLW......
115711978	......QVLLG......IVCLE......ILIME......VVHRD......ADFGA......RIDLW......
118790427	......EVLRK......IVKLL......VIVME......LVHRD......TDFGA......NVDLW......
118082383	......EVLTK......IVKLF......VLVME......IVHRD......TDFGA......TVDLW......
118102421	......EMLRK......IVKLF......VLVME......VVHRD......TDFGA......TVDLW......
119892390	......EVLKK......IVKLF......VLIME......IVHRD......TDFGA......TVDLW......
126306930	......EVLRK......IVKLF......VLVME......IVHRD......TDFGA......TVDLW......
148692474	......EVLKK......IVKLF......VLIME......IVHRD......TDFGA......TVDLW......
148707764	......EVLRR......IVKLF......VLIME......IVHRD......SDFGA......TVDLW......
149411606	......EVLRK......IIKLF......VLVME......IVHRD......TDFGA......TVDLW......
157128996	......ALLRE......VVRYF......YIAVE......IVHRD......SDFGL......SVDIF......
109116775	......QLLRE......VIRYF......YIAIE......IVHRD......SDFGL......TVDIF......
109489194	......QLLRE......VIRYF......YIAIE......IVHRD......SDFGL......TVDIF......
109492083	......QLLRE......VIRYF......YIAIE......IVHRD......SDFGL......TVDIF......
110737860	......QNLIA......IIRWY......YLSLE......IVHRD......SDMGI......AVDMF......
169601438	......KLLQE......VIRYF......YIAVE......IIHRD......SDFGL......AVDIF......
118403804	......QLLRE......VIRYY......YIALE......IVHRD......SDFGL......AVDIF......
115396926	......GLLQE......VIRYY......YIALE......IVHRD......SDFGL......AIDIF......
114661618	......QLLQE......VLRYF......YIALE......IVHRD......SDFGL......AVDIF......
115927437	......DLLRE......VIRYF......FIALE......IVHRD......SDFGL......AIDIF......
116000725	......ELMTA......LVPFF......FIAQE......ITHND......ADVGL......AADIW......
116061785	......DALIA......ILRCF......YMALE......IVHRD......ADMGL......SVDVF......
169862189	......SILQE......VIRYY......YIALE......LVHRD......SDFGL......SVDIF......
121703411	......GLLQE......VIRYF......YIALE......IVHRD......SDFGL......AIDIF......
119497439	......GLLQE......VIRYF......YIALE......IVHRD......SDFGL......AIDIF......
119576206	......QLLQE......VLRYF......YIALE......IVHRD......SDFGL......AVDIF......
153946421	......QLLRE......VIRYF......YIAIE......IVHRD......SDFGL......TVDIF......
125558274	......ENLIA......IVRMY......YISLE......IIHRD......SDMGI......AIDLF......
125600176	......ENLIA......IVRMY......YISLE......IIHRD......SDMGI......AIDLF......
125820487	......--LPE......IVRYV......YLVLQ......VLHRD......ADFGI......SSDIQ......
125950955	......KHLRD......IVRYV......YLALQ......VIHRD......ADFGL......SSDIQ......
145229543	......GLLQE......VIRYY......YIALE......IVHRD......SDFGL......AIDIF......
145342006	......QALIA......ILRCF......YMALE......IIHRD......ADMGL......SVDVF......
145341727	......ELMTA......LVPFF......FIAQE......ITHND......ADVGL......AADIW......
145604959	......RLLRE......VIRYY......YIALE......IVHRD......SDFGL......AIDIF......
145312265	......QLLQE......VLRYF......YIALE......IVHRD......SDFGL......AVDIF......
146418846	......SLLQE......VIRYF......YIALE......IVHRD......SDFGL......AIDIF......
149247889	......RLLQE......VIRYF......YIALE......IVHRD......SDFGL......AIDIF......

148342546	......QLLQE.....VLRYF.....YIALE.....IVHRD.....SDFGL.....AVDIF.....
148342482	......QLLQE.....VLRYF.....YIALE.....IVHRD.....SDFGL.....AVDIF.....
148702352	......QLLRE.....VIRYF.....YIAIE.....IVHRD.....SDFGL.....TVDIF.....
149054584	......QLLRE.....VIRYF.....YIAIE.....IVHRD.....SDFGL.....TVDIF.....
149054583	......QLLRE.....VIRYF.....YIAIE.....IVHRD.....SDFGL.....TVDIF.....
150865950	......RLLQE.....VIRYF.....YIALE.....IVHRD.....SDFGL.....AIDIF.....
149408999	......QLLRE.....VVRYF.....YIALE.....IVHRD.....SDFGL.....AVDIF.....
149635108	......QLLRE.....VIRYF.....YIAIE.....IVHRD.....SDFGL.....TVDIF.....
149723647	......QLLRE.....VIRYF.....YIAIE.....IVHRD.....SDFGL.....TVDIF.....
154280789	......GLLQE.....VIRYF.....YIGLE.....IVHRD.....SDFGL.....AIDIF.....
154299111	......KLLRE.....VIRYF.....YIALE.....IVHRD.....SDFGL.....AIDIF.....
108743472	......ASARS.....TAQVV.....WLATA.....VVHRD.....IDFGI.....ATDVF.....
157122452	......KIASN.....IIGYI.....LLLMP.....IIHRD.....CDFGS.....KADIW.....
157109838	......STLKQ.....IIKFV.....LLVTE.....INHRD.....CDFGS.....KTDVW.....
109101102	......DMHRL.....ILRLV.....WLLLP.....YAHRD.....MDLGS.....RTDVW.....
109101098	......DMHRL.....ILRLV.....WLLLP.....YAHRD.....MDLGS.....RTDVW.....
109472298	......QIMRD.....IVGYI.....LILMD.....IIHRD.....CDFGS.....KADIW.....
109499493	......TIMKE.....IVGYL.....LILME.....IIHRD.....CDFGS.....KADIW.....
110764434	......QIASN.....IIGYL.....LLLMP.....IIHRD.....CDFGS.....KADIW.....
169603361	......EAYTL.....IIHSI.....YILLP.....IAHRD.....MDLGS.....KVDIW.....
111221673	......EAARA.....TARLV.....WMATE.....IVHRD.....IDFGI.....AADVF.....
120612691	......RLLAQ.....LVKVF.....YMVMP.....TLHRD.....LDLGA.....RSDLY.....
115478322	......MVMRS.....VVALV.....LLVME.....IAHRD.....CDFGS.....KVDIW.....
115491099	......EAYTL.....IIHSI.....YILLP.....YAHRD.....MDLGS.....KVDIW.....
114577893	......QIMRD.....IVGYI.....LILMD.....IIHRD.....CDFGS.....KADIW.....
114577897	......QIMRD.....IVGYI.....LILMD.....IIHRD.....CDFGS.....KADIW.....
114583445	......DMHRL.....ILRLV.....WLLLP.....YAHRD.....MDLGS.....RTDVW.....
114583441	......DMHRL.....ILRLV.....WLLLP.....YAHRD.....MDLGS.....RTDVW.....
114577895	......QIMRD.....IVGYI.....LILMD.....IIHRD.....CDFGS.....KADIW.....
114592753	......CFMKK.....IVQFC.....LLLTE.....IIHRD.....CDFGS.....KQDIW.....
114594057	......TIMKE.....IVGYL.....LILME.....IIHRD.....CDFGS.....KADIW.....
114592745	......CFMKK.....IVQFC.....LLLTE.....IIHRD.....CDFGS.....KQDIW.....
114592763	......CFMKK.....IVQFC.....LLLTE.....IIHRD.....CDFGS.....KQDIW.....
114592749	......CFMKK.....IVQFC.....LLLTE.....IIHRD.....CDFGS.....KQDIW.....
114592747	......CFMKK.....IVQFC.....LLLTE.....IIHRD.....CDFGS.....KQDIW.....
114800033	......RILSA.....IVRIL.....WLVMD.....IIHRD.....LDFGV.....ASDIY.....
115379852	......TTLLM.....IIRHR.....YVVLE.....VLHRD.....IDFGC.....KADIF.....
115376629	......QAMAR.....VITVH.....FITLE.....LVHRD.....TDFGL.....RLDQF.....
115374942	......GILLL.....IIHTC.....YLVLD.....VLHRD.....IDLGC.....SDEIF.....
115503760	......QIMRD.....IVGYI.....LILMD.....IIHRD.....CDFGS.....KADIW.....
169859290	......EAYRR.....IIRIL.....YLFLP.....YAHRD.....MDFGS.....KVDIW.....
169845086	......DVMRV.....IVHLI.....FILME.....LLHRD.....CDFGS.....KSDVW.....
118090137	......TIMKE.....IVSYL.....LILME.....IVHRD.....CDFGS.....KADIW.....
118093736	......EMHNL.....ILRLE.....WLLLP.....YAHRD.....MDLGS.....RTDIW.....
118101288	......QIMRD.....IVGYI.....LILMD.....IIHRD.....CDFGS.....KADIW.....
118104296	......CFMKK.....IVQFC.....LLLTE.....IIHRD.....CDFGS.....KQDIW.....
121702607	......ETMKK.....IVKYI.....FLLME.....LLHRD.....CDFGS.....KSDIW.....
119484224	......EAYNL.....IIHSI.....YILLP.....YAHRD.....MDLGS.....KVDIW.....
119603051	......CFMKK.....IVQFC.....LLLTE.....IIHRD.....CDFGS.....KQDIW.....
119626237	......TIMKE.....IVGYL.....LILME.....IIHRD.....CDFGS.....KADIW.....
119626235	......TIMKE.....IVGYL.....LILME.....IIHRD.....CDFGS.....KADIW.....
119626240	......TIMKE.....IVGYL.....LILME.....IIHRD.....CDFGS.....KADIW.....
119626236	......TIMKE.....IVGYL.....LILME.....IIHRD.....CDFGS.....KADIW.....
119620253	......QIMRD.....IVGYI.....LILMD.....IIHRD.....CDFGS.....KADIW.....
119626238	......TIMKE.....IVGYL.....LILME.....IIHRD.....CDFGS.....KADIW.....
119626234	......TIMKE.....IVGYL.....LILME.....IIHRD.....CDFGS.....KADIW.....
119626239	......TIMKE.....IVGYL.....LILME.....IIHRD.....CDFGS.....KADIW.....
119903747	......QIMRD.....IVGYI.....LILMD.....IIHRD.....CDFGS.....KADIW.....
158522517	......QYCQR.....IIRLI.....YVMRR.....VWHRD.....ADFGI.....RADIF.....
123403698	......EIQKK.....VVKVF.....TILME.....ISHRD.....CDFGS.....KSDVW.....
123475740	......DVLQS.....IIKLF.....IILFE.....IIHRD.....CDFGS.....AADVW.....
123504247	......QFLQA.....IVKIV.....KFLFE.....ILHRD.....CDFGS.....PADVW.....
154416568	......FIPCS.....FVKVL.....MLQMK.....VYHKD.....TDFGL.....FFDKY.....
154421193	......NYQKA.....IVKVY.....LILME.....LIHRD.....CDFGS.....KYDIW.....
122891205	......EMHRL.....VLSLT.....WLLLP.....YAHRD.....MDLGS.....RTDIW.....
124008925	......ALTVS.....LLKPT.....YLVMP.....VLHQD.....SDFGS.....ASDIF.....
124266686	......KLLAR.....MVKVH.....YMAMP.....IYHRD.....LDFGA.....WTDLY.....
145509068	......DLHRK.....IVKCY.....YMVLE.....ITHRD.....CDFGS.....KVDIW.....
145516322	......EIMKR.....LVNFI.....LILME.....MTHRD.....CDFGS.....KADVW.....
145521576	......QQRKL.....LVRVF.....VVLGE.....KYHGD.....IDFIP.....KTDVF.....

```
145519147    ......NIMKK......IVRLI......CIVME......YLHRD......CDFGQ......KVDIW......
145524633    ......EIMKR......LVNFM......LILME......MTHRD......CDFGS......KADVW......
145524281    ......SIMTR......IVKLV......CLVME......ISHRD......CDFGS......KVDIW......
145528195    ......DILNK......IVKLE......ILILE......YVHRD......CDFGS......SADIF......
145529225    ......QIFKD......IAKAL......VIVFE......ITHRD......WNFQR......KVDIY......
145535740    ......DLHRQ......IVKCY......YMVLE......ITHRD......CDFGS......KVDIW......
125540098    ......QVMNL......VVTLV......LLVME......IAHRD......CDFGS......KVDIW......
125563031    ......MVMRS......VVALV......LLVME......IAHRD......CDFGS......KVDIW......
125824912    ......QIMRD......IVGFL......LILMD......IIHRD......CDFGS......KADIW......
125852863    ......CFMKK......VVQFC......LILTE......VIHRD......CDFGS......KQDIW......
126644030    ......KLLYR......ILNLI......WLVTE......IVHKD......CDFGD......KVDVW......
126304001    ......QIMRD......IVGYI......LILMD......IIHRD......CDFGS......KADIW......
126331118    ......TIMKE......IVGYL......LILME......IVHRD......CDFGS......KADIW......
154334560    ......DIVSR......IARVF......SIAME......IAHRD......TNFSN......KSDMW......
154336125    ......NIYRD......IVTYL......WVVME......VSHWD......CDFGS......KADIW......
146098459    ......SIYRD......IVTYL......WVVME......VSHWD......CDFGS......KADIW......
146081973    ......DIVSH......IVRVY......SIAME......ITHRD......TNFGN......KTDMW......
145253206    ......EAYNL......IIHSI......YILLP......YAHRD......MDLGS......KVDIW......
145614256    ......ETMKR......IVTYI......FLLME......LLHRD......CDFGS......KSDIW......
146181267    ......-----......IASII......EILMD......LIHKD......TDFGI......RSDIY......
146419529    ......DVMKT......IVQYY......LVLME......LIHRD......CDFGS......KADIW......
146417053    ......DAMKR......IVSYI......FLLME......LLHRD......CDFGL......KLDIW......
146413499    ......NNYHR......IVQSI......YILLP......YAHHD......VDLGS......KTDIW......
149246147    ......DAMRR......IVSYI......LLLME......LIHRD......CDFGS......KLDIW......
149239767    ......EVMKV......VVTYM......LVLME......LIHRD......CDFGS......KADIW......
149235514    ......KNYHR......IIQSI......NILLP......YAHHD......VDLGS......ATDIW......
147778998    ......LVMKV......VVTLH......LLVME......IAHRD......CDFGS......KVDIW......
148273354    ......DLMAQ......VLTVH......YLVME......VLHRD......SDFGI......RSEVW......
147842217    ......RVSSL......LLPLL......YLLFP......YAHND......MDFGS......RTDIW......
148277037    ......QIMRD......IVGYI......LILMD......IIHRD......CDFGS......KADIW......
148358417    ......DTLSK......--RLV......YILTT......LIHRD......VDFGS......ASDLF......
148666780    ......QIMRD......IVGYI......LILMD......IIHRD......CDFGS......KADIW......
148688403    ......TIMKE......IVGYL......LILME......IIHRD......CDFGS......KADIW......
148688152    ......CFLKK......IVQFC......LLLTE......IIHRD......CDFGS......KQDIW......
148688158    ......CFLKK......IVQFC......LLLTE......IIHRD......CDFGS......KQDIW......
148688402    ......TIMKE......IVGYL......LILME......IIHRD......CDFGS......KADIW......
156095931    ......HILKS......IVQYF......IMLME......IIHRD......CDFCS......KVDLW......
149177539    ......KAAGR......VVSIH......YIVME......LVHRD......ADFGL......RSDIY......
149197421    ......KLTAR......IMPIH......YFTMD......VIHLD......CDWGL......QSDIY......
149198638    ......HLTAL......IIPVY......FFTME......VIHLD......CDWGL......AADIY......
149195693    ......RLNAR......IMTVY......FFTMK......ILHLD......CDWGL......RSDIY......
150865648    ......DVMKT......IVKYY......LVLME......LIHRD......CDFGS......KADIW......
150864736    ......DAMRR......IVSYI......FLLME......LIHRD......CDFGS......KSDIW......
150863878    ......KNYHR......IIQSI......YVLLP......FAHRD......VDLGS......KTDIW......
149642275    ......CFMKK......IVQFC......LLLTE......IIHRD......CDFGS......KQDIW......
149642273    ......CFMKK......IVQFC......LLLTE......IIHRD......CDFGS......KQDIW......
149701682    ......TIMKE......IVGYL......LILME......IIHRD......CDFGS......KADIW......
149711139    ......DMHHL......ILRLV......WLLLP......YAHRD......MDLGS......RTDVW......
149727508    ......QIMRD......IVGYI......LILMD......IIHRD......CDFGS......KADIW......
149727510    ......QIMRD......IVGYI......LILMD......IIHRD......CDFGS......KADIW......
149922710    ......QALAK......VVAVH......FVGME......LIHRD......MDFGL......RSDQF......
149921228    ......KALAQ......VVAVH......FIVME......LVHRD......LDFGL......VSDQF......
149920767    ......QALAK......VVAVY......WVAMQ......LLHRD......MDFGL......AADQF......
149920851    ......ELMAA......IVEVF......YACME......VVHRD......IDFGI......RSDIY......
149918410    ......QALAK......VVQVY......FVAME......LVHRD......LDFGL......RSDQF......
149920986    ......QAMAR......VVAVY......FVAME......IVHRD......ADFGF......RTDQF......
149919761    ......QAMAR......VVTVH......FVAME......IVHRD......ADFGL......RTDQF......
149917529    ......QAMAQ......VVAIY......FIAME......LIHRD......TDFGL......AADQF......
154324130    ......EAYAL......IIHSV......YILLP......YAHRD......MDLGS......KVDIW......
115454247    ......QLIAT......IVEYK......CIIIG......ILHRD......GDFGL......KSDIW......
157133185    ......TILSQ......ILRYF......NMLTE......VIHRD......ADFGI......KSDIW......
157129109    ......EVFSK......IIAYL......FIEME......ILHRD......GDFGI......KSDIW......
157124777    ......EVFSK......IIAYL......FIEME......ILHRD......GDFGI......KSDIW......
109018375    ......NLLRE......IVRYY......YIVME......VLHRD......GDFGL......KSDIW......
109018373    ......NLLRE......IVRYY......YIVME......VLHRD......GDFGL......KSDIW......
109018970    ......DLLKQ......VIKYY......NIVLE......VMHRD......GDLGL......KSDIW......
109049391    ......QLLSK......IVKFH......CIITE......ILHRD......GDFGV......KSDIW......
109049403    ......QLLSK......IVKFH......CIITE......ILHRD......GDFGV......KSDIW......
109049400    ......QLLSK......IVKFH......CIITE......ILHRD......GDFGV......KSDIW......
109052569    ......TIIKQ......VVRYY......YIVME......IVHRD......TDFGL......KADVW......
109076142    ......AVLAN......IVQYR......YIVMD......ILHRD......GDFGI......KSDIW......
```

109076144	......AVLAN......IVQYR......YIVMD......ILHRD......GDFGI......KSDIW......
109076150	......AVLAN......IVQYR......YIVMD......ILHRD......GDFGI......KSDIW......
109076148	......AVLAN......IVQYR......YIVMD......ILHRD......GDFGI......KSDIW......
109084348	......VILAL......IIAYY......LIELE......ILHRD......GDYGL......KSDIW......
109113884	......QVLKL......VIEYY......MIAME......ILHRD......GDFGI......KSDIW......
109120826	......VLLAK......IVAFR......YIVME......VLHRD......GDFGS......KSDIW......
109120822	......VLLAK......IVAFR......YIVME......VLHRD......GDFGS......KSDIW......
109120844	......ILLAK......IVAFF......FIVME......ILHRD......GDFGI......KTDIW......
109498390	......NLLRE......IVRYY......YIVME......VLHRD......GDFGL......KSDIW......
109483100	......NLLRE......IVRYY......YIVME......VLHRD......GDFGL......KSDIW......
157786638	......QVLKL......VIEYY......MIAME......ILHRD......GDFGI......KSDIW......
157820741	......DLLKQ......VIKYY......NIVLE......VMHRD......GDLGL......KSDIW......
109503039	......QLLSQ......IVTYK......YIVMG......ILHRD......GDLGI......KSDVW......
109503788	......QLLSQ......IVTYK......YIVMG......ILHRD......GDLGI......KSDVW......
109503790	......QLLSQ......IVTYK......YIVMG......ILHRD......GDLGI......KSDVW......
109504436	......TLLAK......IVTFF......FIVME......VLHRD......GDFGT......KTDIW......
158631240	......VILAL......IIAYY......LIELE......ILHRD......GDYGL......KSDIW......
109730323	......AVLAN......IVQYR......YIVMD......ILHRD......GDFGI......KSDIW......
122920883	......NLLRE......IVRYY......YIVME......VLHRD......GDFGL......KSDIW......
145339108	......SLIAR......IVEFK......CIVTG......VLHRD......GDFGL......KSDIW......
110762582	......KCLYT......IIVYH......YILME......ILHRD......GDFGV......KCDIW......
169622559	......GHMAR......IVRLI......CILMY......VKHKD......SDFDI......PADIF......
169612345	......RMLQC......IVQYR......GIFMQ......IIHRD......GDFGL......RSDV-......
118404244	......QLLSK......IVKFH......CIITE......ILHRD......GDFGV......KSDIW......
111599430	......QLLSR......IVRFH......CIITE......ILHRD......GDFGV......KSDIW......
159900554	......GLLAT......IPKVY......YLVME......IIFRD......IDFGI......RGDVY......
118404818	......DLLKQ......VIKYY......NIVLE......VMHRD......GDLGL......KSDIW......
115386666	......NILSS......IVAYY......YLYME......ILHRD......GDFGL......RSDIW......
114572373	......NLLRE......IVRYY......YIVME......VLHRD......GDFGL......KSDIW......
114587228	......QLLSQ......IVTYK......YIVMG......ILHRD......GDLGI......KSDVW......
114589182	......QLLSK......IVKFH......CIITE......ILHRD......GDFGV......KSDIW......
114585769	......TIIKQ......IVRYY......YIVME......IVHRD......TDFGL......KADVW......
114589180	......QLLSK......IVKFH......CIITE......ILHRD......GDFGV......KSDIW......
114596802	......AVLAN......IVQYR......YIVMD......ILHRD......GDFGI......KSDIW......
114596812	......AVLAN......IVQYR......YIVMD......ILHRD......GDFGI......KSDIW......
114596800	......AVLAN......IVQYR......YIVMD......ILHRD......GDFGI......KSDIW......
114596806	......AVLAN......IVQYR......YIVMD......ILHRD......GDFGI......KSDIW......
114596808	......AVLAN......IVQYR......YIVMD......ILHRD......GDFGI......KSDIW......
114626652	......GLLKQ......IIKYL......NIVLE......VMHRD......GDLGL......KSDIW......
114649784	......VLLAK......IVAFK......YIVME......VLHRD......GDFGS......KSDIW......
114649822	......ILLEK......IVAFF......FIVME......ILHRD......GDFGI......KTDIW......
114649782	......VLLAK......IVAFK......YIVME......VLHRD......GDFGS......KSDIW......
115379519	......KLVAH......IVQIF......FLAME......LIHRD......ADFGI......RADVY......
115379666	......RTVGQ......IVPVH......YFVMK......FIHRD......MDWGL......RSDTY......
115375750	......RITAQ......VVTIL......FIALE......IVHRD......IDFGI......RADIF......
115378703	......DLSTQ......IVKVL......YIVME......IVHCD......GDFGV......EADLW......
115376819	......RITAA......IGQVF......YLAMD......VVHRD......IDFGI......RSDLF......
115372872	......RIGLR......LVTVY......YIAME......LVHRD......LDFGV......RSDLF......
115647291	......DLLKS......VIKYL......NIVLE......VMHRD......GDLGL......KSDIW......
115734267	......EILSM......IISYF......FIEME......VLHRD......GDFGI......KSDVW......
115898536	......AVLRK......IVSYA......YIVMD......ILHRD......GDFGI......--DIW......
115920194	......EILSM......IISYF......FIEME......VLHRD......GDFGI......KSDVW......
115948301	......DLLKS......VIKYL......NIVLE......VMHRD......GDLGL......KSDIW......
116626707	......KAASG......IVHIY......FIAME......IVHRD......LDFGL......RSDIF......
116622694	......KAASA......IIHIY......YIAME......VIHRD......LDFGL......RSDIF......
116283241	......QLLSQ......IVTYK......YIVMG......ILHRD......GDLGI......KSDVW......
116284336	......AVLAN......IVQYR......YIVMD......ILHRD......GDFGI......KSDIW......
118404706	......RLLSH......IVAYR......YIAMG......IMHRD......GDLGI......KSDVW......
169860454	......NILKD......IVRYH......YIIME......ILHRD......GDFGL......KSDIW......
147898616	......AVLAN......IVQYQ......YIVMD......ILHRD......GDFGI......KSDIW......
117616806	......GLLKQ......IIKYL......NIVLE......VMHRD......GDLGL......KSDIW......
117616540	......VLLAK......IVAFK......YIVME......VLHRD......GDFGS......KSDIW......
117616808	......QVLKL......VIEYY......MIAME......ILHRD......GDFGI......KSDIW......
118048828	......ELLQQ......IPRVY......YLTMD......LIYRD......IDFGI......QSDIY......
118048787	......ELLRS......LPVVI......TMIME......IIYRD......VDFGV......RSDIY......
156743279	......KILER......IPRVY......YLAMD......IIFRD......IDFGI......ESDIF......
156743216	......ELLSK......LPVVA......VMIME......IIYRD......IDFGV......RSDVY......
118084896	......ILLAK......VVAYK......YIVME......VLHRD......GDFGS......KSDIW......
118084918	......ILLAK......IVTFY......YIVME......ILHRD......GDFGI......KTDIW......
118089809	......AVLAN......IVLYR......YIVMD......ILHRD......GDFGI......KSDIW......
118091964	......VILAL......IIAYY......LIELE......ILHRD......GDYGL......KSDIW......

```
118096697    ......QLLSQ......IVAYR......YIVMG......ILHRD......GDLGI......KSDVW......
118100220    ......QVLKL......VIEYY......MIAME......ILHRD......GDFGI......KSDIW......
121704006    ......NILSS......IVAYY......YLYME......ILHRD......GDFGL......RSDIW......
119467302    ......NILSS......IVAYY......YLYME......ILHRD......GDFGL......RSDIW......
148839316    ......QLLSQ......IVTYK......YIVMG......ILHRD......GDLGI......KSDVW......
119571522    ......QVLKL......VIEYY......MIAME......ILHRD......GDFGI......KSDIW......
119571644    ......NLLCE......IVHYY......YIVME......VVRRD......GDLGL......KPDIW......
119584782    ......TIIKQ......IVRYY......YIVME......IVHRD......TDFGL......KADVW......
119887009    ......QLLSK......IVKFH......CIITE......ILHRD......GDFGV......KSDIW......
119629309    ......-----......I----......FIVME......ILHRD......GDFGI......KTDIW......
119599618    ......QLLSK......IVKFH......CIITE......ILHRD......GDFGV......KSDIW......
119599619    ......QLLSK......IVKFH......CIITE......ILHRD......GDFGV......KSDIW......
119607984    ......GLLKQ......IIKYL......NIVLE......VMHRD......GDLGL......KSDIW......
119625194    ......AVLAN......IVQYR......YIVMD......ILHRD......GDFGI......KSDIW......
119625196    ......AVLAN......IVQYR......YIVMD......ILHRD......GDFGI......KSDIW......
119625193    ......AVLAN......IVQYR......YIVMD......ILHRD......GDFGI......KSDIW......
119629313    ......VLLAK......IVAFK......YIVME......VLHRD......GDFGS......KSDIW......
149642939    ......GLLKQ......IIKYL......NIVLE......VMHRD......GDLGL......KSDIW......
119902923    ......VILAL......IIAYY......LIELE......ILHRD......GDYGL......KSDIW......
119908816    ......DLLKQ......VIKYY......NIVLE......VMHRD......GDLGL......KSDIW......
119911606    ......QVLKL......VIEYY......MIAME......ILHRD......GDFGI......KSDIW......
139948552    ......VLLAK......IVAFK......YIVME......VLHRD......GDFGS......KSDIW......
119920738    ......TIIKQ......VVRYY......YIVME......IIHRD......TDFGL......KADVW......
148223289    ......DLLKQ......VIKYL......NIVLE......IMHRD......GDLGL......KSDIW......
123407875    ......DLMNL......IVKLY......GILMD......YAHRD......IDFGV......MTDMY......
123430060    ......DVLHA......IVKYV......YIVME......ILHRD......GDFGI......KTDIW......
123437203    ......QFLSS......IVAHR......YIAME......VVHRD......GDFGV......KTDIW......
123447187    ......DCLMQ......IVRLY......YMVME......IAHCD......GDFGL......KSDIW......
123438935    ......KVLAA......IVKYI......YIVME......ILHRD......GDFGI......KTDIW......
123445773    ......KILKT......IIKVC......LILME......LIHRD......GDFGL......SADCW......
123452238    ......NILKK......IVTYY......FLIME......IIHRD......GDFGL......KSDIW......
123476041    ......RLMAS......IISFY......CVITE......VVHRD......GDLGI......KCDMW......
123476352    ......RLMAS......IIRFY......CIVTE......VVHRD......ADLGV......KCDMW......
123472115    ......DLLSK......IVAYK......YIAME......VLHRD......GDFGV......KADIY......
123475767    ......NALRN......VVGYY......YIVME......I-HRD......GDFGL......KSDIW......
123473282    ......TILST......IVRYR......FILMD......IIHRD......GDFGL......SADIW......
123503741    ......QVLSY......IITYI......HIVME......ILHRD......GDFGI......KTDIW......
123487738    ......NLLSE......IIRFY......CTVME......ILHRD......GDFGT......KTDIW......
123499908    ......NVLSA......IVRYI......YIIME......ILHRD......GDFGI......KTDIW......
154412419    ......TLLSN......IIAYI......SIVLE......ILHRD......GDFGI......PADIW......
123509244    ......RIMAS......IISFH......VIVSE......IVHRD......GDLGI......KCDIW......
123490481    ......EILST......IIRYR......YILMD......ILHRD......GDFGI......KSDIW......
123496113    ......NLLLK......IVRCY......HIVMD......IIHRD......GDFGI......KADMW......
154415262    ......KILSS......IIGYR......HIVMD......ILHRD......GDFGI......KSDIW......
123233332    ......GLLKQ......IIKYL......NIVLE......VMHRD......GDLGL......KSDIW......
160415985    ......GLLKQ......IIKYL......NIVLE......VMHRD......GDLGL......KSDIW......
145475619    ......RIMAQ......IINFR......CIVMD......VIHRD......GDFGI......KGDVW......
145476153    ......RILAS......IIGYR......FVVLE......IYHRD......GDLNV......KCDIW......
145480595    ......RILAS......IINYK......YIIME......IVHRD......GDLNV......MSDIW......
145478495    ......RILAS......IICYK......CIVME......ILHRD......GDMNV......KSDIW......
145480309    ......RILAS......IISYK......CIVME......IFHRD......GDMNV......KSDIW......
145480551    ......RILAS......IIEYK......YIVME......ILHRD......GDLNI......PCDIW......
145478637    ......RILAS......IIAYK......CVVME......ILHRD......GDMNV......KSDIW......
145477577    ......RILAS......IIAYK......CIIME......ILHRD......GDMNV......KSDIW......
145479697    ......RFLYS......IVAYK......CIVME......VVHRD......GDLNV......KSDIW......
145478159    ......RILAS......IISYK......CIVME......ILHRD......GDMNV......KSDIW......
145476697    ......MLLQK......IVQYI......IIIME......ILHRD......GDFGI......KSDVW......
145476173    ......RILAS......IVEFK......YVIME......ILHRD......GDLNI......PCDIW......
145476303    ......NILRE......IVQYY......YIITE......ILHRD......GDFGL......SSDIW......
145485921    ......RFIAS......VVSYK......CIIME......ILHRD......GDFNV......KADIW......
145483169    ......TLLKV......IIKYY......CLLME......IVHKD......GDFSI......KSDIW......
145484511    ......RILAS......IIGYK......CIVME......ILHRD......GDMNV......KSDIW......
145482405    ......KLMQP......IIKYL......IIATE......IMHRD......GDLGL......KSDIW......
145486507    ......NILRE......IIKYY......YIIME......ILHRD......GDFGL......KSDIW......
145486923    ......RILAS......IIGYK......CVIME......ILHRD......GDLNV......PSDIW......
145495691    ......RTLAS......-----......NVIME......ILHRD......GDFNI......PCDIW......
145494896    ......SILQN......IIQYV......CLIME......IIHRD......GDFGI......KSDIW......
145491241    ......DVLKN......IIEYR......CIVMD......IIHRD......GDFGI......KSDIW......
145493091    ......RILAS......IVEFK......YVIME......ILHRD......GDLNI......PCDIW......
145491895    ......KIMST......IINFR......CIVMD......ILHRD......GDFGI......KQDIW......
145494071    ......NILRE......IIKYY......YIIME......ILHRD......GDFGL......KSDIW......
145493782    ......KILEF......IVKFR......CIVME......IIHRD......GDFGI......MTDIW......
```

```
145499825   ......KIMST......INFR......CIVMD......ILHRD......GDFGI......KQDIW......
145497435   ......RIMSE......INFR......CIVMD......IIHRD......GDFGI......KGDIW......
145495812   ......RILAS......ISYK......CIVME......IFHRD......GDMNV......KSDIW......
145502633   ......RFIAS......VVSYK......CIIME......ILHRD......GDFNV......KADLW......
145502941   ......KIMKD......VIKYY......CIVME......IIHRD......GDFGI......KSDMW......
145502897   ......SILAK......IVKYY......CIVME......VLHRD......GDLGV......KSDIW......
145501427   ......KILEF......IVRFK......CIVME......IIHRD......GDFGI......MTDIW......
145510464   ......HVLKA......IITYR......CIVMD......ILHRD......GDFGI......KSDIW......
145510560   ......KILQC......IVKFK......CIVME......IIHRD......GDFGI......ASDIW......
145511622   ......ELLKS......IVQYM......IILME......ILHRD......GDFGV......KSDVW......
145510470   ......RILAS......VIQYK......CIVME......IYHRD......GDMNV......KSDIW......
145510052   ......RIMAQ......IINFR......CIVMD......VIHRD......GDFGI......KGDVW......
145515327   ......RILEA......IVKFR......CIVMD......IIHRD......GDFGI......KTDIW......
145515241   ......RLLQK......IVAYK......NIVMI......ILHRD......GDFGI......KSDVW......
145515319   ......KLLRS......IISHY......CIVME......IIHRD......GDFGI......KIDIW......
145514151   ......SLLRV......IIKYY......NIIME......ILHRD......GDFGI......KSDIW......
145514319   ......LILQN......IIQYV......CIIME......IIHRD......GDFGI......KSDIW......
145517328   ......KVLKS......IIKHY......CILME......ILHRD......GDFGI......KIDIW......
145516184   ......KVLQQ......IIKFR......CIIME......ILHRD......GDFGI......ASDIW......
145514664   ......SLLRV......IIKYY......NIIME......ILHRD......GDFGI......KSDIW......
145514149   ......SLLRV......IIKYY......NIIME......ILHRD......GDFGI......KADIW......
145517901   ......RILAS......IIGYR......FLILE......IYHRD......GDLNV......KCDIW......
145521294   ......MLLQK......IVQYT......IIIME......ILHRD......GDFGI......KSDVW......
145518804   ......KVLKS......IIKHY......CILME......ILHRD......GDFGI......KIDIW......
145522536   ......DVLKN......IIEYR......CIVMD......IIHRD......GDFGI......KSDIW......
145523431   ......RFIAS......VVSYK......CIIME......ILHRD......GDFNV......KADIW......
145524036   ......RFIAS......VVSYK......CIIME......ILHRD......GDFNV......KADIW......
145523832   ......SILAK......IVKYY......CIVME......VLHRD......GDLGV......KSDIW......
145521823   ......RILAS......IIGYK......CVIME......ILHRD......GDMNV......PSDIW......
145523345   ......KFLST......IVNFL......CLTME......ILHRD......GDFGI......SIDIW......
145527250   ......RIMSE......IINFR......CIVMD......IIHRD......GDFGI......KGDIW......
145524838   ......RILAS......IVEFK......YVIME......ILHRD......GDLNI......PCDIW......
145525523   ......RLLQK......IVAYK......NIVMI......ILHRD......GDFGI......KSDVW......
145530816   ......RILAS......IIAYK......CVVME......ILHRD......GDMNV......KSDIW......
145533801   ......DILFR......IINVY......ILIMD......VIHRD......ADFGV......SSDLW......
145532224   ......RLLAS......IIGYK......GIIME......IFHRD......GDLNV......KSDIW......
145533394   ......RIMAQ......IINFR......CIVMD......IIHRD......GDFGI......KGDVW......
145537706   ......SIMKV......IIKFY......CIIME......IVHRD......GDFSI......KTDIW......
145536636   ......RILAS......IISYK......CIVME......VMHRD......GDLNV......KSDIW......
145538263   ......RILAS......IISYK......CIIME......VMHRD......GDMNV......KSDIW......
145538447   ......NLLK-......VSVMK......TIVYE......IYPMD......HSIQ-......KCELF......
145535277   ......ELLKS......IVQYM......IILME......ILHRD......GDFGV......KSDVW......
145537458   ......RLLAS......IVAYK......CTVME......IVHRD......GDLNV......KSDIW......
145541052   ......RLLAS......IVAYK......CTVME......IVHRD......GDLNV......KSDIW......
145539982   ......QIHLE......IVKLY......CLVIE......IVHRD......CDFGL......KADVW......
145539814   ......RFLYS......IVAYK......CIVME......VVHRD......GDLNV......KSDIW......
145540832   ......NIMKV......IIKFY......CIIME......IVHRD......GDFSI......KTDIW......
145546127   ......RILAS......VIQYK......CIVME......IYHRD......GDMNV......KSDIW......
145544465   ......QLSKV......VIRFY......GIITE......ICHRD......IDFGE......KSDVF......
145547100   ......KLMQP......IIKYL......IIATE......IMHRD......GDLGL......KSDIW......
145547687   ......RILAS......IINYK......YIIME......IVHRD......GDLNV......MSDIW......
145546869   ......KLMQP......IIKYL......IIATE......IMHRD......GDLGL......KSDIW......
145546157   ......QLLRK......VITYY......CIIME......IIHRD......GDLGV......KVDIW......
145545933   ......QIMHT......IVSYY......NIFME......IVHRD......SDLGE......KIDIW......
145543248   ......QLLRT......IISYY......CIVME......IIHRD......GDLGV......KVDIW......
145549205   ......TLLKV......IIKYY......CILME......IVHKD......GDFSI......KSDIW......
145552505   ......RILAS......IISYK......CIVME......ILHRD......GDMNV......KSDIW......
145552322   ......RLLQK......IVAYK......NIVMI......ILHRD......GDFGI......KSDVW......
145473745   ......RLLAS......IIGYK......GIIME......IFHRD......GDLNV......KSDIW......
145551715   ......QLLRK......IITYY......CIIME......IIHRD......GDLGV......KVDIW......
145549816   ......RILAS......VIQYK......CIVME......IYHRD......GDMNV......KSDIW......
145550947   ......SMIQH......IINYY......FSYYE......YNHKD......CPIGV......KIDSF......
145551911   ......QIMHT......IVSYY......NIFME......IMHRD......SDLGV......KIDIW......
145552230   ......KLLRS......IISHY......CIVME......IIHRD......GDFGI......KIDIW......
145550415   ......RILAS......VIQYK......CIVME......IYHRD......GDMNV......KSDIW......
145549812   ......HVLKA......IITYR......CIVMD......ILHRD......GDFGI......KSDIW......
145551749   ......RILAS......VIQYK......CIVME......IYHRD......GDMNV......KSDIW......
125528482   ......ALIAR......IVEFK......CIVTG......VLHRD......GDFGL......KSDIW......
166233965   ......QLIAT......IVEYK......CIVIG......ILHRD......GDFGL......KSDIW......
125540093   ......SLMAS......IVEYK......CIVTS......VLHRD......ADFGL......KSDIW......
125552497   ......ALIAR......IVEFK......CIVTG......VLHRD......GDFGL......KSDIW......
125587220   ......QLIAT......IVEYK......CIIIG......ILHRD......GDFGL......KSDIW......
```

125582699	......SLMAS.....IVEYK.....CIVTS.....VLHRD......ADFGL......KSDIW......
125837012	......VLLSK.....IVAFR.....YIVME.....VLHRD......GDFGS......KSDVW......
125840692	......TIIKQ.....IVKYF.....YIVME.....IIHRD......TDFGL......KADVW......
125841329	......-----.....---YR.....YIVME.....VLHRD......GDFGC......KSDIW......
125845863	......NFLLH.....IVHHK.....YLVLD.....ILHKD......GEF--......KSEIW......
125845873	......-----.....---YR.....YIVME.....VLHRD......GDFGC......KSDIW......
125846325	......AVLAN.....IVQYK.....YIVMD.....ILHRD......GDFGI......KSDIW......
125846321	......AVLAN.....IVQYK.....YIVMD.....ILHRD......GDFGI......KSDIW......
125849127	......DLLKQ.....VIKYL.....NIVLE.....VMHRD......GDLGL......KSDIW......
125855947	......NFLLH.....IIHHK.....YLVLD.....ILHKD......GEFD-......KSEIW......
125594395	......ALIAR.....IVEFK.....CIVTG.....VLHRD......GDFGL......KSDIW......
126643953	......NVLRK.....IIKYI.....YIIME.....VLHRD......GDFGL......KSDIW......
126654112	......KLLSS.....IVCFR.....NIVME.....ILHRD......GDFGI......SSDIW......
126654390	......EIMKS.....VIKCH.....VIIME.....IIHRD......GDFGI......KSDIW......
126653841	......KIREL.....IVQYR.....FQLME.....ILHRD......SDFGT......YSDIW......
126282301	......VILAL.....IIAYY.....LIELE.....ILHRD......GDYGL......KSDIW......
126282298	......VILAL.....IIAYY.....LIELE.....ILHRD......GDYGL......KSDIW......
126294283	......DLLKQ.....IIKYL.....NIVLE.....VMHRD......GDLGL......KSDIW......
126306795	......NLLRE.....IVRYY.....YIVME.....VLHRD......GDFGL......KSDIW......
126306572	......DLLKQ.....VIKYY.....NIVLE.....VMHRD......GDLGL......KSDIW......
126314043	......QVLKL.....VIEYY.....MIAME.....ILHRD......GDFGI......KSDIW......
126327663	......ILLAK.....IVAFK.....YIVME.....VLHRD......GDFGS......KSDIW......
126331393	......EVLAN.....IVLYR.....YIVMD.....ILHRD......GDFGI......KSDIW......
126336309	......QLLSQ.....IVTYR.....YIVMG.....ILHRD......GDLGI......KSDVW......
126336311	......QLLSQ.....IVTYR.....YIVMG.....ILHRD......GDLGI......KSDVW......
126341658	......QLLSK.....IVKFH.....CIITE.....ILHRD......GDFGV......KSDIW......
126341732	......TIIKQ.....VVRYY.....YIVME.....IVHRD......TDFGL......KADIW......
126660167	......NILDT.....IPAIY.....YLVEE.....LLHRD......IDFGG......TSDIY......
134094246	......RAAGR.....IVTIY.....YIAME.....VVHRD......VDFGI......RTDIY......
133777119	......QVLKL.....IIEYY.....MIAME.....ILHRD......GDFGI......KSDIW......
154332633	......NILQK.....IVRYA.....YIVME.....LLHRD......GDFGI......KSDMW......
154332276	......NASLR.....IVRFL.....FVVMS.....VIHRD......GDFGS......AADVW......
154334385	......RVLSS.....IINYV.....CIVME.....ILHRD......GDFGI......RSDVW......
154331458	......KVLRV.....LVQYV.....FIMME.....ILHRD......GDFGI......AADVW......
154332278	......ILMRD.....VVSQR.....CLVMD.....VVHRD......GDFGI......KSDVW......
154342802	......KCLAS.....IIKYI.....LIVME.....MLHRD......GDFGF......KADVW......
154344100	......QCLAS.....IIKYH.....VIIME.....ILHRD......GDFGF......KADVW......
154341286	......RILAA.....IIRYH.....FIIME.....ILHRD......GDFGI......KSDVW......
154337146	......SLLRN.....IITYY.....CLVTE.....IVHRD......GDFGV......GADIW......
154339072	......RILEE.....VVEYK.....FILME.....ILHRD......SDFGT......ASDVW......
154341306	......IVMDN.....VVKFR.....DIIME.....ILHRD......GDFGV......RSDVW......
154339575	......ATLSS.....IVPYI.....CLAMR.....LIHRD......GDYGL......KTDIF......
154342893	......TVLQQ.....IVRYV.....YIVME.....ILHRD......GDFGI......KSDIW......
154343217	......MCLAH.....IIRYY.....IIITE.....MIHRD......GDFGF......KADVW......
154337288	......DMLRT.....ITRYV.....FIVME.....ILHRD......GDFGI......KSDVW......
154342748	......KCLSR.....IVRCE.....LIVME.....MMHRD......GDFGF......KSEMW......
154340781	......LGMMG.....VVTPH.....CILMD.....RVHRD......GDFGS......KADVW......
154341767	......KVLLQ.....IVSYV.....CLVME.....FLHRD......GDFGL......KVDIW......
154337543	......ENMRC.....IISLI.....LYILE.....IMHRD......GDFGF......KADIW......
146081746	......RVLSS.....IINYV.....CIVME.....ILHRD......GDFGI......RSDVW......
146086670	......ENMRC.....IISLI.....LYILE.....IMHRD......GDFGF......KADIW......
146075507	......KVLRV.....LVQYV.....FIMME.....ILHRD......GDFGI......AADVW......
146096121	......TVLQQ.....IVRYV.....YIVME.....ILHRD......GDFGI......KSDIW......
146093355	......RILAA.....IIRYH.....FIIME.....ILHRD......GDFGI......KSDVW......
146090483	......ATLSS.....IVAYI.....CLAMK.....LIHRD......GDYGL......KTDIF......
146095707	......KCLAS.....IIKYI.....LIVME.....MLHRD......GDFGF......KADVW......
146095599	......KCLSR.....VVRCE.....LIVME.....MMHRD......GDFGF......KSEMW......
146093900	......KVLLQ.....IVSYV.....CLVME.....FLHRD......GDFGL......KVDIW......
146096611	......MCLAH.....IIRYY.....VIVTE.....MIHRD......GDFGF......KADVW......
146086267	......DMLRT.....ITRYV.....FIVME.....ILHRD......GDFGI......KSDVW......
146085938	......SLLKN.....IITYY.....CLVTE.....IVHRD......GDFGV......GADIW......
146082690	......RFMTV.....LVEYL.....CICMP.....IVHGD......TDLDA......SSDMW......
146077250	......NASLR.....IVRFL.....FVVMS.....VIHRD......GDFGS......AADVW......
146104147	......CCLLS.....IVKCH.....AMVLD.....MIHRD......GDFGF......KADMF......
146104429	......MCLRE.....IVRLE.....LLLME.....ILHHD......GDFGF......KSEMW......
146093365	......IVMDN.....IVKFR.....DIIME.....ILHRD......GDFGV......RSDVW......
154345702	......CCLLS.....IVKCH.....AMVLD.....MIHRD......GDFGF......KADMF......
146088949	......RILEE.....VVEYK.....FILME.....ILHRD......SDFGT......ASDVW......
146102203	......HCLAA.....IVKHF.....LLIME.....MMHRD......GDFGF......KADMW......
146104145	......DCLLN.....IVKCH.....ALVLD.....MIHRD......GDFGF......KADMF......
154345700	......DCLLN.....IVKCH.....ALVLD.....MIHRD......GDFGF......KADMF......
146077254	......ILMRD.....VVSQR.....CLVMD.....VVHRD......GDFGI......KSDVW......
146097973	......QCLAS.....IIKYY.....LIIME.....MLHRD......GDFGF......KADVW......

145247224	......NILSS......IVAYY......YLYME......ILHRD......GDFGL......RSDIW......
146092640	......LGMMN......VVTSH......CLLMD......RVHRD......GDFGS......KADIW......
145294096	......QNSGR......IVAVF......YIVME......IIHRD......MDFGI......RSDIY......
145566895	......TIIKQ......VVRYY......YIVME......IVHRD......TDFGL......KADVW......
153816016	......DILKQ......LPQVY......YTVMD......ILHSD......IDFNI......KSDIY......
146183966	......HVLKA......IITYR......CIIME......ILHRD......GDFGI......KSDIW......
146182956	......KVLEN......IIKYY......CIVME......ILHRD......GDFGV......KVDIW......
146180906	......NILRE......VVRYY......YIIME......IMHRD......GDFGL......KSDIW......
146178978	......HILSK......VVKYY......CIVME......ILHRD......GDLGV......KSDIW......
146167890	......ELLKS......IVAYK......IIIME......ILHRD......GDFGI......KSDVW......
146165218	......KFLKE......IIAYR......CIVMD......ILHRD......GDFGI......KTDIW......
146163335	......SILKS......IIKYQ......CIVMD......IIHRD......GDFGI......KVDIW......
146161471	......TLLKV......IIRYY......HIIME......ILHRD......GDFGI......KADIW......
146417212	......RILRE......IVKYY......HIYME......VIHRD......GDFGL......VCDIW......
149234541	......RILKE......IVRYY......HIYME......IIHRD......GDFGL......VCDIW......
147782356	......-----......-----......CIVTG......VLHRD......GDFGL......KSDIW......
147800246	......NLISK......IVGYK......CIVTS......VLHRD......GDFGL......KSDIW......
147843683	......ELIAR......IVEYK......CIVTG......VLHRD......GDFGL......KSDIW......
148342563	......QLLSQ......IVTYK......YIVMG......ILHRD......GDLGI......KSDVW......
148681030	......NLLRE......IVRYY......YIVME......VLHRD......GDFGL......KSDIW......
148681031	......NLLRE......IVRYY......YIVME......VLHRD......GDFGL......KSDIW......
148692831	......QLLSQ......IVTYK......YIVMG......ILHRD......GDLGI......KSDVW......
148689159	......QLLSR......IVRFH......CIITE......ILHRD......GDFGV......KSDIW......
148692832	......QLLSQ......IVTYK......YIVMG......ILHRD......GDLGI......KSDVW......
148700958	......VLLAK......IVAFK......YIVME......VLHRD......GDFGS......KSDIW......
148700960	......ILLAR......IVTFF......FIVME......ILHRD......GDFGT......KTDIW......
148696697	......AVLAN......IVQYK......YIVMD......ILHRD......GDFGI......KSDIW......
156093574	......ELLSK......IVKYI......RIVMK......ILHRD......CDFGI......PSDVW......
156101596	......HIMQK......IVQVV......SLITE......ITHGD......GDFGI......LSDLF......
156095326	......RLIEV......IVRYK......YVAMD......LIHRD......GDFGL......PADIW......
156103357	......NVMRE......IVRYI......YILME......VLHRD......GDFGL......KSDMW......
157823777	......QLLST......IVRFH......CIITE......ILHRD......GDFGV......KSDIW......
157817763	......VILAL......IIAYY......LIELE......ILHRD......GDYGL......KSDIW......
157819907	......AVLAN......IVQYK......YIVMD......ILHRD......GDFGI......KSDIW......
149034203	......QLLSQ......IVTYK......YIVMG......ILHRD......GDLGI......KSDVW......
149057743	......TLLAK......IVTFF......FIVME......VLHRD......GDFGT......KTDIW......
149057744	......TLLAK......IVTFF......FIVME......VLHRD......GDFGT......KTDIW......
149057745	......VLLAK......IVAFK......YIVME......VLHRD......GDFGS......KSDIW......
149196554	......RLTAR......IVPIH......FFTMK......VLHLD......VDWGL......SSDIY......
149195761	......RITSQ......IMTVY......YFTME......VLHLD......CDWGL......TADIY......
149199721	......RLTAA......IIPLH......WFTMK......VLHLD......CDWGL......HTDIF......
149197759	......ETASQ......IVRIF......YIVME......MIHRD......CDFGL......RSDIY......
149196200	......RLTAM......IISIH......FFTMD......IIHLD......CDWGL......KADVF......
150866571	......RILRE......IVKYY......HIYME......VIHRD......GDFGL......VCDIW......
149470428	......QLLSQ......IVTYR......YIVMG......ILHRD......GDLGI......KSDVW......
149481707	......QVLKL......VIEYY......MIAME......ILHRD......GDFGI......KSDIW......
149632095	......LLLSK......IVKFY......CIITE......ILHRD......GDFGV......KSDIW......
149632394	......DLLKQ......IIKYL......NIVLE......VMHRD......GDLGL......KSDIW......
149635868	......ILLAK......IVAFF......YIVME......ILHRD......GDFGI......KTDIW......
149635866	......ILLAK......IVTFK......YIVME......VLHRD......GDFGS......KSDIW......
149636763	......AILGQ......IVTCH......FIVQD......ILHRD......GDFGV......KSDIW......
149641525	......NLLRE......IVRYY......YIVME......VLHRD......GDFGL......KSDIW......
149708630	......NLLRE......IVRYY......YIVME......VLHRD......GDFGL......KSDIW......
149724112	......QVLKL......VIEYY......MIAME......ILHRD......GDFGI......KSDIW......
149728591	......QLLSQ......IVTYK......YIVMG......ILHRD......GDLGI......KSDVW......
149728589	......QLLSQ......IVTYK......YIVMG......ILHRD......GDLGI......KSDVW......
149729670	......TIIKQ......VVRYY......YIVME......IVHRD......TDFGL......KADVW......
149729854	......QLLSK......IVKFH......CIITE......ILHRD......GDFGV......KSDIW......
149730282	......VLLAK......IVTFK......YIVME......VLHRD......GDFGS......KSDIW......
149737528	......VILAL......IIAYY......LIELE......ILHRD......GDYGL......KSDIW......
149738333	......GLLKQ......IIKYL......NIVLE......VMHRD......GDLGL......KSDIW......
149743920	......DLLKQ......VIKYY......NIVLE......VMHRD......GDLGL......KSDIW......
149917847	......RVSML......IVQVF......YMVME......LVHRD......TDFGV......RIDLF......
149921537	......VWGAR......IARVY......WFVME......VVHRD......LDFGV......RADIY......
153812892	......DVLKN......LPQVY......YTVMT......IVHGD......IDFNI......RSDLY......
150384316	......RLACR......VVGVI......YMVME......IVHRD......ADLGI......RSDIY......
154277496	......NILSS......IVAYY......YLYME......ILHRD......GDFGL......YSDIW......
154300010	......AILSS......IVGYY......HLYME......ILHRD......GDFGL......KSDIW......
154300048	......RILRF......ICN-L......GLYFE......IFQND......GDFGV......RGEVW......
157132489	......HYNYQ......ILSCY......VYAQE......LVHRD......CDFGT......SSDSW......
157108304	......HFSLH......IVTTY......VFTQE......LIHRD......CDFGE......AHDVW......

109126130	......CVGRC.....LLTLA.....AFAQE.....LVHAD.....GDLGL.....AVDSW.....
109126128	......CVGLS.....IVTAY.....SFLTE.....LVYRD.....TDFGH.....ALDAW.....
109128049	......SITNS.....IIKVF.....VFAQE.....LVHRD.....ADFGM.....GVDVW.....
109458112	......CVGLS.....IVAAY.....SFLTE.....LVYRD.....TDFGH.....ALDAW.....
109458110	......CVGRC.....LLQTL.....AFAQE.....LVHAD.....GDLGL.....AVDSW.....
109461208	......CVGLS.....IVAAY.....SFLTE.....LVYRD.....TDFGH.....ALDAW.....
110759906	......HYGLH.....IITTY.....VFSQE.....LVHRD.....CDFGE.....SHDVW.....
110763004	......HYSYH.....ILCSY.....VFAQE.....LAHRD.....CDFGC.....SADCW.....
114049841	......EVGSL.....AMPVW.....VMPPA.....WIHRD.....ADWGI.....PTDIY.....
114679136	......CVGLS.....IVTAY.....SFLTE.....LVYRD.....TDFGH.....ALDAW.....
149256768	......CVGLS.....IVTAY.....SFLTE.....LVYRD.....TDFGH.....SLDAW.....
118086596	......CVALS.....IIGMF.....GFLYE.....LVYRD.....TDFGL.....SMDAW.....
118117777	......SITNT.....IIKVF.....VFAQE.....LVHRD.....ADFGM.....SIDVW.....
118573872	......CVGLS.....IVTAY.....SFLTE.....LVYRD.....TDFGH.....ALDAW.....
119592794	......CVGLS.....IVTAY.....SFLTE.....LVYRD.....TDFGH.....ALDAW.....
119911108	......CVGRC.....LLTLA.....AFAQE.....LVHAD.....GDLGL.....ALDSW.....
119911110	......CVGLS.....IVAAC.....SFLTE.....LVYRD.....ADFGH.....ALDAW.....
125822033	......SISLY.....IINMY.....IFAQE.....LVHRD.....SDFGM.....STDVW.....
125823125	......CISLH.....IVGLF.....GFAQE.....LVHRD.....ADFGL.....SLDTW.....
125830267	......SISLY.....IINMF.....VFAQE.....LVHRD.....SDFGM.....STDVW.....
160333570	......NLSCF.....LTRAL.....VFAQE.....FVHRD.....GDFGL.....SLDTW.....
125844668	......NLSAF.....LTSAL.....VFAQQ.....FVHRD.....GDFGM.....CTDSW.....
125854025	......SLNAA.....IIKVL.....VFGQE.....LVHRD.....ADLGM.....TQDVW.....
126335569	......SITNT.....IIKVF.....VFAQE.....LVHRD.....ADFGM.....GVDVW.....
126344657	......CVGRC.....LLTLA.....AFAQE.....LVHAD.....ADFGL.....ELDSW.....
126344657	......CVGLA.....IVATY.....GFLSE.....LVYRD.....TDFGH.....SLDAW.....
156717768	......SVTSA.....IIRAF.....VFAQE.....LVHRD.....ADFGM.....SLDVW.....
148699326	......CVGLS.....IVTAY.....SFLTE.....LVYRD.....TDFGH.....SLDAW.....
149256090	......CVGRC.....LLQTL.....AFAQE.....LVHAD.....GDLGL.....AVDSW.....
149465055	......TVGRC.....LLTLP.....GFMQE.....LVHGD.....GDFGL.....SLDSW.....
149633627	......SITNT.....IIKVF.....VFAQE.....LVHRD.....ADFGM.....SIDVW.....
149722614	......CVGLS.....IVAAY.....SFLTE.....LVYRD.....TDFGH.....ALDAW.....
157124480	......EIQSH.....ILRMY.....YLILE.....VIHRD.....ADFGW.....NVDLW.....
157114085	......EIQSR.....ILRLY.....YLALE.....VIHRD.....ADFGW.....SVDQW.....
109113312	......EIQAH.....ILRLY.....YLILE.....VIHRD.....ADFGW.....KVDLW.....
109126264	......EIQAH.....ILRLY.....YLILE.....VIHRD.....ADFGW.....KVDLW.....
115386018	......EIQSN.....VLRLY.....FLILE.....VMHRD.....SDFGW.....KVDLW.....
114669324	......EIQAH.....ILRLY.....YLILE.....VIHRD.....ADFGW.....KVDLW.....
114679331	......EIQAH.....ILRLY.....YLILE.....VIHRD.....ADFGW.....KVDLW.....
114679333	......EIQAH.....ILRLY.....YLILE.....VIHRD.....ADFGW.....KVDLW.....
114679337	......EIQAH.....ILRLY.....YLILE.....VIHRD.....ADFGW.....KVDLW.....
114679335	......EIQAH.....ILRLY.....YLILE.....VIHRD.....ADFGW.....KVDLW.....
115749607	......EIQAH.....ILRLY.....YLILE.....VIHRD.....ADFGW.....KVDLW.....
115939302	......EIQSH.....ILRLF.....YLILE.....VIHRD.....ADFGW.....KVDLW.....
115945623	......EIQSH.....ILRLF.....YLILE.....VIHRD.....ADFGW.....KVDLW.....
116061136	......AVYSR.....IAAFH.....YIVHE.....VVHRD.....IDFGF.....AIDYW.....
116060004	......EIQSH.....ILRLY.....FLILE.....VIHRD.....ADFGW.....AVDVW.....
169857622	......EIQQN.....VLRLY.....FLMLE.....VIHRD.....GDFGW.....RVDYW.....
117949325	......EIQAH.....ILRLY.....YLILE.....VIHRD.....ADFGW.....KVDLW.....
119390407	......EIQSH.....ILRLY.....YLILE.....VIHRD.....ADFGW.....KVDLW.....
119390405	......EIQSH.....ILRLY.....YLILE.....VIHRD.....ADFGW.....KVDLW.....
121698051	......EIQSN.....VLRLY.....FLILE.....VMHRD.....SDFGW.....KVDLW.....
119480493	......EIQSN.....VLRLY.....FLILE.....VMHRD.....SDFGW.....KVDLW.....
119495781	......EIQSN.....ILRLY.....ILILE.....VIHRD.....SDFGW.....NIDLW.....
119595966	......EIQSH.....ILRLY.....YLILE.....VIHRD.....ADFGW.....KVDLW.....
119610482	......EIQAH.....ILRLY.....YLILE.....VIHRD.....ADFGW.....KVDLW.....
119610479	......EIQAH.....ILRLY.....YLILE.....VIHRD.....ADFGW.....KVDLW.....
123976715	......EIQSH.....ILRLY.....YLVME.....VIHRD.....SDFGW.....AVDIW.....
123470599	......EIQAH.....VLRLY.....YLILE.....VIHRD.....ADFGW.....SVDIW.....
123472445	......EIQSH.....IIRLY.....CLIIE.....IIHRD.....ADFGW.....SVDIW.....
123469147	......EIQSH.....IIRLY.....YLIIE.....IIHRD.....ADFGW.....SVDIW.....
123478685	......GLHSS.....ILRLY.....MMILE.....VLHRD.....GDFGF.....SADVW.....
124378046	......EIQAH.....ILRLY.....YLILE.....VIHRD.....SDFGW.....MVDLW.....
145479457	......KLQGY.....ILKLY.....VLILE.....IIHRD.....ADMGL.....TVDIW.....
145475975	......QIMKQ.....IVKLF.....YLLLE.....IMHRD.....GDFGI.....RIDIW.....

145480725	......KIQYL......ITKLY......VLVLE......VIHRD......ADFSF......KSDIW......
145477429	......KIQRK......IIKLD......YLILE......IIHRD......CDFGW......KLDIW......
145479815	......KIQSY......ILKLF......VLILE......VIHRD......ADFGL......SVDLW......
145480177	......KIQYL......ITRLY......VLVLE......VIHRD......ADFSF......KIDIW......
145477417	......DIQSS......IVKMF......YLITE......IMHRD......GDFGF......RVDIW......
145476391	......TIQQM......IIQLT......CLVME......IIHRD......SDFGW......SIDLW......
145483453	......KIMYS......ILKLY......YLILE......IVHRD......ADFGW......QVDIW......
145481507	......KLHRK......IVKLY......YLVLE......IIHRD......CDFGW......RVDIW......
145484890	......KLHRK......IVKLY......YLVLE......IIHRD......CDFGW......RIDIW......
145485578	......KVQME......IVKLY......YLLME......VMHRD......GDFGC......RVDAW......
145488418	......DIQSS......IVKIF......YLITE......IMHRD......GDFGF......RVDIW......
145487902	......KVHLK......INNLY......YMVLE......VFHRD......CDFGW......KIDIW......
145490162	......KIMYG......IVKLY......YLILE......IIHRD......ADFGW......KIDNW......
145486435	......QIQSS......VVKVF......YIILE......LVHRD......SDFGW......KVDSW......
145488424	......KIQRK......IIDLD......YLILE......IIHRD......CDFGW......KLDIW......
145489408	......KIQRK......ITKLF......FLILE......IIHRD......CDFGW......TLDIW......
145490686	......DIQSK......IVQLF......YLVTE......IMHRD......GDFGC......RVDIW......
145494133	......QIQSS......VVKVF......YIILE......LVHRD......SDFGW......KVDSW......
145491113	......RIQRK......ITQLY......YLILE......IIHRD......CDFGW......TLDIW......
145490692	......KIQRK......IIKLD......YLVLE......IIHRD......CDFGW......KLDIW......
145491883	......EILSH......IIGFY......YLMLE......VIHRD......SDFGW......RVDVW......
145494846	......KIQMY......ILKCF......ALILE......IIHRD......ADLGI......KVDCY......
145499837	......EILSH......IIGFY......YLMLE......VIHRD......SDFGW......RVDVW......
145496593	......KVHLK......IVNLY......YMVLE......VFHRD......CDFGW......KIDIW......
145504402	......KVHLK......IVNLY......YMVLE......VFHRD......CDFGW......KIDIW......
145503676	......RIQRK......ITQLF......YLILE......IIHRD......CDFGW......TLDIW......
145500478	......RVHKK......IVQLY......FIVIE......IFHRD......CDFGW......KIDLW......
145506685	......AVQQM......ILQFV......CLVLE......IIHRD......SDFGW......SIDLW......
145506068	......KIQRR......IVKLY......YLILE......VLHRD......CDFGW......KLDIW......
145505696	......KVHLK......IVNLY......YMVLE......VFHRD......CDFGW......RIDIW......
145508752	......VIQSK......IVKMF......YMMLE......FMHRD......CDFGC......RCDAW......
145510851	......KIMYG......IVKLY......YLILE......IIHRD......ADFGW......KIDHW......
145513000	......KIQGY......ILKLY......VLILE......IIHRD......ADMGL......TVDLW......
145513392	......KIQSY......ILKLY......VLILE......VIHRD......ADFGL......SVDLW......
145516412	......RIQKK......VIQLF......YLVLE......IIHRD......CDFGW......TLDMW......
145514528	......KTQMV......ILKCY......ALILE......IIHRD......ADLGI......KVDCY......
145515377	......KIQRK......ICKLH......YLILE......IIHRD......CDFGW......SLDVW......
145515501	......KIQRR......IVRLH......YLILE......ILHRD......CDFGW......KLDIW......
145519479	......RIQKK......VIQLF......YLILE......IIHRD......CDFGW......SLDMW......
145519996	......KIQRR......ITKLF......FLILE......IIHRD......CDFGW......TLDIW......
145517995	......QIHKK......IIKLH......HMLME......IIHRD......CDFGW......KVDTW......
145519720	......EILTH......IISFY......YLMLE......VIHRD......SDFGW......RVDVW......
145522614	......RIQRK......ITQLY......YLILE......IIHRD......CDFGW......TLDVW......
145524713	......RIQKR......VIQLF......YLILE......IIHRD......CDFGW......TLDMW......
145525074	......TIQQM......IIQLI......CLVME......IIHRD......SDFGW......SIDLW......
145526809	......RVHKK......IVHLY......FMVLE......VFHRD......CDFGW......KIDLW......
145529279	......KIMYQ......IMKLI......YLILE......ILHRD......ADFGS......CVDIW......
145530311	......KIQRR......ITKLF......FLILE......IIHRD......CDFGW......SLDIW......
145528776	......KIQYL......ITKLY......VLVLE......VIHRD......ADFSF......KSDIW......
145530271	......MKQYS......MVDFI......YFVCE......IAHLD......TDFAT......EADLW......
145534131	......KIQGY......ILKLF......VLILE......IIHRD......ADMGL......AVDLW......
145531982	......KIQYL......ITKLY......VLVLE......VIHRD......ADFSF......KSDIW......
145532274	......KIQYL......IVKLY......VLVLE......IIHRD......ADFTY......STDMW......
145531767	......EILTH......IISFY......YLMLE......VIHRD......SDFGW......RVDVW......
145533773	......KIQSY......ILKHY......VLILE......LLKLD......ADFGL......SVDLW......
145538401	......RIQRK......ITQLF......YLILE......IIHRD......CDFGW......TLDVW......
145535482	......TIQSK......IVKMF......YMMLE......FMHRD......CDLGC......RCDAW......
145536143	......GIQSK......IVKMY......YMMLE......YMHRD......CDLGC......RCDAW......
145541335	......KIQRK......ITKLF......FLILE......IIHRD......CDFGW......SLDIW......
145541592	......KLQRS......IIQLY......YFALE......VIHRD......CDFGW......RVDIW......
145542372	......KIQRK......ICKLY......YLVLE......VIHRD......CDFGW......SLDVW......
145541718	......LIQSK......IVKMY......YMMLE......YMHRD......CDLGC......RCDAW......
145539552	......KLQGY......ILKLY......VLILE......IIHRD......ADMGL......TVDIW......
145540327	......KIQYL......IVKLY......VLVLE......IIHRD......ADFTH......STDMW......

145538951	......KIQRK......ICKLH......YLILE......IIHRD......CDFGW......SLDVW......
145539936	......KIQSY......ILKLF......VLILE......VIHRD......ADFGL......SVDLW......
145547801	......KIQYL......ITKLY......VLVLE......VIHRD......ADFSF......KSDIW......
145547501	......KIQYL......ITRLY......VLVLE......VIHRD......ADFSF......KIDIW......
145543693	......KIQYL......IVKLY......VLVLE......IIHRD......ADFTH......STDMW......
145550716	......EIIEE......LPYLY......FLFLK......VIHRD......IDFDR......TYDIW......
145473801	......KIQYL......IVKLY......VLVLE......IIHRD......ADFTY......STDMW......
145548884	......KVQMK......IVKLY......YLLME......VMHRD......GDFGC......RVDAW......
125524746	......EIQSH......ILRLY......YLILE......VIHRD......ADFGW......HVDIW......
125545824	......EIQHG......VLRLF......VLVLE......VIHRD......ADFGW......AVDNW......
125834231	......EIQSH......ILRFY......FLILE......VIHRD......ADFGW......KVDLW......
126644226	......EIQAH......ILQLY......WLVIE......VIHRD......ADFGW......EVDIW......
126303208	......EIQSH......ILRLY......YLILE......VIHRD......ADFGW......KVDLW......
126309136	......EIQSH......ILRLY......YLILE......VIHRD......ADFGW......KVDLW......
126541214	......EIQSH......ILRLY......YLILE......VIHRD......ADFGW......KVDLW......
154341587	......ALQRL......VVRLY......YFVLE......VVLRD......LDFGA......SSDLW......
154341064	......NLQRT......IVRLY......NLVLE......VAHRD......ADFGW......KTDVW......
154340259	......EIAFN......LLRTY......YLILE......ILHRD......ADFGW......TADLW......
145235872	......EIQSN......VLRLY......FLILE......VMHRD......SDFGW......KVDLW......
145243962	......EIQSN......ILRLY......ILILE......VIHRD......SDFGW......KVDLW......
146091668	......EIAFN......LLRTY......YLILE......ILHRD......ADFGW......SADLW......
144226857	......EIQSH......ILRMY......YLMLE......VIHRD......ADFGW......KVDLW......
141521454	......EIQSH......ILRLY......YLILE......VIHRD......ADFGW......KVDLW......
145351480	......EIQSH......ILRLY......FLILE......VIHRD......ADFGW......AVDVW......
145353320	......AVHHS......IVGFF......YLIHE......VIHRD......TDFGF......AVDLW......
145612715	......EIQSN......ILKMY......FIILE......VMHRD......SDFGW......RVDLW......
146182201	......EIQSH......IIECY......FIILE......VLHRD......GDFGC......EADVW......
146172296	......EIQSH......VLRMF......YLILE......IIHRD......ADFGW......TVDIW......
146179789	......KIMYS......ILKLY......YLVLE......IIHRD......ADFGW......GVDIW......
146170354	......KVMYS......IIKLY......YLMIE......IIHRD......VDFGW......KLDIW......
146161563	......KIQKR......ICKLF......YLVLE......IIHRD......CDFGW......RIDIW......
146161565	......KIQKR......ICKLY......YLVLE......IIHRD......CDFGW......RVDIW......
149236359	......EIQSA......ITKLY......YLVLE......IIHRD......SDFGW......KVDVW......
158513308	......EIQSH......ILRLY......YLILE......VIHRD......ADFGW......KVDLW......
147781807	......EIQTS......VLRLY......FLILE......VIHRD......ADFGW......AVDNW......
147799032	......EIQSH......ILRLY......YLILE......VIHRD......ADFGW......SVDIW......
148670472	......EIQAH......ILRLY......YLILE......VIHRD......SDFGW......MVDLW......
148699387	......EIQAH......ILRLY......YLILE......VIHRD......SDFGW......MVDLW......
156094149	......IIQAS......ILQLI......FLILE......IIHRD......ADFGF......NVDLW......
156098641	......HTMTK......VIKLI......YLLYE......VIHRD......IDFGT......ARDLW......
156100769	......ELQAH......IACMY......FFVME......IAHLD......ADFGL......KTDIW......
149252375	......EIQAH......ILRLY......YLILE......VIHRD......SDFGW......MVDLW......
150865015	......EIQSN......ISRLY......YLILE......VIHRD......SDFGW......YVDIW......
149427594	......EIQSH......ILRLY......YLILE......VIHRD......ADFGW......KVDLW......
149722524	......EIQAH......ILRLY......YLILE......VIHRD......ADFGW......KVDLW......
149724949	......EIQAH......ILRLY......YLILE......VIHRD......ADFGW......KVDLW......
149734120	......EIQSH......ILRLY......YLILE......VIHRD......ADFGW......KVDLW......
154274327	......EIQSN......ILRLY......FLILE......IMHRD......SDFGW......KIDLW......
154290083	......EIQSN......ILQLY......FLILE......VMHRD......ADFGW......KVDLW......
108743416	......RITAA......VVTVH......FLVME......VLHRD......LDFGI......RSDLY......
157103645	......KTLAH......IVRYF......YIQMQ......LIHRD......GDFGL......KVDIF......
157111600	......KTLAS......IVPYK......-----......IVHHD......GDFGL......KSDIY......
157111534	......ELLSR......VVRYY......YIQME......MIHRD......GDFGL......KVDLY......
109065908	......KVLAG......IVGYH......HIQMQ......IVHRD......GDFGL......KSDMY......
109103772	......KALAK......IVRYF......YIQMQ......LMHRD......GDFGL......KVDIF......
109103770	......KALAK......IVRYF......YIQMQ......LMHRD......GDFGL......KVDIF......
108802827	......KALAA......IVQYH......FIQME......LIHRD......GDFGL......EVDIF......
121593051	......RLLAQ......LVKVF......YMVMP......TLHRD......LDLGA......WSDLY......
110763777	......KLLSR......VVRYY......YIQME......MIHRD......GDFGL......KVDIY......
110764839	......KALAK......IVRYF......YIQMQ......LIHRD......GDFGL......KIDIY......
118405094	......EALAH......IVRYN......FIQME......LIHRD......GDFGL......EVDIF......
77176927	......KALAT......IVRYY......FIQME......LIHRD......GDFGL......KTDIF......
115438072	......MLLSR......VVRYY......YIQME......IIHRD......GDFGL......KVDMF......
115397819	......QLLQH......LVSYR......FILQQ......YIHRD......SDFGE......KSDIF......

114578712	......KALAK......IVRYF......YIQMQ......LMHRD......GDFGL......KVDIF......
114576950	......KALAK......IVHYN......FIQME......LIHRD......GDFGL......EVDLY......
114576948	......KALAK......IVHYN......FIQME......LIHRD......GDFGL......EVDLY......
114578710	......KALAK......IVRYF......YIQMQ......LMHRD......GDFGL......KVDIF......
114612041	......KVLAG......IVGYH......HIQMQ......IVHRD......GDFGL......MSDMY......
114656310	......TLLSR......IVRYY......YIQME......MIHRD......GDFGL......KVDLF......
114656306	......TLLSR......IVRYY......--QME......MIHRD......GDFGL......KVDLF......
114656312	......TLLSR......IVRYY......YIQME......MIHRD......GDFGL......KVDLF......
114656304	......TLLSR......IVRYY......YIQME......MIHRD......GDFGL......KVDLF......
114656308	......TLLSR......IVRYY......YIQME......MIHRD......GDFGL......KVDLF......
115373822	......QAMAR......VVAYD......FIAME......LVHRD......TDFGV......RSDLF......
115372076	......KLTVS......VLQVF......YMVME......IIHRD......ADFGI......RADLF......
115746620	......KLLSR......VVRYY......YIQME......MIHRD......GDFGL......KVDLY......
115748985	......KLLSR......VVRYY......YIQME......MIHRD......GDFGL......KVDLY......
115889637	......RTLAI......IVRYF......YIQMQ......MIHRD......GDFGL......KVDIF......
115950029	......RTLAI......IVRYF......YIQMQ......MIHRD......GDFGL......KVDIF......
116309697	......KIMSC......VVSFY......IILME......IVHRD......ADFGH......KTDIF......
116310742	......ATLSR......VVRYY......YIQME......IIHRD......GDFGL......KVDMY......
116497041	......KALAK......IVRYF......YIQMQ......LMHRD......GDFGL......KVDIF......
169842990	......NALSR......IVRYY......YIQME......IIHRD......GDFGL......KADMY......
117935051	......KALAT......IVHYH......FIQME......LIHRD......GDFGL......EVDIY......
117645376	......KVLAG......IVGYH......HIQMQ......IVHRD......GDFGL......KSDMY......
118090781	......KALAK......IVRYF......YIQMQ......LMHRD......GDFGL......KVDIF......
118091755	......TLLSR......IVRYY......YIQME......MIHRD......GDFGL......KVDLF......
121707280	......QLLQH......LVSYR......FILQQ......YIHRD......SDFGE......KSDIF......
121712796	......MLLSR......VVRYF......FIQME......IIHRD......GDFGL......KVDMY......
119498409	......MLLSR......VVRYF......YIQME......IIHRD......GDFGL......KVDMY......
119500746	......QLLQH......LVSYR......FILQQ......YIHRD......SDFGE......KSDIF......
119597475	......KALAK......IVRYF......YIQMQ......LMHRD......GDFGL......KVDIF......
147904593	......KALAK......IVRYF......YIQMQ......LMHRD......GDFGL......KVDIF......
147906536	......EALAH......IVRYH......FIGME......LIHRD......GDFGL......EVDIF......
153006398	......EAAAR......VVTLH......FLILE......VVHRD......LDFGL......RADVF......
153004413	......EAAAR......IAALH......YLVYE......VVHRD......LDFGI......RTDLY......
153007011	......EAAAR......IVTVY......YLVLE......VVHRD......LDFGL......RTDVY......
123401100	......AFLSK......IVNFY......FIQME......VIHRD......GDFGL......KSDMY......
123437775	......KIMAK......VVRYY......YIQME......IVHRD......ADFGI......KSDIY......
123469556	......QCLAA......IVRYF......YIQMQ......IIHRD......IDFGI......SDDVY......
166851838	......TLLSR......IVRYY......YIQME......MIHRD......GDFGL......KVDLF......
123235427	......TLLSR......IVRYY......YIQME......MIHRD......GDFGL......KVDLF......
123235426	......TLLSR......IVRYY......YIQME......MIHRD......GDFGL......KVDLF......
123235425	......TLLSR......IVRYY......YIQME......MIHRD......GDFGL......KVDLF......
124001564	......KALAK......IVRYF......YIQMQ......LMHRD......GDFGL......KVDIF......
145482531	......DLLSH......VIRYY......EIQLQ......MVHRD......GDFGL......KRDVF......
145483841	......NGLQE......IVRYY......FIEME......FIHRD......GVLGL......AADIY......
145482159	......KAMLR......VIRLY......YLQQE......FFHRD......CDFDW......KAELF......
145482973	......KILSK......ILRLF......YMQLE......IIHRD......ADFDL......ADEFY......
145488944	......KAMLR......VIRLY......YQQLE......FFHRD......CDFSH......REELF......
145498148	......VLLSR......IVRYY......YIQME......IIHRD......GDFGL......KVDIY......
145501329	......VLLSR......IVRYY......YIQME......IIHRD......GDFGL......KVDIY......
145516721	......KYLAR......IIRYF......YIQTE......LVHRD......GDFGL......KSDIY......
145520589	......GSLQE......VVRYY......YIEME......YIHRD......GDLGL......AADIY......
145526306	......KYLAR......IIRYF......YIQTE......LVHRD......GDFGL......KSDIY......
145550535	......RYLAQ......IMRYY......-VQME......LVHRD......GDFGL......RIDIY......
145548221	......GSLQE......IVRYY......YIEME......YIHRD......GDLGL......AADIY......
145551284	......KILSK......ILRLF......YMQLE......IIHRD......ADFDL......ADEFY......
125548839	......ATLSR......VVRYY......YIQME......IIHRD......GDFGL......KVDMY......
126643929	......TANRT......VVRYY......MIQME......IVHRD......GDFGL......KADIY......
126278352	......TLLSR......IVRYY......YIQME......MIHRD......GDFGL......KVDLF......
126305316	......KALAK......IVRYF......YIQMQ......LMHRD......GDFGL......KVDIF......
126735340	......RSLAR......IVSVH......YMVLD......LLHRD......IDFGA......CSDLY......
145257459	......QLLQH......LVSYR......FILQQ......YIHRD......SDFGE......KSDIF......
154336423	......VLHSS......VVRFY......STRLQ......VVHRD......GDFGL......PSDIF......
154341679	......RVHAM......VVRYH......FLQME......ILHRD......GDFGL......ASDAF......
154335772	......QTLSA......IVRYY......YIQME......IVHRD......ADFGL......KVDEF......
146099103	......TSLAY......MVSTS......FIQME......VVHHD......GDFGL......PSDIF......
146079612	......QTLAA......IVRYY......YIQME......IVHRD......ADFGL......KVDEF......
146093786	......RIHAM......LVRYH......FLQME......ILHRD......GDLGL......ASDAF......

145253605	......MLLSR......VVRYY......FIQME......IIHRD......GDFGL......KVDMY......
134304838	......KALAK......IVRYF......YIQMQ......LMHRD......GDFGL......KVDIF......
145301625	......KALAK......IVHYN......FIQME......IIHRD......GDFGL......EVDLY......
134105965	......KALAK......IVHYN......FIQME......IIHRD......GDFGL......EVDLY......
145601757	......SVFCR......VVKYV......YLVME......IIHRD......GDFGL......KCDMY......
146165413	......KSIMS......IVRYH......YIQLE......IIHRD......ADFGL......KIDVY......
146419898	......LILYE......LIRYN......FILQQ......ILHRD......SDFGE......EVDIY......
146414982	......LSLAR......IVRYY......YIQME......FIHRD......GDFGL......KVDMY......
149234740	......LSLAR......IVRYY......YIQME......FIHRD......GDFGL......KVDMF......
148695960	......TLLSR......IVRYY......YIQME......MIHRD......GDFGL......KVDLF......
156100439	......IMIAK......IVRYY......YILME......IMHRD......GDFGL......KADIF......
149177112	......RSAAG......VCTIY......TIVME......IVHGD......VDFGL......ASDIF......
149173262	......VVTGE......IVPIY......FYSMK......VVHRD......MDWGL......LSDIY......
149050635	......QALAE......IVQYR......FIQME......LIHRD......GDFGL......EVDIF......
150865907	......LSLAR......IVRYY......YIQME......FIHRD......GDFGL......KVDMY......
150951521	......LILYE......LIRYN......FILQQ......ILHRD......SDFGE......ESDIY......
149570328	......TLLSR......IVRYY......YIQME......MIHRD......GDFGL......KVDLF......
149640638	......KALAK......IVRYF......YIQMQ......LMHRD......GDFGL......KVDIF......
149692490	......TLLSR......IVRYY......YIQME......MIHRD......GDFGL......KVDLF......
149755405	......KVLAG......IVGYH......HIQMQ......IVHRD......GDFGL......KSDMY......
149921927	......KLLAQ......IVRIH......WLVLE......MAHRD......LDFGL......KGDQF......
149924660	......RALAR......VVQVY......FVAME......LIHRD......ADFGL......RSDQF......
149922668	......QALAR......VVQIH......FVAME......LVHRD......LDFGL......LSDQF......
149922448	......KAMAQ......VITVH......FIAME......LVHRD......LDFGL......RSDQF......
149924029	......KGLAR......VVQVY......FIAME......LVHRD......LDFGL......RSDQF......
149922268	......QALAQ......VVAVH......FVAME......LVHRD......LDFGI......RSDQF......
149924429	......QALAR......VVAVH......FVAME......LVHRD......LDFGD......RSDQF......
149923601	......QALAQ......VIAVH......FIAME......LIHRD......LDFGL......RSDLF......
149918957	......RAIAR......VVHVH......ILALE......VIHRD......VDFGL......LCDQY......
149916611	......KALAR......VVDIY......YIAME......LVHRD......LDFGL......LTDQF......
149921577	......QAMAR......VVHIH......FLAME......LVHRD......TDFGV......RSDQF......
149920201	......RVLAK......IIRVF......FVVME......VLHGD......ADFGV......RSDLY......
149921353	......QALAR......IVPVH......FIVME......VVHRD......LDFGL......EADQF......
149920426	......QITGQ......IVPVH......FFMMK......VIHLD......MDWGI......RTDVF......
149920924	......MALAR......VVQIY......FLAME......LVHRD......LDFGL......ASDQF......
149920131	......QALAK......VVQVH......YIAME......LVHRD......LDFGL......AADQY......
149917940	......QALAK......VVQVY......FMAME......LVHRD......LDFGL......RADQF......
149918493	......RAIAR......VVAVF......YVVME......VVHRD......LDFGL......ASDQY......
149917668	......KALAR......VVPIY......FIAME......LVHRD......MDFGL......RTDQF......
149920531	......RALAR......VVTIH......FIAME......ILHRD......ADFGL......KSDQY......
154273603	......QLLQH......LVSYR......FILQQ......FIHRD......SDFGE......KSDIF......
154276106	......RTLAR......VVRYY......-----......IVHRD......GDFGL......EVDVF......
154321936	......QLLQG......LVSYR......YILQQ......YIHRD......SDFGE......KSDIF......
154311379	......RLLSQ......VVRYF......YISME......FVHRD......GDLGL......KVDMY......
154317296	......KALAE......IVNYY......-----......LIHRD......GDLGL......KLDVY......
157135771	......TIHKS......IVGFH......YIVLE......IIHRD......GDFGL......EVDVW......
157135769	......TIHKS......IVGFH......YIVLE......IIHRD......GDFGL......EVDVW......
157123022	......SIHSQ......ILELY......YLVLE......ILHRD......SDFGL......PADVW......
109003626	......ELHRD......IVHFS......YIFLE......ILHRD......GDFGL......EADVW......
109075611	......KIHCQ......ILELY......YLVLE......ILHRD......ADFGL......ESDVW......
109077303	......ELHRI......VVQFY......YILLE......ILHRD......GDFGL......ESDIW......
109077307	......ELHRI......VVQFY......YILLE......ILHRD......GDFGL......ESDIW......
109103744	......SIHRS......VVGFH......FVVLE......VIHRD......GDFGL......EVDVW......
109122796	......ALHSR......IVAFH......YMVLE......IVHRD......GDLGL......QSDIW......
109475363	......ELHRD......IVRFS......YIFLE......ILHRD......GDFGL......EADVW......
109477078	......ELHRD......IVRFS......YIFLE......ILHRD......GDFGL......EADVW......
109480237	......ALHSR......IVAFH......YMVLE......IVHRD......GDLGL......KSDIW......
110751117	......AIHSK......VLELY......YLVLE......ILHRD......ADFGL......EADVW......
114107975	......DLHRE......VVKFS......YIFLE......ILHRD......GDFGL......ESDIW......
114600160	......ELHRI......VVQFY......YILLE......ILHRD......GDFGL......ESDIW......
114600162	......ELHRI......VVQFY......YILLE......ILHRD......GDFGL......ESDIW......
114600156	......ELHRI......VVQFY......YILLE......ILHRD......GDFGL......ESDIW......
114595982	......KIHCQ......ILELY......YLVLE......ILHRD......ADFGL......ESDVW......
114661622	......SIHRS......VVGFH......FVVLE......VIHRD......GDFGL......EVDVW......
115694606	......EIHCQ......ILELY......YLVLE......ILHRD......ADFGL......QSDVW......
116057051	......EIHAR......VVRFE......YILME......IVHRD......GDFGL......EVDTW......
118783668	......TIHRS......IVGFH......YIVLE......IIHRD......GDFGL......EVDIW......
169850748	......KIHRS......IVTFI......YITLE......VIHRD......GDFGL......EVDIW......

118094529	......ELHRD......IVKFS......YIFLE......ILHRD......GDFGL......ESDVW......
118103791	......ELHRM......VVQFY......YILLE......ILHRD......GDFGL......ESDIW......
121719952	......QIHSK......IVEFH......YVILE......VAHRD......GDFGL......KVDIW......
119484938	......QIHSK......IVEFH......YVVLE......VAHRD......GDFGL......KVDIW......
119895446	......ALHSR......IVAFH......YMVLE......IVHRD......GDLGL......QSDIW......
119913077	......ELHRI......VVQFY......YILLE......ILHRD......GDFGL......ESDIW......
123390085	......KLHRS......IVKLY......YIILE......IVHRD......CDFGL......KIDIW......
123419270	......QIQSA......VLRSY......YIVTE......VIHRD......ADFGL......EVDIW......
123424398	......AIQAS......ILRSY......YIVTE......VIHRD......ADFGL......EVDIW......
123466309	......DIHKS......IVEFK......YILLE......VIHRD......ADFGL......SVDIW......
123458255	......KIQSS......VVKAY......YIITE......IIHRD......ADFGL......EVDIW......
123478423	......SIQSS......VLRSF......YILLE......IIHRD......ADFGL......EVDVW......
154415222	......SIQAS......ILRSY......YILLE......IIHRD......ADFGL......EVDIW......
123122068	......ELHRD......IVRFS......YIFLE......ILHRD......GDFGL......EADVW......
145477567	......KIHKS......IVQFI......YIILE......IIHRD......GDFGL......EVDIW......
145478727	......KLHKS......IVQFE......YILLE......VLHRD......GDFGL......QADIW......
145476965	......TIHQE......IVHLI......YLIME......IIHRD......ADFGL......KVDLW......
145484499	......KIHKS......IVQFI......YIILE......IIHRD......GDFGL......EVDIW......
145494320	......KIHKS......VVQFE......YILLE......VIHRD......GDFGL......EVDVW......
145493730	......AIHLE......VVHLA......YLIME......IIHRD......ADFGL......KVDLW......
145497230	......RIHQQ......ILKYE......YILLE......ILHRD......GGFNY......EVDLW......
145497212	......KIHKS......IVQFE......YILLE......VIHRD......GDFGL......EVDVW......
145502293	......TIHSE......IVHLV......YLIME......IIHRD......ADFGL......KVDLW......
145508233	......KIHRS......IVGFH......YILLE......VIHRD......GDFGL......EVDIW......
145522910	......KIHKS......VVQFE......YILLE......VIHRD......GDFG-......EVDVW......
145530902	......KLHKS......IVQFE......YILLE......VLHRD......GDFGL......QADIW......
145542027	......KIHKS......IVQFE......YILLE......VIHRD......GDFGL......EVDVW......
145538574	......VIHSE......IVNLI......YLIME......IIHRD......ADFGL......KVDLW......
145552114	......KIHKS......IVQFE......YILLE......VIHRD......GDFGL......EVDIW......
145548361	......KIHRS......IVGFN......YILLE......VIHRD......GDFGL......EVDIW......
169646793	......EIQCR......VLELY......YLVLE......IMHRD......ADFGL......ESDVW......
134085942	......KIHCQ......ILELY......YLVLE......ILHRD......ADFGL......ESDIW......
126323633	......ELHSQ......VVGFH......YLVLE......IIHRD......GDLGL......QSDIW......
126330594	......KIHCQ......ILELY......YLVLE......ILHRD......ADFGI......ETDVW......
126334963	......NIHKS......VVGFH......FVVLE......VIHRD......GDFGL......EVDVW......
134034992	......ALHSR......IVAFH......YMVLE......ILHRD......GDLGL......Q-DIW......
154335192	......SIHRR......VVEFL......YMLLE......VIHRD......GDFGL......EVDTW......
146083432	......SIHRR......VVEFL......YMLLE......VIHRD......GDFGL......EVDTW......
145250947	......QIHSK......IVGFH......YVILE......VAHRD......GDFGL......KVDIW......
156718006	......ELHRT......VVQFY......YILLE......ILHRD......GDFGL......ESDIW......
145345884	......RIHKS......VVRFE......YILME......VIHRD......GDFGL......EVDTW......
145605303	......A----......IVQFY......YLVLE......IIHRD......GDFGL......NVDIW......
146387057	......SIHRS......VVGFH......FVVLE......VIHRD......GDFGL......EVDVW......
146415154	......KIHKS......IVNFV......YILLE......VIHRD......GDFGL......EVDIW......
149243893	......KIHKS......IVNFV......YILLE......VIHRD......GDFGL......EVDIW......
148685326	......SIHRS......VVGFH......FVVLE......VIHRD......GDFGL......EVDVW......
148703194	......KIHCQ......VLELY......YLVLE......ILHRD......ADFGL......ESDIW......
148703197	......KIHCQ......VLELY......YLVLE......ILHRD......ADFGL......ESDIW......
148698617	......ELHRD......IVRFS......YIFLE......ILHRD......GDFGL......EADVW......
149773496	......ELHRT......IVHFY......YILLE......ILHRD......GDFGL......ESDVW......
149034550	......ALHSR......IVAFH......YMVLE......IVHRD......GDLGL......KSDIW......
149035546	......ELHRD......IVRFS......YIFLE......ILHRD......GDFGL......EADVW......
149048793	......KIHCQ......VLELY......YLVLE......ILHRD......ADFGL......ESDIW......
149048790	......KIHCQ......VLELY......YLVLE......ILHRD......ADFGL......ESDIW......
149048791	......KIHCQ......VLELY......YLVLE......ILHRD......ADFGL......ESDIW......
150866257	......KIHKS......IVNFI......YILLE......VIHRD......GDFGL......EVDVW......
149633975	......ELHSC......VVGFH......YMVLE......IIHRD......GDLGL......QSDVW......
149639460	......ELHRI......VVQFY......YILLE......ILHRD......GDFGL......ESDIW......
154323294	......GLMAL......-----......---ME......IIHRD......GDFGL......AVDIW......
115455355	......NIHKT......IVRLW......CTVLE......IIHYD......TDFGL......KVDVW......
157104596	......NIHKA......VVKLY......CTVLE......IIHYD......TDFGL......KVDVW......
109100047	......RIHKE......IVKLY......CTVLE......IIHYD......TDFGL......KVDVW......
109116514	......RIHKE......IVKLY......CTVLE......IIHYD......TDFGL......KVDVW......
109150068	......RIHKE......IVKLY......CTVLE......IIHYD......TDFGL......KVDVW......
109489161	......RIHKE......IVKLY......CTVLE......IIHYD......TDFGL......KVDVW......
109492057	......RIHKE......IVKLY......CTVLE......IIHYD......TDFGL......KVDVW......
110755587	......NIHKA......VVKLY......CTVLE......VIHYD......TDFGL......KVDVW......
114581665	......RIHKE......IVKLY......CTVLE......IIHYD......TDFGL......KVDVW......
114581667	......RIHKE......IVKLY......CTVLE......IIHYD......TDFGL......KVDVW......
114669802	......RIHKE......IVKLY......CTVLE......IIHYD......TDFGL......KVDVW......

```
118404426    ......RIHKE......IVKLY......CTVLE......IIHYD......TDFGL......KVDVW......
115608283    ......EIHKS......VVKLY......CTVLE......IIHYD......TDFGL......KVDVW......
115646482    ......NIHKA......VVKLY......CTVLE......VIHYD......TDFGL......KVDVW......
116061052    ......SIHKA......VVRLI......CTVLE......IIHYD......TDFGL......KVDTW......
117616794    ......RIHKE......IVKLY......CTVLE......IIHYD......TDFGL......KVDVW......
118093645    ......RIHKE......IVKLY......CTVLE......IIHYD......TDFGL......KVDVW......
118102856    ......RIHKE......IVKLY......CTVLE......IIHYD......TDFGL......KVDVW......
118102854    ......RIHKE......IVKLY......CTVLE......IIHYD......TDFGL......KVDVW......
119887575    ......RIHKE......IVKLY......CTVLE......IIHYD......TDFGL......KVDVW......
119614750    ......RIHKE......IVKLY......CTVLE......IIHYD......TDFGL......KVDVW......
162951881    ......RIHKE......IVKLY......CTVLE......IIHYD......TDFGL......KVDVW......
119614749    ......RIHKE......IVKLY......CTVLE......IIHYD......TDFGL......KVDVW......
119631625    ......RIHKE......IVKLY......CTVLE......IIHYD......TDFGL......KVDVW......
119912546    ......RIHKE......IVKLY......CTVLE......IIHYD......TDFGL......KVDVW......
145476047    ......DIHKR......IVSLY......CTVLE......IIHYD......TDFGL......KVDVW......
145498807    ......DIHKR......IVSLY......CTVLE......IIHYD......TDFGL......KVDVW......
145506969    ......RIHKQ......IISLF......CTILE......IIHYD......CDFGL......KVDIW......
145507758    ......RVHKE......IVKLY......CTVLE......IIHYD......SDFGL......KVDIW......
145505573    ......RVHKE......IVKLY......CTVLE......IIHYD......SDFGL......KVDIW......
145535890    ......KVHRE......IVKLY......CTVLE......IIHYD......SDFGL......KVDIW......
145538738    ......DIHKR......IVSLY......CTVLE......IIHYD......TDFGL......KVDVW......
125812101    ......RIHKE......IVKLY......CTVLE......IIHYD......TDFGL......KVDVW......
160333632    ......RIHKE......IVKLY......CTVLE......IIHYD......TDFGL......KVDVW......
125825471    ......RIHKQ......IVKLY......CTVLE......IIHYD......TDFGL......KVDVW......
126308371    ......RIHKE......IVKLY......CTVLE......IIHYD......TDFGL......KVDVW......
126308373    ......RIHKE......IVKLY......CTVLE......IIHYD......TDFGL......KVDVW......
126326566    ......RIHKE......IVKLY......CTVLE......IIHYD......TDFGL......KVDVW......
154342726    ......DIMRA......LTHLY......ISVME......IIHYD......TDFGL......KVDIW......
146095554    ......DIMRA......LTHLY......ISVME......IIHYD......TDFGL......KVDIW......
134085607    ......NIHKA......VVKLY......CTVLE......VIHYD......TDFGL......KVDVW......
145353556    ......DIHKS......VVRLI......CTVLE......IIHYD......TDFGL......KVDTW......
146182303    ......KIHSE......IVQLY......ATILE......IIHYD......SDFGL......KVDIW......
148695122    ......RIHKE......IVKLY......CTVLE......IIHYD......TDFGL......KVDVW......
148695123    ......RIHKE......IVKLY......CTVLE......IIHYD......TDFGL......KVDVW......
148702287    ......RIHKE......IVKLY......CTVLE......IIHYD......TDFGL......KVDVW......
149054514    ......RIHKE......IVKLY......CTVLE......IIHYD......TDFGL......KVDVW......
149249432    ......RIHKE......IVKLY......CTVLE......IIHYD......TDFGL......KVDVW......
149614066    ......RIHKE......IVKLY......CTVLE......IIHYD......TDFGL......KVDVW......
149639524    ......RIHKE......IVKLY......CTVLE......IIHYD......TDFGL......KVDVW......
149730720    ......RIHKE......IVKLY......CTVLE......IIHYD......TDFGL......KVDVW......
157128431    ......KLLAK......VVALH......FLVME......VIHLD......IDFGI......KSDIW......
169602953    ......DLLQK......VVRLY......VLVLM......IVHSD......IDFGI......PSDVW......
115384954    ......DLLKK......VVRLF......TLVLM......IVHSD......IDFGI......PSDVW......
114608216    ......AYLNK......IIRLY......YMVME......IVHSD......IDFGI......KSDVW......
116057782    ......KLLKT......IIQLI......FMVLE......IVHSD......IDFGI......SSDIW......
118404752    ......SHLNK......IIRLY......YMVME......IVHSD......IDFGI......KGDVW......
169858600    ......ALLKR......IIRLI......LLVME......IVHSD......IDFGI......PSDVW......
118088789    ......AHLSK......IIRLY......YMVME......IVHSD......IDFGI......KSDVW......
121705132    ......DLLKK......VVRLF......TLVLM......IVHSD......IDFGI......PSDVW......
119492529    ......DLLKK......VVRLF......TLVLM......IVHSD......IDFGI......PSDVW......
119901123    ......AYLNK......IIRLY......YMVME......IVHSD......IDFGI......KSDVW......
123448317    ......EILEQ......IIQLI......YLVQE......YCHTD......IDFGI......TTDVW......
123439298    ......EILQQ......IIHMY......YIVQE......ILHAD......IDFGI......PADIW......
125545426    ......EYLNK......IIQLI......YMVLE......IVHSD......IDFGI......PSDIW......
126310506    ......AYLSK......IIRLY......YMVME......IVHSD......IDFGI......KSDVW......
145233825    ......DLLKK......VVRLF......TLVLM......IVHSD......IDFGI......PSDVW......
145351583    ......KLLKT......IIQLV......FMVLE......IVHSD......IDFGI......ASDIW......
146422938    ......DLLTK......VVQLV......YLVME......IVHSD......IDFGI......PSDVW......
149235243    ......DLLLK......VVKLV......YLIME......IVHSD......IDFGI......PSDVW......
148694527    ......AFLNK......IIRLY......YMVME......IVHSD......IDFGI......RSDVW......
148694526    ......AFLNK......IIRLY......YMVME......IVHSD......IDFGI......RSDVW......
148694524    ......AFLNK......IIRLY......YMVME......IVHSD......IDFGI......RSDVW......
148694525    ......AFLNK......IIRLY......YMVME......IVHSD......IDFGI......RSDVW......
157823481    ......AYLNK......IIRLY......YMVME......IVHSD......IDFGI......RSDVW......
149019016    ......AYLNK......IIRLY......YMVME......IVHSD......IDFGI......RSDVW......
150866395    ......DLLLK......VVKLI......YLVME......IVHSD......IDFGI......PSDVW......
149638918    ......AHLKK......IIRLY......YMVME......IVHSD......IDFGI......KSDVW......
149723010    ......AYLNK......IIRLY......YMVME......IVHSD......IDFGI......KSDVW......
154279226    ......DLLKK......VVRLF......SVLME......VVHSD......IDFGI......PSDVW......
154308854    ......DLLKK......VIHLY......VLVLM......IVHSD......IDFGI......PSDIW......
115450393    ......AILST......ILRLI......YLILE......IVHRD......GDFGF......KADLW......
108796996    ......QNAAA......IVAVY......YIVME......IIHRD......MDFGI......RSDVY......
157117068    ......EIQRN......IIRML......IAVTE......ILHRD......CDFGF......HADLW......
157135348    ......SLMMK......IVRLQ......ILCME......ITHRD......TDLGY......SVDYW......
```

109081952	......EILKG.....IVQLK.....YLIME.....ISHLD.....ADFGF.....RVDLW.....
109090232	......QIMKK.....VVKAC.....ILAME.....IIHRD.....IDLGY.....TVDYW.....
109090234	......QIMKK.....VVKAC.....ILAME.....IIHRD.....IDLGY.....TVDYW.....
109090230	......QIMKK.....VVKAC.....ILAME.....IIHRD.....IDLGY.....TVDYW.....
109101008	......EIMRG.....IVHML.....VVVTD.....ILHRD.....CDFGF.....TADLW.....
109101010	......EIMRG.....IVHML.....VVVTD.....ILHRD.....CDFGF.....TADLW.....
109113681	......KILKE.....IVALY.....FLVME.....IIHRD.....ADFGF.....KADLW.....
109486156	......EIMRG.....IVHML.....VVVTD.....ILHRD.....CDFGF.....TADLW.....
109483554	......EILKG.....IVQLK.....YLIME.....ISHLD.....ADFGF.....RVDLW.....
109484108	......A----.....CLTYH.....WVVGS.....ILFCD.....SNFCL.....TSDLW.....
109485609	......A----.....CLTYH.....WLLEE.....ILFCD.....SNFCL.....TSDLW.....
109488256	......KILKE.....IVALY.....FLVME.....IIHRD.....ADFGF.....KADLW.....
109490959	......KILKE.....IVALY.....FLVME.....IIHRD.....ADFGF.....KADLW.....
110005239	......NSIAQ.....VVKVY.....CLVLE.....IIHRD.....ADFGV.....RSDIY.....
110798996	......TAIAN.....VVNVL.....YIVME.....IIHRD.....TDFGI.....RTDLY.....
110803942	......TAIAN.....VVNVL.....YIVME.....IIHRD.....TDFGI.....RTDLY.....
110736434	......EFLSS.....IIRLL.....VMVLE.....IIHRD.....ADFSL.....KADMW.....
110749150	......YLLKI.....IVEMR.....YIVME.....VSHMD.....GDFGF.....RVDLW.....
110761987	......EIQRR.....IVQML.....VVVTE.....VLHRD.....CDFGF.....NADLW.....
111023893	......RAAGQ.....VVNVL.....FIVTP.....ILHRD.....TDFGI.....AADVY.....
111019572	......HVAAR.....VIPIH.....YIDMR.....LVHRD.....VDFGI.....RADVY.....
111021257	......DHAAH.....IVTVH.....WIAMQ.....ILHRD.....TDFGT.....RVDIY.....
111020683	......QNAAA.....IVAVY.....YIVME.....IVHRD.....MDFGI.....RSDVY.....
111026927	......HAAAR.....VVPIH.....YIDMR.....LVHRD.....VDFGI.....RADIY.....
111026929	......EHAAR.....IVTVY.....WIAMQ.....ILHRD.....TDFGI.....RADVY.....
169597683	......GVHLR.....TVNLL.....YIVLE.....IYHRD.....GDFGL.....KADIW.....
111074982	......QAVAG.....IVNVY.....YIVME.....IIHRD.....TDFGI.....KSDIY.....
111226088	......QSSAA.....IVSVY.....YIVME.....IIHRD.....MDFGI.....RSDVY.....
111219769	......SVLLR.....VVRVR.....AIVMD.....IIHRD.....SDFGI.....PADIY.....
112491038	......RTAGR.....VVPIH.....YVDXR.....ATHRD.....VDFGI.....RADIY.....
150456432	......EILKG.....IVQLK.....YLIME.....ISHLD.....ADFGF.....RVDLW.....
114680063	......KVHQL.....FIKIY.....VIVMD.....FIHND.....CDYGL.....SFDWY.....
159900825	......EVWMR.....IVEAT.....HIVME.....LIHRD.....TDFGL.....TSDIY.....
159897230	......QLLQT.....LPKVV.....TLVME.....IIFRD.....IDFGV.....RADIY.....
159896868	......QTAAQ.....IARIF.....FFVQE.....YVHCD.....LDFGI.....ASDIY.....
159899989	......RAAAG.....IVPIY.....YIVMK.....VIHRD.....TDFGL.....ASDLY.....
159900462	......RLLAR.....LPRVY.....FLVME.....IIHRD.....IDFGL.....RSDIF.....
159896556	......RAIAT.....IVQIY.....YFVME.....VIHRD.....MDFGL.....QSDLY.....
115397145	......RILNE.....RVALH.....FHILS.....FCHRD.....TDFGF.....RSDIW.....
115397795	......HILKG.....IVALI.....HLVME.....LIHRD.....ADFGF.....KADLW.....
115401730	......KIMRD.....IVQYV.....YIIME.....ITHRD.....SDFGL.....SVDMW.....
114705226	......S-LKG.....MVEIL.....FLVMA.....LVHRD.....LDFGV.....TTEGW.....
114777821	......EIAGM.....IVTIF.....YIAME.....VIHRD.....TDFGI.....RSDLF.....
114586303	......RLTHE.....IVTFH.....WLVVE.....ILFCD.....SNFCL.....SSDLW.....
114586307	......RLTHE.....IVTFH.....WLVVE.....ILFCD.....SNFCL.....SSDLW.....
114632325	......QIMKK.....VVKAC.....ILAME.....IIHRD.....IDLGY.....TVDYW.....
114632323	......QIMKK.....VVKAC.....ILAME.....IIHRD.....IDLGY.....TVDYW.....
114632321	......QIMKK.....VVKAC.....ILAME.....IIHRD.....IDLGY.....TVDYW.....
114658114	......EILKG.....IVQLK.....YLIME.....ISHLD.....ADFGF.....RVDLW.....
114668677	......KILKE.....IVALY.....FLVME.....IIHRD.....ADFGF.....KADLW.....
126700197	......EAVAS.....IVNVY.....YIVME.....IIHRD.....ADFGI.....NADLY.....
115311891	......RLTHE.....IVTFH.....WLVVE.....ILFCD.....SNFCL.....TSDLW.....
115313788	......DIMRG.....IVLLL.....VVVTE.....ILHRD.....CDFGF.....STDLW.....
115380258	......RISVQ.....VVHIH.....FIAME.....IIHRD.....IDFGI.....RSDVF.....
115379535	......QLLSA.....VVRII.....CLVME.....IIHRD.....LDFGI.....RSDLY.....
115380264	......AIARR.....IIRVF.....ALVLE.....VIHKD.....ADFSI.....RADFY.....
115379292	......EVASR.....VITIF.....YYVME.....CVHRD.....LDFGL.....RSDLY.....
115380345	......RVSVG.....IVQVF.....FLAME.....LVHRD.....SDFGI.....RSDVY.....
115378742	......EIGRR.....AVAVL.....AIVME.....VIHKD.....IDFGL.....RADLY.....
115376999	......QLTSQ.....TIAIY.....YYAME.....MLHRD.....LDFGL.....RSDLY.....
115375181	......RLAAQ.....IVQIH.....FIAME.....IVHRD.....LDFGI.....RSDIF.....
115377229	......AVLEE.....LQPVL.....YLVTP.....VIHRD.....VDFGL.....TSDLY.....
115376001	......RIVNQ.....VVDIH.....YYVME.....IVHRD.....LDFGI.....NTDVY.....
115377552	......RAIAQ.....IVRVF.....WLIME.....VIHRD.....LDFGL.....RTDIW.....
115376341	......KAIAQ.....IVRIF.....FLIME.....IVHRD.....LDFGL.....RTDIW.....
115376948	......LIAAR.....IAQVY.....YIAME.....VVHRD.....VDFGI.....RADIF.....
115375391	......EALTR.....LVEVL.....YLVME.....VLHLD.....LDFGL.....QVDLY.....
115377726	......SLTSK.....TVTVI.....YFIME.....IVHRD.....LDFGL.....RSDVY.....
115372260	......RITAA.....IAQVY.....FLAME.....VIHRD.....LDFGI.....RSDLF.....
115373601	......RVVNA.....IIDIF.....YVVME.....VIHRD.....LDFGI.....TADLY.....
115373126	......RSVNQ.....IVEIF.....YCVME.....VVHRD.....LDFGV.....RADIY.....

```
115374090    ......RAQAR.....VCEMY.....FIAMQ.....LIHRD.....MDFGL.....RVDVY......
115373590    ......RAAAR.....VVTLY.....YLISE.....VLHRD.....LDFGL.....RSDVY......
115374074    ......KLARR.....VARVF.....FLTME.....VAHRD.....TDFGI.....RTDLY......
115372971    ......RATAR.....IVIIY.....FMVLE.....IVHRD.....LDFGI.....RTDIW......
115373811    ......RAQAR.....VCQVF.....FIAMQ.....LIHRD.....MDFGL.....RADVY......
115374960    ......ETLAE.....VVKVF.....YLVME.....LVHRD.....MDFGL.....RADLY......
115374417    ......KAIAR.....VVQVH.....YIVME.....IVHRD.....LDFGL.....RSDLW......
115372469    ......QALAR.....VIAVH.....FLAMD.....LVHRD.....TDFGL.....LSDQF......
115374149    ......KAASR.....SISIL.....FIAME.....VIHRD.....LDFGI.....RSDLY......
115373723    ......KISVQ.....IGQIF.....FIAME.....IVHRD.....IDFGI.....RSDVF......
116054435    ......RTLAR.....TVTIH.....YMAME.....LVHRD.....SDFGI.....RADLY......
115695318    ......EIMKE.....IIEML.....VAVTD.....ILHRD.....CDFGF.....TADLW......
115926775    ......EILKK.....IVELK.....YLIME.....ITHMD.....ADFGF.....KADLW......
115928320    ......RLTHE.....IVEFY.....WLIVE.....MLFSD.....DDFSL.....ATDLW......
116055167    ......LFSRA.....VLKIV.....WLVLE.....LIHCD.....RGFGL.....ATDLW......
116055704    ......AFLSS.....MCEFL.....WMVME.....VVHRD.....SDLGL.....TSDLY......
116055390    ......DIMRT.....VIKML.....VVVME.....VIHRD.....CDFGF.....TVDLW......
116333600    ......LAATE.....IVQVY.....YLVME.....IIHRD.....TDFGI.....QSDIY......
116328804    ......EILNY.....MIQAF.....TLILE.....VIHKD.....VDFGI.....RSDFY......
116331714    ......EILNY.....MIQAF.....TLILE.....VIHKD.....VDFGI.....RSDFY......
116621589    ......RAISS.....ICILH.....YFVME.....IIHRD.....LDFGL.....RTDVY......
116622280    ......RAVAA.....ICMLY.....YLVME.....IIHRD.....LDFGL.....RSDIF......
116622428    ......RAVAA.....IVAVF.....YMVSE.....IVHRD.....LDFGL.....RSDIF......
116622793    ......RAASA.....ILSVF.....YIVSE.....IVHRD.....LDFGL.....RSDIF......
145590211    ......TILRS.....VPKYL.....YIAME.....AIHLD.....IDFGL.....RSDIF......
169862747    ......QILKS.....ITKLI.....YLIME.....LIHRD.....ADFGF.....KADLW......
169864152    ......NLLKR.....ILNLI.....YLISD.....IIHRD.....IDFGL.....ETDWW......
116873255    ......QSATS.....IVSIY.....YIVME.....IIHRD.....TDFGI.....KSDIY......
118034466    ......RKAQV.....IVTVY.....YLTME.....FVHCD.....IDFGI.....RDDIY......
118048028    ......GLLAT.....IPKVY.....YLILE.....IVFRD.....IDFGI.....VGDIY......
118046368    ......EMLQD.....FPRYI.....YYVQS.....IVHGD.....VDFGL.....RSDLF......
118047071    ......--LAQ.....FLALR.....YLVAD.....LLLGD.....APFAC.....AVDIY......
146307338    ......KTLAR.....TVTIH.....YMAME.....LVHRD.....SDFGI.....RTDLY......
156740529    ......VLAAR.....IVPIY.....FIAMR.....VVHRD.....TDFGI.....RADVY......
170726231    ......WIGHR.....VVKLL.....YYVTE.....TLHQD.....IDFGS.....RGDLF......
118093761    ......EIMRG.....IIQML.....VMVTD.....ILHRD.....CDFGF.....TADLW......
118098424    ......KILKE.....IVALY.....YLVME.....IIHRD.....ADFGF.....KADLW......
118101148    ......SVLKR.....IIQVI.....FLVMK.....IAHRD.....TDFGC.....KADAW......
118444123    ......SSAAS.....IVGIY.....YIVME.....IIHRD.....TDFGI.....KTDIY......
118465657    ......RTAGR.....VVPVH.....FLEMR.....VMHRD.....VDFGI.....RADIY......
118462481    ......RIAAG.....VVPIH.....YVDMR.....LIHRD.....IDFGL.....RADVY......
118465473    ......DIAGR.....VVPIH.....FVEMR.....VTHRD.....ADFGI.....SVDIY......
118466237    ......RAVTR.....VLPLH.....FIDMQ.....LMHLD.....ADFGL.....QTDIY......
118466937    ......QNAAA.....IVAVY.....YIVME.....IIHRD.....MDFGI.....RSDVY......
118472763    ......RAAAA.....VVAVH.....YIVME.....IVHRD.....ADFGI.....ADDLY......
118471844    ......QNAAA.....IVAVY.....YIVME.....IIHRD.....MDFGI.....RSDVY......
118471062    ......DIAAG.....IVGLH.....WIAMD.....LLHRD.....ADFGV.....RADQY......
118472741    ......DLAAK.....IVGIH.....WISMD.....LLHRD.....GDFGV.....RADQY......
118473542    ......DVAAA.....IVEIH.....WIAMD.....LLHRD.....ADFGI.....RADQY......
118587053    ......QAAAQ.....IVKVL.....YLTME.....IVHRD.....VDFGI.....KSDIY......
118602682    ......WVGQR.....VVKIY.....AYAMV.....MLHCD.....IDFGS.....ASDQF......
118618539    ......RTAGR.....VVPIH.....YVDMR.....VTHRD.....VDFGI.....RADIY......
118615935    ......QNAAA.....IVAVY.....YIVME.....IIHRD.....MDFGI.....RSDVY......
151301204    ......K----.....IVTFH.....WLVVE.....ILFCD.....SNFCL.....SSDLW......
118726728    ......QAVAS.....VVSIY.....YIVME.....IVHRD.....TDFGI.....KSDLY......
118743900    ......EAAGT.....IVTIY.....YVAME.....IVHRD.....ADFGI.....RSDLF......
152997728    ......WIAKR.....VAKAI.....YLATE.....MIHQD.....IDFG-.....RSDIY......
157961638    ......WVGSR.....VVAVC.....YYLTE.....TLHQD.....VDFGF.....KADLY......
119026032    ......RSAAA.....IVQVY.....FLVME.....VVHRD.....TDFGL.....QGDLY......
121707316    ......HILKG.....IVALI.....HLVME.....LIHRD.....ADFGF.....KADLW......
119481853    ......KIMRD.....IVQYI.....YIIME.....ITHRD.....SDFGL.....SVDMW......
119500722    ......HILKG.....IVALI.....HLVME.....LIHRD.....ADFGF.....KADLW......
119493259    ......QVLYQ.....IPQFC.....FLVQD.....IIHRD.....IDFGV.....SSDLY......
119492101    ......RQLKL.....IPQLY.....YLVQE.....VIHRD.....VDFGA.....ASDIY......
119491229    ......RQLQQ.....IPTLY.....FLVQE.....VIHRD.....IDFGV.....ASDLF......
119490879    ......QILYQ.....IPCFR.....FVVQD.....VVHRD.....IDFGG.....HSDLY......
119487570    ......ALVAN.....VVRML.....MLVME.....IIHKD.....IDFSC.....RSDFY......
119489007    ......AILEK.....IPRLY.....YLVQE.....LIHRD.....IDFGA.....--DLY......
119487305    ......QITHN.....TIQAY.....AMILE.....IIHKD.....IDFGM.....RSDFY......
119513336    ......TISKN.....IIQPL.....ILVME.....IIHKD.....IDFSI.....RSDFY......
```

```
119511416   ......KVLYR......IPRLF......YLVQE......IIHRD......IDFGA......CSDVY......
119512149   ......IKLAR......IVRIY......CMVME......LLHRD......IDFGI......RADVY......
119511131   ......QQLQQ......IPALL......YLVQQ......IIHRD......IDFGA......ASDLY......
119509409   ......KVLYR......IPRLF......YLVQE......IIHRD......IDFGA......CSDVY......
119509882   ......QTLQE......IPKYI......YLVQE......IIHRD......VDFGA......ASDLY......
119512026   ......KVLES......IPTLY......YLVQQ......VIHRD......IDFGA......SSDLY......
119512025   ......IVLES......IPTLY......YLVQQ......VIHRD......IDFGA......SSDLY......
119510956   ......HYLSQ......IAKVY......YLVME......LLHRD......IDFGA......QTDFY......
119509881   ......KVLKQ......IPHYL......CLVQQ......IIHRD......VDFGA......-SDLY......
119512003   ......QLLHK......IPQLL......FLVQQ......VIHRD......IDFGA......SSDIY......
119511284   ......QILQQ......IPRYI......YLVQE......IIHRD......VDFDL......ASDLY......
119510305   ......KLLQE......IPTLL......YLVQQ......VIHRD......IDFGS......-SDLF......
119591056   ......EIMRG......IVHML......VVVTD......ILHRD......CDFGF......TADLW......
119585037   ......K----......IVTFH......WLVVE......ILFCD......SNFCL......SSDLW......
119585036   ......K----......IVTFH......WLVVE......ILFCD......SNFCL......SSDLW......
119585033   ......K----......IVTFH......WLVVE......ILFCD......SNFCL......SSDLW......
119585034   ......K----......IVTFH......WLVVE......ILFCD......SNFCL......SSDLW......
119585038   ......K----......IVTFH......WLVVE......ILFCD......SNFCL......SSDLW......
119585039   ......K----......IVTFH......WLVVE......ILFCD......SNFCL......SSDLW......
119591055   ......EIMRG......IVHML......VVVTD......ILHRD......CDFGF......TADLW......
119591054   ......EIMRG......IVHML......VVVTD......ILHRD......CDFGF......TADLW......
119591053   ......EIMRG......IVHML......VVVTD......ILHRD......CDFGF......TADLW......
119900063   ......IALAR......IANVF......YMVME......VVHRD......MDFGI......RSDLY......
119897279   ......ALAGK......IVQIF......YVVME......ITHRD......SDFGA......RTDIY......
119897189   ......RVAGT......IVTIF......FIAME......VVHQD......MDFGI......RSDLY......
119872609   ......QRYLE......VVKAY......YILLE......VVHLD......GDMGI......ASDIY......
159035727   ......QNAAS......IVAVY......FIVME......IIHRD......MDFGI......RSDVY......
119888432   ......EIMRG......IVHML......VVVTD......ILHRD......CDFGF......TADLW......
119964537   ......KVHQL......FIQIY......VIVMD......FVHND......CDYGL......SFDWY......
119619711   ......EILKG......IVQLK......YLIME......ISHLD......ADFGF......RVDLW......
119909117   ......KILKE......IVALY......YLVME......IIHRD......ADFGF......KADLW......
119914426   ......RLTHE......IVTFH......WLVVE......ILFCD......SNFSL......TSDLW......
119960545   ......KAAAS......IVGVL......YLVME......LIHRD......GDFGL......RSDIY......
121533693   ......QAAAR......IVNIY......YIVME......LIHCD......TDFGI......KSDIY......
121637194   ......RIAGR......VVPIH......FLEMR......VMHRD......VDFGI......RADIY......
153005969   ......RAASR......VVTVT......FMVME......ILHRD......LDFGI......RSDLF......
153004956   ......RAVAR......IVEIY......YLVTE......VIHRD......ADFGI......RSDLF......
158520440   ......QAMAQ......ILQVF......YIAME......IVHRD......ADFGL......CADVY......
158521378   ......ESAGT......IVTIY......YIAME......IVHRD......TDFGI......RSDIF......
125860127   ......HTLMK......YINLY......VLIMD......YVHND......ADYGL......HFDWW......
123343201   ......ESLCA......IIKIY......YVIVE......LAHRD......CDFGL......KADVW......
123372558   ......DCMKT......IINLY......YLCME......VAHND......CDFGL......KADIW......
123375343   ......RIISR......IVKVY......LIVME......ISHRD......IDFGL......KADIW......
123393188   ......ECLMN......IVRLY......YLLME......IAHCD......GDFGL......LADVW......
123406084   ......KCMIE......IVPLY......YLLME......IAHGD......CDFGL......AADIW......
123405832   ......DCLMT......IVRLY......YLLME......IAHCD......CDFGM......RTDIW......
123401267   ......DALIK......IVNIY......FIILE......MAHHD......ADFGF......ASDVW......
123401011   ......KSLMN......IIKIY......VVILE......IAHHD......ADFGI......KADIW......
123400631   ......NLMNE......IVRMY......YILYE......IAHLD......CDFGL......KADIW......
123408144   ......ECLKH......IINLY......YLLME......IAHQD......CDFGL......KADIW......
123417735   ......-MGKK......VCPLV......MFISE......FIYGD......SIPGF......RSDMW......
123424000   ......ECLIE......KTHLY......YLLME......IAHSD......SDFGL......IADMW......
123431857   ......DVMKD......IIRLY......YLLME......VAHLD......CDFGL......KADIW......
123428533   ......ECIKQ......IVSLY......YLLME......IAHCD......GDFGL......KADIW......
123427576   ......RCLME......IVYLY......YFVME......IAHRD......SDFGL......SSDIW......
123430668   ......-----......IIKFY......WFIIE......IFLRD......SDFTR......ASDFY......
123436185   ......QCLIA......IVSLY......YLLME......IAHCD......GDFGL......ISDMW......
123427089   ......EIVKK......ILEFN......VMLCP......IIHGD......IDFGI......KSDIY......
123428292   ......EVQSK......VLPII......IMVMP......IVHRD......ADFSC......KADIW......
123428273   ......EIAKM......CLKVF......IVTMP......IVHRD......CDYSV......PPDLW......
123431273   ......ECLKA......IIHLY......YLLME......IAHCD......SDFGL......KSDIW......
123447181   ......ECLML......IVRLY......YLLME......IAHCD......GDFGM......KSDIW......
123446818   ......RILER......IAKFV......VIITE......IFHRD......IDFGF......KADMW......
123439581   ......QCMIE......IVPLY......YLVLE......ISHRD......CDFGL......FCDIW......
123438843   ......ETLTS......IISLY......YLLME......IAHGD......CDFGL......KSDIW......
123448024   ......NIQIQ......IVPIV......CFIMP......YFHND......CDFGF......LADNW......
123447937   ......DCLIS......VVNLY......YLLLE......VAHGD......CDFGL......KADIW......
123447939   ......ELMKE......VIKMI......YLLME......IAHSD......CDFGF......KADIW......
123447943   ......ECMKR......ACNLY......YMLLE......IAHSD......CDFGL......KADIW......
123438775   ......NILQK......IMRLL......CLVTE......IIHRD......CDFGF......KVDIW......
123455852   ......-----......IIQLY......DFFQE......YIHRD......EDLEN......KSDIY......
```

```
123976861  ......ESLQQ.....VVRLY.....YLLME.....ISHGD.....CDFGM.....KADIY.....
123976369  ......KALTS.....IVRVY.....FLILE.....FAHCD.....CDFGL.....KADVW.....
123975407  ......SLQKS.....IVKII.....YIVLE.....IVHRD.....CDFGF.....KIDIW.....
123451416  ......EIMAS.....TINLY.....YMLME.....IAHSD.....CDFGL.....KADIW.....
123976155  ......TTLTK.....VINIY.....VLILE.....ICHHD.....ADFGI.....KADVW.....
123456010  ......DILKK.....IMRLL.....CLVTE.....IIHRD.....CDFGF.....KIDIW.....
123477864  ......ECLKS.....IVNLY.....YLLME.....ISHCD.....CAFGL.....SSDIW.....
123474568  ......RIMKK.....IVNFR.....YLIME.....FVHQD.....GDFGI.....KEDVW.....
123477866  ......DCFKI.....IVNLY.....YLVME.....IAHCD.....CNFGL.....AADVW.....
123477633  ......KIMMS.....ILSLI.....IIFMP.....IVHRD.....SDFSV.....PADVW.....
123466313  ......DILSK.....VITLN.....VIVMP.....IVHRD.....SDFSV.....PADVW.....
123475956  ......DCLME.....IISLY.....YLLME.....ISHSD.....CDFGL.....KADIW.....
123477902  ......RNLAK.....ILSIH.....YIVLE.....FAHKD.....SDFGI.....KEYVW.....
123476225  ......DSMSL.....IVKLH.....YLVLD.....VAHRD.....TDFGL.....RSDIW.....
123471352  ......DIMAK.....VISLI.....FIIMP.....VVHRD.....TDLSV.....KTDVW.....
123476150  ......ECLKT.....IINLY.....YLLME.....IAHND.....CDFSL.....KADIW.....
123477874  ......ECLKS.....IVRLY.....YMMME.....VAHSD.....CDFGL.....IADIW.....
123475744  ......TMMNK.....ICNLS.....YLILE.....FVHYD.....CDFGI.....AADIY.....
123469128  ......AVMRT.....IINLV.....YIFEE.....IAHRD.....ADFGY.....LADAW.....
123469772  ......ECLKA.....IMNLY.....YLLTE.....IAHND.....CDFGI.....KADIW.....
123489101  ......EFLKK.....IVQLY.....FIVFE.....IVHRD.....IDWGC.....GCDAW.....
123496029  ......DSLAI.....IVRLY.....YLLLE.....LAHGD.....TDFGM.....KSDIW.....
123487166  ......NLMKK.....IVKLY.....FLVME.....IIHRD.....ADFGF.....AVDMW.....
123505557  ......--LKD.....ILKIY.....WYYIE.....VILKD.....ADFTR.....ASDLY.....
123490423  ......NALIK.....VINIY.....VLVLE.....ICHHD.....ADFGI.....KADIW.....
123502260  ......NVLVH.....LIHLY.....YMIID.....YAHHD.....ADFGM.....KGDIW.....
123498923  ......DIMTV.....VIKIL.....VYVME.....IIHYD.....ADFGL.....STDTW.....
123486609  ......YIHSR.....IIRLH.....YIILE.....VAHRD.....SDFGL.....KADIW.....
123483773  ......ECMLQ.....TVTLY.....YLILE.....ISHGD.....CDFGL.....AADIW.....
123977201  ......RNMRL.....IIRMK.....CIVFE.....IVHQD.....SDFGI.....KEDVW.....
123509050  ......SLLQE.....IVQLY.....-LVFE.....IVHRD.....IDWGC.....GCDIW.....
154412195  ......NALMQ.....IIKLY.....YIILE.....IVHLD.....ADFGV.....AADVW.....
154412173  ......NCLIS.....TINLY.....YMLME.....IAHLD.....CDFGL.....KADIW.....
123492124  ......FILKT.....IVQII.....SIVME.....IIHRD.....GDLGL.....AVDMW.....
123507686  ......DCLLR.....IVSLY.....YMLME.....IAHSD.....GDFGL.....VADIW.....
123506694  ......SILDT.....VTQLV.....YIVME.....INHFD.....ADFGM.....PSDIW.....
123495559  ......DVMCQ.....IMSYH.....ILVLP.....LAHRD.....HHFKS.....KSDVW.....
123493751  ......DIMAK.....VISLI.....IIIIP.....VVHRD.....TDLSV.....KADVW.....
123480159  ......DCLIA.....IVSLY.....YLLME.....IAHCD.....TDFGL.....AADIW.....
123493778  ......EIMRQ.....VVQLH.....YLIVD.....IVHQD.....SDFGI.....KEDVW.....
123479513  ......EIQHQ.....ITKFY.....FVFLE.....FLFID.....NDFTG.....ASDLY.....
154418181  ......EIAQS.....CLPIL.....YVIMP.....YVHRD.....CDFSV.....KADMW.....
154414926  ......EFLSQ.....IIQCY.....YLILE.....IAHMD.....ADFGL.....KADIY.....
154422556  ......RVVQN.....IVEVK.....VMVMP.....IAHRD.....IDFGL.....KVDVW.....
124001155  ......ESMIE.....IVPLY.....YLLME.....ISHGD.....CDFGL.....FCDIW.....
154413108  ......NILKK.....IMRML.....NVVSE.....IIHRD.....CDFGF.....KIDVW.....
154416737  ......--LSK.....IIPSY.....GLTMK.....YIWLD.....TDIGN.....KSDIF.....
154420593  ......EILKS.....VVQLY.....ILVFE.....VVHRD.....IDWGC.....SCDLW.....
123208199  ......DIMRG.....IVLLL.....VVVTE.....ILHRD.....CDFGF.....STDLW.....
124008919  ......EAGQ-.....IVNSM.....FIVME.....IIHKD.....TDFGI.....TNDIF.....
124002699  ......ALMAK.....IVDLY.....FLVME.....IVHRD.....MDFGI.....RSDIF.....
124002151  ......HIVKN.....IRKVF.....ALFLE.....VIHKD.....IDFGI.....RTDLY.....
124005244  ......EAMAS.....IIDLY.....FLIME.....IIHRD.....LDFGI.....CSDIY.....
124006752  ......KISQD.....IPKAL.....AILQE.....VIHKD.....IDFDI.....RTDLY.....
124007253  ......QLCAQ.....IVKLL.....FAVFE.....IVHRD.....LDFGA.....KLDLY.....
124005397  ......TTLSN.....IITLY.....FLIME.....VIHRD.....LDFGI.....RTDVY.....
124002442  ......ERVKY.....LVRYY.....IVVME.....IIHRD.....LYFGL.....RSDIW.....
124006477  ......EILKS.....VLRAH.....SFAMN.....VIHKD.....IDFGI.....RTDLY.....
124003737  ......QLSAK.....IVKLL.....FAVFE.....IVHRD.....LDFGI.....KSDLY.....
145473911  ......QLSKV.....VIRFY.....FIITE.....ICHRD.....IDFGE.....KSDVF.....
145473975  ......QALRM.....ILKLF.....YLLTE.....VMHRD.....IDFGL.....QCDIF.....
145475109  ......LFLSK.....IIKMY.....YIVLE.....IIHRD.....CDFNW.....KLDIW.....
145475743  ......GMKRQ.....LIRLQ.....YVVEE.....FIHGD.....CLLHY.....TSDLQ.....
145475319  ......QIMKE.....IISFY.....NIVLE.....IMHRD.....ADFGL.....KCDIF.....
145475021  ......EILRR.....IILLL.....CLVTE.....IIHRD.....CDFGF.....TVDLW.....
145475299  ......NVLKL.....LMKVF.....YLICE.....IIHRD.....ADFGF.....KSDIW.....
145475013  ......EILRR.....IILLL.....CLVTE.....IIHRD.....CDFGF.....TVDLW.....
145479335  ......NALKS.....ILKLI.....YIITE.....FIHRD.....ADFGF.....KGDVW.....
145479743  ......--LKG.....LVNTI.....FVLME.....IIHRD.....CDFGL.....SVDIW.....
145476861  ......EILES.....FAQYY.....YIVQE.....IIHRD.....IDFGL.....SVDIW.....
145480717  ......SLLNQ.....IVKLK.....YIVME.....IMHRD.....IDFGL.....KADIY.....
145476755  ......KILEK.....FVKII.....YIVME.....LIHRD.....IDTGQ.....KIDIW.....
```

145480431	......DQLKS.....TIKVF.....YIIME.....ILHHD.....SDFGI.....KCDIF.....
145479029	......QILRL.....VLTIK.....FLVEE.....IIHRD.....ADFGL.....KVDIY.....
145477213	......MTMQS.....IVKIK.....FIIMD.....YIHRD.....CDFGF.....QCDVF.....
145475921	......KIMGF.....VVKLI.....YIVQE.....IIHRD.....ADFGF.....KSDIW.....
145477107	......QIMKQ.....IVKFI.....YIVTE.....IIHRD.....ADFGF.....KSDIW.....
145479075	......QIYKN.....VVALI.....YCVFE.....IVHRD.....ADFGF.....KCDIW.....
145477043	......QLLQR.....LITIY.....YVFMD.....MIHRD.....TDFGL.....KCDIF.....
145479177	......KTFQS.....LVKMI.....CIVME.....IIHRD.....ADFGF.....KCDIY.....
145478405	......YNARC.....IIQEI.....CLVME.....ISHRD.....LDFGV.....EIDIW.....
145476801	......EALSQ.....IMRLY.....YLITE.....IVHRD.....ADFGF.....KSDIW.....
145476091	......DIMKD.....IVKLL.....YIVSE.....IVHRD.....ADFGF.....KCDVW.....
145480333	......KALQR.....IVKMK.....YIVME.....YVYRD.....IDFGL.....KIDMF.....
145476023	......RIMEM.....VINLK.....YLVME.....VIHRD.....ADFGL.....KADIW.....
145477189	......QILQK.....IISFE.....IIVME.....IAHRD.....CDVGF.....KSDVF.....
145479329	......EILKK.....IVKFI.....YIITE.....IIHRD.....ADFGF.....KCDIW.....
145477597	......EILKK.....IVRFI.....YIIIE.....IIHRD.....ADFGF.....KCDIW.....
145477607	......QILEQ.....IVQYL.....YICME.....IAHCD.....CDFAS.....LSDVF.....
145479855	......RSMKQ.....IVKFL.....YIISE.....LIHRD.....ADFGF.....KCDIW.....
145479913	......EINNE.....FVKVK.....YIIMD.....IMHRD.....VDFGL.....KCDIF.....
145477211	......QIHSL.....IVRLL.....YLLLE.....IIHRD.....IDFGL.....KCDIF.....
145477305	......---QN.....VVNIL.....IITMD.....LIHRD.....CDTGL.....KTDIW.....
145480365	......NALKL.....VIKLH.....YIITE.....VIHRD.....ADYGF.....KTDIW.....
145479267	......D-RQQ.....LIKIK.....SITVE.....NEIQD.....FDFLE.....STDVF.....
145476641	......EKMST.....ILRVY.....YVIQE.....IIHRD.....CDFGL.....AADAF.....
145477209	......SILQS.....IIKVK.....WLITD.....YVHRD.....IDFGL.....LSDIF.....
145478485	......GI---.....LLQYL.....WIFTE.....IVHCD.....IDYGS.....KIDIW.....
145480603	......FCMQN.....MVQLK.....YILME.....FIYRD.....IDFGL.....KSDMF.....
145480011	......RMHQN.....IIQFK.....YIIME.....IIHRD.....ADFNF.....KMDVY.....
145479957	......TVMKQ.....IVELI.....YMIIE.....YIHRD.....ADFGF.....KSDIW.....
145485793	......SLLQS.....ILKLK.....YIVTE.....IMHRD.....IDFGL.....RADVY.....
145483193	......KILKE.....INRIY.....YIVLE.....IAHRD.....GDFGF.....KVDIW.....
145481923	......EILRT.....LIKLQ.....YLIFD.....IIHRD.....IDFGL.....KSDMF.....
145481943	......EIYLK.....LVSMI.....YCVFE.....IVHRD.....ADFGF.....KCDIW.....
145486214	......TLMME.....VVRIL.....FLIIE.....YMHRD.....ADFGL.....KCDIW.....
145481981	......TALQV.....IVKML.....YIITE.....VIHRD.....ADYGF.....KSDIW.....
145484306	......QILRT.....LIKLN.....YLVFD.....IIHRD.....IDFGL.....QIDMF.....
145481589	......ESMRS.....LIRYK.....NIVME.....IVHRD.....IDFGF.....ANDIW.....
145485825	......EILKQ.....VISLV.....YIILE.....YSHRD.....TDVNL.....KADIY.....
145483261	......KVMQK.....IVQLM.....YIIQE.....IIHRD.....ADFGF.....KTDIW.....
145485199	......QILRH.....VIQMI.....CMLLE.....ITHGD.....CDLGF.....KCDMF.....
145484376	......KILSM.....LVKLV.....CLVME.....IVHRD.....ADFGF.....KCDIY.....
145484081	......KILSQ.....IIKPQ.....LLFLE.....IAHND.....ADFGF.....FCDVY.....
145483819	......HILRS.....IVQLI.....YLVFE.....YIHRD.....IDFGL.....KVDMF.....
145483739	......QILRA.....FLVLK.....YVVTS.....IIHRD.....VDFGL.....KVDIF.....
145483747	......NALKS.....ILKLY.....YIITE.....YIHRD.....ADFGF.....KGDVW.....
145484539	......ALMQK.....INPII.....YVVCP.....IIHRD.....SDPGF.....KCDIW.....
145485602	......EALSQ.....IMRLY.....YLITE.....IVHRD.....ADFGF.....KSDIW.....
145482813	......SCLIN.....VQNLH.....YLIQE.....IMHRD.....TEFDL.....KVDLF.....
145482941	......DILRK.....VISLL.....FLITE.....IIHRD.....SDFGL.....KIDSY.....
145481775	......TLLED.....VVKLI.....CLIME.....IIHRD.....ADFSL.....KCDIY.....
145482361	......RKRKI.....LVQLL.....MVVSE.....QAHGD.....AEYGF.....KSDVF.....
145486228	......EILGK.....IVQMY.....YIFLE.....VMHRD.....SDFGF.....KCDIW.....
145484527	......EILKK.....IVRFI.....YIIIE.....IIHRD.....ADFGF.....KCDIW.....
145484013	......NVLRK.....IVGFI.....YLVTE.....IIHRD.....ADFGL.....LSDMW.....
145485107	......HIQST.....IVKVI.....YIISE.....IVHRD.....GDFGW.....KVDSW.....
145481985	......HILRQ.....TSHII.....YLIQE.....ILHRD.....SDFGL.....KVDVY.....
145485973	......KKIYS.....VIQYL.....YIFME.....ITFVD.....GQINI.....ASDLY.....
145485829	......QILEL.....IVKLI.....YLVLE.....VVHRD.....CDFGF.....KSDVF.....
145484374	......KVIKE.....LVKLI.....CIVME.....FVHRD.....ADFGF.....KCDIF.....
145482801	......NFQTG.....IVQTF.....YIVME.....IIHRD.....GDFGV.....KCDIF.....
145483175	......AILRR.....VQRLQ.....YIVYD.....VFHRD.....VNFSS.....QIDVF.....
145480743	......QAIQR.....IIGII.....VVVME.....LFHRD.....IDFGL.....SVDIW.....
145482851	......EILKQ.....LLHID.....EVVME.....IIHRD.....ADFGF.....KCDIY.....
145481137	......IALQV.....IVKMH.....FIITE.....VIHRD.....ADYGF.....KSDVW.....
145482849	......SIIKE.....IIKFI.....IAVFD.....YIHRD.....ADFGF.....KCDIW.....
145487001	......KALEL.....VVRFI.....YIVME.....IIHRD.....ADLGF.....SSDMF.....
145487163	......EIMKL.....IVKLI.....YIITE.....IIHRD.....TDFGF.....KCDIW.....
145487814	......HILRS.....ILKFY.....YIITE.....IIHRD.....ADFGF.....KTDIW.....

```
145487210   ......QASQK......II-VY......WILYE......YYHCD......CDLGF......KTEVF......
145489083   ......EILRQ......IIKLI......YLVFE......IVHRD......ADFGF......KCDVW......
145489803   ......ETMRI......ILQLH......YLVME......IMHRD......ADFGL......VCDIF......
145487858   ......AVMNK......FVKIQ......LIIMN......IIHRD......IDFGF......SCDIF......
145487678   ......KVLRT......IIKLY......YLIME......IMHRD......ADFGF......KADIW......
145489364   ......QYQNQ......LIKIY......CIIME......LVHRD......IDFGL......KVDIW......
145488563   ......DIMKD......IVKLL......YIVSE......IIHRD......ADFGF......KCDVW......
145488003   ......QIVDQ......VIKIA......CIIME......VIHRD......IDFGV......QCDIF......
145488003   ......EAYKR......TLRVY......YIFTE......INVRD......SD--Y......KCDIY......
145486379   ......KVLRT......TVSLL......YVVME......IFHRD......TDYGL......KVDLY......
145488701   ......KIMRD......VVRFI......YIVQE......IIHRD......ADFGF......KSDIW......
145487065   ......DIMKD......IVRLI......YIISE......IIHRD......ADFGF......KCDVW......
145487356   ......ETLKQ......IVKLV......LIVLE......FVHRD......IDFGI......KIDIF......
145489606   ......SIYKK......IIKVF......YVIME......IIHRD......IDFGV......KCDIY......
145487185   ......NALRS......IMKLH......YIITE......FIHRD......ADFGF......QGDVW......
145486503   ......ELLSS......IVKHI......LLVME......VIHRD......GDFGL......PVDVW......
145488330   ......KTLSE......IVNIL......IIIMD......LIHRD......CDTGL......KVDIW......
145486591   ......QILKK......LIIVY......FIIME......IMHRD......GDLGA......QTDIY......
145487003   ......SVLAK......VLKLE......YILME......IIHRD......ADLGF......QSDIF......
145489863   ......SVYRS......IIKLY......DFYFE......LFHRD......CDFGV......KNEAW......
145488340   ......DNTSK......IVKFE......YFFME......VIHRD......ADFGL......KSDVW......
145490323   ......SSLRK......VIFFN......YIVYN......VFHRD......TNFCY......KADMF......
145489602   ......KIMQK......IVTLL......YIVQE......IIHRD......ADFGF......KTDVW......
145487808   ......NILKL......VPNFE......YIIME......VMHRD......INFSN......ECDMF......
145486509   ......KALQN......IIKLK......YIITN......FIHRD......IDFGF......KCDIF......
145492999   ......ELMKI......IVRVE......VMIME......IIHRD......ADLGL......SADIF......
145494979   ......QNQFQ......IVKMI......YFILE......IIHRD......GDFGF......KVDIW......
145492461   ......AILTK......IVKLI......YMILE......VIHRD......ADFGF......KCDIW......
145491151   ......SILEK......IIRLL......YLVME......IIHRD......ADFGL......TVDTW......
145492770   ......QILRH......IIQMI......CMLLE......ITHGD......CDLGF......KCDMF......
145494352   ......GILKI......ILKFY......YIITE......IIHRD......ADFGF......KSDIW......
145493125   ......ELMQM......IVGVH......ILILE......VIHRD......ADFGL......SADIY......
145491804   ......QVLET......VLNIQ......LIFSE......IILRD......SDFGV......TCDIY......
145494752   ......AILKQ......LMKVY......YIICE......IIHRD......SDFGF......KSDIW......
145492027   ......EIQNK......IVC--......CLRIE......LLHLD......ADFGL......KNDVY......
145493942   ......MTMQS......IIKNK......FIIME......YIHRD......CDFGF......QCDVF......
145490576   ......VLMME......VVRIL......FLIIE......YMHRD......ADFGL......KCDIW......
145490544   ......EILQK......FVKLL......YLITD......LIHRD......IDFGL......KCDVF......
145491989   ......RILAK......IVRLH......YQIID......IIHRD......ADFGF......KCDIW......
145492126   ......QIQKR......AVHFP......VISLQ......IAHND......ADFGC......KMNLF......
145495288   ......NILSQ......VVHLV......FMITE......FVHRD......CDFGF......EAEVF......
145495033   ......GILHS......ILKIF......YIIVE......VIHRD......IDFGL......LCDIF......
145491722   ......EIMKS......IVKLL......YIVTE......IVHRD......TDFGF......KSDIW......
145495625   ......TIMKE......IVGVE......VLILE......IIHRD......SDLGL......SADIF......
145493615   ......NINRT......IIKSY......YWVME......ICHRD......IDFGV......KVDVW......
145496913   ......EIVKK......LVKIL......FIFME......IMHRD......ADYGM......KCDIY......
145499389   ......-----......IVQIM......WIIQE......VTHFD......IDFGS......KIDVW......
145499679   ......MIWSE......IAKFY......YIVME......IIHRD......IDFGL......KSDIF......
145496308   ......YNARC......IIQEI......CLVME......ISHRD......LDFGV......EVDVW......
145496621   ......SIMES......IIKLY......YLVME......IIHRD......ADFGL......AVDMW......
145496543   ......QILSK......ILKLY......YIITE......IIHRD......ADFGF......KSDIW......
145497467   ......RIHKH......IIQFH......YIIME......IIHRD......CDFGL......KIDVW......
145497931   ......NMLKR......IIEIL......YIILE......IAHRD......ADFGY......KSDIW......
145500097   ......KIQST......ILKVH......YIISE......IIHRD......GDFGW......KVDCY......
145496983   ......EIMKS......IVKLL......YIVTE......IVHRD......TDFGF......KSDIW......
145496133   ......EILRR......IILLL......CLVTE......IIHRD......CDFGF......TVDLW......
145498345   ......TIHGM......IVKFI......FIVTE......IAHRD......CDFNA......ECDIF......
145497743   ......NIVQS......ITKIK......VIIYE......LIHRD......IDFGL......KCDMF......
145498415   ......NALLQ......VVRIY......YMILE......IIHRD......ADLGF......AVDIF......
145497365   ......YVLQK......IVKFY......YICME......IIHRD......IDFGM......KCDIW......
145499443   ......EIHCN......VVKML......NLILE......TLHRD......ADFGF......KVDIW......
145498224   ......KILEQ......LVKIY......YIVME......VIHRD......TDFGM......KCDVY......
145496553   ......NILKI......LPKLV......YIVME......VIHRD......IDFAK......ESDMF......
145499076   ......QALKL......VLGFN......YLAMD......IIHRD......IDFGL......KIDVF......
145498795   ......YIMQL......LISLH......ILLME......ITHRD......GGYEE......LADVY......
145499552   ......LNQKP......LLHCD......YLVMD......IIHKD......FDFGL......KSDIF......
145500159   ......QILRH......IITM-......CMLLE......ITHGD......CDFGF......KCDMF......
145496591   ......-VQKN......LVQVY......IVIME......VIHRD......ADFGI......KVDIY......
145497755   ......QVLSL......LMKIY......YLICE......IIHRD......ADFGF......KSDIW......
```

```
145495742 ......NALKL......VIKLH......YIITE......VIHRD......ADYGF......KTDIW......
145497047 ......ELQKS......IAKIY......YIIME......IIHRD......IDFGF......KQDVW......
145499032 ......QVMQK......LVGFY......YLFIE......VIHRD......ADFGF......QFDIW......
145498343 ......HILSQ......IVHLV......FMVTE......FVHRD......CDFGF......EAEVF......
145496244 ......QLKST......LVQYL......WIFTE......IVHCD......IDYGS......KIDIW......
145499912 ......GILHS......ILKIY......YIIVE......VIHRD......IDFGL......LCDIF......
145495778 ......RKLQI......IVKLK......YIVME......YVYRD......IDFGL......KIDMF......
145497489 ......IALKT......IEQIY......VIIIE......FVHRD......VDFGF......KGDVF......
145497911 ......RILSQ......ILKLR......ILIFD......IMHRD......IDFGF......LIDIY......
145497393 ......KIMRQ......LMKLY......YVAME......IMHRD......ADFGL......VCDIY......
145498614 ......KILQE......FVHLY......YLIQN......IIHRD......ADFGL......QIDIY......
145499257 ......GILRS......ILKIY......YIIVE......VIHRD......IDFGL......LCDIF......
145498170 ......QIMKQ......IVKFI......YIVTE......IIHRD......ADFGF......KSDIW......
145498216 ......EIIQM......LIQVF......YIIME......IIHRD......ADLGM......KADVY......
145498011 ......EILRR......IILLL......CLVTE......IIHRD......IDFGF......TVDLW......
145497909 ......KVNKT......LMKLL......IFIYE......ILHRD......IDFGF......LCDIY......
145500008 ......-----......IVQIM......WIIQE......VTHFD......IDFGS......KIDVW......
145496037 ......KIMQK......IVQLL......YIVQE......ITHRD......ADFGF......KTDVW......
145498196 ......EILKQ......ILTLY......ILITE......YVHRD......IDFGF......VGDVF......
145498206 ......EILNE......IVRII......YVVQE......IVHRD......SDFGL......TVDIW......
145496168 ......HCLGK......CIRLY......YMLMQ......IVHRD......IDFGT......KSDIY......
145495973 ......QTNCK......IISLI......ALVFE......IIHRD......IDFGC......SSDIY......
145496973 ......YVLKR......VLKLC......ILVTE......YVHRD......IDFGF......VGDIF......
145501355 ......QIMKQ......IVKFI......YIVTE......IIHRD......ADFGF......KSDIW......
145503641 ......KILSK......IVQLF......YIFLE......VIHRD......SDFGF......KCDIW......
145502789 ......TILTK......VVQLI......FLILE......VVHRD......IDFAF......PTEVY......
145500576 ......QIIRS......ITKFF......FLIYE......ILHRD......ADFGL......KIDIY......
145503619 ......QILQK......FVKLH......YLVTD......IIHRD......IDFGL......KCDIF......
145501397 ......EILNL......LVELV......YIIME......VIHRD......ADLGM......KADVY......
145504476 ......EVLQK......VVKFI......HFVYE......IIHRD......ADFGF......KADIW......
145503564 ......QYQKQ......IVKIF......IIVME......LLHRD......IDFGL......TIDIW......
145502807 ......AIQQG......IIQLY......YLFME......VCHLD......SDFGL......LLDVW......
145503659 ......QIDDK......IIKIL......LMVME......YSHRD......IDFGL......TVDIW......
145501707 ......ALLKQ......IIKII......FIITE......VAHRD......CDFGF......KTDVF......
145503578 ......EYQNK......IIRIY......VLVME......LFHRD......IDFGL......AVDVW......
145504232 ......NVLRT......IIKLY......YLIIE......IMHRD......ADFGF......KADIW......
145500302 ......SILRQ......LLKLE......YIVTE......IFHHD......INFGK......KKDIY......
145500626 ......EIMRL......IIKLF......YLLLD......IIHRD......ADFGL......KVDVY......
145500796 ......KIYMK......LLGAH......IYIMP......IMHRD......MDFGY......LCDIY......
145500260 ......KIMQK......IIQLV......YLILE......VMHRD......VDFGL......ICDEF......
145502729 ......QILED......VVKLI......YLILE......VVHRD......CDFGF......KSDVF......
145500622 ......SVMRT......VVKFV......YLMME......IVHRD......ADFGF......KADVW......
145500480 ......KILLQ......LLRVY......YIVSE......IIHRD......VDFGF......KSDVW......
145500312 ......RALSL......VVKMY......YIITE......VIHRD......ADYGF......KTDIW......
145500304 ......QYLKL......IVKYL......ILISE......IFHRD......IDFGL......KVDIY......
145501274 ......KVMKY......ILKLY......YLITE......LMHRD......-QFGL......KCDVF......
145501351 ......AALQS......VVQLK......LLIME......FVHRD......SDFGF......LCDMF......
145504378 ......AVMNK......FVQIQ......LIIMN......IIHRD......IDFGF......SCDVF......
145504174 ......KIQKQ......LIRID......IIVME......YFHRD......IDFGI......SIDVW......
145503546 ......KIEKS......IIKIY......LVIME......YIHRD......IDFGL......SIDIW......
145501329 ......QILQK......IIAFE......CIVME......IAHRD......CDVGF......KSDVF......
145500436 ......KVMQT......VVRML......YMAIE......YIHRD......ADFGF......KSDIW......
145504424 ......-ISQH......IVKVY......IIIME......IIHRD......SDFGT......QVDVY......
145503643 ......QNDDK......IIKIL......LIVIE......YCHRD......IDFGL......TADVW......
145506220 ......ESLRQ......IVKLI......QIVLQ......FVHRD......IDFAS......KVDIF......
145504669 ......SIMKQ......IVQLI......YIITE......FIHRD......ADFGF......KCDIW......
145504845 ......SVLTK......VLKLE......YIVME......VIHRD......ADLGF......QSDIF......
145509302 ......QYQNQ......LIKIY......CIIME......LVHRD......IDFGL......KVDIW......
145504767 ......DIMKD......IVRLI......YIISE......IIHRD......ADFGF......KCDVW......
145508744 ......TILRS......VIQLL......YLIFE......YIHRD......IDFGL......KVDMF......
145505003 ......NILKQ......LPRFE......YIVLE......IIHRD......VDFGL......ESDIF......
145508063 ......KILSQ......IIKFI......SLFLE......IAHND......ADFGF......SADVY......
145507975 ......EALST......IMKLY......YLITE......IIHRD......ADFGF......KSDIW......
145509304 ......QYQNQ......LIKIY......CIIME......LVHRD......IDFGL......KVDIW......
145507194 ......LLLRM......FIKCI......YWVTE......VLHQD......SDLGI......KCDVW......
145505662 ......VILQK......LVKYY......IIIME......ILHRD......TDFGI......KRDVY......
145507660 ......WIMQK......IVQLI......YIIQE......IIHRD......ADFGF......KCDVW......
```

145507206	......NILKQ.....LPHFE.....YIVLE.....IIHRD.....VDFGL.....ESDIF.....
145505609	......EIMKK.....IVELY.....YLVQE.....FIHRD.....VDFGF.....KCDVW.....
145508387	......NILRS.....IVKLI.....YLVFE.....YIHRD.....IDFGL.....KVDMF.....
145509108	......QTLAR.....VVKFI.....YFVYE.....IMHRD.....GDFGF.....KADIW.....
145507917	......EVMSK.....IIQVI.....VIIQE.....IIHRD.....ADFGF.....QADIF.....
145508525	......NALRE.....ILKLH.....YLVTE.....IMHRD.....IDFGL.....QCDIF.....
145504627	......NALRS.....IMKLH.....YIITE.....FIHRD.....ADFGF.....QGDVW.....
145506809	......QVLAQ.....VVKMN.....YLVME.....VIHRD.....GDLGF.....LADMF.....
145507802	......EIMKK.....IVELY.....YLVEE.....FIHRD.....ADFGF.....KCDVW.....
145505587	......EIMKK.....VVKLL.....YIITE.....IIHRD.....TDFGF.....KCDIW.....
145508455	......NALKS.....ILKLH.....YIITE.....YIHRD.....ADFGF.....KGDVW.....
145508902	......--LSQ.....VVQLL.....FIVQE.....IVHRD.....TDFGL.....SVDIW.....
145509142	......QNMKL.....VVKFI.....YIVME.....VMHRD.....LTLCI.....TADIF.....
145508463	......QILRA.....FLVFK.....YVVTS.....IIHRD.....VDFGL.....KVDIF.....
145505575	......QTLSK.....IVKFI.....YLIYD.....ILHRD.....ADFGF.....KADVW.....
145508606	......KALQA.....IIKII.....IVVMD.....FFHRD.....IDFGL.....SIDIC.....
145504849	......KALQL.....IIEFY.....FIVLE.....IIHRD.....GDLGF.....ASDMF.....
145507760	......QTLSK.....IVKFI.....YLIYD.....ILHRD.....ADFGF.....KADVW.....
145508988	......NILRT.....ILELK.....FIVTE.....VIHRD.....IDFGL.....KVDIF.....
145510712	......QILRS.....LVEIY.....QIVTE.....ILHRD.....IDFGL.....KVDVY.....
145512613	......EIMKL.....IVRLI.....YIVTE.....IIHRD.....TDFGF.....KCDIW.....
145513620	......NINLL.....VASMV.....ILIYP.....IIHRD.....IDFGF.....KCDIY.....
145512804	......Q----.....LIKIL.....SITSE.....II--D.....YDFLE.....NVNVF.....
145513426	......NTLKL.....ILKLL.....FLVTE.....IMHGD.....ADFGF.....QADMW.....
145510428	......-----.....--QLK.....YPI-E.....YIHFD.....CDFSS.....TCDIW.....
145513114	......NLYQV.....VIKVY.....VFAME.....LFHRD.....ADFGA.....KSDVF.....
145513284	......MIQKM.....-----.....CIAMD.....IIHRD.....CDLGL.....SVDIW.....
145511794	......ETMRI.....ILLLH.....YLVME.....IMHRD.....ADFGL.....SCDIF.....
145513508	......--LSQ.....VVQLL.....FIVQE.....IVHRD.....TDFGL.....SVDIW.....
145510160	......KLLSE.....VIKLF.....ILVLE.....IIHRD.....IDLGL.....QADMY.....
145509831	......KILKQ.....LMKVY.....YIVCE.....IIHRD.....SDFGF.....KSDIW.....
145510983	......NILFE.....ILEIE.....IFIMK.....IIHRD.....IDFGF.....LVDIY.....
145510168	......KMFSG.....ILKFY.....WVILE.....IIYCD.....SDLGN.....QSDLW.....
145513554	......KVQKQ.....-IQFP.....FISQK.....IVHND.....ADFGC.....KMNLF.....
145510322	......QILKM.....IVELY.....ILILE.....-IHRD.....IDFGF.....NCEIF.....
145512892	......EILEQ.....IVKMY.....YIVSE.....IIHRD.....ADFGF.....KTDIW.....
145510562	......QIRSQ.....LCKML.....FIMYE.....WVHSD.....IDYGS.....QIDIW.....
145513288	......--FKE.....LVNIY.....YIVME.....LAHRD.....CDFGT.....SVDIW.....
145509489	......GILKI.....ILKFY.....YIITE.....IIHRD.....ADFGF.....KSDLW.....
145510080	......AIFSQ.....IVKFY.....LIEME.....FVHRD.....TDFGL.....NVDVW.....
145513674	......QTLAR.....VVKFI.....YFVYE.....IMHRD.....GDFGF.....KADIW.....
145511193	......KVMKY.....ILKLY.....YLITQ.....LMHRD.....SDFGV.....KCDVF.....
145510430	......DMMRR.....LINIL.....YIVME.....YAHRD.....CDFGT.....TVDVW.....
145511748	......QPKKQ.....LVRFL.....WIFQE.....IVHSD.....IDYGS.....KIDVW.....
145510258	......QIMQR.....LVKFY.....YFMME.....IVHRD.....SDFGF.....KTDIW.....
145512878	......SALKA.....IMKLH.....YIITE.....YIHRD.....ADFGF.....KGDVW.....
145509723	......RILQR.....IIRHY.....YTQIE.....IIHGD.....ADLNH.....QQSLQ.....
145516334	......HINRT.....VVKAL.....HLVME.....ICHRD.....IDFGV.....KVDIW.....
145515643	......EALKQ.....LVKLI.....SIVME.....FVHRD.....IDFGI.....KVDIF.....
145516647	......NILRR.....IINLV.....FLVFE.....IIHGD.....CNFNQ.....SIDIY.....
145517053	......QIMKS.....IVQLK.....NMILE.....VVHRD.....GDFGF.....KCDIW.....
145517284	......QILKS.....IIEII.....YIILE.....IMHRD.....GDFGF.....KSDIW.....
145515181	......SILQQ.....IVKLF.....YLFLE.....IIHRD.....TDFGF.....KCDVW.....
145515838	......DILNF.....IVNVY.....VIIME.....VIHRD.....ADFGV.....KVDIY.....
145514644	......SIIKQ.....LMKVY.....YIICE.....IIHRD.....SDFGF.....KSDIW.....
145517400	......KIHKL.....IIQLL.....YLVLE.....IIHRD.....CDFGL.....SVDIW.....
145517458	......MVLRK.....IIRLY.....ILIME.....FVHRD.....VDFGF.....KGDVF.....
145516859	......EILSE.....ILQLH.....YLVME.....IMHRD.....ADFGL.....LCDIF.....
145517420	......EILEQ.....LVKYI.....YIIMD.....ITHRD.....IDFGL.....SVDMW.....
145515311	......KVMKC.....LLKFY.....YIVMS.....FMHRD.....SDFGV.....KCDIF.....
145517682	......IIQSV.....ILKCY.....ALVLE.....IIHRD.....ADLGI.....KVDCY.....
145517718	......KALSL.....VIKMY.....YIITE.....VIHRD.....ADYGF.....KTDIW.....
145516474	......QILSA.....IIEVI.....YTIME.....FFHRD.....IDFGI.....SVDIW.....
145517504	......TTLKN.....IMKLY.....ILIME.....FVHRD.....VDFGF.....KSDIF.....
145517314	......EILQT.....IIKLF.....FIITE.....IIHND.....ADFGF.....QSDIW.....
145517524	......YALGL.....IIKMY.....YIITE.....IIHRD.....ADYGF.....KSDIW.....
145515864	......NILKY.....IINVV.....YVFME.....IIHRD.....SDFGM.....KTDVF.....
145516759	......QILIH.....IIEL-.....CLVLQ.....FTHGD.....CDFGF.....KSDLF.....

```
145515968    ......LIHMN.....VVKLY.....YLVLE.....IIHRD.....CDFGS.....KVDVW.....
145516426    ......EILKK.....IVRFI.....YIITE.....VIHRD.....ADFGF.....KCDIW.....
145518718    ......EILEQ.....LVKYI.....YIVMD.....ITHRD.....IDFGL.....SVDMW.....
145518680    ......MVLRK.....IIRLY.....ILIME.....FVHRD.....VDFGF.....KGDVF.....
145518448    ......RALSL.....VVKMF.....YIITE.....VIHRD.....ADYGF.....KTDIW.....
145519271    ......SLWKQ.....IVKFI.....YFFLE.....VFHRD.....ADFGF.....KCDVW.....
145518734    ......KIHKL.....IIQLL.....YLILE.....IIHRD.....CDFGL.....SVDIW.....
145520747    ......SSLRE.....ILKLH.....YLVTE.....IMHRD.....IDFGL.....QCDIF.....
145520397    ......NVLRK.....IVGFI.....YLVTE.....IIHRD.....ADFGL.....LSDMW.....
145519922    ......QIYEK.....IIQIY.....CIVME.....QIHRD.....IDFGW.....KIDIW.....
145521398    ......EALSQ.....IMRLY.....YLITE.....IVHRD.....ADFGF.....KSDIW.....
145518640    ......TTLKN.....IMKLY.....ILIME.....FVHRD.....VDFGF.....KSDIF.....
145518468    ......AIQSL.....ILKCY.....ALVLE.....IIHRD.....ADLGI.....KVDCF.....
145518438    ......RILRQ.....IIQYK.....ILVQE.....IFHRD.....IDFGL.....KVDIY.....
145520301    ......KILSQ.....IIKTQ.....LLFLE.....IAHND.....ADFGF.....FCDVY.....
145519471    ......EILKK.....IVRFI.....YIITE.....VIHRD.....ADFGF.....KCDIW.....
145520225    ......QLLQQ.....ILKLK.....YIVTE.....IMHRD.....IDFGL.....KADIY.....
145520681    ......QVLRT.....LV-LS.....YVVTS.....IIHRD.....VDFGL.....KVDIF.....
145521466    ......EILES.....FAQYF.....YIVQE.....IIHRD.....IDFGL.....SVDIW.....
145520014    ......HMMKQ.....MVEFI.....YFVSE.....IAHLD.....TDFAT.....EADLW.....
145519906    ......KGQFN.....VIRIY.....YLIME.....LIHRD.....IDFGL.....AVDVW.....
145521166    ......EVLMS.....IAKTY.....YIFRE.....LTNRD.....FDFGQ.....KSDIW.....
145519553    ......HLNRG.....VVKAH.....HLVME.....VCHRD.....IDFGV.....KIDIW.....
145519862    ......EVLIR.....IAKTY.....YIFRE.....LTNRD.....FDFGS.....KSDIW.....
145521582    ......QMLNQ.....LLHSD.....YLLMD.....IIHKD.....IDFGL.....KSDIF.....
145521414    ......AIHSS.....IVKFY.....YILLE.....IIHRD.....CDFGW.....KVDTW.....
145520777    ......RVKSK.....IRPVY.....DVLFD.....---RD.....-DFGL.....QYQ-W.....
145518015    ......QVLAQ.....VVKMN.....YIVME.....VIHRD.....GDLGF.....LADMF.....
145520729    ......KMCKD.....LVQIY.....YIFME.....IIHRD.....ADFGL.....RCDIF.....
145524241    ......QILSK.....LIKIY.....YIFME.....IIHRD.....CDFGF.....KTDVF.....
145524060    ......QIQQE.....IVKFF.....LIEME.....IAHRD.....TDFGL.....TVDVW.....
145523333    ......KILQK.....VISLL.....NIVLE.....YCHRD.....TDVNM.....KADIY.....
145523906    ......TILTK.....VVQLI.....FLILD.....VVHRD.....IDFAF.....PSEVY.....
145524559    ......DALQK.....IVKLI.....CILLE.....IIHRD.....ADFGF.....KSDMF.....
145522151    ......EIMKL.....IVRLI.....YIITE.....IIHRD.....TDFGF.....KCDIW.....
145523337    ......QILEL.....IVKLI.....YLVLE.....VVHRD.....CDFGF.....KSDVF.....
145524723    ......EILKK.....IVRFI.....YIITE.....VIHRD.....ADFGF.....KCDIW.....
145523301    ......SLLQS.....IVKLK.....YIVTE.....IMHRD.....IDFGL.....RADVY.....
145522125    ......DILSL.....IIKLE.....SIILE.....IVHRD.....ADFGL.....KVDIY.....
145524151    ......QILSA.....IIEVI.....YTIME.....FFHRD.....IDFGI.....SVDIW.....
145523047    ......IFGRQ.....IVKFY.....YMFME.....AVHRD.....IDFGM.....KNDVW.....
145524177    ......EAYKI.....IIKIE.....YLIME.....IIHRD.....IDFGV.....KCDIF.....
145522594    ......SILKL.....FITLK.....CLVTA.....IIHRD.....VDFGL.....KVDIF.....
145522598    ......FIMRS.....IPKLY.....GLVMT.....IMHRD.....IDFGF.....QCDVY.....
145528373    ......NLNRG.....VVKAL.....HLVME.....VCHRD.....IDFGV.....KIDIW.....
145525675    ......NICTT.....IIKLI.....VIILE.....FSHRD.....IDFGE.....KSDLW.....
145526987    ......QYLKL.....IVKYL.....ILIQE.....IFHRD.....IDFGL.....KVDIY.....
145526925    ......CIQSC.....ILKCY.....ALVLE.....IIHRD.....ADLGI.....KVDCY.....
145527120    ......KINQL.....VINVV.....LLVME.....IFHRD.....TDFGV.....KSDIF.....
145526525    ......KIYMK.....LLGAR.....IYIMH.....IMHRD.....MDFGY.....LCDIY.....
145528089    ......SLWKQ.....IVKFI.....YFFLE.....VFHRD.....ADFGF.....KCDVW.....
145527732    ......KICQY.....IIQLR.....ILIFD.....IMHRD.....IDFGF.....QVDIY.....
145527929    ......KTLEE.....ILKIY.....YIFME.....IIHRD.....SDYGL.....KADLF.....
145525473    ......EIIQV.....IAQFY.....VLLME.....IIHMD.....TDFGE.....AYDIY.....
145527280    ......RIHKH.....IIQFH.....YIVME.....IIHRD.....CDFGL.....KVDVW.....
145525084    ......INLYK.....IIEYY.....FIVMQ.....FQHRD.....CDFEF.....KCDIY.....
145527300    ......IALKT.....IEQIY.....VIIIE.....FVHRD.....VDFGF.....KGDVF.....
145527794    ......KIMGK.....IVQLL.....YIVQE.....IIHRD.....ADFGF.....KTDVW.....
145527730    ......GVSKT.....LMKLQ.....IFIYE.....IIHRD.....IDYGF.....LCDIY.....
145527879    ......AIVNK.....LVKMY.....VEFME.....IIHRD.....TDFGV.....KADIF.....
145526867    ......KVMQT.....VVRML.....YMAIE.....YIHRD.....ADFGF.....KSDIW.....
145526715    ......DILRI.....VIKLL.....FVVYE.....IIHRD.....ADFGL.....KVDVY.....
145527124    ......RILRQ.....LLKLY.....YIVTE.....IYHTD.....INYGK.....KKDIY.....
145525244    ......NILNH.....FIKLH.....YLILD.....IIHRD.....VDFGL.....VCDIF.....
145526422    ......NILRR.....IINLV.....FLVFE.....IIHGD.....CNFNQ.....SVDIY.....
145525635    ......EVLRQ.....VIKFI.....YIVLE.....VLHRD.....ADLGF.....AADMF.....
145526551    ......EFINK.....IAQIY.....QYLLK.....ILHLD.....CDFGE.....KADVF.....
145528307    ......EILKK.....IVRFI.....YIITE.....VIHRD.....ADFGF.....KCDIW.....
145525453    ......SILQS.....IVKLY.....YLFLE.....IIHRD.....TDFGF.....KCDVW.....
```

145526268	......QILIH......IIEL-......CLVLQ......FTHGD......CDFGF......KTDMF......
145526807	......KILLQ......LLRVY......YIVSE......IIHRD......VDFGF......KSDVW......
145526601	......KIHKQ......IIQLL......YLIQE......IIHRD......CDFGL......SVDIW......
145525595	......KVMKS......ILKCY......YIVMD......YMHRD......SDFGV......KCDIF......
145530073	......KIMQK......IVQLL......YIVQE......IIHRD......ADFGF......KTDVW......
145529964	......MALKK......IVEIY......IFVYE......IFHRD......IDFDR......QCDVW......
145529924	......QILKT......FLVLK......YLVTS......IIHRD......VDFGL......RVDIF......
145530387	......TAFQK......LIRVY......VLIME......LIHRD......IDFGL......SVDIW......
145530383	......TAFQK......LIRVY......VLIME......LIHRD......IDFGL......SVDIW......
145530383	......EYQKK......LVKIF......YLVME......YLHRD......TDFGL......EIDIW......
145528758	......SLLSS......IVKLK......YIIME......VMHRD......IDFGL......KADIY......
145530600	......MMLKT......IVKIL......YIVMN......VVHRD......IDFGL......SVDLW......
145528652	......GILHS......ILKIY......YIIVE......VIHRD......IDFGL......LCDIF......
145529864	......SSLRE......ILKLH......YLVTE......IMHRD......IDFGL......KCDIF......
145529884	......DICKM......LVQIY......YIFME......IIHRD......ADFGL......ECDIF......
145530505	......ETLKR......VIKFI......YLVYE......ILHRD......ADFGF......SADIW......
145528708	......QNQLQ......VVKMI......YFILE......IVHRD......GDFGF......KADIW......
145530523	......SLLER......VIKLY......LLVLE......IIHRD......IDFGF......PADIY......
145530511	......ESLSE......IIRVY......LILME......IIHRD......IDFGL......SVDIW......
145533563	......QINLH......VASMV......IFIYP......IIHRD......IDFGF......KCDIY......
145533865	......EILSR......LVNTI......FLLME......IIHRD......CDFGL......SVDIW......
145531405	......NSLQD......IVKCY......IIIME......IFHRD......CDFGS......QSDIF......
145532367	......MILKK......IVKLF......YLIQE......IVHRD......GDFGL......KCDIW......
145534790	......KNGQE......IVKVL......YLIME......IVHRD......TDFEH......KCDIF......
145531411	......QILRM......IVHII......YVIME......FIHRD......ADFGM......KCDVY......
145533068	......QIMKE......IISFY......NCVLE......IMHRD......ADFGL......KCDIF......
145533675	......--LSQ......VVQLL......FIVQE......IVHRD......TDFGL......SVDVW......
145534233	......NALKA......IMKLY......YIITE......YIHRD......ADFGF......KGDVW......
145531990	......SLLSS......IVKLK......YIIME......VMHRD......IDFGL......KADIY......
145531227	......QILRH......IITMI......CMLLE......ITHGD......CDFGF......KCDMF......
145531307	......QIQST......ILKVY......YIISE......IIHRD......GDFGW......KVDCY......
145532791	......EILKR......ILEIK......YIVTE......IIHRD......IDFGL......KVDLF......
145531385	......TILRK......IVRLL......YLIQE......IVHRD......GDFGM......KCDIW......
145533733	......NTLKL......ILKLL......FLVTE......IMHGD......ADFGF......QADMW......
145532673	......SLLQE......IVKLY......YLIME......IIHRD......IDLGL......LVDIF......
145534025	......NLYQV......VIKVH......VLVME......LFHRD......ADFGA......KSDIF......
145535778	......TVLSK......ILKLH......YIITE......IIHRD......ADFGF......KSDIW......
145535151	......QPKQQ......LVRFL......WIFQE......IVHSD......IDYGS......KIDVW......
145537860	......NALKA......IMKLC......YIITE......YIHRD......ADFGF......KGDVW......
145537580	......KVMQK......IVQLM......YIIQE......IIHRD......ADFGF......KTDIW......
145535103	......ETMRI......ILQLH......FLVME......IMHRD......ADFGL......SCDIF......
145535610	......--LSQ......VVQLF......FIVQE......IVHRD......TDFGF......SVDIW......
145536800	......KIMQS......VINLI......YLLME......IAHRD......CDVGC......KSDVY......
145536764	......SILKS......LLKLY......YIVTE......IVHRD......ADFGF......KTDLW......
145538107	......KIMQQ......VINLI......YLLME......IAHRD......CDVGC......KSDVY......
145534917	......QIMKS......IVELK......NMILE......VVHRD......GDFGF......KCDIW......
145537301	......LILKS......IIKLY......VLVME......IYHRD......TDFGV......KSDVY......
145537800	......TILKA......IVRLY......YIFLE......IIHRD......ADFGF......KCDVW......
145536486	......QILKI......FLTLK......YLVTD......IIHRD......VDFGL......KVDIF......
145537239	......SILKI......IVKLF......ILILE......FVHRD......IDFGF......NCDMF......
145536383	......NLQQL......VCRTY......YQVIE......ISHRD......IDFGV......KVDIW......
145535812	......QNMKL......VVRFI......YIVME......VMHRD......GDLGF......TADIF......
145538351	......DLMRR......IVKMI......YIILE......IIHRD......IDFGL......KVDVF......
145537247	......RIMRQ......LINLH......YLIID......IMHRD......SDFGV......RCDIF......
145535953	......VIMQT......CLKIY......VIVME......VTHRD......ADLGG......TSDVY......
145536566	......ELMKL......IVKMV......YIIME......IIHRD......IDFGL......KVDMF......
145538417	......NYLQA......IVEYI......YIVLE......IIHRD......CDFNW......GVDIW......
145536187	......SLLEK......IVHFH......YIIME......IIHRD......IDLGL......TVDIF......
145539862	......---KE......VINTY......CVVMD......IIHRD......CDLGL......SVDVW......
145541828	......QVMKR......LIQLY......IVVME......LLHRD......GDFGS......KCDIW......
145538977	......-----......IIRIY......YIVME......YFHRD......IDFGC......EIDIW......
145540940	......EILQE......INRIY......YIVLE......IAHRD......GDFGF......KVDVW......
145540710	......NALKA......IMKLC......YIITE......YIHRD......ADFGF......KGDVW......
145540990	......ELMKM......IVRVE......VLILE......IIHRD......ADLGL......SADIF......
145539866	......QNFKK......LIQIY......YVVME......LAHRD......CDFGV......ECDIW......
145542420	......-----......IIGIY......IVVME......LFHRD......IDFGF......SVDIW......
145541516	......VIMQT......CLKIY......VIVME......VTHRD......ADLGG......--DVY......
145540365	......QCLNK......VQRLH......YLIQE......IMHRD......TDFHL......KVDIF......
145539386	......EILKK......IVKFI......YIITE......IIHRD......ADFGF......KCDIW......

```
145539972   ......RSMKQ......IVKFL......YIISE......IIHRD......ADFGF......KCDIW......
145542325   ......ETMRI......ILQLH......YLVME......IMHRD......ADFGL......VCDIF......
145539658   ......NLYSA......VIKVY......VFAME......LFHRD......VDFGA......KSDVF......
145541413   ......EYQNQ......IITIY......VLIME......LFHRD......IDFGL......KVDIW......
145540411   ......EIMKQ......IVKLI......VLIIE......YIHRD......ADFGF......KCDIW......
145540291   ......RLLYH......IVKFF......LI--E......IVHGD......CDFGF......KCDIF......
145539586   ......QALQE......INKII......HLFLK......WLYRD......IDFGM......SFDIY......
145538718   ......YIMQM......LISLH......ILLME......ITHRD......GGFEE......LADVY......
145541113   ......KIMQK......IVQLL......YIVQE......IIHRD......ADFGF......KTDVW......
145540605   ......QLNRQ......IIKIY......LVFQM......LYHRD......ADFGV......KSDVF......
145541507   ......DLLRK......VIKLF......YLVLE......IIHRD......LDLGL......EADIY......
145540357   ......RIMRD......IVKII......YLIME......YLHRD......ADFGF......KSDIW......
145541389   ......DYQKS......LIRLY......LIVME......IFHRD......IDFGL......SVDIW......
145539656   ......ESMRT......IVKFL......YIISE......IIHRD......ADFGF......KCDIW......
145541754   ......SLLEK......IVHFH......YIIME......IIHRD......IDLGL......TVDIF......
145541826   ......QINQD......FVGNY......LIIEE......RSHND......HDFGA......KSDIY......
145540894   ......KVMQK......IVSLM......YIIQE......IIHRD......ADFGF......KTDIW......
145538971   ......EILQK......FIKLY......YIVTD......IIHRD......IDFGL......KCDIF......
145539416   ......NILKQ......ILKMY......YIITE......IIHRD......ADFGF......KTDLW......
145540758   ......TILKA......IVRLY......YIFLE......IIHRD......ADFGF......KCDVW......
145539874   ......G--KE......LVNIF......SIVME......IAHRD......CDFGT......SVDIW......
145539398   ......RALKS......ILKLF......YIITE......FIHRD......ADFGF......KGDVW......
145540208   ......NLQRL......ICRAY......YQVIE......ICHRD......IDFGV......KVDIW......
145540674   ......QIMRE......LMRLY......YMGLE......IMHRD......ADFGL......VCDVY......
145541928   ......NLQRQ......VCRTY......YQVIE......ISHRD......IDFGV......KVDIW......
145540265   ......SVMQK......VVRLL......YIVQE......IIHRD......GDFGF......KCDIW......
145546827   ......KTISE......LVEII......YIVME......IIHQD......SDFSI......KSDIY......
145543949   ......QMNRQ......IIKIY......LVFQM......LYHRD......ADFGV......KSDVF......
145543725   ......RILRD......IVKII......YLIME......YLHRD......ADFGF......KCDIW......
145543552   ......ESLKK......ILQYI......YIITE......IIHRD......ADFGF......QSDVW......
145545692   ......SILKQ......IIKVL......WIVQE......IVHRD......IDFGF......NSDMF......
145548106   ......EIMKS......IVKLL......YIVTE......IVHRD......TDFGF......KSDIW......
145544479   ......EIMKK......VVKLL......YIITE......IIHRD......TDFGF......KCDVW......
145547693   ......QCMQN......LVQLK......YIFME......YIYRD......IDFGL......KSDIF......
145545141   ......DALKK......VVGFI......YLVME......IIHRD......ADLGF......SSDMF......
145545133   ......SIMST......IVKMA......YIFME......IIHRD......SDFGL......KTDIW......
145545117   ......CALKL......IVKLY......YMVME......IIHRD......ADFGF......KCDIW......
145545139   ......TALSL......VIKLY......IFILE......IIHRD......ADLGF......QADMF......
145543639   ......AILQK......IVRLF......YMFLE......IIHRD......TDFGF......KCDVW......
145546925   ......EILKR......ILEIK......YIVTE......IIHRD......IDFGL......KVDLF......
145543783   ......SIMKE......IVKLL......YVVID......YIHRD......ADFGF......KCDIW......
145547150   ......EILKK......VLKIQ......YIVTE......IIHRD......IDFGL......KVDLF......
145544100   ......ELMRL......IVKMV......YIIME......IIHRD......IDFGL......KVDMF......
145543572   ......AILLR......LLRVY......YIVSE......IIHRD......ADFGF......SSDIW......
145543412   ......MMLKQ......FTKIL......HVGLE......LQYHD......LELC-......KQNVF......
145547443   ......EIYNH......LVAIK......CILMD......VIHRD......TDFGV......KIDVY......
145543945   ......QAMVS......IAELL......YMVMQ......YMHRD......IDFGL......KCDIF......
145546767   ......IALKS......IEKIY......VIIIE......FVHRD......VDFGF......KGDVF......
145544517   ......QALRM......ILKLY......YLLTE......VMHRD......IDFGL......QCDIF......
145546384   ......EVLN-......MIAIS......YIIFE......FICRD......TEFGQ......EQDVY......
145545873   ......SILLR......LMKVY......YIVTE......IIHRD......ADFGF......KSDIW......
145543649   ......RLLYH......IVKFY......LI--E......IVHGD......CDFGF......KCDMF......
145544306   ......NILKS......LLKLF......YIVTE......IVHRD......ADFGF......KTDLW......
145542921   ......DFAPL......----Y......IIIQE......FFHRD......CDFGE......SADIW......
145548116   ......YILKR......VLKLC......ILVTE......YVHRD......IDFGF......VGDIF......
145546641   ......SILQQ......IVKLY......YLFLE......IIHRD......TDFGF......KCDVW......
145543143   ......ELMRK......IVKMI......YIILE......IIHRD......IDFGL......KVDIF......
145545712   ......EIMSK......IVKFV......FIVTE......VIHRD......ADFGF......KCDIW......
145544334   ......KIMQS......VINLI......YLLME......IAHRD......CDVGC......KSDVY......
145547060   ......RKRKI......LVQLL......MVISE......QVHGD......AEFGF......KSDVF......
145548176   ......EIVER......-CEYL......YIAIS......IIHRD......ADYGA......KCDVY......
145548140   ......QILEG......IIKII......YIFLE......IVHRD......IDFGV......QCDVF......
145544168   ......QTLIK......VVRYE......YIISE......IIHRD......IDFGF......KVDVW......
145546669   ......DNKKK......IAQFY......HLFME......VIHND......TDFGE......KSDIF......
145546382   ......ETLGQ......IVKLI......LIVLE......FVHRD......IDFGI......KIDVF......
145551466   ......KTSNQ......IIQYY......CFVME......HIHRD......CDFGF......KVDIW......
145552090   ......NLMRQ......IIKLY......YVCLE......IMHRD......ADFGL......VCDIY......
145548908   ......EALSQ......IMRLY......YLITE......IVHRD......ADFGF......KSDIW......
145553038   ......QITQQ......IVKII......YILME......IIHRD......ADFGL......KVDIW......
```

145551418	......KLLIN......LVRIE......EIIME......IVHRD......TDFDF......KCDIY......
145548511	......KILSQ......IIKLD......SLLLE......IAHND......ADFGF......SADVY......
145551961	......SILLR......LMKVF......YIVTE......IIHRD......ADFGF......KSDIW......
145549277	......KVMQK......IVQLM......YIIQE......IIHRD......ADFGF......KTDIW......
145551566	......AILQK......IVRLY......YMFLE......IIHRD......TDFGF......KCDVW......
145553056	......SILQS......INKIY......YIVLE......IAHRD......GDFGF......QVDIW......
145552583	......NNSRC......IIQEY......TLVME......ISHRD......LDFGV......EIDLW......
145553225	......LILKS......IIRLY......VLVME......IYHRD......SDFGV......KSDVY......
145549590	......QIMSR......LVKFY......YFMME......IVHRD......SDFGF......KTDIW......
145549476	......KLLSE......VIKLF......ILILE......IIHRD......IDLGL......QADMY......
145549780	......EMLNK......LIKIF......YIVME......YAHRD......CDFGT......KVDIW......
145552916	......ATMKD......IAKII......VIILE......YIHRD......SDFGF......KCDLY......
145553257	......RIMRQ......LINLH......YLIID......IMHRD......SDFGV......KCDIF......
145550850	......EILRQ......IIKLI......YLVFE......IVHRD......ADFGF......KCDVW......
145549926	......QIKSQ......LCKML......FIMYE......WVHSD......IDYGS......QIDIW......
145548575	......EALST......IMKLY......YLITE......IIHRD......ADFGF......KSDIW......
145550233	......EVLQK......VVKFI......HFVYE......IIHRD......ADFGF......KADIW......
145548912	......KIHTS......IVRLY......YILLE......IIHRD......CDFGW......KLDSW......
145552380	......SILQQ......IVKLY......YLFLE......IIHRD......TDFGF......KCDVW......
145552202	......EVLKQ......VVRLV......YIVLE......ILHRD......ADLGF......AADMF......
145549430	......QILRR......IQKLL......YILFD......IIHKD......ANFAS......KIDIF......
145550179	......ESLKK......ILQYI......YIVTE......IIHRD......ADFGF......QSDIW......
145549482	......KMFSG......ILKFY......WVILE......IIYCD......SDLGN......QSDLW......
145553042	......GNLSQ......IVNVI......CILME......ITHRD......GDFGL......LVDIW......
145553483	......EICKK......IVELY......YIIFE......VVHRD......GDFGF......KCDIW......
145552282	......KILQT......IAQFY......ILLME......IIHMD......SDFGE......AYDIF......
125624851	......FAMAE......IVGIS......YIVME......IIHRD......TDFGI......QSDIY......
124523284	......QSATS......IVSIY......YIVME......IIHRD......TDFGI......KSDIY......
125718643	......KAMAD......IVRIT......YLAME......IVHRD......TDFGF......QSDIY......
125525258	......RMLHS......VLKFY......WLILE......IIYCD......CDFGL......ASDFW......
125536465	......EILRK......IIEMI......CVVTE......IIHRD......CDFGF......TADLW......
125547865	......EVLRT......LLPVL......ALIYE......IVHCD......GDFGI......SGDVY......
125542182	......AILST......ILRLI......YLILE......IVHRD......GDFGF......KADLW......
125553206	......A----......CADFI......WLIWR......IVHRD......IDLGA......PSDIY......
125569792	......RMLHS......VLKFY......WLILE......IIYCD......CDFGL......ASDFW......
125579189	......EILRK......IIEMI......CVVTE......IIHRD......CDFGF......TADLW......
125595113	......A----......CADFI......WLIWR......IVHRD......IDLGA......PSDIY......
125809355	......SLNLL......IVHMI......ILIME......IFHRD......IDFGC......RADAW......
125809367	......SLNLL......IVHMI......ILIME......IFHRD......IDFGC......RADAW......
125824800	......KILKE......IVSLL......YLVME......IIHRD......ADFGF......KADLW......
125836027	......AYMLM......VIEMY......SLVME......VSHND......IDFGC......-ANIR......
125836023	......AYMLM......VIEMY......SLVME......VSHND......IDFGC......-ANIR......
125837754	......KILKE......IVALY......FLVME......IIHRD......ADFGF......KADLW......
126275020	......SILKS......IVGLL......HIVMD......LVHRD......ADFGF......KADLW......
126432628	......QNAAA......IVAVY......YIVME......IIHRD......MDFGI......RSDVY......
126458653	......NRYLE......IVKAY......YILLE......VVHLD......GDMGI......ASDVY......
126272490	......EILKA......IVELK......YLIME......ISHLD......ADFGF......RVDLW......
126272374	......QIMKK......VVKAC......ILAME......IIHRD......IDLGY......TVDYW......
126314263	......KILKE......IVALY......FLVME......IIHRD......ADFGF......KADLW......
126337822	......EIMRG......IIQML......VVVTD......ILHRD......CDFGF......TADLW......
126341610	......RLTHE......IVTFH......WLVVE......ILFCD......SNFSL......ASDLW......
126648214	......QILAK......IARLY......FFTME......IVHLD......LDFGV......VSDVY......
126649671	......LSATS......IVSVY......YIVME......IIHRD......TDFGI......QSDIY......
126649810	......QILQQ......LPKVH......YFIMD......IVHRD......IDFGL......ISDLY......
126659780	......KILSQ......IIKTY......VIILE......IIHKD......IDFGI......HTDFY......
126659478	......IITRH......IVKSL......ALIM-......IIHKD......IDFSI......RTDFY......
126660483	......QILAK......VVKVR......YIVME......IYHLD......IDFGA......QSDLF......
126661109	......EITRC......IIKVY......VMLLE......IIHKD......IDFGL......RSDFY......
126660132	......SCLVS......IVACE......YLVME......IIHCD......TDFGI......ACDIY......
126661391	......RVLRS......IPRYL......CLVQE......IIHGD......IDFGF......ASDLY......
126656911	......TVSAL......IVRVR......FYVME......IIHRD......LDFGI......RSDIY......
126734438	......RSIAK......IVGVH......YMVLD......LLHRD......IDFGA......SSDLY......
126734022	......RSLAK......IVGVH......YMALD......ILHRD......IDFGA......SSDLY......
126741159	......ENIRE......VVRYS......YLLMD......IVHRD......IDFGI......RSDIY......
153196329	......QSATS......IVSVY......YIVME......IIHRD......TDFGI......KSDIY......
153167664	......QSATS......IVSVY......YIVME......IIHRD......TDFGI......KSDIY......
139438760	......QAAAG......IVGVY......YIVME......IIHRD......MDFGI......TSDIY......
134098292	......RSAAK......VVAVY......YLVMQ......MVHRD......ADFGL......RTDIY......
134096662	......QNAAA......IVAVY......YIVME......IIHRD......MDFGI......RSDVY......
134099731	......HAAAR......VIPIH......YLDMR......LVHRD......VDFGI......RADVY......
134097137	......RNTSA......IVPLY......YIAMR......LVHLD......ADFGL......RTDLY......

```
157073917    ......EILKT......ILELK......FLITE......ISHLD......ADFGF......RVDLW......
148237566    ......EILKT......IVQLK......YLILE......ISHLD......SDFGF......RVDLW......
154333828    ......EILTK......IIMLF......VVVME......IIHRD......ADFGF......RVDLW......
145257504    ......SILKQ......IVALL......HLVME......LIHRD......ADFGF......KADLW......
154342446    ......RMASR......LVNVF......YLVMD......IVHRD......ADFGD......YSDLW......
154340279    ......QLLRS......IIEFH......WIITE......VVYND......HDFSL......ASDLW......
154344945    ......DLLRE......IVNIV......YFSME......IIHRD......IDFGF......TSDIW......
146080679    ......EILTK......IIMLF......VVVME......IIHRD......ADFGF......RVDLW......
145237846    ......RIARD......TVEFQ......SLVLE......IAHRD......ADFGV......AVDIW......
146091705    ......QLLRS......VIEFH......WIITE......VVYND......HDFSL......ASDLW......
134278075    ......RLLAQ......LVKVL......YMVMP......CFHRD......LDFGA......QTDLY......
144574973    ......HLIKN......VPKIF......YMVLE......IIHRD......IDFGI......SVDIY......
145347961    ......EVMRR......ILRFI......YIVLE......IVHRD......ADFGF......KADLW......
145354871    ......DIMKT......VIKML......VVVME......VIHRD......CDFGL......AVDLW......
145353911    ......QVMKA......VLKFI......WLILE......LVHCD......CGFGL......ATDLW......
145353983    ......QVMKA......VLKFI......WLILE......LVHCD......CGFGL......ATDLW......
145609493    ......TATTK......FVESM......YVTME......FAHRD......GGFGI......RADVW......
145225811    ......QAAAG......VVPIH......YLDMR......LIHRD......IDFGL......RSDIY......
145590326    ......NRYLE......VVRAY......YILLE......IIHLD......GDMGI......SSDIY......
145332831    ......IILRK......IIRFI......NLVLE......IIHRD......ADFGF......KADLW......
146283287    ......HIVAS......IITIH......YLAME......LVHRD......TDFGV......RSDIY......
146308202    ......HTVAS......IITIY......FLAME......LVHRD......SDFGV......RSDIY......
146318083    ......RAMAE......IVRIS......YLAME......IVHRD......TDFGF......QSDIY......
148926991    ......KILYK......IVRIF......YIIME......IIHRD......IDFGL......QTDIY......
153816012    ......DILKR......LPSII......LIVMD......IIYRD......IDFGT......RTDIY......
153815683    ......QAAAG......VVNVY......YMVME......IIHRD......TDFGI......KSDLY......
148980059    ......WAGML......IMKVY......YQVCE......MVHRD......IDLGL......RSDLF......
146184466    ......NMLQK......VVNLL......YLVLN......CISND......SDLGL......YSDEY......
146185494    ......KALMT......IVKYV......IIIME......FIHRD......SDFGL......KVDIW......
146185419    ......KIFNL......ILKYY......WAIFE......IIYCD......CDFGL......QSDLW......
146184454    ......KIMKL......VFKSI......YISKK......LIHSD......CDYGE......KYDIY......
146185848    ......ENLQK......VVKLF......YMFLE......IIHRD......ADFGF......KCDVW......
146186035    ......QIMMS......VLRLL......YFILE......VFHRD......ADFGL......KNDIW......
146186319    ......EICLK......ILRIY......YLFLE......IIHRD......SDFGL......KCDVW......
146184755    ......RIMQQ......VVQLY......YVVFE......IIHRD......CDFGL......KSDSY......
146184459    ......NMLEK......VVNLL......YLVLS......CISND......SDLGL......KSECF......
146184006    ......EIMQD......IVQLF......YIILE......IIHRD......SDFGF......KCDIW......
146184263    ......NILRQ......CLKLV......YILTE......IMHRD......IDFGL......KCDLF......
146183701    ......KIMKL......VFKTI......YQISK......LIHSD......CDYGE......KYDIY......
146183572    ......ALQQK......TVELY......YLILE......IIHRD......ADFGA......EVDIY......
146183780    ......EILKT......VISLL......YLVLT......CLLND......CDFGT......KVDEY......
146182048    ......TKYKQ......LAQIH......YIVQE......LIHGD......TDFGL......KMDTY......
146181909    ......KILKL......VFKSI......YQISK......LIHSD......CDYGE......KYDIY......
146181438    ......ECFKQ......VVKLI......YLVFQ......CSVLD......SKFVL......KRESF......
146180930    ......RILDC......KISCQ......YIITE......IMHRD......GDFGI......QIDYW......
146181406    ......CLLKF......FIQLQ......ILVMD......ICHRD......GDFSE......LSDLY......
146181246    ......NILQK......IVKIF......YIILE......IVHRD......GDFGF......LVDIW......
146181525    ......NVMKK......IVKLL......VIILE......YIHRD......SDFGF......KSDIW......
146180855    ......QIITQ......IVEAY......YIVSE......VIHRD......ADFGL......KADIY......
146180683    ......RIMQQ......VVQLY......YVVFE......IIHRD......CDFGL......KSDSY......
146180928    ......EVSKY......ICRIF......FVIME......IVHRD......GDFGL......EVDIW......
146167959    ......KTLSQ......VVSFI......YFVYE......IMHRD......ADFGF......KADIW......
146168000    ......AILQK......IVSLY......YMFLE......IIHRD......SDFGF......KCDVW......
146164665    ......EV-KE......FLKCY......FIVYE......IVHCD......IDFGS......SVDMW......
146164888    ......NIMKG......LIDTI......SIIME......IIHLD......SDFGT......QSDVY......
146164531    ......EVLEK......VIKLQ......YLVLD......IAHRD......SDFGC......KSDIF......
146165315    ......QTLTK......VITFI......YLVYE......ILHRD......ADFGF......KADIW......
146163546    ......RIMQQ......VVQLY......YVVFE......IIHRD......CDFGL......KSDSY......
146163446    ......NILEK......MIKFY......NIVME......IAHRD......CDVGS......KSDVF......
146164015    ......-LLKE......VFRSI......YQISK......FIHSD......CDYGD......KYDIY......
146163549    ......KMLEV......IIKVF......FYVME......VVHRD......CDLAF......LSDIF......
146162247    ......QVAQE......ICELK......YLISE......CIHRD......CDFGF......KADIY......
146162733    ......RIMQQ......VVQLY......YVVFE......IIHRD......CDFGL......KSDSY......
146161621    ......QLSKV......VIKFY......FIITE......ICHRD......IDFGE......KSDVF......
146161691    ......RIIEQ......LVSFE......YFIME......ILHRD......ADFGL......QIDMF......
146161148    ......TIQKI......TVQVI......IIIQE......CMHRD......GDFGF......EADMF......
148244779    ......WVGQR......VVKIY......AYAMV......MLHCD......IDFGS......ASDQF......
153954000    ......TAAAS......IVNIY......YIVME......IIHRD......TDFGI......RTDIY......
146413787    ......SILKN......IVGLI......HLVMD......LVHRD......ADFGF......KADLW......
```

```
149234796 ......ENMKM......IMPIL......TLILP......IVHRD......SDFGY......-LDLW......
149237536 ......QILKT......IVKLL......HLVMD......LVHRD......ADFGF......KADLW......
147798769 ......EILRK......IIEML......CVVTE......IIHRD......CDFGF......TADLW......
147838284 ......FILKK......IIRLH......HLVLE......LIHRD......ADFGF......KADLW......
147856746 ......EILRT......IIRLL......FLVLE......LIHRD......GDFGF......KADLW......
148368824 ......YVHQI......FVKLH......YWTMD......IIHND......CDYGL......SFDWW......
148359976 ......EFCRK......I---K......YLTMK......IIHRD......FDYGL......KADIF......
148380458 ......TSVAS......IVNIY......YIVME......IIHRD......TDFGI......RTDIY......
148377478 ......AILQK......FPYYV......YLVME......IIHRD......LDFGL......KTDIY......
148667928 ......EIMRG......IVHML......VVVTD......ILHRD......CDFGF......TADLW......
148667929 ......EIMRG......IVHML......VVVTD......ILHRD......CDFGF......TADLW......
148667927 ......EIMRG......IVHML......VVVTD......ILHRD......CDFGF......TADLW......
148667926 ......EIMRG......IVHML......VVVTD......ILHRD......CDFGF......TADLW......
148667925 ......EIMRG......IVHML......VVVTD......ILHRD......CDFGF......TADLW......
148677215 ......RLTHE......IVTFH......WLVVE......ILFCD......SNFCL......TSDLW......
148688054 ......KILKE......IVALY......YLVME......IIHRD......ADFGF......KADLW......
148693965 ......EILKG......IVQLK......YLIME......ISHLD......ADFGF......RVDLW......
148745708 ......KILKE......IVALH......YLVME......IIHRD......ADFGF......KADLW......
156094756 ......KIVQK......VVKIY......FIVME......IIHRD......IDF--......KTDIY......
156097522 ......KILSK......IIKLC......WLVTK......IIHRD......CDFGD......KWDIW......
156099173 ......NIHIH......IVKLF......LVGME......VHHCD......SDFGL......KIDIW......
149177804 ......DIAVQ......IVKTF......FLVME......WIHRD......IDFGL......KIDIF......
149177838 ......EKLAE......IISLQ......YYMME......ILHCD......CDFGQ......RWDVY......
149177724 ......KSAAR......ISRVF......FIAFE......VVHRD......VDLGL......RSDIY......
149178309 ......QAVAS......IVRAY......FLVME......MVHRD......LDLGL......RADIY......
149178815 ......RATAA......VVNIY......FLVME......LIHRD......TDFGL......RSDLF......
149177656 ......QAAAA......IVTIH......YLVME......LIHRD......TDFGL......RSDLF......
149178193 ......QAAAH......IVSVY......YIALE......IVHRD......TDLGL......RSDIY......
149175687 ......RVAAT......IVRAY......FLVME......IVHRD......LDLGL......RSDIY......
149176002 ......RTSAR......VVRIH......YLVME......LIHRD......TDFGL......RADLF......
149174252 ......RVAAT......IVRAY......FLVME......IVHRD......LDLGL......RSDIY......
149175165 ......QATAG......TIEIY......YYVME......LIHRD......LDFGL......RSDIY......
149173362 ......QLTSQ......TIAIY......YYAME......VIHRD......LDFGL......RSDLY......
149176058 ......RAAAT......ICPVY......YITMA......VIHRD......MDFGL......QADIY......
149173376 ......EVCKQ......IVPIY......YFTME......YTHRD......IDFGL......RSDLY......
149174777 ......QTLAQ......IVRVF......LLYMQ......VLHRD......ADFNI......RSDLY......
149175637 ......ELIGQ......IVKAT......FLVME......LIHRD......LDLGL......RADIY......
149182348 ......QSATS......IVSIY......YIVME......IVHRD......TDFGI......KSDIY......
149179700 ......DILNR......FPKLI......YLIMD......IVHRD......IDFGL......RADFY......
149016118 ......EIMRG......IVHML......VVVTD......ILHRD......CDFGF......TADLW......
149041798 ......EILKG......IVQLK......YLIME......ISHLD......ADFGF......RVDLW......
158508716 ......QIMRR......VVAAR......LLAME......IIHRD......IDLGY......TVDYW......
157820595 ......KILKE......IVALY......YLVME......IIHRD......ADFGF......KADLW......
149194517 ......WIARR......FPKAV......YYVME......LVHGD......VDFG-......SSEIF......
149197846 ......LLAAS......LVRIY......FLAME......IIHRD......ADLGI......RSDIY......
149200153 ......YIMDH......LPRLA......YLAMN......AIHRD......IDWGL......RTDIY......
149199558 ......RLAAA......VIRIY......YLVME......IIHRD......ADLGI......RSDIY......
149200477 ......QVTSQ......IVPIY......FYAMK......VVHRD......MDWGI......RADIY......
149196519 ......KIGYQ......LAQVF......YMVME......YLYRD......VDYGL......RADMY......
149198961 ......KSTAN......TVTLY......YYVME......MVHRD......LDFGI......LADIY......
149196895 ......RLTAS......ITTIY......FFTME......VLHLD......CDWGI......KTDIY......
149196280 ......RSAAS......IVQAY......YFGME......IIHRD......MDLGL......RGDLY......
149197465 ......AIGKK......FVNMH......CILMQ......LIHRD......MDYGI......RADIF......
149196490 ......LIMAY......IIPVY......FYSMP......IIHRD......LDWGL......STDVF......
149196504 ......QLTAQ......IIPIY......YFAMK......VIHRD......MDWGI......PTDVY......
149197228 ......RITCR......TICIY......YYAME......LIHRD......LDFGL......SIDIY......
149196333 ......QAAAA......IVQAY......YFAME......LTHGD......ADLGL......RADIY......
149195665 ......QNSAA......IVTTY......FITMQ......MIHRD......MDFGL......HTDMY......
149197702 ......RASAK......IVQAY......YLAME......LVHRD......MDLGL......RGDIY......
149200229 ......QILAQ......IVPVY......YMAMG......ILHRD......MDMGI......RTDLY......
149195590 ......RLVAH......IVTIY......YFTMD......IIHLD......FDWGL......QTDIY......
149200640 ......QITSQ......IVPIY......FYVMK......VVHRD......MDWGI......RADIY......
149198184 ......RLAAS......LVRVM......YYVME......IIHRD......GDLGL......RADIF......
149197556 ......RITAS......IVPVH......FFTMK......VLHLD......CDWGL......TTDIY......
149196762 ......KALFK......IVKML......YIVFE......IIHRD......TDFGI......QSDIY......
149195646 ......ELLSS......FIHVF......YLAMD......LIHRD......TDLGI......RCDIY......
149260414 ......RLTHE......IVTFH......WLVVE......ILFCD......SNFCL......TSDLW......
149260517 ......RLTHE......IVTFH......WLVVE......ILFCD......SNFCL......TSDLW......
149374554 ......EAAGR......IVTVY......FIAMD......IIHRD......TDFGI......ATDTY......
149369888 ......EILKK......LVAYC......YVILD......IIHND......IDFGY......QSDVF......
149388991 ......SILKS......IVGLL......HIVMD......LVHRD......ADFGF......KADLW......
149410746 ......QIMRR......VVAAR......LLAME......IIHRD......IDLGY......TVDYW......
```

```
149638554 ......QIMKK......IAPLL......TVAMV......IIHRD......IDLGY......TVDYW......
149641770 ......KILKE......IVALY......FLVME......IIHRD......ADFGF......KADLW......
149725008 ......KILKE......IVALY......FLVME......IIHRD......ADFGF......KADLW......
154485076 ......QSAAS......IVNVY......YIVME......IVHRD......TDFGI......KSDIY......
154484410 ......RVFSL......IVSYY......YILME......IIHRD......GDFGV......SVDNY......
154484783 ......EVLKN......LPKIF......FLVME......IVYRD......IDFGI......RADIY......
149909183 ......QLVGL......IVRLL......YAVFE......VIHRD......LDFGI......KTDLY......
149923728 ......QAMAK......VVTVH......FVAME......IVHRD......ADFGL......RSDQF......
149924677 ......EVTTR......TIRVF......YMVME......IFHRD......LDFGI......RTDIY......
149922348 ......QSASA......VVEIS......FFVME......IVHRD......LDFGI......RADIY......
149921899 ......HNAAS......VVSVV......FLVME......ILHRD......LDFGI......RSDLY......
149924820 ......QAMAR......TVTVH......FIAME......IVHRD......LDFGL......RGDQF......
149923455 ......QALAR......VVPIY......YLVME......IVHRD......LDFGL......RSDQF......
149924577 ......RIAAR......VVEIL......FMVME......LVHRD......GDFGI......RTDLF......
149921776 ......ALAHS......VVRIH......FFSME......IVHRD......MDFGV......QSDLY......
149920864 ......QLLRG......VVRAL......VLVLE......IIHRD......ADFGI......RSDLY......
149920577 ......RAASM......IVDIM......FFVME......IIHRD......LDFGI......RTDVY......
149920440 ......TILRS......VVRTF......YMLME......IVHRD......LDFGL......ASDVY......
149921198 ......RALCD......LIELL......FFTME......CIHRD......LDFGL......AADLY......
149920936 ......RAAAK......VVSVL......YIAME......IIHRD......LDFGI......RADLW......
149920937 ......RAMAA......VVEVF......YLVME......VVHRD......LDFGI......RADLY......
149921291 ......KAMAS......VIAIH......FIAME......IVHRD......GDFGL......RADQF......
149919159 ......RAASS......IVEIY......FFCME......IVHRD......VDFGI......RLDIY......
149921360 ......RTASS......VVEIT......YLVME......VIHRD......LDFGL......RTDIY......
149921422 ......TSAGR......ICDIL......YIVME......IIHRD......MDFGI......RTDIW......
149919485 ......RAASM......VVEIT......FFIME......IIHRD......LDFGI......RVDVY......
149918092 ......TALAQ......IVAVH......YMVME......IVHRD......LDFGV......VGDLW......
149919775 ......VVAAN......LVRVL......WFAME......IAHCD......IDFGI......RVDLY......
149921142 ......QAMAR......VVAIY......FIAME......LIHRD......TDFGL......RSDQF......
149919471 ......QSAAR......VVPIY......FVAME......LAHRD......VDFGL......RSDQF......
149917151 ......RLSLH......VVSVF......FIVME......VVHRD......TDFGL......RTDVF......
149921093 ......KIALS......IVQVF......YLAME......IVHRD......LDFGI......RSDLY......
149919540 ......HSASR......CVRVT......YLVME......VVHRD......VDFGI......RTDLY......
149919622 ......GLLQA......VVRAR......VLLLD......IIHRD......ADFGI......RSDLY......
149918858 ......RVAAT......VVHTY......FIAME......LVHRD......VDFGI......RADIF......
149920684 ......QSLAR......VVPVY......FVAME......IIHRD......LDFGL......RADQY......
149919484 ......RVAST......VIDII......YFVME......IVHRD......LDFGV......RSDLY......
149921363 ......QATGQ......IVEIL......FFVME......VVHRD......LDFGI......RTDVY......
149921175 ......QALAR......VVAIH......YLVMS......LVHRD......VDFGL......RSDQF......
149917998 ......RAVSL......VVGVV......YLVME......IVHRD......LDFGL......RVDIY......
153812152 ......STMEY......VVSVE......YIFME......IIHRD......GDFGI......RVDIY......
153809819 ......QAAAG......IVNVF......YIVME......IVHRD......TDFGI......KSDIY......
150007683 ......KILSE......IVNVL......YIAME......ILHLD......IDFGV......EGDIY......
157375897 ......WIGHR......VVKLL......YYVTE......TIHQD......IDFGS......SADLF......
150385685 ......KLVAN......IVQIY......YFVLE......IVHCD......TDFGI......RSDIY......
150385312 ......RAAGK......IVQAY......YFAME......LVHQD......ADLGL......RSDIY......
154497986 ......QAVAM......IVAVY......YIVME......IIHRD......ADFGI......RSDIY......
154286726 ......QNIKS......IIEFV......QLIME......ITHRD......ADFGL......KVDVW......
152981619 ......RAAGR......IVTIY......YIAME......VVHRD......VDFGI......RTDIY......
152989942 ......---LT......LVRLI......YEIYE......IYHRD......CDFGI......KSDYW......
152993076 ......WMAKR......FPKAV......YYIMS......LVHGD......VDFG-......QTEVY......
152991175 ......WNATR......FPKAV......YYIME......LVHGD......VDFGF......SSEIF......
157129164 ......YAHAV......VVRYY......LIQNE......LVHMD......GDLGH......KADIF......
157112423 ......RRYEQ......CVKLY......FMQME......LIHLD......ADFGL......SVDIF......
157694528 ......YAHAV......VVRYY......IIQNE......MVHLD......GDLGH......KADIF......
109471950 ......YAHAV......VVRYY......VIQNE......MVHLD......GDLGH......KADIF......
109473386 ......YAHAV......VVRYY......VIQNE......MVHLD......GDLGH......KADIF......
113197962 ......GSHEK......CVRLE......YLQTE......LVHLD......GDFGL......AADVF......
115444073 ......QALAA......IVGYF......FIQME......IAHLD......GDFGC......KVDIF......
114154836 ......YAHAV......VVRYY......IIQNE......MVHLD......GDLGH......KADIF......
115386078 ......DVLKS......IISFM......YIQTE......FIHLD......ADFGM......PADIF......
114616430 ......YAHAV......VVRYY......IIQNE......MVHLD......GDLGH......KADIF......
114636085 ......YAHAV......VVRYF......LIQNE......LVHMD......GDLGH......KADIF......
114660534 ......GSHEK......CVRLE......YLQTE......LVHLD......GDFGL......AADVF......
114660556 ......GSHEK......CVRLE......YLQTE......LVHLD......GDFGL......AADVF......
114660546 ......GSHEK......CVRLE......YLQTE......LVHLD......GDFGL......AADVF......
114660552 ......GSHEK......CVRLE......YLQTE......LVHLD......GDFGL......AADVF......
114660544 ......GSHEK......CVRLE......YLQTE......LVHLD......GDFGL......AADVF......
114660554 ......GSHEK......CVRLE......YLQTE......LVHLD......GDFGL......AADVF......
114660548 ......GSHEK......CVRLE......YLQTE......LVHLD......GDFGL......AADVF......
```

115709928	......KKHES.....CVEFY.....YIQTE.....LLHLD.....GDFGL.....HADVY.....
115952027	......KKHES.....CVEFY.....YIQTE.....LLHLD.....GDFGL.....HADVY.....
116059964	......EAAAL.....IVRYY.....YIQME.....IIHLD.....GDFGL.....SADVF.....
169847227	......EVLKH.....VLAYI.....FIQTE.....VIHLD.....GDFGM.....AADVF.....
121698099	......DALKA.....VISLV.....YIQTE.....FIHLD.....ADFGM.....PADIF.....
119480519	......DVLKA.....VISFV.....YIQTE.....FIHLD.....ADFGM.....PADIF.....
119604383	......YAHAV.....VVRYY.....IIQNE.....MVHLD.....GDLGH.....KADIF.....
119921578	......YAHAV.....VVRYY.....IIQNE.....MVHLD.....GDLGH.....KADIF.....
123498760	......EMYKT.....VMKYY.....FIQME.....YMHLD.....SDFGT.....KTDIF.....
154421343	......-----.....LVKSY.....MLKME.....WMHLD.....ADFGT.....TTDIF.....
145483909	......QALAY.....IIRYY.....YLQME.....ITHFD.....ADLGL.....KSDIF.....
145508293	......QALAY.....IIRYY.....YLQME.....ITHFD.....ADLGL.....KSDIF.....
145537478	......YLNAK.....LVRYF.....YLLME.....ISHLD.....ADLGL.....KTDIF.....
145541036	......YLNQK.....LVRYF.....YLLME.....ITHLD.....ADLGL.....KTDIF.....
145546342	......CILAN.....IVQYY.....YLVME.....LVHMD.....ADFGL.....KVDIF.....
125537987	......QALAA.....IVGYF.....FIQME.....IAHLD.....GDFGC.....KVDIF.....
125580724	......QALAA.....IVGYF.....FIQME.....IAHLD.....GDFGC.....KVDIF.....
126332153	......YAHAV.....VVRYF.....LIQNE.....LVHMD.....GDLGH.....KADIF.....
126335329	......GGHEK.....CVRLE.....YLQTE.....LAHLD.....GDFGL.....AADVF.....
126340889	......YAHAV.....VVRYY.....IIQNE.....LVHLD.....GDLGL.....KGDVF.....
134026290	......YAHAV.....VVRYY.....IIQNE.....LVHLD.....GDLGH.....KADIF.....
154336289	......SVMMV.....IVQLS.....YMQLE.....IAHVD.....SDFGC.....AGDMF.....
154341334	......YALSS.....IVRYF.....FVRLE.....VVHMD.....CDFGL.....PADMY.....
146093375	......YALSS.....IVRYF.....FVRLE.....VVHMD.....CDFGL.....PADMY.....
146098786	......SVMMV.....VVQLS.....YLQLE.....IAHVD.....SDFGC.....AGDMF.....
145235908	......DILKS.....IISFM.....YIQTE.....FIHLD.....ADFGM.....PADIF.....
156717916	......YAHAV.....VVRYY.....IIQNE.....LVHMD.....GDLGH.....KADIF.....
148230222	......YAHSV.....VVRYY.....MIQNE.....MVHMD.....GDLGH.....KADIF.....
145351668	......QVLAS.....IVRYQ.....YIQME.....LAHMD.....GDWGR.....RADIF.....
145351068	......EAAAL.....IVRYF.....YIQME.....IIHLD.....GDLGL.....SADVF.....
145613195	......LVLRS.....ILQYV.....YIQTE.....FIHLD.....GDFGM.....PSDIF.....
147905388	......WNHEE.....ILGFI.....YIQTE.....FAHLD.....GDFGL.....AADIF.....
146414373	......EALRV.....LVYFI.....YIMTE.....YLHLD.....GDFGL.....FADIF.....
149239600	......EALRN.....LVYFI.....YIMTE.....YLHLD.....GDFGL.....FADIF.....
148681637	......YAHAV.....VVRYY.....VIQNE.....MVHLD.....GDLGH.....KADIF.....
148681638	......YAHAV.....VVRYY.....VIQNE.....MVHLD.....GDLGH.....KADIF.....
148690317	......GGHEK.....CVRLE.....YLQTE.....LVHLD.....GDFGL.....AADVF.....
149065332	......YAHAV.....VVRYY.....VIQNE.....MVHLD.....GDLGH.....KADIF.....
150864847	......EALRV.....LVYFI.....YIMTE.....YLHLD.....GDFGL.....FADIF.....
149409501	......YAHAV.....VVRYF.....LIQNE.....LVHMD.....GDLGH.....KADIF.....
149447274	......YAHAV.....VVRYY.....IIQNE.....LVHLD.....GDLGH.....KADVF.....
149525629	......GGHEK.....CVHLE.....YLQTE.....LVHLD.....GDFGL.....AADVF.....
149720028	......YAHAV.....VVRYF.....LIQNE.....LVHMD.....GDLGH.....KADIF.....
149747598	......YAHAV.....VVRYY.....IIQNE.....MVHLD.....GDLGH.....KADIF.....
149751109	......GGHEK.....CVRLE.....YLQTE.....LVHLD.....GDFGL.....AADVF.....
154281133	......GC---.....VVRVY.....RILYA.....IIHKD.....GDFGL.....KLDIY.....
157134355	......EMLKK.....IVRFY.....VLVTE.....IIHRD.....GDLGL.....AVDVY.....
109095041	......EMLKG.....IVRFY.....VLVTE.....IIHRD.....GDLGL.....SVDVY.....
109095037	......EMLKG.....IVRFY.....VLVTE.....IIHRD.....GDLGL.....SVDVY.....
109095039	......EMLKG.....IVRFY.....VLVTE.....IIHRD.....GDLGL.....SVDVY.....
109112348	......EMLKG.....IVRFY.....VLVTE.....IIHRD.....GDLGL.....SVDVY.....
109130874	......EMLKG.....IVRFY.....VLVTE.....IIHRD.....GDLGL.....SVDVY.....
109130872	......EMLKG.....IVRFY.....VLVTE.....IIHRD.....GDLGL.....SVDVY.....
109504743	......EMLKG.....IVRFY.....VLVTE.....IIHRD.....GDLGL.....SVDVY.....
110763235	......EMLKG.....IVRFY.....VLVTE.....IIHRD.....GDLGL.....SVDVY.....
111154405	......HLLKT.....IMKFY.....NFITE.....IIHRD.....GDLGL.....LVDIY.....
115472925	......HLLKT.....IMKFY.....NFITE.....IIHRD.....GDLGL.....LVDIY.....
115470927	......HLLAA.....IVRLH.....NFVTE.....IIHRD.....GDLGL.....LADVY.....
114642763	......EMLKG.....IVRFY.....VLVTE.....IIHRD.....GDLGL.....SVDVY.....
114642775	......EMLKG.....IVRFY.....VLVTE.....IIHRD.....GDLGL.....SVDVY.....
114642769	......EMLKG.....IVRFY.....VLVTE.....IIHRD.....GDLGL.....SVDVY.....
114642765	......EMLKG.....IVRFY.....VLVTE.....IIHRD.....GDLGL.....SVDVY.....
114642771	......EMLKG.....IVRFY.....VLVTE.....IIHRD.....GDLGL.....SVDVY.....
114642773	......EMLKG.....IVRFY.....VLVTE.....IIHRD.....GDLGL.....SVDVY.....
114642777	......EMLKG.....IVRFY.....VLVTE.....IIHRD.....GDLGL.....SVDVY.....
114642767	......EMLKG.....IVRFY.....VLVTE.....IIHRD.....GDLGL.....SVDVY.....
114688771	......EMLKG.....IVRFY.....VLVTE.....IIHRD.....GDLGL.....SVDVY.....
114688769	......EMLKG.....IVRFY.....VLVTE.....IIHRD.....GDLGL.....SVDVY.....
115679126	......EMLKG.....IVSFY.....VLVTE.....IIHRD.....GDLGL.....AVDVY.....
115725069	......EMLKG.....IVSFY.....VLVTE.....IIHRD.....GDLGL.....AVDVY.....
116059049	......EILKR.....VLKFY.....NFITE.....IVHRD.....GDLGL.....RVDIY.....

```
118083044    ......GMLKG......IVRFY.....VLVTE.....IIHRD......GDLGL......SVDVY......
118103021    ......EMLKG......IVRFY.....VLVTE.....IIHRD......GDLGL......AVDVY......
147899537    ......GMLKG......IVRFY.....VLVTE.....IIHRD......GDLGL......SVDVY......
119583261    ......EMLKG......IVRFY.....VLVTE.....IIHRD......GDLGL......SVDVY......
119583262    ......EMLKG......IVRFY.....VLVTE.....IIHRD......GDLGL......SVDVY......
119583264    ......EMLKG......IVRFY.....VLVTE.....IIHRD......GDLGL......SVDVY......
119583263    ......EMLKG......IVRFY.....VLVTE.....IIHRD......GDLGL......SVDVY......
119609360    ......EMLKG......IVRFY.....VLVTE.....IIHRD......GDLGL......SVDVY......
119609364    ......EMLKG......IVRFY.....VLVTE.....IIHRD......GDLGL......SVDVY......
119609359    ......EMLKG......IVRFY.....VLVTE.....IIHRD......GDLGL......SVDVY......
119900779    ......EMLKG......IVRFY.....VLVTE.....IIHRD......GDLGL......SVDVY......
119923285    ......EMLKG......IVRFY.....VLVTE.....IIHRD......GDLGL......SVDVY......
123456394    ......SQIK-......LLQIS.....VYVTE.....IIHND......GIPSF......RSDVW......
123495350    ......SILHQ......ILKIF.....IFITE.....VIHRD......GDFGL......KADIW......
145479529    ......NILNN......IISLI.....IFITE.....VIHRD......GDFGL......PVDIY......
145489586    ......AILKS......IIRFI.....VFITE.....VIHRD......GDLGL......KVDIY......
145510905    ......SILSS......IINFI.....IFITE.....IIHRD......GDLGL......KVDIY......
145513088    ......SILNN......IISFI.....IFITE.....VIHRD......GDFGL......SVDIY......
145528999    ......SILSS......IINFV.....VFITE.....IIHRD......GDLGL......KVDIY......
145530113    ......TILKT......IINFI.....VFITE.....IIHRD......GDLGL......KVDIY......
145534047    ......SILNN......IISFI.....IFITE.....VIHRD......GDFGL......SVDIY......
145541155    ......AILKT......IISFV.....VFITE.....IIHRD......GDLGL......KVDIY......
125535867    ......QLLKT......IEKMF.....NIITE.....IIHRD......GDFGL......LVDIY......
125540642    ......HLLKS......VMKFY.....NVITE.....IIHRD......GDLGL......LVDIY......
125535542    ......SLLAD......IIAFH.....NFITE.....VIHRD......GDLGL......GVDVY......
125550539    ......RLLKT......IIKFY.....NFITE.....VIHRD......GDLGL......LVDIY......
125558881    ......HLLKT......IMKFY.....NFITE.....IIHRD......GDLGL......LVDIY......
125557483    ......HLLAA......IVRLH.....NFVTE.....IIHRD......GDLGL......LADVY......
125583223    ......HLLKS......VMKFY.....NVITE.....IIHRD......GDLGL......LVDIY......
125592542    ......RLLKT......IIKFY.....NFITE.....VIHRD......GDLGL......LVDIY......
125812195    ......EMLKC......IVRFY.....ILVTE.....IIHRD......GDLGL......AVDVY......
125829868    ......EMLKG......IVRFY.....VLVTE.....IIHRD......GDLGL......AVDVY......
125830419    ......EMLKG......IVRFY.....LLVTE.....IIHRD......GDLGL......AVDVY......
125852180    ......EMLKG......IVRFY.....VLVTE.....IVHRD......GDLGL......SVDVY......
125854785    ......GMLKG......IVRFY.....VLVTE.....IIHRD......GDLGL......SVDVY......
125950869    ......EMLKG......IVRFY.....VLVTE.....IIHRD......GDLGL......SVDVY......
126253824    ......EMLKG......IVRFY.....VLVTE.....IIHRD......GDLGL......SVDVY......
126336546    ......EMLKG......IVRFY.....VLVTE.....IIHRD......GDLGL......SVDVY......
126340106    ......EMLKG......IVRFY.....VLVTE.....IIHRD......GDLGL......SVDVY......
126340108    ......EMLKG......IVRFY.....VLVTE.....IIHRD......GDLGL......SVDVY......
145349684    ......EILKR......VLKFY.....NFITE.....IVHRD......GDLGL......RVDIY......
147781463    ......NLIKS......IIKCY.....NIITE.....IIHRD......GDLGL......LVDIY......
147783408    ......PMIDR......-----.....-----.....IIHRD......GDFGL......LVDIY......
147815157    ......HLLKT......IMKFY.....NFVTE.....VIHRD......GDLGL......LVDIY......
148612809    ......EMLKG......IVRFY.....VLVTE.....IIHRD......GDLGL......SVDVY......
148667212    ......EMLKG......IVRFY.....VLVTE.....IIHRD......GDLGL......SVDVY......
148667213    ......EMLKG......IVRFY.....VLVTE.....IIHRD......GDLGL......SVDVY......
148709114    ......EMLKG......IVRFY.....VLVTE.....IIHRD......GDLGL......SVDVY......
149031320    ......EMLKG......IVRFY.....VLVTE.....IIHRD......GDLGL......SVDVY......
149045044    ......EMLKG......IVRFY.....VLVTE.....IIHRD......GDLGL......SVDVY......
149049592    ......EMLKG......IVRFY.....VLVTE.....IIHRD......GDLGL......SVDVY......
149049591    ......EMLKG......IVRFY.....VLVTE.....IIHRD......GDLGL......SVDVY......
149272327    ......EMLKG......IVRFY.....VLVTE.....IIHRD......GDLGL......SVDVY......
149412695    ......EMLKG......IVRFY.....VLVTE.....IIHRD......GDLGL......SVDVY......
149758016    ......EMLKG......IVRFY.....VLVTE.....IIHRD......GDLGL......SVDVY......
149758014    ......EMLKG......IVRFY.....VLVTE.....IIHRD......GDLGL......SVDVY......
150175959    ......EMLKG......IVRFY.....VLVTE.....IIHRD......GDLGL......SVDVY......
110749546    ......EIKNR......MMDVS.....VLVSE.....---ED......IDFGC......QTDLY......
169599821    ......QAKRR......IVSAY.....YLVEE.....VIHGD......IDFGR......QIDYH......
118403495    ......QIRER......FIGFQ.....VLVGD.....IIHGD......IDLGQ......QTDYF......
115398872    ......QRLSR......IIRAH.....VLVED.....ILHGD......IDFGR......QIDLY......
114579502    ......QLMER......FMKFY.....VLVGE.....IIHGD......IDLGQ......QIDYF......
115653055    ......ELHAR......IMTAE.....CMVTE.....IIHGD......IDFGR......QVDYY......
116060678    ......RLLER......ALHLL.....VLVMP.....IVHAD......IDYGR......QADCY......
169859586    ......ALPAN......VLP-H.....FLILD.....FIHGD......IDFGR......QTDYF......
121699339    ......TAHER......IIRAH.....ILVED.....ILHGD......IDFGR......QIDLY......
119469256    ......IAHER......IIRAH.....ILVED.....VLHGD......IDFGR......QIDLY......
119902314    ......KLKER......D-NLC.....IVWHQ.....IVHGD......VDFSY......QVDLF......
119902317    ......KLKER......D-NLC.....IVWHQ.....IVHGD......VDFSY......QVDLF......
163937845    ......QLMER......FIKFY.....ILVGE.....IIHGD......IDLGQ......QIDYF......
125558471    ......QLDTR......FGYTH.....VLVCD.....IIHGD......VDWGR......QADTY......
125600377    ......QLDTR......FGYTH.....VLVCD.....IIHGD......VDWGR......QADTY......
125845805    ......QLNKR......YNSIH.....VLVGQ.....IIHAD......IDLGQ......QTDYF......
126278361    ......KLKER......FSDDC.....IILHQ.....IVHGD......VDFSY......QVDLI......
```

```
126304245   ......QLKER......FIQFD......VLVGD......IIHGD......IDLGQ......QTDYF......
145243342   ......TANER......IIRAH......ILVED......ILHGD......IDFGR......QIDLY......
145616442   ......LAHDR......VASLS......FLMLP......LLHGD......IDFGR......QIDCH......
146421479   ......-IQAR......VVAPR......YMVMD......IIHGD......IDFGR......EADYY......
149237436   ......LLKSP......FIKCE......FLILD......IIHGD......IDFGR......EADYY......
148696263   ......QLMER......FIKFY......ILVGE......IIHGD......IDLGQ......QIDYF......
148696262   ......QLMER......FIKFY......ILVGE......IIHGD......IDLGQ......QIDYF......
150865296   ......LLRDR......IIHPE......YLIMD......ILHGD......IDFGR......EADYY......
149692488   ......QLQER......D-NLC......IVWHQ......IVHGD......VDFSY......QVDLF......
149727365   ......QLMER......FIKFY......VLVGD......IVHGD......IDLGQ......QIDYF......
154311154   ......QAKRR......IIDVY......YLIEE......ILHGD......IDFGR......QIDYH......
156120669   ......QLMER......FIKFY......VLVGD......IIHGD......IDLGQ......QIDYF......
157120285   ......NVL-N......IVRVL......LVIME......IVHLD......CDFGS......MADIY......
111955036   ......NI-AR......IVRVV......TIIME......ILHLD......SDFGC......KADIY......
114620198   ......NV-AR......IVRVV......TIIME......IVHLD......SDFGC......KADIY......
85542929    ......NV-AH......VVHVI......TIIME......IVHLD......ADFGC......KADIY......
85542983    ......NV-AH......VVHVI......TIIME......IVHLD......GDFGC......KADIY......
85542933    ......NV-AH......VVHVI......TIIME......IVHLD......GDFGC......KADIY......
85542987    ......NV-AH......VVHVI......TIIME......IVHLD......GDFGC......KADIY......
85542963    ......NV-AH......VVHVI......TIIME......IVHLD......GDFGC......KADIY......
85542991    ......NV-AH......VVHVI......TIIME......IVHLD......GDFGC......KADIY......
85542947    ......NV-AH......VVHVI......TIIME......IVHLD......GDFGC......KADIY......
85542973    ......NV-AH......VVHVI......TIIME......IVHLD......GDFGC......KADIY......
85542997    ......NV-AH......VVHVI......TIIME......IVHLD......GDFGC......KADIY......
85542949    ......NV-AH......VVHVI......TIIME......IVHLD......GDFGC......KADIY......
85542937    ......NV-AH......VVHVI......TIIME......IVHLD......GDFGC......KADIY......
85542975    ......NV-AH......VVHVI......TIIME......IVHLD......GDFGC......KADIY......
85542943    ......NV-AH......VVHVI......TIIME......IVHLD......GDFGC......KADIY......
85542931    ......NV-AH......VVHVI......TIIME......IVHLD......GDFGC......KADIY......
85542953    ......NV-AH......VVHVI......TIIME......IVHLD......GDFGC......KADIY......
85542961    ......NV-AH......VVHVI......TIIME......IVHLD......GDFGC......KADIY......
85542939    ......NV-AH......VVHII......TIIME......IVHLD......GDFGC......KADIY......
85542989    ......NV-AH......VVHVI......TIIME......IVHLD......GDFGC......KADIY......
85542993    ......NV-AH......VVHVI......TIIME......IVHLD......GDFGC......KADIY......
85542977    ......NV-AH......VVHVI......TIIME......IVHLD......GDFGC......KADIY......
85542999    ......NV-AH......VVHII......TIIME......IVHLD......GDFGC......KADIY......
85542927    ......NV-AH......VVHVI......TIIME......IVHLD......GDFGC......KADIY......
85542995    ......NV-AH......VVHVI......TIIME......IVHLD......GDFGC......KADIY......
85542925    ......NV-AH......VVHVI......TIIME......IVHLD......GDFGC......KADIY......
85542965    ......NV-AH......VVHVI......TIIME......IVHLD......GDFGC......KADIY......
85542957    ......NV-AH......VVHVI......TIIME......IVHLD......GDFGC......KADIY......
85542945    ......NV-AH......VVHVI......TIIME......IVHLD......GDFGC......KADIY......
85542951    ......NV-AH......VVHVI......TIIME......IVHLD......GDFGC......KADIY......
85542959    ......NV-AH......VVHVI......TIIME......IVHLD......GDFGC......KADIY......
85542923    ......NV-AH......VVHVI......TIIME......IVHLD......GDFGC......KADIY......
85542967    ......NV-AH......VVHVI......TIIME......IVHLD......GDFGC......QADIY......
85542981    ......NV-AH......VVHVI......TIIME......IVHLD......GDFGC......KADIY......
85542985    ......NV-AH......VVRVI......TIIME......IVHLD......GDFGC......KADIY......
85542971    ......NV-AH......VVHVI......TIIME......IVHLD......GDFGC......KADIY......
85542935    ......NV-AH......VVHVI......TIIME......IVHLD......GDFGC......KADIY......
85542969    ......NV-AH......VVHVI......TIIME......IVHLD......GDFGC......KADIY......
85542941    ......NV-AH......VVHVI......TIIME......IVHLD......GDFGC......KADIY......
85542955    ......NV-AH......VVHVI......TIIME......IVHLD......GDFGC......KADIY......
120564949   ......NV-AH......VVHII......TIIME......IVHLD......GDFGC......KADIY......
126321360   ......NV-AR......VVRVL......TIIME......IVHLD......GDFGC......KADIY......
148673766   ......NI-AR......IVRVV......TIIME......ILHLD......SDFGC......KADIY......
109092648   ......WALTS......VVQFE......WFVME......IVHRD......ADFGL......KADIF......
114680533   ......WALTS......VVQFE......WFVME......IVHRD......ADFGL......KADIF......
115432021   ......WAITA......VIALY......WLVME......IVHRD......ADFGL......QADIF......
117606299   ......WALAS......VVQLE......WFVME......IVHRD......ADFGL......KADIF......
119631008   ......WALTS......VVQFE......WFVME......IVHRD......ADFGL......KADIF......
166157498   ......WALTS......VVQFE......WFVME......IVHRD......ADFGL......KADIF......
123250196   ......WALSS......VIHLE......WFVMD......IIHRD......ADFGL......KADIF......
125852538   ......WALAS......VVQLE......WFVME......IVHRD......ADFGL......KADIF......
126304203   ......WALQS......VIWLK......WFVME......IVHRD......TDFGL......KADIF......
126328651   ......WALSS......VIHLE......WFVMD......IIHRD......ADFGL......KADIF......
148696300   ......WALTS......IVQFE......WFVME......IVHRD......ADFGL......KADIF......
148698073   ......WALSS......VIHLE......WFVMD......IIHRD......ADFGL......KADIF......
148698072   ......WALSS......VIHLE......WFVMD......IIHRD......ADFGL......KADIF......
148725937   ......WALSS......VIHLE......WFVMD......IIHRD......ADFGL......KADIF......
149497173   ......WALSS......VIHLE......WFVMD......IIHRD......ADFGL......KADIF......
108799873   ......RSAAM......IVAVH......FIVME......VLHRD......ADFGI......ADDLY......
109017881   ......AALEQ......IVTLY......CLLLE......YVHAD......IDFGL......AVDLW......
```

```
109017879   ......AALEQ.....IVTLY.....CLLLE.....YVHAD.....IDFGL.....AVDLW.....
109086291   ......QMLKS.....VVTLL.....-ILTE.....MGTRD.....NDLDA.....KIDIW.....
113476852   ......AYLQR.....IAKVY.....YMVME.....LLHRD.....IDFGT.....ASDIY.....
110006315   ......NNVMR.....IVRLV.....VICFE.....IYHGD.....GDFGL.....KSDVF.....
121593345   ......RALAQ.....VVSVL.....YMVMN.....MLHLD.....IDFGA.....PTDIY.....
111020684   ......RTTAQ.....IAGIY.....YLVME.....VVHRD.....TDFGI.....ASDVY.....
111020581   ......RFLAE.....IVKIY.....YIVME.....LVYND.....IDMGA.....ASDIY.....
111022800   ......RAMGK.....VVDIL.....YIVMP.....TLHRD.....TDFGI.....ASDVY.....
111022297   ......RAMGR.....IVNVL.....FIVMP.....IVHRD.....TDFGI.....ASDIY.....
111019188   ......QFLAE.....IVKIF.....YIVME.....LVYND.....IDLG-.....ASDIY.....
111019396   ......RAMGR.....IVNIM.....YIVMQ.....ILHRD.....TDFGI.....ASDVY.....
111017305   ......YAMGG.....IVNIL.....YIVMH.....TLHRD.....TDFGI.....RSDVY.....
111026298   ......RAAGR.....VVNVL.....YIVMP.....ILHRD.....TDFGI.....AADVY.....
111025675   ......QAMGR.....IVVLQ.....YIVMP.....VLHRD.....TDFGI.....VTDVY.....
111024860   ......RAMGQ.....IVNVL.....FLVMQ.....ILHRD.....SDFGI.....ASDVY.....
121611074   ......RALAQ.....VVSVL.....YMVMN.....MLHLD.....IDFGA.....PTDIY.....
110833945   ......WVGNR.....LLHAL.....YQHLE.....VIHGD.....VDFGH.....QSDLF.....
169618894   ......ANLQT.....ILPLY.....SFLFP.....SCHRD.....ADFGL.....ASDIW.....
169618896   ......RVLQI.....IISLE.....VLVFE.....IFHGD.....TDFSF.....GADMY.....
169600253   ......AIIRG.....IISVH.....GILLE.....IRHKD.....TDFGY.....KSDVY.....
111225843   ......AAVAA.....VARLL.....WIATE.....VVHRD.....IDFGI.....ACDIF.....
111223971   ......RNAAR.....VVTVH.....WIVMD.....VLHRD.....TDFGI.....AGDLF.....
111223728   ......RILTT.....IVKIL.....YIVME.....LVYCD.....IDLGG.....TTDTF.....
111223745   ......RMVAK.....VVTVL.....WIIME.....ITHRD.....VDFGV.....ASDLF.....
111224906   ......DNARR.....TAAVL.....YLITE.....IVHRD.....IDFGI.....AADVF.....
111220231   ......RHAAK.....VVTIL.....WIVME.....IVHRD.....TDFGI.....EADLF.....
111223302   ......RFLAA.....IVKIY.....YIVME.....LVFCD.....IDLGA.....ASDLY.....
111226078   ......EAARR.....TAEVL.....YLVTE.....LVHRD.....IDFGI.....EADVF.....
111223426   ......TILAC.....IARVR.....FLIMD.....LVHQD.....LDFGL.....RSDLY.....
111223813   ......AAARR.....VAAVL.....WLATE.....VVHRD.....IDFGI.....PADIF.....
111222068   ......KHLNK.....IVRAT.....ALIAE.....VLHRD.....IDFGV.....AADLY.....
111225750   ......DHLRR.....LAAIL.....YVVVE.....IVFGD.....VDFGL.....ASDIF.....
111223258   ......AVARR.....TAEVL.....YLVTE.....LVHRD.....IDFGL.....AADIF.....
111221261   ......QACFR.....TAQLL.....WLATE.....LIHRD.....-DFGV.....ASDVF.....
111220703   ......DIARR.....TAEVL.....YLVTE.....LVHRD.....IDFGI.....AADVF.....
111224466   ......KAVAR.....VVSVL.....YLVME.....IIHRD.....ADFGL.....RSDVY.....
111220066   ......DAARR.....TAPVL.....WLVTA.....LVHRD.....IDFGI.....ASDVF.....
111225532   ......ATHRT.....VVRFL.....WVALE.....IVHRD.....IDFGL.....ACDVF.....
111224332   ......ESARR.....TAAVL.....YLVTE.....IVHRD.....IDFGL.....AVDIF.....
111226087   ......RHAAR.....VASVY.....YLVME.....VVHRD.....TDFGI.....ASDVY.....
111223424   ......DNARR.....TPSVL.....YLVTE.....IVHRD.....IDLGI.....ATDVF.....
120610153   ......RLLAK.....LVHVY.....YMVMQ.....CYHRD.....LDFGA.....QTDIY.....
120612228   ......RALAQ.....VVSVL.....YMVMN.....MLHLD.....IDFGA.....PTDIY.....
115460772   ......VLIAK.....LVRLI.....LLIYE.....IIHRD.....SDFGM.....KSDTY.....
113866975   ......RSLAR.....VVRVV.....YMVMN.....LLHLD.....LDFGA.....WTDVY.....
115436090   ......EATMQ.....IVKVF.....FIVYK.....IVHRD.....SDFGI.....KCDVY.....
115455631   ......GFLAK.....IVPLL.....LLVYK.....LVHRD.....GSLDV.....SYDVY.....
115470615   ......TTLGN.....IVKLY.....LILYE.....VIHRD.....GDFGL.....KCDIY.....
159897016   ......QPSTL.....RIAIA.....GFIMP.....LIHQD.....TDIDS.....EWDNF.....
145592613   ......RTMAT.....VVDIY.....FLVME.....IVHRD.....TDFGI.....SSDVY.....
159900833   ......ELLTR.....IPQVF.....TIVME.....LVHGD.....VDFGA.....ASDIY.....
159901044   ......HTLAS.....LAPVL.....MLVMG.....LIFRD.....IDFGI.....RSDLY.....
159901503   ......QVAVQ.....IVRVF.....FIVME.....ILHRD.....VDLGL.....RTDIY.....
159897975   ......SALAL.....LPEVI.....YIIME.....ITHRD.....IDFGF.....RSDLY.....
159897246   ......SLLAR.....LPRVI.....FLVME.....IIYRD.....IDFGI.....RSDIY.....
159901650   ......YQCQI.....IKALY.....VFTLD.....IVHGD.....IDFDN.....LSDIF.....
159899552   ......RLLKK.....VIGFV.....TLATT.....VIHRD.....IDLSV.....ATDQY.....
159899479   ......DAAAQ.....IVRVY.....YIVME.....LVHRD.....SDFGI.....LSDVY.....
160873775   ......CCLTT.....LIKVV.....GLVME.....VSHGD.....GDFGA.....--EVR.....
114053449   ......KTLWR.....LVRLL.....VLIYE.....IIHRD.....GDFGL.....KSDVY.....
115386652   ......AKFSQ.....CVKSF.....FISME.....FAHRD.....GDFGI.....AVDVW.....
115386068   ......TIYRI.....ILNCL.....CIRFP.....IVHAD.....CNFSG.....VTDIF.....
115383850   ......NALHA.....VVQVE.....GLLIT.....YVQGD.....ID---.....KTDLY.....
115384834   ......QTLTR.....VVPFH.....SLVMS.....VVHGD.....ADFSS.....ASDVF.....
114765051   ......Q-MRS.....VVRYS.....FLVMD.....IVHRD.....IDFGI.....RADLY.....
114561007   ......AALEQ.....IVTLY.....CLLLE.....YVHAD.....IDFGL.....AVDLW.....
114561009   ......AALEQ.....IVTLY.....CLLLE.....YVHAD.....IDFGL.....AVDLW.....
114568299   ......SCLQS.....LVTFY.....FVCVT.....YTHQD.....ADFDK.....KRDLE.....
114568301   ......SCLQS.....LVTFY.....FVCVT.....YTHQD.....ADFDK.....KRDLE.....
114619992   ......QMLKS.....VVTLL.....-MLTE.....VGTRD.....NDLD-.....KIDIW.....
116249127   ......EIGMA.....IAETQ.....ALVME.....FVHCD.....ADFGI.....RSDLY.....
```

115495227	......SVLEK......IVTLY......CLLLE......FVHAD......IDFGL......AVDLW......
115379558	......KVSVA......VVQVL......YMVME......IVHRD......ADFGI......RSDIF......
115379328	......RAIAR......VVSVF......YIVSE......VLHRD......LDFGL......RSDLY......
115380749	......TTLQR......IVEIL......FIAME......VVHRD......VDFGV......RTDLY......
115379748	......ACLLI......VVRVW......YVVMD......IHHRD......VDFGA......SDDLY......
115379837	......GILQR......VAKPY......VLLLE......VIHKD......IDFGV......RTDFY......
115379329	......ELLSR......VPKLV......YVVME......GLHRD......MDFGC......ARDVY......
115377974	......ACLQQ......IIRML......YIVLD......VLHRD......IDFGA......KEDIF......
115380274	......RAIAQ......VVQIY......YIVME......IVHRD......LDFGL......RSDLW......
115380398	......KAVNL......IVNIF......YLVME......VVHRD......LDFGI......RTDLY......
115379341	......ELLTR......VPRLL......YLVMQ......GLHRD......MDFGC......ADDVY......
115379545	......RAIAR......VVTAF......YLVSE......VLHRD......LDFGL......QSDLY......
115379612	......ELLSR......VPRLL......YLVMQ......GLHRD......MDFGC......ADDVY......
115379157	......HLATY......IARTL......YIVSD......IVHRD......TDFAR......RSDLF......
115378095	......DLMGV......LVRKL......FIAIE......LVHGD......GDFGV......ATDLF......
115376146	......VLLSR......VPSLH......FLVMD......GVHRD......IDFGA......ADDVY......
115378682	......ELLSR......VPRLR......YLVMD......CLHRD......VDFGC......YEDVY......
115375989	......ELLSR......VPRLR......YLVMD......GVHRD......MDFGS......SDDVY......
115377324	......RVLVK......IAQVL......YMALE......LVHRD......IDFGL......RSDLY......
115377990	......ELLTR......VPRLL......YVVMQ......GLHRD......MDFGC......ADDVY......
115375287	......VLLSR......VPRLR......YLVME......GVHRD......MDFGS......QADVY......
115376140	......VLLSR......VPSLH......FLVMD......GVHRD......IDFGA......ADDVY......
115377289	......GLLAQ......VPCLL......YVVMQ......GLHRD......MDFGC......ADDVY......
115379017	......ELVAS......VLEIH......FLVCE......VVHRD......ADFGI......ASDVY......
115376167	......TTLLM......IIRPQ......YVALE......VLHRD......IDFGC......ADEIF......
115378310	......TTLLM......IIRHR......YVALE......VLHRD......IDFGC......ADEIF......
115375385	......ELLTR......VPRLL......FLVMQ......GLHRD......VDFGC......ADDVY......
115377281	......GLLAQ......VPCLL......YVVMQ......GLHRD......MDFGC......ADDVY......
115375013	......ELLSR......IPRLS......FLVMD......CLHRD......TDFGS......YADLY......
115376249	......ELLSR......VPRLL......YLVMD......AVHRD......TDFGA......QDDVF......
115378342	......LVTAR......IVPVY......FFAMK......IIHRD......IDWGL......RADVY......
115377760	......DLLQR......ITRAH......GLLLE......VIHKD......IDFGI......RTDLY......
115372861	......ELLSR......VPALV......FLVMQ......GVHRD......MDFGS......YEDIY......
115374087	......RAIAR......VVTVH......YLVTE......VLHRD......LDFGL......RSDLY......
115374755	......RLAAE......IVQIF......FLAME......VVHRD......VDFGI......RTDVY......
115374797	......ELLTR......VPRLL......YVVMQ......GLHRD......MDFGC......ADDVY......
115372767	......ALLSR......VPSLL......YLVMD......GVHRD......LDFGS......SDDVF......
115373579	......ELLSR......IPRLL......YIVME......AIHRD......MDFGS......KDDLY......
115372584	......RIAAL......IAQIY......FIAME......LIHRD......IDFGV......RSDVF......
115371859	......ELLSR......IPRLS......FFVMD......CLHRD......TDFGS......YADLY......
115372830	......EIGRK......AVVVL......AIVME......VIHKD......IDFGL......RSDLY......
115373480	......ELLSR......VPRLL......YLAME......GVHRD......MDFGS......GEDVY......
115374280	......TTLLM......IIRHR......YVALE......VLHRD......IDFGC......ADEIF......
115374777	......GILQR......VARPY......VLLLE......VIHKD......IDFGV......RTDFY......
115373588	......RLVGR......LVTIL......CLAME......LVHRD......IDFGV......RSDQF......
115374883	......TTLLM......IIRPQ......YVALE......VLHRD......IDFGC......ADEIF......
115373908	......ELLSR......VPRLY......YVVMN......GVHRD......MDFGS......TDDLY......
116049730	......WFLRR......FPELH......YYLMR......LLHRD......LDFGL......RQDLY......
115665255	......EALVN......FPKTV......SLVLE......LLHND......IDMGH......QTDIY......
115670794	......VHLTL......FPKAI......SLVLE......LLHND......IDMGK......QTDIF......
115715494	......AVHRL......FPRCH......GLVVE......LLHND......IDMGN......QSDIF......
115724273	......AIHRR......IVQMM......FLVMQ......ILHLD......CDLGL......KHDIW......
116057718	......KLLTR......IVKAH......YMVTE......FAHRD......CDFGS......KSDVY......
116061326	......ETVHR......CARFY......YLMWE......IVHRD......DLGGS......KFDLF......
116074021	......ACLDR......IPRVL......CFIQE......LVHGD......VDFSL......AADLY......
116074408	......PSMQA......IPRFG......WLVRE......LVHGD......LDFGL......WMDLH......
116513453	......VNIKM......IPNYI......FLCEE......IFLGD......VDLEQ......AQDNQ......
116334440	......NILHN......KPRVL......FMLES......VVIRD......VDFEI......SRDWY......
116283303	......SCLRD......LVAFY......YVCVS......YSHQD......ADFD-......RRDLE......
116310797	......ALLKK......LVQLL......IVITE......IIHRD......ADFGF......KSDVF......
169857929	......RVWKQ......VLPLY......SMVCP......IIHGD......CDFGL......KSDVY......
169863095	......STVSH......VARFH......-IVVE......ITHGD......IDFGL......SSDVF......
169851235	......IIWSQ......VLPFY......FLLSP......VIHGD......GDFGL......ASDVY......
117574634	......FCYKN......VVTLI......FLLMM......IVHTD......GDFG-......ATDAW......
117616942	......HYMRS......LVDLH......LLIME......LVHRD......TDLGF......SVDVY......
117616944	......HYMRS......LVDLH......LLIME......LVHRD......TDLGF......SVDVY......
117617014	......QMLKS......VVTLV......-ILTE......LGTRD......NDLD-......KVDIW......
117621930	......NNVMR......IVRLV......VICFE......IYHGD......GDFGL......KSDVF......
117621924	......NNVMR......IVRLV......VICFE......IYHGD......GDFGL......KSDVF......
118047989	......RTAAN......IVTVY......YIAME......AVHRD......TDFGI......RSDLY......
118048078	......RIAAA......ILRIN......YIISE......VVHGD......SGFAL......AADVF......

```
118046089    ......QILAR......LPRVI......FLVME......IIHRD......LDFGL......RSDIY......
118049150    ......RSLAQ......VVSVL......YMVMN......MLHLD......IDFGA......WTDIY......
156743428    ......DILAK......LLPLR......YMVCA......LFLGD......TPFTL......VSDVY......
156740595    ......RIIAN......IVQVY......YMVQE......VIHRD......TDFGI......ASDIY......
156742260    ......QLLAG......LPKVS......FLVME......IIHRD......LDFGL......RSDLY......
156742983    ......QAAAG......IVNVY......YIVME......MVHRD......TDFGI......RSDIY......
156741462    ......VTVAN......IVPVY......YIAME......AVHRD......TDFGI......RSDLY......
156742137    ......GLLAT......IPKVF......YLILE......IVFRD......IDFGI......QSDLY......
156740357    ......QLLAS......LPRVI......FLVME......VIHRD......LDFGL......RSDLY......
157371254    ......RLLAR......LLHVL......YMGTQ......YLHRD......LDFGS......QTDIY......
170726211    ......RALAK......IVQIY......VLVME......VVHCD......VDFGI......LSDLF......
170728480    ......FMLSQ......VPPAI......ILVMA......LVHGD......IDWGS......RFDEQ......
118463342    ......QFLAE......IVQIF......YIVME......LVYND......IDLGA......ATDIY......
118467837    ......HAAAG......VVPIH......YLDMR......LIHRD......IDFGL......RSDIY......
118472918    ......RTVAM......IASVY......YLVME......LVHRD......TDFGI......ASDVY......
118468912    ......QFLAE......IVKIY......YIVME......LAYND......IDLGA......ATDIY......
118479084    ......QSVTT......IVNMY......YLVME......IVHRD......TDFGI......QSDIY......
118477622    ......SSLVG......IVTKE......SIVMP......VAHND......VDVEY......EADRF......
118588933    ......KILRK......LTQYY......YLAME......VIHRD......IDFGI......RSDIY......
118619812    ......RAMAK......IVSVL......YLVMP......ILHRD......TDFGL......ASDVY......
118619813    ......RAMAR......IVSVL......YLVMP......IVHRD......TDFGI......ASDVY......
118618692    ......RNAGR......VVPIH......YLDMR......VMHRD......VDFGI......RADIY......
118573868    ......QMLKS......VVTLL......-ILTE......MGTRD......NDLDA......KIDIW......
118430798    ......FCYKN......VVTLI......FLLMM......IVHTD......GDFG-......ATDAW......
170735554    ......DVIRR......VPEII......YEVAE......LRHRD......SGFGS......ASDWW......
160897473    ......RSLAQ......VVSVL......YMVMN......MLHLD......IDFGA......WTDIY......
160899272    ......RLLAR......LVHVY......YMVMR......CFHRD......LDFGA......QTDIY......
152997646    ......QLLAS......IAKVF......YISME......IIHGD......IDFNL......SIDIY......
146303616    ......AKMQE......MVRIL......AIVME......YVHAD......SDFGS......SMDVY......
157960415    ......FMLSQ......VPPVI......ILVMA......LIHGD......IDWGS......RFDEQ......
146304560    ......SKLQE......MVTLY......TMVME......YVHLD......GDLGA......RMDIY......
157962756    ......-CLQA......LIKVV......GLVME......ISHGD......GDFGA......AIEVR......
146303644    ......DLLEL......LVKLV......MIVME......YVHCD......ADLGS......RDDVY......
119026630    ......MALQK......VCGIV......FIVTE......IVHRD......VDFGI......MTDWW......
119385168    ......EAMRD......VVRYS......FLVMD......VVHRD......IDFGI......ASDLY......
121705254    ......LVLTR......IVPFH......SIVMS......IVHGD......ADFSS......ASDVF......
121716138    ......EKTAG......LTNLL......HLVFP......SVHGD......TDLGL......KWDNW......
121709688    ......QNLGL......ISLPL......CFV--......ICHGD......IDFGE......GCDLW......
121712796    ......EALKK......LVDFI......YLLLE......FVHGD......ASIEE......KTDVW......
121715704    ......NALHA......VIQVE......GLLIS......YVQGD......ID---......KTDLY......
119481219    ......NALHA......VIQVE......GLLIS......YVQGD......ID---......KTDLY......
119489928    ......SIYHR......LVRIL......GLILE......IIHCD......IDFSG......ATDLF......
119492260    ......LALTR......IVPFH......AIVMG......MVHGD......ADFSS......ASDVF......
119470225    ......YMLSQ......VPKFY......ILMMA......IVHGD......VDWGS......NADVY......
119492428    ......EILEK......IPLLL......YLVEE......VIHRD......IDFGS......NSDLY......
119493596    ......KVLSK......TVDKD......CLVME......YFHRD......IDFGT......QSDFF......
119490480    ......SVLKS......IPQYL......AFVQT......VIHRD......IDLGS......ASDLY......
119488959    ......KVLCQ......IVEQD......CLVME......YYFRD......TDYGL......D-DFF......
119487600    ......VRLDQ......IPDLL......YLVQE......VIHRD......VDFGA......ASDLY......
119489016    ......VRLEQ......IPQLY......FLVEE......IVHRD......IDFGV......ASDLY......
119483263    ......QILQQ......LVRYR......CLVTE......VLHRD......IDFGC......ASDLY......
119487083    ......VRLDE......IPELY......YLVQE......VIHRD......VDFGA......SSDIY......
119487581    ......RQLQQ......IPTLS......YLLQE......VIHRD......LDFGV......ASDLF......
119484724    ......KQLKY......IPQWL......YLLQE......IIHRD......VDFGA......ASDIY......
119489320    ......SVLAR......VPKVS......CLVME......IIHRD......IDFGG......PADFY......
119490072    ......QTLEK......IPQLL......YLVES......VIHRD......IDFGA......NSDIY......
119486583    ......TTLGK......IPRLL......YLVQE......VIHRD......IDFGA......ASDIY......
119489842    ......QLLHR......IPRLY......YFVQE......VIHRD......IDFGA......ASDIY......
119487723    ......VITKN......VVKSL......ALVME......VIHKD......IDFSI......RTDFY......
119505093    ......AAMTS......VPQIY......WLLLE......LVHGD......IDFSS......ADDQY......
119502888    ......RKSQQ......IITVY......YMTME......IIHSD......LDFGI......GDDIY......
119512967    ......QVLYK......IPQLL......YLVQE......VIHRD......IDFGA......SSDIY......
119512796    ......ETLKS......IPRYL......-LVQS......VIHRD......VDFGS......ASDLY......
119512795    ......ETLKS......IPRYL......-LVQS......VIHRD......VDFGS......ASDLY......
119508927    ......TITKD......IVKPL......VLIME......IIHKD......IDFSI......RSDFY......
119509293    ......QVLQN......IPRYR......CLVQD......VLHRD......VDFGA......ASDLY......
119511961    ......ERLEE......IPELM......YLVQE......VIHRD......VDFGA......ISDLY......
119512046    ......NVLKT......IPRFH......FLVQD......VVHRD......IDFGG......NSDLY......
```

```
119512035     ......VQLDD......IPALL......YLVQE......VIHRD......VDFGA......ASDIY......
119511566     ......AILED......IPTLY......YLVQE......IIHRD......IDFGA......SSDLY......
119511728     ......DILEV......IPELL......YLIQE......IIHRD......IDFGA......SSDIY......
119512547     ......EVLSI......IPKVD......CLVME......YFHRD......IDFGT......QSDFF......
119511368     ......FRLYE......IPRLL......YLVQE......VIHRD......IDFGG......GSDLY......
119512522     ......EVLAQ......IPDLF......YLVQE......IIHRD......LDFGA......STDLY......
119511124     ......HTLKS......IPKYY......YLAME......LIHRD......LDFGA......QSDLY......
119509071     ......ERLEE......IPELM......YLVQE......VIHRD......VDFGA......ISDLY......
119511565     ......AILED......IPTLY......YLVQE......IIHRD......IDFGA......SSDLY......
119509196     ......EVLIK......VVESD......CLVME......IIHRD......IDFGT......AADFY......
119717927     ......QLAGT......VVQVF......YVVME......IVHRD......LDFGV......ASDVF......
119900177     ......RLLAQ......LVKVY......YMAMP......CFHRD......LDFGA......QTDVY......
119900250     ......RSLAK......VVRVL......YMVMQ......LLHLD......LDFGA......WSDIY......
159035726     ......RTMAT......VVDIY......FLVME......IVHRD......TDFGI......VSDVY......
119875215     ......RILQK......LVPFV......FLVQE......VVLRD......IDLEF......QEDHH......
159039020     ......KTIAR......VVAVY......YLVME......LVHRD......ADFGL......RTDVY......
119611115     ......AALEQ......IVTLY......CLLLE......YVHAD......IDFGL......AVDLW......
119962857     ......QAVAA......IVAIF......YIVME......IVHRD......MDFGI......RSDLY......
119960526     ......RLLSD......LIRVH......GIVMD......TEHGD......ADLGI......SRDVY......
119963247     ......RHTAL......IANVF......YLVME......LVHRD......TDFGI......SSDIY......
120406349     ......RSAAA......IVAVH......FIVME......VVHRD......ADFGI......ADDLY......
121528290     ......RSLAK......IVRVL......YMVMT......LLHLD......LDFGA......WTDIY......
153003964     ......AVTRR......IVEVL......FLVME......IVHCD......GDFGV......RTDVF......
153006613     ......IAASR......VVDVV......YYVME......VVHRD......LDFGI......RADVY......
153005777     ......RIAVR......VVDVL......YLVME......IVHRD......LDFGI......RADLY......
153006193     ......AVLAA......VPRLV......YLVME......VIHLD......VDFGL......RSDVF......
158520662     ......ELVRS......VVRVL......ALVVE......IVHRD......TDFGI......HTDLY......
123329031     ......----P......LAVPN......-----......IILRD......ADFDV......KSDIF......
123377540     ......----A......LLKFT......YLLME......ISISD......NFLSL......MSDIW......
123384663     ......DCMKQ......IIRLY......YLLME......IAHLD......CDFGM......KADIW......
123404998     ......DALCA......IVRLY......YLTME......ISHGD......GDFGL......KADVW......
123406065     ......ESMIA......VVTLY......YMVME......FTHGD......CDFGL......AADIW......
123432995     ......RCLEA......IVSLY......YLMME......IAHCD......NDFSK......KADIW......
123431601     ......ESMIT......IVTLY......YLMME......ISHGD......CDFGL......KADIL......
123447741     ......QYIEK......EIELY......YILQD......MSQND......SSMTI......KSDIW......
123439738     ......ETYGK......IVKFF......FIFYN......IVCHD......VAYGL......KSDIF......
123452148     ......DCMIQ......IVTLY......YLLME......ITHGD......CDFGL......AADIW......
123452903     ......QSMQD......VIRLY......YLLME......VAHLD......TDFGL......KADIW......
123477142     ......EVCKT......VNKPY......FIIYE......YVCGD......INYNL......KSDAY......
123477989     ......NTLKA......VLRFY......TIIYS......VIHRD......VDFGL......EVDVW......
123496188     ......SILEL......VTQLL......YIVME......INHFD......ADFGE......ASDVW......
123479787     ......KLLEQ......VLSYK......FILME......ILHRD......CDFGT......FSDIW......
123504260     ......DCLIA......IVSLY......YLLME......IAHSD......GDFGF......TADIW......
123491888     ......QRMKQ......ILPVV......IVIYE......IVHGD......ASVGI......QSDIY......
123487567     ......DKRNL......IVKTI......LLKIE......WIHLD......ADFGT......PTDIF......
123509930     ......TILEL......VTQLV......YIVME......INHFD......ADFGE......ASDVW......
123509434     ......DIVSE......IISLV......NIIMP......IVHRD......TDFYL......KADVW......
123504358     ......KYLAS......IKTIV......PILVD......IVHGD......ADFQI......ESDIY......
154413545     ......GRRKV......TFQTL......WIISE......YAILD......TNYGI......TSNIF......
154422777     ......NAMAC......VCTLY......TLLFK......FVCGD......LSTHN......ESDIY......
148539606     ......HYTRS......IVAVI......LLLME......LVHRD......TDLGF......SVDTY......
89741418      ......HYTRS......LVNLH......LLIME......LLHRD......TDLGF......SVDVY......
124002653     ......RVAQT......VMKVY......ALFLE......VIHRD......IDFGL......RSDLY......
124006290     ......TAQAS......AVILP......GFLMP......YVFAD......IDVDS......EIDHF......
124006293     ......QHRPE......SVIYP......GFIMK......VVFVD......IDLDS......KSDLF......
124265262     ......QMAGR......IVSVR......YIAME......VTHRD......TDFGI......RSDLY......
124265347     ......ALVGR......VVQIF......YVVME......LIHRD......SDFGS......RADVY......
145475681     ......SILKT......IVTSY......CYFME......IAHLD......CDFGE......AFDIF......
145485841     ......KFLST......IVNFL......CLTME......ILHRD......GDFGI......SIDIW......
145483967     ......RQRNA......LLKIL......AIIIQ......VVHGD......AECSM......KSDIY......
145489229     ......EIIEQ......QPYLY......ILFLK......IVHRD......IDYDR......SYDIW......
145492310     ......Y-LQR......ILRWN......FITLD......IMHKD......GGFSQ......KFDIY......
145492178     ......P----......VLGFN......YIAMD......IIHRD......IDLGL......KIDIF......
145499707     ......CILC-......TVSKI......GLYFE......LLHLD......IDFGL......KNDVY......
145507662     ......NDRKK......ILQYY......LFFFE......LFHGD......LYHNA......KTDSF......
145514303     ......PKL--......ILKVY......CILME......YLHTD......IDFGN......KQDIW......
145519844     ......QNNQN......FIRIY......VSVME......LIHKD......INFGS......AADVW......
145522293     ......KQRNS......LLKIL......AIIIQ......LFHGD......SECSM......KSDIY......
145532829     ......QMQSR......LIEML......CTQME......KSHGD......TDVQQ......KSDVF......
145533991     ......QMGNK......LIQMM......CSQLE......KQHGD......TDI--......KSDVF......
```

145537191	......DIINK.....IIRFF.....IIVME.....LIHCD.....CDMGF.....KVDIW......
145541423	......DKQGS.....IIRIY.....VQVME.....LIHRD.....IDFGL.....SLDIW......
145546975	......QMQSR.....LIEML.....CTQME.....RSHGD.....TDIQS.....KSDVF......
145547040	......NMTSR.....LIQMI.....CTQME.....KSHGD.....TDI--.....KSDVF......
145543925	......RLLER.....CTKLF.....FIFMK.....IIHRD.....IDLDR.....SYDIW......
145550144	......ACMEN.....VATLY.....IIVMK.....VTHYD.....TDFGS.....SSDVW......
125534054	......LNLMS.....IVKLI.....VICFE.....IYHLD.....ADFGL.....KFDVF......
125539862	......DALMS.....IVKLF.....FLVYE.....IVHRD.....SDFGI.....KCDVY......
125536653	......DCLMR.....IVWFV.....VIC--.....IIHLD.....ADFGL.....KSDIY......
125531516	......NALKR.....LVRII.....GLVLE.....IAHCD.....GDFSL.....EGDIY......
125549876	......VLIAK.....LVRLL.....LLIYE.....IIHRD.....SDFGM.....KSDTY......
125549866	......VLIAK.....LVRLI.....LLIYE.....IIHRD.....SDFGM.....KSDTY......
125545867	......GFLAK.....IVPLL.....LLVYK.....LVHRD.....GSLDV.....SYDVY......
125549877	......VLIAK.....LVRLL.....LLIYE.....IIHRD.....SDFGM.....KSDTY......
125557263	......TTLGN.....IVKLY.....LILYE.....VIHRD.....GDFGL.....KCDIY......
125570031	......EATMQ.....IVKVF.....FIVYK.....IVHRD.....SDFGI.....KCDVY......
125591748	......VLIAK.....LVRLI.....LLIYE.....IIHRD.....SDFGM.....KSDTY......
125603026	......VSIGR.....VVTLL.....ALVYE.....VVHLD.....SDFGL.....KSDVY......
125603033	......SSIGR.....VVTLL.....ALIYE.....VVHLD.....SDFGL.....KSDVY......
125523137	......QNIMA.....VVKLV.....YIVAE.....IVHMD.....ADFGL.....KSDIY......
125523160	......QNIMA.....VVKLV.....YIVAE.....IVHMD.....ADFGL.....KSDIY......
125819982	......SVLEK.....IVTLY.....CLLLE.....FVHAD.....IDFGL.....AVDLW......
126193502	......WFLRR.....FPELH.....YYLMR.....LLHRD.....LDFGL.....RQDLY......
126303443	......KMLKA.....VVMLL.....-LLTE.....LVMCD.....NDLD-.....KTDIW......
126306157	......AALEQ.....IVTLY.....CLLLE.....YVHAD.....IDFGL.....AVDLW......
126306498	......ACIQQ.....LMMFH.....YLCFS.....YTHQD.....VDFDK.....KDDLK......
126305270	......AVLEQ.....IMTLY.....CLLLE.....YVHAD.....IDFGL.....AVDLW......
159041697	......TSYLL.....VVKVY.....YIVMD.....MLHLD.....GDLGA.....RADVY......
126348159	......RVAAA.....VTVVH.....FLVME.....IVHRD.....CDFGI.....RSDLY......
126347677	......HAAQR.....TAPVI.....WLATA.....VVHRD.....IDFGI.....ATDVF......
126653955	......CMLQQ.....IVKYR.....YIVTD.....IIHRD.....LDFGS.....YTDIY......
126659312	......ATLKS.....IPQYL.....GLVQN.....VIHRD.....VDFGA.....ASDLY......
126659523	......EVLQS.....IPKYR.....GLVQD.....VIHRD.....IDFGA.....ASDIY......
126661152	......ATLSN.....IPKHL.....CLIQQ.....VFHRD.....VDFGV.....SSDLY......
126658072	......ELLQQ.....IPRYL.....GLVLE.....IIHRD.....IDLGF.....ASDLY......
126657546	......NILKS.....ISQYL.....CLVQK.....IIHRD.....IDFGL.....ATDLY......
126658536	......TITKH.....IVRPL.....AMVME.....IIHKD.....IDFSI.....RSDFY......
126657477	......AILEE.....IPTLY.....YLVQE.....IIHRD.....IDFGI.....SSDLY......
126667139	......YILEQ.....VPRLI.....ILVME.....IVHGD.....IDWGS.....RFDEQ......
126731255	......EGVRE.....VVRYF.....YLVMD.....IVHRD.....IDFGI.....RSDIY......
134095313	......RALAR.....VVSVT.....YMVMG.....LLHLD.....LDFGA.....WTDIY......
134097469	......RVTAR.....VPQVY.....FLVME.....VVHRD.....LDFGI.....HSDLY......
134098455	......QMARS.....TATVV.....WLATE.....LVHRD.....IDFGI.....PSDVF......
134101146	......RILHA.....GVPLI.....YLVTT.....VVVRD.....IDFGN.....EDDYF......
134100560	......RALEA.....VVEF-.....YLAMR.....LVLRD.....IDFEL.....LADRF......
134098662	......RLLAG.....LVPVF.....YLVMQ.....IVHRD.....ADFGI.....AVDVY......
134097872	......LAAAR.....VIPIH.....YLDMR.....LVHRD.....VDFGI.....RADVY......
134103179	......LLLTT.....LVRGY.....AVVTE.....WVHLD.....LDLSL.....AADVW......
145232093	......NALHA.....VVRVE.....VLLIS.....YVQGD.....ID---.....KTDLF......
145234005	......--SAR.....VVSLG.....SIVMS.....MVHGD.....ADFSS.....ESDVF......
154332599	......EAINC.....VIRLV.....-----.....IIHRD.....IDFGL.....ASDVW......
154341272	......ERMRQ.....LIRYL.....CVVMP.....LVHRD.....MDLDL.....KADVF......
154345117	......NIVSS.....IIRIK.....YILLP.....IVHAD.....SDFGI.....MSDMW......
146077953	......EVIYS.....VIRIV.....-----.....IVHRD.....IDFGL.....ASDVW......
146101793	......NIVST.....IIRIK.....YILLP.....IVHGD.....SDFGI.....MSDMW......
146093341	......ERMRQ.....LIRYL.....CVVMP.....LVHRD.....MDLDL.....KADVF......
145241432	......LALSN.....IAKLY.....VLFVE.....IVHGD.....SDFGV.....RFDSY......
134141902	......SCLQS.....LVTFY.....FVCVT.....YTHQD.....ADFDK.....KRDLE......
134141900	......SCLQS.....LVTFY.....FVCVT.....YTHQD.....ADFDK.....KRDLE......
145294097	......QAAEN.....VVATY.....FIVME.....MVHRD.....TDFGI.....ASDIY......
145296750	......--LAD.....IVKAY.....FIVME.....VVYND.....IDLGA.....SSDIF......
145346489	......TLLTR.....IVKVQ.....YMVTE.....FAHRD.....CDFGS.....KSDIY......
145351534	......ACPGA.....VSAFE.....WLVWK.....IVHRD.....IDLGA.....EAPV-......
152999169	......CCLTT.....LIKVV.....GLVME.....VSHGD.....GDFGA.....--EVR......
145602223	......NFLKI.....IVQIT.....MIILK.....LIHLD.....ADFGL.....LTNVW......
145604986	......RIHLK.....IAQLL.....TIIIE.....VAHKD.....LDFGQ.....PGDIW......
145613237	......NALTQ.....VIGLY.....GLLIS.....FVQGD.....ID---.....KSDLY......
145220807	......QFLAE.....IVKIY.....YIVME.....LVYND.....IDLGA.....ATDIY......
146307125	......WFLRR.....FPELH.....YYVQR.....IIHRD.....LDFGL.....QQDLY......
146303114	......SKLQE.....IVKLY.....YIVME.....YVHLD.....GDLGS.....DMDVF......

```
146183341   ......QNILN.....LIV--.....IQIIQ.....IYHSD.....SDFGA.....YHDIY......
146178946   ......NALRE.....INKMI.....YFLLE.....YIYRD.....IDLGY.....EVDVY......
146172282   ......SILYN.....IIQYV.....QIFLE.....IVHGD.....IDFSE.....QIDYY......
146169639   ......MTSKK.....KIYIY.....ISLME.....YAHKD.....ADFGS.....EVDFY......
146163549   ......GIMKD.....VIKFY.....VYEIE.....IVHRD.....ADFDQ.....NSDIF......
146163027   ......NTLQL.....VVKLL.....YIITD.....LPHRD.....TNFRP.....KADIF......
146161687   ......RLMKQ.....VIKVN.....FLAME.....ILHLD.....IDLGL.....QSDIY......
146161685   ......NILKS.....VVEAY.....ITIMD.....ILHLD.....IDMGI.....QSDIY......
95108238    ......DCLRH.....FLTFC.....YVCVA.....YVHQD.....SDFD-.....KNDLE......
148225905   ......SCLQS.....LVTFY.....YVCLA.....YSHQD.....ADFDK.....KRDLE......
147899900   ......SCLQS.....VVTFY.....HVCLA.....YSHQD.....ADFDK.....KRDLE......
95108244    ......SCLPL.....LVTFY.....YVCLA.....YTHQD.....ADFDK.....KTDLQ......
147903123   ......RMLQA.....VVTLL.....-ILTE.....LGTLD.....NDLDA.....KSDIW......
147773362   ......QTLAQ.....LVRFY.....IVVVE.....IIHRD.....ADFGF.....KSDVY......
147767799   ......SLLGR.....LVNLV.....MLIYE.....VIHRD.....ADFGL.....KSDIY......
147767326   ......RALTE.....IVKLL.....FLVYE.....IVHRD.....SDFGT.....KCDVY......
147826902   ......RALTE.....IVKFY.....FLVYK.....IIHKD.....GDV--.....KTDVY......
148271194   ......RHAAL.....IANVF.....FLVME.....LVHRD.....TDFGI.....STDVY......
148359013   ......QGLLQ.....VKPPI.....YLIME.....LIHRD.....IDFGF.....KADVW......
170739241   ......SALHN.....IVRYF.....YLAME.....IIHRD.....IDFGI.....QSDIY......
148707731   ......HYMRS.....LVDLH.....LLIME.....LVHRD.....TDLGF.....SVDVY......
156098629   ......IIMSR.....ILSLL.....YLIYE.....VYHRD.....GDFGL.....QTEIY......
156097528   ......VILRE.....VVKIY.....CFLLQ.....YIYRD.....TDFNL.....TVDIW......
149178052   ......ASVAK.....IVQIF.....FFAME.....LVHRD.....VDFGL.....RSDLY......
149177537   ......RLVYR.....IVSVE.....YAIMQ.....TLHCD.....TGFSL.....RSDLY......
149178274   ......LIAQK.....LITPR.....GHVTP.....YAHGD.....SVIDF.....EADRF......
149177819   ......RAAGR.....IISIH.....YFTMD.....IVHRD.....TDFGL.....RSDIF......
149173225   ......RSAAR.....IVSIY.....ILVTE.....VIHRD.....TDFGL.....RCDIY......
149174255   ......QMLAR.....VVKAL.....YFVQE.....IVHRD.....ADFGL.....SSDIY......
149175801   ......QVVAR.....LVAVQ.....YIMME.....IIHCD.....IDIGS.....QSDLA......
149176103   ......RAVAT.....IVTAH.....FLEME.....IIHQD.....ADFGL.....TSDIY......
149175796   ......KAAAG.....IVQVY.....YIAQE.....IVHRD.....ADFGL.....RSDIY......
149174682   ......QTAAR.....IVTAY.....YLICE.....VIHRD.....LDMGL.....RSDIY......
149174908   ......-----.....LVTIF.....WVVME.....LVHRD.....GDVGL.....EVDVY......
149175021   ......QAAAA.....VIEIH.....YLVMS.....LVHRD.....TDFGL.....ASDLF......
149174278   ......KAAAK.....IVAAY.....YFVME.....VIHRD.....LDMGL.....RSDIY......
153833077   ......DFLSK.....WANFH.....WLMSQ.....YLHGD.....IDFGS.....NDDFY......
153832051   ......SRLEL.....VADFI.....YLMYE.....ICHLD.....IDFGA.....QDDVY......
149058639   ......HYMRS.....LVDLH.....LLIME.....LVHRD.....TDLGF.....SVDVY......
149196744   ......FLTAS.....IIKIL.....FFTME.....VIHLD.....SDWGL.....ATDIY......
149200271   ......RITGR.....IVPVH.....FFTMK.....VVHRD.....MDWGL.....LSDIF......
149196291   ......QILLK.....IVKVH.....FFVQE.....IIHRD.....MDLGM.....RTDIY......
149197415   ......FLTAS.....IIPVY.....YFTMK.....IIHLD.....CDWGI.....QSDIY......
149198452   ......RLTGL.....IISVH.....FFTME.....VLHLD.....CDWGL.....QTDIY......
149196301   ......RFTAQ.....IIPVH.....FYTMK.....IIHRD.....VDWGL.....LTDIF......
149198343   ......RITAM.....IVPLY.....YFTMK.....IAHLD.....CDWGL.....RSDIF......
149196907   ......DVVMS.....IATAY.....FLLMD.....FIYRD.....VDFGL.....RSDMY......
149197003   ......QITAS.....IIPIY.....YYAME.....ILHLD.....IDWGL.....QTDIY......
149195856   ......DVFTS.....LLPLE.....GLITP.....FLYPD.....CDYGH.....RSSLY......
149198461   ......RLTAL.....IISVY.....YFTME.....VLHLD.....CDWGL.....QTDIY......
149196966   ......RICAR.....IIPVY.....YFTME.....VLHLD.....IDWGL.....QTDIY......
149196399   ......RLTSL.....IISVH.....YFTME.....VIHLD.....CDWGL.....LCDIY......
149375292   ......WIARR.....VLNAG.....YTVTE.....ILHQD.....IDFGS.....RSDLY......
149376247   ......YILEQ.....VPRLV.....ILVME.....IVHGD.....IDWGS.....RFDEQ......
149707975   ......HYMRS.....LVDLH.....LLIME.....LVHRD.....TDLGF.....SVDVY......
154484278   ......DRLKD.....IVDVM.....VAVEE.....IVHRD.....IDFDI.....RSDIY......
149755786   ......AALEQ.....IVTLY.....CLLLE.....YVHAD.....IDFGL.....AVDLW......
149909444   ......WIGSR.....ILKIV.....YYLME.....TLHQD.....IDFGS.....QSDIF......
149921743   ......EALRT.....VAAVL.....YVITE.....VLHLD.....VGLGL.....GADQF......
149923441   ......RSLAS.....VVQVY.....FLAME.....VVHRD.....TDFGL.....SADVY......
149921645   ......RSLAK.....VITVY.....FVAME.....LVHRD.....LDFGL.....ASDQF......
149921658   ......RTIAR.....VVDVL.....FYAME.....LAHRD.....VDFGI.....RLDIY......
149921669   ......RLTAS.....VLTVH.....FLALE.....LVHHD.....GDFGL.....RSDQF......
149922355   ......RSVAR.....VVEFS.....FLVME.....VIHRD.....VDFGL.....RSDVY......
149921773   ......QVLQS.....VVQAL.....VLLLD.....VIHRD.....ADFGI.....RSDLY......
149924527   ......KLLAE.....VVRIH.....YLVLE.....LVHRD.....LDFGL.....QGDQF......
149922141   ......QGLAR.....VVQVY.....YIAME.....LVHRD.....MDFGL.....RSDQF......
149922683   ......QAMAK.....VVAVH.....YVAMA.....LIHRD.....MDFGL.....AADQF......
149923599   ......LAMAR.....VIDIY.....YIAME.....LVHRD.....LDFGL.....ASDQF......
```

```
149924582 ......RSQAQ.....VVPVH.....VLVME.....LVHRD.....LDFGL.....ASDQY.....
149922074 ......RLSLM.....ITRTY.....FMVME.....VIHRD.....MDFGI.....TVDIF.....
149922492 ......KLIQQ.....VVRAL.....ALVLE.....VVHRD.....ADFGI.....RSDLY.....
149922977 ......RVAAA.....VIEIY.....YLAME.....AVHRD.....LDFGL.....ASDVY.....
149924216 ......RLIQG.....VVQAL.....TLILD.....VIHRD.....ADFGI.....RSDLY.....
149921638 ......QSASR.....CVRVD.....YLVME.....IVHRD.....VDFGI.....RSDLY.....
149917071 ......RAASR.....IVDII.....YFVME.....VIHRD.....LDFGI.....RADIY.....
149917746 ......QGMAR.....VVSVF.....WIAME.....LVHRD.....ADFGL.....RSDQF.....
149920831 ......KALAA.....IVKIF.....WLVLE.....LIHRD.....TDFGL.....RSDQF.....
149921206 ......KALAA.....VVSIF.....WLVLE.....LVHRD.....TDFGL.....RSDQF.....
149920619 ......QVLAK.....VVTVH.....YLVME.....IIHRD.....GDFGL.....RSDQF.....
149916881 ......RALAR.....VVQVH.....YLAME.....VVHRD.....VDFGL.....RSDQF.....
149918846 ......RIAAQ.....IASIY.....YIVLE.....LIHRD.....IDFGV.....RSDVF.....
149920422 ......QGLAR.....VVQVY.....FIVME.....LVHRD.....MDFGL.....RSDQF.....
149921369 ......RATAA.....IVDIE.....FFAME.....IVHRD.....LDFGI.....RADVY.....
149918650 ......RA-RR.....LIRPL.....FFTME.....LVHRD.....ADFGL.....PGDFY.....
149920903 ......ELIRR.....VVRAL.....ALILE.....VIHRD.....ADFGI.....RSDLY.....
149916611 ......KALAQ.....VVQIY.....YIAME.....LVHRD.....MDFGL.....RSDQF.....
149917104 ......RLLES.....VVKPI.....VLLLE.....VIHQD.....ADFGI.....RSDLY.....
149918588 ......HLLQG.....VVRPI.....VLLLD.....IVHRD.....ADFGL.....RSDLY.....
149921161 ......RTVGS.....IVPIH.....FFVMK.....LVHRD.....MDWGI.....RSDIY.....
149921565 ......RAASA.....IVEVF.....YLVME.....IIHRD.....LDFGI.....RFDIY.....
149919270 ......QALAK.....VVTVY.....FVAME.....LVHRD.....MDFGL.....ASDQF.....
149920057 ......RAATQ.....IIDVI.....FFVME.....IIHRD.....LDFGI.....KSDIY.....
149918963 ......RPGSA.....TWPMA.....GFVMA.....HRIGD.....VDFEI.....SSDLY.....
149917410 ......RLLTQ.....LAAVY.....YFTQE.....LVHED.....IDFGL.....RSDIY.....
149921247 ......RCLAR.....VVGVH.....FIVME.....LHHGD.....VDFGL.....RSDQF.....
149921227 ......QALAR.....VVTVY.....FIAME.....LVHRD.....MDFGL.....RSDQF.....
149918660 ......RAATR.....IVTVY.....AIAME.....IVHRD.....LDFGV.....KVDVY.....
149917436 ......RLLQA.....VVRAL.....VLLLD.....VVHRD.....VDFGI.....RSDLY.....
149920394 ......QALAR.....VVGVY.....YIVME.....LVHRD.....ADFGL.....RSDQF.....
149918078 ......QALTR.....VTTVF.....VIVME.....IVHRD.....VDFGL.....AADQY.....
149919393 ......EQLTH.....VVRAL.....VLVLA.....VLHRD.....ADFGI.....RSDLY.....
149916949 ......QITGQ.....IVPVH.....FFSMK.....VVHCD.....MDWGI.....RSDIF.....
149918716 ......RLSMM.....IVQMF.....YLAME.....VLHRD.....TDFGI.....ASDLF.....
149921617 ......RVASR.....VVATV.....FLVLE.....VVHRD.....IDLGL.....RSDVF.....
149921177 ......QAMAR.....VVHVY.....FLAME.....LVHRD.....LDFGL.....QSDQF.....
149919128 ......RAMAR.....VVHIH.....YLAME.....MVHRD.....ADFGT.....KSDQF.....
149921357 ......RRLAT.....VVTVV.....FLVME.....LVHGD.....FDFGL.....RSDQF.....
149921109 ......GILAR.....VVRCL.....VMALE.....IIHRD.....MDFGI.....ASDLY.....
149918968 ......ELLKH.....LVALV.....LYAMR.....IYHLD.....ADIDI.....RRDVF.....
149918088 ......ETLNS.....VIKAY.....VLVQE.....VIHKD.....IDFGI.....RTDFY.....
149916969 ......QALAR.....VVTIH.....FVAME.....LVHRD.....LDFGL.....RSDQF.....
149920070 ......LIAQL.....LVEVL.....ALVFD.....VVHKD.....IDFGI.....RSDYY.....
149917100 ......QALAR.....IVKVY.....FMTME.....IVHRD.....VDFGL.....ASDQF.....
149917906 ......QALAR.....VVQIY.....YLAME.....LVHRD.....ADFGL.....RADQF.....
149919272 ......ALLEA.....VVKAL.....VLLLE.....VIHRD.....ADFGI.....RSDLY.....
149926000 ......RLLAQ.....LVKVY.....YMVMP.....CYHRD.....LDFGA.....QTDVY.....
157377144 ......FMLAQ.....VPPAI.....ILVMA.....LVHGD.....IDWGS.....RFDEQ.....
150384706 ......KLVAK.....VIPIY.....FLVMR.....VVHHD.....GDFDL.....RGDIF.....
150383730 ......RLNAR.....IVPVY.....EVMMK.....IMHGD.....MDWGS.....QVDIF.....
150383580 ......RVIAM.....IVKVF.....YYAME.....ILHRD.....SDFGL.....ATDQY.....
150383731 ......RLNAR.....IVPVY.....EVMMK.....IMHGD.....MDWGS.....QVDVF.....
150386165 ......RATAQ.....IVPVH.....YFSMK.....ILHCD.....MDWGM.....LSDIY.....
154498278 ......TC---.....LPTIY.....LVLEE.....AVHRD.....IDFDA.....RTDIY.....
154276764 ......A----.....LVQ--.....MFVFE.....IVHTD.....GDLED.....PSDIF.....
154278116 ......SPFLR.....I----.....IFVYR.....IVHLD.....IDLEN.....PSDVF.....
154313922 ......NAMEE.....ILKLV.....YLLLY.....VRHRD.....ADFGI.....SADVF.....
154319874 ......DR---.....ILKIY.....RIYLE.....MCHLD.....GDFGL.....QLDFC.....
154298402 ......NALHQ.....VIQFG.....CLLIS.....FVQGD.....ID---.....KSDLY.....
152981814 ......RALAR.....VVSVT.....YMVMG.....LLHLD.....LDFGA.....WTDIY.....
152967225 ......SMLRR.....VVAVH.....VLVLD.....VVHGD.....ADLGV.....ASDVY.....
152964889 ......ATLER.....VAEVL.....HLVTR.....VVHRD.....-DFGI.....ATDWW.....
109116362 ......MACAG.....IVPLY.....NIFME.....ILHGD.....CDFGH.....KVDVW.....
145592614 ......QNAAS.....IVAVY.....FIVME.....IIHRD.....MDFGI.....RSDVY.....
114629911 ......EIQAC.....IAELY.....HLFME.....VIHHD.....VDFGL.....KADIY.....
114629909 ......EIQAC.....IAELY.....HLFME.....VIHHD.....VDFGL.....KADIY.....
115374481 ......KVLVH.....IAQVY.....YMALE.....IVHRD.....IDFGL.....RSDIY.....
115372654 ......RIGAL.....IVRTL.....YLVME.....LIHRD.....IDFGI.....RSDLF.....
```

115697876	......AIHKR.....IVQLM.....MIVMQ.....ILHLD.....CDLGL.....ASDVW.....
72165717f	......SIHKR.....IVSLM.....LIVME.....ILHLD.....CDLGL.....GNDIW.....
118085619	......EIQAC.....IAELY.....HLFME.....IIHHD.....VDFGL.....KADIY.....
149642673	......EIQAC.....IAELY.....HLFME.....VIHHD.....VDFGL.....KADIY.....
119912410	......MACAG.....IVPLY.....NIFME.....ILHGD.....CDFGH.....KVDVW.....
125812979	......GTWSA.....VVELF.....VLFMD.....VLHLD.....CDFGQ.....KVDIW.....
125812973	......GTWSA.....VVELF.....VLFMD.....VLHLD.....CDFGQ.....KVDIW.....
126308357	......TACAG.....FVPLY.....NIFME.....ILHGD.....CDFGH.....KVDIW.....
126341206	......EIQAC.....IAELY.....HLFME.....VIHHD.....VDFGL.....KADIY.....
148702237	......VACAG.....IVPLY.....NIFME.....ILHGD.....CDFGH.....KVDIW.....
149177846	......QRLQG.....LVLPR.....VIISH.....VVHGD.....VDPGL.....QSDLY.....
149032577	......EIQAC.....IAELY.....HLFME.....VIHHD.....VDFGL.....KADIY.....
149054446	......IACAG.....IVPLY.....NIFME.....VLHGD.....CDFGH.....KVDIW.....
149634742	......EIQAC.....IAELY.....HLFME.....VIHHD.....VDFGL.....KADIY.....
149723683	......MACAG.....IVPLY.....NIFME.....ILHGD.....CDFGH.....KVDVW.....
149743649	......EIQAC.....IAELY.....HLFME.....VIHHD.....VDFGL.....KADIY.....
149918013	......RVLQG.....VPDFI.....YSVQA.....VIHRD.....VDFDL.....ATDLY.....
149920390	......EVLRR.....LPEAL.....WLVTA.....THHGD.....TD---.....AADRF.....
152967176	......KSAAR.....VVAVL.....YLVME.....VLHRD.....ADFGL.....RSDVY.....
157132902	......SIMKK.....IVKLI.....VVIME.....IIHRD.....SDFGL.....KSDVW.....
157117156	......DLFRT.....VVKLF.....YMLLE.....IIHKD.....SLPGL.....KSDIF.....
157110006	......AMMKQ.....IVALL.....TMIME.....ITHRD.....SDFGL.....KSDVW.....
157103615	......KIMVH.....VVNLL.....MVIVE.....VLHGD.....CDFGL.....YSDVW.....
157128577	......EVMKS.....IVCII.....MLLTE.....VVHRD.....SDFGL.....QSDVW.....
157110899	......QVLKS.....VVRLL.....FVILE.....IIHRD.....ADFGF.....KSDIW.....
109071259	......EMFGK.....VVRLL.....YMVLE.....FVHKD.....SALGL.....KSDVW.....
109095632	......QFHQY.....LVQLE.....YMVLE.....LFHGD.....CGLGL.....RADVW.....
109472732	......QFYQY.....LVQLE.....YMMLE.....LFHGD.....CHLGL.....RGDVW.....
109474350	......QFYQY.....LVQLE.....YMMLE.....LFHGD.....CHLGL.....RGDVW.....
110758654	......EIMMA.....LVSLI.....CILLE.....ITHRD.....SDFGL.....KSDVW.....
110761667	......EIMQQ.....VVTLL.....YLILE.....IIHRD.....ADFGM.....KTDVW.....
110766302	......TIMKT.....VVKIL.....CLVME.....IVHRD.....SDFGL.....RSDVW.....
110776976	......RVMKN.....VVRLL.....FVILE.....IIHRD.....ADFGF.....KSDIW.....
114607487	......EMFGK.....VVRLL.....YMVLE.....FVHKD.....SALGL.....KSDVW.....
115649663	......EMLGK.....IVKLL.....LMITE.....FIHGD.....CTMSV.....KSDVW.....
115675405	......RLMIE.....VLEVI.....YMVTE.....FYHGD.....SDFGL.....QSDIW.....
115704746	......KLMID.....ILQII.....YLITE.....YYHGD.....SDFGL.....QSDVW.....
115744182	......SLYMY.....IVNIL.....YIIME.....CIHRD.....SDFGL.....QSDVW.....
115898435	......ELMKE.....VVSLL.....CLLVE.....FVHRD.....GDFGL.....KGDVW.....
115936379	......SLYMY.....IVNIL.....YIIME.....CIHRD.....SDFGL.....QSDVW.....
115971070	......EMLGK.....IVKLL.....LMITE.....FIHGD.....CTMSV.....KSDVW.....
118788966	......DIMKN.....IVEFI.....VVVME.....IVHRD.....SDFGL.....QSDVW.....
118083403	......KFHQN.....LVELV.....YMIME.....LFHGD.....CGFGL.....KADIW.....
119892990	......QFYLY.....LVQLE.....YMVLE.....LFHGD.....CGLGL.....KGDIW.....
119624564	......EMFGK.....VVRLL.....YMVLE.....FVHKD.....SALGL.....KSDVW.....
119915291	......EMFGK.....VVRLL.....YMVLE.....FVHKD.....SALGL.....KSDVW.....
124784421	......ALLKD.....LLTLI.....YVVTE.....IQHRD.....GDFGL.....RSDVW.....
125837834	......SIMRK.....VMQLL.....LLIME.....VVHCD.....AEFGL.....KGDVW.....
125846435	......DMFAK.....VARLL.....YMIME.....FVHKD.....SSLGL.....KTDVW.....
125852178	......LFHAA.....IVKLL.....YLLLE.....VIHGD.....SGLDF.....RSDVW.....
126015619	......ELMHS.....LLNLI.....MIIVE.....CVHRD.....ADFGL.....ESDVW.....
126310050	......EMFGK.....LVRLL.....YMILE.....FVHRD.....SSLGL.....KSDVW.....
126340163	......HFHHF.....LVQLE.....YMVLE.....LFHGD.....CGLGL.....RGDVW.....
148691564	......EMFGK.....VVRLL.....YMVLE.....FVHKD.....SALGL.....KSDVW.....
148688937	......QFYQY.....LVQLE.....YMMLE.....LFHGD.....CHLGL.....RGDIW.....
148691565	......EMFGK.....VVRLL.....YMVLE.....FVHKD.....SALGL.....KSDVW.....
153792686	......DMFSK.....VVRLL.....YIVLE.....FVHKD.....SALGL.....KSDVW.....
157818047	......EMFGK.....VVRLL.....YMVLE.....FVHKD.....SALGL.....KSDVW.....
149713723	......QFYQY.....LVQLE.....YMVLE.....LFHGD.....CGLGL.....RGDIW.....
149732548	......EMFGK.....VVRLL.....YMVLE.....FVHKD.....SALGL.....KSDVW.....
154284247	......RFFKD.....IID--.....HIFLS.....FCHND.....IDFGR.....RTEQF.....
115450869	......RIMRR.....IVLFM.....SIVSE.....IVHRD.....CDFGL.....KCDVY.....
108862829	......SLMKK.....ILLFM.....CIVTE.....IIHRD.....ADFGL.....KSDVY.....
110180226	......SLMKR.....VLLFM.....CIVTE.....IIHRD.....ADFGL.....KSDVY.....
110180242	......AIMKR.....VVLFM.....SIITE.....VVHWD.....CDFGL.....KSDVY.....
110180234	......SILSR.....VVLFL.....SMITE.....IVHRD.....CDFGL.....KCDIF.....
110180228	......AIMKR.....IVLFM.....SIVTE.....IVHRD.....CDFGL.....KSDVY.....
110180240	......SLMQR.....VLLFM.....CIVSE.....IIHRD.....ADFGL.....KSDIY.....
110180238	......SLMKR.....VLLFM.....CIVSE.....IIHRD.....ADFGL.....KSDIY.....
110739818	......RIMKK.....IVLFM.....SIVTE.....IVHRD.....CDFGL.....KCDVY.....
111220616	......HAALR.....VAAVL.....WMVTE.....VVHRD.....IDFGV.....SADIF.....

```
10177613    ......SLMKR......VLLFM......CIVTE......IIHRD......ADFGL......KSDVY......
115467252   ......QIMKR......VVLFM......SIVTE......VVHRD......CDFGL......KCDVF......
115480725   ......AIMKS......IVLFM......SIVTE......IVHRD......CDFGL......KSDVY......
115484411   ......NALRK......LVPIL......ALVYK......VVHCD......GDFGL......AADVY......
115891550   ......SIHKR......IVSLM......LIVME......ILHLD......CDLGL......KADIW......
116054989   ......SFLSR......IVQFI......CIVMD......IIHRD......GDFGL......AVDVY......
116056048   ......EIMRA......IVKIF......CLILQ......IIHRD......ADFGV......KVDVY......
116057496   ......SIMTK......IVLLL......AIVQE......VLHRD......GDFNL......ASDVY......
116057812   ......QVLAK......LLLFM......C--TE......ILHLD......ADFGL......HADSY......
116060744   ......DIMRR......VVLLM......SIVTE......IVHRD......CDFGL......KADVW......
116309972   ......AIMKR......VVLFM......SIVTE......IVHWD......GDFGL......KCDVY......
116643218   ......RIMKK......IVLFM......SIVTE......IVHRD......CDFGL......KCDVY......
116643214   ......EIMLR......VVLFM......SILTE......VVHRD......CDFGL......KCDVY......
116643208   ......NIMKK......VLLFM......AIIME......IVHRD......GDFGL......KCDVF......
116643212   ......LLMKR......VLLFM......CIVSE......IIHRD......ADFGL......KSDIY......
116643206   ......RMMRR......IVLFM......SIVTE......IVHRD......CDFGL......KCDVY......
116643210   ......SLMKR......VLLFM......CIVTE......IIHRD......ADFGL......KSDVY......
116643250   ......TLFEK......VVQFV......MIVSE......VIHCD......AGFGL......SVDSY......
116643262   ......SMLSR......LVKFI......VIVTE......VIHRD......ADFGL......KVDAY......
116643264   ......EMLSR......LVKFI......VIVTE......IIHRD......ADFGL......KVDAY......
116643260   ......AMLSK......LVKFI......VIVTE......IIHRD......ADFGL......KVDAY......
116643266   ......NMMSR......LVKFI......VIVTE......IIHRD......ADFGL......KVDVY......
116643272   ......LLLSK......IVRFI......MIITE......IIHRD......ADFGL......KVDVY......
116643288   ......AVWHK......VTRFV......CVVVE......IVHRD......ADFGV......RCDVY......
116643280   ......SMLAN......IVRFI......CIVTE......FIHRD......ADFGV......KVDVY......
116643278   ......ALLSR......IVQFI......CIITE......VIHRD......ADFGT......KVDVY......
116643290   ......TVWHK......VTKFV......CVVVE......IVHRD......ADFGV......RCDVY......
121592911   ......TILPT......APRFV......YLVME......VCHLD......LDFGL......RSDIF......
121488653   ......TIMKR......IVLFM......SIVTE......IVHRD......CDFGL......KSDVY......
123402768   ......NTLAS......LLQFV......CVVTK......IIHRD......CDFGS......KIDVY......
123407927   ......AVLAV......LIRFI......TIITE......IVHRD......CDFGF......KIDVY......
123423246   ......EVLTK......CLNLI......IIVTE......IIHRD......IDFGL......KVDVY......
123436047   ......MVMSK......VIELT......QIITR......VVHRD......ADFGL......LVDVY......
123436283   ......KTMAK......LVPFV......AIVSE......IIHRD......ADFGI......KVDVY......
123438259   ......QILSK......LLGFV......IIVTE......IMHRD......IDFGL......KIDVY......
123456922   ......AVLAN......LLHLV......CIIME......IVHRD......CDFGF......KIDVY......
123974670   ......KVMSL......VVPFV......TIITR......IIHRD......CDFGL......KVDVY......
123456628   ......KTMSQ......LVPFV......AIITE......IIHRD......GDFGI......KVDVY......
123458299   ......ETLAR......LVPFI......TIVTE......IIHRD......CDFGI......KVDVY......
123472710   ......SVLAT......LIGFV......CIVTE......IIHRD......CDFGF......KVDVY......
123472125   ......DALMR......VIKLI......CIVTT......LIHRD......CDFGL......SVDVY......
123475114   ......FTTAS......IVKLV......SIIAE......LIHRD......IDFGI......SVDVF......
123488916   ......TVLAT......VLGFV......CIVTE......IVHRD......GDFGF......KVDVY......
123478661   ......SILAA......LLKFV......CIITE......IIHRD......GDFGY......KVDVY......
123485635   ......EVLIR......CLPIV......SIITQ......IIHRD......CDFGL......KIDVY......
123479543   ......ETMLK......LIPLV......TIITE......IIHRD......IDFGI......KVDVY......
123479850   ......ETMAK......LVPFV......SIITE......IIHRD......CDFGI......KVDVY......
123495711   ......SSCKF......INKKL......FTIMD......VYHPD......IDF--......KSGMY......
123504599   ......AVLAT......LLRLI......CIITE......IVHRD......CDFGF......KVDVY......
123493591   ......RALAR......VNKLI......SIITE......IIHRD......IGFGI......QSEMY......
123493767   ......TVLAS......LVQLV......CIVTK......FVHRD......CDFGI......KVDVY......
154413981   ......KTMAK......LLPIS......SIITP......IIHRD......CDFGI......KVDVY......
154416211   ......SVLAS......LLRFV......CIVTE......IIHRD......IDFGL......KVDVY......
154420500   ......KILAC......LVPLL......LIITE......IIHRD......IDFGI......KVDVY......
154420153   ......RTMAR......LTKLI......SIITE......ILHRD......CDFGI......KADVY......
154412690   ......QTLAA......FVKFL......SILTE......IIHRD......CDFGI......PFDVY......
154415149   ......TTMAK......FLKLI......VIITE......IIHRD......CDFGV......EVDVY......
124359937   ......RSMKR......ILLFM......CIVTE......IIHRD......GDFGL......KSDVY......
145485335   ......HAMEA......IVMFL......AIVLE......ILHRD......ADFGW......KADVF......
145487588   ......QVISE......IVLYM......YLITE......IMHCD......CDFGL......KSDVF......
145491081   ......EVISN......IVLYM......YLITE......VMHCD......CDFGL......KADVY......
145492632   ......HAMEA......IVMFL......AIVLE......ILHRD......ADFGW......KADVF......
145501912   ......HAMEA......IVMFL......AIVLE......ILHRD......ADFGW......KADVF......
145505976   ......QVISN......IVLYM......YLITE......IMHCD......CDFGL......KSDVY......
145511191   ......SALVT......LVQLL......YIVTE......LIHRD......ADFGL......KADVY......
145522654   ......EVISN......IVLYM......YLITE......VMHCD......CDFGL......KSDIY......
145521985   ......EVISN......IVLYM......YLITE......VMHCD......CDFGL......KADVY......
145528195   ......TLVKV......LVCLI......YIITE......FMHRD......ADFGL......KADVY......
145546588   ......QVISN......IVLYM......YLITE......IMHCD......CDFGL......KSDVY......
114229341   ......TIMKR......IVLFM......SIVTE......IVHRD......CDFGL......KSDIY......
114229339   ......TIMKR......IVLFM......SIVTE......IVHRD......CDFGL......KSDIY......
114229343   ......TIMKR......IVLFM......SIVTE......IVHRD......CDFGL......KSDIY......
125537325   ......AVWHE......ITKFI......CVVVE......IVHRD......ADFGV......KCDVY......
125537032   ......SLMKK......ILLFM......CIVTE......IIHRD......ADFGL......KSDVY......
```

```
125538650    ......GIMSR.....VVLFL.....SILTE.....IVHRD.....SDFGM.....KCDVY.....
125531041    ......SSIGG.....LVRLI.....FLVYE.....ILHRD.....ADFGL.....SSDMY.....
125532044    ......RIMRR.....IVLFM.....SIVSE.....IVHRD.....CDFGL.....KCDVY.....
125542492    ......RIMRR.....IVLFM.....SIVSE.....IVHRD.....CDFGL.....KCDVY.....
125541105    ......RIIKR.....VVLFM.....SIVTE.....IVHRD.....CDFGL.....KCDVF.....
125546197    ......NMMCK.....LVKFI.....VIVSE.....IIHRD.....TDFGL.....KVDVY.....
125554654    ......QIMKR.....VVLFM.....SIVTE.....VVHRD.....CDFGL.....KCDVF.....
125560745    ......DALRR.....LVKVI.....ALVLE.....IVHCD.....TDFGL.....DGDIY.....
125570736    ......QLLCE.....LARLV.....LMFFD.....IVHRD.....ADFGL.....KSDVY.....
125579732    ......SLMKK.....ILLFM.....CIVTE.....IIHRD.....ADFGL.....KSDVY.....
125584997    ......RIMRR.....IVLFM.....SIVSE.....IVHRD.....CDFGL.....KCDVY.....
125581337    ......GIMSR.....VVLFL.....SILTE.....IVHRD.....SDFGM.....KCDVY.....
125591596    ......AIMKR.....VVLFM.....SIVTE.....IVHWD.....GDFGL.....KCDVY.....
125606670    ......AIMKS.....IVLFM.....SIVTE.....IVHRD.....CDFGL.....KSDVY.....
125606506    ......YIMRK.....VVQFI.....CIVTE.....IIHRD.....ADFGV.....KADVF.....
145357121    ......SFLTR.....IVQFI.....CIIMD.....IIHRD.....GDFGL.....AVDVY.....
145352577    ......DIMRR.....VILLM.....SIVTE.....IVHRD.....CDFGL.....KADIY.....
145352844    ......GLMLE.....VLGVV.....ELFFP.....VVHRD.....GDFGL.....GADVY.....
146164160    ......NMLAT.....IILLM.....CIVTD.....IVHRD.....CDFGL.....KVDVF.....
147771926    ......KILQK.....LVRLE.....YLVYE.....VVHKD.....ANFGL.....AVD--.....
147781068    ......AIMKR.....VVLFM.....SIVTE.....IVHWD.....CDFGL.....KSDVY.....
147820054    ......RIMKR.....VVLFM.....SIVTE.....IVHRD.....CDFGL.....KCDVF.....
147827145    ......SLMKR.....VLLFM.....CIVTE.....IIHRD.....GDFGL.....KSDVY.....
147839113    ......ALLFR.....IITFV.....CIITE.....ILHRD.....ADFGI.....KVDVY.....
147856467    ......EILGT.....IVKLL.....LLVYE.....TIHRD.....TDFGL.....KIDVY.....
147862394    ......ASIGR.....LVRLY.....ALVYE.....IIHYD.....ADFGL.....KCDVY.....
147862319    ......SCLSR.....VIKFV.....VVITE.....IIHRD.....ADFGI.....KVDVY.....
156082231    ......NTYRS.....IVKYY.....GIVLE.....FVHRD.....CDFGK.....KSDIW.....
156093779    ......NILST.....VSLFL.....-IALE.....ILHCD.....CDFGL.....QADVY.....
157130761    ......RTWAE.....LVRMK.....VLVME.....LVHAD.....IDVSQ.....KKDVS.....
157128409    ......RISAT.....MKALY.....CVIME.....VIHGD.....IDFPQ.....KFGYE.....
109069559    ......KTWAE.....LIRLN.....MLRSH.....LVHAD.....IDVSQ.....KHSVA.....
109069561    ......KTWAE.....LIRLN.....MLRSH.....LVHAD.....IDVSQ.....KHSVA.....
109069563    ......KTWAE.....LIRLN.....MLRSH.....LVHAD.....IDVSQ.....KHSVA.....
169613122    ......KMWAD.....LARIY.....VLVME.....LVHAD.....IDVSQ.....SRNVE.....
115384436    ......KLWAE.....LRRIY.....VMVMG.....LVHAD.....IDVSQ.....RKSVD.....
115387539    ......RLAAM.....MKALR.....AIVMS.....LIHGD.....IDFPQ.....RKFHF.....
114605361    ......KTWAE.....LIRLN.....VLVMS.....LVHAD.....IDVSQ.....KHSVA.....
114605359    ......KTWAE.....LIRLN.....VLVMS.....LVHAD.....IDVSQ.....KHSVA.....
115647172    ......RTWAE.....LLRLH.....VLVMD.....LVHAD.....IDVSQ.....KNGVC.....
117617010    ......RLSAM.....MKALY.....AVIME.....LIHGD.....IDFPQ.....KRFSY.....
117617008    ......RTWAE.....LCRLK.....VLLMG.....LVHAD.....IDVSQ.....KHAVA.....
118086426    ......KTWAE.....LIRLN.....VLLMG.....LVHAD.....IDVSQ.....KHNVA.....
121716268    ......KLWAE.....LRRIY.....VLVMG.....LVHAD.....IDVSQ.....RQGVE.....
119481679    ......KLWAE.....LRRIY.....VLVMG.....LVHAD.....IDVSQ.....RKGVE.....
119936397    ......KTWAE.....LIRLN.....VLVMS.....LVHAD.....IDVSQ.....KHGVA.....
154418263    ......KLWSE.....LSRLH.....IIFME.....LVHAD.....IDVGQ.....SRGVK.....
145232344    ......KLWAE.....LRRIY.....VLVMG.....LVHAD.....IDVSQ.....RKNVD.....
145609517    ......KLWAE.....LRRIY.....VLVMS.....LVHAD.....IDVSQ.....RKGVD.....
145339355    ......RLAAL.....MKALE.....CVIMS.....LIHCD.....IDFPQ.....KRFNM.....
146415444    ......KMWAE.....LKRLY.....VLVMQ.....LVHAD.....IDVSQ.....KKINV.....
149246902    ......KIWAE.....LNRIY.....VIVME.....LVHAD.....IDVSQ.....KSGIQ.....
148688555    ......RLSAM.....MKALY.....AVIME.....LIHGD.....IDFPQ.....KRFSY.....
156098157    ......KIAAT.....LKALH.....MILMS.....IIHGD.....IDFPQ.....KYKIK.....
150864425    ......KVWAE.....LKRLY.....VLVME.....LVHAD.....IDVSQ.....RKKIN.....
149638620    ......RTWAE.....LIRLN.....VLVMG.....LVHAD.....IDVSQ.....KHAVA.....
149726482    ......RLSAM.....MKALY.....AVVME.....LIHGD.....IDFPQ.....KRFSY.....
149731782    ......KTWAE.....LIRLN.....VLVMG.....LVHAD.....IDVSQ.....KRGVA.....
55661618     ......KTWAE.....LIRLN.....VLVMS.....LVHAD.....IDVSQ.....RHSVA.....
```
