# Supplementary material for: Deciphering the Structural Basis of Eukaryotic Protein Kinase Regulation
Source: PLoS Biol. 2013 Oct 15;11(10):e1001680. doi: 10.1371/journal.pbio.1001680 (PMC3797032; doi:10.1371/journal.pbio.1001680)
Supplement: Table S2 — Radioactive phosphoryl transfer assay of different PKA mutants. The activity is represented by the percent of catalytic activity of each mutant relative to the WT-PKA for triplicate experiments using a radioactive phosphoryl transfer assay and the standard error of each mutant. (PDF) [file pbio.1001680.s005.pdf]

	Activity (%)	Relative Standard Deviation
WT	100.00	10.42
E91A	1.83	0.26
RS1N	2.15	0.46
RS2N	0.57	0.02
RS3N	84.50	6.34
RS4N	96.83	10.55
RS1G	0.17	0.12
RS2G	0.21	0.07
RS3G	97.58	15.13
RS4G	92.65	2.42
RS3G	97.58	7.56
RS3G/Sh2A	3.63	2.44
Sh1G	5.73	1.83
RS4G/Sh2A/Sh3A	0.03	0.11
RS4G/Sh3A	13.66	4.18
RS4G/Sh3A/RS3G	23.69	6.72
RS4G/Sh2A	47.21	10.52
RS3G/Sh2A	3.63	2.44
Sh2A/Sh3A	0.06	0.06
RS1N + PDK1	43.59	3.99
RS1N/H87A + PDK1	19.75	5.41
RS2N + PDK1	75.42	4.50
RS2N/H87A + PDK1	3.34	1.16
RS3N + PDK1	92.65	2.42
RS3N/H87A + PDK1	25.13	8.84
RS4N + H87A	39.61	0.15
