# Supplementary material for: Deciphering the Structural Basis of Eukaryotic Protein Kinase Regulation
Source: PLoS Biol. 2013 Oct 15;11(10):e1001680. doi: 10.1371/journal.pbio.1001680 (PMC3797032; doi:10.1371/journal.pbio.1001680)
Supplement: Table S3 — Summary of different conformations of the 172 Apo EPK structures. Number of EPK structures that belong to the active and four inactive conformations and the PDB IDs of the structures belonging to each group. (PDF) [file pbio.1001680.s006.pdf]

Group	Number of Apo Structures	PDB ID
Active	118	1CKI, 1ERK,1FGK, 1FOT, 1GZO, 1H24, 1H25, 1H26, 1H27, 1H28, 1H4L, 1JAM, 1JKS, 1JKT, 1JOW, 1K9A, 1KOA, 1M14, 1NA7, 1OKV, 1OKW, 1OKW, 1OL1, 1OL2, 1OMW, 1Q62, 1R1W, 1SNX, 1SYK, 1TKI, 1URC, 1VR2, 1WMK, 1WVW, 1XBA, 1XQZ, 1Y0J, 1Y16, 1Y8G, 1YHV, 1YHW, 1YM7, 1Z9X, 1ZMU, 1ZMV, 1ZMW, 1ZWS, 1ZYC, 2A1A, 2BDW, 2BFX, 2CPK, 2DYL, 2EB2, 2ERK, 2EXE, 2FYS, 2G15, 2GNG, 2GPH, 2GS2, 2GSF, 2HEL, 2JGZ, 2JIT, 2KTY, 2KUL, 2LAV, 2O8Y, 2OIB, 2ONL, 2OZA, 2QG5, 2QKW, 2QOD, 2QOL, 2QOO, 2QVS, 2R0I, 2RFE, 2V5Q, 2W5H, 2W99, 2WFY, 2WHB, 2WMA, 2YHV, 2ZV7, 3A7F, 3A7G, 3AGM, 3BU3, 3BU6, 3C4Y, 3COI, 3COM, 3D5U, 3D5V, 3D7U, 3DAE, 3DBQ, 3DGK, 3E3P, 3FY2, 3FZO, 3GOP, 3HGK, 3HYH, 3IEC, 3KK9, 3KN6, 3KXX, 3MFT, 3PSC, 3PY3, 3Q6U, 3QD2, 3SDM
Inactive I – DFG Out	19	1BI7, 1BI8, 1FVR, 1G3N, 1GZK, 1GZN, 1LUF, 1M7N, 1MRV, 1MRY, 1P4O, 1RJB, 1T45, 2H6D, 2OGV, 2RF9, 2YZA, 3MN3, 3QA8
Inactive II – αC-helix Out	20	1A06, 1BUH, 1FMK, 1HCL, 1K2P, 1OB3, 1PW2, 1V0B, 2BVA, 2I6L, 2PTK, 2W9F, 2W9Z, 3BBW, 3DXN, 3G33, 3GBZ, 3I6U, 3I6W, 3P08
Inactive III – HRD Out	2	2HAK, 3H4J
Inactive IV – Twisted lobe	13	1LEW, 1LEZ, 1P14, 1P38, 1UKH, 1W98, 1WFC, 2FH9, 2OKR, 2QNJ, 2WZJ, 3FE3, 3P4K,
**Total**	**172**	
