# Supplementary material for: Deciphering the Structural Basis of Eukaryotic Protein Kinase Regulation
Source: PLoS Biol. 2013 Oct 15;11(10):e1001680. doi: 10.1371/journal.pbio.1001680 (PMC3797032; doi:10.1371/journal.pbio.1001680)
Supplement: Table S4 — Amino acid numbers of each R-spine and Shell residues for the representatives of each conformation. A list of the R-spine and Shell residues for PKA, AKT, Src, AMPK, and P38-MAPK. (PDF) [file pbio.1001680.s007.pdf]

	PKA	AKT	Src	AMPK	P38-MAPK
RS0	D220	D332	D444	D213	D205
RS1	Y164	Y273	H384	H154	H148
RS2	F185	F294	F405	F175	F169
RS3	L95	L204	M314	L86	L75
RS4	L106	L215	L325	L97	L86
Sh1	V104	T213	V323	I95	I84
Sh2	M120	M229	T338	I111	T106
Sh3	M118	F227	I336	M109	L104
